# Azoarene activation for Schmidt-type reaction and mechanistic insights

**DOI:** 10.1038/s41467-022-35141-4

**Published:** 2022-12-01

**Authors:** Fan-Tao Meng, Ya-Nan Wang, Xiao-Yan Qin, Shi-Jun Li, Jing Li, Wen-Juan Hao, Shu-Jiang Tu, Yu Lan, Bo Jiang

**Affiliations:** 1 School of Chemistry & Materials Science, Jiangsu Normal University, Xuzhou, 221116 P. R. China; 2 College of Chemistry and Institute of Green Catalysis, Zhengzhou University, Zhengzhou, Henan 450001 China

**Keywords:** Synthetic chemistry methodology, Homogeneous catalysis, Reaction mechanisms

## Abstract

The Schmidt rearrangement, a reaction that enables C-C or C-H σ bond cleavage and nitrogen insertion across an aldehyde or ketone substrate, is one of the most important and widely used synthetic tools for the installation of amides and nitriles. However, such a reaction frequently requires volatile, potentially explosive, and highly toxic azide reagents as the nitrogen donor, thus limiting its application to some extent. Here, we show a Schmidt-type reaction where aryldiazonium salts act as the nitrogen precursor and *in-situ-*generated cyclopenta-1,4-dien-1-yl acetates serve as pronucleophiles from gold-catalyzed Nazarov cyclization of 1,3-enyne acetates. Noteworthy is that cycloketone-derived 1,3-enyne acetates enabled ring-expansion relay to access a series of 2-pyridone-containing fused heterocycles, in which nonsymmetric cycloketone-derived counterparts demonstrated high regioselectivity. Aside from investigating the scope of this Schmidt-type reaction, mechanistic details of this transformation are provided by performing systematic theoretical calculations.

## Abstract (web summary)

The Schmidt reaction enables nitrogen insertion across an aldehyde or ketone substrate by frequently using azide reagents. Here, the authors show a Schmidt reaction of aryldiazonium salts as a replacement of azide reagents to access skeletally diverse cyclic lactams.

## Introduction

**Fig. 1:**
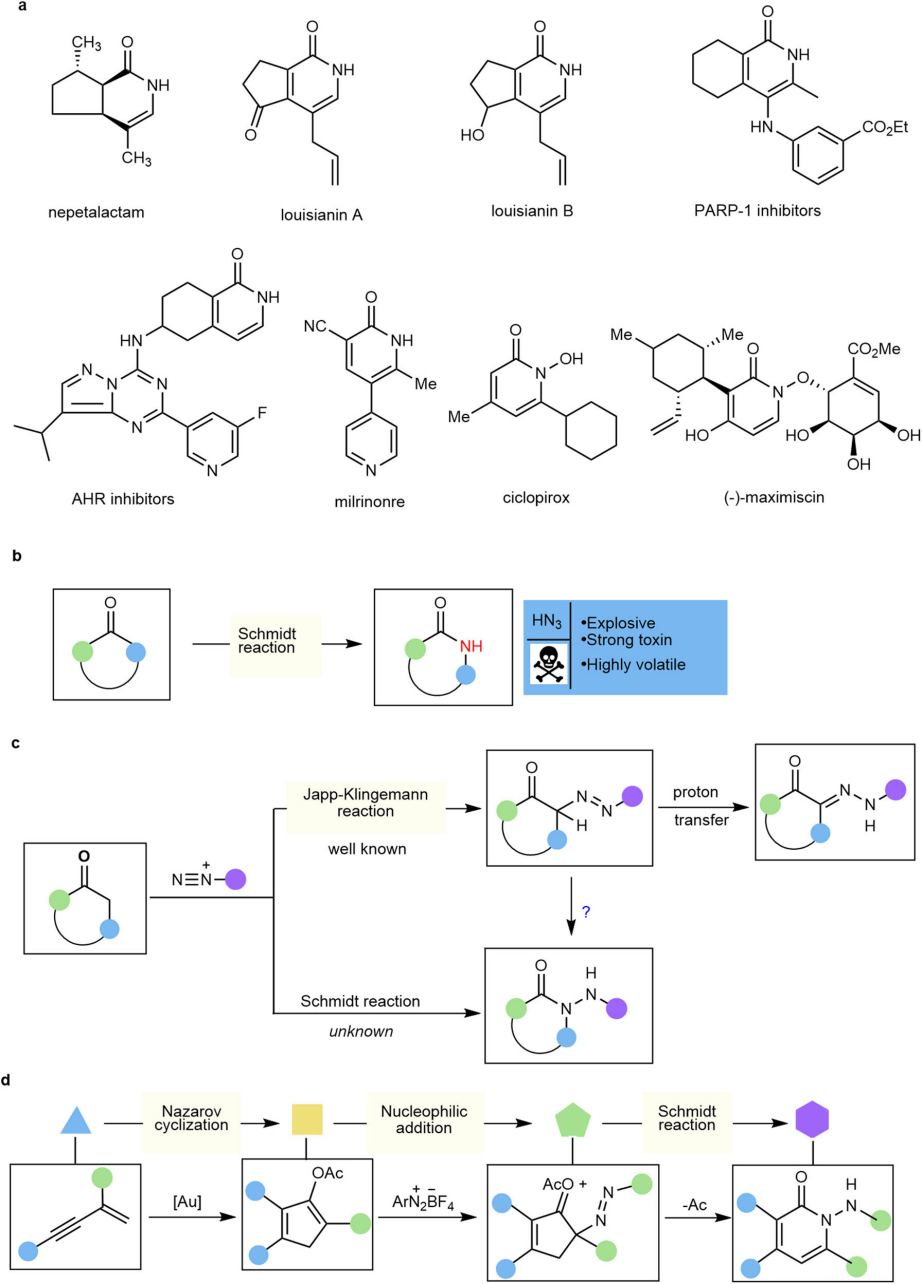
Azoarene activation for the Schmidt reaction. **a** Natural products and bioactive molecules containing 2-pyridinone structures. **b** Traditional Schmidt reaction with azides as the limiting reagent. **c** Reactivity patterns of aryldiazonium salts. **d** Our study on 2-pyridinone synthesis via the sequence involving Nazarov cyclization, nucleophilic addition, and Schmidt reaction.

Nitrogen-based heterocycles constitute the basic structural scaffold of substantial bioactive natural products and are present in approximately half of marketed drugs, with nonplanar heterocycles featuring five- and/or six-membered ring frameworks being of great interest to the fields of pharmaceutical discovery, chemical biology, and medical chemistry^1–5^. Especially abundant among these heterocycles are those incorporating a 2-pyridinone framework, a privileged structure that is composed of the core of many alkaloids exhibiting potent biological activities (Fig. 1a)^6–11^. Consequently, the development of innovative strategies for the assembly of 2-pyridone-containing heterocycles has been a hot research topic in the organic community^12–14^. The Schmidt reaction represents a long-standing popular nitrogenation approach to furnish these aza-heterocycles from cyclic ketones with HN_3_ or alkyl azides^15–19^. However, its dependence on volatile, highly toxic, and potentially explosive azide reagents offers great possibilities for further exploration^20–22^ (Fig. 1b). Despite the astonishingly persistent efforts on hydrazoic acid replacements, the use of azide still prevails in this field^23–25^. The Beckmann rearrangement starting from cyclic oximes has been recognized as a well-known alternative pathway to approach cyclic amides^26,27^. However, typically catalytic Beckmann rearrangements often require some specialized active cyclic oximes, strong Brønsted acids, or preactivation of the hydroxyl (e.g., by tosylation)^28–31^, thus limiting the potential application to some extent. Ideally, if one easily available and widespread chemical could be activated by a suitable catalytic system while endowed with new reactivity^32–37^, it would advance innovative chemical technology that influences broad fields of academia and industry.

Because of their highly electrophilic character and moderate oxidizability, aryldiazonium salts, easily prepared from the corresponding anilines, demonstrate broad-spectrum reactivities and are often used as both aryl precursors and dinitrogen proelectrophiles in numerous transformations, such as the Meerwein arylation^38,39^, cross-coupling reactions^40–42^, [3 + 2] annulation^43,44^, and Japp-Klingemann reaction^45–47^. In the well-known Japp-Klingemann procedure, the addition of cyclic ketones into aryldiazonium salts produces rather unstable azo compounds, which are converted into hydrazones via 1,3-hydrogen transfer (Fig. 1c)^45–47^. In contrast, the reactivity of aryldiazonium salts as a nitrogen donor for the Schmidt reaction remains unknown. We hypothesized that the key to realizing the Schmidt reaction is to suppress the 1,3-hydrogen transfer process in the Japp-Klingemann procedure. Therefore, the removal of the proton at the α-position of azoarenes for migration while exploiting the intrinsic nucleophilicity of azoarenes may provide a feasible scheme for the Schmidt reaction.

Homogeneous gold catalysis has been acknowledged as an effective strategy for constructing functional molecules in synthetic chemistry benefited from the impressive characteristics of its high catalytic capabilities, high levels of regioselectivity, and mild reaction conditions as well as good functional group tolerance^48–53^. The gold-catalyzed Nazarov cyclization of nucleophilic 1,n-enyne acetates toward cyclopenta-1,4-dien-1-yl acetates is among the more exploited reactions^54–61^. However, to date, there are only a few reports that deal with the inherent nucleophilicity of in-situ generated cyclopenta-1,4-dien-1-yl acetates toward intermolecular nucleophilic substitution reactions^55,58^. In sharp contrast, the nucleophilic addition of this intermediate remains elusive. Along this line, we believe that highly electrophilic aryldiazonium salts could be trapped by in-situ-generated nucleophilic cyclopenta-1,4-dien-1-yl acetates to access the azo intermediate, which facilitates acyl transfer and the subsequent rearrangement process for the Schmidt-type reaction (Fig. 1d). Thus, this cascade activation strategy would demonstrate simple and available aryldiazonium salts as a cyclic nitrogenated partner akin to azides, but with remarkable elimination of associated hazards.

In this work, we report that gold-catalyzed transformation of 1,3-enyne acetates with aryldiazonium salts enables merging Nazarov cyclization with a Schmidt-type reaction for the general synthesis of valuable 2-pyridinone-based heterocyclic systems as well as synthetically appealing azasteroid chemistry (Fig. 1d). Of note is that the gold complex could be compatible with aryldiazonium salts without observation of their redox transformation with each other. Intrigued by the ring-expansion relay and high regioselectivity observed in nonsymmetric cycloketone-derived 1,3-enyne acetates, we carefully performed systematic theoretical calculations to elucidate the catalytic cycle for σ-bond migration, the azoarene activation mode and the influence on the regioselectivity (see the mechanism section and Supplementary Information).

## Results

### Reaction optimization

To test our initial hypothesis, we began by studying the gold-catalyzed reaction of 1,3-enyne acetates and aryldiazonium salts as test substrates to develop straightforward access to highly functionalized cyclopenta[*c*]pyridin-1-ones, which are classically synthesized via catalytic cyclization reactions that exhibit a lack of flexibility^62–64^. Diverse gold catalysts and solvents were evaluated at room temperature for 6 h, as shown in Table 1. To our delight, by using JohnPhosAu(MeCN)SbF_6_ (1.0 mol %) as a catalyst, the reaction of strained cyclobutanone-derived 1,3-enyne acetate **1a** with **2a** in 1,2-dichloroethane (DCE) afforded cyclopenta[*c*]pyridin-1-one product **3** in 65% yield through a Schmidt-type reaction (entry 1). Several other gold catalysts widely used in catalytic transformations, such as Ph_3_PAuCl, XPhosAuCl, SPhosAuCl, IPrAuNTf_2_, AuCl_3_, and AuCl, were then examined (entries 2–7). The results revealed that all these catalysts could drive the transformation to the desired product **3** except for SPhosAuCl, and the former two led to moderate yields of product **3** (entries 2–3); in contrast, the latter three demonstrated very poor catalytic capabilities and provided lower yields (<29%, entries 5–7). Taking JohnPhosAu(MeCN)SbF_6_ as the catalyst, we then investigated the effect of the solvent by exploiting toluene, tetrahydrofuran (THF), acetone, and DCM (entries 8–11). However, the use of the former two was almost ineffective, as only a trace amount of product **3** was detected (entries 8–9). The reaction independently proceeded in the latter two solvents, but both gave unsatisfactory results associated with the reaction yields in comparison to DCE (entries 10–11 vs entry 1). This reaction could run at higher temperatures, but gave a slightly complex system and a decrease in yields was detected (entries 12–13).

**Table 1:** Optimization conditions for forming 3^a^

Entry	[Au] (1 mol%)	Solvent	Yield (%)^b^
1	JohnPhosAu(MeCN)SbF_6_	DCE	65
2	Ph_3_PAuCl	DCE	56
3	XPhosAuCl	DCE	48
4	SPhosAuCl	DCE	trace
5	IPrAuNTf_2_	DCE	29
6	AuCl_3_	DCE	25
7	AuCl	DCE	15
8	JohnPhosAu(MeCN)SbF_6_	THF	N.R.
9	JohnPhosAu(MeCN)SbF_6_	Acetone	N.R.
10	JohnPhosAu(MeCN)SbF_6_	DCM	50
11	JohnPhosAu(MeCN)SbF_6_	Toluene	25
12^c^	JohnPhosAu(MeCN)SbF_6_	DCE	54
13^d^	JohnPhosAu(MeCN)SbF_6_	DCE	49

^a^Reaction conditions: **1a** (0.2 mmol), **2a** (0.4 mmol), and at room temperature in the solvent (2.0 ml) for 6 h.

^b^Isolated yield based on **1a**.

^c^The reaction was conducted at 40 °C for 6 h.

^d^The reaction was conducted at 55 °C for 6 h.

N.R. = not reaction. JohnPhos = 2-(di-*tert*-butylphosphino)biphenyl, SPhos = 2-dicyclohexylphosphino-2’,6’-dimethoxybiphenyl, XPhos = 2-(dicyclohexylphosphino)-2’,4’,6’-tri-*i*-propyl-1,1’-biphenyl, IPr =  isopropyl, NTf_2_ = bis(trifluoromethylsulfonyl)imide.

### Evaluation of the substrate scope

**Fig. 2:**
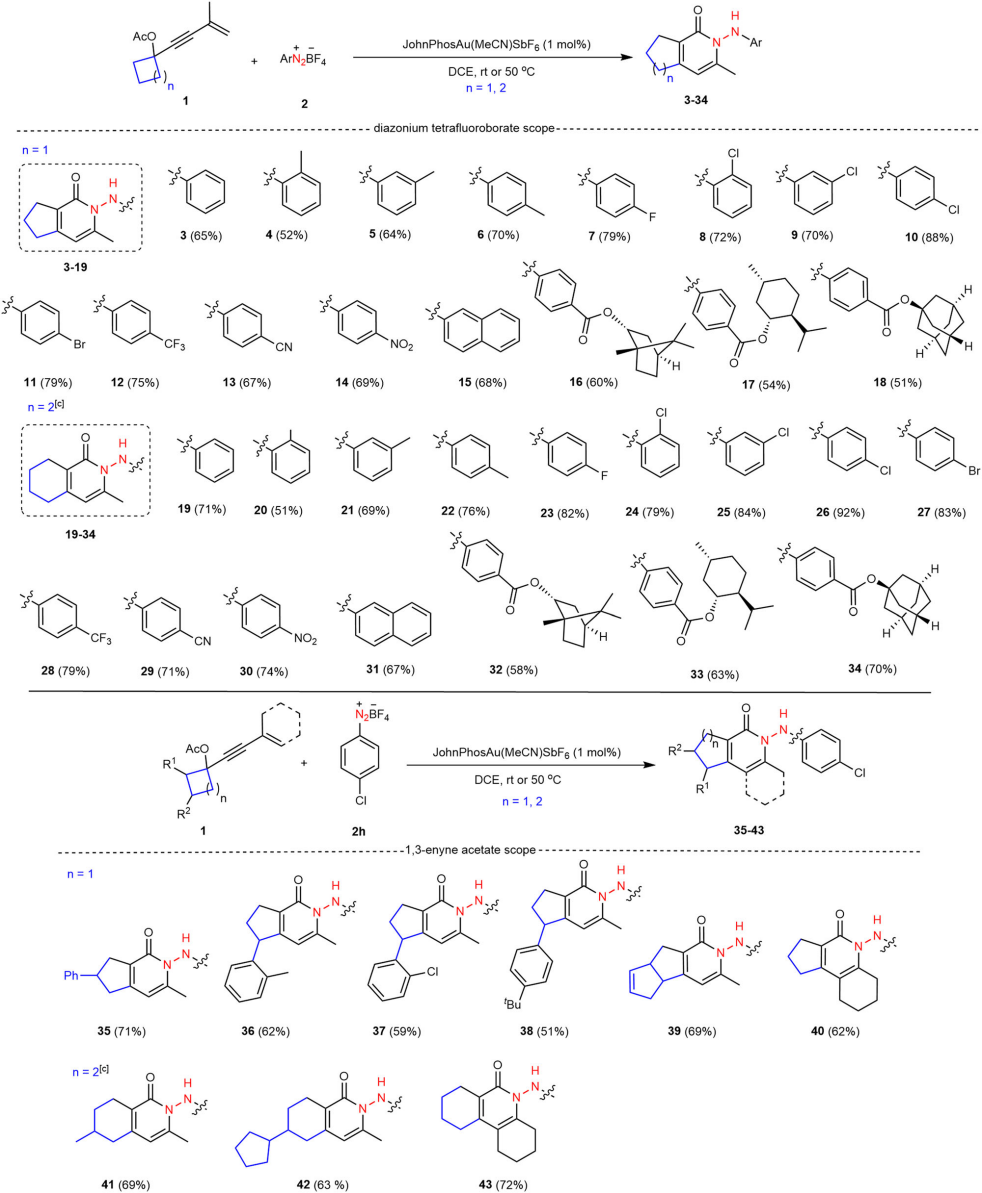
Schmidt-type reaction for the synthesis of bicyclic 2-pyridinones. Reaction conditions: **1** (0.2 mmol), **2** (0.4 mmol), JohnPhosAu(MeCN)SbF_6_ (1 mol%), DCE (2.0 ml), room temperature (rt), 6 h; the yield refers to isolated yield based on **1**; use of cyclopentyl acetate **1** is at 50 °C.

With these acceptable reaction conditions (Table 1, entry 1), we set out to systematically investigate the scope of this transformation by examining the behaviors of 1,3-enyne acetates and aryldiazonium salts. The results are summarized in Fig. 2. First, aryldiazonium salts associated with various substituents successfully participated in this gold-catalyzed ring-expansion relay with **1a**, orienting regiospecific access to the desired cyclopenta[*c*]pyridin-1-ones **4–18** in good yields (Fig. 2). Both electron-donating (EDG) (e.g., methyl, **2b**−**2d**) and electron-withdrawing (EWG) (e.g., fluoro, **2e**; chloro, **2f**−**2h**; bromo, **2i**; trifluoromethyl, **2j**; cyano, **2k**, and nitro, **2l**) groups at different positions (*ortho*, *meta*, or *para*) demonstrated good compatibility of this transformation. Of these functional groups, both *o*-tolyl (**2b**) and *o*-chlorophenyl (**2f**) analogs with strong steric congestion were well-tolerated with this catalytic system, illustrating that the increased steric hindrance had little influence on the reactivity. In addition, three strong electron-withdrawing groups such as CF_3_, CN, and NO_2_ at the *para*-position remained highly reactive in this catalysis, delivering the targets **12–14** in 67–75% yields. Furthermore, the protocol was also compatible with naphthalen-2-yl counterpart **2m**. Notably, three derivatives of naturally occurring chiral alcohol-based esters (L(-)-borneol (**2n**), L-menthol (**2o**), and 1-adamantanol (**2p**)) were also applicable, producing corresponding products **16–18** in good yields through two carbon-carbon bond cleavages. In addition to the cyclobutanone-derived substrate, less strained cyclopentanone-derived counterpart **1b** worked readily, enabling a similar catalytic ring-expansion/nitrogen insertion process to deliver functionalized tetrahydroisoquinolin-1(2H)-ones **19–34** in synthetically useful yields (Fig. 2). Next, the potential variations in the structure of 1,3-enyne acetates were further examined, with *p*-chlorophenyl substrate **2h** as the other partner. 1,3-Enyne acetate **1c**, which bears a phenyl group at the C3 position of the cyclobutene ring, afforded desired product **35** in good yield. Importantly, this ring-expansion reaction proceeded with complete regioselectivity when aryl substituents were installed into the C2 position of the cyclobutene ring, in which a less steric methylene group is more prone to migrate than methine functionality with a substituent under gold-catalyzed conditions. As exemplified by 1,3-enyne substrates **1d–1f**, the expected products **36–38** as single regioisomers were isolated, albeit with moderate yields. A similar phenomenon was discovered in the transformations of both bicyclo[3.2.0]hept-2-en-6-one-derived (**1g**) and C2-alkylated cyclopentanone-derived (**1i** and **1j**) substrates, and regioisomeric products **39**, **41** and **42** were generated with good yields. Moreover, internal alkynes **1h** and **1k** that bear a cyclohexenyl substituent were still tolerated, providing tricyclic products **40** and **43** in acceptable yields (Fig. 2).

**Fig. 3:**
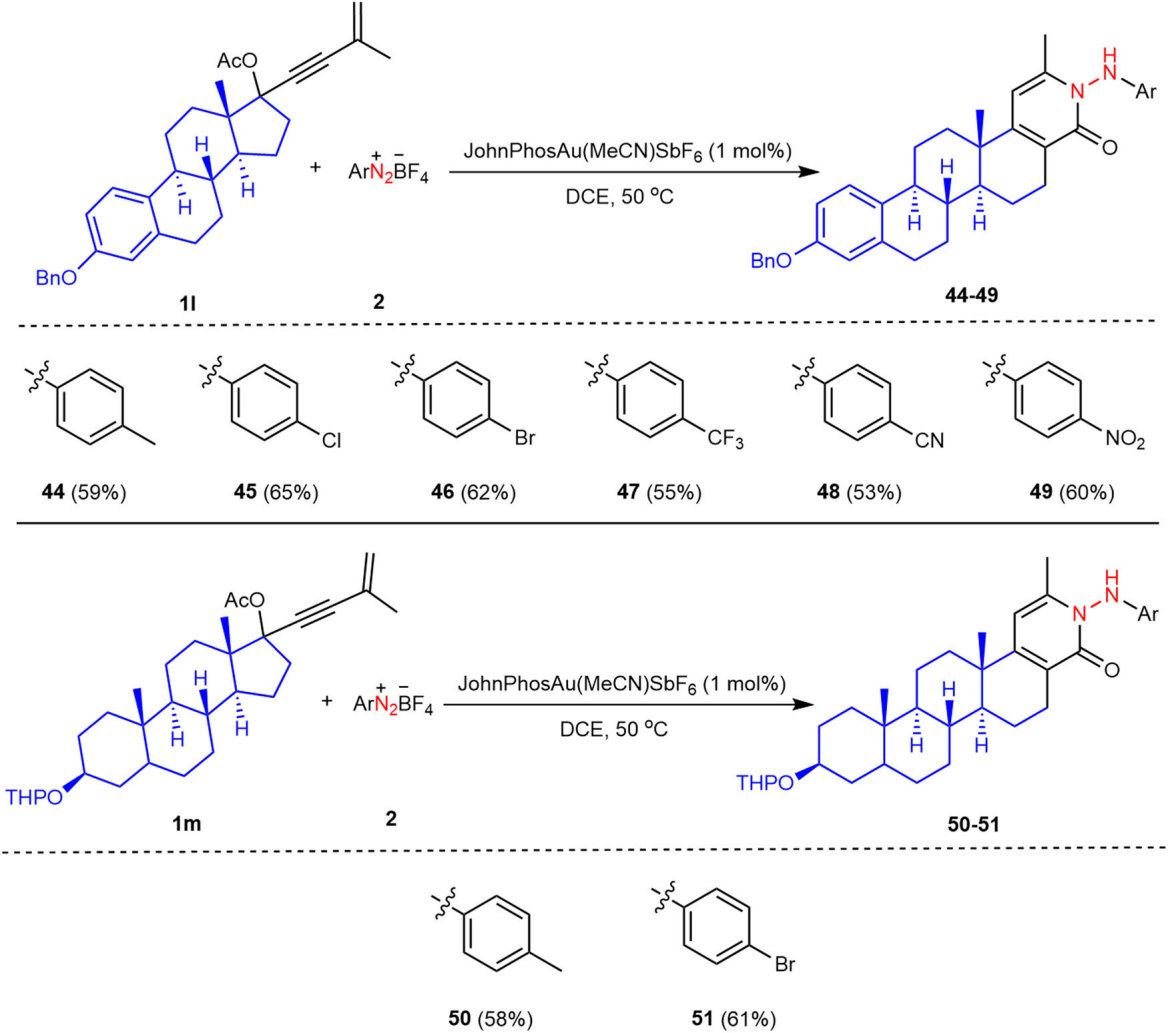
Schmidt-type reaction for the synthesis of azasteroids. Reaction conditions: **1** **l** or **1** **m** (0.2 mmol), **2** (0.4 mmol), JohnPhosAu(MeCN)SbF_6_ (1 mol%), DCE (2.0 ml), 50 °C, 6 h; the yield refers to isolated yield based on **1**.

After the success of the catalytic ring-expansion relay reactions of formal cycloketone-derived 1,3-enyne acetates and aryldiazonium salts, we proceeded to examine other types of naturally occurring cycloketone derivatives, and therefore offer a general ring-expansion strategy to build up other *N*-heterocycles incorporating two medicinally relevant privileged core structures. Azasteroids, normally derived from chemical modifications of steroids, are recognized as a privileged pharmacophore prevalent in pharmaceutical agents^65,66^, but relatively few approaches to their syntheses have been documented in recent years^67–69^. We questioned whether steroid-derived 1,3-enyne acetates could be included in our catalytic cycle as pro-nucleophilic partners to react with aryldiazonium salts **2**. If successful, this reaction would demonstrate an efficient and regiospecific approach to form pentacyclic azasteroid motifs. Following this idea, we tested the reaction of estrone-derived 1,3-enyne acetate **1l** with aryldiazonium salt **2d** under the optimized conditions (Table 1, entry 1). To our delight, the desired pentacyclic azasteroid **44** was obtained in 59% yield (Fig. 3). Furthermore, an array of electron-withdrawing groups, such as halo, trifluoromethyl, cyano, and nitro substituents, at the *para*-position of the benzene ring of aryldiazonium salts proved to be compatible with this protocol (Fig. 3). In addition, epiandrosterone-derived substrate **1m** (tetrahydropyran protection) could be utilized to react with aryldiazonium salts, furnishing diversely substituted azasteroids **50–51** in good yields (Fig. 3).

**Fig. 4:**
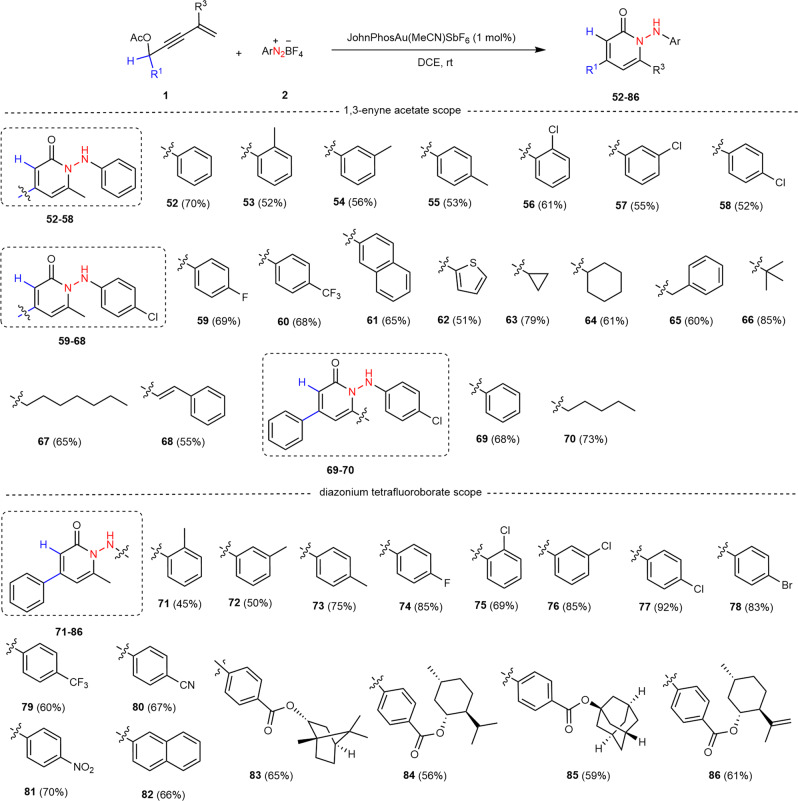
Schmidt-type reaction for the synthesis of poly-substituted 2-pyridinones. Reaction conditions: **1** (0.2 mmol), **2** (0.4 mmol), JohnPhosAu(MeCN)SbF_6_ (1 mol%), DCE (2.0 ml), room temperature (rt), 6 h; the yield refers to isolated yield based on **1**.

To further expand the utility of this methodology, we devoted our effort to testing aldehyde-derived 1,3-enynes as pro-nucleophiles for pyridin-2(1*H*)-one synthesis, because such compounds are a class of prevalent heteroaromatic structures that are frequently encountered in a broad variety of natural products, bioactive agents and approved drugs^70–72^. For this reason, reactions of aldehyde-derived 1,3-enyne acetates with aryldiazonium salts were carried out under standard conditions, and used to produce a wide range of pyridin-2(1*H*)-ones **52-70** with generally good yields in a regiospecific manner (Fig. 4). Aromatic aldehyde-derived 1,3-enyne acetates **1n-1w** bearing both electron-donating and electron-withdrawing groups, regardless of their positions, could be readily engaged in the reaction. Various functional groups, including methyl (**1o–1q**), halogens (Cl, **1r–1t** and F, **1** **u**), and trifluoromethyl (**1v**), were compatible with this catalytic system. The naphthalene ring (**1w**) was also exclusively installed into the product (**61**). As exemplified by substrate **1x**, this protocol was also adaptable to the 1,3-enyne acetate attached to a heteroarene such as thiophene in an acceptable yield (**62**, 51%). Moreover, switching the aryl group at the α-position of 1,3-enyne substrates to other substituents, such as cycloalkyl (cyclopropyl **1** **y** and cyclohexyl **1z**), benzyl (**1aa**), branched (**1ab**) and linear (**1ac**) alkyl, and cinnamyl (**1ad**), had a negligible effect on the reaction performance, orienting the complete regioselectivity to access the target pyridin-2(1*H*)-one products **63-68** with acceptable yields. When the methyl group (R^3^) on the terminal alkene moiety of 1,3-enyne acetates was replaced by phenyl and *n*-pentyl groups, products **69** and **70** were isolated in 68% and 73% yields, respectively. Then, we examined the generality of this catalytic annulation/nitrogen insertion cascade regarding aryldiazonium salts **2** by combining with benzaldehyde-derived substrate **1n**. The detailed investigations on substituents including alkyl, halogens, trifluoromethyl, cyano, and nitro at different positions in the arene ring as well as a sterically more demanding bicyclic aromatic example, such as naphthyl group, revealed that all these attempts were applicable for the reaction (products **71–82**, Fig. 4). The presence of halogen, cyano, and nitro moieties in these synthons adds greatly to the synthetic value of the cyclization products, as they can act as an additional handle for late-stage transformations. Aryldiazonium salts that bear a chiral alcohol-based ester such as L(-)-borneol (**2n**), L-menthol (**2o**), 1-adamantanol (**2p**) and (-)-isopulegol (**2q**) on the benzene ring successfully gave corresponding products **83–86** in 56-65% yields (Fig. 4).

### Gram-scale synthesis and synthetic applications

**Fig. 5:**
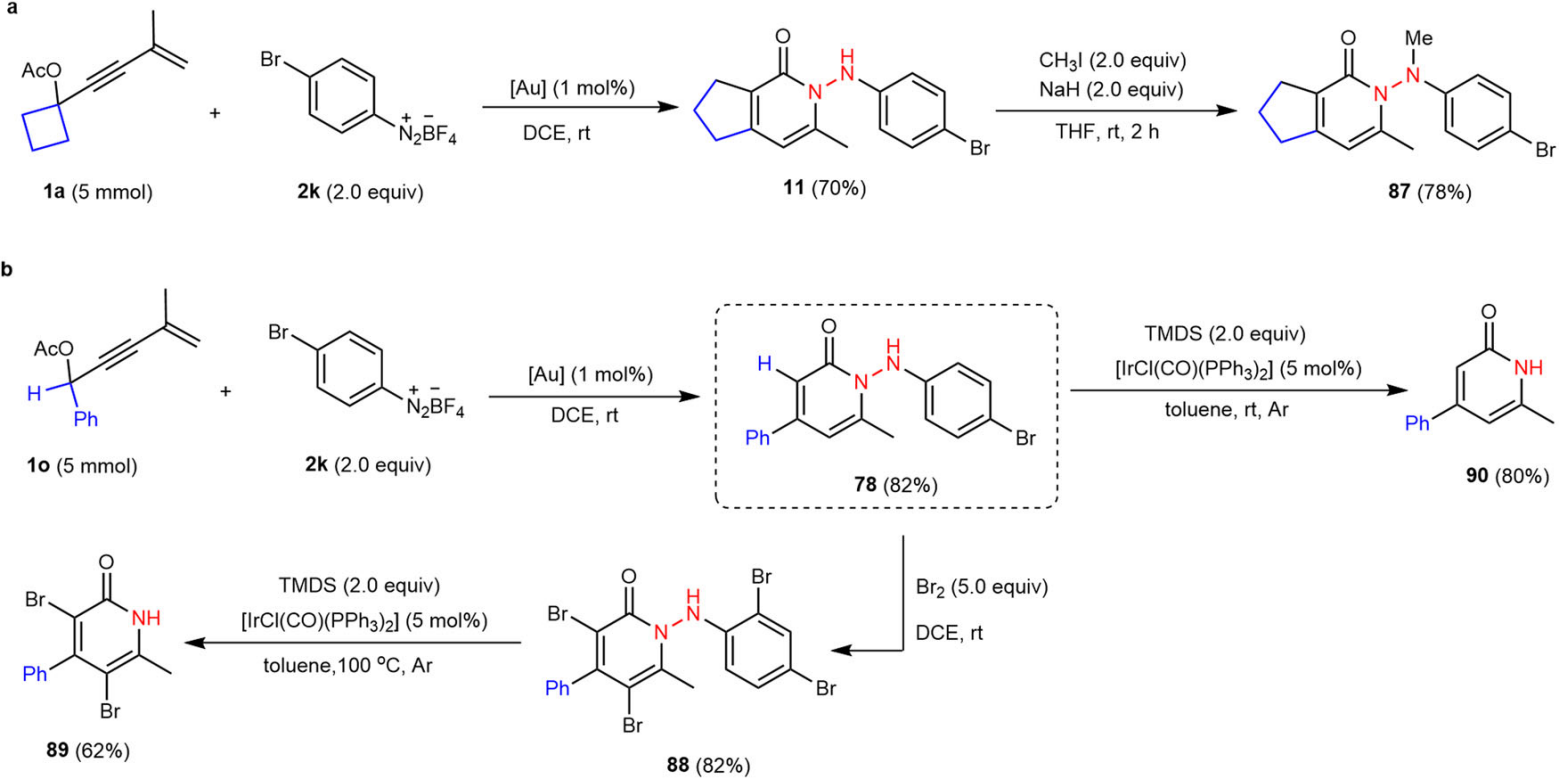
Gram-scale experiments and synthetic applications. **a** Gram-scale synthesis of **11** and *N*-protection of **11**. **b** Gram-scale synthesis of **78** and synthetic transformations. TMDS, tetramethyldisilazane.

To demonstrate the practicality and scalability of the present protocol, gram-scale reactions of **1a** and **1o** (5.0 mmol) and **2k** were independently carried out with 1 mol% JohnPhosAu(MeCN)SbF_6_ under the optimized conditions, affording products **11** and **78** in slightly diminished yields (Fig. 5a, b). Subsequently, compound **11** reacted with methyl iodide in the presence of NaH, furnishing methyl-protected **87** in 78% yield (Fig. 5a). Treatment of **78** with Br_2_ gave polybrominated product **88** in 82% yield, followed by the Ir-catalyzed reaction of tetramethyldisilazane (TMDS) to access 3,5-dibromopyridin-2(1H)-one **89** in 62% yield through N-N bond cleavage (Fig. 5b). A similar transformation occurred when using **78** and TMDS in the presence of iridium catalyst (Fig. 5b).

### Mechanistic studies

**Fig. 6:**
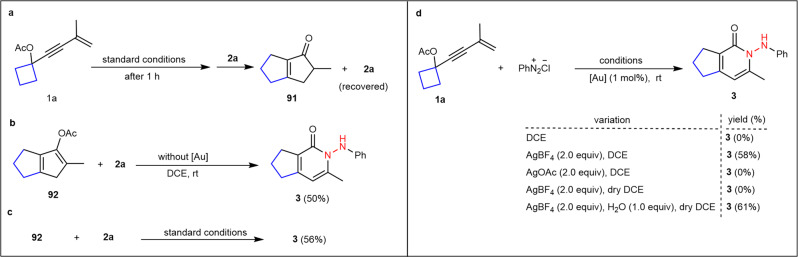
Preliminary mechanism investigations. **a** Two-step reaction of **1a** with **2a**. **b** Metal-free reaction of **92** with **2a**. **c** The role of the gold catalyst in the Schmidt-type reaction. **d** Investigations on the roles of tetrafluoroborate anion and H_2_O for Schmidt-type reaction.

To gain mechanistic insights into this nitrogen insertion process, some control experiments were conducted, as shown in Fig. 6. In Zhang’s procedure, gold-catalyzed Nazarov cyclization of 1,3-enyne acetates led to two different products of cyclopentenones and unstable cyclopentadienes^54^. To investigate the possibility of intermediates for this reaction, a gold-catalyzed reaction of **1a** was run under standard conditions for 1 h, and then **2a** was placed into this reaction system. As a result, instead of the target **3**, 2-methyl-3,4,5,6-tetrahydropentalen-1(2*H*)-one **91** was generated, along with the recovered **2a** (Fig. 6a). Without a gold catalyst, the reaction between preformed 2-methyl-3,4,5,6-tetrahydropentalen-1-yl acetate **92** and **2a** gave a 50% yield of **3a** (Fig. 6b). Next, treatment of **92** with **2a** under standard conditions provided a slightly increased yield (56%, Fig. 6c). These outcomes demonstrate that cyclopentadienes, rather than cyclopentenones, are potential reaction intermediate, and the gold catalyst is unnecessary but could promote the nitrogen insertion reaction to some extent. Let us examine in detail the roles of tetrafluoroborate anion and H_2_O (Fig. 6d). Without tetrafluoroborate, the reaction of **1a** with benzenediazonium chloride did not proceed under the standard conditions whereas the use of 2.0 equivalents of silver tetrafluoroborate as tetrafluoroborate sources provided **3a** in 58% yield. Exchanging silver tetrafluoroborate for silver acetate did not give **3a**. The reaction in the absence of H_2_O also did not work. Evidently, when 1.0 equivalent of H_2_O was added to the above reaction system, product **3a** was obtained in 61% yield. These results indicate that both tetrafluoroborate and H_2_O are crucial for this transformation (for their roles, see the mechanism section).

### Possible reaction pathways

**Fig. 7:**
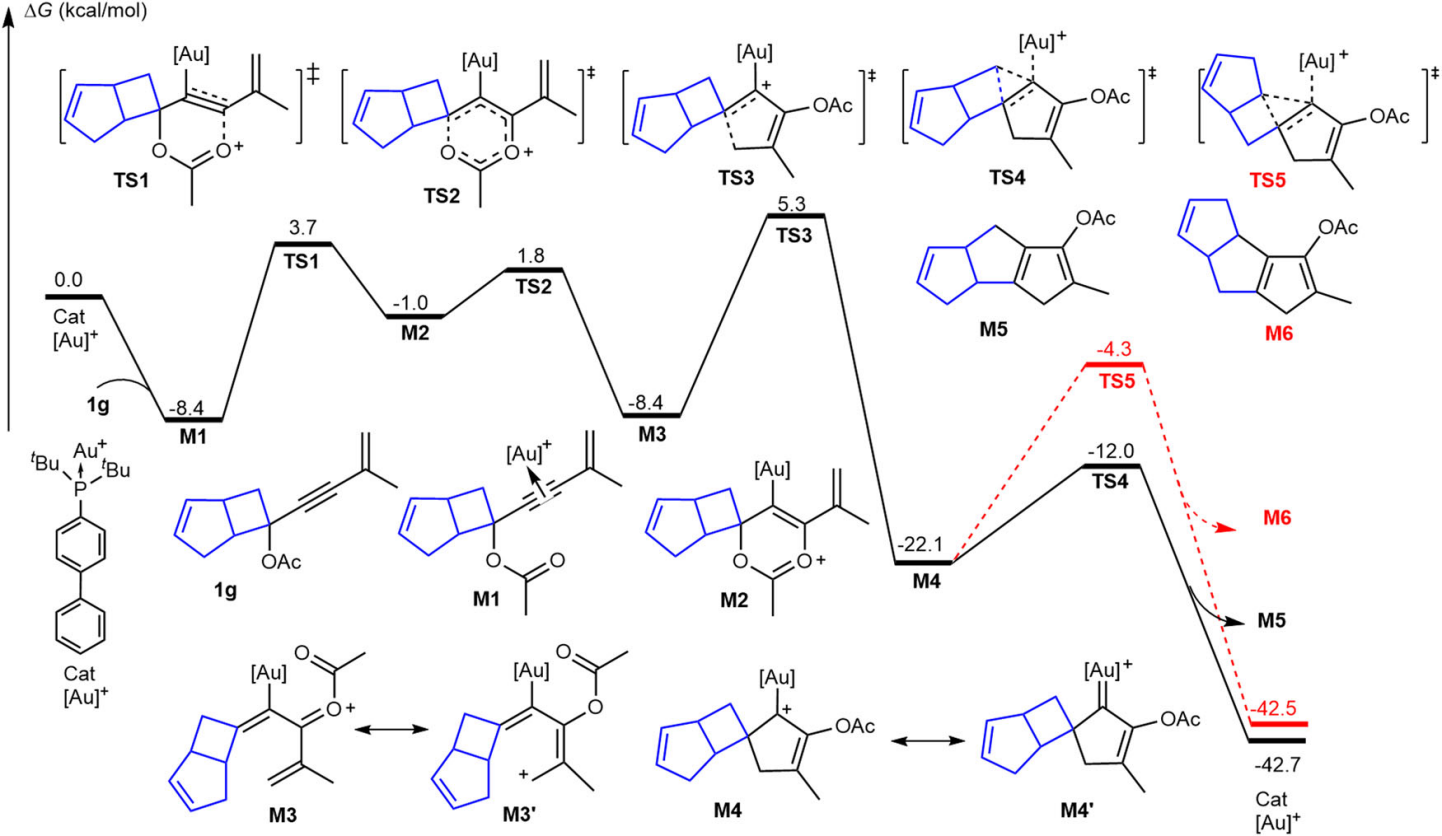
Calculated free energy profiles for the cationic Au(I) catalyzed cyclization of cyclopentadienyl acetate. The relative free energies are computed at the M06-L/def2-TZVPP/SMD_dichloroethane_//M06-L/def2-SVP/SMD_dichloroethane_ level.

To investigate the possible mechanism of the Au(I)-catalyzed Schmidt-type reaction between cyclopentadienyl acetate and aryldiazonium salt, a theoretical study employing density functional theory (DFT) calculations was performed at M06-L/def2-TZVPP/SMD_dichloroethane_//M06-L/def2-SVP/SMD_dichloroethane_ level of theory^73^ (Fig. 7). As a typical π-acetic metal, Au in active species catalyst could be coordinated by cyclopentadienyl acetate **1g** to form complex **M1** with exergonic energy of 8.4 kcal/mol. Activated by the π-acetic Au(I) cation, the coordinating C-C triple bond could be nucleophilically attacked by the intramolecular ester group to achieve stepwise acetoxy transfer via the six-membered ring transition states **TS1** and **TS2**. The corresponding free energy barriers are 12.1 kcal/mol and 2.8 kcal/mol, respectively, indicating a rapid process. The consequence of stepwise acetoxy transfer is the formation of oxonium intermediate **M3**, which could be regarded as an equilibrium with intermediate **M1** in thermodynamic^74–76^. Resonance structure **M3’** clearly reveals the electrophilicity of its terminal methylene position, which results in an intramolecular electrophilic 1,5-annulation via transition state **TS3** to irreversibly form an Au-carbene complex **M4**. The calculated free energy barrier for the step was detected to be 13.7 kcal/mol. The resonance structure of **M4’** reveals a cationic carbon, which would cause 1,2-carbon cation rearrangement. The calculated free energy barrier for the intramolecular 1,2-shift of the unsubstituted methyl group via transition state **TS4** is 10.1 kcal/mol. However, the corresponding energy barrier of the cyclopentyl shift via transition state **TS5** is 17.8 kcal/mol. Therefore, fused pentalenyl acetate **M5** was found to be the major product, which is fully consistent with the experimental observations.

**Fig. 8:**
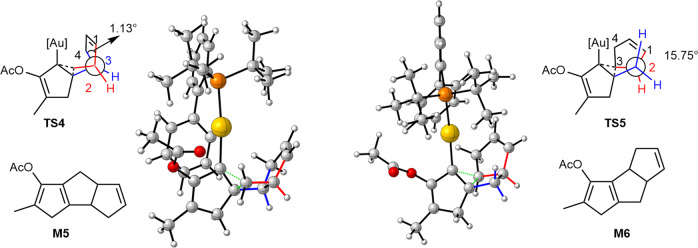
The Newman projected structure of the transition states TS4 and TS5. The torsion angles are computed at the M06-L/def2-TZVPP/SMD_dichloroethane_//M06-L/def2-SVP/SMD_dichloroethane_ level.

To further analyze the regioselectivity, the Newman projections of transition states **TS4** and **TS5** are given in Fig. 8. In the geometry of transition state **TS4**, the strain of the fused five-membered ring (right part) promoted the rotation of the projecting C-C single bond, which led to the 1,2-shift of the methyl group with strain release. Meanwhile, the corresponding projecting C-C bond in **TS5** is free without strain. Therefore, the lack of a driving force makes the corresponding 1,2-shift difficult via **TS5**.

**Fig. 9:**
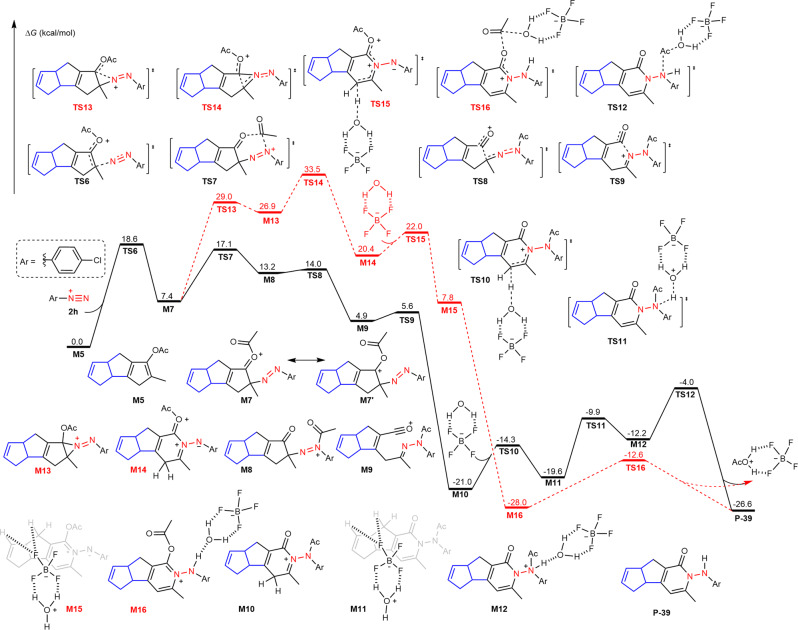
Calculated free energy profiles for the cyclization of aryldiazonium salt and intermediate 3*H*-cyclopenta[*a*]pentalen-1-yl acetate M5. The relative free energies are computed at the M06-L/def2-TZVPP/SMD_dichloroethane_//M06-L/def2-SVP/SMD_dichloroethane_ level.

The reaction pathway of further transformation of fused pentalenyl acetate **M5** was also considered by DFT calculations. As shown in Fig. 9, the enol ester moiety of **M5** could nucleophilically attack aryldiazoniom salt **2** **h** via transition state **TS6** affording oxonium intermediate **M7** with a free energy barrier of only 18.6 kcal/mol. After that, two possible pathways for the expansion of the ring could occur: acyl transfer promoted arrangement (solid black lines) or nitrogen-insertive expansion (dashed red lines). In intermediate **M7**, the azo moiety reveals nucleophilicity, which can attack the acyl group to achieve an intramolecular acyl transfer via transition state **TS7** with a free energy barrier of only 9.7 kcal/mol. The acyldiazenium moiety in intermediate **M8** leads to the cleavage of an active C-C bond via transition state **TS8** with an energy barrier of only 0.8 kcal/mol. The generated carbonyl cation in intermediate **M9** could undergo an intramolecular nucleophilic attack by carbonyl cation via transition state **TS9**, which provides an oxodihydropyridinium intermediate **M10**. Furthermore, a deprotonation process could be associated with a complex of water and BF_4_ anion via transition state **TS10** with a free energy barrier of only 6.7 kcal/mol. Finally, the stepwise hydrolysis of Ac-protected product **M11** could lead to the formation of the final product **P-39**. Moreover, resonance structure **M7’** exhibits an electrophilic carbon atom, which can be nucleophilically attacked by an azo moiety via transition state **TS13** to afford aziridinium intermediate **M13**. Although C-C bond cleavage via transition state **TS14**, hydrolysis of amide and deprotonation could also provide the same product **P-39**, the much higher free energy barrier of this process makes it an unfavorable path.

## Discussion

In this work, the discovery of a Schmidt-type reaction triggered by the soft nucleophilicity of azoarenes is described. This provides a general and practical approach to highly substituted 2-pyridinones and their fused heterocyclic system from readily available 1,3-enyne acetates with aryldiazonium salts in a highly regioselective fashion. The reaction is catalyzed by a well-defined air-stable gold catalyst, involving a 3,3-rearrangement/Nazarov cyclization and nitrogen insertion pathway, under mild conditions and demonstrates wide substrate compatibility. Mechanistic studies reveal a soft nucleophilic property of azoarenes to induce C-C cleavage and nitrogen insertion. Systematic theoretical calculations led us to propose that ring strain controls the regioselectivity of C-C σ-bond migration for nonsymmetric cycloketone-derived 1,3-enyne acetates and that the nucleophilicity of azoarenes induces acyl migration to achieve Schmidt-type rearrangement, thereby giving rise to 2-pyridinone-based cyclic products with high annulation efficiency and regioselectivity from unsaturated linear substrates.

## Methods

General procedure for the gold-catalyzed domino reaction of 1,3-enyne acetates and aryldiazonium tetrafluoroborates. To a 10 mL Schlenk tube under air conditions, cyclobutanone- and aldehyde-derived 1,3-enyne acetates (**1**, 0.2 mmol), aryldiazonium tetrafluoroborates (**2**, 0.4 mmol), JohnPhosAu(MeCN)SbF_6_ (1 mol%), and 1,2-dichloroethane (2.0 ml) were successively added. The mixture was stirred at room temperature (the reaction of cyclopentanone-derived 1,3-enyne acetates was at 50 °C) for 6 h. After the reaction was completed (indicated by TLC, petroleum ether: ethyl acetate = 2:1), the reaction mixture was concentrated by vacuum distillation and was purified by flash column chromatography to afford the desired pure products **3–86**.

## Supplementary information

SUPPLEMENTARY INFORMATION

Description of Additional Supplementary Files

Supplementary Data 1

## Data Availability

The data generated in this study are provided in the Supplementary Information file. For the experimental procedures, data of NMR and HRMS analysis and computational details, see Supplementary Methods and Figures in the Supplementary Information file. The authors declare that all these data supporting the findings of this study are available within the article and Supplementary Information files and are also available from the corresponding author upon request.

## Code availability

The X-ray crystallographic data generated in this study have been deposited at the Cambridge Crystallographic Data Centre (CCDC) under deposition number CCDC 2142493 (**5**), 2142494 (**39**), and 2142495 (**55**). The X-ray crystallographic data are available free of charge from The Cambridge Crystallographic Data Centre via http://www.ccdc.cam.ac.uk/data_request/cif.
