# Supplementary material for: Azoarene activation for Schmidt-type reaction and mechanistic insights
Source: Nat Commun. 2022 Dec 1;13:7393. doi: 10.1038/s41467-022-35141-4 (PMC9712421; doi:10.1038/s41467-022-35141-4)
Supplement: Supplementary file 1 — SUPPLEMENTARY INFORMATION [file 41467_2022_35141_MOESM1_ESM.pdf]

Supplementary Information

Azoarene Activation for Schmidt-Type Reaction and Mechanistic Insights

Fan-Tao Meng,[1,∇] Ya-Nan Wang,[2,∇] Xiao-Yan Qin,[1] Shi-Jun Li,[2] Jing Li,[1] Wen-Juan Hao*,[1] Shu-Jiang Tu,[1] Yu Lan*,[2] & Bo Jiang*,[1]

[1]School of Chemistry & Materials Science, Jiangsu Key Laboratory of Green Synthetic Chemistry for Functional Materials, Jiangsu Normal University, Xuzhou, 221116, P. R. China, *E-mail: wjhao@jsnu.edu.cn (WJH); jiangchem@jsnu.edu.cn (BJ)

[2]College of Chemistry and Institute of Green Catalysis, Zhengzhou University, Zhengzhou, Henan, 450001, China, *E-mail: lanyu@cqu.edu.cn

[∇]F.T.M. and Y.N.W. contributed equally

Supplementary Note…………………………..……………………………S3
Supplementary Methods……………………..……………………………S4
Supplementary References…………………………………………… ……S259

1. Supplementary Note

1.1 General Information

All reactions were carried with standard procedures under air atmosphere except for the special mention. All chemicals were obtained from commercial sources (Energy-chemical, Bidepharm & Innochem), and were used without further purification. Gold catalysts were obtained from commercial sources (Laajoo). Column chromatography was generally performed on silica gel (300-400 mesh) and reactions were monitored by thin-layer chromatography (TLC) using 254 nm UV light. ^{1}H NMR (400 MHz), and ^{13}C NMR (100 MHz) were measured on Bruker DPX 400 spectrometer. Chemical shifts are expressed in parts per million (ppm) with respect to tetramethylsilane. Coupling constants were reported as Hertz (Hz), signal shapes and splitting patterns were indicated as follows: s = singlet; d = doublet; t = triplet; q = quartet; m = multiplet. High-resolution mass spectra (HRMS) were recorded on microTOF-QII HRMS/MS instrument (BRUKER) equipped with the ESI or APCI source and a Q-TOF detector. The X-ray analysis of crystal structure was performed with a Siemens SMART CCD and a Siemens P4 diffractometer.

2. Supplementary Methods

2.1 Synthesis of substrates 1 and 2

2.1.1 Preparation of 1,3-Enyne Acetates 1

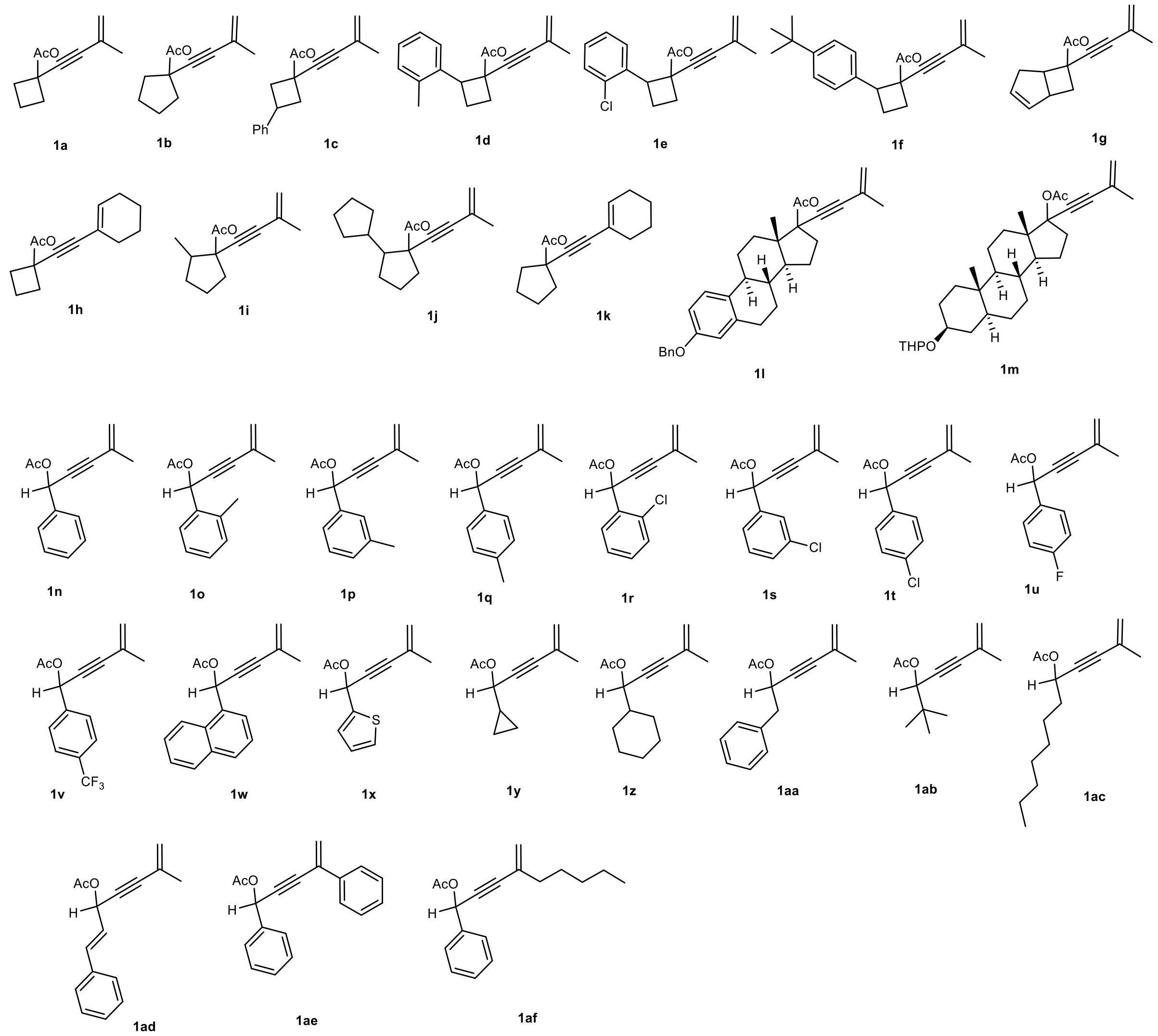

Supplementary Figure 1 Substrate Scope of 1,3-Enyne Acetates **1**

2.1.1.1 General procedure A (Substrates 1a-1c and 1g-1ad) [1-3]

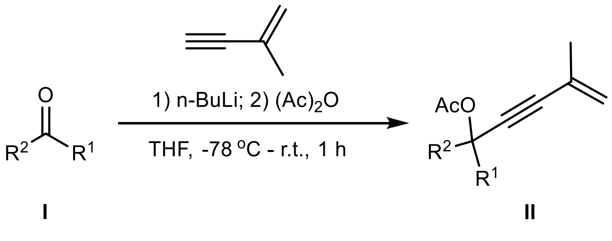

Supplementary Figure 2 Synthesis of Compounds **1a**-**1c** and **1g**-**1ad**

Step 1: To a THF (0.5 M) solution of 2-methylbut-1-en-3-yne (1.0 equiv), *n*-BuLi (2.5 M in hexane, 1.1 equiv) was added at -78 °C, and the mixture was stirred at this temperature for 0.5 hour. Then, **I**

(1.0 equiv) was added to this solution at -78 °C, and the reaction mixture was warmed to room temperature, and stirred for 1 hour. The solution was added with acetic anhydride (1.2 equiv) and stirred for 1 hour. To this reaction mixture, a saturated NH_4Cl solution was added and the solution was extracted with diethyl ether (2 x 50 mL) and washed with brine (30 mL). The combined organic extracts were dried over anhydrous $MgSO_4$ and concentrated under reduced pressure. The crude material was then purified by column chromatography on silica gel with a mixture of petroleum ether and ethyl acetate to give **II**.

2.1.1.2 General procedure B (Substrates 1d-1f)[4-7]

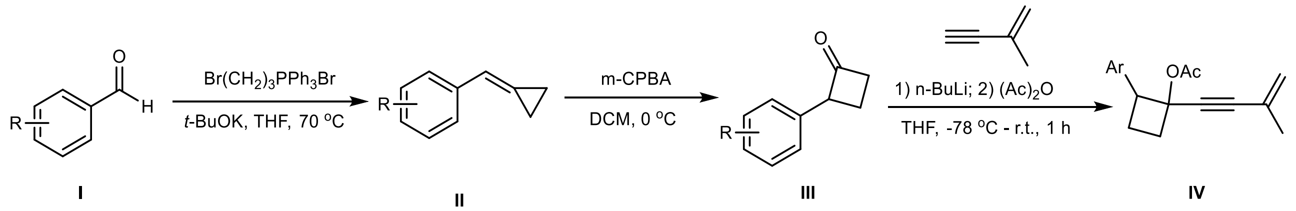

Supplementary Figure 3 Reaction Synthesis of Compounds **1d-1f**

Step 1: A solution of (3-bromopropyl)triphenylphosphonium bromide (13.92 g, 30 mmol, 3.0 equiv) in dry THF (60 mL) was added to a Schlenk tube (200 mL) under the protection with argon. Then, the solution of *t*-BuOK (6.73 g, 30 mmol, 3.0 equiv) in dry THF (100 mL) was slowly dropped to the stirred mixture at 0 °C. Then the resulting orange solution was heated to 70 °C in oil bath and stirred for 2 hours before arylaldehyde **I** (10 mmol, 1.0 equiv) were added, and stirring was continued at 70 °C for overnight. After cooling, the suspension was filtered and the solvent of the filtrate was removed under vacuum, the products were purified by column chromatography on silica gel to afford (arylmethylene)cyclopropanes **II**.[4-6]

Step 2: To solution of (arylmethylene)cyclopropanes **II** (1.0 equiv) in DCM (0.15 M), a solution of *m*-CPBA (1.05 equiv.) in DCM (0.38 M) was added by dropwise at 0 °C and stirred for 1 hour. After completing reaction (by TLC), the reaction mixture was quenched by a saturated solution of aqueous $Na_2S_2O_3$ (30 mL) at room temperature, and the aqueous layer was extracted with DCM (3 × 20 mL). The combined organic layers were washed with a saturated solution of aqueous $NaHCO_3$ (3 × 30 mL), brine (30 mL), dried over anhydrous $MgSO_4$, filtered, and concentrated under reduced pressure. The crude product was purified by column chromatography on silica gel to give the corresponding 2-arylcyclobutanones **II**.

Step 3: To a THF (0.5 M) solution of 2-methylbut-1-en-3-yne (1.0 equiv), *n*-BuLi (2.5 M in hexane,

1.1 equiv) was added at -78 °C, and the mixture was stirred at this temperature for 0.5 hour. Then, **III** (1.0 equiv) was added to this solution at -78 °C, and the reaction mixture was warmed to room temperature, and stirred for 1 hour. The solution was added with acetic anhydride (1.2 equiv) and stirred for 1 hours. To this reaction mixture was added, a saturated NH_4Cl solution, and the solution was extracted with diethyl ether (2 x 50 mL) and washed with brine (30 mL). The combined organic extracts were dried over $MgSO_4$ and concentrated under reduced pressure. The crude material was then purified by column chromatography on silica gel with a mixture of petroleum ether and ethyl acetate to give **IV**.

Compounds **1c**-**1k** are new compounds, while the rest have been previously reported, and their characterization data are in agreement with the literature.

2.1.2 Preparation of aryldiazonium salts 2

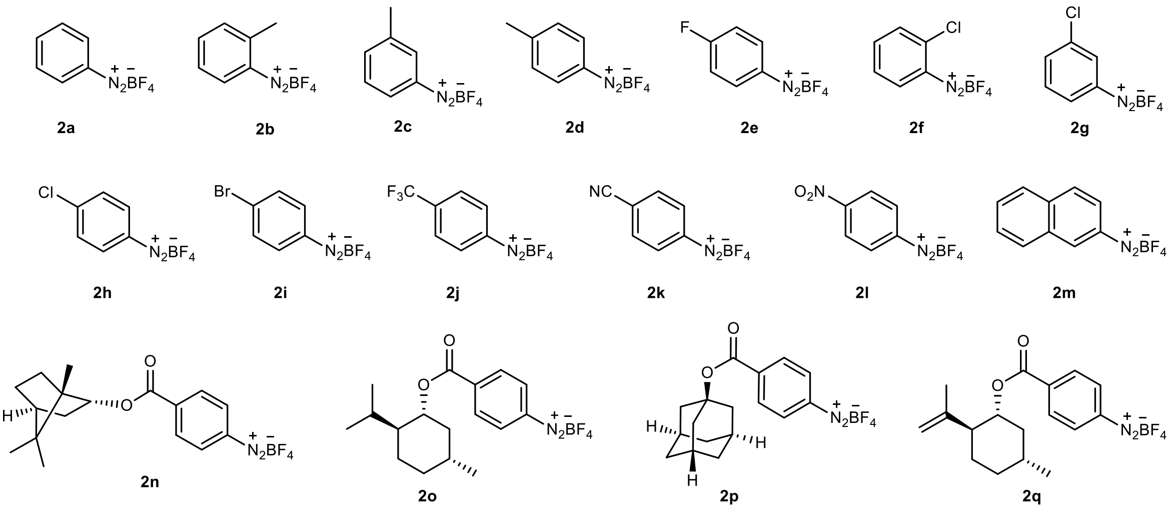

Supplementary Figure 4 Substrate Scope of Aryldiazonium Salts **2**

Aryl amines (1.0 equiv, 10 mmol) were dissolved in a mixture of H_2O (3.0 mL) and an aqueous solution of HBF_4 (48%, 3ml) in a 100 mL round-bottom flask with a stir bar, the mixture was stirred for 2 minutes at 0 °C. Then, $NaNO_2$ (1.1 equiv, 11 mmol, 0.759 g) was dissolved in 3 mL of water and added to the system, stirred at 0 °C for 20 minutes. Finally, Et_2O (20 mL) was added to precipitate aryldiazonium salts. The solid was filtered off and washed with Et_2O (3 × 10 mL). Aryldiazonium salts **2a**-**2q** were dried in vacuo and directly used without further purification.[8]

2.1.3 Characterization data of new compounds 1

1-(3-Methylbut-3-en-1-yn-1-yl)-3-phenylcyclobutyl acetate (1c)

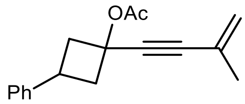

yellowish oil (general procedure A, 90% yield); ^{1}H NMR (400 MHz, $CDCl_3$): δ 7.42-7.37 (m, 2H), 7.30 (d, J = 6.8 Hz, 3H), 5.45 (s, 1H), 5.35 (s, 1H), 3.64-3.53 (m, 1H), 3.20-3.11 (m, 2H), 2.62-2.57 (m, 2H), 2.14 (s, 3H), 2.01 (s, 3H); ^{13}C NMR (100 MHz, $CDCl_3$): δ 169.1, 143.7, 128.6, 126.6, 122.7, 88.3, 85.6, 68.4, 44.6, 32.6, 23.5, 21.4; IR (KBr, v, cm^{-1}) 2932, 1744, 1610, 1499, 1232, 1018, 734; HRMS (ESI-TOF) calcd for $C_{17}H_{18}O_2Na$ [M+Na]$^+$ 277.1204, found 277.1211.

1-(3Mmethylbut-3-en-1-yn-1-yl)-2-(o-tolyl)cyclobutyl acetate (1d, major)

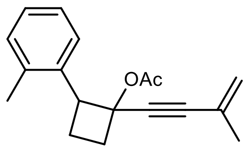

yellowish oil (general procedure B, 64% yield); ^{1}H NMR (400 MHz, $CDCl_3$): δ 7.26 (d, J = 3.2 Hz, 1H), 7.23-7.21 (m, 3H), 5.12 (d, J = 19.6 Hz, 2H), 4.12-4.07 (m, 1H), 2.77-2.73 (m, 1H), 2.62-2.57 (m, 1H), 2.46 (s, 3H), 2.43-2.40 (m, 1H), 2.18-2.15 (m, 1H), 2.13 (s, 3H), 1.68 (s, 3H); ^{13}C NMR (100 MHz, $CDCl_3$): δ 169.1, 169.1, 137.9, 136.9, 135.8, 130.1, 128.0, 126.9, 125.3, 122.6, 88.8, 85.3, 78.0, 48.5, 34.5, 23.2, 21.6, 20.43, 18.8; IR (KBr, v, cm^{-1}) 3234, 3047, 2923, 1652, 1558, 1488, 771, 692; HRMS (ESI-TOF) calcd for $C_{18}H_{20}O_2Na$ [M+Na]$^+$ 291.1361, found 291.1372.

2-(4-(tert-Butyl)phenyl)-1-(3-methylbut-3-en-1-yn-1-yl)cyclobutyl acetate (1f, major)

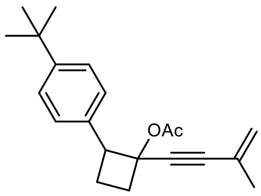

yellowish oil (general procedure B, 65% yield); ^{1}H NMR (400 MHz, $CDCl_3$): δ 7.37 (d, J = 8.4 Hz, 3H), 7.24 (s, 1H), 5.07 (d, J = 24.4 Hz, 2H), 3.85-3.80 (m, 1H), 2.75-2.59 (m, 2H), 2.37 (d, J = 11.2 Hz, 1H), 2.22-2.19 (m, 1H), 2.09 (s, 3H), 1.63 (s, 3H), 1.32 (s, 9H); ^{13}C NMR (100 MHz, $CDCl_3$): δ 169.2, 149.7, 136.0, 127.8, 126.7, 125.6, 124.8, 122.6, 88.9, 85.7, 64.3, 51.2, 34.7, 31.4, 23.0, 21.5, 19.2; IR (KBr, v, cm^{-1}) 3234, 3047, 2923, 1652, 1558, 1488, 771, 692; HRMS (ESI-TOF) calcd for $C_{21}H_{26}O_2Na$ [M+Na]$^+$ 333.1830, found 333.1838.

6-(3-Methylbut-3-en-1-yn-1-yl)bicyclo[3.2.0]hept-2-en-6-yl acetate (1g)

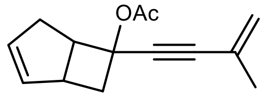

yellowish oil (general procedure A, 81% yield); ^{1}H NMR (400 MHz, $CDCl_3$): δ 5.81 (s, 1H), 5.78 (d, J = 2.4 Hz, 1H), 3.47 (d, J = 1.6 Hz, 1H), 3.24 (d, J = 5.6 Hz, 1H), 3.00-2.94 (m, 1H), 2.67-2.51 (m, 2H), 2.36 (s, 3H), 2.19-2.14 (m, 1H), 2.08 (s, 3H), 1.60 (s, 2H); ^{13}C NMR (100 MHz, $CDCl_3$): δ 169.1, 133.6, 133.5, 132.4, 132.4, 92.4, 84.3, 70.0, 48.0, 43.1, 39.5, 33.5, 32.7, 21.1; IR (KBr, ν, cm^{-1}) 2963, 1746, 1614, 1368, 1239, 1030, 896; HRMS (ESI-TOF) calcd for $C_{14}H_{16}O_2Na$ $[M+Na]^+$ 239.1048, found 239.1056.

1-(Cyclohex-1-en-1-ylethynyl)cyclobutyl acetate (1h)

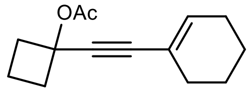

yellowish oil (general procedure A, 85% yield); ^{1}H NMR (400 MHz, $CDCl_3$): δ 6.15-6.09 (m, 1H), 2.58-2.51 (m, 2H), 2.45-2.37 (m, 2H), 2.13-2.06 (m, 4H), 2.03 (s, 3H), 1.98-1.84 (m, 2H), 1.63-1.53 (m, 4H).; ^{13}C NMR (100 MHz, $CDCl_3$): δ 169.3, 135.6, 135.6, 120.2, 86.6, 86.2, 72.5, 37.1, 29.2, 25.7, 22.3, 21.5, 14.7; IR (KBr, ν, cm^{-1}) 2937, 1745, 1436, 1238, 1020, 971, 842; HRMS (ESI-TOF) calcd for $C_{14}H_{18}O_2Na$ $[M+Na]^+$ 241.1204, found 241.1199.

2-Methyl-1-(3-methylbut-3-en-1-yn-1-yl)cyclopentyl acetate (1i)

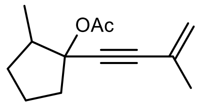

yellowish oil (general procedure A, 84% yield); ^{1}H NMR (400 MHz, $CDCl_3$): δ 5.26 (s, 1H), 5.18 (s, 1H), 2.50-2.36 (m, 1H), 2.27-2.22 (m, 1H), 2.13-2.02 (m, 1H), 2.01 (s, 3H), 1.87 (s, 3H), 1.85-1.82 (m, 1H), 1.75-1.65 (m, 2H), 1.35-1.30 (m, 1H), 1.09 (d, J = 6.8 Hz, 3H); ^{13}C NMR (100 MHz, $CDCl_3$): δ 169.6, 126.5, 121.9, 88.2, 86.3, 84.3, 44.5, 38.8, 30.5, 23.5, 21.8, 21.2, 16.2; IR (KBr, ν, cm^{-1}) 2951, 1747, 1451, 1236, 1032, 895, 735; HRMS (ESI-TOF) calcd for $C_{13}H_{18}O_2Na$ $[M+Na]^+$ 229.1204, found 229.1220.

2-(3-Methylbut-3-en-1-yn-1-yl)-[1,1'-bi(cyclopentan)]-2-yl acetate (1j, major)

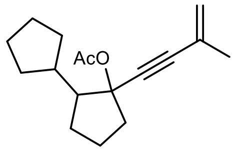

yellowish oil (general procedure A, 86% yield); ^{1}H NMR (400 MHz, $CDCl_3$): δ 5.23 (s, 1H), 5.17 (s, 1H), 5.16 (s, 1H), 2.61-2.50 (m, 2H), 2.15 (d, J = 8.8 Hz, 1H), 2.01 (s, 3H), 1.91-1.87 (m, 3H), 1.84 (s, 3H), 1.64-1.60 (m, 4H), 1.52-1.49 (m, 3H), 1.40-1.37 (m, 2H), 1.13 (d, J = 8.4 Hz, 1H); ^{13}C NMR (100 MHz, $CDCl_3$): δ 169.4, 143.2, 126.7, 121.52, 89.2, 86.4, 81.5, 57.7, 40.9, 40.0, 32.2, 31.7, 31.4, 30.6, 29.5, 28.0, 25.3, 24.6, 23.3, 21.9; IR (KBr, v, cm^{-1}) 2947, 1747, 1614, 1220, 1078, 946, 699; HRMS (ESI-TOF) calcd for $C_{17}H_{24}O_2Na$ $[M+Na]^+$ 283.1674, found 283.1670.

1-(Cyclohex-1-en-1-ylethynyl)cyclopentyl acetate (1k)

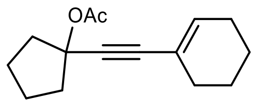

yellowish oil (general procedure A, 80% yield); ^{1}H NMR (400 MHz, $CDCl_3$): δ 6.08-6.07 (m, 1H), 2.28-2.16 (m, 2H), 2.14-2.04 (m, 6H), 2.01 (s, 3H), 1.79-1.67 (m, 4H), 1.61-1.53 (m, 4H); ^{13}C NMR (100 MHz, $CDCl_3$): δ 169.7, 135.1, 120.3, 86.9, 86.4, 81.2, 40.6, 29.2, 25.7, 23.4, 22.3, 21.9, 21.5; IR (KBr, v, cm^{-1}) 2946, 2213, 1747, 1682, 1221, 1018, 720; HRMS (ESI-TOF) calcd for $C_{15}H_{20}O_2Na$ $[M+Na]^+$ 255.1361, found 255.1368.

(8R,9S,13S,14S)-3-(Benzyloxy)-13-methyl-17-(3-methylbut-3-en-1-yn-1-yl)-7,8,9,11,12,13,14,15,16,17-decahydro-6H-cyclopenta[a]phenanthren-17-yl acetate (1l)

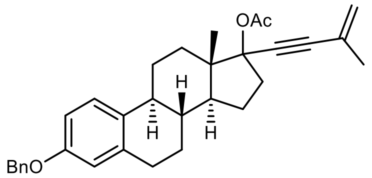

colorless oil (general procedure A, 80% yield); ^{1}H NMR (400 MHz, $CDCl_3$): δ 7.48-7.33 (m, 5H), 7.26 (d, J = 8.0 Hz, 1H), 6.84-6.81 (m, 1H), 6.76 (s, 1H), 5.34 (s, 1H), 5.25 (s, 1H), 5.06 (s, 2H), 2.89 (d, J = 3.6 Hz, 3H), 2.38 (s, 1H), 2.30-2.24 (m, 1H), 2.13 (s, 1H), 2.09 (d, J = 6.0 Hz, 3H), 2.03 (d, J = 4.0 Hz, 1H), 1.94 (s, 3H), 1.91-1.85 (m, 2H), 1.79-1.73 (m, 1H), 1.57-1.40 (m, 4H), 0.96 (d, J = 4.8 Hz, 3H); ^{13}C NMR (100 MHz, $CDCl_3$): δ 169.5, 156.8, 138.0, 137.4, 132.8, 128.6, 127.9, 127.5, 126.6, 126.5, 121.9, 114.9, 112.4, 88.2, 87.8, 85.0, 70.0, 48.3, 48.1, 43.7, 39.2, 37.5, 3.4, 29.9, 27.4, 26.5, 23.6, 23.4, 21.6, 13.63; IR (KBr, v, cm^{-1}) 2939, 1735, 1366, 1240, 1024, 903, 738; HRMS (ESI-TOF) calcd for $C_{32}H_{36}O_3Na$ $[M+Na]^+$ 491.2562, found 491.2553.

(3S,5S,8R,9S,10S,13S,14S)-10,13-Dimethyl-17-(3-methylbut-3-en-1-yn-1-yl)-3-((tetrahydro-2H-pyran-2-yl)oxy)hexadecahydro-1H-cyclopenta[a]phenanthren-17-yl acetate (1m)

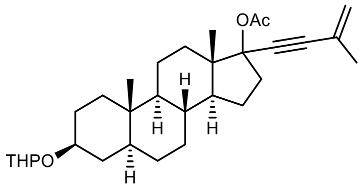

colorless oil (general procedure A, 75% yield); ^{1}H NMR (400 MHz, $CDCl_3$): δ 5.22 (s, 1H), 5.15 (s, 1H), 4.66 (d, J = 2.8 Hz, 1H), 3.88-3.83 (m, 1H), 3.67 (s, 3H), 3.55-3.51 (m, 1H), 3.43-3.40 (m, 1H), 2.67-2.57 (m, 1H), 1.96 (s, 3H), 1.83 (s, 3H), 1.78 (d, J = 7.2 Hz, 2H), 1.71-1.55 (m, 8H), 1.47 (s, 5H), 1.40-1.29 (m, 3H), 1.27-1.13 (m, 5H), 0.95-0.87 (m, 2H), 0.78 (d, J = 9.6 Hz, 6H), 0.64-0.57 (m, 1H); ^{13}C NMR (100 MHz, $CDCl_3$): δ 169.4, 126.7, 121.7, 97.0, 96.7, 94.6, 88.4, 87.7, 85.2, 62.9, 54.1, 49.1, 48.1, 45.1, 44.8, 43.6, 37.6, 36.0, 35.9, 35.8, 34.4, 33.1, 33.3, 31.7, 28.5, 27.5, 23.8, 23.6, 21.6, 21.0, 20.1, 13.7, 12.4; IR (KBr, v, cm^{-1}) 2956 , 1668, 1491, 1322, 1113, 811, 759; HRMS (ESI-TOF) calcd for $C_{31}H_{46}O_4Na$ $[M+Na]^+$ 505.3294, found 505.3294.

2.2 Preparation of 2-pyridinone-based cyclic products

2.2.1 General procedure for the synthesis of products 3-84

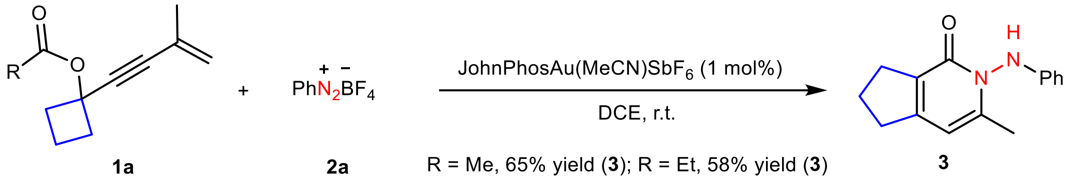

Supplementary Figure 5 Reaction Synthesis of Compound **3**

To a 10-mL Schlenk tube under air conditions, 1-(3-methylbut-3-en-1-yn-1-yl)cyclobutyl acetate (**1a**, 0.2 mmol, 1.0 equiv, 35.65 mg), benzenediazonium tetrafluoroborate (**2a**, 0.4 mmol, 2.0 equiv, 76.77 mg), JohnPhosAu(MeCN)SbF_6 (1 mol%, 1.54 mg), and 1,2-dichloroethane (2 mL) were successively added. The mixture was stirred at room temperature for 6 hours. After the reaction was completed (indicated by TLC, petroleum ether: ethyl acetate = 2:1 v/v), the reaction mixture was concentrated by vacuum distillation and purified by flash column chromatography (eluent: petroleum ether: ethyl acetate = 2:1 v/v) to afford the desired pure product **3** (31.2 mg, 65% yield) as white solid. (When 1,3-enyne acetate **1a** was replaced by 1,3-enyne propionate, the reaction proceeded under standard condition to give product **3** in 58% yield).

3-Methyl-2-(phenylamino)-2,5,6,7-tetrahydro-1H-cyclopenta[c]pyridin-1-one (3)

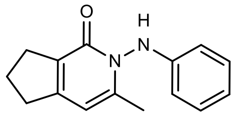

white solid (31.2 mg, 65% yield); mp: 179-180 °C; ^{1}H NMR (400 MHz, CDCl$_3$): δ 7.25-7.21 (m, 2H), 6.97-6.93 (m, 1H), 6.66 (d, J = 8.0 Hz, 2H), 6.15 (s, 1H), 2.84 (d, J = 3.6 Hz, 4H), 2.34 (s, 3H), 2.15-2.04 (m, 2H); ^{13}C NMR (100 MHz, CDCl$_3$): δ 160.4, 155.0, 147.0, 146.9, 129.3, 122.1, 114.2, 103.8, 34.4, 30.2, 23.8, 19.3; IR (KBr, v, cm^{-1}) 3221, 3026, 2957, 1645, 1557, 1444, 751, 691; HRMS (ESI-TOF) calcd for $C_{15}H_{15}N_2O$ [M-H]$^-$ 239.1184, found 239.1174.

3-Methyl-2-(o-tolylamino)-2,5,6,7-tetrahydro-1H-cyclopenta[c]pyridin-1-one (4)

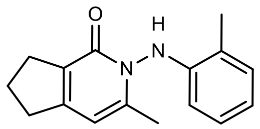

Brown solid (26.5 mg, 52% yield); mp: 136-137 °C; ^{1}H NMR (400 MHz, CDCl$_3$): δ 7.27 (s, 1H), 7.14 (d, J = 7.2 Hz, 1H), 7.04-7.00 (m, 1H), 6.90-6.87 (m, 1H), 6.24-6.10 (m, 2H), 2.88-2.83 (m, 4H), 2.40 (s, 3H), 2.33 (s, 3H), 2.13-2.05 (m, 2H); ^{13}C NMR (100 MHz, CDCl$_3$): δ 154.9, 146.6, 144.5, 130.7, 126.9, 125.1, 122.1, 111.9, 103.9, 34.4, 30.1, 23.7, 19.1, 17.1; IR (KBr, v, cm^{-1}) 3222, 2924, 2853, 1659, 1560, 1489, 748, 704; HRMS (ESI-TOF) calcd for $C_{16}H_{17}N_2O$ [M-H]$^-$ 253.1341, found 253.1335.

3-Methyl-2-(m-tolylamino)-2,5,6,7-tetrahydro-1H-cyclopenta[c]pyridin-1-one (5)

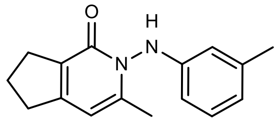

white solid (32.6 mg, 64% yield); mp: 169-171 °C; ^{1}H NMR (400 MHz, CDCl$_3$): δ 7.46 (s, 1H), 7.12-7.08 (m, 1H), 6.76 (d, J = 7.2 Hz, 1H), 6.49 (s, 1H), 6.43 (d, J = 8.0 Hz, 1H), 6.13 (s, 1H), 2.86-2.82 (m, 4H), 2.33 (s, 3H), 2.27 (s, 3H), 2.12-2.04 (m, 2H); ^{13}C NMR (100 MHz, CDCl$_3$): δ 160.5, 155.0, 147.0, 139.3, 129.3, 123.2, 115.0, 111.3, 103.8, 34.5, 30.3, 23.8, 21.7, 19.3; IR (KBr, v, cm^{-1}) 3234, 3047, 2923, 1652, 1558, 1488, 771, 692; HRMS (ESI-TOF) calcd for $C_{16}H_{17}N_2O$ [M-H]$^-$ 253.1341, found 253.1340.

3-Methyl-2-(p-tolylamino)-2,5,6,7-tetrahydro-1H-cyclopenta[c]pyridin-1-one (6)

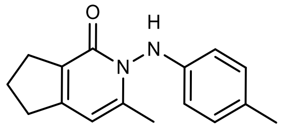

white solid (35.6 mg, 70% yield); mp: 152-153 ºC; ^{1}H NMR (400 MHz, CDCl$_3$): δ 7.42 (s, 1H), 7.03 (d, J = 8.0 Hz, 2H), 6.57 (d, J = 8.0 Hz, 2H), 6.12 (s, 1H), 2.85-2.82 (m, 4H), 2.33 (s, 3H), 2.25 (s, 3H), 2.11-2.04 (m, 2H); ^{13}C NMR (100 MHz, CDCl$_3$): δ 160.3, 154.8, 146.6, 144.4, 131.7, 129.8, 129.2, 114.5, 103.7, 34.4, 30.2, 23.7, 20.6, 19.2; IR (KBr, v, cm^{-1}) 3240, 3028, 2923, 1656, 1558, 1455, 812, 697; HRMS (ESI-TOF) calcd for $C_{16}H_{17}N_2O$ [M-H]$^-$ 253.1341, found 253.1340.

2-((4-Fluorophenyl)amino)-3-methyl-2,5,6,7-tetrahydro-1H-cyclopenta[c]pyridin-1-one (7)

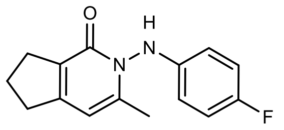

white solid (40.8 mg, 79% yield); mp: 164-165 ºC; ^{1}H NMR (400 MHz, CDCl$_3$): δ 7.63 (s, 1H), 6.91-6.87 (m, 2H), 6.61-6.57 (m, 2H), 6.12 (s, 1H), 2.85-2.81 (m, 4H), 2.30 (s, 3H), 2.11-2.04 (m, 2H); ^{13}C NMR (100 MHz, CDCl$_3$): δ 159.7 ($^1J_{CF}$ = 66.6 Hz), 157.3, 155.1, 146.6, 142.9 ($^2J_{CF}$ = 2.6 Hz), 129.3, 116.0, 115.8 ($^3J_{CF}$ = 8 Hz), 104.0, 34.4, 30.2, 23.7, 19.1; IR (KBr, v, cm^{-1}) 3240, 3062, 2957, 1656, 1559, 1431, 830, 714; HRMS (ESI-TOF) calcd for $C_{15}H_{14}FN_2O$ [M-H]$^-$ 258.1168, found 257.1080.

2-((2-Chlorophenyl)amino)-3-methyl-2,5,6,7-tetrahydro-1H-cyclopenta[c]pyridin-1-one (8)

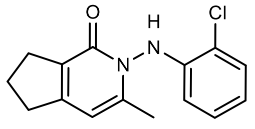

brown solid (39.6 mg, 72% yield); mp: 182-183 ºC; ^{1}H NMR (400 MHz, CDCl$_3$): δ 7.48 (s, 1H), 7.34 (d, J = 7.9 Hz, 1H), 7.07 (t, J = 7.7 Hz, 1H), 6.88 (t, J = 7.6 Hz, 1H), 6.27 (d, J = 8.1 Hz, 1H), 6.14 (s, 1H), 2.86-2.80 (m, 4H), 2.35 (s, 3H), 2.12-2.03 (m, 2H); ^{13}C NMR (100 MHz, CDCl$_3$): δ 160.0, 155.2, 146.9, 143.1, 129.9, 127.8, 122.6, 121.2, 113.3, 103.9, 34.5, 30.2, 23.7, 19.2; IR (KBr, v, cm^{-1}) 3303, 2958, 2852, 1663, 1560, 1443, 748, 685; HRMS (ESI-TOF) calcd for $C_{15}H_{14}ClN_2O$ [M-H]$^-$ 273.0795, found 273.0787.

2-((3-Chlorophenyl)amino)-3-methyl-2,5,6,7-tetrahydro-1H-cyclopenta[c]pyridin-1-one (9)

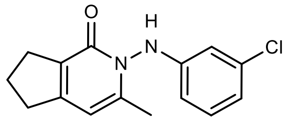

yellow solid (38.5 mg, 70% yield); mp: 143-145 °C; ^{1}H NMR (400 MHz, CDCl3): δ 7.94 (s, 1H), 7.10-7.06 (m, 1H), 6.86 (d, J = 7.2 Hz, 1H), 6.57 (s, 1H), 6.51 (d, J = 7.6 Hz, 1H), 6.13 (s, 1H), 2.87-2.83 (m, 4H), 2.30 (s, 3H), 2.13-2.05 (m, 2H); ^{13}C NMR (100 MHz, $CDCl_3$): δ 160.5, 155.5, 148.6, 147.1, 135.1, 130.4, 121.8, 113.9, 112.2, 104.2, 34.5, 30.3, 23.7, 19.2; IR (KBr, v, cm^{-1}) 3227, 3033, 2854, 1652, 1557, 1476, 768, 681; HRMS (ESI-TOF) calcd for $C_{15}H_{14}ClN_2O$ [M-H]$^-$ 273.0795, found 273.0789.

2-((4-Chlorophenyl)amino)-3-methyl-2,5,6,7-tetrahydro-1H-cyclopenta[c]pyridin-1-one (10)

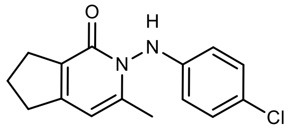

brown solid (48.4 mg, 88% yield); mp: 177-178 °C; ^{1}H NMR (400 MHz, $CDCl_3$): δ 7.76 (m, 1H), 7.19 (d, J = 8.8 Hz, 2H), 6.60 (d, J = 8.4 Hz, 2H), 6.18 (s, 1H), 2.88 (d, J = 4.0 Hz, 4H), 2.35 (s, 3H), 2.16-2.09 (m, 2H); ^{13}C NMR (100 MHz, $CDCl_3$): δ 160.3, 155.2, 146.7, 145.7, 129.5, 129.2, 126.9, 115.5, 104.0, 34.4, 30.2, 23.7, 19.1; IR (KBr, v, cm^{-1}) 3231, 3099, 2961, 1656, 1557, 1490, 821, 736; HRMS (ESI-TOF) calcd for $C_{15}H_{14}ClN_2O$ [M-H]$^-$ 273.0795, found 273.0788.

2-((4-Bromophenyl)amino)-3-methyl-2,5,6,7-tetrahydro-1H-cyclopenta[c]pyridin-1-one (11)

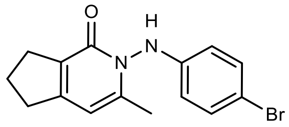

brown solid (50.4 mg, 79% yield); mp: 159-160 °C; ^{1}H NMR (400 MHz, $CDCl_3$): δ 7.82 (s, 1H), 7.29-7.24 (m, 2H), 6.52-6.49 (m, 2H), 6.13 (s, 1H), 2.83 (d, J = 4.8 Hz, 4H), 2.29 (s, 3H), 2.11-2.04 (m, 2H); ^{13}C NMR (100 MHz, $CDCl_3$): δ 160.4, 155.3, 146.9, 146.2, 132.1, 129.5, 115.7, 114.0, 104.1, 34.4, 30.2, 23.7, 19.2; IR (KBr, v, cm^{-1}) 3230, 2960, 2885, 1656, 1488, 1004, 818, 735; HRMS (ESI-TOF) calcd for $C_{15}H_{14}BrN_2O$ [M-H]$^-$ 317.0290, found 317.0306.

3-Methyl-2-((4-(trifluoromethyl)phenyl)amino)-2,5,6,7-tetrahydro-1H-cyclopenta[c]pyridin-1-one (12)

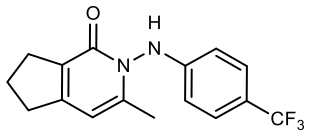

white solid (46.2 mg, 75% yield); mp: 176-177 °C; ^{1}H NMR (400 MHz, $CDCl_3$): δ 8.15 (s, 1H), 7.39 (d, J = 8.4 Hz, 2H), 6.63 (d, J = 8.0 Hz, 2H), 6.17 (s, 1H), 2.93-2.76 (m, 4H), 2.30 (s, 3H), 2.13-2.06 (m, 2H); ^{13}C NMR (100 MHz, $CDCl_3$): δ 160.6, 155.8, 150.2, 147.3, 129.9, 126.7 ($^{1}J_{CF}$ = 3.7 Hz), 113.4, 104.4, 34.5, 30.3, 23.8, 19.2; IR (KBr, v, cm^{-1}) 3223, 3027, 2966, 1659, 1558, 1422, 831, 738; HRMS (ESI-TOF) calcd for $C_{16}H_{13}F_3N_2O$ [M-H]$^-$ 307.1058, found 307.1057.

4-((3-Methyl-1-oxo-1,5,6,7-tetrahydro-2H-cyclopenta[c]pyridin-2-yl)amino)benzonitrile (13)

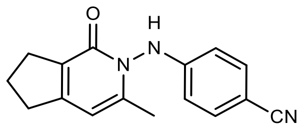

brown solid (35.6 mg, 67% yield); mp: 187-188 °C; ^{1}H NMR (400 MHz, $CDCl_3$): δ 8.47 (s 1H), 7.45 (d, J = 6.8 Hz, 2H), 6.66-6.61 (m, 2H), 6.22 (s, 1H), 2.94-2.80 (m, 4H), 2.31 (s, 3H), 2.17-2.10 (m, 2H); ^{13}C NMR (100 MHz, $CDCl_3$): δ 156.2, 151.2, 147.4, 133.7, 129.9, 119.5, 113.6, 104.83, 103.9, 34.6, 30.3, 23.8, 19.2; IR (KBr, v, cm^{-1}) 3220, 3081, 2964, 2221, 1608, 1507, 832, 736; HRMS (ESI-TOF) calcd for $C_{16}H_{14}N_3O$ [M-H]$^-$ 264.1137, found 264.1135.

3-Methyl-2-((4-nitrophenyl)amino)-2,5,6,7-tetrahydro-1H-cyclopenta[c]pyridin-1-one (14)

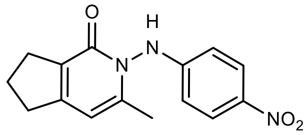

brown solid (39.4 mg, 69% yield); mp: 179-180 °C; ^{1}H NMR (400 MHz, $CDCl_3$): δ 9.08 (d, J = 5.6 Hz, 1H), 7.95 (d, J = 8.8 Hz, 2H), 6.56 (d, J = 8.8 Hz, 2H), 6.18 (s, 1H), 2.89-2.76 (m, 4H), 2.21 (s, 3H), 2.14-2.08 (m, 2H); ^{13}C NMR (100 MHz, $CDCl_3$): δ 156.5, 153.1, 147.4, 141.7, 130.0, 125.79, 112.7, 105.1, 34.6, 30.4, 23.8, 19.2; IR (KBr, v, cm^{-1}) 3202, 2989, 2958, 1655, 1598, 1456, 843, 693; HRMS (ESI-TOF) calcd for $C_{15}H_{14}N_3O_3$ [M-H]$^-$ 284.1035, found 284.1042.

3-Methyl-2-(naphthalen-2-ylamino)-2,5,6,7-tetrahydro-1H-cyclopenta[c]pyridin-1-one (15)

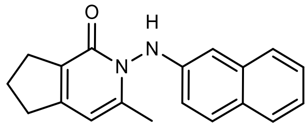

brown solid (39.5 mg, 68% yield); mp: 188-189 °C; ^{1}H NMR (400 MHz, $CDCl_3$): δ 7.89 (s, 1H), 7.71 (d, J = 8.0 Hz, 1H), 7.67-7.63 (m, 1H), 7.58 (d, J = 8.0 Hz, 1H), 7.39-7.35 (m, 1H), 7.32-7.28 (m, 1H), 7.07 (d, J = 8.8 Hz, 1H), 6.70 (s, 1H), 6.18 (s, 1H), 2.90-2.87 (m, 4H), 2.36 (s, 3H), 2.15-2.08 (m, 2H); ^{13}C NMR (100 MHz, $CDCl_3$): δ 160.4, 155.2, 147.0, 144.8, 134.3, 129.9, 129.5, 129.4, 127.7, 126.8, 126.4, 123.9, 123.8, 116.8, 108.1, 104.0, 34.5, 30.3, 23.8, 19.2; IR (KBr, ν, cm^{-1}) 3225, 3054, 2913, 1636, 1558, 1470, 811, 699; HRMS (ESI-TOF) calcd for $C_{19}H_{17}N_2O$ [M-H]$^-$ 289.1341, found 289.1355.

(1S,2R,4S)-1,7,7-trimethylbicyclo[2.2.1]heptan-2-yl 4-((3-methyl-1-oxo-1,5,6,7-tetrahydro-2H-cyclopenta[c]pyridin-2-yl)amino)benzoate (16)

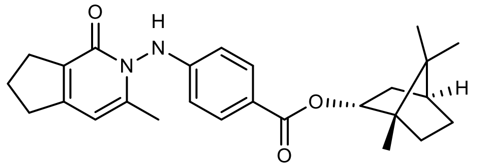

yellow solid (48.8 mg, 60% yield); mp: 187-188 °C; ^{1}H NMR (400 MHz, $CDCl_3$): δ 7.92 (d, J = 8.4 Hz, 2H), 7.85 (s, 1H), 6.65 (d, J = 8.8 Hz, 2H), 6.15 (s, 1H), 5.09-5.04 (m, 1H), 2.87-2.81 (m, 4H), 2.46-2.40 (m, 1H), 2.31 (s, 3H), 2.12-2.04 (m, 3H), 1.81-1.73 (m, 1H), 1.72-1.70 (m, 1H), 1.39-1.33 (m, 1H), 1.29-1.24 (m, 1H), 1.10-1.05 (m, 1H), 0.95 (s, 3H), 0.90-0.87 (m, 6H); ^{13}C NMR (100 MHz, $CDCl_3$): δ 166.5, 160.4, 155.5, 151.2, 146.9, 131.3, 129.7, 113.2, 104.1, 80.1, 49.2, 47.9, 45.1, 37.0, 34.5, 30.2, 28.2, 27.5, 23.7, 19.8, 19.2, 19.0, 13.7; IR (KBr, ν, cm^{-1}) 3228, 2954, 2880, 1659, 1558, 1271, 1016, 736; HRMS (ESI-TOF) calcd for $C_{26}H_{31}N_2O_3$ [M-H]$^-$ 419.2335, found 419.2330.

(1R,2S,5R)-2-Isopropyl-5-methylcyclohexyl 4-((3-methyl-1-oxo-1,5,6,7-tetrahydro-2H-cyclopenta[c]pyridin-2-yl)amino)benzoate (17)

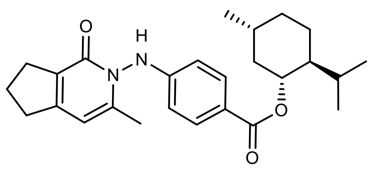

yellow solid (45.6 mg, 54% yield); mp: 109-111 °C; ^{1}H NMR (400 MHz, $CDCl_3$): δ 7.99-7.91 (m, 1H), 7.89 (d, J = 8.4 Hz, 2H), 6.62 (d, J = 8.4 Hz, 2H), 6.15 (s, 1H), 4.89-4.83 (m, 1H), 2.88-2.78 (m, 4H), 2.30 (s, 3H), 2.11-2.05 (m, 3H), 1.94-1.87 (m, 1H), 1.70 (d, J = 12.0 Hz, 2H), 1.55-1.46 (m, 2H), 1.13-1.03 (m, 2H), 0.96-0.86 (m, 8H), 0.76 (d, J = 6.8 Hz, 3H); ^{13}C NMR (100 MHz, $CDCl_3$): δ 165.9, 155.6, 151.3, 147.1, 131.4, 129.8, 124.3, 113.1, 104.2, 74.5, 47.5, 41.2, 34.5, 31.6, 30.3, 26.6, 23.8,

22.2, 20.9, 19.2, 16.7; IR (KBr, *v*, cm^{-1}) 3229, 2956, 2869, 1659, 1559, 1274, 963, 769; HRMS (ESI-TOF) calcd for $C_{26}H_{33}N_2O_3$ $[M-H]^-$ 421.2491, found 421.2483.

(3s,5s,7s)-Adamantan-1-yl 4-((3-methyl-1-oxo-1,5,6,7-tetrahydro-2H-cyclopenta[c]pyridin-2-yl)amino)benzoate (18)

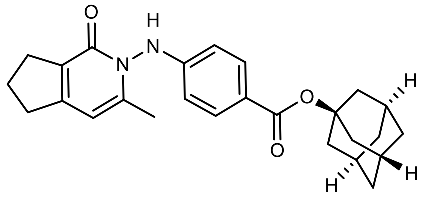

yellow solid (42.7 mg, 51% yield); mp: 130-131 ºC;^{1}H NMR (400 MHz, $CDCl_3$): δ 7.86 (d, J = 8.4 Hz, 2H), 7.61 (s, 1H), 6.63 (d, J = 8.4 Hz, 2H), 6.15 (s, 1H), 2.87-2.81 (m, 4H), 2.31 (s, 3H), 2.21 (s, 8H), 2.10-2.05 (m, 2H), 1.77-1.63 (m, 7H); ^{13}C NMR (100 MHz, $CDCl_3$): δ 165.2, 155.3, 150.7, 146.7, 131.2, 129.6, 125.8, 113.2, 104.0, 80.5, 41.5, 36.3, 34.4, 30.9, 30.1, 23.6, 19.1; IR (KBr, *v*, cm^{-1}) 3234, 2911, 2874, 1653, 1558, 1271, 1055, 770; HRMS (ESI-TOF) calcd for $C_{26}H_{29}N_2O_3$ $[M-H]^-$ 417.2178, found 417.2164.

3-Methyl-2-(phenylamino)-5,6,7,8-tetrahydroisoquinolin-1(2H)-one (19)

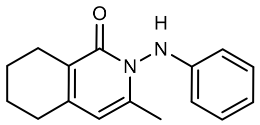

white solid (36.1 mg, 71% yield); mp: 167-168 ºC; ^{1}H NMR (400 MHz, $CDCl_3$): δ 7.24-7.21 (m, 2H), 6.97-6.93 (m, 1H), 6.66 (d, J = 8.0 Hz, 2H), 5.90 (s, 1H), 2.54 (d, J = 13.2 Hz, 4H), 2.30 (s, 3H), 1.75 (s, 4H). ^{13}C NMR (100 MHz, $CDCl_3$): δ 162.2, 147.3, 143.7, 129.4, 124.5, 122.2, 114.3, 107.5, 29.4, 23.6, 22.2, 22.1, 18.6; IR (KBr, *v*, cm^{-1}) 3241, 2929, 1648, 1563, 1243, 751, 692; HRMS (ESI-TOF) calcd for $C_{16}H_{17}N_2O$ $[M-H]^-$ 253.1341, found 253.1335.

3-Methyl-2-(o-tolylamino)-5,6,7,8-tetrahydroisoquinolin-1(2H)-one (20)

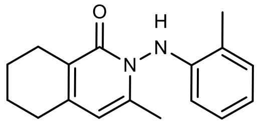

brown solid (27.4 mg, 51% yield); mp: 112-113 ºC; ^{1}H NMR (400 MHz, $CDCl_3$): δ 7.14 (d, J = 7.2 Hz, 1H), 7.04-7.01 (m, 1H), 6.90-6.86 (m, 1H), 6.18 (d, J = 8.0 Hz, 1H), 5.94 (s, 1H), 2.55 (d, J = 16.4

Hz, 4H), 2.40 (s, 3H), 2.30 (s, 3H), 1.76 (s, 4H). ^{13}C NMR (100 MHz, CDCl$_3$): δ 147.2, 144.6, 143.5, 130.7, 126.9, 124.9, 122.0, 111.8, 107.6, 29.3, 23.5, 22.1, 22.0 18.4, 17.1; IR (KBr, *v*, cm^{-1}) 3247, 1653, 1559, 1385, 762, 669; HRMS (ESI-TOF) calcd for $C_{17}H_{19}N_2O$ [M-H]$^-$ 267.1497, found 267.1484.

3-Methyl-2-(m-tolylamino)-5,6,7,8-tetrahydroisoquinolin-1(2H)-one (21)

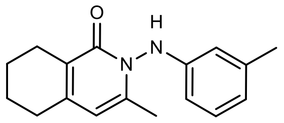

brown solid (37.0 mg, 69% yield); mp: 148-149 ºC; ^{1}H NMR (400 MHz, CDCl$_3$): δ 7.11-7.07 (m, 1H), 6.75 (d, J = 7.2 Hz, 1H), 6.50 (s, 1H), 6.42 (d, J = 8.0 Hz, 1H), 5.89 (s, 1H), 2.53 (s, 4H), 2.30 (s, 3H), 2.27 (s, 3H), 1.75 (s, 4H). ^{13}C NMR (100 MHz, CDCl$_3$): δ 162.3, 147.3, 143.8, 139.3, 129.2, 124.5, 123.1, 115.0, 111.2, 107.6, 29.4, 23.6, 22.2, 22.1, 21.7, 18.6; IR (KBr, *v*, cm^{-1}) 3242, 2929, 1652, 1563, 1265, 1164, 771; HRMS (ESI-TOF) [M-H]$^-$ calcd for $C_{17}H_{19}N_2O$ 267.1497, found 267.1486.

3-Methyl-2-(p-tolylamino)-5,6,7,8-tetrahydroisoquinolin-1(2H)-one (22)

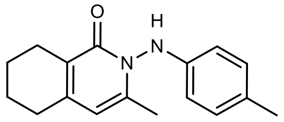

brown solid (40.8 mg, 76% yield); mp: 148-149 ºC; ^{1}H NMR (400 MHz, CDCl$_3$): δ 7.03 (d, J = 8.4 Hz, 2H), 6.57 (d, J = 8.4 Hz, 2H), 5.88 (s, 1H), 2.53 (d, J = 12.0 Hz, 4H), 2.30 (s, 3H), 2.25 (s, 3H), 1.74 (s, 4H). ^{13}C NMR (100 MHz, CDCl$_3$): δ 162.1, 147.2, 144.6, 143.6, 131.6, 129.9, 124.4, 114.4, 107.5, 29.3, 23.6, 22.2, 22.1, 20.7, 18.6; IR (KBr, *v*, cm^{-1}) 3240, 2927, 1652, 1564, 1243, 812; HRMS (ESI-TOF) calcd for $C_{17}H_{19}N_2O$ [M-H]$^-$ 267.1497, found 267.1490.

2-((4-Fluorophenyl)amino)-3-methyl-5,6,7,8-tetrahydroisoquinolin-1(2H)-one (23)

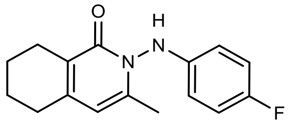

white solid (44.6 mg, 82% yield); mp: 144-146 ºC; ^{1}H NMR (400 MHz, CDCl$_3$): δ δ 6.93-6.89 (m, 2H), 6.63-6.60 (m, 2H), 5.89 (s, 1H), 2.53 (d, J = 11.2 Hz, 4H), 2.28 (s, 3H), 1.74 (s, 4H). ^{13}C NMR (100 MHz, CDCl$_3$): δ 162.1, 158.5 ($^1J_{CF}$ = 4 Hz), 147.4, 143.5, 143.1, 124.5, 116.0, 115.8 ($^2J_{CF}$ = 13.1 Hz), 107.7, 29.3, 23.5, 22.1, 22.0, 18.5; IR (KBr, *v*, cm^{-1}) 3250, 2960, 1647, 1507, 1215, 1107, 833;

HRMS (ESI-TOF) calcd for $C_{16}H_{16}FN_2O$ $[M-H]^-$ 271.1247, found 271.1248.

2-((2-Chlorophenyl)amino)-3-methyl-5,6,7,8-tetrahydroisoquinolin-1(2H)-one (24)

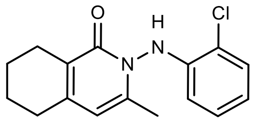

brown solid (45.5 mg, 79% yield); mp: 137-138 °C; ^{1}H NMR (400 MHz, $CDCl_3$): δ 7.47 (s, 1H), 7.33 (d, J = 8.0 Hz, 1H), 7.08-7.04 (m, 1H), 6.89-6.85 (m, 1H), 6.29 (d, J = 8.0 Hz, 1H), 5.89 (s, 1H), 2.51 (d, J = 20.0 Hz, 4H), 2.30 (s, 3H), 1.73 (s, 4H). ^{13}C NMR (100 MHz, $CDCl_3$): δ 161.7, 147.5, 143.7, 143.2, 129.78, 127.7, 124.8, 122.4, 121.0, 113.2, 107.6, 29.3, 23.5, 22.1, 22.0, 18.5; IR (KBr, v, cm^{-1}) 3300, 2930, 1655, 1505, 1299, 1051, 775; HRMS (ESI-TOF) calcd for $C_{16}H_{16}ClN_2O$ $[M-H]^-$ 287.0951, found 287.0970.

2-((3-Chlorophenyl)amino)-3-methyl-5,6,7,8-tetrahydroisoquinolin-1(2H)-one (25)

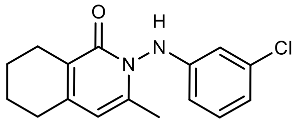

white solid (48.4 mg, 84% yield); mp: 108-110 °C; ^{1}H NMR (400 MHz, $CDCl_3$): δ 7.71 (s, 1H), 7.13-7.09 (m, 1H), 6.88 (d, J = 8.0 Hz, 1H), 6.59 (s, 1H), 6.54 (d, J = 8.0 Hz, 1H), 5.90 (s, 1H), 2.53 (d, J = 9.2 Hz, 4H), 2.27 (s, 3H), 1.75 (s, 4H). ^{13}C NMR (100 MHz, CDCl3): δ 162.2, 148.6, 147.7, 143.8, 135.1, 130.3, 124.7, 121.8, 113.9, 112.2, 107.8, 29.3, 23.6, 22.1, 22.0, 18.5; IR (KBr, v, cm^{-1}) 3223, 2928, 1648, 1561, 1243, 1078, 765; HRMS (ESI-TOF) calcd for $C_{16}H_{16}ClN_2O$ $[M-H]^-$ 287.0951, found 287.0938.

2-((4-Chlorophenyl)amino)-3-methyl-5,6,7,8-tetrahydroisoquinolin-1(2H)-one (26)

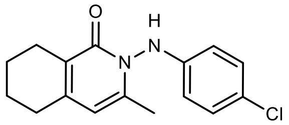

brown solid (53.0 mg, 92% yield); mp: 144-145 °C; ^{1}H NMR (400 MHz, $CDCl_3$): δ 7.54 (s, 1H), 7.21 (d, J = 8.8 Hz, 2H), 6.63 (d, J = 8.8 Hz, 2H), 5.94 (s, 1H), 2.57 (d, J = 17.6 Hz, 4H), 2.32 (s, 3H), 1.79 (s, 4H). ^{13}C NMR (100 MHz, $CDCl_3$): δ 162.2, 147.7, 145.9, 143.6, 129.4, 127.1, 124.7, 115.6, 107.8, 29.4, 23.6, 22.2, 22.1, 18.6; IR (KBr, v, cm^{-1}) 3232, 2930, 1652, 1561, 1242, 1092, 821; HRMS (ESI-TOF) calcd for $C_{16}H_{16}ClN_2O$ $[M-H]^-$ 287.0951, found 287.0937.

2-((4-Bromophenyl)amino)-5,6,7,8-tetrahydroisoquinolin-1(2H)-one (27)

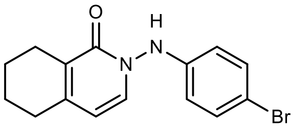

brown solid (53.0 mg, 83% yield); mp: 153-155 ºC; ^{1}H NMR (400 MHz, CDCl$_3$): δ 8.18 (d, J = 63.6 Hz, 1H), 7.47-7.43 (m, 2H), 6.74-6.64 (m, 2H), 5.97 (s, 1H), 2.58 (d, J = 21.6 Hz, 4H), 1.80 (s, 4H). ^{13}C NMR (100 MHz, CDCl$_3$): δ 162.3, 150.3, 148.0, 143.9, 126.7, 124.9, 113.4, 108.0, 29.4, 23.6, 22.1, 22.0, 18.5; IR (KBr, v, cm^{-1}) 3228, 2941, 1651, 1563, 1327, 1112, 831; HRMS (ESI-TOF) calcd for $C_{15}H_{14}BrN_2O$ [M-H]$^-$ 317.0290, found 317.0277.

3-Methyl-2-((4-(trifluoromethyl)phenyl)amino)-5,6,7,8-tetrahydroisoquinolin-1(2H)-one (28)

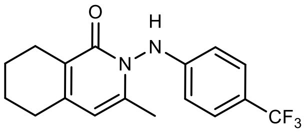

white solid (50.9 mg, 79% yield); mp: 140-142 ºC; ^{1}H NMR (400 MHz, CDCl$_3$): δ 8.14 (s, 1H), 7.40 (d, J = 8.4 Hz, 2H), 6.64 (d, J = 8.0 Hz, 2H), 5.92 (s, 1H), 2.53 (d, J = 21.2 Hz, 4H), 2.26 (s, 3H), 1.75 (s, 4H). ^{13}C NMR (100 MHz, CDCl$_3$): δ 162.3, 150.3, 148.0, 144.0, 126.6, 125.8, 124.8, 123.1, 113.2, 108.0, 29.3, 23.6, 22.1, 22.0, 18.5; IR (KBr, v, cm^{-1}) 3228, 2939, 1652, 1563, 1327, 1112, 831; HRMS (ESI-TOF) calcd for $C_{17}H_{16}F_3N_2O$ [M-H]$^-$ 321.1215, found 321.1203.

4-((3-Methyl-1-oxo-5,6,7,8-tetrahydroisoquinolin-2(1H)-yl)amino)benzonitrile (29)

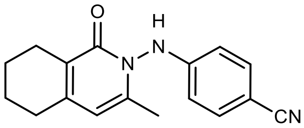

brown solid (39.7 mg, 71% yield); mp: 93-94 ºC; ^{1}H NMR (400 MHz, CDCl$_3$): δ 8.17 (s, 1H), 7.43 (d, J = 8.0 Hz, 2H), 6.62 (d, J = 8.0 Hz, 2H), 5.93 (s, 1H), 2.51 (d, J = 33.6 Hz, 4H), 2.24 (s, 3H), 1.74 (s, 4H). ^{13}C NMR (100 MHz, CDCl$_3$): δ 162.2, 151.2, 148.3, 143.8, 133.6, 124.9, 119.4, 113.5, 108.3, 104.0, 29.4, 23.6, 22.0, 21.9, 18.4; IR (KBr, v, cm^{-1}) 3223, 2932, 2220, 1651, 1561, 1267, 1172, 831; HRMS (ESI-TOF) calcd for $C_{17}H_{16}N_3O$ [M-H]$^-$ 278.1293, found 278.1290.

3-Methyl-2-((4-nitrophenyl)amino)-5,6,7,8-tetrahydroisoquinolin-1(2H)-one (30)

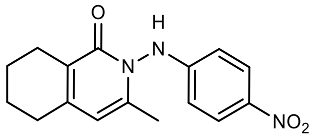

brown solid (44.3 mg, 74% yield); mp: 113-114 ºC; ^{1}H NMR (400 MHz, CDCl$_3$): δ 8.65 (d, J = 88.0 Hz, 1H), 8.02 (d, J = 8.8 Hz, 2H), 6.60 (d, J = 8.4 Hz, 2H), 5.95 (s, 1H), 2.66 - 2.38 (m, 4H), 2.21 (d, J = 9.2 Hz, 3H), 1.76 (s, 4H). ^{13}C NMR (100 MHz, CDCl$_3$): δ 162.2, 153.0, 148.6, 143.9, 141.5, 125.7, 125.0, 112.5, 108.5, 29.4, 23.6, 22.0, 21.9, 18.4; IR (KBr, v, cm^{-1}) 3202, 2399, 1652, 1560, 1330, 1110, 751; HRMS (ESI-TOF) calcd for $C_{16}H_{16}N_3O_3$ [M-H]$^-$ 298.1192, found 298.1172.

3-Methyl-2-(naphthalen-2-ylamino)-5,6,7,8-tetrahydroisoquinolin-1(2H)-one (31)

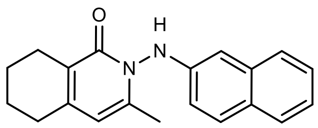

brown solid (40.8 mg, 67% yield); mp: 118-119 ºC; ^{1}H NMR (400 MHz, CDCl$_3$): δ 7.72 (d, J = 8.8 Hz, 2H), 7.60 (d, J = 8.4 Hz, 1H), 7.40-7.36 (m, 1H), 7.33-7.28 (m, 1H), 7.11-7.08 (m, 1H), 6.73 (s, 1H), 5.95 (s, 1H), 2.57 (d, J = 18.0 Hz, 4H), 2.34 (s, 3H), 1.78 (s, 4H). ^{13}C NMR (100 MHz, CDCl$_3$): δ 162.1, 147.5, 144.8, 143.7, 134.3, 130.0, 129.5, 127.8, 126.9, 126.5, 124.6, 124.0, 116.7, 108.4, 107.7, 29.4, 23.6, 22.1, 18.5; IR (KBr, v, cm^{-1}) 3231, 2930, 1652, 1563, 1265, 951, 843; HRMS (ESI-TOF) calcd for $C_{20}H_{19}N_2O$ [M-H]$^-$ 303.1497, found 303.1483.

(1S,2R,4S)-1,7,7-trimethylbicyclo[2.2.1]heptan-2-yl 4-((3-methyl-1-oxo-5,6,7,8-tetrahydroisoquinolin-2(1H)-yl)amino)benzoate (32)

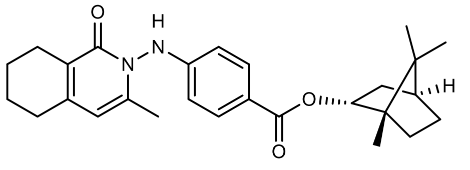

brown solid (54.8 mg, 58% yield); mp: 110-111 ºC; ^{1}H NMR (400 MHz, CDCl$_3$): δ 7.91 (d, J = 8.4 Hz, 2H), 6.64 (d, J = 8.4 Hz, 2H), 5.90 (s, 1H), 5.09-5.02 (m, 1H), 2.54-2.42 (m, 5H), 2.26 (s, 3H), 2.10-2.04 (m, 1H), 1.78-1.68 (m, 6H), 1.37 (d, J = 12.4 Hz, 1H), 1.25 (s, 1H), 1.09-1.03 (m, 1H), 0.93 (d, J = 6.0 Hz, 3H), 0.89 (s, 3H), 0.87 (d, J = 2.0 Hz, 3H). ^{13}C NMR (100 MHz, CDCl$_3$): δ 166.6, 151.4, 147.9, 143.9, 131.3, 124.9, 124.1, 113.0, 107.9, 80.1, 49.2, 47.9, 45.1, 37.0, 29.4, 28.2, 27.4, 23.6, 22.1, 22.0, 19.8, 19.0, 18.5, 13.7; IR (KBr, v, cm^{-1}) 3227, 2935, 1644, 1547, 1288, 1093, 737; HRMS (ESI-TOF) calcd for $C_{27}H_{33}N_2O_3$ [M-H]$^-$ 433.2491, found 433.2494.

(1R,2S,5R)-2-Isopropyl-5-methylcyclohexyl -4-((3-methyl-1-oxo-5,6,7,8-tetrahydroisoquinolin-2(1H)-yl)amino)benzoate (33)

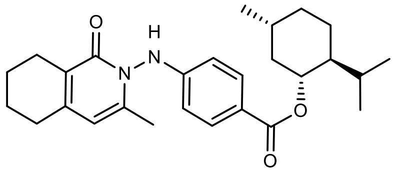

brown solid (50.6 mg, 63% yield); mp: 102-103 ºC; ^{1}H NMR (400 MHz, $CDCl_3$): δ 8.03 (d, J = 8.4 Hz, 1H), 7.88 (d, J = 8.4 Hz, 2H), 6.61 (d, J = 8.4 Hz, 2H), 5.89 (s, 1H), 4.89-4.82 (m, 1H), 2.50 (d, J = 24.0 Hz, 4H), 2.24 (s, 3H), 2.10-2.05 (m, 1H), 1.94-1.87 (m, 1H), 1.70 (d, J = 17.2 Hz, 6H), 1.55-1.46 (m, 2H), 1.13-1.02 (m, 2H), 0.91-0.87 (m, 7H), 0.76 (d, J = 6.8 Hz, 3H). ^{13}C NMR (100 MHz, $CDCl_3$): δ 165.9, 151.4, 147.9, 131.4, 124.8, 112.9, 107.9, 74.3, 47.4, 41.1, 34.4, 31.5, 29.4, 26.6, 23.6, 22.1, 20.8, 18.5, 16.6; IR (KBr, v, cm^{-1}) 3230, 2930, 1706, 1610, 1267, 1109, 737; HRMS (ESI-TOF) calcd for $C_{27}H_{35}N_2O_3$ $[M-H]^-$ 435.2648, found 435.2655.

(3s,5s,7s)-Adamantan-1-yl 4-((3-methyl-1-oxo-5,6,7,8-tetrahydroisoquinolin-2(1H)-yl)amino)benzoate (34)

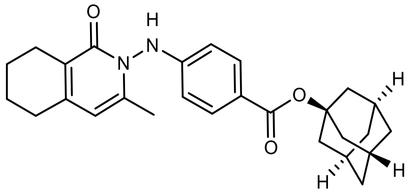

brown solid (60.6 mg, 70% yield); mp: 188-190 ºC; ^{1}H NMR (400 MHz, $CDCl_3$): δ 7.85 (d, J = 8.4 Hz, 2H), 6.62 (d, J = 8.4 Hz, 2H), 5.91 (s, 1H), 2.52 (d, J = 26.4 Hz, 4H), 2.27 (s, 3H), 2.21 (s, 10H), 1.71 (d, J = 16.4 Hz, 9H). ^{13}C NMR (100 MHz, $CDCl_3$): δ 213.6, 173.3, 143.5, 136.1, 135.4, 134.7, 133.3, 131.4, 130.3, 129.6, 128.1, 127.3, 126.2, 126.1, 124.5, 47.8, 45.4, 41.8, 28.6, 24.0, 21.6, 15.6; IR (KBr, v, cm^{-1}) 3234, 2912, 1705, 1609, 1270, 1116, 770; HRMS (ESI-TOF) calcd for $C_{27}H_{31}N_2O_3$ $[M-H]^-$ 431.2335, found 431.2340.

2-((4-Chlorophenyl)amino)-3-methyl-6-phenyl-2,5,6,7-tetrahydro-1H-cyclopenta[c]pyridin-1-one (35)

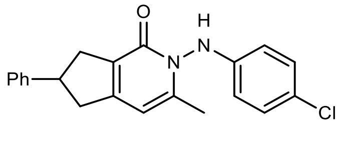

brown solid (49.8 mg, 71% yield); mp: 155-156 ºC; ^{1}H NMR (400 MHz, $CDCl_3$): δ 7.74 (s, 1H), 7.35-7.28 (m, 4H), 7.26-7.22 (m, 1H), 7.17 (d, J = 8.4 Hz, 2H), 6.59 (d, J = 8.8 Hz, 2H), 6.17 (s, 1H), 3.78-3.69 (m, 1H), 3.38-3.28 (m, 2H), 3.06-2.93 (m, 2H), 2.35 (s, 3H); ^{13}C NMR (100 MHz, $CDCl_3$): δ 160.1, 154.0, 147.5, 145.6, 129.3, 128.6, 128.3, 126.9, 126.5, 115.4, 103.9, 43.79, 42.4, 38.3, 19.3; IR (KBr, v, cm^{-1}) 3231, 3027, 2854, 1652, 1558, 1240, 1091, 700; HRMS (ESI-TOF) calcd for $C_{21}H_{18}ClN_2O$ [M-H]$^-$ 349.1108, found 349.1104.

2-((4-Chlorophenyl)amino)-3-methyl-5-(o-tolyl)-2,5,6,7-tetrahydro-1H-cyclopenta[c]pyridin-1-one (36)

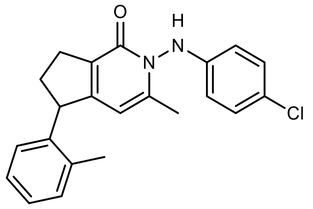

yellow solid (45.2 mg, 62% yield); mp: 172-173 ºC; ^{1}H NMR (400 MHz, $CDCl_3$): δ 7.35 (s, 1H), 7.17-7.13 (m, 3H), 7.07-7.01 (m, 2H), 6.75 (d, J = 7.6 Hz, 1H), 6.54 (d, J = 7.6 Hz, 2H), 6.21 (s, 1H), 4.61 (d, J = 7.2 Hz, 1H), 3.04-2.98 (m, 1H), 2.85 (d, J = 14.4 Hz, 1H), 2.67-2.58 (m, 1H), 2.42 (s, 3H), 2.35 (s, 3H), 1.93 (s, 1H); ^{13}C NMR (100 MHz, CDC13): δ 159.8, 156.2, 147.8, 145.7, 142.6, 135.6, 131.5, 130.5, 129.3, 126.0, 115.5, 103.9, 103.8, 45.1, 33.1, 29.8, 20.0, 19.31; IR (KBr, v, cm^{-1}) 3231, 2964, 2861, 1655, 1559, 1490, 821, 739; HRMS (ESI-TOF) calcd for $C_{22}H_{20}ClN_2O$ [M-H]$^-$ 363.1264, found 363.1276.

5-(2-Chlorophenyl)-2-((4-chlorophenyl)amino)-3-methyl-2,5,6,7-tetrahydro-1H-cyclopenta[c]pyridin-1-one (37)

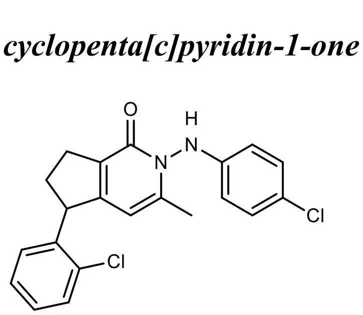

brown solid (45.5 mg, 59% yield); mp: 173-174 ºC; ^{1}H NMR (400 MHz, $CDCl_3$): δ 7.57 (s, 1H), 7.43 (d, J = 8.4 Hz, 1H), 7.19 (d, J = 8.4 Hz, 3H), 7.07 (d, J = 26.0 Hz, 1H), 6.61 (d, J = 8.4 Hz, 2H), 5.91 (s, 1H), 4.84 (s, 1H), 3.05-2.95 (m, 1H), 2.92-2.88 (m, 1H), 2.69 (m, 1H), 2.28 (s, 3H), 2.73-2.66 (m, 1H); ^{13}C NMR (100 MHz, $CDCl_3$): δ 160.4, 156.1, 147.5, 145.5, 134.0, 129.4, 128.2, 127.4, 127.3,

115.7, 103.8, 49.3, 33.4, 29.19, 19.3; IR (KBr, *v*, cm^{-1}) 3220, 2926, 2896, 1653, 1559, 1490, 1092, 755; HRMS (ESI-TOF) calcd for $C_{21}H_{17}Cl_2N_2O$ $[M-H]^-$ 383.0718, found 383.0718.

5-(4-(tert-Butyl)phenyl)-2-((4-chlorophenyl)amino)-3-methyl-2,5,6,7-tetrahydro-1H-cyclopenta[c]pyridin-1-one (38)

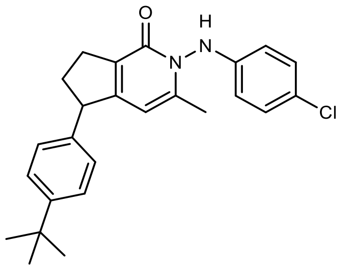

white solid (33.7 mg, 51% yield); mp: 201-203 °C;^{1}H NMR (400 MHz, $CDCl_3$): δ 7.25 (s, 2H), 7.13 (d, J = 6.0 Hz, 3H), 7.05 (s, 1H), 6.51 (d, J = 8.4 Hz, 2H), 6.20 (s, 1H), 4.39 (d, J = 8.8 Hz, 1H), 3.13-3.06 (m, 1H), 2.92-2.80 (m, 1H), 2.63-2.55 (m, 1H), 2.32 (s, 3H), 2.16-2.12 (m, 1H), 1.27 (s, 9H); ^{13}C NMR (100 MHz, $CDCl_3$): δ 160.0, 155.3, 148.8, 147.9, 145.8, 141.6, 129.3, 126.8, 125.3, 115.4, 103.8, 48.6, 34.4, 33.8, 33.5, 31.5, 19.3; IR (KBr, *v*, cm^{-1}) 3219, 2962, 1653, 1558, 1249, 819, 740; HRMS (ESI-TOF) calcd for $C_{25}H_{26}ClN_2O$ $[M-H]^-$ 405.1734, found 405.1735.

2-((4-Chlorophenyl)amino)-3-methyl-4b,5,7a,8-tetrahydropentaleno[2,1-c]pyridin-1(2H)-one (39)

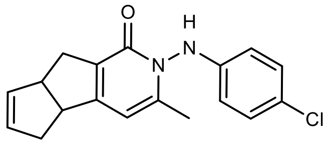

white solid (43.2 mg, 69% yield); mp: 167-168 °C; ^{1}H NMR (400 MHz, $CDCl_3$): δ 7.83 (s, 1H), 7.10 (d, J = 8.0 Hz, 2H), 6.52 (d, J = 8.0 Hz, 2H), 6.10 (s, 1H), 5.68-5.58 (m, 2H), 3.76 (d, J = 6.4 Hz, 1H), 3.59 (s, 1H), 3.01 (d, J = 8.8 Hz, 1H), 2.84-2.77 (m, 2H), 2.47 (d, J = 15.2 Hz, 1H), 2.30 (s, 3H); ^{13}C NMR (100 MHz, $CDCl_3$): δ 160.7, 157.7, 147.6, 145.7, 134.6, 129.2, 128.4, 127.5, 126.6, 115.3, 103.5, 49.1, 48.5, 37.9, 35.3, 19.3; IR (KBr, *v*, cm^{-1}) 3231, 2919, 2851, 1655, 1559, 1490, 1092, 821; HRMS (ESI-TOF) calcd for $C_{18}H_{16}ClN_2O$ $[M-H]^-$ 311.0951, found 311.0962.

5-((4-Chlorophenyl)amino)-1,2,3,5,6,7,8,9-octahydro-4H-cyclopenta[c]quinolin-4-one (40)

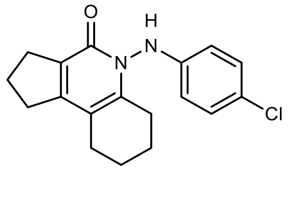

brown solid (39.0 mg, 62% yield); mp: 165-166 °C; ^{1}H NMR (400 MHz, CDCl$_3$): δ 7.82 (s, 1H), 7.10 (d, J = 8.4 Hz, 2H), 6.53 (d, J = 8.8 Hz, 2H), 2.96-2.75 (m, 5H), 2.50-2.38 (m, 3H), 2.11-2.05 (m, 2H), 1.73-1.68 (m, 4H); ^{13}C NMR (100 MHz, CDCl$_3$): δ 159.7, 155.7, 146.0, 144.2, 129.1, 128.8, 126.5, 115.4, 112.8, 32.7, 30.4, 25.6, 25.0, 23.3, 22.3, 21.9; IR (KBr, v, cm^{-1}) 3227, 2939, 2861, 1652, 1549, 1264, 822, 736; HRMS (ESI-TOF) calcd for $C_{18}H_{18}ClN_2O$ [M-H]$^-$ 313.1108, found 313.1108.

2-((4-Chlorophenyl)amino)-3,6-dimethyl-5,6,7,8-tetrahydroisoquinolin-1(2H)-one (41)

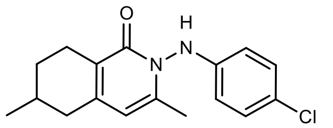

brown solid (41.7 mg, 69% yield); mp: 104-105 °C; ^{1}H NMR (400 MHz, CDCl$_3$): δ 7.23 (d, J = 8.4 Hz, 2H), 6.64 (d, J = 8.4 Hz, 2H), 6.05 (s, 1H), 2.73 (s, 1H), 2.62-2.47 (m, 2H), 2.35 (s, 3H), 1.91-1.74 (m, 3H), 1.59 (d, J = 6.8 Hz, 1H), 1.31 (s, 3H). ^{13}C NMR (100 MHz, CDCl$_3$): δ 145.7, 143.4, 129.3, 127.2, 124.4, 115.6, 106.7, 32.5, 30.0, 24.0, 21.32, 18.96, 18.6; IR (KBr, v, cm^{-1}) 3231, 2931, 1653, 1559 , 1248, 1092, 820; HRMS (ESI-TOF) calcd for $C_{17}H_{18}ClN_2O$ [M-H]$^-$ 301.1108, found 301.1098.

2-((4-Chlorophenyl)amino)-6-cyclopentyl-3-methyl-5,6,7,8-tetrahydroisoquinolin-1(2H)-one (42)

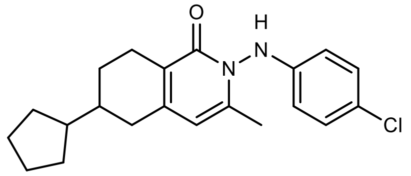

brown solid (45.0 mg, 63% yield); mp: 78-80 °C; ^{1}H NMR (400 MHz, CDCl$_3$): δ 7.48 (s, 1H), 7.17 (d, J = 8.4 Hz, 2H), 6.57 (d, J = 8.5 Hz, 2H), 6.02 (s, 1H), 2.48 (d, J = 27.2 Hz, 3H), 2.29 (s, 3H), 2.06 (d, J = 7.9 Hz, 1H), 1.78-1.53 (m, 10H), 1.26 (s, 2H). ^{13}C NMR (100 MHz, CDCl$_3$): δ 151.6, 145.8, 142.6, 129.4, 127.1, 124.7, 115.6, 44.4, 32.0, 31.0, 29.8, 25.5, 24.6, 23.5, 18.7, 18.3; IR (KBr, v, cm^{-1}) 3231, 2931, 1653, 1490, 1247, 1092, 821; HRMS (ESI-TOF) calcd for $C_{21}H_{24}ClN_2O$ [M-H]$^-$ 355.1577, found 355.1591.

5-((4-Chlorophenyl)amino)-1,3,4,5,7,8,9,10-octahydrophenanthridin-6(2H)-one (43)

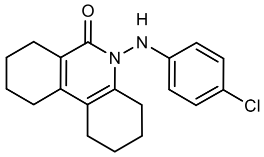

brown solid (47.4 mg, 72% yield); mp: 110-111 ºC; ^{1}H NMR (400 MHz, $CDCl_3$): δ 7.66 (s, 1H), 7.12 (d, J = 8.4 Hz, 2H), 6.55 (d, J = 8.4 Hz, 2H), 2.93 (d, J = 18.0 Hz, 1H), 2.54 (s, 2H), 2.48-2.41 (m, 5H), 1.81-1.63 (m, 8H). ^{13}C NMR (100 MHz, $CDCl_3$): δ 161.0, 147.5, 146.0, 141.2, 129.2, 126.6, 124.7, 115.4, 114.1, 26.4, 25.4, 24.1, 24.0, 22.2, 22.0, 21.8; IR (KBr, v, cm^{-1}) 3227, 2935, 1644, 1547, 1288, 1093, 737; HRMS (ESI-TOF) calcd for $C_{19}H_{20}ClN_2O$ [M-H]$^-$ 327.1264, found 327.1252.

(6aS,6bS,12bS,14aS)-10-(Benzyloxy)-2,14a-dimethyl-3-(p-tolylamino)-5,6,6a,6b,7,8,12b,13,14,14a-decahydrophenanthro[2,1-f]isoquinolin-4(3H)-one (44)

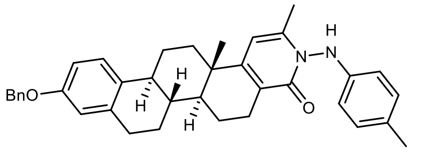

brown solid (64.3 mg, 59% yield); mp: 104-106 ºC; ^{1}H NMR (400 MHz, $CDCl_3$): δ 7.46 (s, 1H), 7.45-7.39 (m, 4H), 7.34 (d, J = 7.2 Hz, 1H), 7.26-7.19 (m, 1H), 7.04 (d, J = 8.4 Hz, 2H), 6.82 (m, 1H), 6.84-6.81 (d, J = 1.9 Hz, 1H), 6.59 (d, J = 8.0 Hz, 2H), 6.17 (s, 1H), 5.06 (s, 2H), 2.92-2.88 (m, 2H), 2.79 (d, J = 16.4 Hz, 1H), 2.52 - 2.44 (m, 2H), 2.36 (s, 3H), 2.32 (s, 1H), 2.27 (s, 3H), 2.23-2.16 (m, 3H), 1.64 (d, J = 8.4 Hz, 2H), 1.48-1.35 (m, 5H), 1.15 (s, 3H). ^{13}C NMR (100 MHz, $CDCl_3$): δ 162.2, 157.0, 144.6, 143.9, 138.1, 137.4, 133.0, 131.7, 129.9, 128.7, 128.0, 127.5, 126.3, 123.0, 114.6, 114.5, 112.6, 70.1, 43.2, 39.0, 37.3, 36.8, 30.2, 26.6, 26.5, 20.7, 19.8, 19.0; IR (KBr, v, cm^{-1}) 3240, 2925, 1649, 1510, 1236, 1025, 735; HRMS (ESI-TOF) calcd for $C_{37}H_{40}N_2O_2$ [M+H]$^+$ 545.3168, found 545.3169.

(6aS,6bS,12bS,14aS)-10-(Benzyloxy)-3-((4-chlorophenyl)amino)-2,14a-dimethyl-5,6,6a,6b,7,8,12b,13,14,14a-decahydrophenanthro[2,1-f]isoquinolin-4(3H)-one (45)

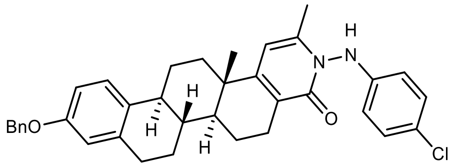

brown solid (73.5 mg, 65% yield); mp: 114-115 ºC; ^{1}H NMR (400 MHz, $CDCl_3$): δ 7.80-7.60 (m, 1H), 7.47 (d, J = 7.6 Hz, 2H), 7.43-7.39 (m, 2H), 7.36 (d, J = 7.2 Hz, 1H), 7.27 (d, J = 6.4 Hz, 1H), 7.18 (d, J = 8.0 Hz, 2H), 6.84 (d, J = 8.4 Hz, 1H), 6.78 (s, 1H), 6.60 (d, J = 8.4 Hz, 2H), 6.21 (s, 1H), 5.07

(s, 2H), 2.97-2.88 (m, 2H), 2.80 (d, J = 17.2 Hz, 1H), 2.49 (s, 2H), 2.36 (s, 1H), 2.35 (s, 3H), 2.34-2.30 (m, 1H), 2.26-2.16 (m, 2H), 1.68 (d, J = 10.0 Hz, 2H), 1.47-1.35 (m, 5H), 1.16 (s, 3H). ^{13}C NMR (100 MHz, $CDCl_3$): δ 156.4, 145.2, 143.4, 137.5, 136.8, 132.3, 128.7, 128.0, 127.4, 126.9, 126.3, 125.7, 122.7, 114.9, 114.0, 112.0, 102.8, 69.5, 42.6, 38.4, 36.8, 36.2, 29.6, 25.9, 19.2, 18.4; IR (KBr, v, cm^{-1}) 3235, 2927, 1651, 1491, 1251, 1025, 736; HRMS (ESI-TOF) calcd for $C_{36}H_{36}ClN_2O_2$ [M-H]$^-$ 563.2465, found 563.2465.

(6aS,6bS,12bS,14aS)-10-(Benzyloxy)-3-((4-bromophenyl)amino)-2,14a-dimethyl-5,6,6a,6b,7,8,12b,13,14,14a-decahydrophenanthro[2,1-f]isoquinolin-4(3H)-one (46)

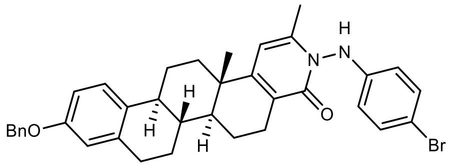

brown solid (75.6 mg, 62% yield); mp: 119-121 °C; ^{1}H NMR (400 MHz, $CDCl_3$): δ 7.45 (d, J = 7.2 Hz, 2H), 7.42-7.38 (m, 2H), 7.35-7.31 (m, 3H), 7.24 (s, 1H), 6.83 (d, J = 8.4 Hz, 1H), 6.77 (s, 1H), 6.55 (d, J = 8.8 Hz, 2H), 6.20 (s, 1H), 5.07 (s, 2H), 2.94-2.87 (m, 2H), 2.78 (d, J = 16.8 Hz, 1H), 2.56-2.44 (m, 2H), 2.34 (s, 1H), 2.34 (s, 3H), 2.33-2.30 (m, 1H), 2.24-2.14 (m, 2H), 1.67 (d, J = 10.4 Hz, 2H), 1.48-1.32 (m, 5H), 1.15 (s, 3H). ^{13}C NMR (100 MHz, $CDCl_3$): δ 162.2, 157.0, 146.3, 143.8, 138.0, 137.4, 132.9, 132.2, 128.6, 127.9, 127.5, 126.2, 123.2, 116.0, 114.6, 114.4, 112.6, 103.3, 70.1, 43.2, 39.0, 37.3, 36.8, 30.1, 26.6, 26.4, 19.8, 18.9; IR (KBr, v, cm^{-1}) 3230, 2925 , 1648, 1498, 1234, 1025, 734; HRMS (ESI-TOF) calcd for $C_{36}H_{36}BrN_2O_2$ [M-H]$^-$ 607.1960, found 607.1067.

(6aS,6bS,12bS,14aS)-10-(Benzyloxy)-2,14a-dimethyl-3-((4-(trifluoromethyl)phenyl)amino)-5,6,6a,6b,7,8,12b,13,14,14a-decahydrophenanthro[2,1-f]isoquinolin-4(3H)-one (47)

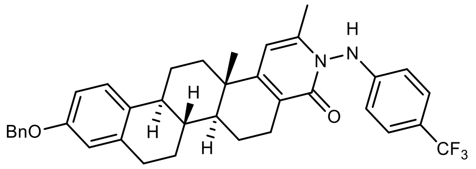

brown solid (65.9 mg, 55% yield); mp: 124-126 °C; ^{1}H NMR (400 MHz, $CDCl_3$): δ 7.76-7.73 (m, 2H), 7.62 (d, J = 8.0 Hz, 2H), 7.45 (s, 1H), 7.40 (s, 2H), 7.33 (d, J = 7.2 Hz, 2H), 7.11-7.09 (m, 1H), 6.83 (d, J = 8.8 Hz, 1H), 6.77 (d, J = 3.2 Hz, 2H), 6.24 (s, 1H), 5.07 (s, 2H), 2.94-2.89 (m, 2H), 2.81 (d, J = 16.8 Hz, 1H), 2.56-2.49 (m, 2H), 2.40 (s, 3H), 2.36 (s, 2H), 2.25-2.16 (m, 2H), 1.68 (d, J = 8.0 Hz, 2H), 1.54-1.32 (m, 5H), 1.18 (s, 3H). ^{13}C NMR (100 MHz, $CDCl_3$): δ 162.1, 157.0, 144.7, 143.9, 138.1,

137.4, 134.2, 133.0, 130.1, 129.5, 128.6, 127.9, 127.7, 127.5, 126.9, 126.5, 126.2, 124.0, 123.2, 116.7, 114.6, 112.6, 70.1, 43.2, 39.0, 37.3, 36.8, 30.2, 26.6, 26.5, 19.8, 18.9; IR (KBr, *v*, cm^{-1}) 3225, 2926, 1651, 1499, 1234, 1024, 743; HRMS (ESI-TOF) calcd for $C_{37}H_{37}F_3N_2O_2Na$ [M+Na]$^+$ 621.2705, found 621.2706.

4-(((6aS,6bS,12bS,14aS)-10-(Benzyloxy)-2,14a-dimethyl-4-oxo-5,6,6a,6b,7,8,12b,13,14,14a-decahydrophenanthro[2,1-f]isoquinolin-3(4H)-yl)amino)benzonitrile (48)

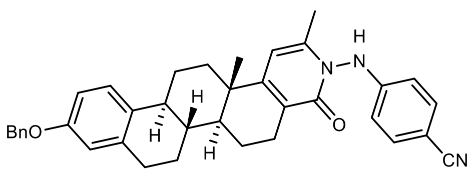

brown solid (58.9 mg, 53% yield); mp: 141-142 ºC; ^{1}H NMR (400 MHz, $CDCl_3$): δ 7.49-7.44 (m, 4H), 7.41-7.34 (m, 2H), 7.34 (d, *J* = 7.2 Hz, 1H), 7.23 (d, *J* = 11.6 Hz, 1H), 6.83 (d, *J* = 6.0 Hz, 1H), 6.76 (s, 1H), 6.64 (d, *J* = 8.4 Hz, 2H), 6.23 (s, 1H), 5.06 (s, 2H), 2.94-2.88 (m, 2H), 2.80-2.72 (m, 1H), 2.53-2.42 (m, 2H), 2.34 (s, 1H), 2.31 (s, 3H), 2.23-2.15 (m, 3H), 1.69-1.62 (m, 2H), 1.45-1.36 (m, 5H), 1.16 (d, *J* = 15.6 Hz, 3H). ^{13}C NMR (100 MHz, $CDCl_3$): δ 162.3, 157.7, 156.9, 151.2, 144.2, 137.3, 133.7, 128.6, 127.9, 127.5, 126.3, 119.4, 114.6, 113.7, 112.6, 104.3, 103.9, 70.0, 46.4, 46.0, 43.2, 38.9, 37.4, 36.6, 30.1, 26.4, 24.4, 21.3, 19.69, 18.9; IR (KBr, *v*, cm^{-1}) 3219, 2927, 2221, 1609, 1500, 1235, 1025, 735; HRMS (ESI-TOF) calcd for $C_{37}H_{36}N_3O_2$ [M-H]$^-$ 554.2808, found 554.2825.

(6aS,6bS,12bS,14aS)-10-(Benzyloxy)-2,14a-dimethyl-3-((4-nitrophenyl)amino)-5,6,6a,6b,7,8,12b,13,14,14a-decahydrophenanthro[2,1-f]isoquinolin-4(3H)-one (49)

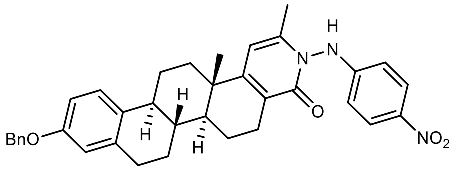

brown solid (69.1 mg, 60% yield); mp: 134-135 ºC; ^{1}H NMR (400 MHz, $CDCl_3$): δ 8.07-8.04 (m, 2H), 7.45 (d, *J* = 7.6 Hz, 2H), 7.41-7.37 (m, 2H), 7.34 (d, *J* = 7.2 Hz, 1H), 7.26-7.19 (m, 1H), 6.82 (s, 1H), 6.76 (s, 1H), 6.63 (d, *J* = 8.4 Hz, 2H), 6.25 (s, 1H), 5.06 (s, 2H), 2.94-2.88 (m, 2H), 2.79-2.74 (m, 1H), 2.49-2.44 (m, 2H), 2.34 (s, 1H), 2.31 (s, 3H), 2.24-2.17 (m, 3H), 1.70-1.63 (m, 2H), 1.40-1.34 (m, 5H), 1.16 (d, *J* = 16.4 Hz, 3H). ^{13}C NMR (100 MHz, $CDCl_3$): δ 162.3, 158.0, 157.0, 153.0, 144.1, 141.8, 138.0, 137.3, 132.8, 128.6, 127.9, 127.5, 126.3, 125.8, 123.7, 114.6, 112.6, 104.1, 70.0, 46.4, 46.0, 43.2, 38.9, 38.8, 37.4, 36.6, 30.1, 26.4, 21.44, 21.25, 19.68, 18.9; IR (KBr, *v*, cm^{-1}) 3203, 2928, 1601,

1501, 1333, 1111, 735; HRMS (ESI-TOF) calcd for $C_{36}H_{36}N_3O_4$ [M-H]$^-$ 574.2706, found 574.2712.

(6aS,6bR,10S,12aS,12bS,14aS)-2,12a,14a-Trimethyl-10-((tetrahydro-2H-pyran-2-yl)oxy)-3-(p-tolylamino)-5,6,6a,6b,7,8,8a,9,10,11,12,12a,12b,13,14,14a-hexadecahydrophenanthro[2,1-f]isoquinolin-4(3H)-one (50)

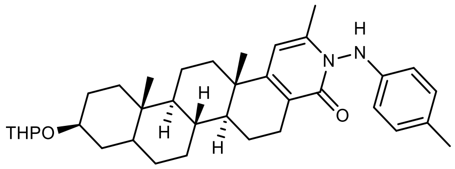

brown solid (64.8 mg, 58% yield); mp: 104-105 °C; ^{1}H NMR (400 MHz, $CDCl_3$): δ 7.03 (d, J = 8.0 Hz, 2H), 6.55 (d, J = 8.0 Hz, 2H), 6.10 (s, 1H), 3.64-3.57 (m, 1H), 2.73-2.67 (m, 1H), 2.44-2.35 (m, 1H), 2.32 (s, 3H), 2.25 (s, 3H), 2.15 (d, J = 12.0 Hz, 1H), 2.05-2.00 (m, 3H), 1.87-1.69 (m, 4H), 1.59 (d, J = 12.4 Hz, 1H), 1.52-1.21 (m, 10H), 1.20-1.11 (m, 2H), 1.09 (s, 3H), 1.03-0.85 (m, 3H), 0.84 (s, 3H), 0.77-0.72 (m, 1H). ^{13}C NMR (100 MHz, $CDCl_3$): δ 156.7, 144.6, 143.7, 131.7, 129.9, 123.0, 114.5, 103.1, 100.0, 71.3, 53.7, 44.4, 38.1, 37.2, 36.9, 36.9, 35.7, 35.2, 31.5, 31.1, 28.7, 21.0, 20.7, 19.8, 19.0, 12.4, 12.4; IR (KBr, v, cm^{-1}) 3238, 2917, 1698, 1641, 1229, 998, 769; HRMS (ESI-TOF) calcd for $C_{36}H_{49}N_2O_3$ [M-H]$^-$ 621.2692, found 621.2686.

(6aS,6bR,10S,12aS,12bS,14aS)-3-((4-Bromophenyl)amino)-2,12a,14a-trimethyl-10-((tetrahydro-2H-pyran-2-yl)oxy)-5,6,6a,6b,7,8,8a,9,10,11,12,12a,12b,13,14,14a-hexadecahydrophenanthro[2,1-f]isoquinolin-4(3H)-one (51)

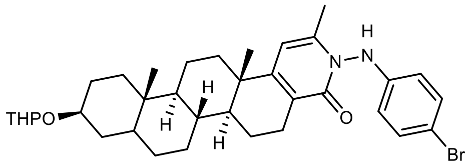

brown solid (76.1 mg, 61% yield); mp: 106-107 °C; ^{1}H NMR (400 MHz, $CDCl_3$): δ 7.43 (s, 1H), 7.32 (d, J = 8.4 Hz, 2H), 6.53 (d, J = 8.0 Hz, 2H), 6.12 (s, 1H), 3.76-3.48 (m, 1H), 2.69 (d, J = 18.4 Hz, 1H), 2.43-2.34 (m, 1H), 2.30 (s, 3H), 2.15 (d, J = 11.6 Hz, 1H), 2.03 (s, 3H), 1.90-1.51 (m, 9H), 1.47-1.24 (m, 10H), 1.24-0.87 (m, 9H), 0.85 (d, J = 6.8 Hz, 3H), 0.75 (d, J = 10.0 Hz, 1H). ^{13}C NMR (100 MHz, $CDCl_3$): δ 170.8, 162.1, 146.2, 143.5, 132.2, 123.2, 116.0, 114.5, 103.4, 73.7, 71.3, 53.6, 44.3, 38.1, 36.9, 36.8, 36.7, 35.7, 35.7, 35.1, 33.9, 31.5, 31.1, 28.7, 27.5, 21.6, 20.9, 19.7, 18.9, 12.4; IR (KBr, v, cm^{-1}) 3230, 2939, 1719, 1639, 1238, 1021, 786; HRMS (ESI-TOF) calcd for $C_{35}H_{46}FN_2O_3$ [M-H]$^-$ 621.2692, found 621.2705.

6-Methyl-4-phenyl-1-(phenylamino)pyridin-2(1H)-one (52)

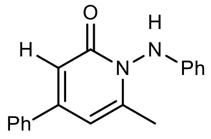

brown solid (38.7 mg, 70% yield); mp: 120-122 °C; ^{1}H NMR (400 MHz, $CDCl_3$): δ 7.84 (s, 1H), 7.66-7.60 (m, 2H), 7.47 (d, J = 6.8 Hz, 3H), 7.25-7.22 (m, 2H), 6.99-6.95 (m, 1H), 6.43 (s, 1H), 2.43 (s, 3H). ^{13}C NMR (100 MHz, $CDCl_3$): δ 162.1, 150.7, 148.2, 146.4, 137.7, 131.9, 130.3(3), 130.3(1), 130.0, 129.5, 127.2, 122.5, 117.6, 114.4, 107.9, 19.2; IR (KBr, v, cm^{-1}) 2961, 1669, 1542, 1473, 1080, 765, 668; HRMS (ESI-TOF) calcd for $C_{18}H_{15}N_2O$ [M-H]$^-$ 275.3310, found 275.1171.

6-Methyl-1-(phenylamino)-4-(o-tolyl)pyridin-2(1H)-one (53)

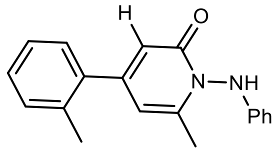

white solid (30.2 mg, 52% yield); mp: 118-119 °C; ^{1}H NMR (400 MHz, $CDCl_3$): δ 7.66 (s, 1H), 7.33-7.27 (m, 4H), 7.26 (s, 2H), 7.02-6.98 (m, 1H), 6.73 (d, J = 8.0 Hz, 2H), 6.54 (s, 1H), 6.14 (s, 1H), 2.41 (s, 3H), 2.37 (s, 3H). ^{13}C NMR (100 MHz, $CDCl_3$): δ 162.2, 153.5, 148.1, 146.5, 138.9, 134.9, 130.8, 129.5, 128.6, 126.1, 122.6, 117.0, 114.5, 108.1, 108.1, 20.4, 19.2; HRMS (ESI-TOF) calcd for $C_{19}H_{17}N_2O$ [M-H]$^-$ 289.1341, found 289.1322.

6-Methyl-1-(phenylamino)-4-(m-tolyl)pyridin-2(1H)-one (54)

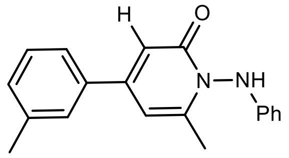

brown solid (32.5 mg, 56% yield); mp: 113-114 °C; ^{1}H NMR (400 MHz, $CDCl_3$): δ 7.68 (s, 1H), 7.42 (d, J = 8.8 Hz, 2H), 7.38-7.34 (m, 1H), 7.28 (s, 1H), 7.24 (d, J = 8.0 Hz, 2H), 7.00-6.96 (m, 1H), 6.81 (d, J = 1.6 Hz, 1H), 6.72 (d, J = 8.0 Hz, 2H), 6.42 (s, 1H), 2.43 (d, J = 1.6 Hz, 6H). ^{13}C NMR (100 MHz, $CDCl_3$ δ 162.5, 151.8, 148.7, 146.5, 138.8, 137.7, 130.4, 129.5, 129.0, 127.7, 124.1, 122.5, 114.4, 105.6, 21.6, 19.3; IR (KBr, v, cm^{-1}) 3033, 1660, 1576, 1496, 1028, 786, 692; HRMS (ESI-TOF) calcd for $C_{19}H_{17}N_2O$ [M-H]$^-$ 289.1341, found 289.1361.

6-Methyl-1-(phenylamino)-4-(p-tolyl)pyridin-2(1H)-one (55)

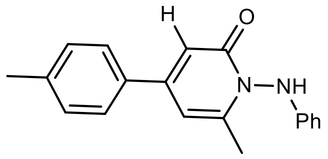

brown solid (30.8 mg, 53% yield); mp: 143-144 ºC; ^{1}H NMR (400 MHz, $CDCl_3$): *δ* 7.69 (s, 1H), 7.53 (d, *J* = 8.0 Hz, 2H), 7.28 (d, *J* = 8.0 Hz, 2H), 7.23 (d, *J* = 8.0 Hz, 2H), 6.99-6.96 (m, 1H), 6.80 (s, 1H), 6.72 (d, *J* = 8.0 Hz, 2H), 6.42 (s, 1H), 2.42 (s, 6H). ^{13}C NMR (100 MHz, $CDCl_3$): *δ* 162.4, 151.5, 148.6, 144.0, 137.8, 132.1, 132.1, 130.0, 129.6, 129.1, 127.0, 114.7, 114.0, 105.5, 20.7, 19.4; IR (KBr, *v*, cm^{-1}) 3032, 1654, 1541, 1496, 1029, 809, 692; HRMS (ESI-TOF) calcd for $C_{19}H_{17}N_2O$ $[M-H]^-$ 289.1341, found 289.1327.

4-(2-Chlorophenyl)-6-methyl-1-(phenylamino)pyridin-2(1H)-one (56)

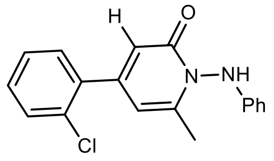

brown solid (37.1 mg, 61% yield); mp: 181-182 ºC; ^{1}H NMR (400 MHz, $CDCl_3$): *δ* 7.73 (s, 1H), 7.50-7.48 (m, 1H), 7.38-7.33 (m, 3H), 7.28-7.26 (m, 1H), 7.24 (s, 1H), 7.00-6.97 (m 1H), 6.73 (d, *J* = 8.0 Hz, 2H), 6.64 (d, *J* = 1.6 Hz, 1H), 6.26 (s, 1H), 2.41 (s, 3H). ^{13}C NMR (100 MHz, $CDCl_3$): *δ* 162.5, 151.6, 148.9, 146.5, 137.7, 129.5, 129.1, 127.0, 122.4, 114.3, 114.1, 105.6, 19.3; IR (KBr, *v*, cm^{-1}) 3056, 1663, 1541, 1496, 1038, 758, 693; HRMS (ESI-TOF) calcd for $C_{18}H_{14}ClN_2O$ $[M-H]^-$ 309.0795, found 309.0775.

4-(3-Chlorophenyl)-6-methyl-1-(phenylamino)pyridin-2(1H)-one (57)

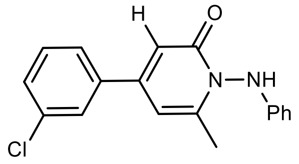

white solid (34.2 mg, 55% yield); mp: 149-150 ºC; ^{1}H NMR (400 MHz, $CDCl_3$): *δ* 7.59 (s, 2H), 7.49 (d, *J* = 6.8 Hz, 1H), 7.41 (d, *J* = 6.4 Hz, 2H), 7.27 (s, 1H), 7.25 (d, *J* = 7.6 Hz, 1H), 7.01-6.97 (m, 1H), 6.77 (s, 1H), 6.71 (d, *J* = 8.0 Hz, 2H), 6.37 (s, 1H), 2.44 (s, 3H). ^{13}C NMR (100 MHz, $CDCl_3$): *δ* 162.1, 150.2, 149.0, 146.2, 139.6, 135.0, 130.3, 129.5, 129.5, 127.1, 125.1, 122.7, 114.4, 105.1, 19.3; IR (KBr,

v, cm^{-1}) 2956, 1647, 1559, 1457, 1051, 757, 703; HRMS (ESI-TOF) calcd for $C_{18}H_{14}ClN_2O$ $[M-H]^-$ 309.0795, found 309.0792.

4-(4-Chlorophenyl)-6-methyl-1-(phenylamino)pyridin-2(1H)-one (58)

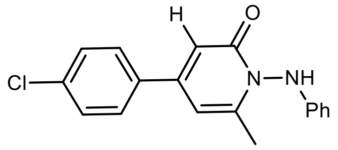

brown solid (32.3 mg, 52% yield); mp: 99-100 °C; ^{1}H NMR (400 MHz, $CDCl_3$): δ 7.55 (d, J = 8.0 Hz, 3H), 7.45 (d, J = 8.4 Hz, 2H), 7.28 (s, 1H), 7.24 (s, 1H), 7.02-6.98 (m, 1H), 6.79 (s, 1H), 6.72 (d, J = 8.0 Hz, 2H), 6.39 (s, 1H), 2.44 (s, 3H). ^{13}C NMR (100 MHz, $CDCl_3$): δ 162.2, 150.3, 148.9, 146.2, 136.1, 135.8, 129.5, 129.5, 128.2, 122.7, 114.5, 114.0, 105.1, 19.3; IR (KBr, *v*, cm^{-1}) 3055, 1662, 1585, 1444, 1092, 754, 693; HRMS (ESI-TOF) calcd for $C_{18}H_{14}ClN_2O$ $[M-H]^-$ 309.0795, found 309.0793.

1-((4-Chlorophenyl)amino)-4-(4-fluorophenyl)-6-methylpyridin-2(1H)-one (59)

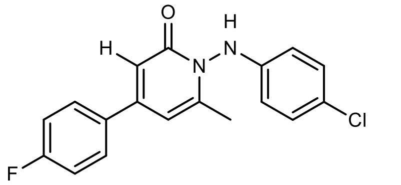

yellow solid (45.4 mg, 69% yield); mp: 176-177 °C; ^{1}H NMR (400 MHz, $CDCl_3$): δ 7.90-7.77 (m, 1H), 7.61-7.57 (m, 2H), 7.21-7.11 (m, 4H), 6.75 (d, J = 1.2 Hz, 1H), 6.62-659 (m, 2H), 6.38 (s, 1H), 2.40 (s, 3H). ^{13}C NMR (100 MHz, $CDCl_3$): δ 163.8 ($^1J_{CF}$ = 248.7 Hz), 162.4, 150.8, 148.9, 145.2, 133.7, 129.4, 128.9 ($^2J_{CF}$ = 8.5 Hz), 116.3, 115.6 ($^3J_{CF}$ = 2.9 Hz), 114.1, 105.6, 19.3; IR (KBr, *v*, cm^{-1}) 3034, 1660, 1577, 1490, 1092, 738, 591; HRMS (ESI-TOF) calcd for $C_{18}H_{1}ClFN_2O$ $[M-H]^-$ 327.0700, found 327.0703.

1-((4-Chlorophenyl)amino)-6-methyl-4-(4-(trifluoromethyl)phenyl)pyridin-2(1H)-one (60)

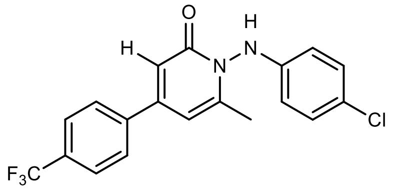

white solid (51.5 mg, 68% yield); mp: 154-155 °C; ^{1}H NMR (400 MHz, $CDCl_3$): δ 8.05 (s, 1H), 7.80-7.74 (m, 4H), 7.20 (d, J = 8.8 Hz, 2H), 6.87 (d, J = 2.0 Hz, 1H), 6.65 (d, J = 8.8 Hz, 2H), 6.46 (s, 1H), 2.47 (s, 3H). ^{13}C NMR (100 MHz, $CDCl_3$): δ 162.3, 150.5, 149.6, 145.0, 141.2, 131.8, 131.4, 129.4,

127.4, 126.1 ($^{1}J_{CF}$ = 3.9 Hz), 115.5, 115.0, 105.6, 19.4; IR (KBr, *v*, cm^{-1}) 3031, 1667, 1541, 1411, 1017, 741, 504; HRMS (ESI-TOF) calcd for $C_{19}H_{13}ClF_3N_2O$ [M-H]$^-$ 377.0669, found 377.0669.

1-((4-Chlorophenyl)amino)-6-methyl-4-(naphthalen-2-yl)pyridin-2(1H)-one (61)

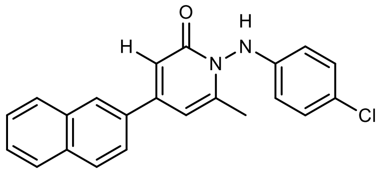

brown solid (46.9 mg, 65% yield); mp: 179-181 °C; ^{1}H NMR (400 MHz, $CDCl_3$): δ 8.10 (s, 1H), 7.96-7.87 (m, 3H), 7.71 (d, *J* = 8.4 Hz, 1H), 7.57-7.55 (m, 2H), 7.20 (d, *J* = 7.6 Hz, 2H), 6.94 (s, 1H), 6.66 (d, *J* = 8.0 Hz, 2H), 6.57 (s, 1H), 2.45 (s, 3H). ^{13}C NMR (100 MHz, $CDCl_3$): δ 151.8, 148.7, 145.3, 134.9, 133.9, 133.4, 129.5, 129.0, 128.7, 127.9, 127.2, 126.9, 126.7, 124.3, 115.8, 19.4; IR (KBr, *v*, cm^{-1}) 3055, 1683, 1540, 1472, 1092, 744, 648; HRMS (ESI-TOF) calcd for $C_{22}H_{16}ClN_2O$ [M-H]$^-$ 359.0951, found 359.0951.

1-((4-Chlorophenyl)amino)-6-methyl-4-(thiophen-2-yl)pyridin-2(1H)-one (62)

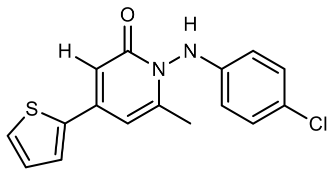

brown solid (32.3 mg, 51% yield); mp: 101-102 °C; ^{1}H NMR (400 MHz, $CDCl_3$): δ 7.46 (d, *J* = 4.8 Hz, 2H), 7.19 (d, *J* = 8.0 Hz, 2H), 7.15-7.13 (m, 1H), 6.83 (s, 1H), 6.63 (d, *J* = 8.4 Hz, 2H), 6.43 (s, 1H), 2.39 (s, 3H). ^{13}C NMR (100 MHz, $CDCl_3$): δ 148.6, 145.0, 144.5, 140.3, 129.4, 128.5, 128.3, 127.5, 126.5, 115.64, 111., 104.4, 19.1; IR (KBr, *v*, cm^{-1}) 3102, 1655, 1578, 1490, 1092, 706, 674; HRMS (ESI-TOF) calcd for $C_{16}H_{12}ClN_2OS$ [M-H]$^-$ 315.0359, found 315.0380.

1-((4-Chlorophenyl)amino)-4-cyclopropyl-6-methylpyridin-2(1H)-one (63)

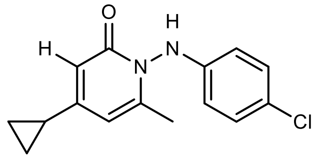

white solid (43.4 mg, 79% yield); mp: 126-128 °C; ^{1}H NMR (400 MHz, $CDCl_3$): δ 7.98 (s, 1H), 7.09 (d, *J* = 8.0 Hz, 2H), 6.49 (d, *J* = 8.0 Hz, 2H), 6.26 (d, *J* = 1.2 Hz, 1H), 5.82 (s, 1H), 2.27 (s, 3H), 1.73-1.68 (m, 1H), 1.07-1.03 (m, 2H), 0.79 (d, *J* = 4.8 Hz, 2H). ^{13}C NMR (100 MHz, $CDCl_3$): δ 162.2,

157.9, 148.1, 145.5, 129.1, 126.6, 115.1, 112.0, 104.9, 19.0, 15.3, 10.2, 10.0; IR (KBr, *v*, cm^{-1}) 3012, 1660, 1578, 1491, 1052, 736, 647; HRMS (ESI-TOF) calcd for $C_{15}H_{14}ClN_2O$ [M-H]$^-$ 273.0795, found 273.0806.

1-((4-Chlorophenyl)amino)-4-cyclohexyl-6-methylpyridin-2(1H)-one (64)

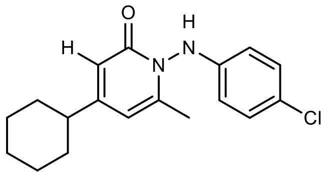

brown solid (38.7 mg, 61% yield); mp: 121-123 °C; ^{1}H NMR (400 MHz, $CDCl_3$): δ 7.73 (s, 1H), 7.13 (d, J = 8.8 Hz, 2H), 6.54 (d, J = 8.8 Hz, 2H), 6.40 (d, J = 0.8 Hz, 1H), 6.03 (s, 1H), 2.34 (d, J = 11.2 Hz, 1H), 2.30 (s, 3H), 1.87-1.85 (m, 4H), 1.77 (s, 1H), 1.42-1.24 (m, 5H). ^{13}C NMR (100 MHz, $CDCl_3$): δ 160.5, 147.7, 145.4, 129.3, 127.1, 115.5, 113.9, 106.8, 44.1, 32.8, 26.5, 26.0, 19.0; IR (KBr, *v*, cm^{-1}) 2928, 1663, 1542, 1490, 1008, 738, 645; HRMS (ESI-TOF) calcd for $C_{18}H_{20}ClN_2O$ [M-H]$^-$ 315.1264, found 315.1275.

4-Benzyl-1-((4-chlorophenyl)amino)-6-methylpyridin-2(1H)-one (65)

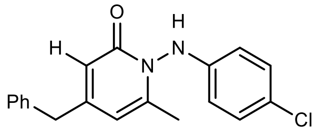

brown solid (39.0 mg, 60% yield); mp: 135-136 °C; ^{1}H NMR (400 MHz, $CDCl_3$): δ 7.88 (s, 1H), 7.37-7.33 (m, 2H), 7.29 (d, J = 7.2 Hz, 1H), 7.23 (d, J = 7.6 Hz, 2H), 7.15-7.08 (m, 2H), 6.52 (d, J = 8.4 Hz, 2H), 6.41 (s, 1H), 5.98 (s, 1H), 3.79 (s, 2H), 2.26 (s, 3H). ^{13}C NMR (100 MHz, $CDCl_3$): δ 162.4, 154.0, 148.4, 145.3, 138.0, 129.2, 128.9, 126.9, 116.4, 115.3, 107.8, 41.7, 18.9; IR (KBr, *v*, cm^{-1}) 3028, 1660, 1581, 1429, 1008, 736, 644; HRMS (ESI-TOF) calcd for $C_{19}H_{16}ClN_2O$ [M-H]$^-$ 323.0951, found 323.0951.

4-(tert-Butyl)-1-((4-chlorophenyl)amino)-6-methylpyridin-2(1H)-one (66)

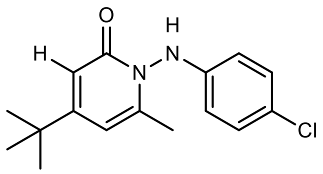

white solid (49.4 mg, 85% yield); mp: 100-101 °C; ^{1}H NMR (400 MHz, $CDCl_3$): δ 8.40 (s, 1H), 7.02

(d, J = 8.8 Hz, 2H), 6.54 (d, J = 2.4 Hz, 1H), 6.44 (d, J = 8.8 Hz, 2H), 6.17 (s, 1H), 2.29 (s, 3H), 1.25 (s, 9H). ^{13}C NMR (100 MHz, $CDCl_3$): δ 163.6, 162.6, 147.6, 145.4, 129.1, 115.2, 113.0, 105.4, 35.1, 29.9, 19.1; IR (KBr, v, cm^{-1}) 2967, 1663, 1578, 1508, 1092, 736, 646; HRMS (ESI-TOF) calcd for $C_{16}H_{18}ClN_2O$ $[M-H]^-$ 289.1108, found 289.1125.

1-((4-Chlorophenyl)amino)-4-heptyl-6-methylpyridin-2(1H)-one (67)

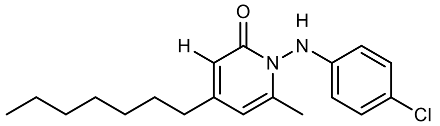

white solid (43.3 mg, 65% yield); mp: 133-134 °C; ^{1}H NMR (400 MHz, $CDCl_3$): δ 7.82 (s, 1H), 7.12 (d, J = 8.4 Hz, 2H), 6.53 (d, J = 8.8 Hz, 2H), 6.39 (s, 1H), 6.00 (s, 1H), 2.47-2.41 (m, 2H), 2.29 (s, 3H), 1.65-1.56 (m, 2H), 1.43-1.25 (m, 10H), 0.97-0.87 (m, 3H). ^{13}C NMR (100 MHz, $CDCl_3$): δ 156.0, 147.8, 145.4, 129.3, 127.0, 115.5, 107.9, 35.6, 31.8, 29.4, 29.1, 22.7, 19.0, 14.2; IR (KBr, v, cm^{-1}) 2952, 1658, 1556, 1469, 1078, 756, 644; HRMS (ESI-TOF) calcd for $C_{19}H_{24}ClN_2O$ $[M-H]^-$ 331.1577, found 331.1586.

(E)-1-((4-Chlorophenyl)amino)-6-methyl-4-styrylpyridin-2(1H)-one (68)

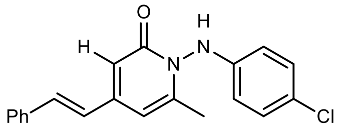

white solid (37.1 mg, 55% yield); mp: 147-148 °C; ^{1}H NMR (400 MHz, $CDCl_3$): δ 7.54 (d, J = 8.0 Hz, 2H), 7.43-7.34 (m, 3H), 7.23 (s, 1H), 7.18 (d, J = 6.0 Hz, 2H), 6.89 (d, J = 16.4 Hz, 1H), 6.62 (d, J = 8.8 Hz, 3H), 6.40 (s, 1H), 2.38 (s, 3H). ^{13}C NMR (100 MHz, $CDCl_3$): δ 147.9, 145.3, 136.0, 134.7, 129.4, 129.2, 129.0, 127.3, 125.7, 115.6, 103.5, 19.2; IR (KBr, v, cm^{-1}) 3031, 1683, 1521, 1490, 1091, 741, 642; HRMS (ESI-TOF) calcd for $C_{20}H_{16}ClN_2O$ $[M-H]^-$ 335.0951, found 335.0951.

1-((4-chlorophenyl)amino)-4,6-diphenylpyridin-2(1H)-one (69)

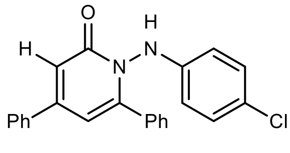

white solid (50.7 mg, 68% yield); mp: 119-121 °C; ^{1}H NMR (400 MHz, $CDCl_3$): δ 7.68-7.64 (m, 2H), 7.53-7.48 (m, 5H), 7.43-7.32 (m, 4H), 7.12 (d, J = 8.8 Hz, 2H), 6.95 (d, J = 1.6 Hz, 1H), 6.61 (s, 1H), 6.58 (s, 2H). ^{13}C NMR (100 MHz, $CDCl_3$): δ 151.8, 150.8, 145.3, 137.3, 133.8, 129.9, 129.5, 129.2,

129.2, 129.0, 128.1, 127.4, 127.0, 116.2, 115.6, 107.6; IR (KBr, ν, cm^{-1}) 3230, 1653, 1570, 1490, 913, 743; HRMS (ESI-TOF) calcd for $C_{23}H_{17}ClN_2ONa$ $[M+Na]^+$ 395.0927, found 395.0914.

1-((4-chlorophenyl)amino)-6-pentyl-4-phenylpyridin-2(1H)-one (70)

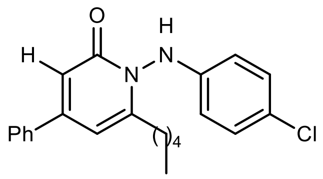

white solid (53.6 mg, 73% yield); mp: 137-139 °C; ^{1}H NMR (400 MHz, $CDCl_3$): δ 7.62 (d, J = 8.0 Hz, 2H), 7.53-7.45 (m, 3H), 7.18 (d, J = 8.0 Hz, 2H), 6.80 (s, 1H), 6.62 (d, J = 8.0 Hz, 2H), 6.41 (s, 1H), 2.88-2.78 (m, 1H), 2.62-2.48 (m, 1H), 1.74-1.64 (m, 2H), 1.35-1.31 (m, 4H), 0.90-0.87 (m, 3H). ^{13}C NMR (100 MHz, $CDCl_3$): δ 152.7, 151.9, 145.5, 137.8, 129.7, 129.4, 129.1, 127.0, 115.6, 114.2, 104.9, 32.2, 31.5, 28.0, 22.5, 14.0; IR (KBr, v, cm^{-1}) 3222, 2929, 1655, 1490, 1276, 913, 742; HRMS (ESI-TOF) calcd for $C_{22}H_{24}ClN_2O$ $[M+H]^+$ 367.1577, found 367.1564.

6-Methyl-4-phenyl-1-(o-tolylamino)pyridin-2(1H)-one (71)

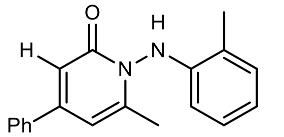

brown solid (26.1 mg, 45% yield); mp: 85-86 °C; ^{1}H NMR (400 MHz, $CDCl_3$): δ 7.68 (d, J = 6.4 Hz, 2H), 7.52 (d, J = 7.2 Hz, 3H), 7.37 (s, 1H), 7.23 (d, J = 7.2 Hz, 1H), 7.13-7.10 (m, 1H), 7.00-6.96 (m, 1H), 6.87 (d, J = 1.6 Hz, 1H), 6.48 (s, 1H), 6.34 (d, J = 8.0 Hz, 1H), 2.46 (s, 6H). ^{13}C NMR (100 MHz, $CDCl_3$): δ 151.6, 148.5, 144.0, 137.7, 133.7, 130.9, 129.6, 129.1, 127.0, 125.5, 122.5, 113.9, 112.1, 105.7, 19.2, 17.2; IR (KBr, v, cm^{-1}) 2932 ,1684, 1565, 1456, 1098, 765, 689; HRMS (ESI-TOF) calcd for $C_{19}H_{17}N_2O$ $[M-H]^-$ 289.1341, found 289.1351.

6-Methyl-4-phenyl-1-(m-tolylamino)pyridin-2(1H)-one (72)

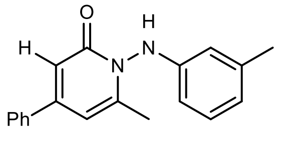

brown solid (29.0 mg, 50% yield); mp: 121-122 °C; ^{1}H NMR (400 MHz, $CDCl_3$): δ 7.73-7.56 (m, 3H), 7.47 (d, J = 6.8 Hz, 3H), 7.15-7.11 (m, 1H), 6.85-6.75 (m, 2H), 6.55 (s, 1H), 6.49 (d, J = 8.0 Hz, 1H), 6.43 (s, 1H), 2.44 (s, 3H), 2.28 (s, 3H). ^{13}C NMR (100 MHz, $CDCl_3$): δ 162.5, 151.6, 148.8, 146.4,

139.4, 137.7, 129.6, 129.3, 129.0, 126.9, 123.3, 115.0, 114.0, 111.3, 105.5, 21.6, 19.3; IR (KBr, *v*, cm^{-1}) 2922, 1659, 1574, 1488, 1056, 766, 695; HRMS (ESI-TOF) calcd for $C_{19}H_{17}N_2O$ $[M-H]^-$ 289.1341, found 289.1356.

6-Methyl-4-phenyl-1-(p-tolylamino)-5,6-dihydropyridin-2(1H)-one (73)

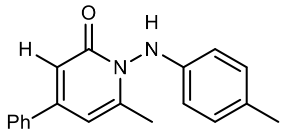

white solid (43.6 mg, 75% yield); mp: 131-132 °C; ^1H NMR (400 MHz, $CDCl_3$): δ 7.65-7.59 (m, 2H), 7.56 (s, 1H), 7.47 (d, J = 6.8 Hz, 3H), 7.06 (d, J = 7.6 Hz, 2H), 6.81 (d, J = 1.6 Hz, 1H), 6.64 (d, J = 8 Hz, 2H), 6.41 (s, 1H), 2.43 (s, 3H), 2.28 (s, 3H). ^{13}C NMR (100 MHz, $CDCl_3$): δ 162.5, 151.5, 148.6, 146.6, 139.8, 129.8, 129.5, 126.8, 114.4, 105.5, 21.4, 19.3; IR (KBr, *v*, cm^{-1}) 2923, 1654, 1570, 1456, 1114, 765, 696; HRMS (ESI-TOF) calcd for $C_{19}H_{17}N_2O$ $[M-H]^-$ 289.1341, found 289.1350.

1-((4-Fluorophenyl)amino)-6-methyl-4-phenylpyridin-2(1H)-one (74)

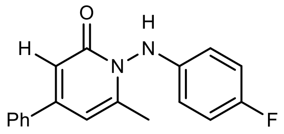

white solid (50.0 mg, 85% yield); mp: 122-124 °C; ^1H NMR (400 MHz, $CDCl_3$): δ 7.64-7.59 (m, 2H), 7.47 (d, J = 6.8 Hz, 3H), 6.96-6.91 (m, 2H), 6.82 (s, 1H), 6.69-6.66 (m, 2H), 6.43 (s, 1H), 2.42 (s, 3H). ^{13}C NMR (100 MHz, $CDCl_3$): δ 161.2 ($^1J_{CF}$ = 250.6 Hz), 151.8, 148.6, 137.7, 129.7, 129.2, 127.0, 116.2 ($^2J_{CF}$ = 6.1 Hz), 116.0, 114.1, 105.8, 19.3; HRMS (ESI-TOF) calcd for $C_{18}H_{14}FN_2O$ $[M-H]^-$ 293.1090, found 293.1099.

1-((2-Chlorophenyl)amino)-6-methyl-4-phenylpyridin-2(1H)-one (75)

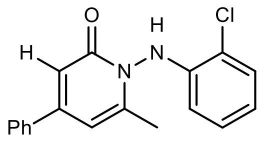

white solid (42.9 mg, 69% yield); mp: 131-132 °C; ^1H NMR (400 MHz, $CDCl_3$): δ 7.65-7.58 (m, 2H), 7.53 (s, 1H), 7.47 (d, J = 6.4 Hz, 3H), 7.37 (d, J = 8.0 Hz, 1H), 7.13-7.10 (m, 1H), 6.94-6.90 (m, 1H), 6.80 (d, J = 1.6 Hz, 1H), 6.48-6.35 (m, 2H), 2.44 (s, 3H). ^{13}C NMR (100 MHz, $CDCl_3$): δ 162.0, 151.8, 148.8 142.7, 137.6, 130.0, 129.7, 129.1, 127.9, 126.9, 122.8, 121.3, 114.4, 113.2, 105.5, 19.3; IR (KBr, *v*, cm^{-1}) 3081, 1654, 1593, 1507, 1039, 768, 637; HRMS (ESI-TOF) calcd for $C_{18}H_{14}ClN_2O$ $[M-H]^-$

309.0795, found 309.0806.

1-((3-Chlorophenyl)amino)-6-methyl-4-phenylpyridin-2(1H)-one (76)

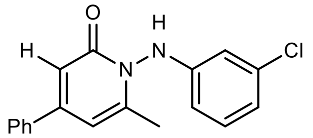

white solid (52.8 mg, 85% yield); mp: 102-104 ºC; ^{1}H NMR (400 MHz, $CDCl_3$): δ 8.12 (s, 1H), 7.66-7.59 (m, 2H), 7.47 (d, J = 6.8 Hz, 3H), 7.13-7.09 (m, 1H), 6.91 (d, J = 8.0 Hz, 1H), 6.83 (d, J = 1.2 Hz, 1H), 6.64 (s, 1H), 6.57 (d, J = 8.0 Hz, 1H), 6.44 (s, 1H), 2.41 (s, 3H). ^{13}C NMR (100 MHz, $CDCl_3$): δ 162.3, 151.9, 147.8, 137.5, 135.3, 130.5, 129.6, 129.1, 126.9, 122.5, 114.3, 114.2, 112.6, 105.8, 19.2; IR (KBr, v, cm^{-1}) 3031, 1699, 1570, 1472, 1072, 764, 669; HRMS (ESI-TOF) calcd for $C_{18}H_{14}ClN_2O$ $[M-H]^-$ 309.0795, found 309.0809.

1-((4-Chlorophenyl)amino)-6-methyl-4-phenylpyridin-2(1H)-one (77)

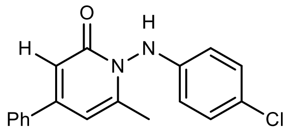

white solid (57.2 mg, 92% yield); mp: 147-148 ºC; ^{1}H NMR (400 MHz, $CDCl_3$): δ 8.19 (s, 1H), 7.63-7.60 (m, 2H), 7.4 (m, 3H), 7.13 (d, J = 8.4 Hz, 2H), 6.82 (s, 1H), 6.57-6.61 (m, 2H), 6.43 (s, 1H), 2.41 (s, 3H). ^{13}C NMR (100 MHz, $CDCl_3$): δ 162.5, 152.0, 148.8, 148.0, 137.6, 135.3, 130.6, 129.8, 129.2, 127.0, 122.4, 114.3, 112.5, 106.0, 19.4; IR (KBr, v, cm^{-1}) 3032, 1662, 1576, 1490, 1092, 765, 696; HRMS (ESI-TOF) calcd for $C_{18}H_{14}ClN_2O$ $[M-H]^-$ 309.0795, found 309.0811.

1-((4-Bromophenyl)amino)-6-methyl-4-phenylpyridin-2(1H)-one (78)

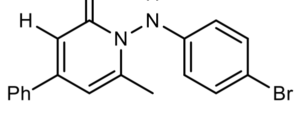

brown solid (59.0 mg, 83% yield); mp: 146-148 ºC; ^{1}H NMR (400 MHz, $CDCl_3$): δ 7.73 (s, 1H), 7.63-7.59 (m, 2H), 7.47 (d, J = 6.4 Hz, 3H), 7.33 (d, J = 8.8 Hz, 2H), 6.80 (s, 1H), 6.58 (d, J = 8.4 Hz, 2H), 6.43 (s, 1H), 2.41 (s, 3H). ^{13}C NMR (100 MHz, $CDCl_3$): δ 162.3, 151.9, 148.6, 145.6, 137.5, 132.3, 129.7, 129.1, 126.9, 116.0, 114.7, 114.1, 105.8, 19.2; IR (KBr, v, cm^{-1}) 2988, 1660, 1574, 1487, 1072, 765, 699; HRMS (ESI-TOF) calcd for $C_{18}H_{14}BrN_2O$ $[M-H]^-$ 353.0290, found 353.0295.

6-Methyl-4-phenyl-1-((4-(trifluoromethyl)phenyl)amino)pyridin-2(1H)-one (79)

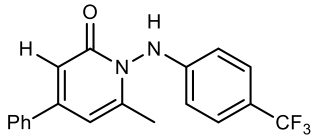

brown solid (41.3 mg, 60% yield); mp: 123-125 °C; ^{1}H NMR (400 MHz, $CDCl_3$): δ 8.70 (m, 1H), 7.67-7.60 (m, 2H), 7.48 (d, J = 6.4 Hz, 3H), 7.38 (d, J = 8.4 Hz, 2H), 6.86 (s, 1H), 6.67 (d, J = 8.4 Hz, 2H), 6.49 (s, 1H), 2.42 (s, 3H). ^{13}C NMR (100 MHz, $CDCl_3$): δ 162.8, 152.3, 149.8, 149.5, 137.5, 129.9, 129.2, 127.0, 126.7 ($^1J_{CF}$ = 4 Hz), 114.5, 113.3, 106.2, 19.3; IR (KBr, v, cm^{-1}) 3031, 1663, 1576, 1420, 1010, 766, 697; HRMS (ESI-TOF) calcd for $C_{19}H_{14}F_3N_2O$ [M-H]$^-$ 343.1058, found 343.1076.

4-((6-Methyl-2-oxo-4-phenylpyridin-1(2H)-yl)amino)benzonitrile (80)

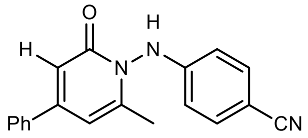

brown solid (40.4 mg, 67% yield); mp: 128-129 °C; ^{1}H NMR (400 MHz, $CDCl_3$): δ 8.74 (s, 1H), 7.63-7.59 (m, 2H), 7.48 (d, J = 4.8 Hz, 3H), 7.41 (d, J = 8.4 Hz, 2H), 6.82 (s, 1H), 6.64 (d, J = 8.4 Hz, 2H), 6.48 (s, 1H), 2.38 (s, 3H). ^{13}C NMR (100 MHz, $CDCl_3$): δ 162.6, 152.6, 150.6, 149.2, 137.2, 133.6, 129.9, 129.2, 126.9, 119.3, 114.4, 113.6, 106.4, 104.2, 19.22; IR (KBr, v, cm^{-1}) 3064, 1662, 1575, 1457, 1072, 766, 697; HRMS (ESI-TOF) calcd for $C_{19}H_{14}N_3O$ [M-H]$^-$ 300.1137, found 300.1150.

6-Methyl-1-((4-nitrophenyl)amino)-4-phenylpyridin-2(1H)-one (81)

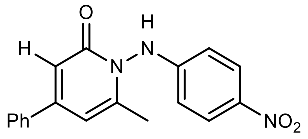

brown solid (45.0 mg, 70% yield); mp: 87-88 °C; ^{1}H NMR (400 MHz, $CDCl_3$): δ 8.79 (s, 1H), 8.02 (d, J = 8.8 Hz, 2H), 7.64-7.61 (m, 2H), 7.51-7.48 (m, 3H), 6.84 (s, 1H), 6.65 (d, J = 8.8 Hz, 2H), 6.51 (s, 1H), 2.40 (s, 3H). ^{13}C NMR (100 MHz, $CDCl_3$): δ 162.6, 152.8, 152.5, 149.1, 142.0, 137.2, 130.1, 129.3, 127.0, 125.8, 114.5, 112.9, 106.7, 19.3; IR (KBr, v, cm^{-1}) 3057, 1654, 1595, 1490, 1110, 751, 652; HRMS (ESI-TOF) calcd for $C_{18}H_{14}N_3O_3$ [M-H]$^-$ 320.1035, found 320.1045.

6-Methyl-1-(naphthalen-2-ylamino)-4-phenylpyridin-2(1H)-one (82)

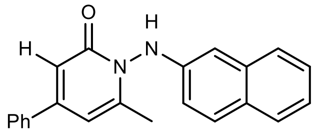

brown solid (43.1 mg, 66% yield); mp: 129-130 ºC; ^{1}H NMR (400 MHz, $CDCl_3$): δ 7.91 (s, 1H), 7.73 (d, J = 8.0 Hz, 1H), 7.70-7.65 (m, 3H), 7.60 (d, J = 8.0 Hz, 1H), 7.41-7.37 (d, J = 7.2 Hz, 3H), 7.39 (m, 1H), 7.35-7.31 (m, 1H), 7.13-7.08 (m, 1H), 6.88 (s, 1H), 6.79 (s, 1H), 6.49 (s, 1H), 2.47 (s, 3H). ^{13}C NMR (100 MHz, $CDCl_3$): δ 162.3, 151.7, 148.7, 144.1, 137.7, 134.2, 130.1, 129.6, 129., 127.73, 126.93, 126.56, 124.09, 116.75, 114.13, 108.39, 105.68, 19.24; IR (KBr, v, cm^{-1}) 3055, 1653, 1558, 1457, 1067, 765, 669; HRMS (ESI-TOF) calcd for $C_{22}H_{17}N_2O$ [M-H]$^-$ 325.1341, found 325.1347.

(1S,2R,4S)-1,7,7-trimethylbicyclo[2.2.1]heptan-2-yl 4-((6-methyl-2-oxo-4-phenylpyridin-1(2H)-yl)amino)benzoate (83)

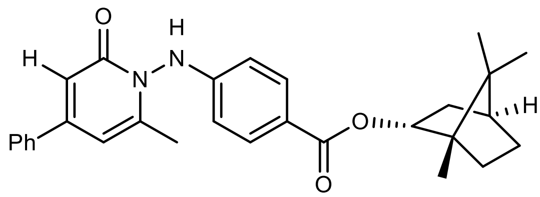

yellow solid (59.4 mg, 65% yield); mp: 132-133 ºC; ^{1}H NMR (400 MHz, $CDCl_3$): δ 7.96 (d, J = 8.4 Hz, 3H), 7.64-7.59 (m, 2H), 7.48 (d, J = 6.4 Hz, 3H), 6.82 (s, 1H), 6.72 (d, J = 8.4 Hz, 2H), 6.45 (s, 1H), 5.07 (d, J = 9.6 Hz, 1H), 2.49-2.43 (m, 1H), 2.42 (s, 3H), 2.12-2.05 (m, 1H), 1.82-1.75 (m, 1H), 1.73-1.70 (m, 1H), 1.40-1.34 (m, 1H), 1.29 (d, J = 12.4 Hz, 1H), 1.09 (d, J = 15.6 Hz, 1H), 0.95 (s, 3H), 0.89 (d, J = 7.2 Hz, 6H). ^{13}C NMR (100 MHz, $CDCl_3$): δ 166.5, 152.1, 150.7, 148.9, 137.5, 131.4, 129.7, 129.1, 126.9, 114.4, 113.3, 105.9, 80.2, 49.1, 47.9, 45.1, 28.2, 27.5, 19.8, 19.3, 19.0, 13.7; IR (KBr, v, cm^{-1}) 2954, 1662, 1576, 1455, 1016, 766, 653; HRMS (ESI-TOF) calcd for $C_{29}H_{31}N_2O_3$ [M-H]$^-$ 455.2335, found 455.2364.

(1R,2R,5S)-2-Isopropyl-5-methylcyclohexyl 4-((6-methyl-2-oxo-4-phenylpyridin-1(2H)-yl)amino)benzoate (84)

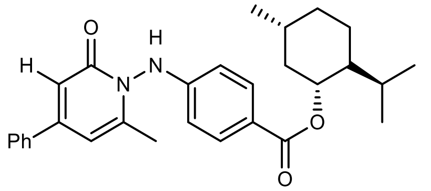

brown solid (51.4 mg, 56% yield); mp: 118-119 ºC; ^{1}H NMR (400 MHz, $CDCl_3$): δ 7.94 (d, J = 8.4 Hz, 3H), 7.64-7.59 (m, 2H), 7.50-7.45 (m, 3H), 6.81 (s, 1H), 6.71 (d, J = 8.4 Hz, 2H), 6.45 (s, 1H), 4.91-4.85 (m, 1H), 2.41 (s, 3H), 2.10 (d, J = 12.0 Hz, 1H), 1.95-1.90 (m, 1H), 1.71 (d, J = 11.6 Hz, 2H), 1.54-1.48 (m, 2H), 1.16-1.02 (m, 2H), 0.93-0.88 (m, 6H), 0.77 (d, J = 6.8 Hz, 3H). ^{13}C NMR

(100 MHz, $CDCl_3$): δ 165.8, 162.6, 152.2, 150.7, 149.0, 137.6, 131.5, 129.8, 129.2, 127.0, 114.4, 113.3, 106.0, 74.6, 47.5, 41.2, 34.5, 31.6, 26.7, 23.8, 22.2, 20.9, 19.3, 16.7; IR (KBr, v, cm^{-1}) 2955, 1663, 1541, 1457, 1109, 767, 697; HRMS (ESI-TOF) calcd for $C_{29}H_{33}N_2O_3$ $[M-H]^-$ 457.2491, found 457.2495.

(3s,5s,7s)-Adamantan-1-yl 4-((6-methyl-2-oxo-4-phenylpyridin-1(2H)-yl)amino)benzoate (85)

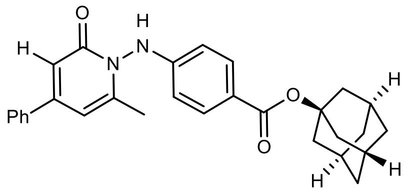

brown solid (53.6 mg, 59% yield); mp: 153-155 °C; ^1H NMR (400 MHz, $CDCl_3$): δ 7.91 (s, 1H), 7.87 (d, J = 8.4 Hz, 2H), 7.63-7.60 (m, 2H), 7.50-7.46 (m, 3H), 6.81 (d, J = 1.2 Hz, 1H), 6.68 (d, J = 8.4 Hz, 2H), 6.44 (s, 1H), 2.41 (s, 3H), 2.21 (d, J = 10.0 Hz, 9H), 1.75-1.64 (m, 6H). ^{13}C NMR (100 MHz, $CDCl_3$): δ 170.8, 162.2, 157.0, 145.8, 143.8, 129.3, 126.9, 123.2, 115.4, 103.4, 73.7, 53.5, 44.2, 37.2, 36.8, 36.6, 35.7, 35.1, 33.9, 31.0, 28.6, 27.5, 21.6, 20.9, 19.7, 19.0, 12.3; IR (KBr, v, cm^{-1}) 2910, 1661, 1575, 1456, ,1055, 766, 697; HRMS (ESI-TOF) calcd for $C_{29}H_{29}N_2O_3$ $[M-H]^-$ 453.2178, found 453.2189.

(1R,2S,5R)-5-Methyl-2-(prop-1-en-2-yl)cyclohexyl 4-((6-methyl-2-oxo-4-phenylpyridin-1(2H)-yl)amino)benzoate (86)

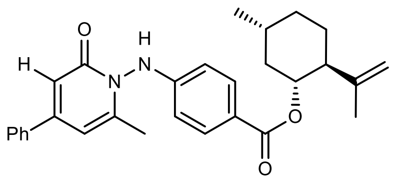

brown solid (55.7 mg, 61% yield); mp: 147-148 °C; ^1H NMR (400 MHz, $CDCl_3$): δ 7.90 (d, J = 8.4 Hz, 2H), 7.80 (s, 1H), 7.63-7.59 (m, 2H), 7.50-7.45 (m, 3H), 6.81 (s, 1H), 6.69 (d, J = 8.4 Hz, 2H), 6.44 (s, 1H), 5.00-4.93 (m, 1H), 4.72 (d, J = 25.2 Hz, 2H), 2.41 (s, 3H), 2.29-2.23 (m, 1H), 2.14 (d, J = 12.4 Hz, 1H), 1.72 (d, J = 12.0 Hz, 2H), 1.67 (s, 3H), 1.48-1.43 (m, 1H), 1.12-1.09 (m, 1H), 1.02-0.97 (m, 1H), 0.94 (d, J = 6.8 Hz, 3H). ^{13}C NMR (100 MHz, $CDCl_3$): δ 165.7, 152.1, 150.6, 148.8, 146.3, 137.5, 131.5, 129.8, 129.2, 127.0, 124.8, 114.4, 113.3, 112.0, 105.9, 74.1, 51.0, 40.7, 34.3, 31.5, 30.6, 22.2, 19.6; IR (KBr, v, cm^{-1}) 2926, 1663, 1577, 1458, 1012, 766, 697; HRMS (ESI-TOF) calcd for $C_{29}H_{31}N_2O_3$ $[M-H]^-$ 455.2335, found 455.2357.

2.2.2 Application of 2-pyridinone-based cyclic products

2.2.2.1 Gram-scale experiment for the synthesis of product 11

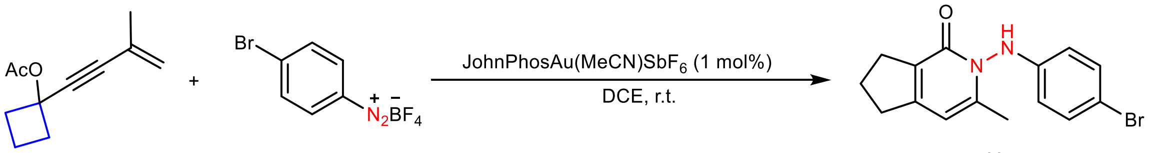

Supplementary Figure 6 Synthesis of Compound **11**

To a 50-mL Schlenk tube under air conditions, 1-(3-methylbut-3-en-1-yn-1-yl)cyclobutyl acetate (**1a**, 5 mmol, 1.0 equiv, 0.89 g), benzenediazonium tetrafluoroborate (**2k**, 10 mmol, 2.0 equiv, 2.52 g), JohnPhosAu(MeCN)SbF_6 (1 mol%, 38.61 mg), and 1,2-dichloroethane (15 mL) were successively added. The mixture was stirred at room temperature for about 6 hours. After the reaction was completed (indicated by TLC, petroleum ether : ethyl acetate = 2:1 v/v), the reaction mixture was concentrated by vacuum distillation and was purified by flash column chromatography (eluent: petroleum ether: ethyl acetate = 2:1 v/v) to afford the desired pure product **11** (1.12 g, 70% yield) as brown solid.

2.2.2.2 Gram-scale experiment for the synthesis of product 78

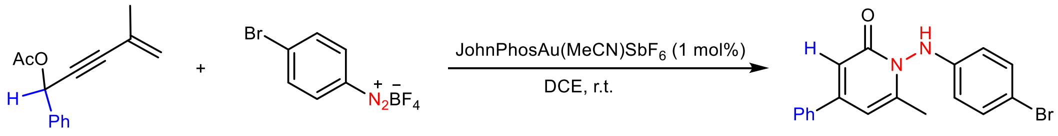

Supplementary Figure 7 Synthesis of Compound **78**

To a 10-mL Schlenk tube under air conditions, 1-(3-methylbut-3-en-1-yn-1-yl)cyclobutyl acetate (**1n**, 5 mmol, 1.0 equiv, 1.07 g), benzenediazonium tetrafluoroborate (**2k**, 10 mmol, 2.0 equiv, 2.52 g), JohnPhosAu(MeCN)SbF_6 (1 mol%, 38.61 mg), and 1,2-dichloroethane (15 mL) were successively added. The mixture was stirred at room temperature for about 6 hours. After the reaction was completed (indicated by TLC, petroleum ether : ethyl acetate = 2:1 v/v), the reaction mixture was concentrated by vacuum distillation and was purified by flash column chromatography (eluent, petroleum ether/ethyl acetate = 2:1 v/v) to afford the desired pure product **78** (1.46 g, 82% yield) as brown solid.

2.2.2.3 General procedure for the synthesis of product 87

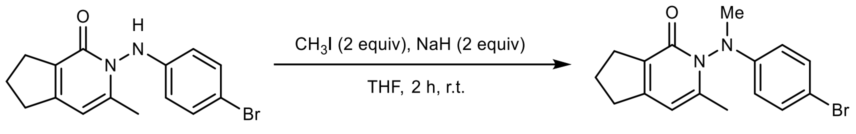

11 87

Supplementary Figure 8 Synthesis of Compound **87**

To a solution of 2-((4-bromophenyl)amino)-3-methyl-2,5,6,7-tetrahydro-1*H*-cyclopenta[*c*]pyridin-1-one (**11**, 0.2 mmol, 1.0 equiv, 63.84 mg) in THF (2 mL) at 0 °C, NaH (0.4 mmol, 2.0 equiv, 9.6 mg) was added. The reaction mixture was stirred for 0.5 hour at room temperature. The solution was added with CH_3I (0.4 mmol, 2.0 equiv, 56.78 mg) and stirred for 12 hours. The reaction mixture was then quenched with saturated aq. NH_4Cl and extracted with ethyl acetate (3 x 20 mL). The combined organic layers were washed with brine, dried over anhydrous Na_2SO_4 and concentrated. The residue was purified by column chromatography (eluent, petroleum ether/ethyl acetate = 5:1 v/v) on silica gel to afford **87** (52.0 mg, 78% yield).

2-((4-Bromophenyl)(methyl)amino)-3-methyl-2,5,6,7-tetrahydro-1H-cyclopenta[c]pyridin-1-one (87)

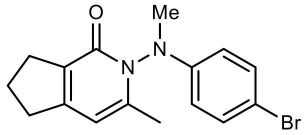

brown solid (52.0 mg, 78% yield); mp: 163-164 °C; ^{1}H NMR (400 MHz, $CDCl_3$): δ 7.31 (d, J = 8.8 Hz, 2H), 6.42 (d, J = 9.2 Hz, 2H), 6.10 (s, 1H), 3.37 (s, 3H), 2.84-2.77 (m, 4H), 2.26 (s, 3H), 2.10-2.02 (m, 2H); ^{13}C NMR (100 MHz, $CDCl_3$): δ 160.2, 155.0, 147.4, 147.2, 132.1, 131.5, 113.5, 111.9, 103.9, 39.2, 34.4, 30.1, 23.6, 19.1.; IR (KBr, v, cm^{-1}) 3374, 2948, 1652, 1454, 1113, 1032, 665; HRMS (ESI-TOF) calcd for $C_{16}H_{18}BrN_2O$ $[M+H]^+$ 333.0603, found 333.0601.

2.2.2.4 General procedure for the synthesis of product 88

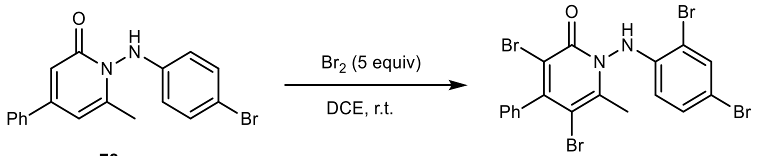

78 88

Supplementary Figure 9 Synthesis of Compound **88**

To a solution of 1-((4-bromophenyl)amino)-6-methyl-4-phenylpyridin-2(1H)-one (**78,** 0.2 mmol, 1.0 equiv, 71.05 mg) in THF (2 mL) at 0 °C, Br_2 (5.0 equiv) was added. The reaction mixture was stirred for 24 hours at room temperature. The reaction mixture was then quenched with saturated aq. $Na_2S_2O_3$

and extracted with DCM (3 x 20 mL). The residue was purified by column chromatography (eluent, petroleum ether/ethyl acetate = 5:1 v/v) on silica gel to afford **88** (97.08 mg, 82% yield).

3,5-dibromo-1-((2,4-dibromophenyl)amino)-6-Methyl-4-phenylpyridin-2(1H)-one (88)

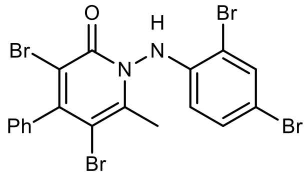

white solid (97.1 mg, 82% yield); mp: 131-133 °C; ^{1}H NMR (400 MHz, $CDCl_3$): δ 7.72 (d, J = 1.6 Hz, 1H), 7.68 (s, 1H), 7.52-7.47 (m, 3H), 7.32 (d, J = 8.4 Hz, 1H), 7.20 (d, J = 13.2 Hz, 2H), 6.26 (d, J = 8.4 Hz, 1H), 2.66 (s, 3H). ^{13}C NMR (100 MHz, $CDCl_3$): δ 157.7, 154.0, 146.5, 142.1, 140.0, 135.4, 131.5, 128.9, 128.6, 115.2, 114.6, 114.4, 111.4, 101.4, 20.0; IR (KBr, v, cm^{-1}) 3059, 1660, 1567, 1474, 1384, 1035, 698; HRMS (ESI-TOF) calcd for $C_{29}H_{29}N_2O_3$ [M-H]$^-$ 590.7564, found 590.7562.

2.2.2.5 General procedure for the synthesis of product 89

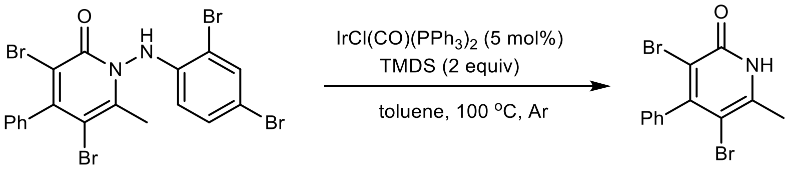

Supplementary Figure 10 Synthesis of Compound **89**

To a 10-mL Schlenk tube under Ar conditions, 3,5-dibromo-1-((2,4-dibromophenyl)amino)-6-methyl-4-phenylpyridin-2(1H)-one (**88,** 0.16 mmol, 1.0 equiv), $IrCl(CO)(PPh_3)_2$ (5 mol%), TMDS (2.0 equiv, 42.67 mg), and toluene (2.5 mL) were successively added. Then, the tube was stirred at 100 °C for 15 hours until complete consumption of **88**, as monitored by TLC analysis. After the reaction was completed, the reaction mixture was concentrated in vacuum and the resulting residue was purified by column chromatography on silica gel (eluent, petroleum ether/ethyl acetate = 10:1 v/v) to afford the desired product **89** as white solid (34.0 mg, 62% yield).

3,5-dibromo-6-methyl-4-phenylpyridin-2(1H)-one (89)

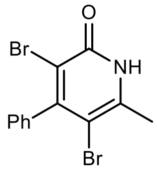

white solid (34.0 mg, 62% yield; ^{1}H NMR (400 MHz, $CDCl_3$): δ 8.62 (s, 1H), 7.52–7.45 (m, 3H), 7.19 (d, J = 7.6 Hz, 2H), 2.71 (s, 3H). ^{13}C NMR (100 MHz, $CDCl_3$): δ 157.3, 150.8, 149.1, 139.5, 128.8,

128.6, 128.3, 123.4, 119.3, 25.9; IR (KBr, ν, cm^{-1}) 2921, 1558, 1435, 1351, 1043, 913, 744; HRMS (ESI-TOF) calcd for $C_{12}H_8Br_2NO$ $[M-H]^-$ 341.8952, found 341.8977.

2.2.2.6 General procedure for the synthesis of product 90

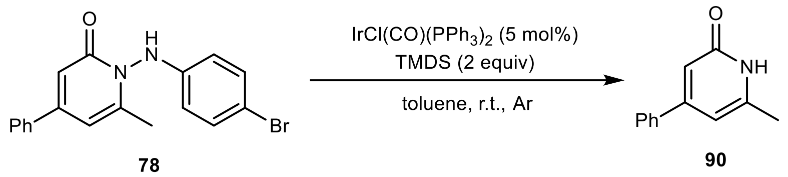

Supplementary Figure 11 Reaction Synthesis of Compound **90**

To a 10-mL Schlenk tube under Ar conditions, 1-((4-bromophenyl)amino)-6-methyl-4-phenylpyridin-2(1H)-one (**78**, 0.2 mmol, 1.0 equiv, 71.05 mg), $IrCl(CO)(PPh_3)_2$ (5 mol%, 7.80 mg), TMDS (2.0 equiv, 53.34 mg), and toluene (2.5 mL) were successively added. Then, the tube was stirred at room temperature for 15 hours until complete consumption of **78**, as monitored by TLC analysis. After the reaction was completed, the reaction mixture was concentrated in vacuum and the resulting residue was purified by column chromatography on silica gel (eluent, petroleum ether/ethyl acetate = 10:1 v/v) to afford the desired product **90** as white solid (29.6 mg, 80% yield).

6-methyl-4-phenylpyridin-2(1H)-one (90)

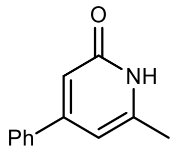

white solid (29.6 mg, 80% yield; ^1H NMR (400 MHz, $CDCl_3$): δ 8.54 (d, J = 5.2 Hz, 1H), 7.63 (d, J = 7.6 Hz, 2H), 7.51–7.43 (m, 3H), 7.38 (s, 1H), 7.32 (d, J = 5.2 Hz, 1H), 2.63 (s, 3H). ^{13}C NMR (100 MHz, $CDCl_3$): δ 158.9, 149.6, 148.9, 138.5, 129.1, 129.0, 127.1, 121.4, 119.0, 24.6; IR (KBr, ν, cm^{-1}) 2923, 1683, 1540, 1456, 1257, 1024, 746; HRMS (ESI-TOF) calcd for $C_{12}H_{11}NO$ $[M+H]^+$ 186.0919, found 186.0919.

2.3 Mechanism Details

2.3.1 Control experiment A

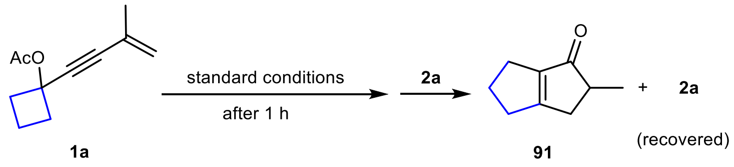

Supplementary Figure 12 Control Experiment A

To a 10-mL Schlenk tube under air conditions, 1-(3-methylbut-3-en-1-yn-1-yl)cyclobutyl acetate (**1a**, 0.2 mmol, 1.0 equiv, 35.65 mg), JohnPhosAu(MeCN)SbF_6 (1 mol%, 1.54 mg), and 1,2-dichloroethane (2 mL) were successively added. After 1 hour, benzenediazonium tetrafluoroborate (**2a**, 0.4 mmol, 2.0 equiv, 76.77 mg) was add. Then, the mixture was stirred at room temperature for about 5 hours. The desired product **3** was not detected by TLC.

2.3.2 Control experiment B

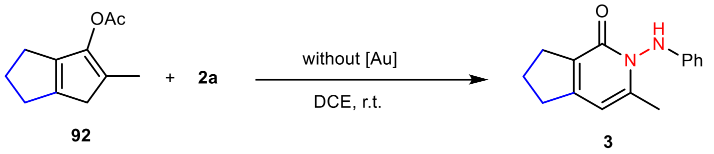

Supplementary Figure 13 Control Experiment B

To a 10-mL Schlenk tube under air conditions, 2-methyl-3,4,5,6-tetrahydropentalen-1-yl acetate (**92**, 0.2 mmol, 1.0 equiv, 35.65 mg), benzenediazonium tetrafluoroborate (**2a**, 0.4 mmol, 2.0 equiv, 76.77 mg), and 1,2-dichloroethane (2 mL) were successively added. The mixture was stirred at room temperature for about 6 hours. After the reaction was completed (indicated by TLC, petroleum ether : ethyl acetate = 2:1 v/v), the reaction mixture was concentrated by vacuum distillation and was purified by flash column chromatography (eluent: petroleum ether: ethyl acetate = 2:1 v/v) to afford the desired pure product (**3**, 24.0 mg, 50% yield) as white solid.

2.3.3 Control experiment C

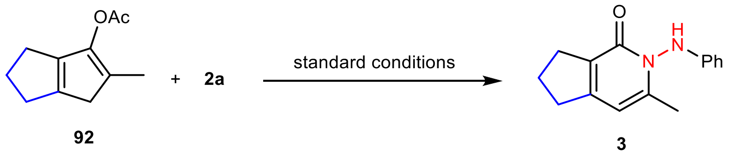

Supplementary Figure 13 Control Experiment C

To a 10-mL Schlenk tube under air conditions, 2-methyl-3,4,5,6-tetrahydropentalen-1-yl acetate (**92**, 0.2 mmol, 1.0 equiv, 35.65 mg), benzenediazonium tetrafluoroborate (**2a**, 0.4 mmol, 2.0 equiv, 76.77 mg), JohnPhosAu(MeCN)SbF_6 (1 mol%, 1.54 mg), and 1,2-dichloroethane (2 mL) were successively added. The mixture was stirred at room temperature for about 6 hours. After the reaction was completed (indicated by TLC, petroleum ether: ethyl acetate = 2:1 v/v), the reaction mixture was concentrated by vacuum distillation and was purified by flash column chromatography (eluent: petroleum ether: ethyl acetate = 2:1 v/v) to afford the desired pure product (**3**, 29.9 mg, 56% yield) as white solid.

2.3.4 Control experiment D

Supplementary Table 1 Control Experiment D

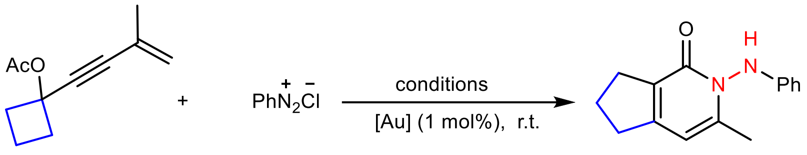

entry	variation	Yield (%)
1	DCE	0
2	$AgBF_4$ (2.0 equiv), DCE	58
3	AgOAc (2.0 equiv), DCE	0
4	$AgBF_4$ (2.0 equiv), dry DCE	0
5	$AgBF_4$ (2 equiv), H_2O (1.0 equiv), dry DCE	61%

Entry 1: To a 10-mL Schlenk tube under air conditions, 1-(3-methylbut-3-en-1-yn-1-yl)cyclobutyl acetate (**1a**, 0.2 mmol, 1 equiv, 35.65 mg), benzenediazonium chloride (0.4 mmol, 2.0 equiv, 56.23 mg), JohnPhosAu(MeCN)SbF_6 (1 mol%, 1.54 mg), and 1,2-dichloroethane (2 mL) were successively added. The mixture was stirred at room temperature for about 6 hours. The desired product **3** was not detected by TLC.

Entry 2: To a 10 mL Schlenk tube under air conditions, 1-(3-methylbut-3-en-1-yn-1-yl)cyclobutyl acetate (**1a**, 0.2 mmol, 1.0 equiv, 35.65 mg), benzenediazonium chloride (0.4 mmol, 2.0 equiv, 56.23 mg), JohnPhosAu(MeCN)SbF_6 (1 mol%, 1.54 mg), $AgBF_4$ (0.4 mmol, 2.0 equiv, 77.87 mg), and 1,2-dichloroethane (2 mL) were successively added. The mixture was stirred at room temperature for about 6 hours. After the reaction was completed (indicated by TLC, petroleum ether : ethyl acetate = 2:1 v/v), the reaction mixture was concentrated by vacuum distillation and was purified by flash column chromatography (eluent: petroleum ether: ethyl acetate = 2:1 v/v) to afford the desired pure product **3** (30.97 mg, 58% yield) as white solid.

Entry 3: To a 10-mL Schlenk tube under air conditions, 1-(3-methylbut-3-en-1-yn-1-yl)cyclobutyl acetate (**1a**, 0.2 mmol, 1.0 equiv, 35.65 mg), benzenediazonium chloride (0.4 mmol, 2.0 equiv, 56.23 mg), JohnPhosAu(MeCN)SbF_6 (1 mol%, 1.54 mg), AgOAc (0.4 mmol, 2.0 equiv, 66.76 mg), and 1,2-dichloroethane (2 mL) were successively added. The mixture was stirred at room temperature for about 6 hours. The desired product **3** was not detected by TLC.

Entry 4: To a 10-mL Schlenk tube under argon conditions, 1-(3-methylbut-3-en-1-yn-1-yl)cyclobutyl acetate (**1a**, 0.2 mmol, 1.0 equiv, 35.65 mg), benzenediazonium chloride (0.4 mmol, 2.0 equiv, 56.23 mg), JohnPhosAu(MeCN)SbF_6 (1 mol%, 1.54 mg), $AgBF_4$ (0.4 mmol, 2.0 equiv, 77.87 mg), and dry

1,2-dichloroethane (2 mL) were successively added. The mixture was stirred at room temperature for about 6 hours. The desired product **3** was not detected by TLC.

Entry 5: To a 10-mL Schlenk tube under argon conditions, 1-(3-methylbut-3-en-1-yn-1-yl)cyclobutyl acetate (**1a**, 0.2 mmol, 1.0 equiv, 35.65 mg), benzenediazonium chloride (0.4 mmol, 2.0 equiv, 56.23 mg), JohnPhosAu(MeCN)SbF_6 (1 mol%, 1.54 mg), $AgBF_4$ (0.4 mmol, 2.0 equiv, 77.87 mg), H_2O (0.2 mmol, 3.60 mg) and dry 1,2-dichloroethane (2 mL) were successively added. The mixture was stirred at room temperature for about 6 hours. The desired product **3** was detected by TLC. After the reaction was completed (indicated by TLC, petroleum ether : ethyl acetate = 2:1 v/v), the reaction mixture was concentrated by vacuum distillation and was purified by flash column chromatography (eluent: petroleum ether: ethyl acetate = 2:1 v/v) to afford the desired pure product **3** (32.57 mg, 61% yield) as white solid.

Intermediate Trapping Experiments

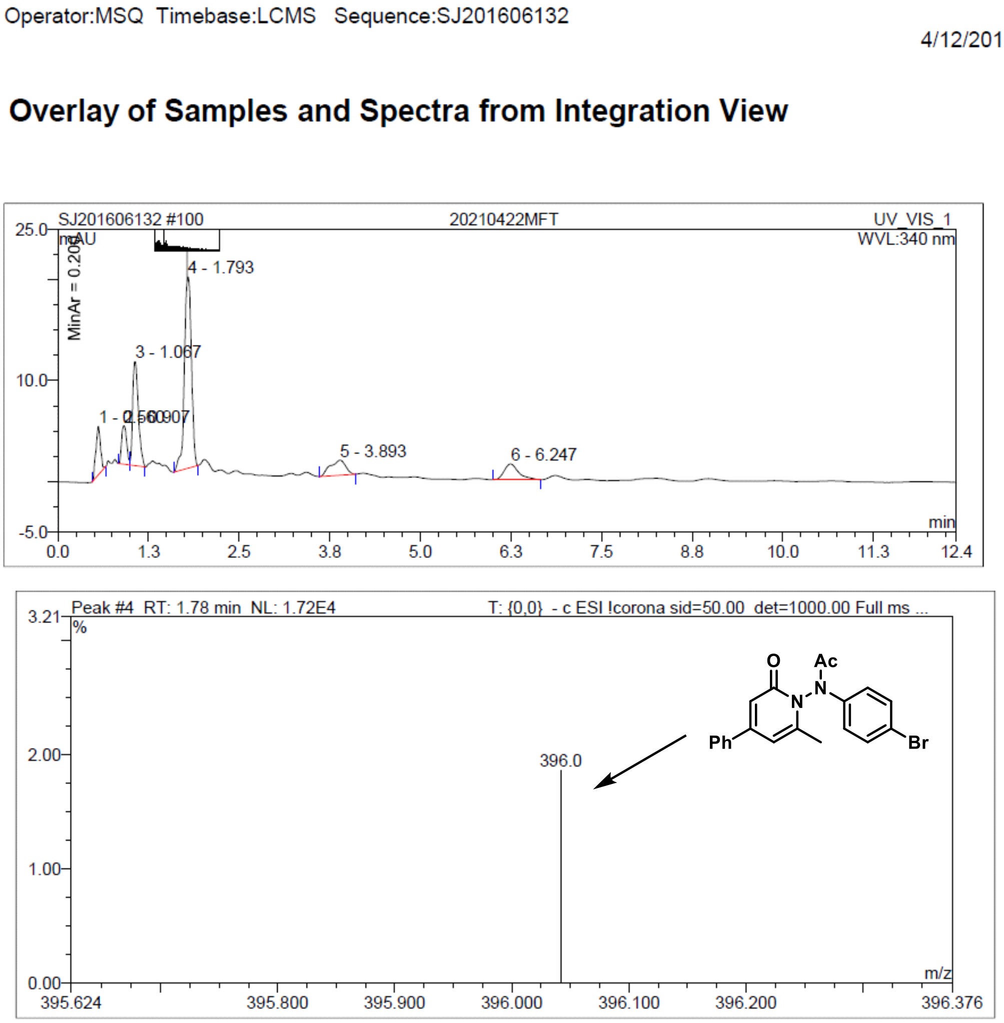

Supplementary Figure 14 Intermediate Trapping Experiments

2.3.5 Control experiment E

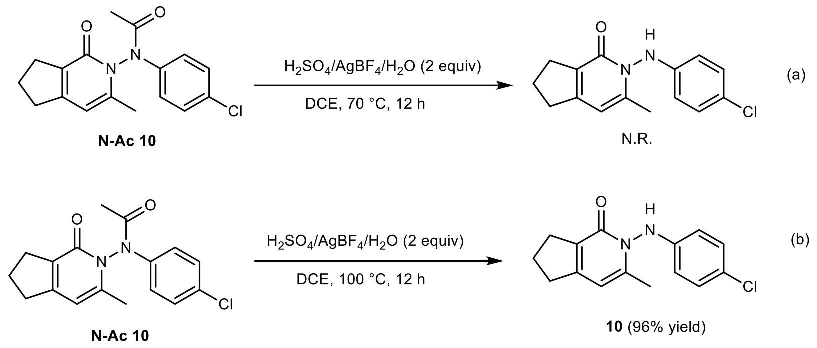

Supplementary Figure 15 The Hydrolysis Reaction of ***N*-Ac 10**

Synthesis of *N*-Ac 10

To a 100-mL Schlenk tube under Ar conditions, 2-((4-chlorophenyl)amino)-3-methyl-2,5,6,7-tetrahydro-1H-cyclopenta[*c*]pyridin-1-one (**10**, 2 mmol, 1 equiv, 0.549 g), NaH (4 mmol, 2 equiv, 0.160g), and THF (10 mL) were successively added. Then, the tube was stirred at 0 °C for 20 min. The solution was added with Ac_2O (3 mmol, 1.5 equiv, 0.306 g) and stirred for 1 hour, as monitored by TLC analysis. After the reaction was completed, the reaction mixture was concentrated in vacuum and the resulting residue was purified by column chromatography on silica gel (eluent, petroleum ether/ethyl acetate = 5:1) to afford the desired product ***N*-Ac 10** as a white solid (0.520 g, 82% yield). ^{1}H NMR (400 MHz, DMSO) (δ, ppm): 7.45 (d, J = 8.0 Hz, 2H), 7.27 (d, J = 8.0 Hz, 2H), 6.38 (s, 1H), 6.18 (s, 1H), 2.80 (d, J = 36.8 Hz, 4H), 2.19 (s, 3H), 2.05-1.99 (m, 2H), 1.93 (s, 3H); ^{13}C NMR (100 MHz, DMSO) (δ, ppm): 171.0, 159.6, 156.8, 146.1, 138.7, 129.9, 129.3, 124.2, 105.6, 30.5, 23.3, 22.1, 19.0.

Hydrolysis Reaction of *N*-Ac 10

To a 10-mL Schlenk tube under air conditions, *N*-(4-chlorophenyl)-*N*-(3-methyl-1-oxo-1,5,6,7-tetrahydro-2H-cyclopenta[*c*]pyridin-2-yl)acetamide (***N*-Ac 10**, 0.2 mmol, 1 equiv, 63.4 mg), H_2SO_4 (0.4 mmol, 2 equiv, 39.2 mg), $AgBF_4$ (0.4 mmol, 2 equiv, 77.9 mg), H_2O (0.4 mmol, 2 equiv, 7.3 mg), 1,2-dichloroethane (2 mL) were successively added. The mixture was stirred at 70 °C or 100 °C for about 12 hours. Compound ***N*-Ac 10** did not be consumed at 70 °C, but was completely converted to product **10** (detected by TLC analysis) when the reaction temperature was raised to 100 °C. After flash column chromatography (eluent: petroleum ether: ethyl acetate = 2:1 v/v), pure product **10** was isolated in 96% yield (53.5 mg).

2.4 The ORTEP Drawing of 5, 39 and 55

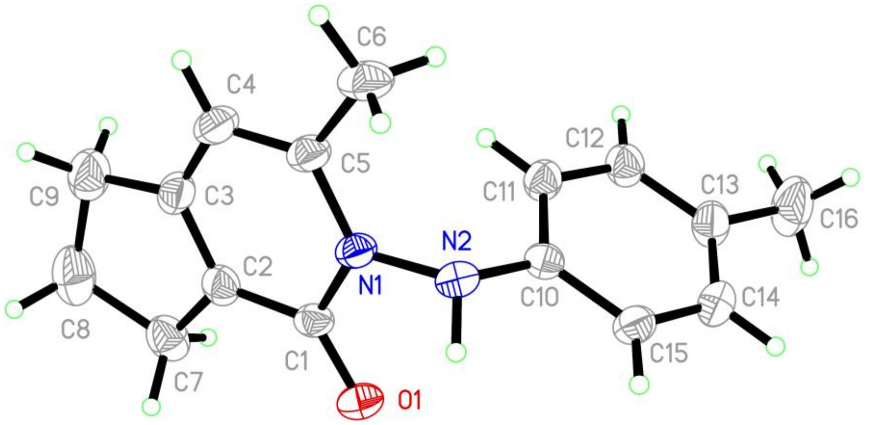

Supplementary Figure 16 The ORTEP Drawing of **5** (CCDC 2142493)

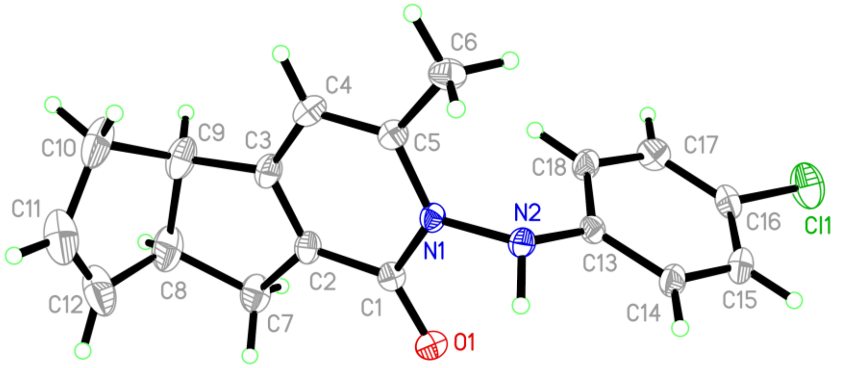

Supplementary Figure 17 The ORTEP Drawing of **39** (CCDC 2142494)

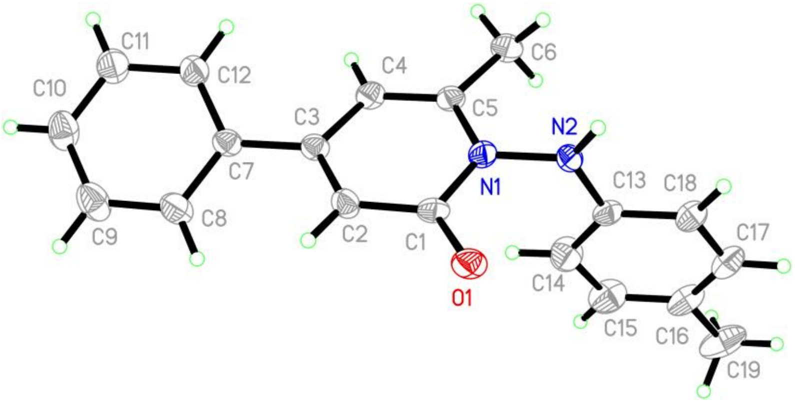

Supplementary Figure 18 The ORTEP Drawing of **55** (CCDC 2142495)

2.5 Computational Details

All the calculations were performed with the Gaussian 09 suite of programs by using density functional theory.[9] Geometry optimizations were carried out with the M06-L functional and the def2-SVP basis sets. The stationary points were optimized in dichloroethane (DCE) solvent with a SMD continuum solvation model.[10] Moreover, we further refined the energy by employing the single-point energy calculations at the M06-2X/ def2-TZVPP/$SMD_{dichloroethane}$ level based on the M06-L/def2-SVP/$SMD_{dichloroethane}$. All the optimized stationary points had been identified as minima (zero imaginary frequencies) and transition states (one imaginary frequency), via the vibrational analysis. The 3D diagrams of structures were generated using CYLView.[11]

2.5.1 Test Results of the Different Computational Methods

We have compared the relative Gibbs free energies ($\Delta G^{\ddagger}$) of key transition states, which are computed by the Gibbs free energy corrections at M06-L/def2-SVP/$SMD_{dichloroethane}$ (denoted as L1), M06-L/def2-SVP/$SMD_{dichloroethane}$ plus the refined single-point energies at M06-L/def2-TZVPP/$SMD_{dichloroethane}$ (L2), M06-L-D3(BJ)/def2-TZVPP/$SMD_{dichloroethane}$ (L3). In L3 level, we performed dispersion correction on key transition states to determine the accuracy of our calculations. The calculated result shows that the relative Gibbs free energy at L2 and L3 are quite close. Hence, the energy profiles of Figure 2 and Figure 4 in the revised manuscript have been updated at M06-L/def2-TZVPP/$SMD_{dichloroethane}$//M06-L/def2-SVP/$SMD_{dichloroethane}$ level.

Supplementary Table 2 Comparison of Relative Gibbs Free Energy Barrier ($\Delta G^{\ddagger}$) Differences of Key Transition States at Three Levels (unit: kcal/mol).

Species	L1	L2	L3
**TS4**	9.9	10.1	10.0
**TS5**	19.4	17.8	18.0
**TS6**	11.2	18.6	16.9
**TS7**	10.1	17.1	16.2
**TS11**	20.1	29.2	28.0

2.5.2 The Reaction of Five-Membered Ring Substrates

According to the information in the article, when n = 2, the reaction needs to be at 50 °C. We choose the simplest structure to consider the different reactivity of these substrates in Figure S19. We performed DFT calculations on key structures **M3a-M7a**, the five-membered ring reactant **R1a** also undergoes a six-membered ring transition state to form the oxonium intermediate **M3a**. Subsequent the terminal methylene electrophilic **M3a** results an intramolecular electrophilic 1,5-annulation via transition state **TS3a** to from Au-carbene complex **M4a** with an energy barrier of 12.5 kcal/mol. In the subsequent 1,2-carbocation rearrangement process shows different reactivity, the calculated free energy barrier for the 1,2-shift of five-membered ring *via* the transition state **TS4a** is 20.4 kcal/mol, which is much higher than that of 1,2-carbon cation rearrangement *via* transition state **TS4** in four-membered ring ($\Delta G^{\ddagger}$ = 10.1 kcal/mol). Therefore, when n = 2, the reaction needs of higher temperature.

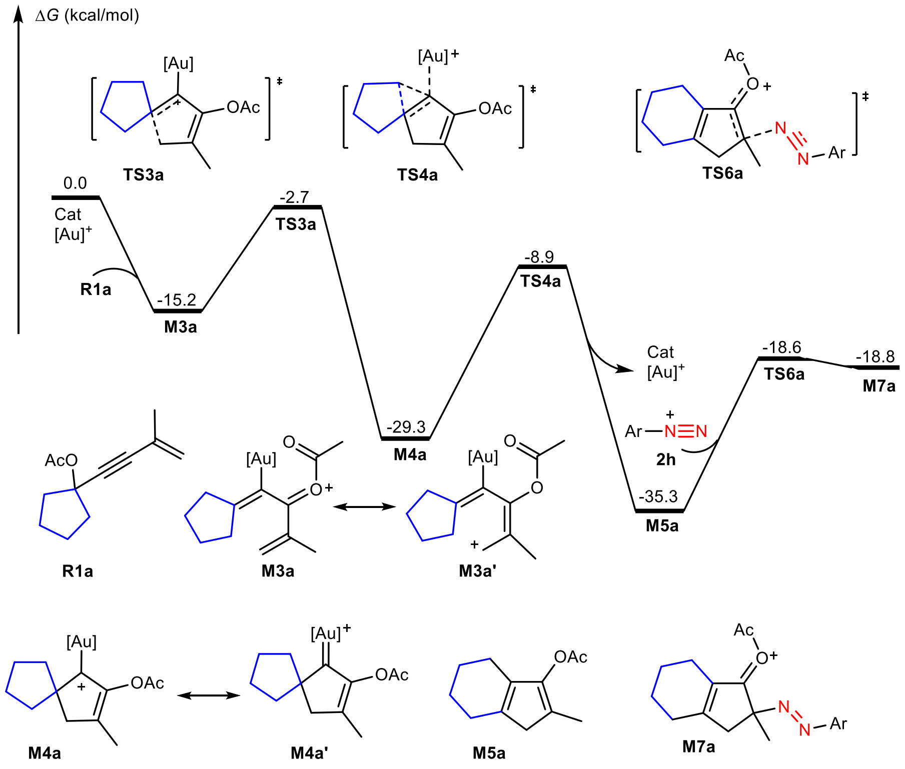

Supplementary Figure 19 The Energy Profile for the Cyclization of Five-Membered Ring Substrate **R1a**.

As shown in Figure 20, we also performed DFT calculations for the key step of the polycyclic substituted five-membered ring **R1l**. The 1,2-carbon cation rearrangement via transition state **TS4l** in polycyclic substituted five-membered ring is 21.3 kcal/mol, it indicates that the five-membered ring substrates need to react at a higher temperature, which is in good agreement with the experimental observations.

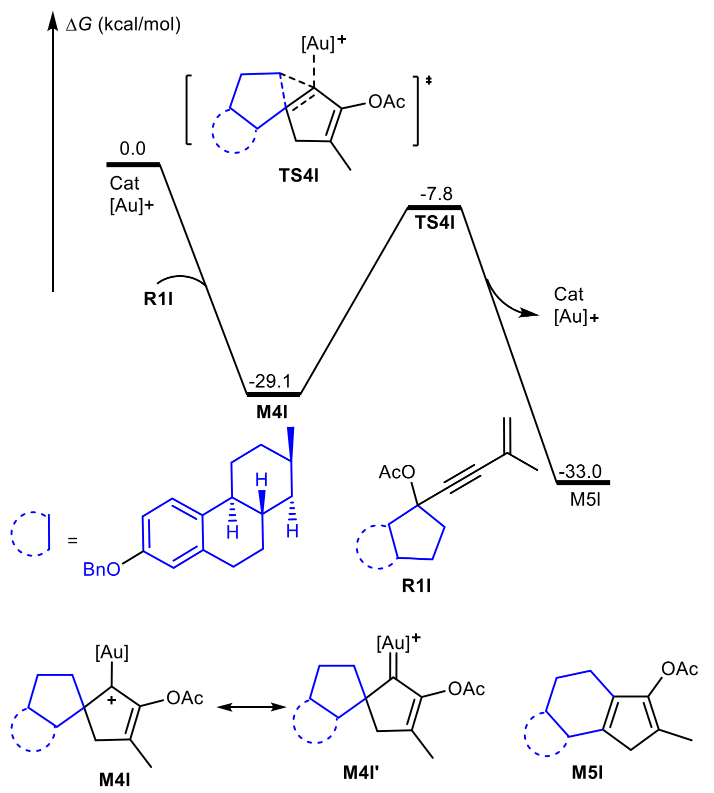

Supplementary Figure 20 The Energy Profile for the Cyclization of Polycyclic Substituted Five-Membered Ring **R1l**

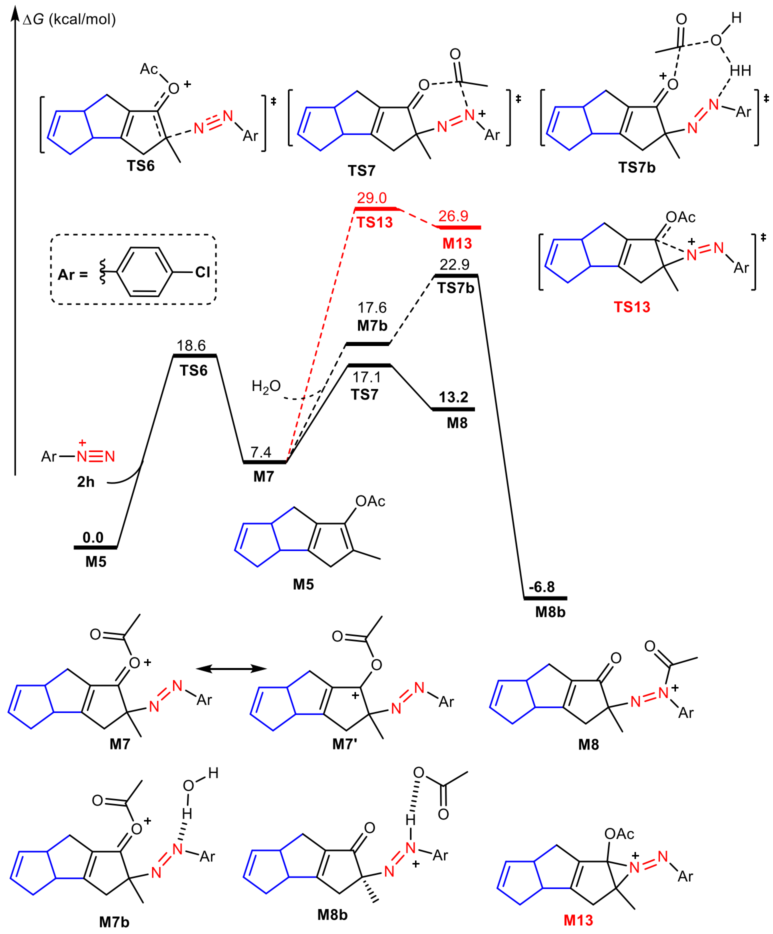

Supplementary Figure 21. The Free Energy Profile for the Hydrolysis Reaction of **M7**

Furthermore, we have also investigated the alternative pathway for the hydrolysis part. After the

formation of intermediate **M7**, the hydrolysis process has been considered as a stepwise pathway shown in the Figure S21. The intermediate **M7b** can be formed by the combination of **M7** and water molecule via a weak hydrogen bond with the azo moiety with the endergonic of 10.2 kcal/mol. Then, hydrolysis process would occur via an eight membered ring transition state **TS7b** with a free energy barrier of 5.3 kcal/mol. Comparing with the other two pathways, the hydrolysis process would be higher than the most favorable acyl transfer pathway for 5.8 kcal/mol, which shows the direct hydrolysis process from the intermediate **M7** would be not the most kinetic favorable pathway.

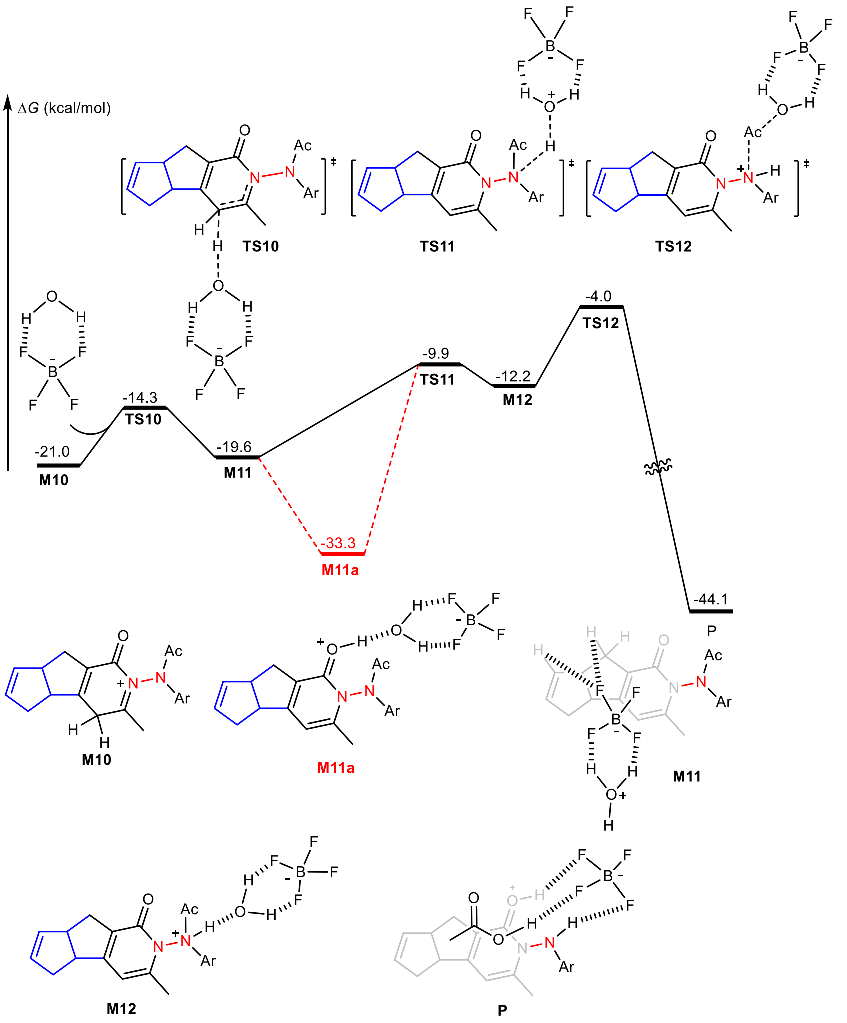

Supplementary Figure 22. The Free Energy Profile for the Hydrolysis Reaction of ***N*-Ac P39**

In control experiment E (**Supplementary Figure 15**), we observed that the hydrolysis of ***N*-Ac 10** did not work in DCE in the presence of H_2SO_4, $AgBF_4$ and H_2O at 70 °C. When the reaction temperature was raised to 100 °C, the reaction gave **10** in almost quantitative yield. Based on this observation, we have re-conducted DFT studies on our previously proposed reaction process and the hydrolysis of ***N*-Ac 10**. As shown in Figure S22, the calculated results show that the carbonyl moiety and the acid in intermediate **M11** can form a strong hydrogen bond, leading to formation of intermediate **M11a** with an exergonic of 13.7 kcal/mol. Therefore, the starting point in the experiment would be **M11a**, rather

than **M11**. The free energy barrier for the hydrolysis of ***N*-Ac P39** would be increased to 29.3 kcal/mol via transition state **TS12**, which would be hard to occur in the previous conditions. A higher reaction temperature is necessary, which is coincident with extra experimental observations. Furthermore, we believe that the acid moiety would like to form the complex with the final product via hydrogen bond with an exothermic of 40.1 kcal/mol. Hence, it is hard to simulate the real condition in the experiment.

2.5.3 Absolute Single-Point Energies and Gibbs Free Energy (GFE) of the Structures Optimized

Supplementary Table 3 The Single-Point Energies (E), Gibbs Free Energy Corrections (GFEC), and GFE (=E+GFEC) Energies of the Stationary Points Involved in the Reaction of Mechanistic (unit: a.u.)

SP	GFEC (M06-L/def2-SVP / $SMD_{dichloroethane}$)	*E* (M06-L/def2-TZVPP / $SMD_{dichloroethane}$)	GFE
**1g**	0.219437	-693.644207	-693.42477
**Cat**	0.366137	-1255.67181	-1255.305669
**M1**	0.609516	-1949.35332	-1948.743799
**TS1**	0.60968	-1949.33421	-1948.724525
**M2**	0.615069	-1949.34707	-1948.732002
**TS2**	0.611711	-1949.33921	-1948.727501
**M3**	0.609656	-1949.35345	-1948.743797
**TS3**	0.612855	-1949.33488	-1948.722029
**M4**	0.614077	-1949.37969	-1948.765615
**TS4**	0.616307	-1949.36383	-1948.749572
**M5**	0.224819	-693.71761	-693.492791
**TS5**	0.616484	-1949.35372	-1948.73724
**M6**	0.224913	-693.717352	-693.492439
**2h**	0.056723	-800.621939	-800.565216
**TS6**	0.306262	-1494.33466	-1494.028402
**M7**	0.307235	-1494.35346	-1494.046224
**TS7**	0.30861	-1494.33932	-1494.030711
**TS7a**	0.327948	-1570.800595	-1570.472647
**M8**	0.30907	-1494.34603	-1494.036964
**TS8**	0.307406	-1494.34309	-1494.03568
**M9**	0.306158	-1494.35635	-1494.050188
**TS9**	0.306997	-1494.3561	-1494.049102
**M10**	0.342372	-1995.62089	-1995.278516
**H_2O-BF_4^-**	0.004923	-501.191976	-501.187053

**TS10**	0.337047	-1995.60484	-1995.267794
**M11**	0.34277	-1995.61914	-1995.276368
**TS11**	0.340184	-1995.60108	-1995.260896
**M12**	0.340169	-1995.60464	-1995.264468
**TS12**	0.342334	-1995.59373	-1995.251391
**$AcOH_2^+BF_4^-$**	0.047834	-654.3082	-654.260366
**P-39**	0.341061	-1995.656421	-1995.31536
**TS13**	0.307424	-1494.318828	-1494.011404
**M13**	0.308861	-1494.323589	-1494.014728
**TS14**	0.307471	-1494.311742	-1494.004271
**M14**	0.339202	-1995.551684	-1995.212482
**TS15**	0.337435	-1995.54738	-1995.209945
**M15**	0.341016	-1995.573632	-1995.232616
**M16**	0.340168	-1995.629781	-1995.289613
**TS16**	0.3369	-1995.602022	-1995.265122
**R1a**	0.209309	-617.463905	-617.254596
**M3a**	0.601683	-1873.18622	-1872.584537
**TS3a**	0.600638	-1873.16516	-1872.564521
**M4a**	0.604606	-1873.2116	-1872.60699
**TS4a**	0.6071	-1873.18147	-1872.574373
**M5a**	0.21508	-617.526	-617.31092
**TS6a**	0.296525	-1418.1459	-1417.849378
**M7a**	0.297697	-1418.14742	-1417.84972
**R1l**	0.540191	-1467.06646	-1466.526271
**M4l**	0.939334	-2722.81759	-2721.878251
**TS4l**	0.936399	-2722.78071	-2721.844308
**M5l**	0.54442	-1467.12328	-1466.578862
**M11a**	0.344438	-1995.642524	-1995.298086
**H_2O**	0.003746	-76.454805	-76.451059
**M7b**	0.329222	-1570.810278	-1570.481056
**TS7b**	0.327948	-1570.800595	-1570.472647

**M8b**	0.330031	-1570.849936	-1570.519905

2.6 NMR spectra

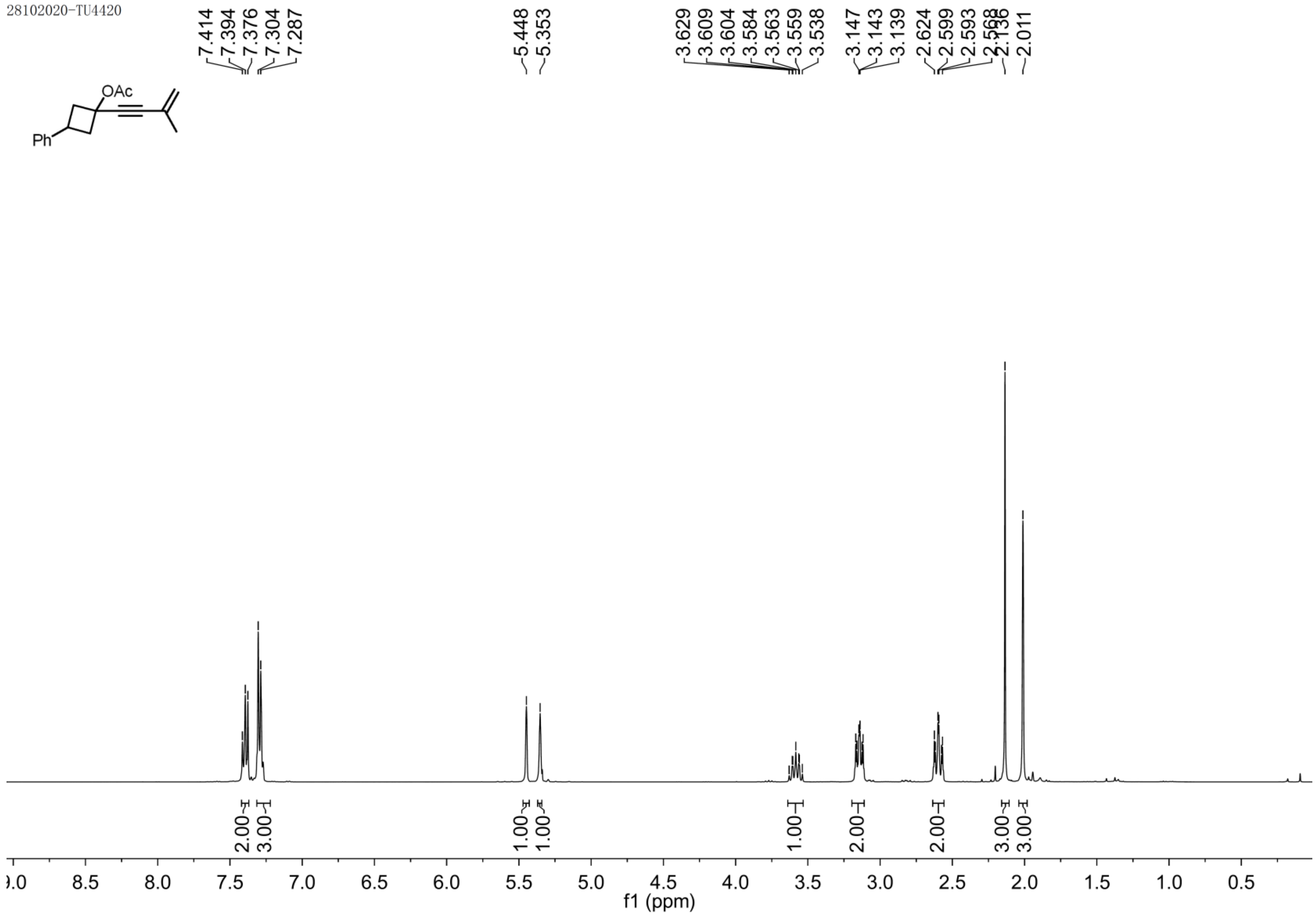

Supplementary Figure 23 ^{1}H NMR Spectrum of Compound **1c**

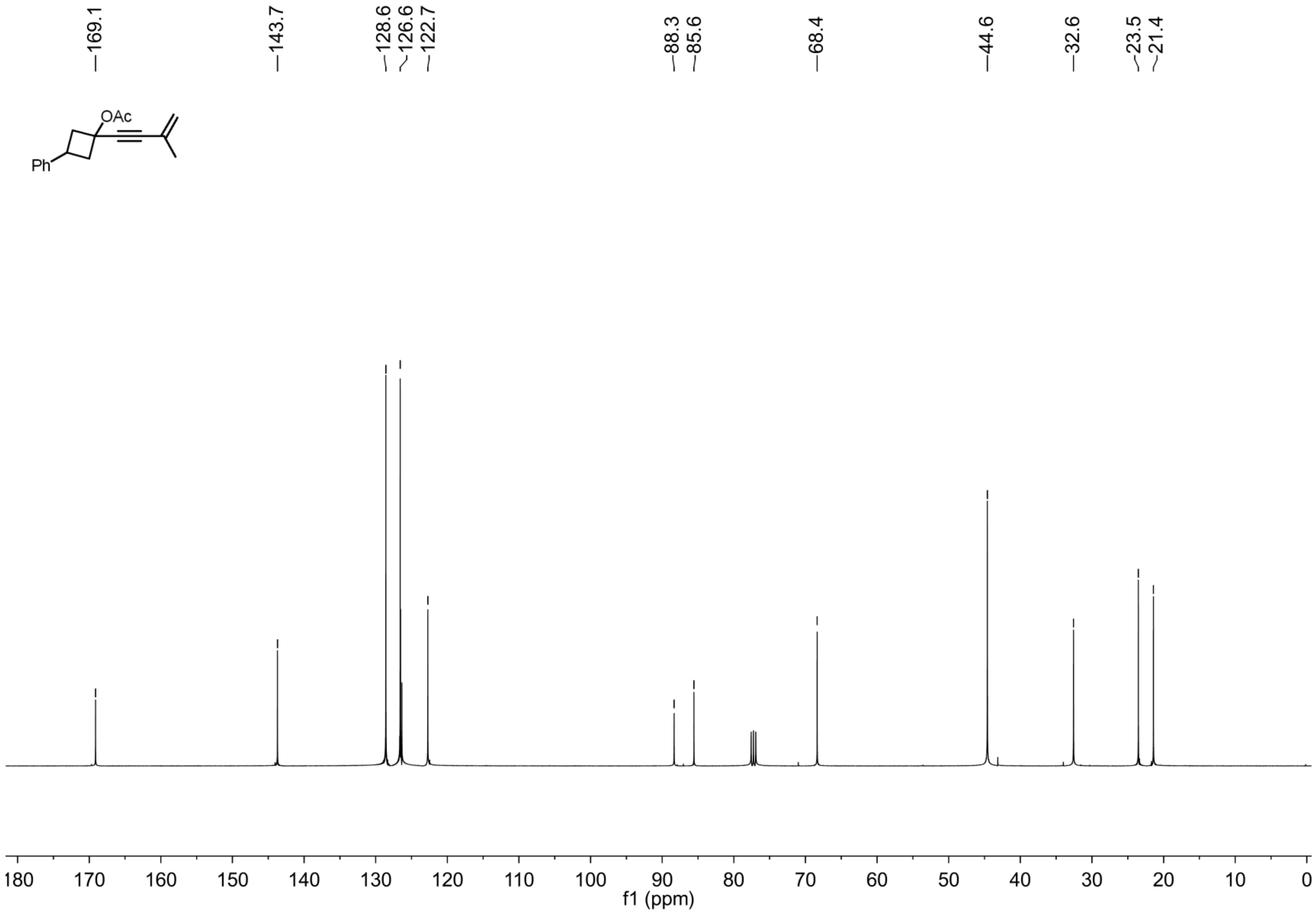

Supplementary Figure 24 ^{13}C NMR Spectrum of Compound **1c**

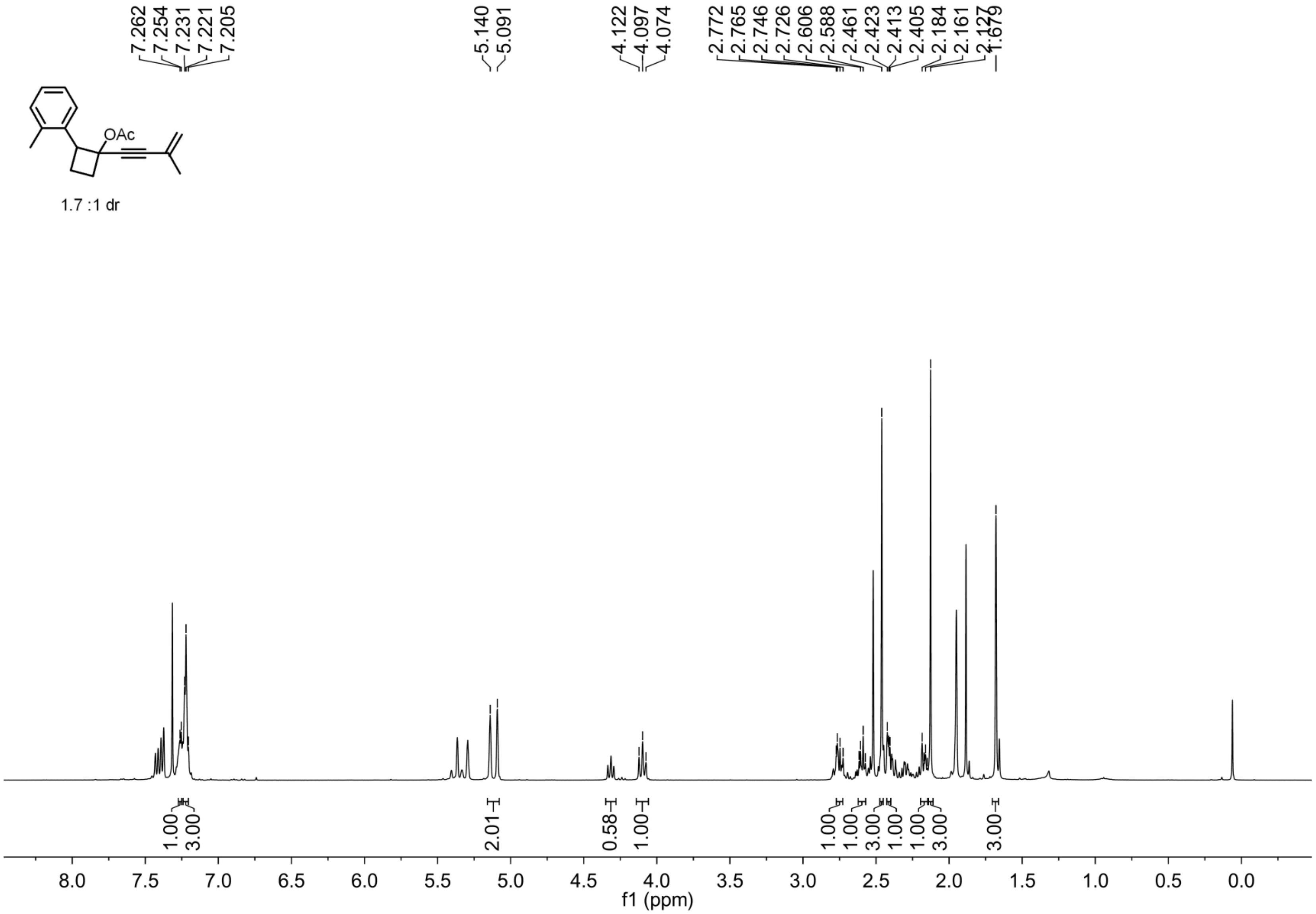

Supplementary Figure 25 ^{1}H NMR Spectrum of Compound **1d**

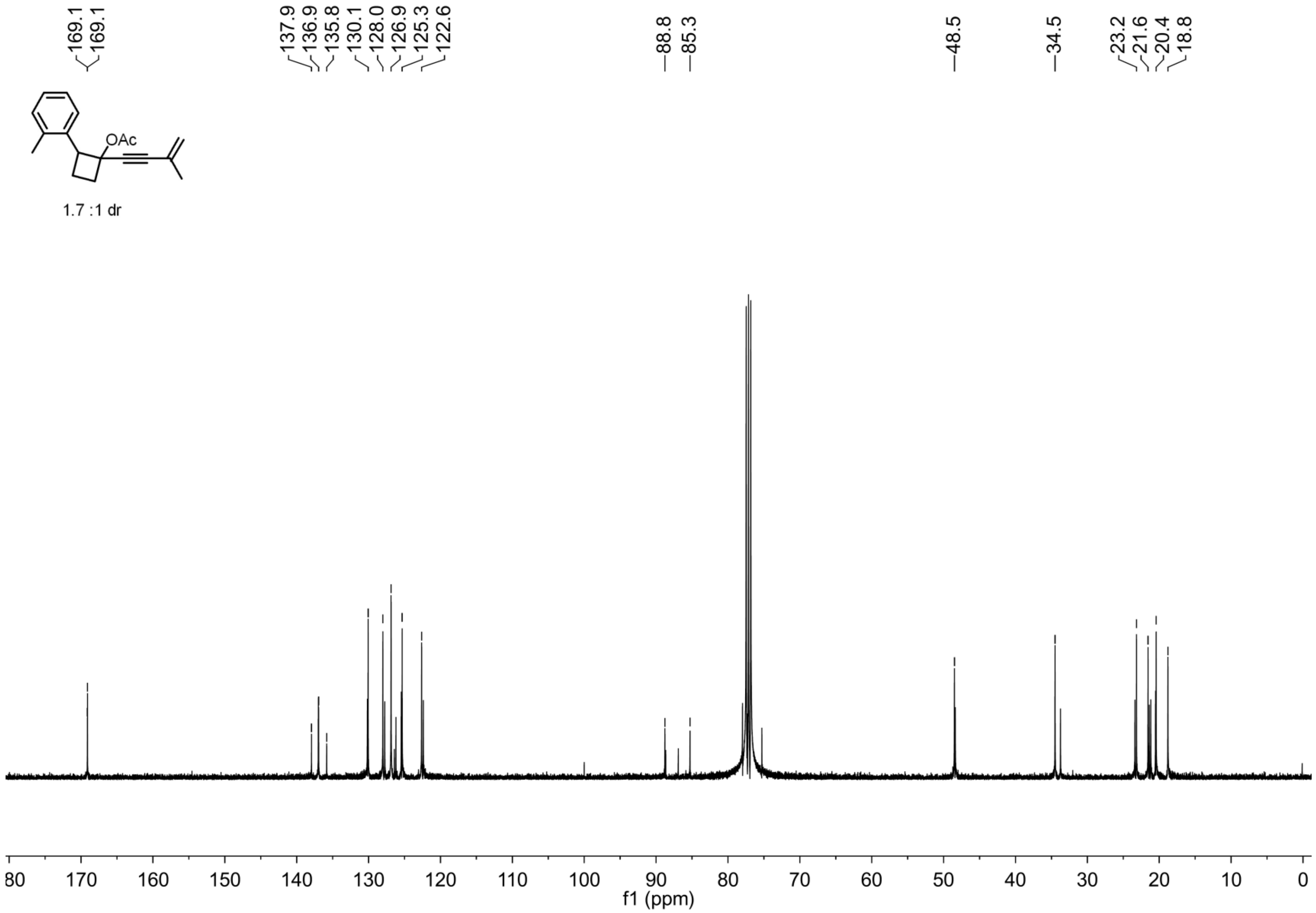

Supplementary Figure 26 ^{13}C NMR Spectrum of Compound **1d**

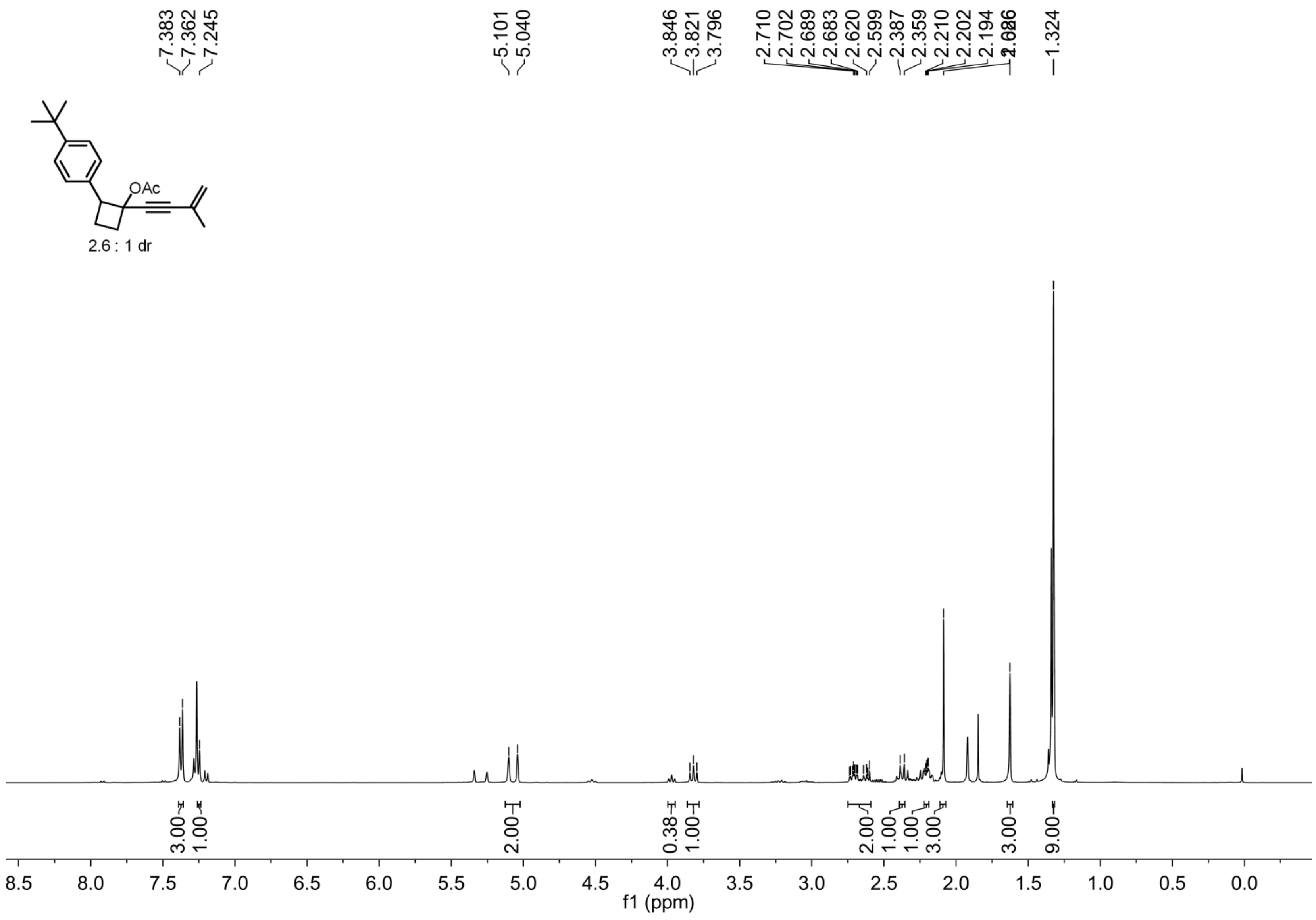

Supplementary Figure 27 ^{1}H NMR Spectrum of Compound **1f**

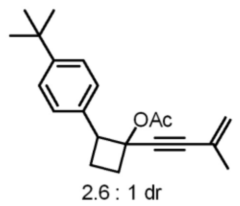

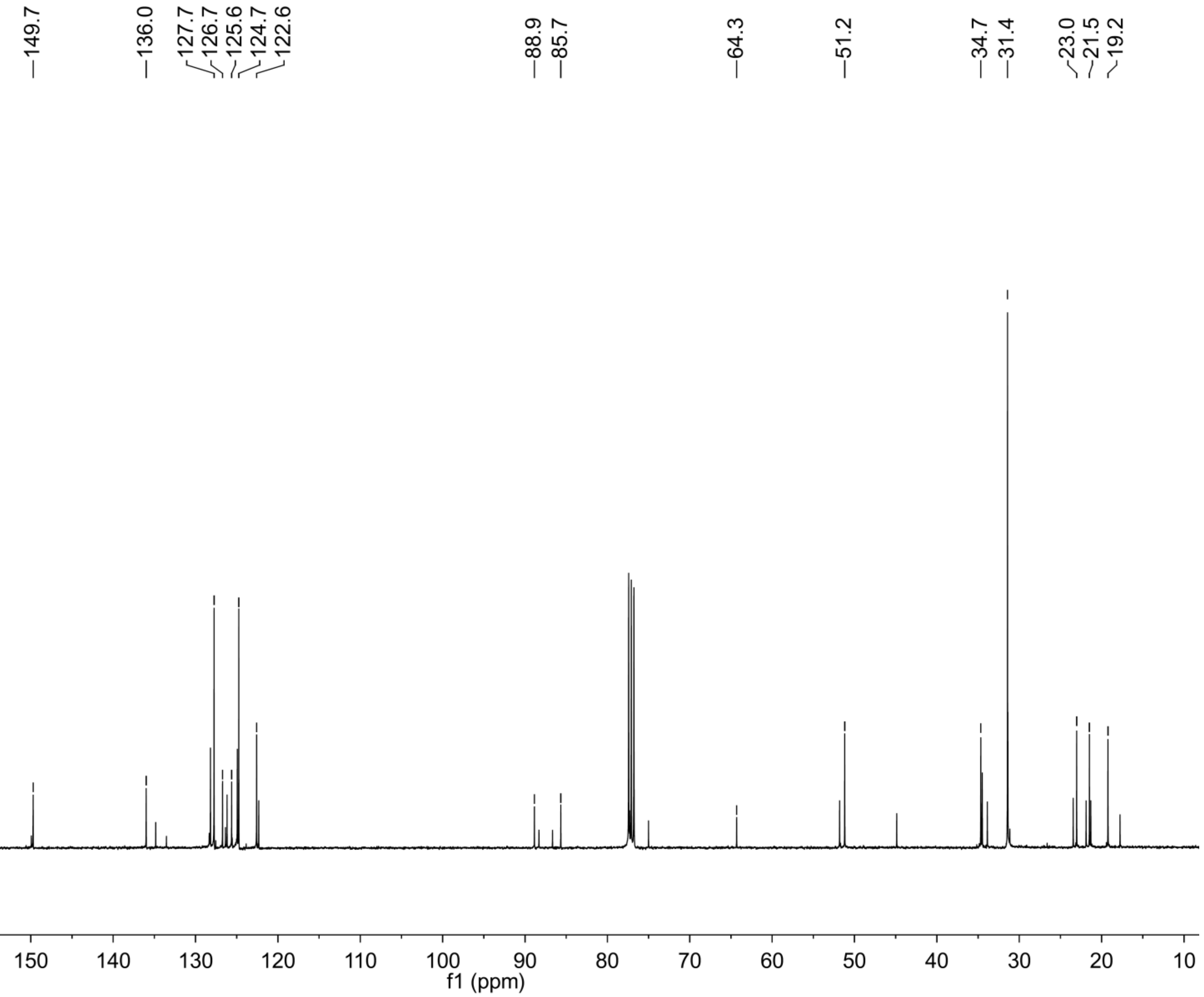

Supplementary Figure 28 ^{13}C NMR Spectrum of Compound **1f**

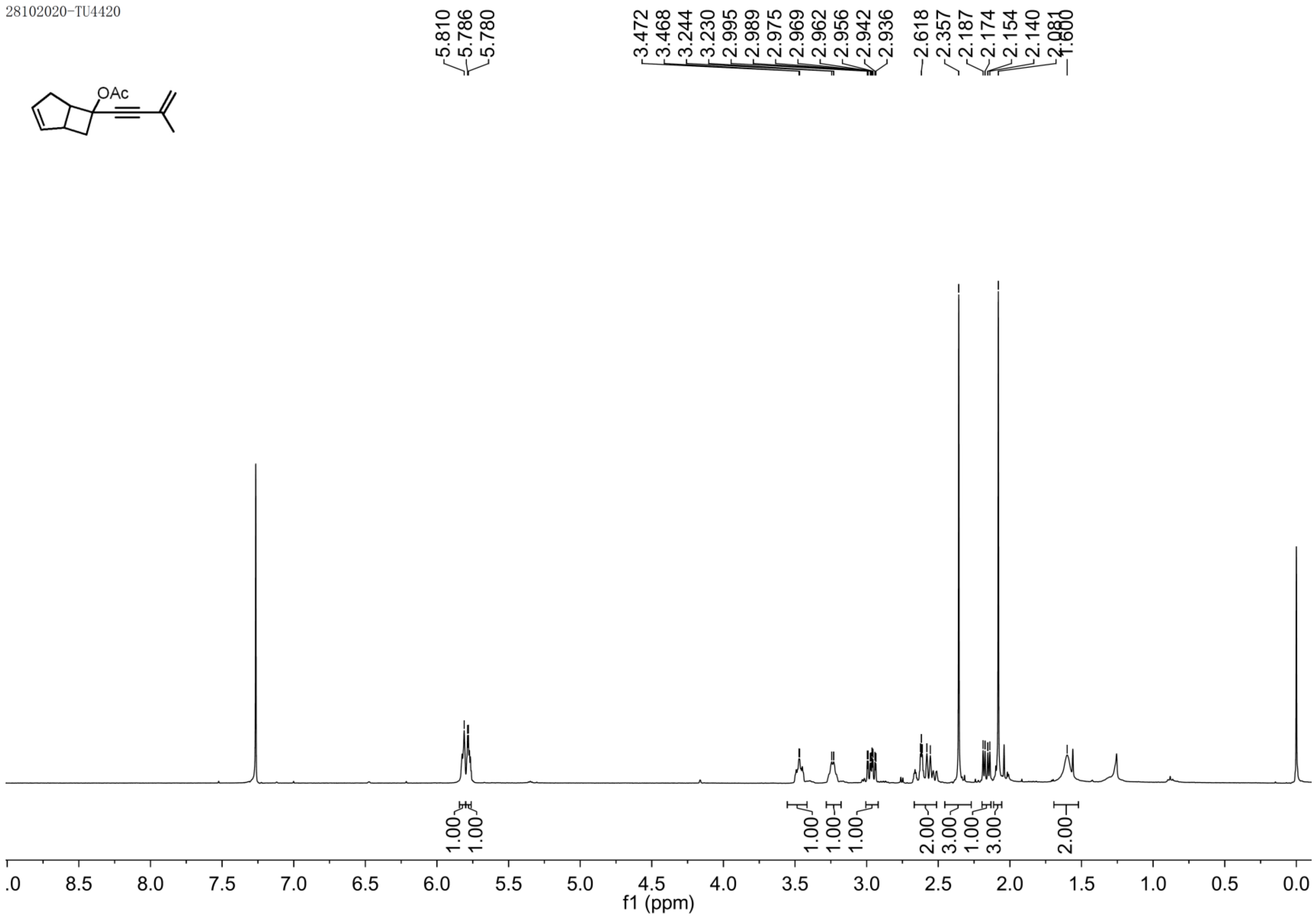

Supplementary Figure 29 ^{1}H NMR Spectrum of Compound **1g**

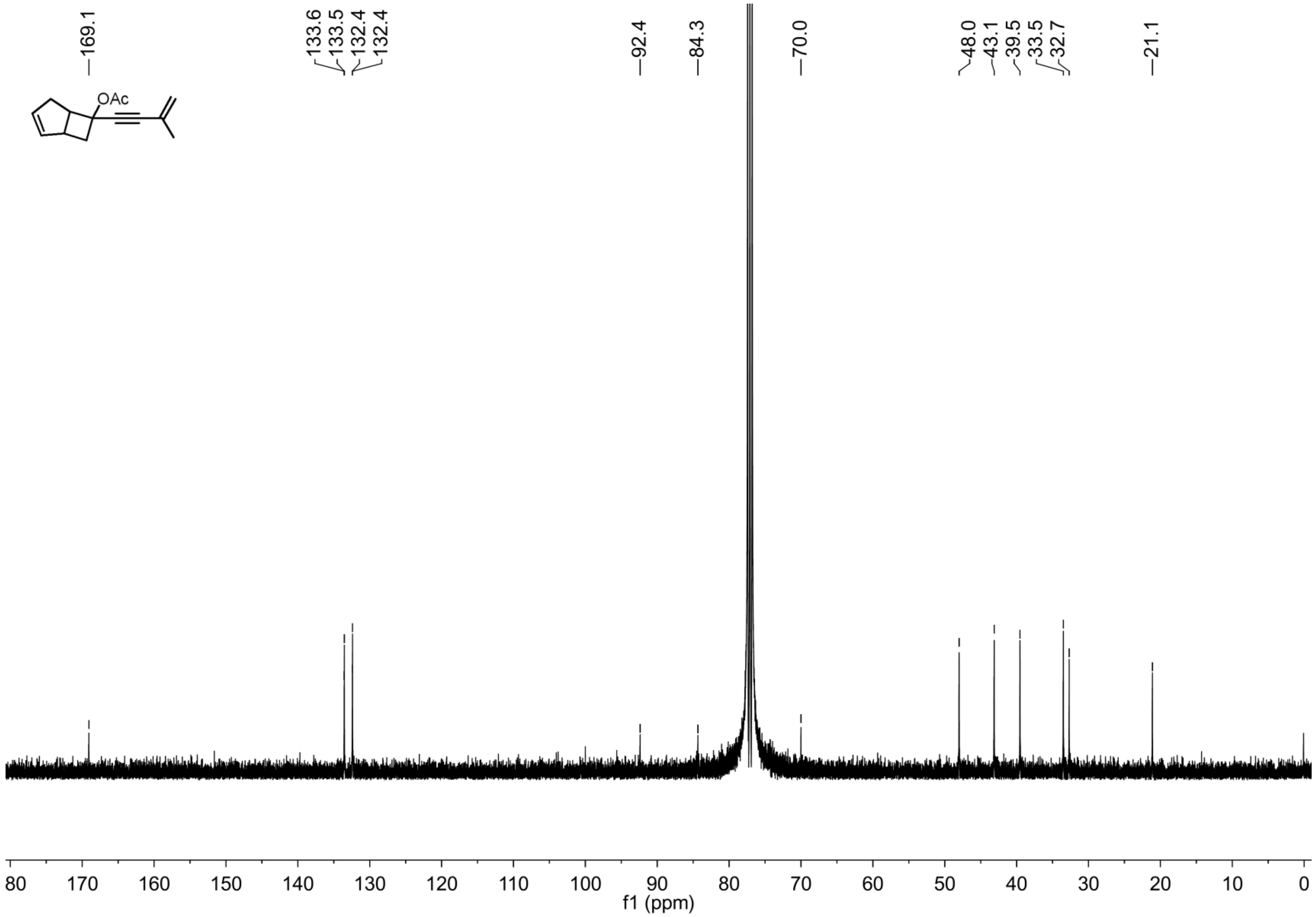

Supplementary Figure 30 ^{13}C NMR Spectrum of Compound **1g**

26112020-TU5390

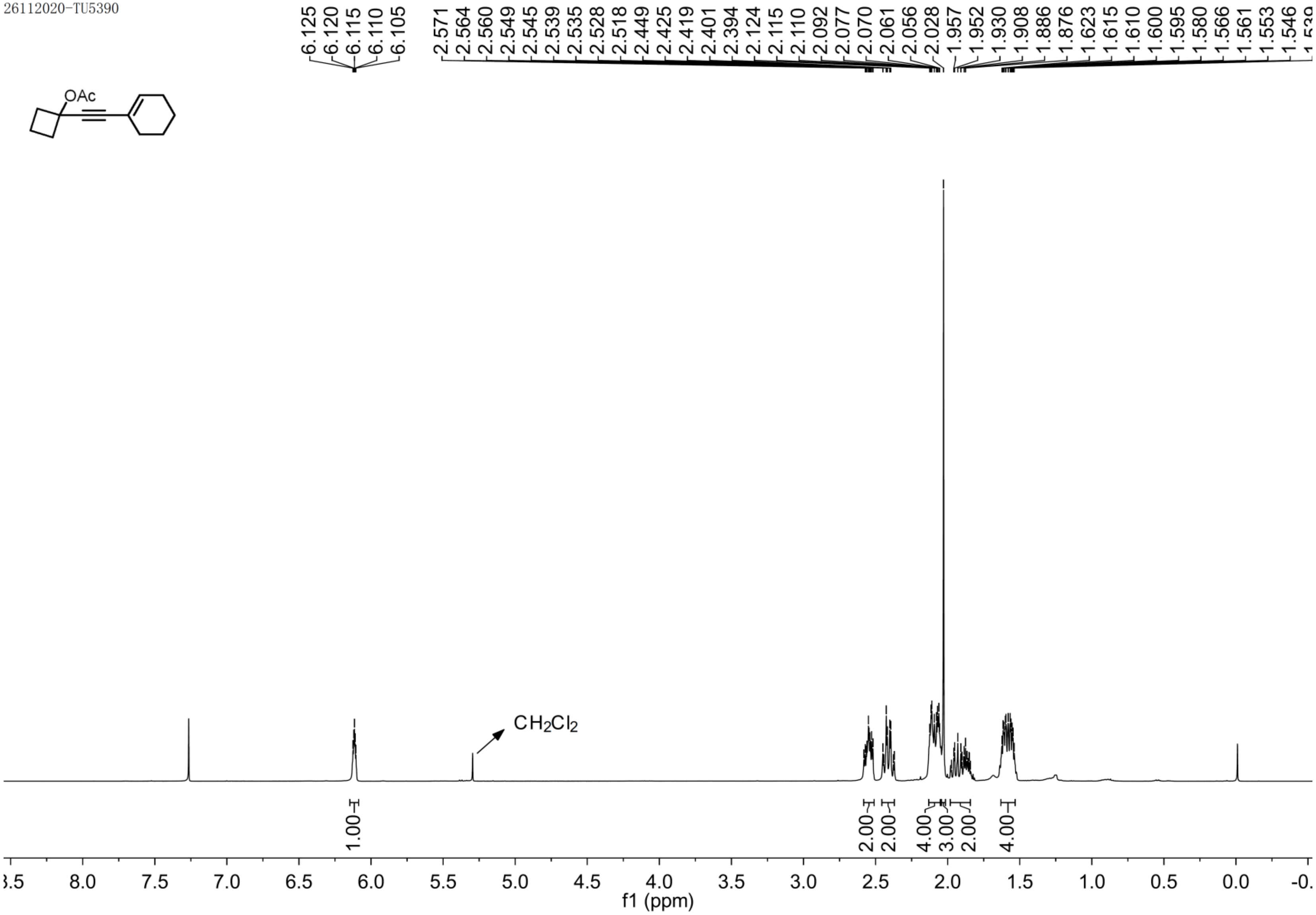

Supplementary Figure 31 ^{1}H NMR Spectrum of Compound **1h**

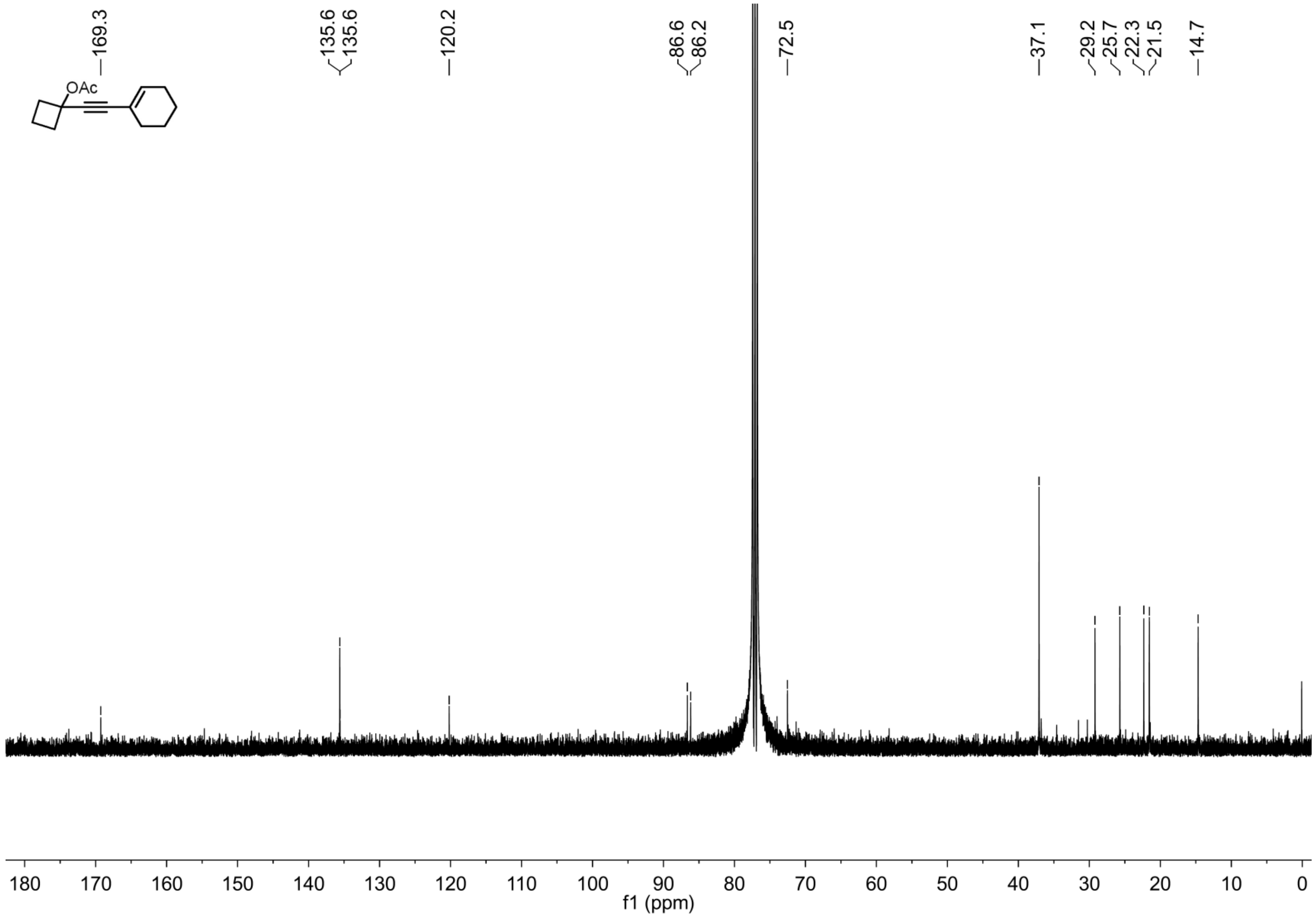

Supplementary Figure 32 ^{13}C NMR Spectrum of Compound **1h**

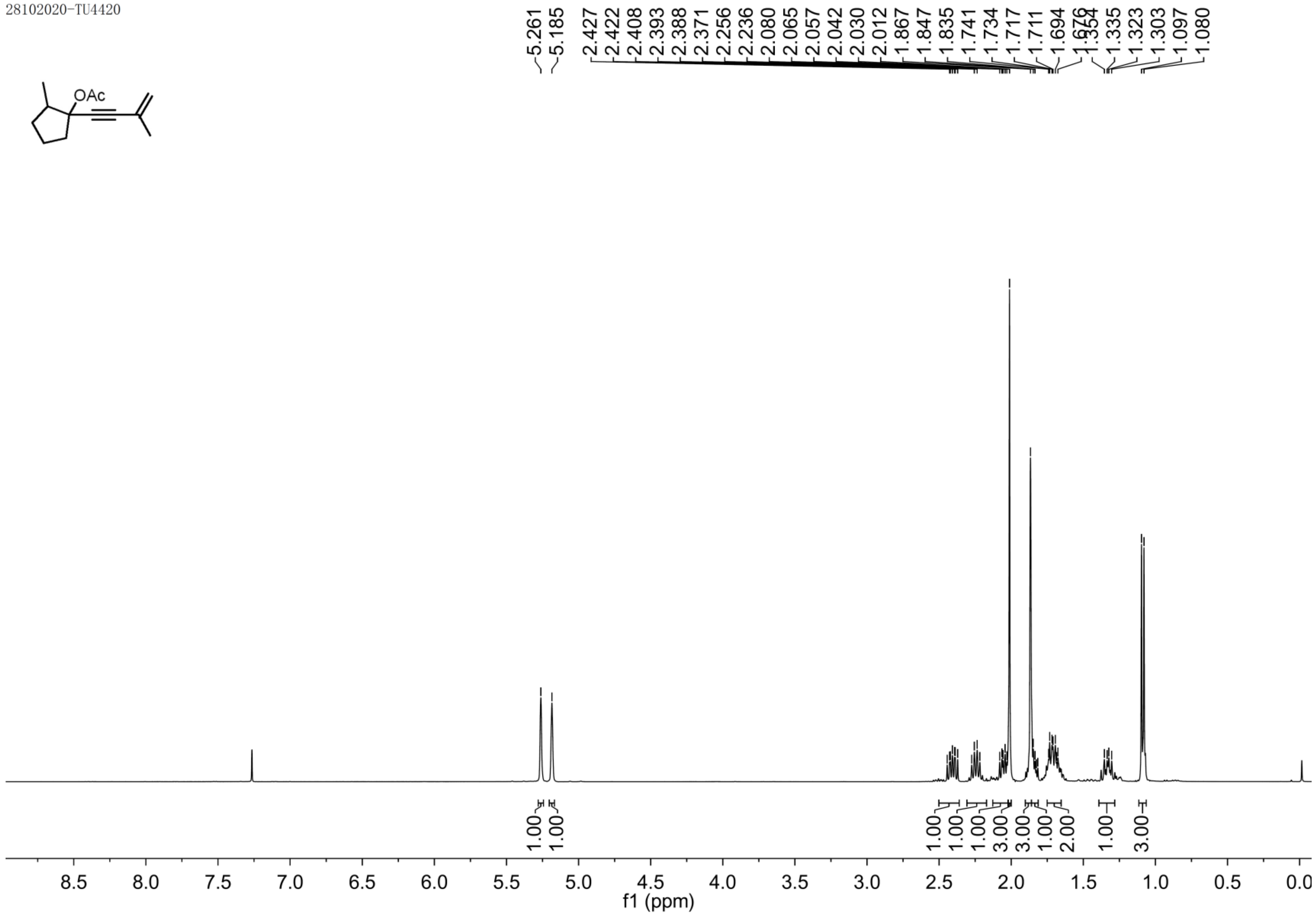

Supplementary Figure 33 ^{1}H NMR Spectrum of Compound **1i**

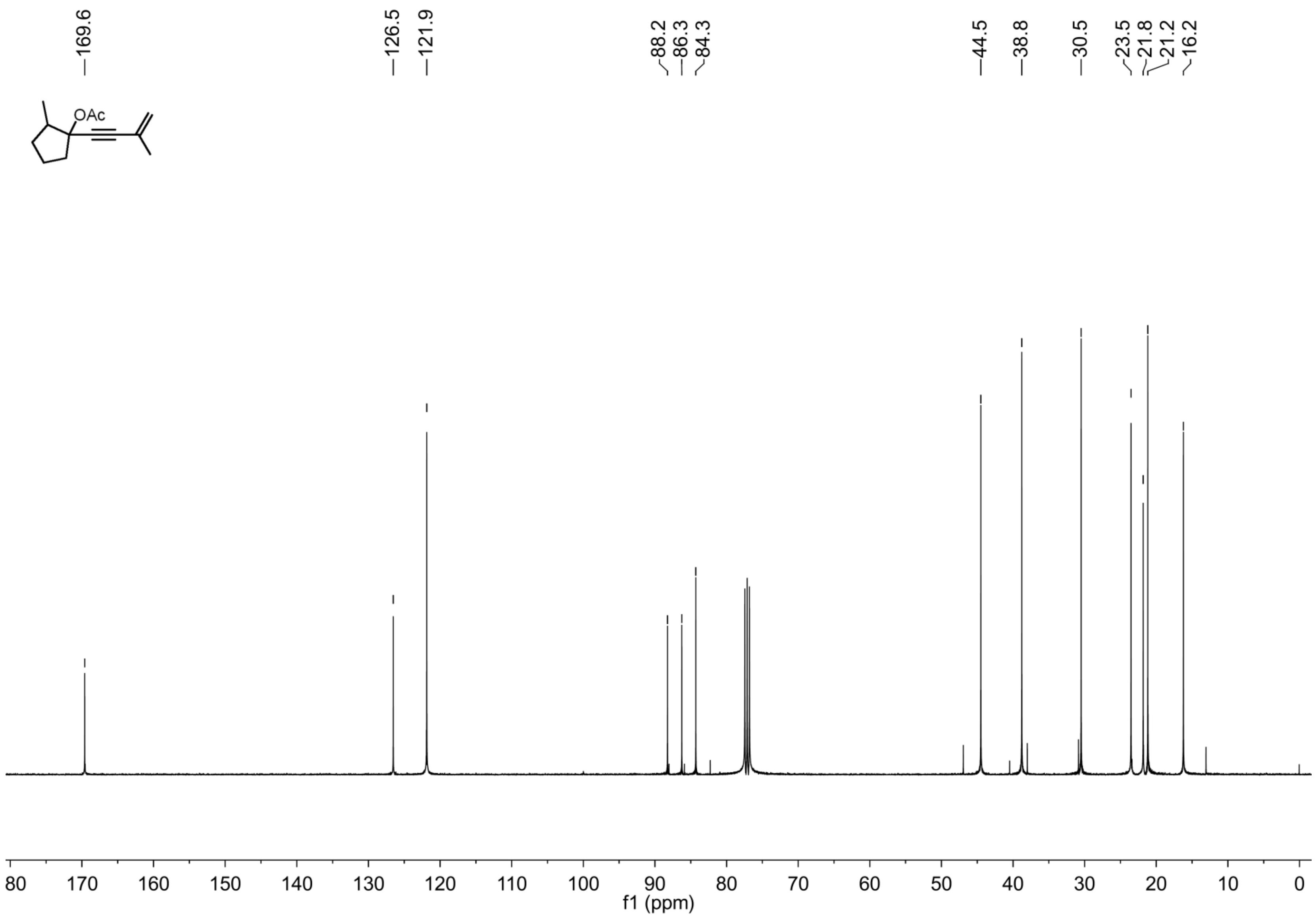

Supplementary Figure 34 ^{13}C NMR Spectrum of Compound **1i**

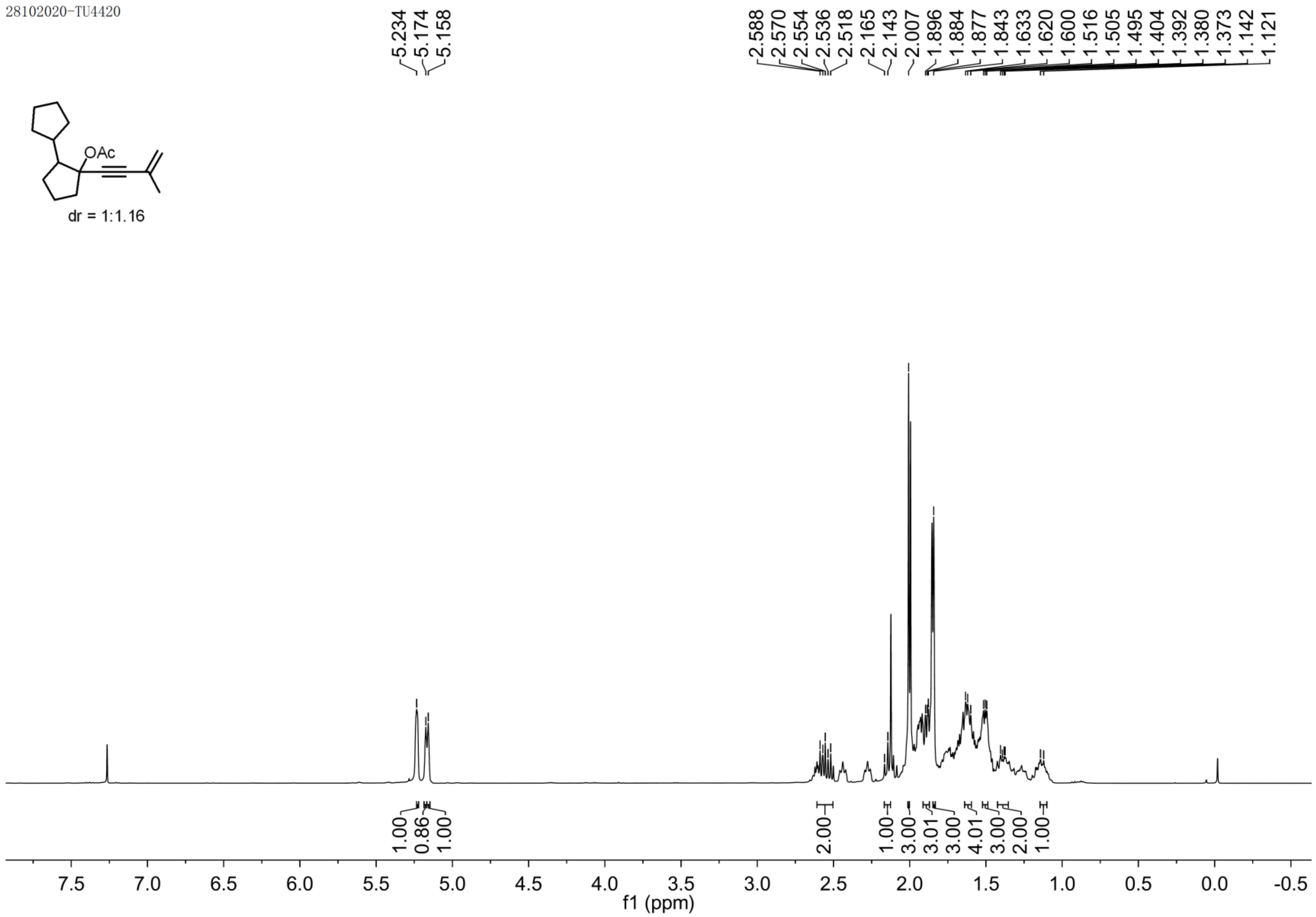

Supplementary Figure 35 ¹H NMR Spectrum of Compound **1j**

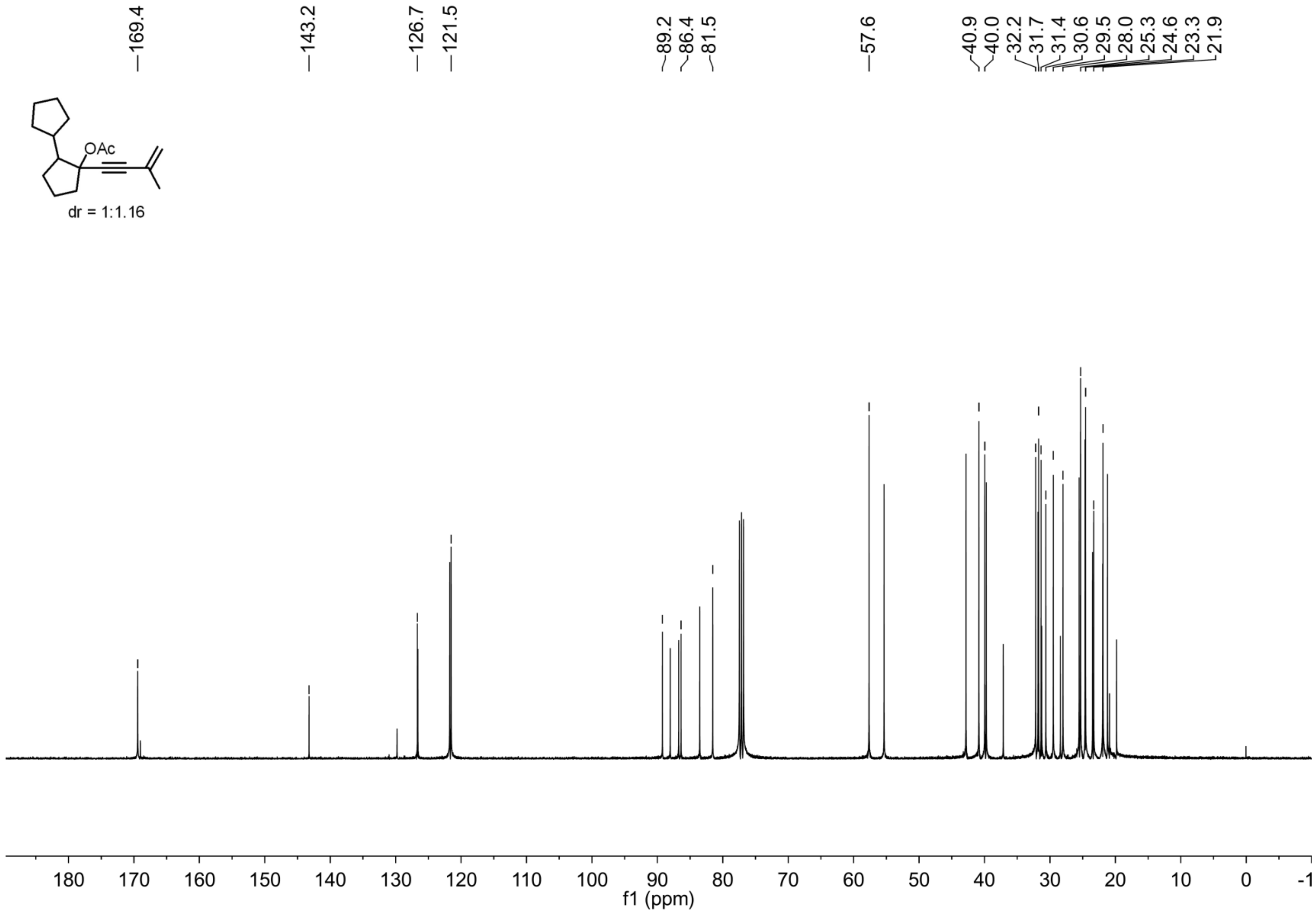

Supplementary Figure 36 ^{13}C NMR Spectrum of Compound **1j**

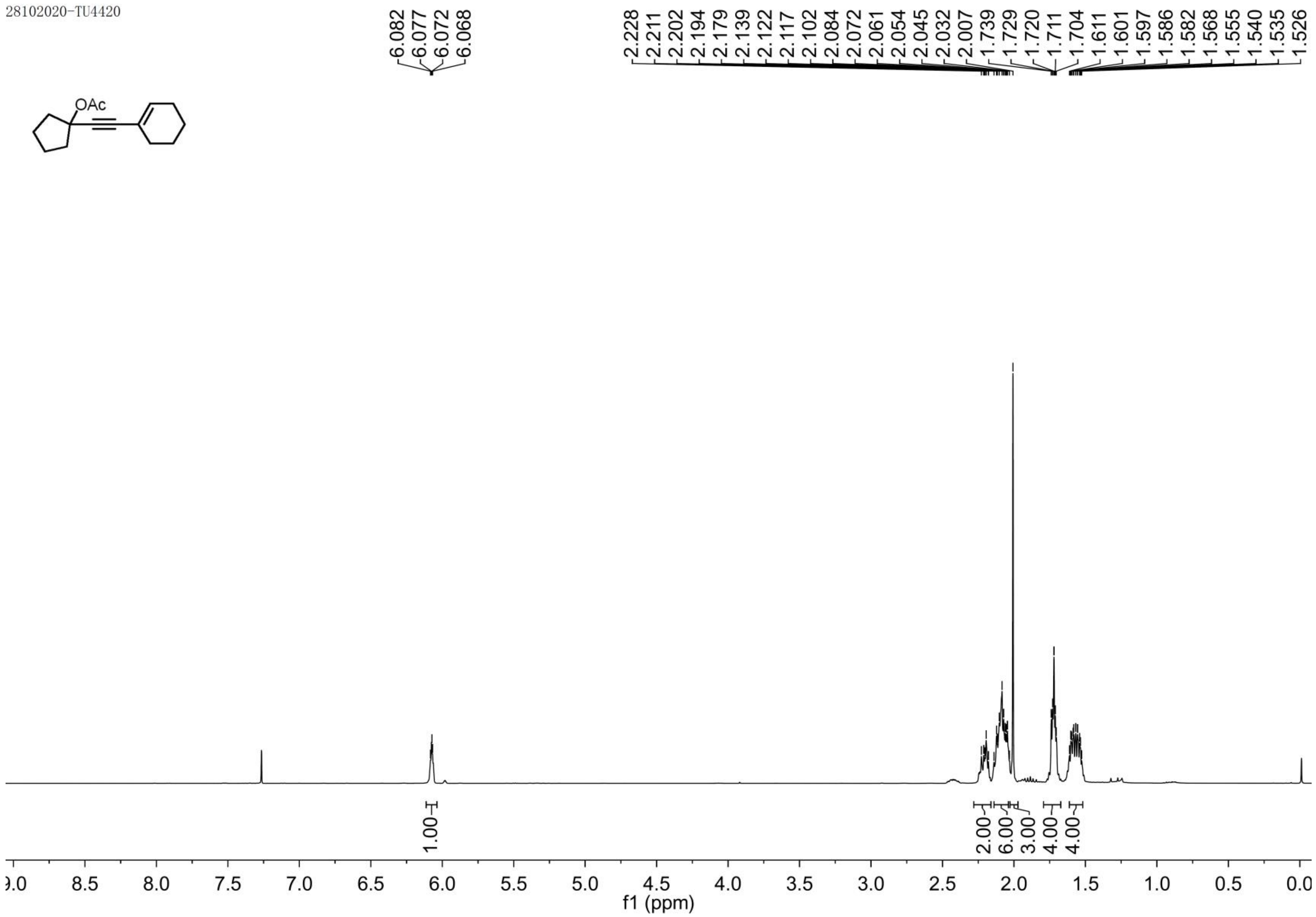

Supplementary Figure 37 ^{1}H NMR Spectrum of Compound **1k**

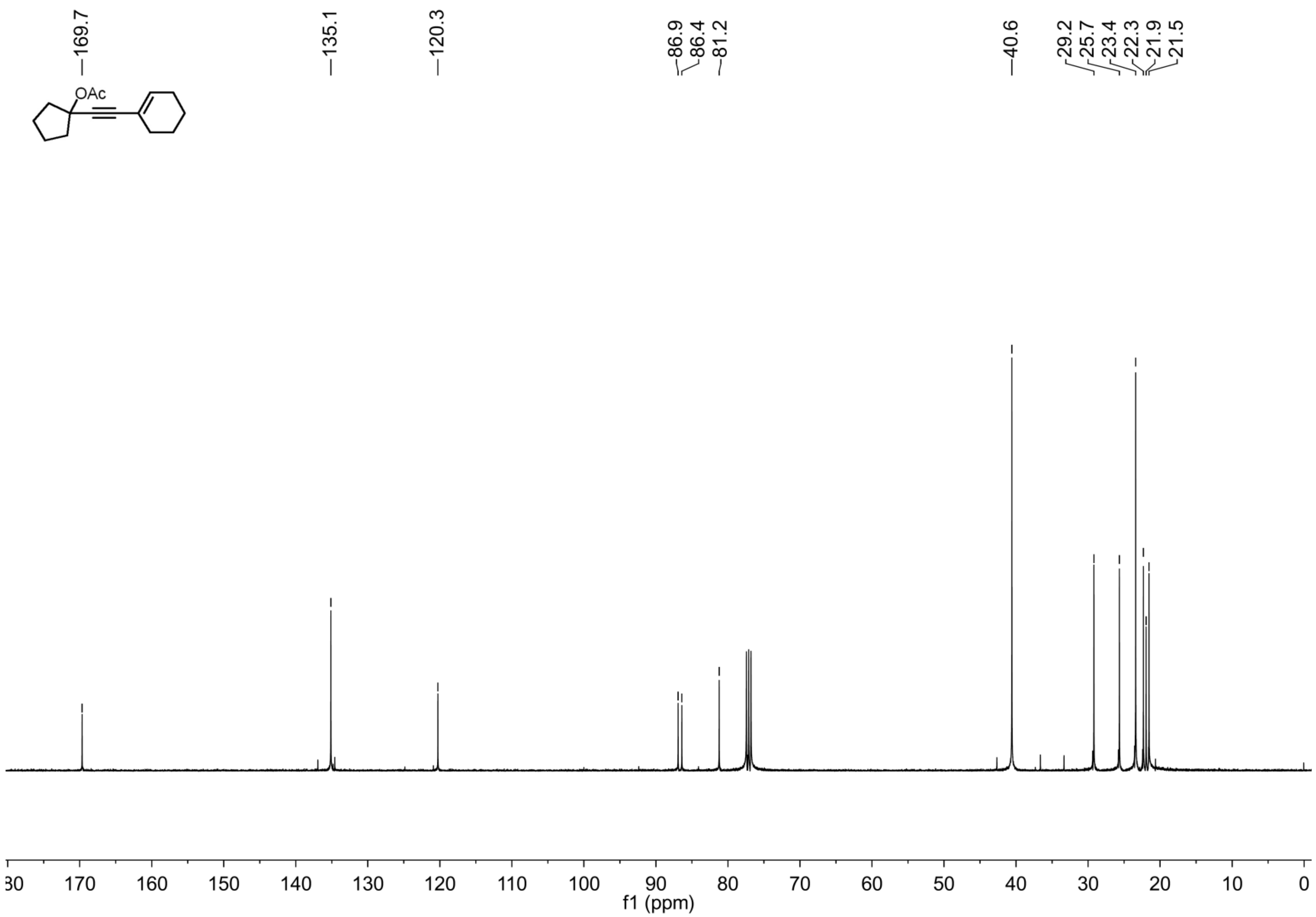

Supplementary Figure 38 ^{13}C NMR Spectrum of Compound **1k**

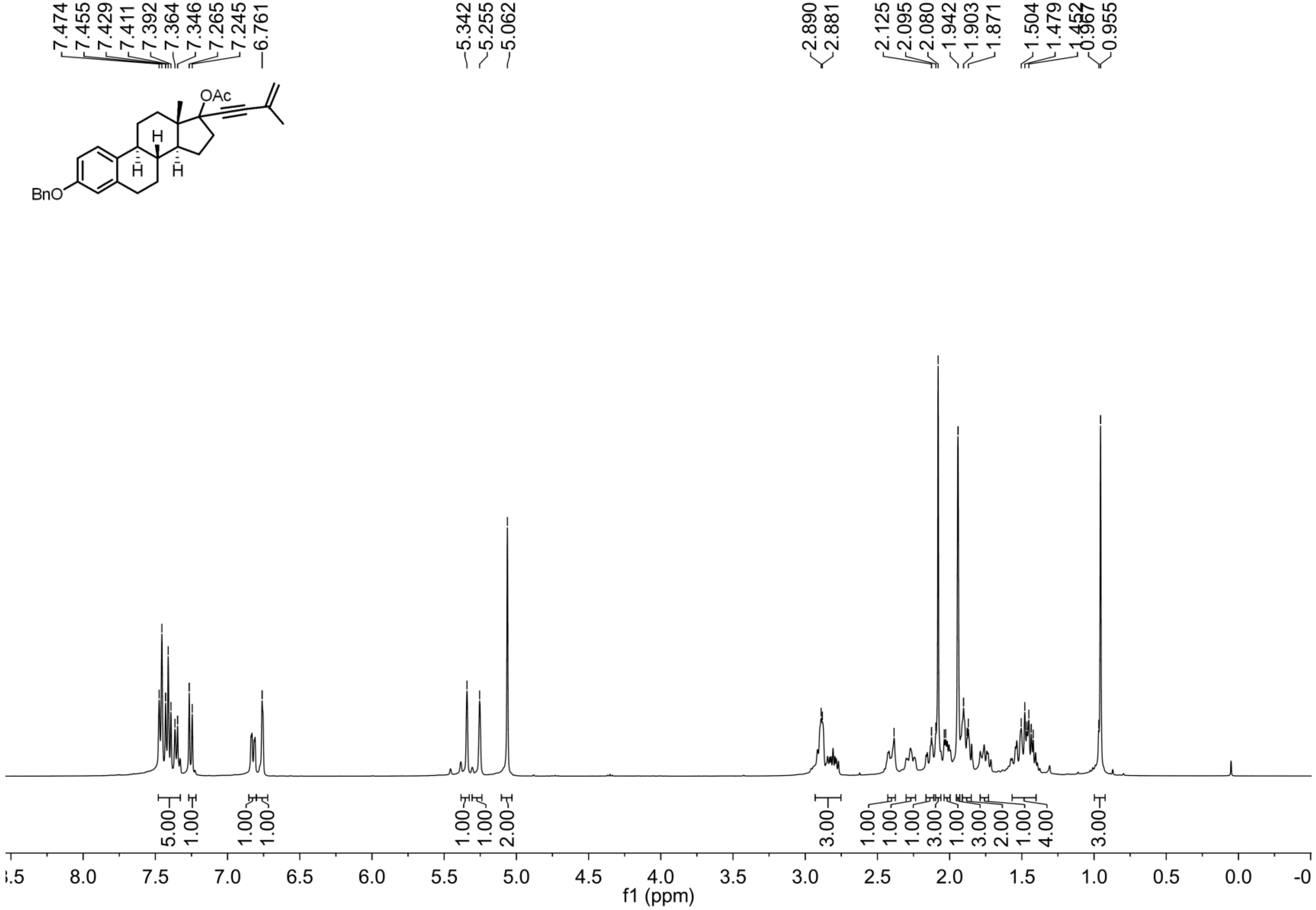

Supplementary Figure 39 ^{1}H NMR Spectrum of Compound **1l**

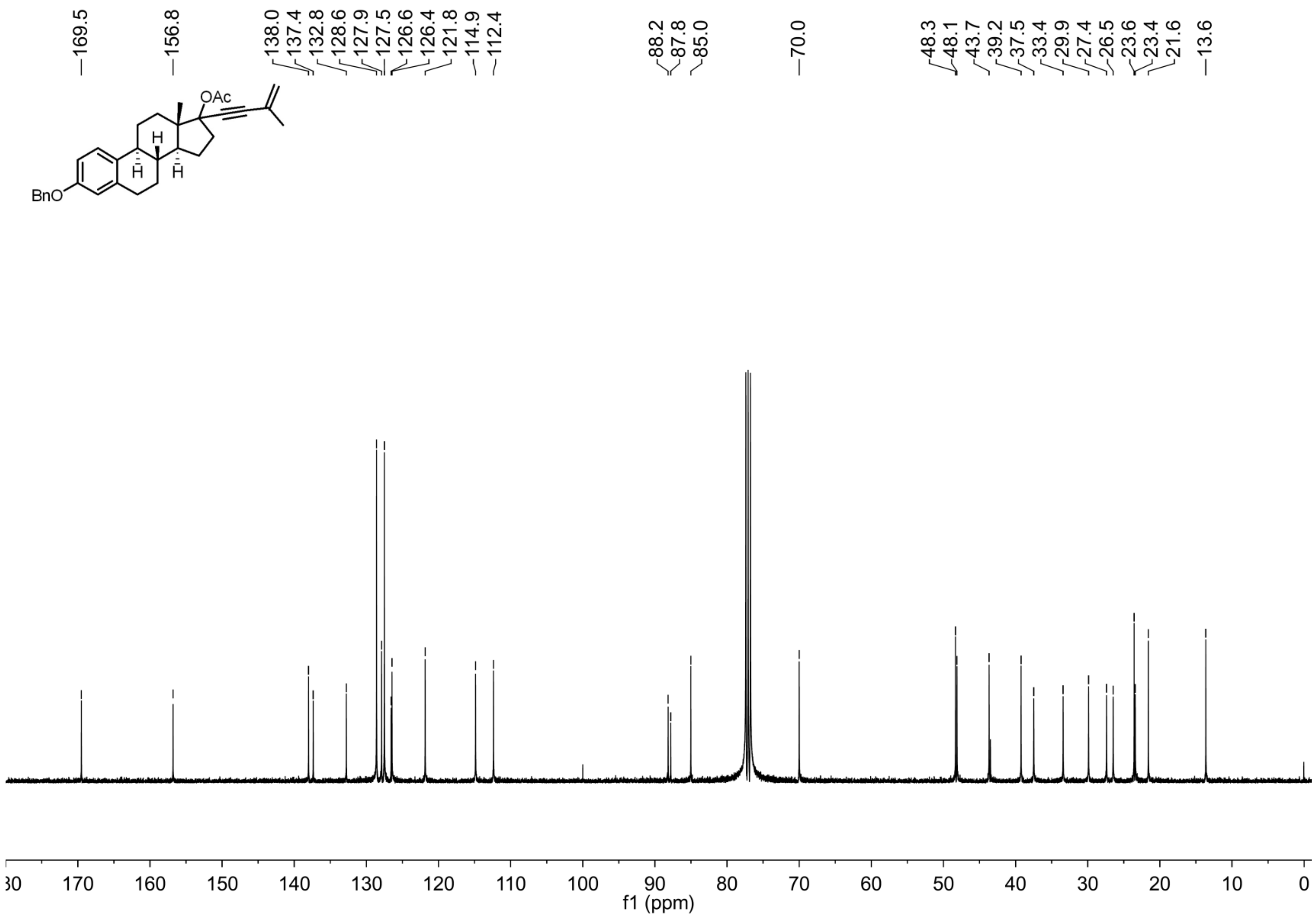

Supplementary Figure 40 ^{13}C NMR Spectrum of Compound **1l**

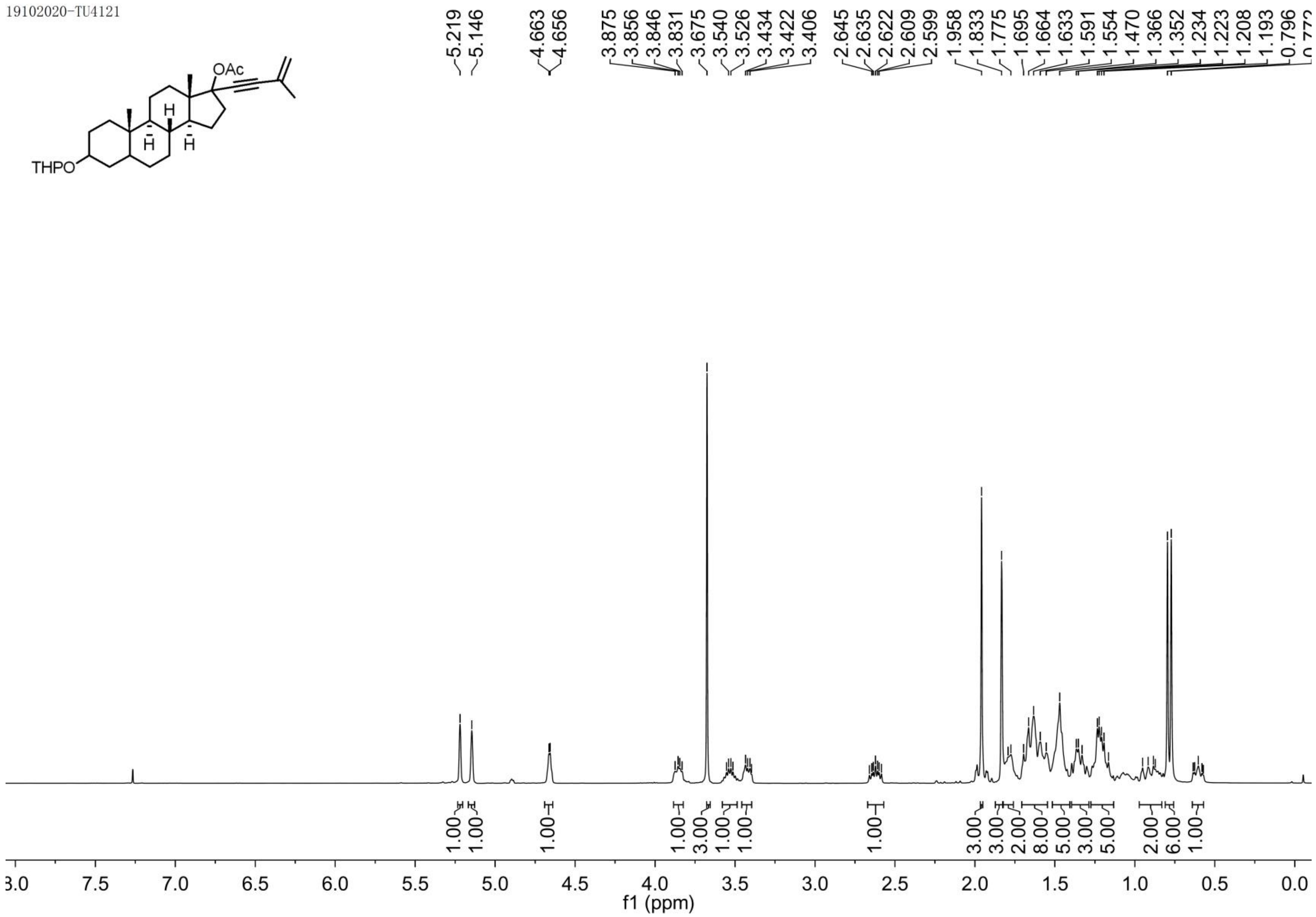

Supplementary Figure 41 ^{1}H NMR Spectrum of Compound **1m**

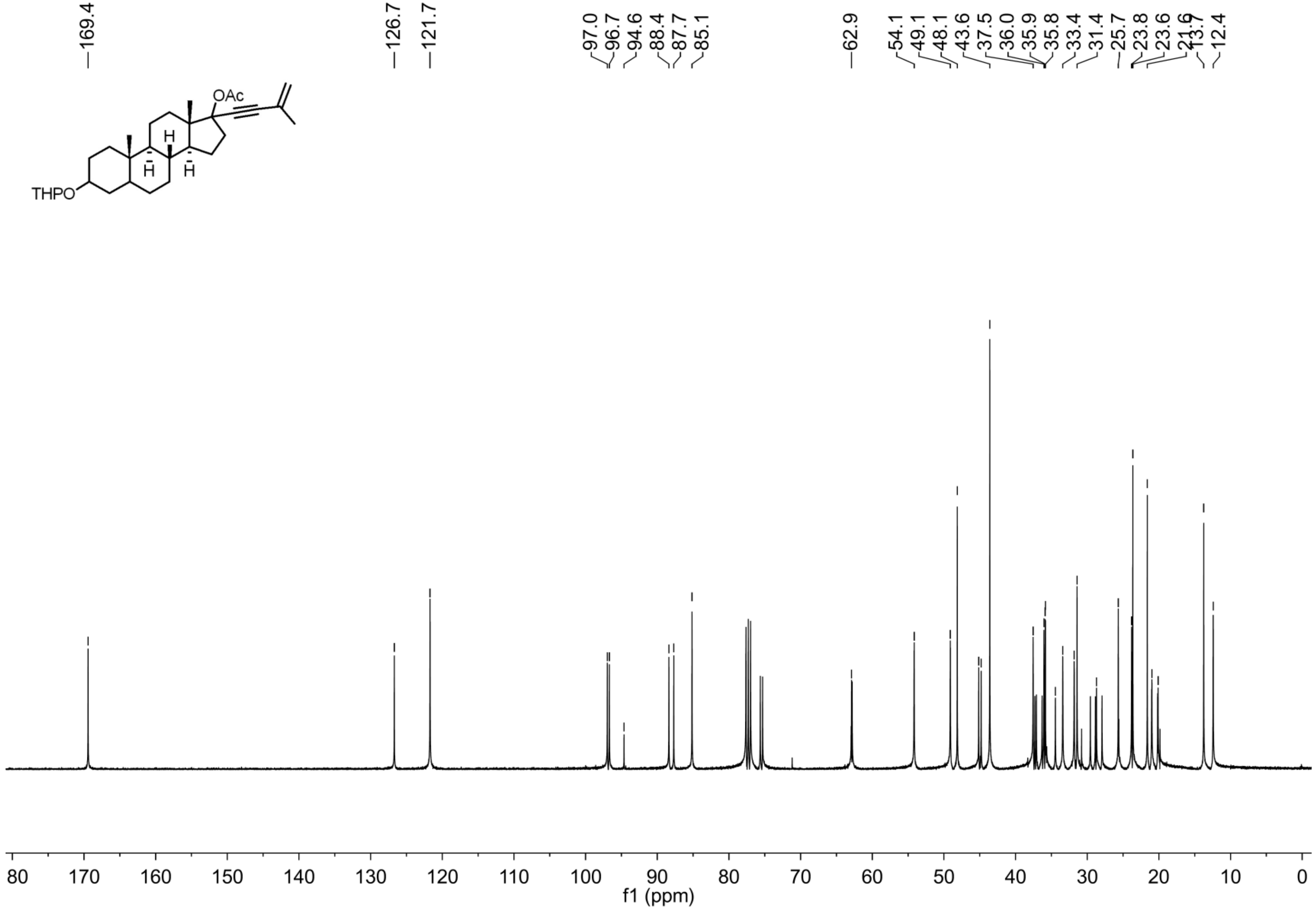

Supplementary Figure 42 ^{13}C NMR Spectrum of Compound **1m**

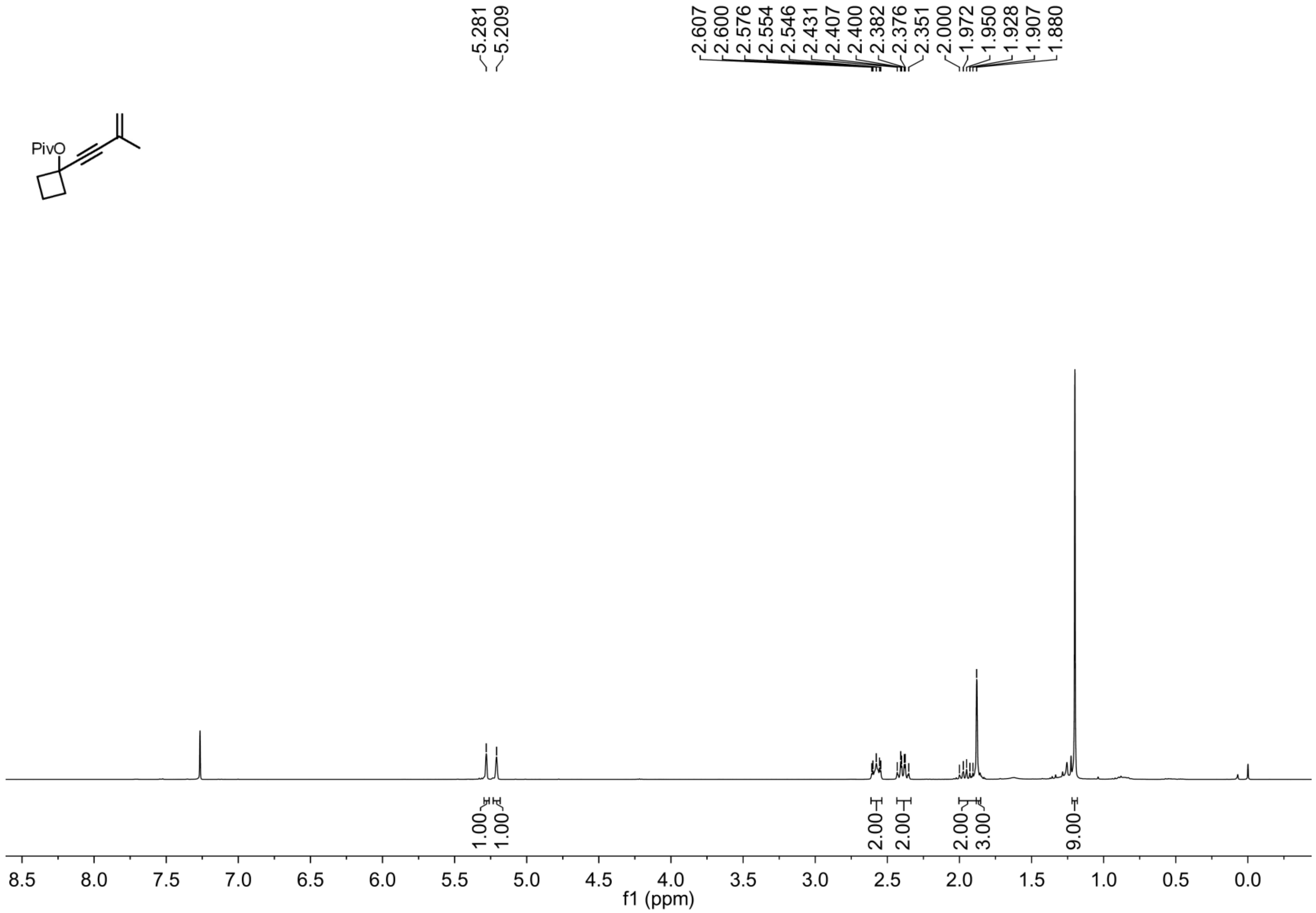

Supplementary Figure 43 ^{1}H NMR Spectrum of Compound **1ae**

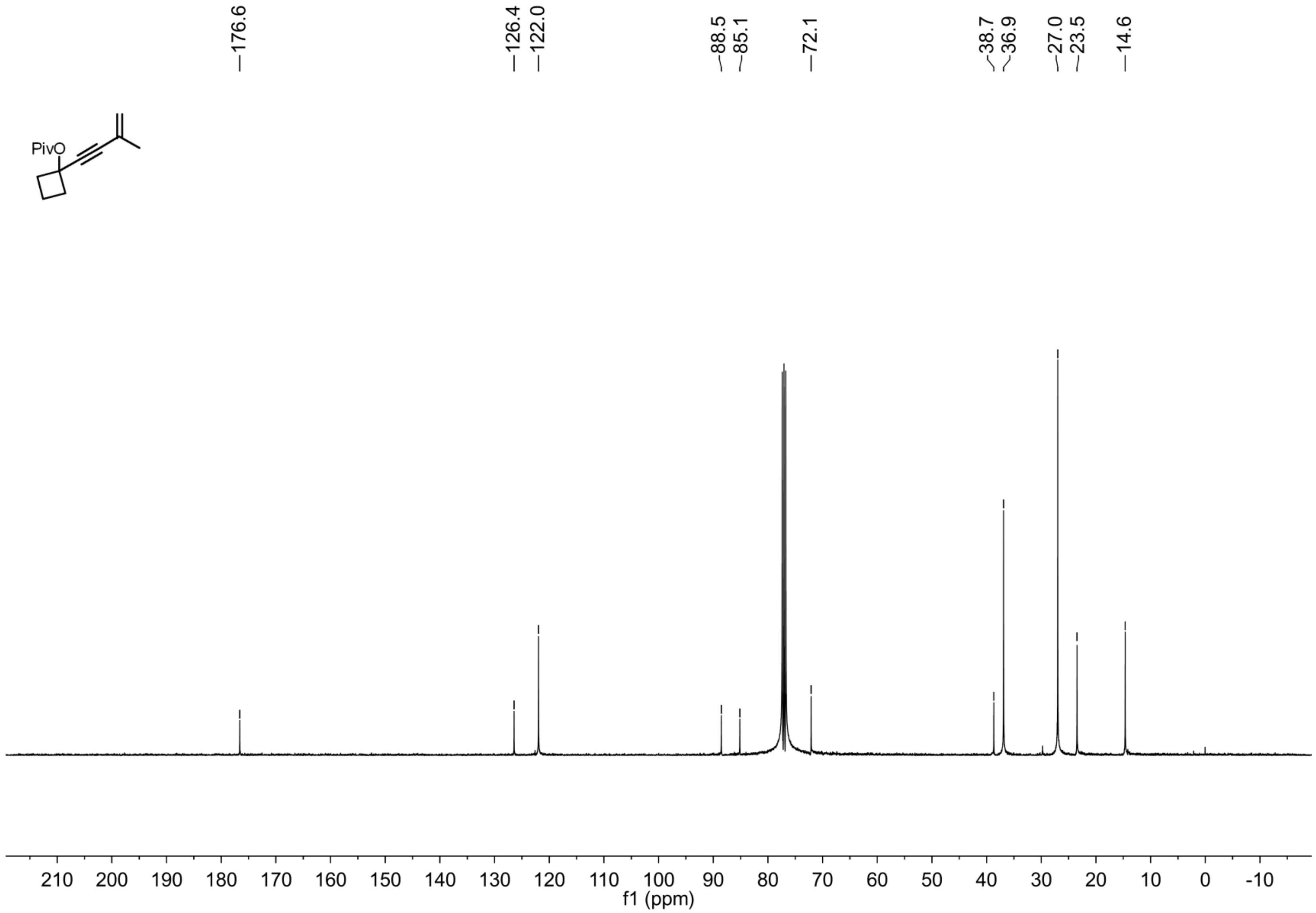

Supplementary Figure 44 ^{13}C NMR Spectrum of Compound **1ae**

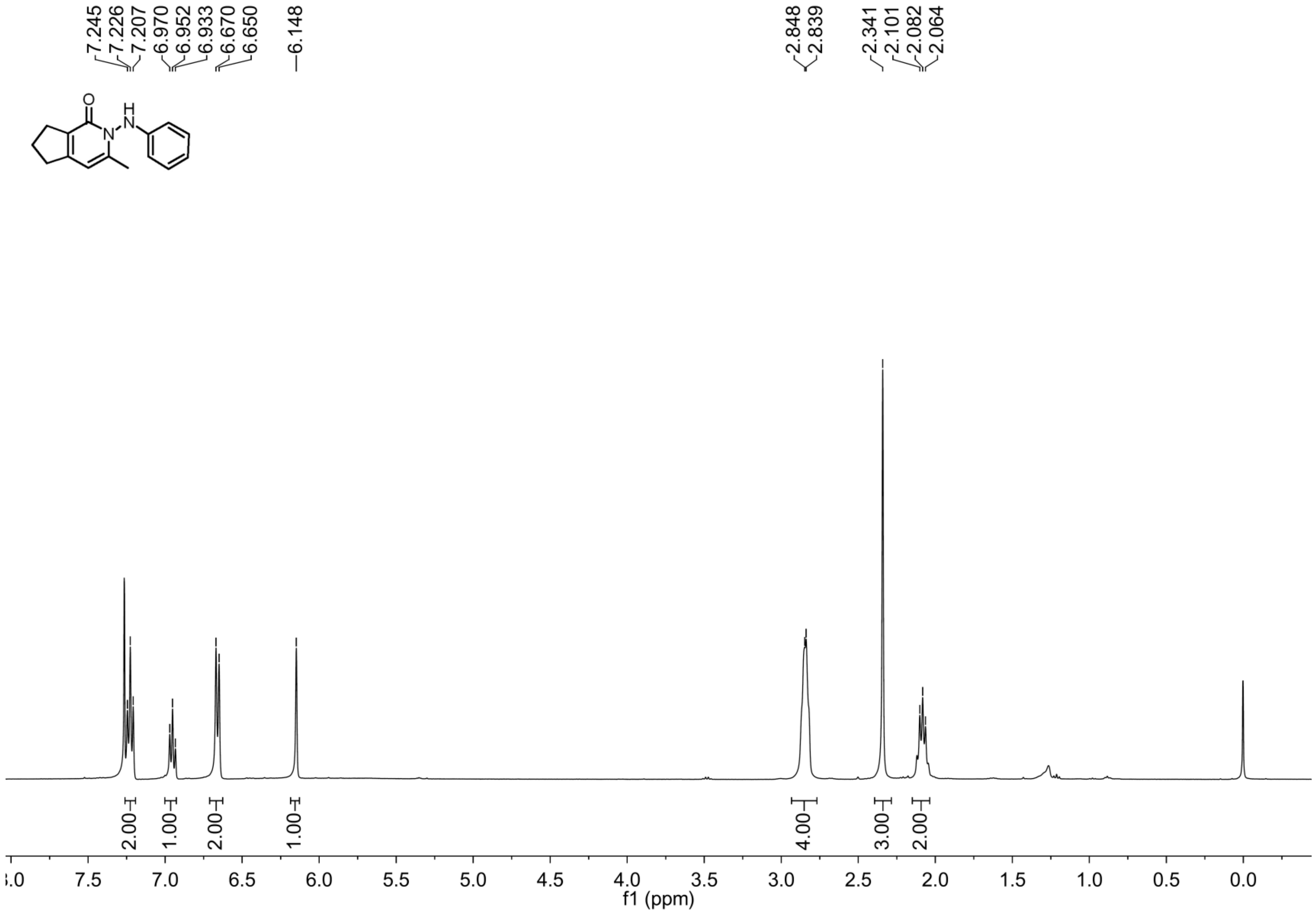

Supplementary Figure 45 ^{1}H NMR Spectrum of Compound **3**

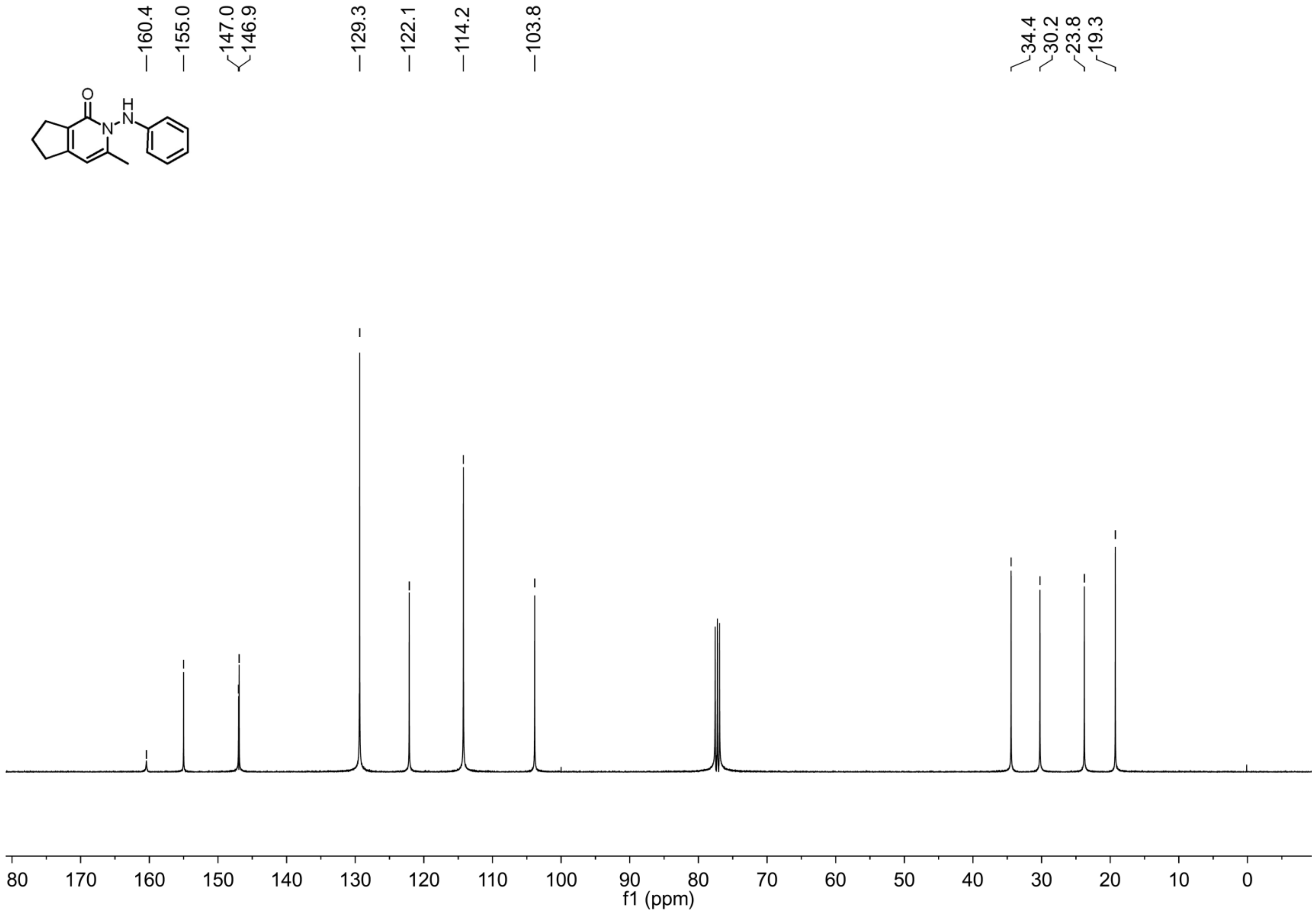

Supplementary Figure 46 ^{13}C NMR Spectrum of Compound **3**

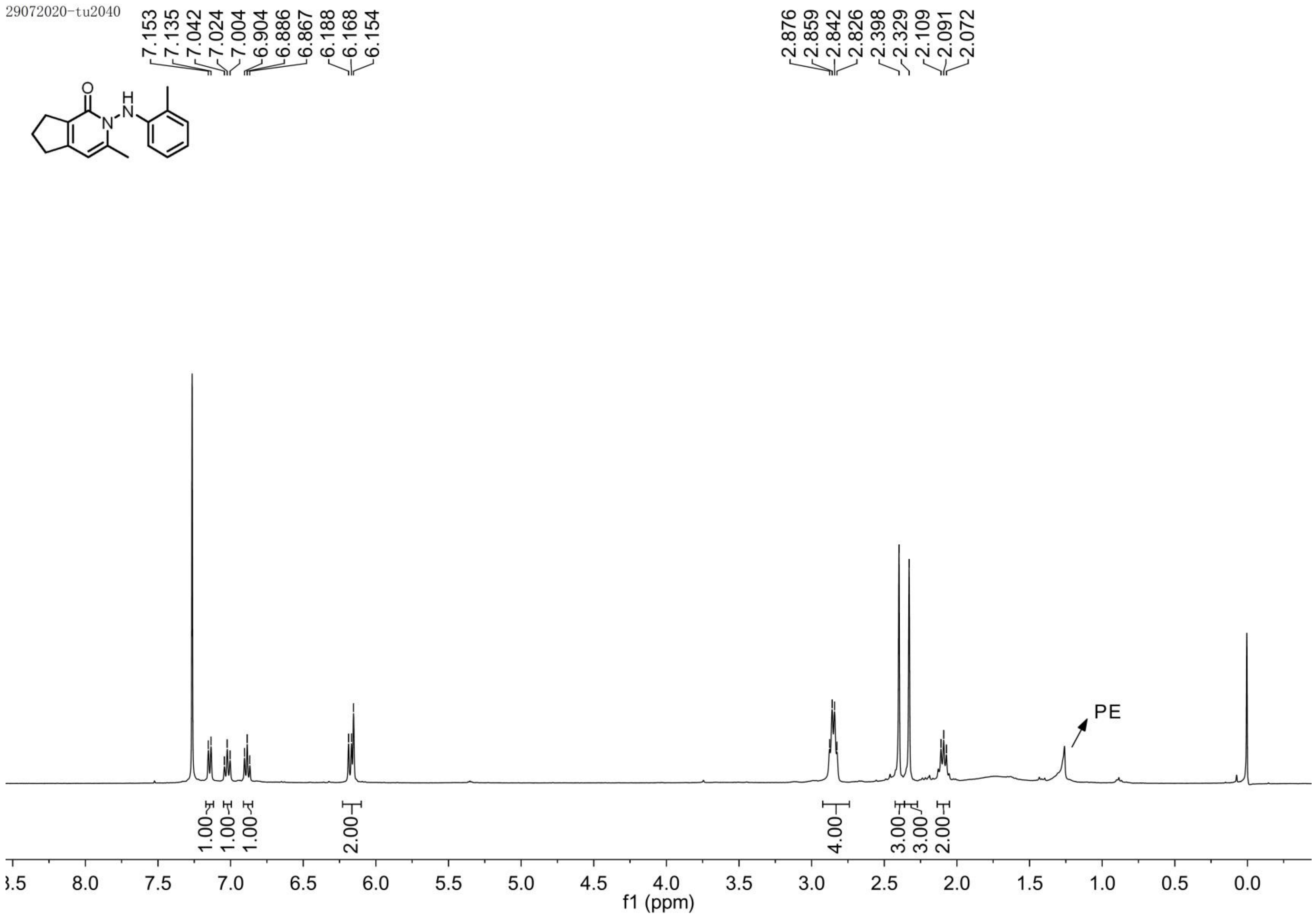

Supplementary Figure 47 ^{1}H NMR Spectrum of Compound **4**

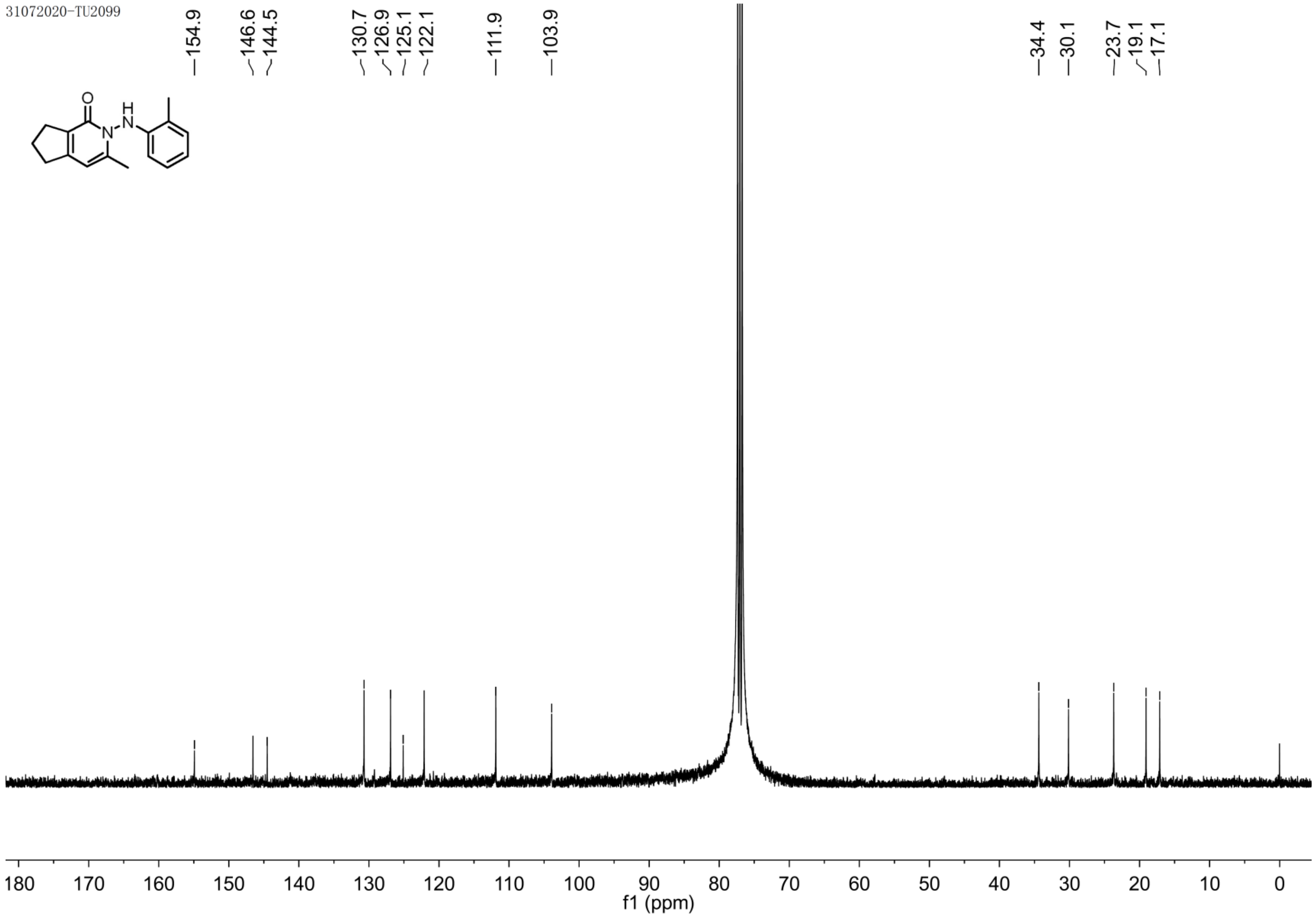

Supplementary Figure 48 ^{13}C NMR Spectrum of Compound **4**

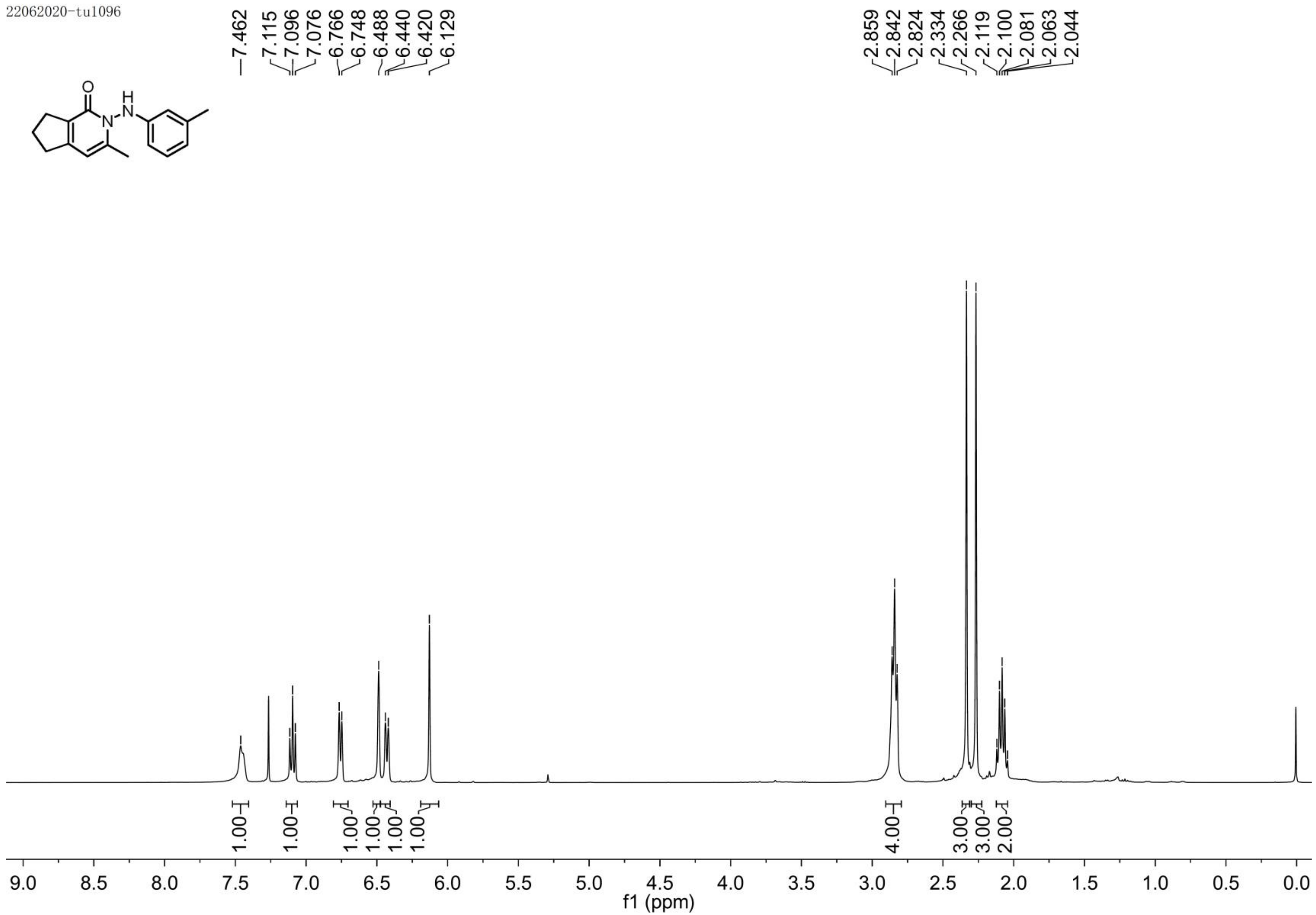

Supplementary Figure 49 ^{1}H NMR Spectrum of Compound **5**

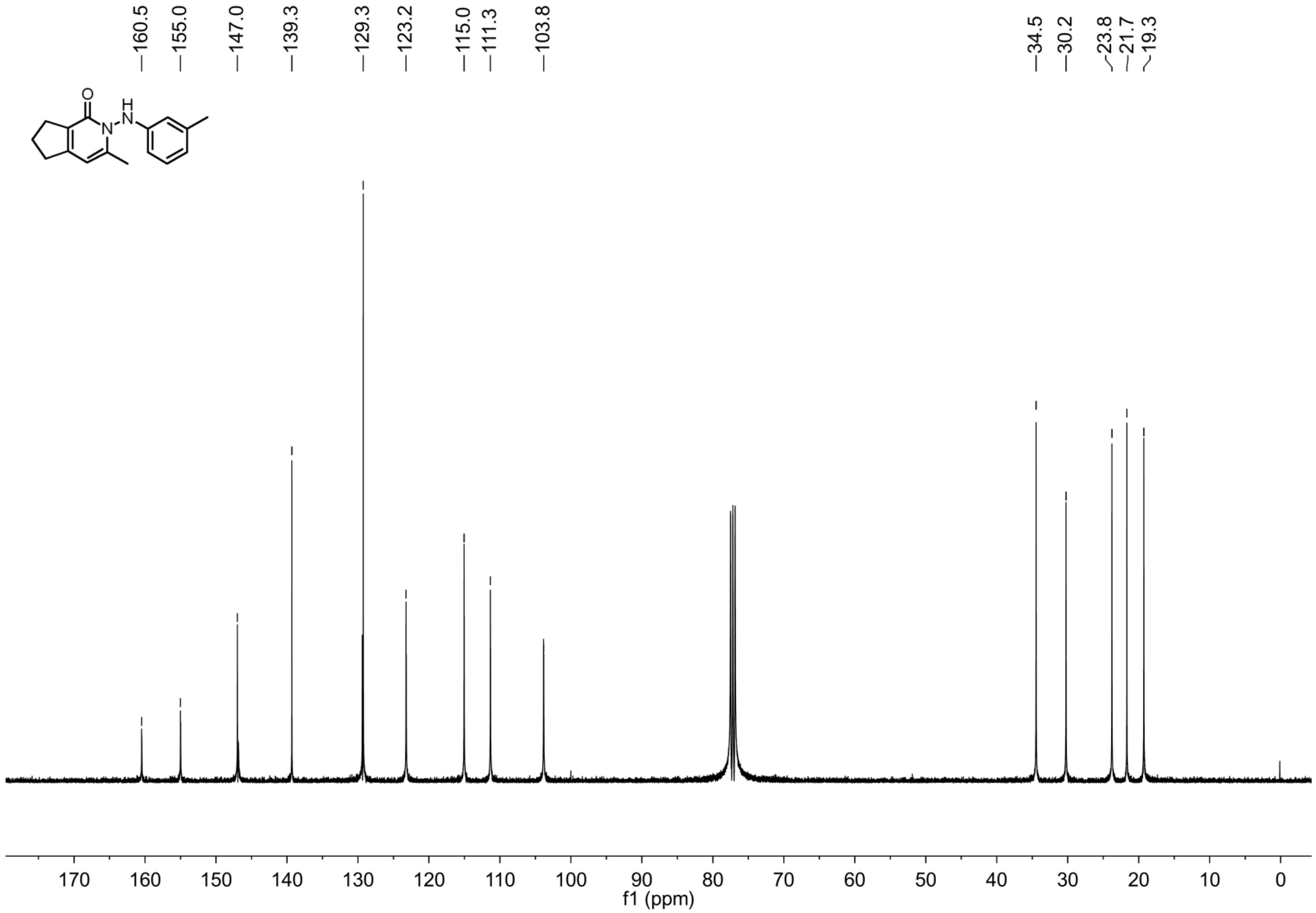

Supplementary Figure 50 ^{13}C NMR Spectrum of Compound **5**

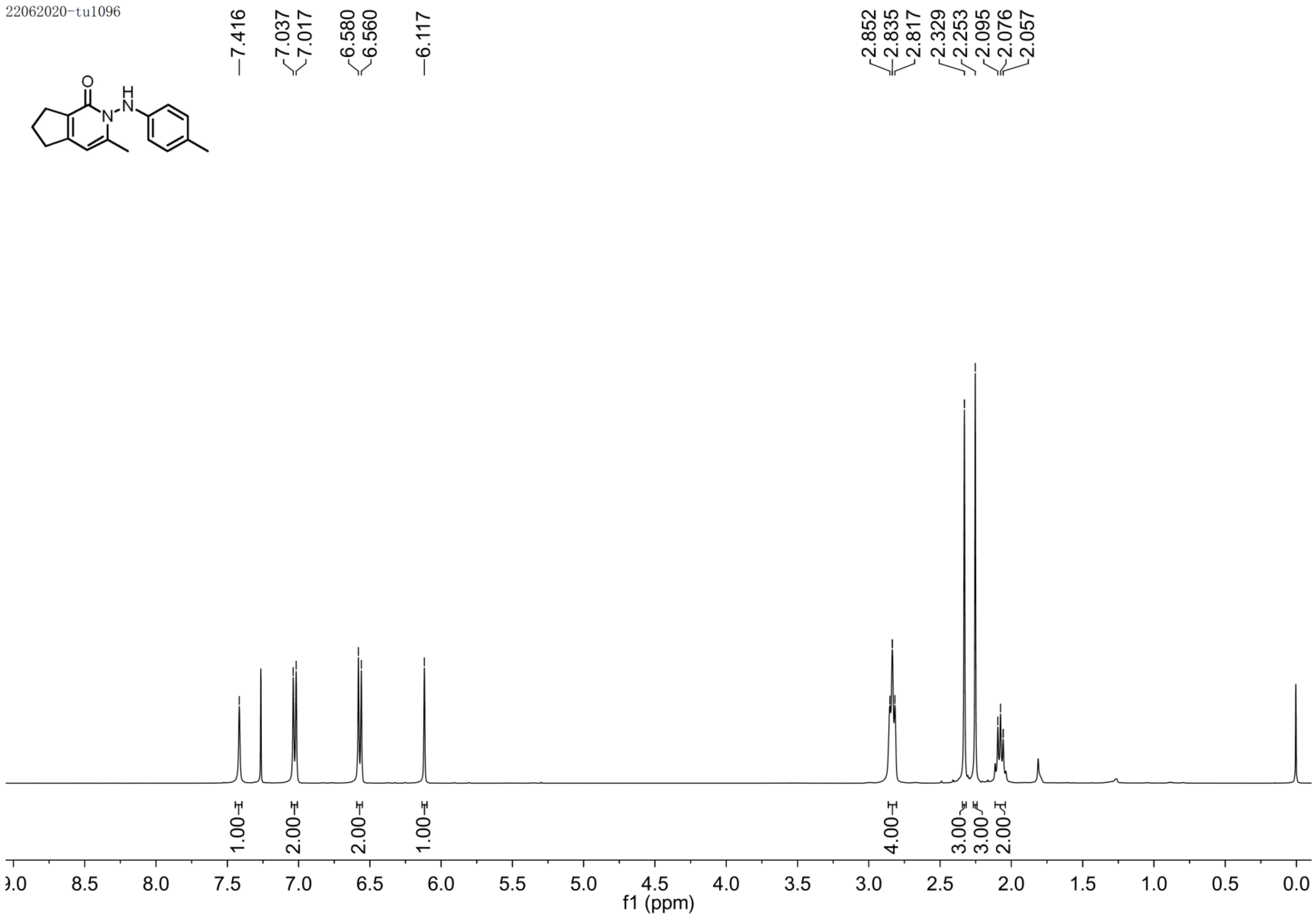

Supplementary Figure 51 ^{1}H NMR Spectrum of Compound **6**

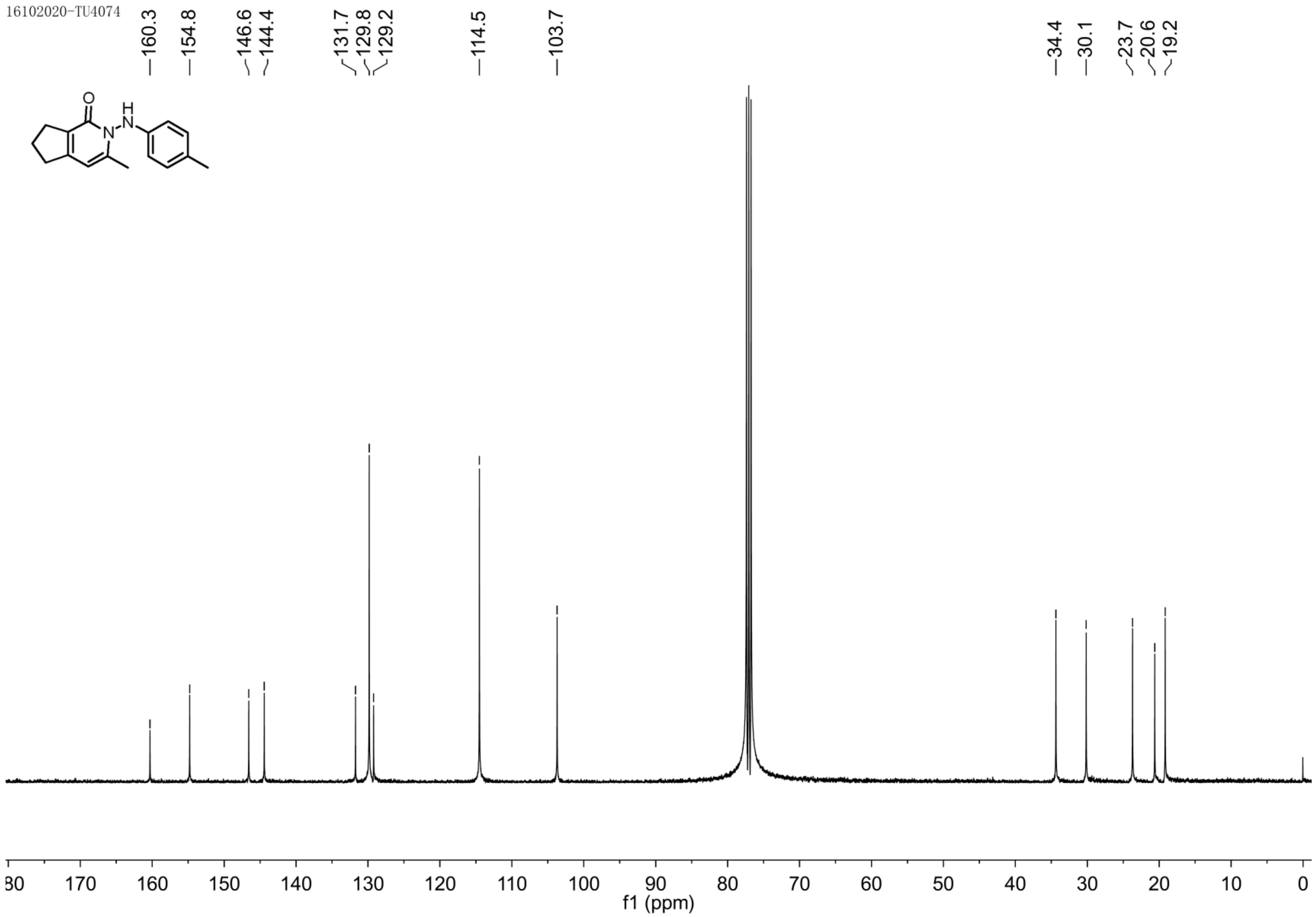

Supplementary Figure 52 ^{13}C NMR Spectrum of Compound **6**

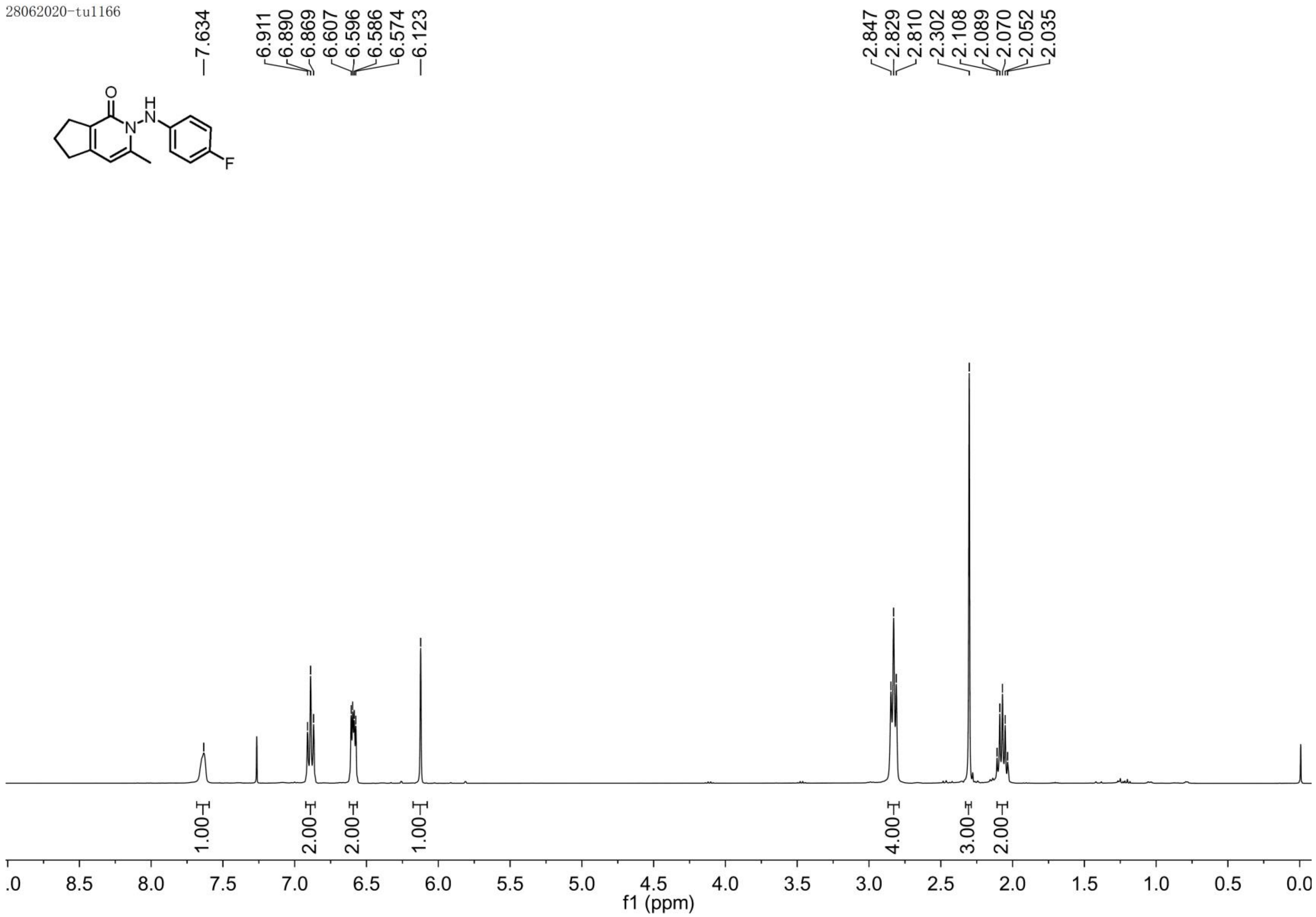

Supplementary Figure 53 ^{1}H NMR Spectrum of Compound **7**

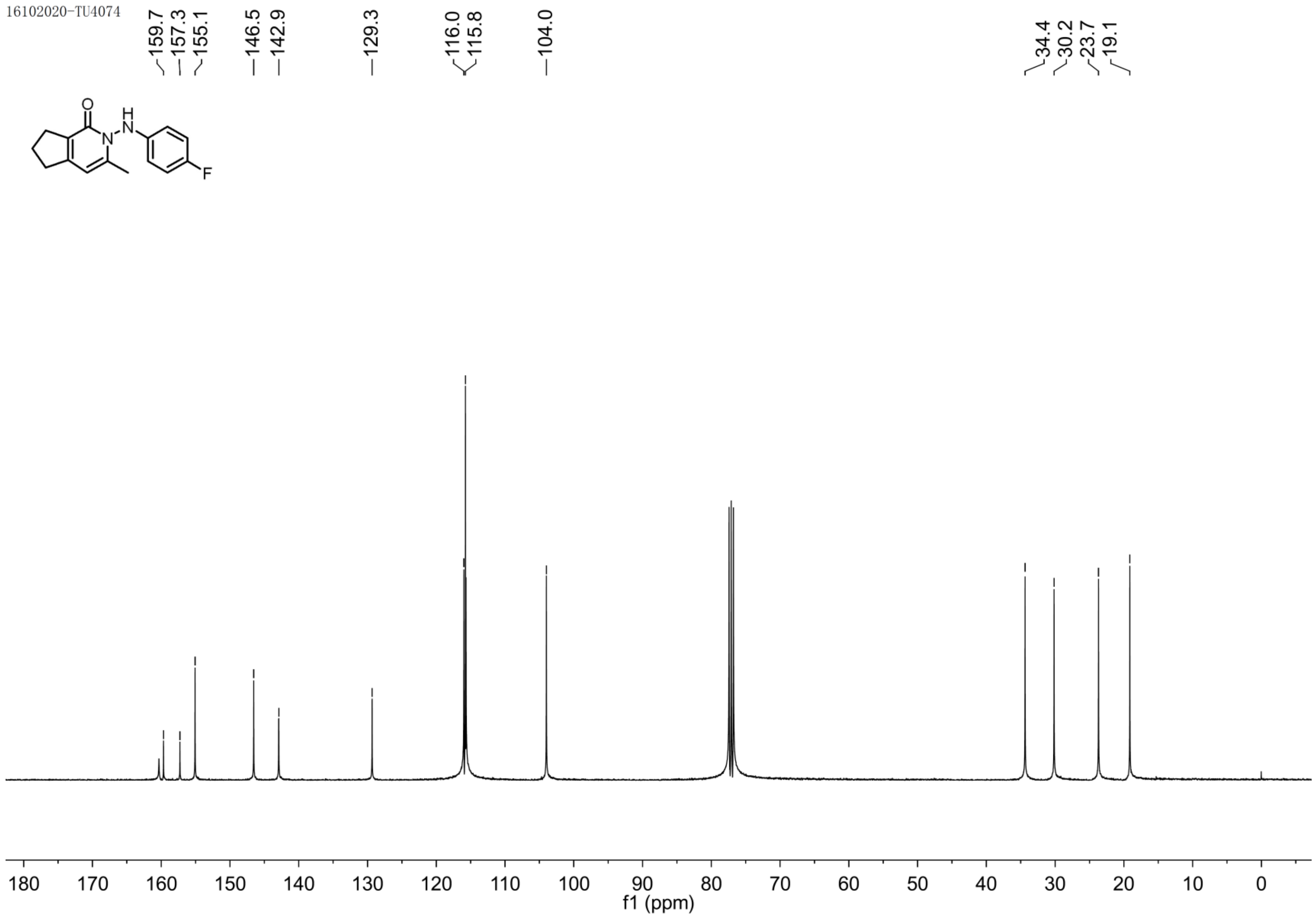

Supplementary Figure 54 ^{13}C NMR Spectrum of Compound **7**

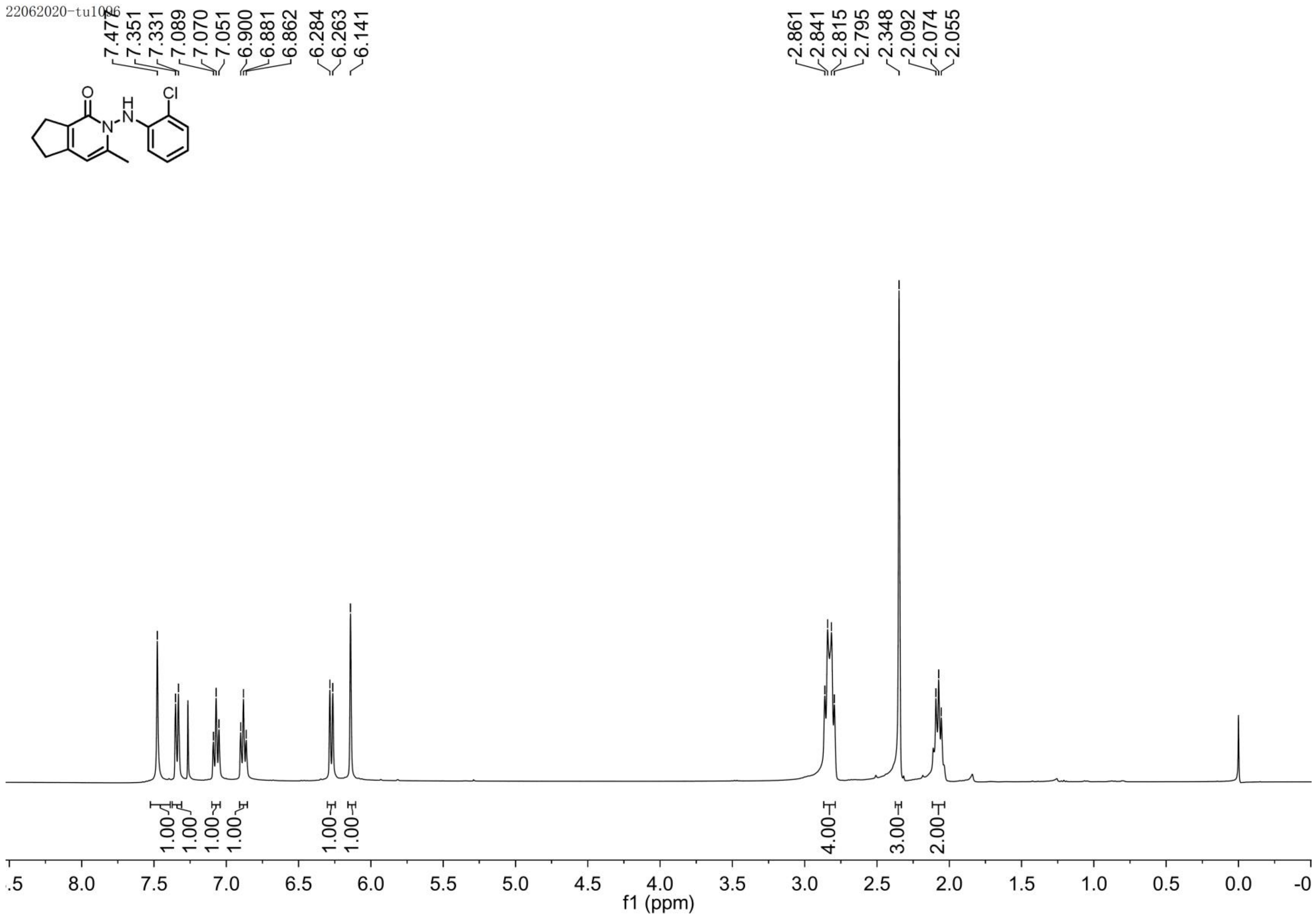

Supplementary Figure 55 ^{1}H NMR Spectrum of Compound **8**

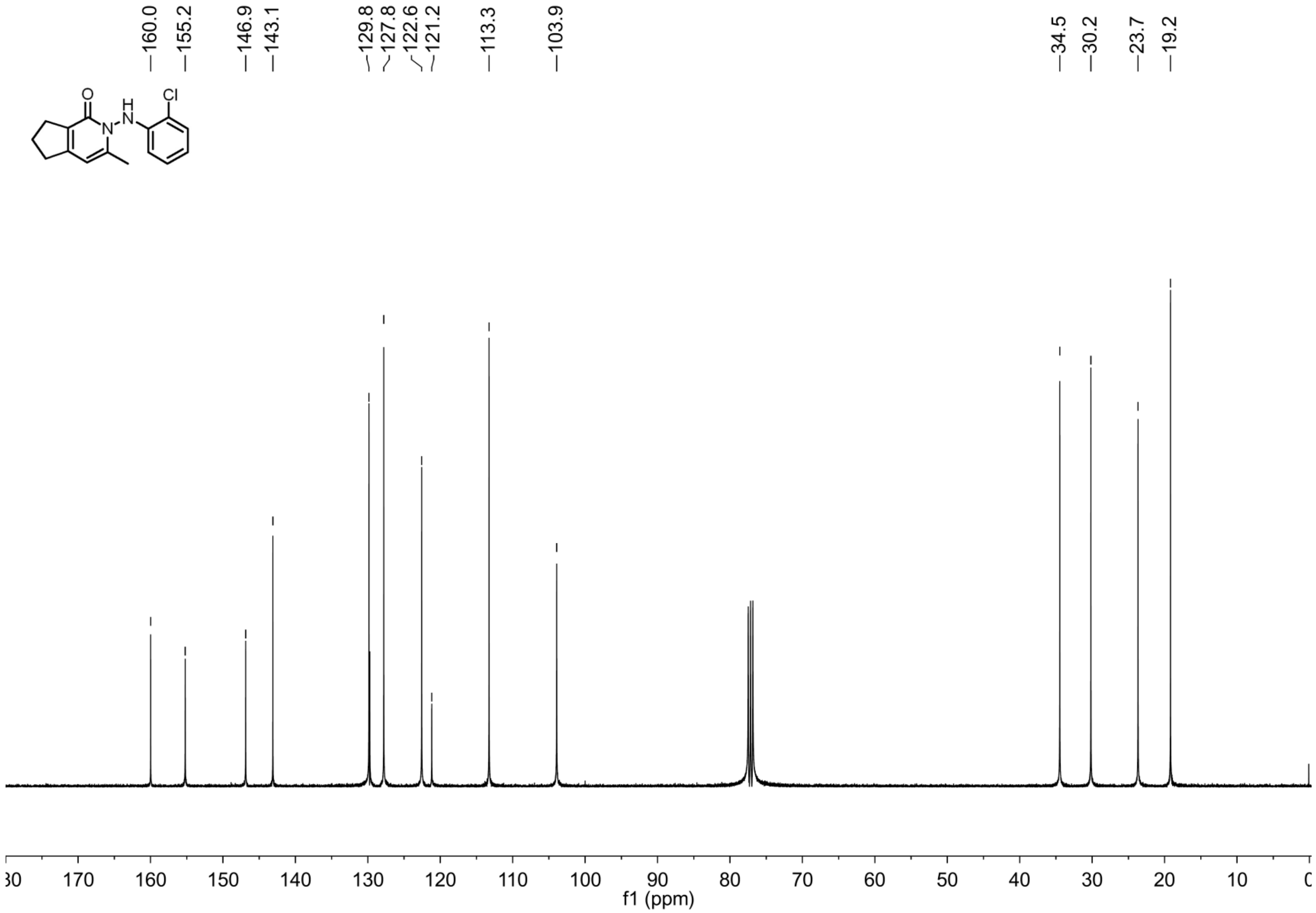

Supplementary Figure 56 ^{13}C NMR Spectrum of Compound **8**

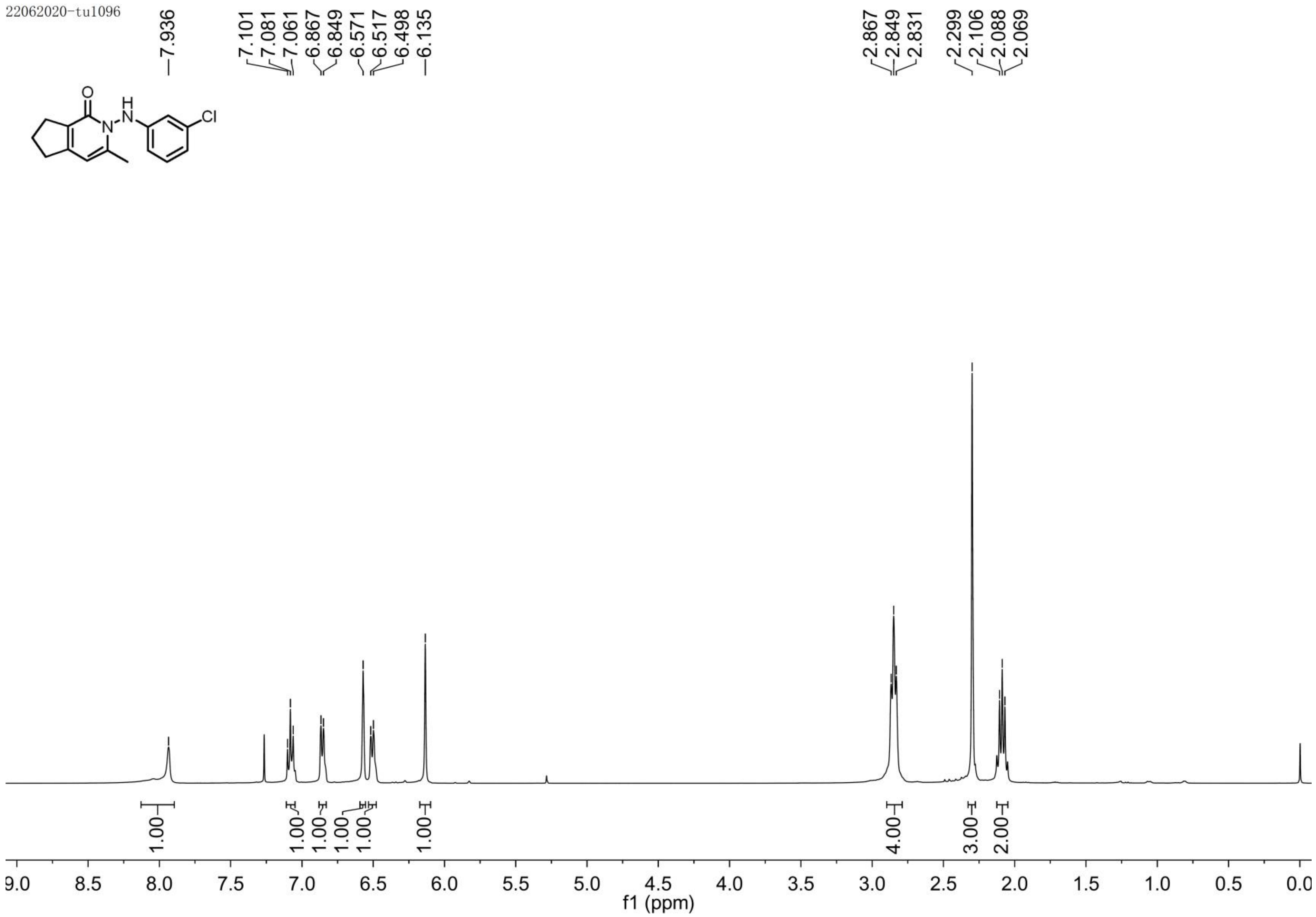

Supplementary Figure 57 ^{1}H NMR Spectrum of Compound **9**

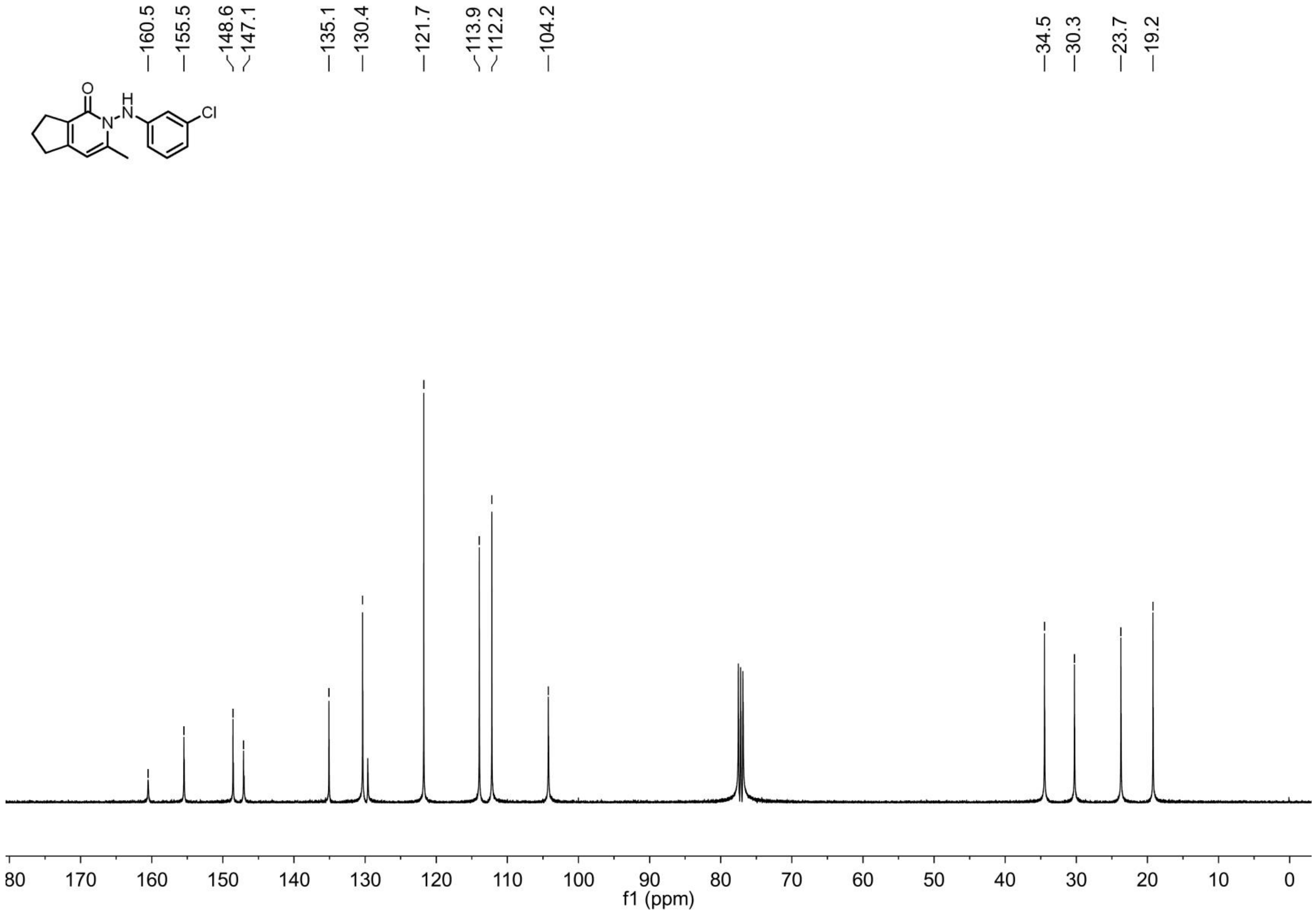

Supplementary Figure 58 ^{13}C NMR Spectrum of Compound **9**

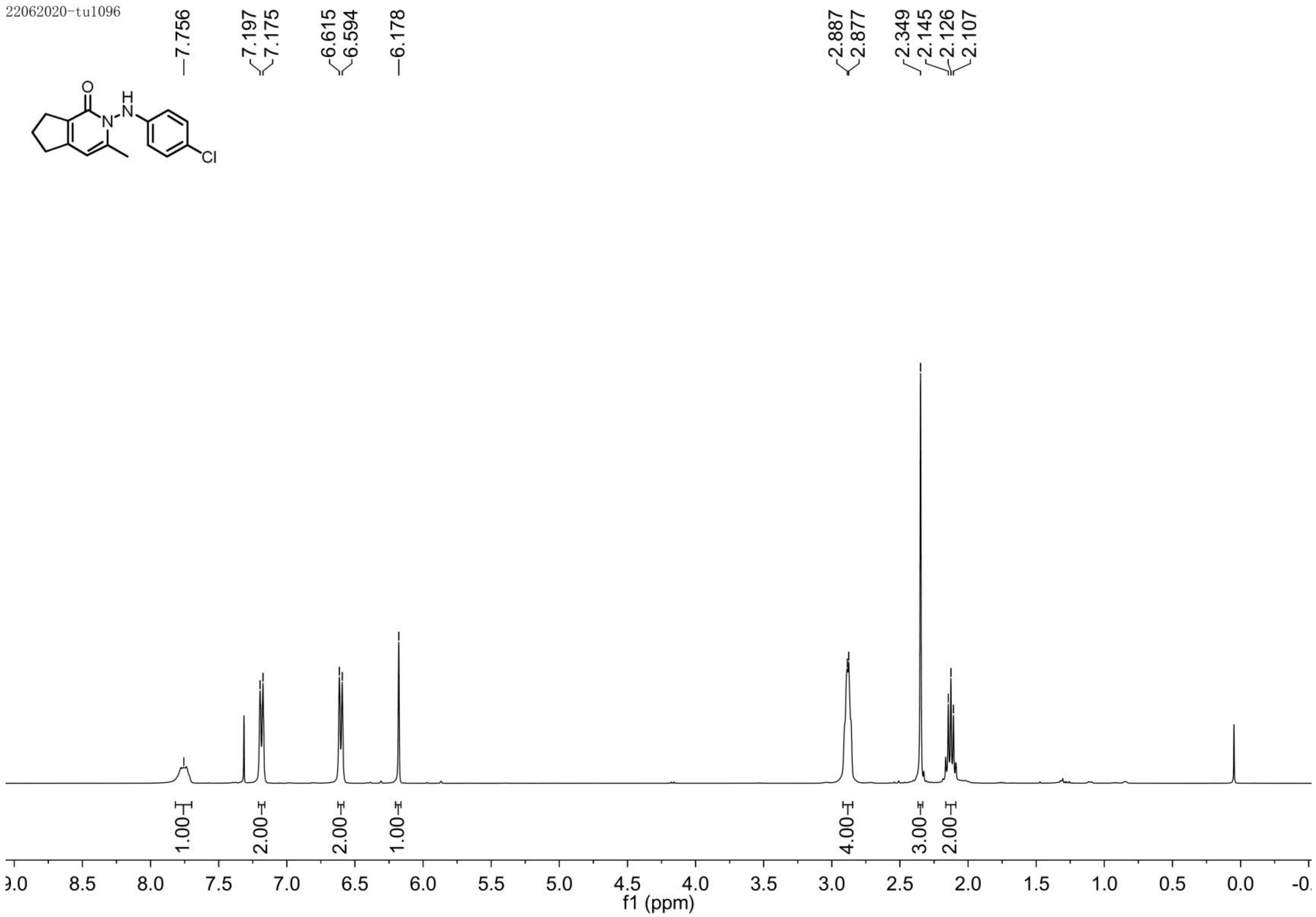

Supplementary Figure 59 ^{1}H NMR Spectrum of Compound **10**

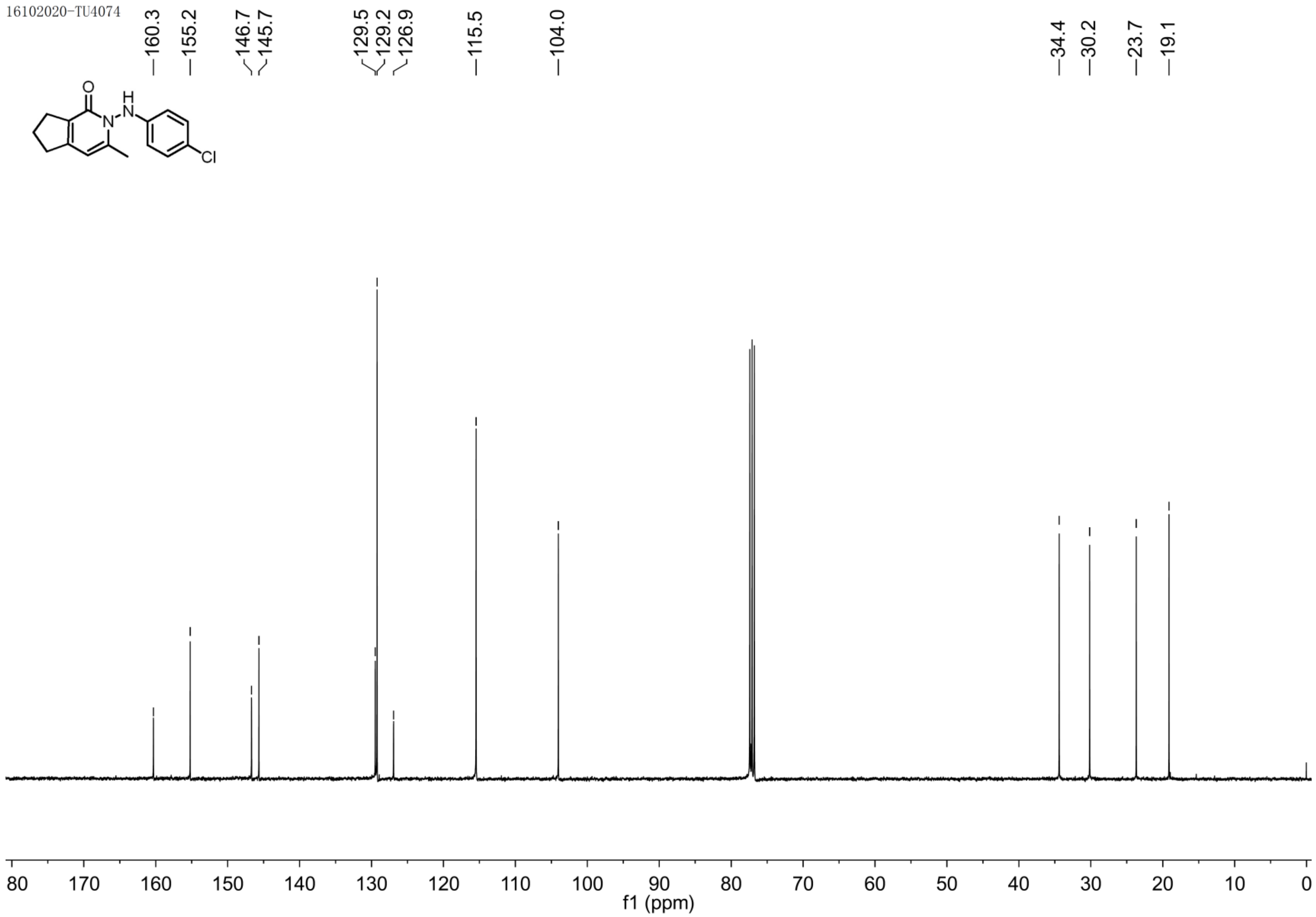

Supplementary Figure 60 ^{13}C NMR Spectrum of Compound **10**

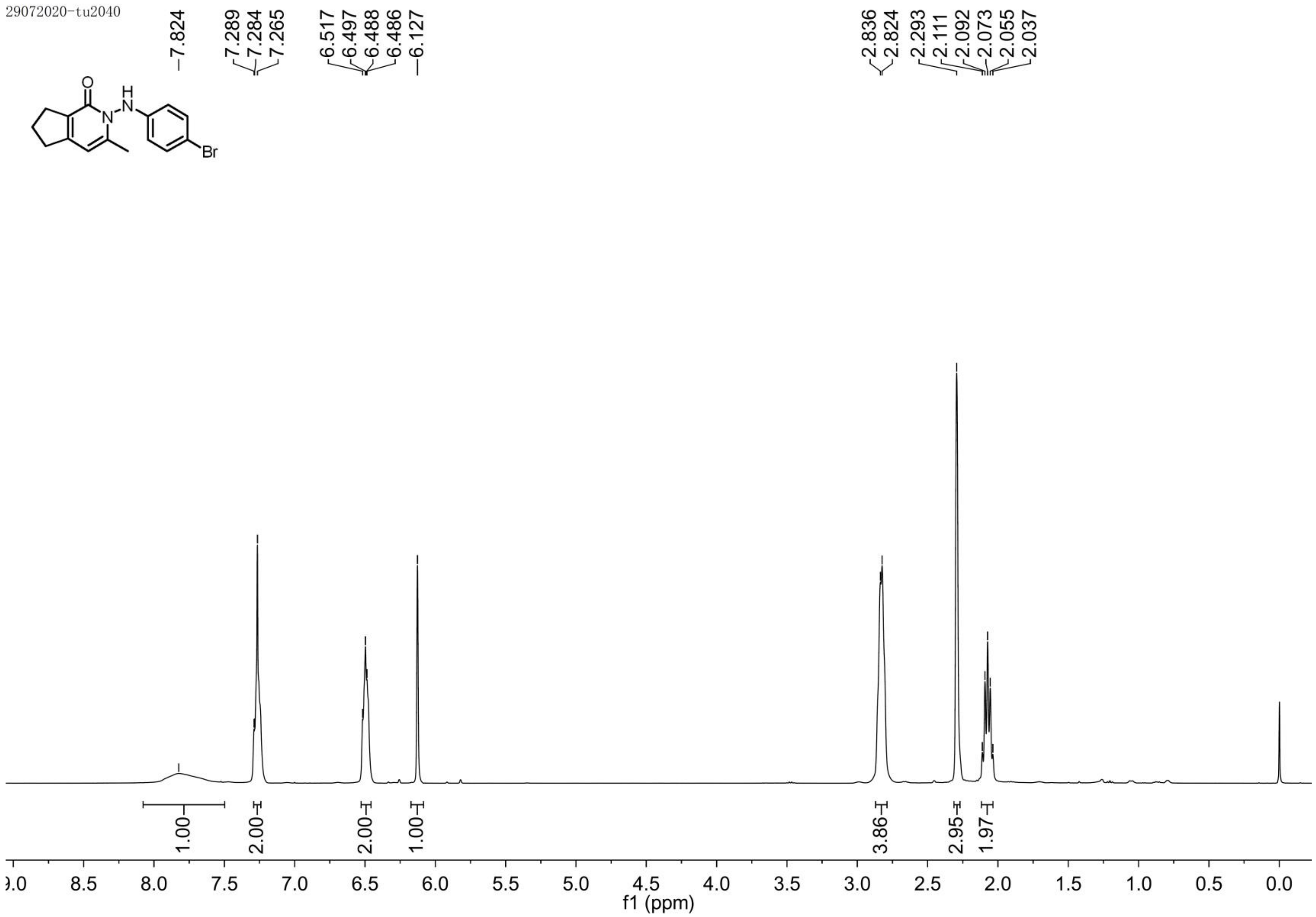

Supplementary Figure 61 ^{1}H NMR Spectrum of Compound **11**

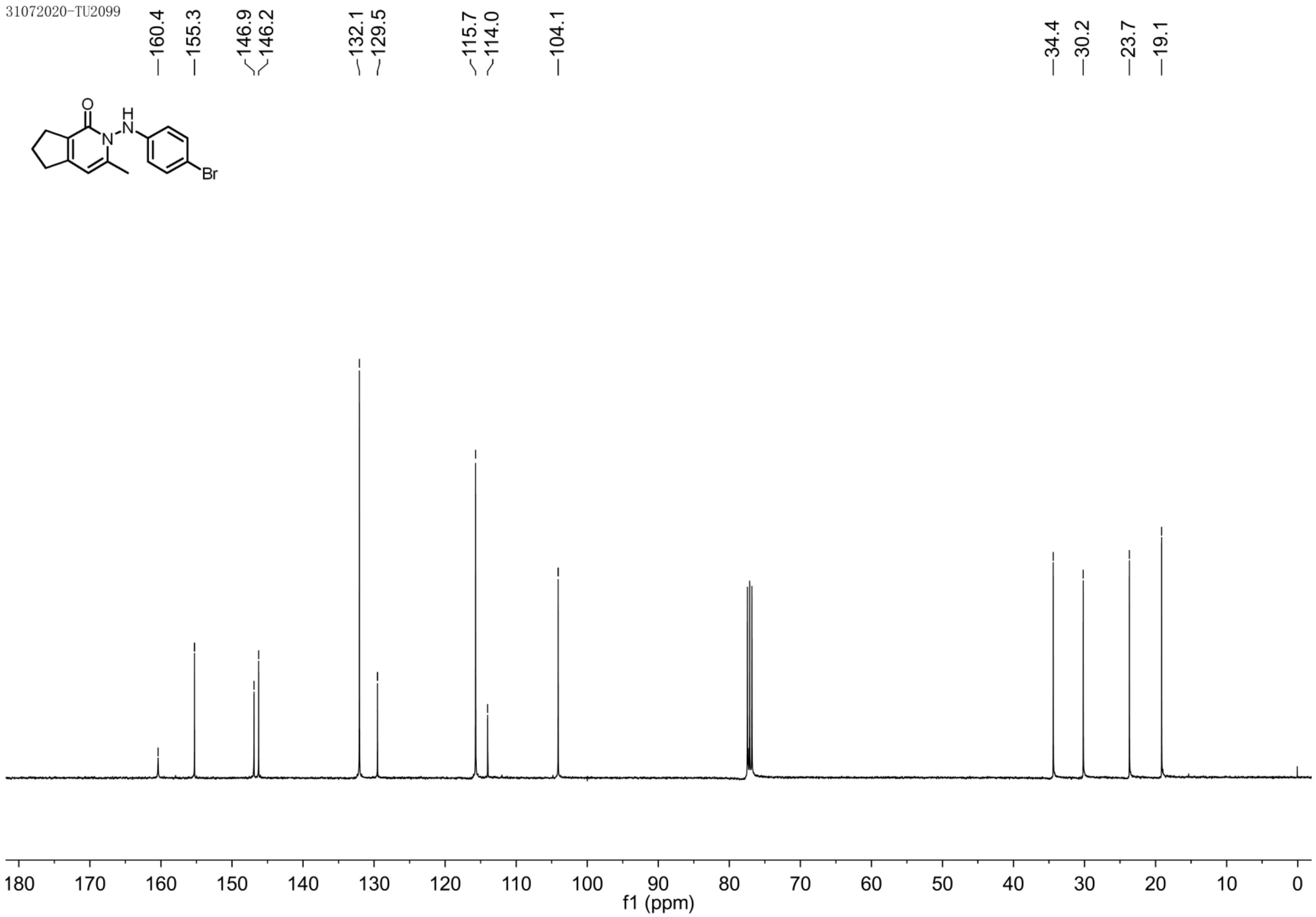

Supplementary Figure 62 ^{13}C NMR Spectrum of Compound **11**

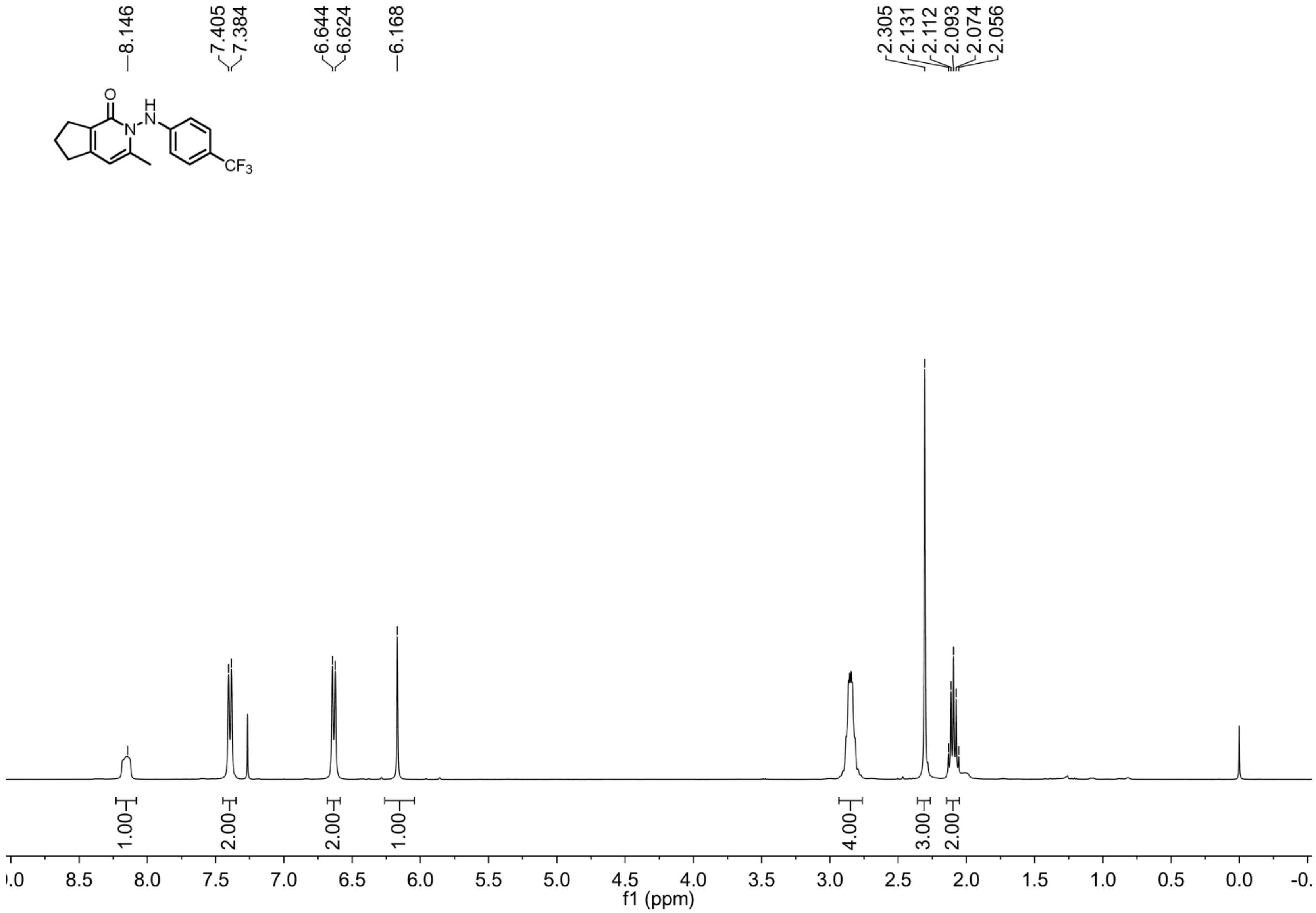

Supplementary Figure 63 ^{1}H NMR Spectrum of Compound **12**

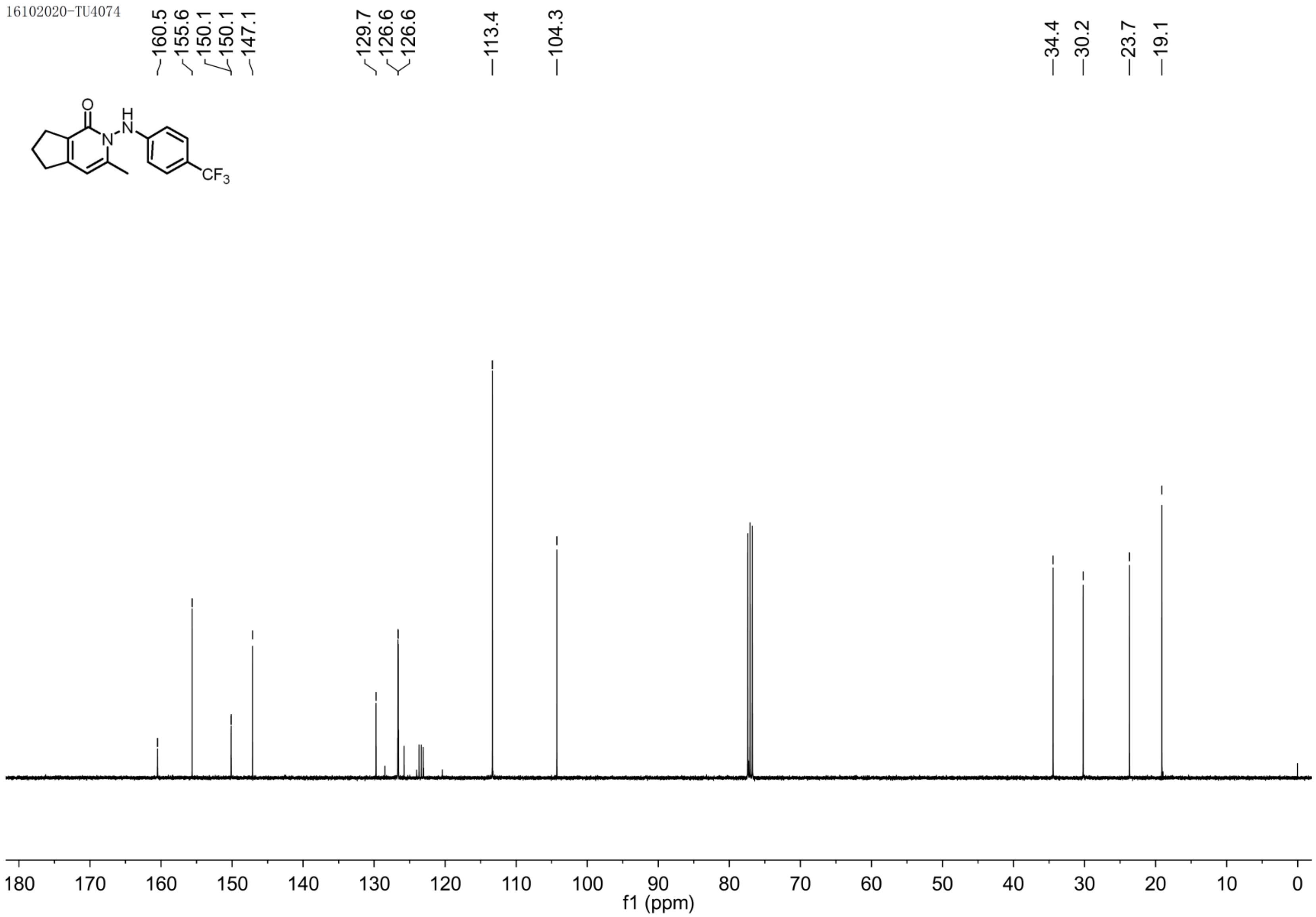

Supplementary Figure 64 ^{13}C NMR Spectrum of Compound **12**

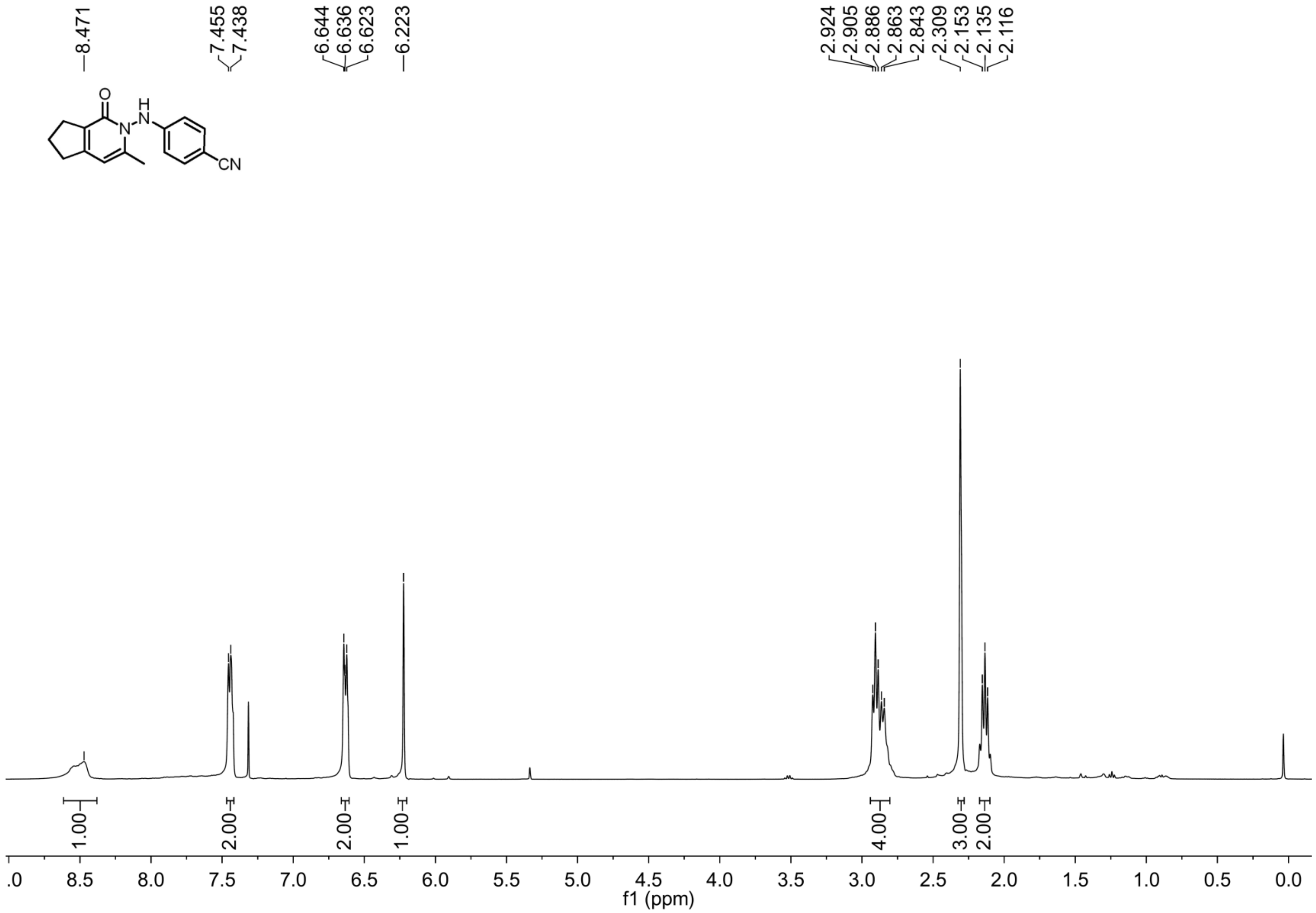

Supplementary Figure 65 ^{1}H NMR Spectrum of Compound **13**

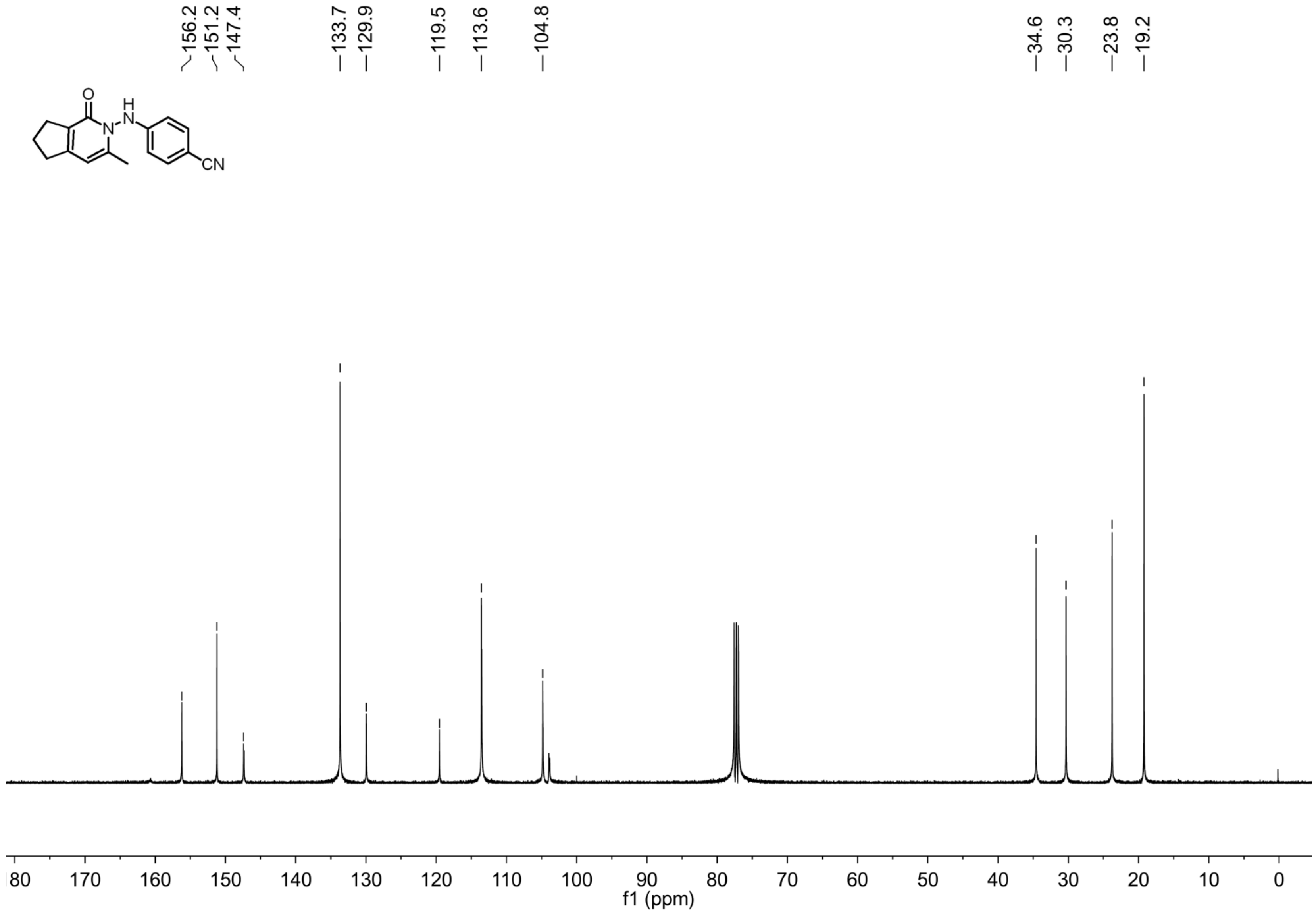

Supplementary Figure 66 ^{13}C NMR Spectrum of Compound **13**

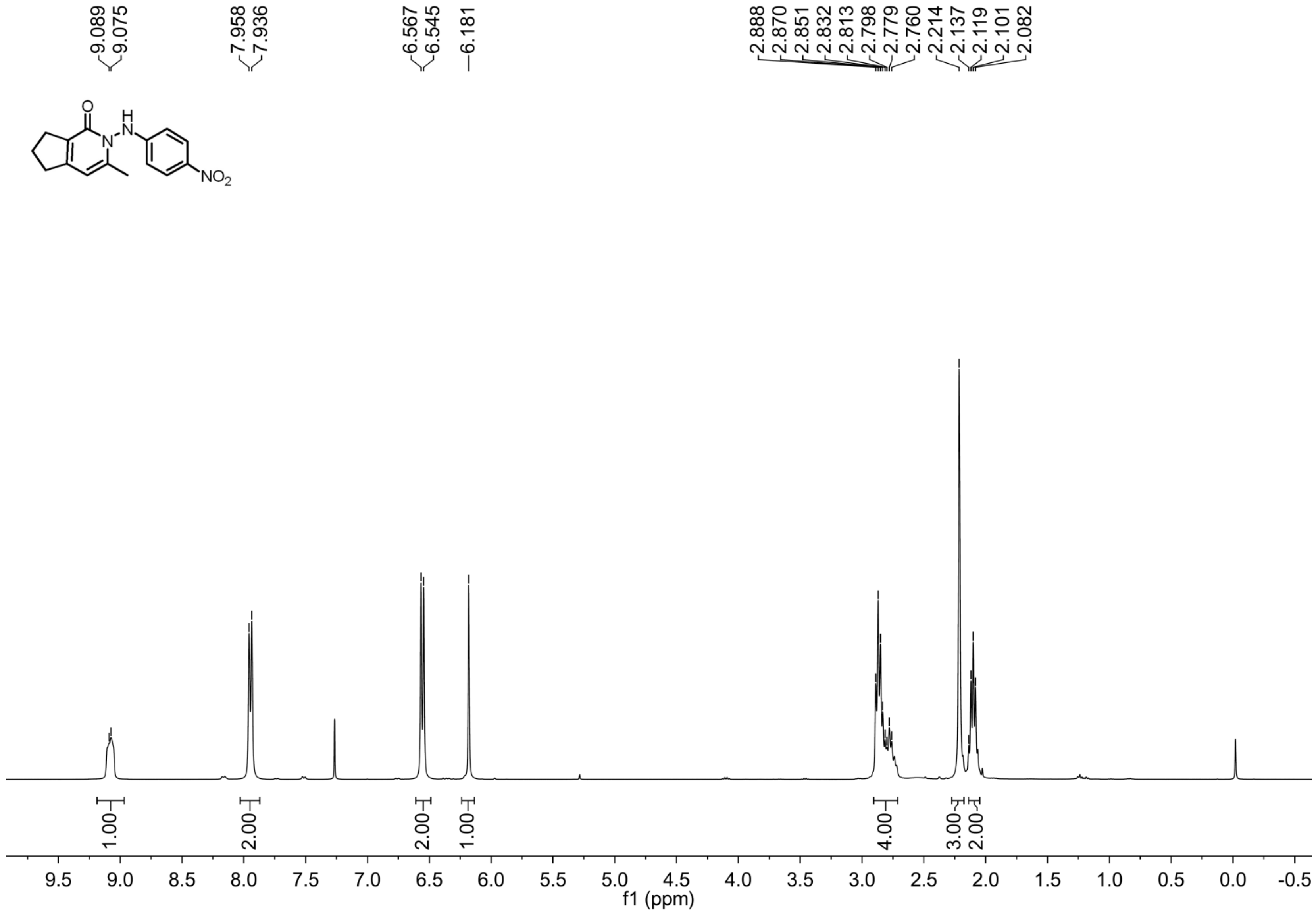

Supplementary Figure 67 ^{1}H NMR Spectrum of Compound **14**

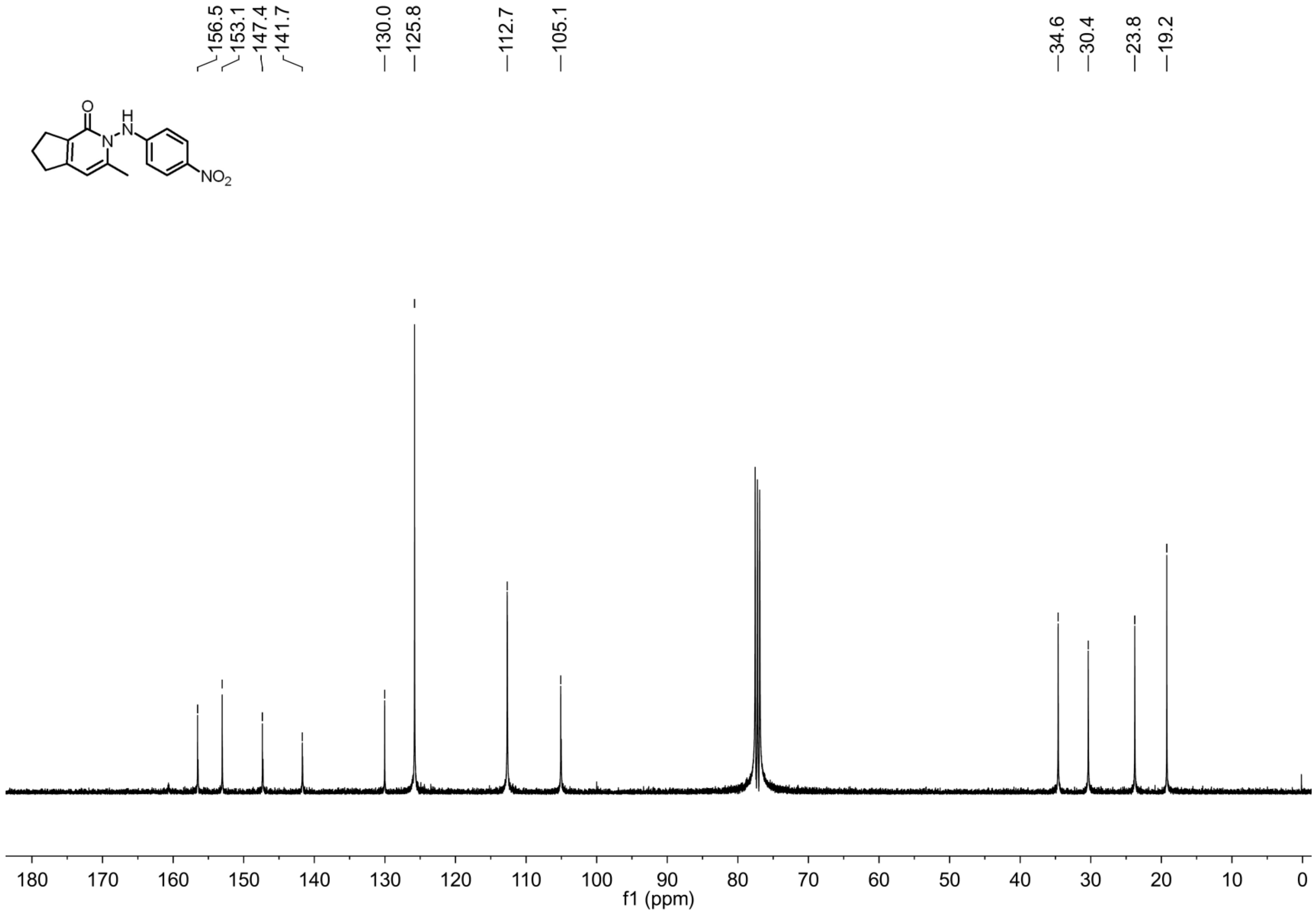

Supplementary Figure 68 ^{13}C NMR Spectrum of Compound **14**

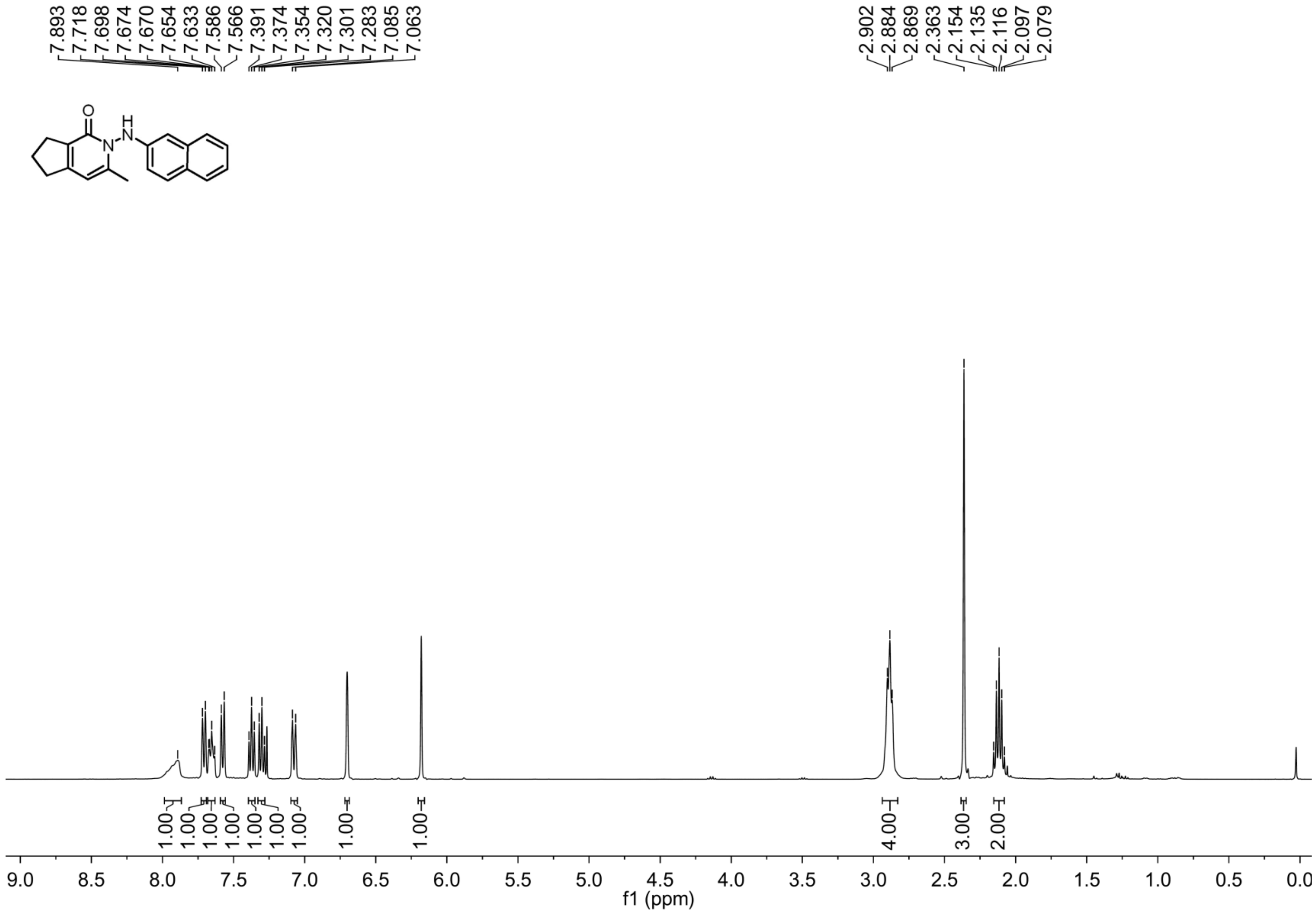

Supplementary Figure 69 ^{1}H NMR Spectrum of Compound **15**

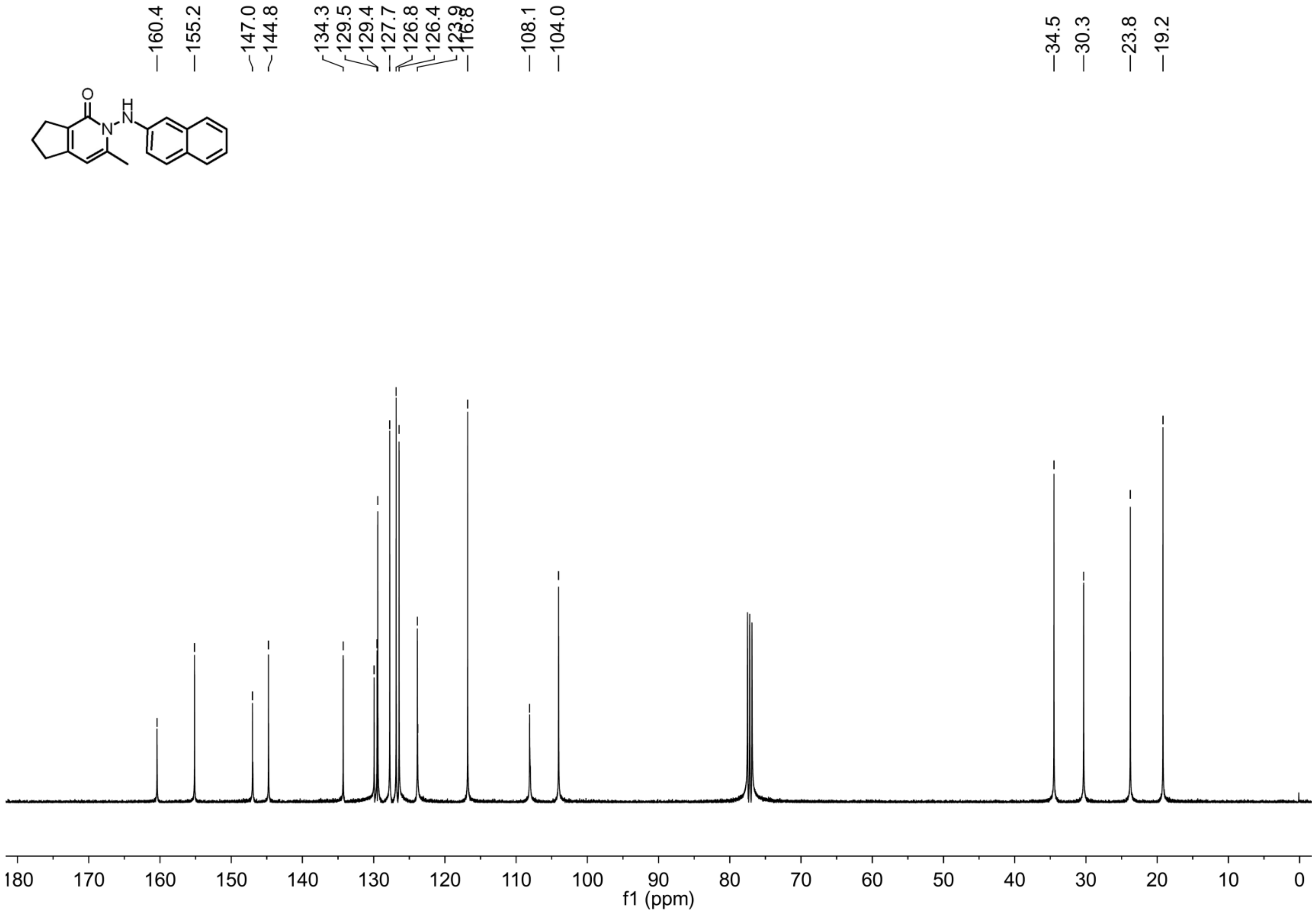

Supplementary Figure 70 ^{13}C NMR Spectrum of Compound **15**

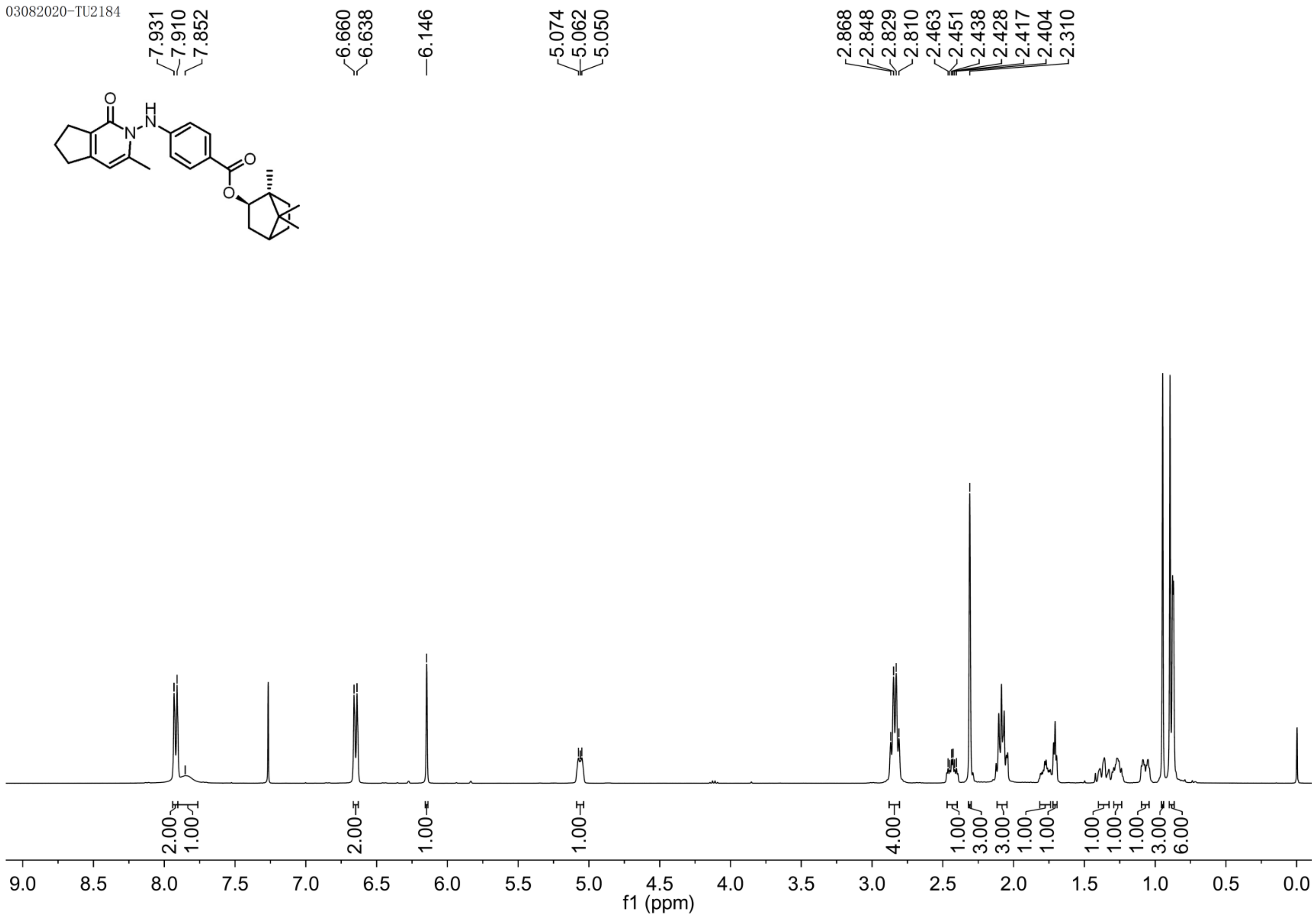

Supplementary Figure 71 ^{1}H NMR Spectrum of Compound **16**

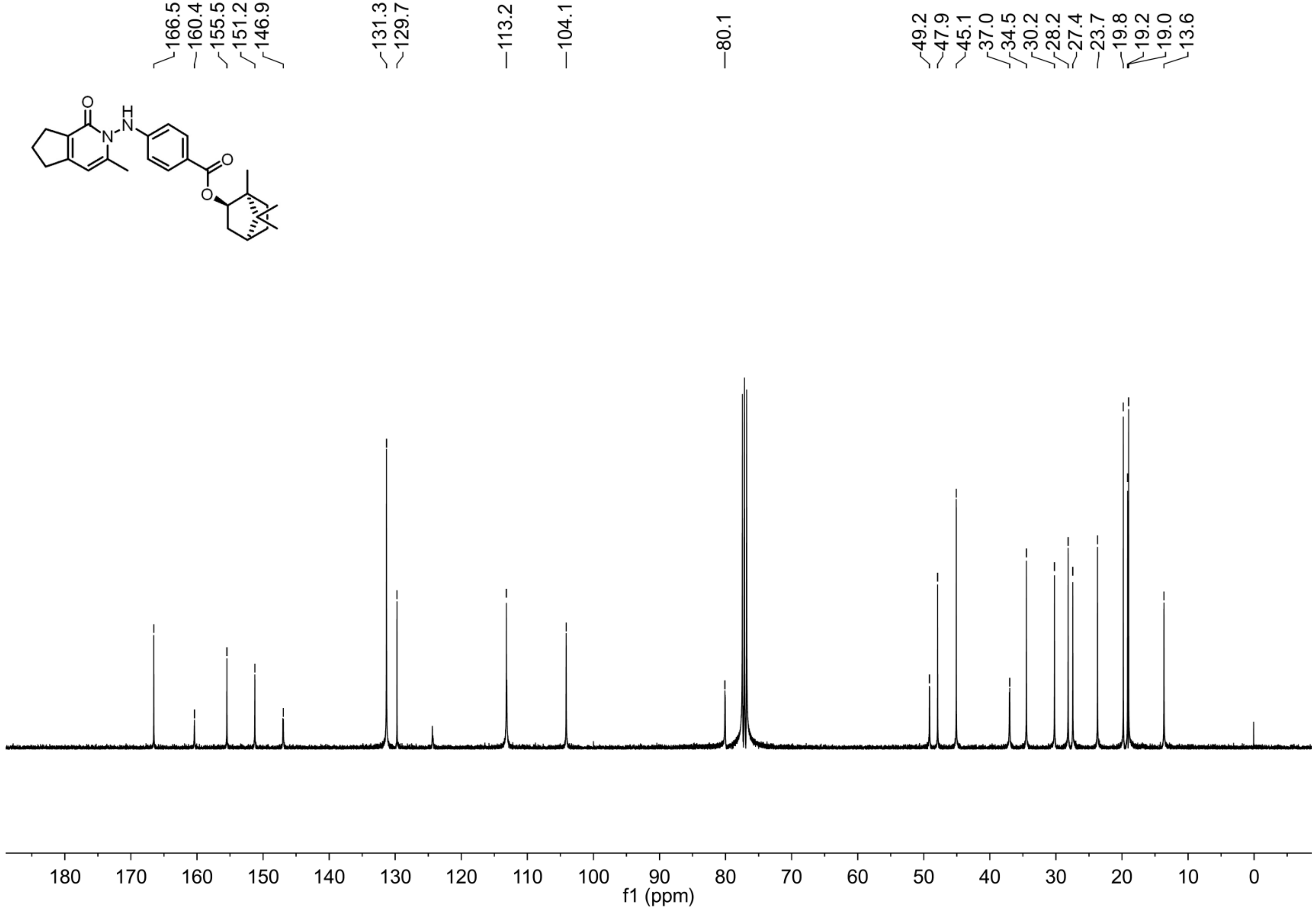

Supplementary Figure 72 ^{13}C NMR Spectrum of Compound **16**

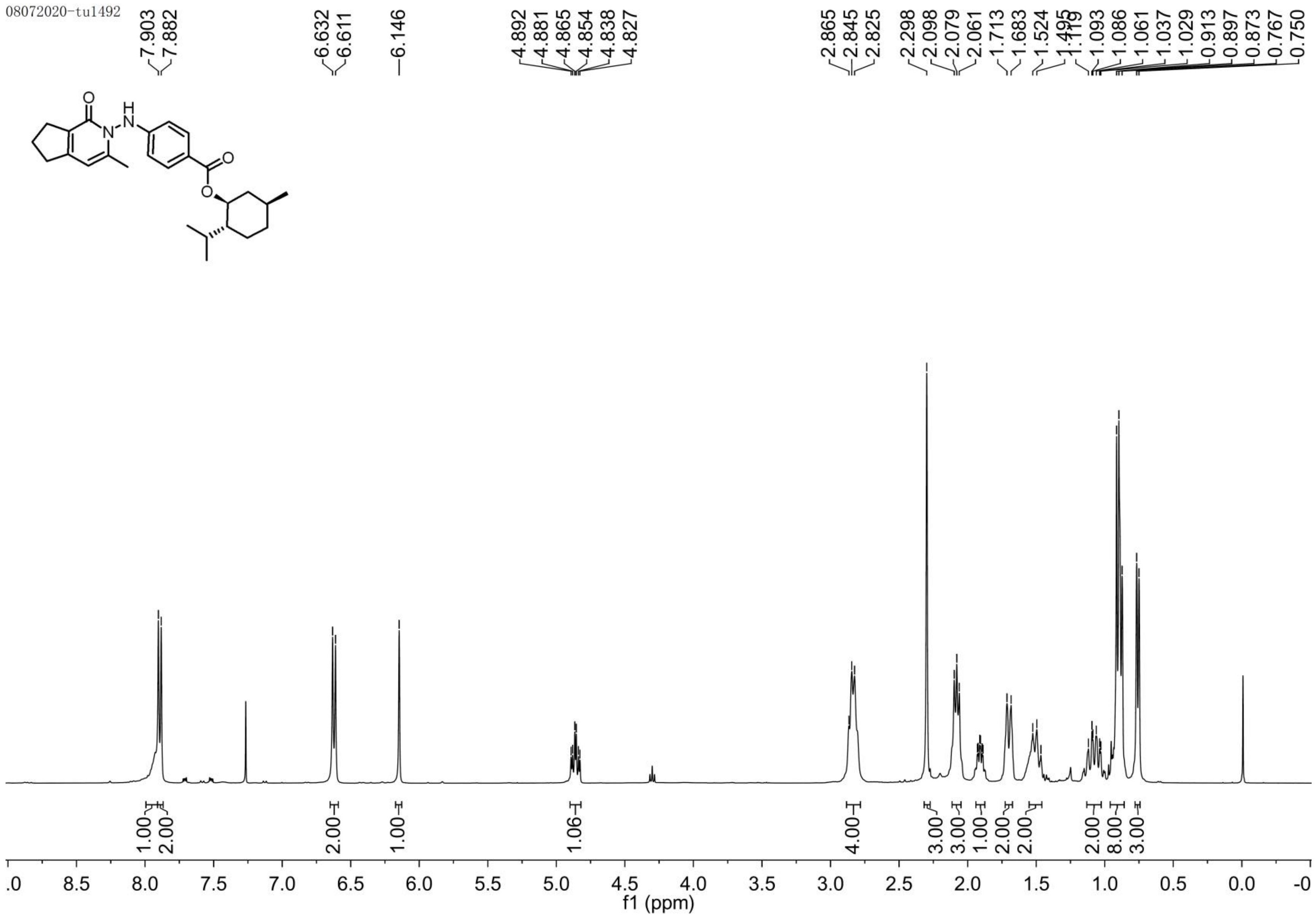

Supplementary Figure 73 ^{1}H NMR Spectrum of Compound **17**

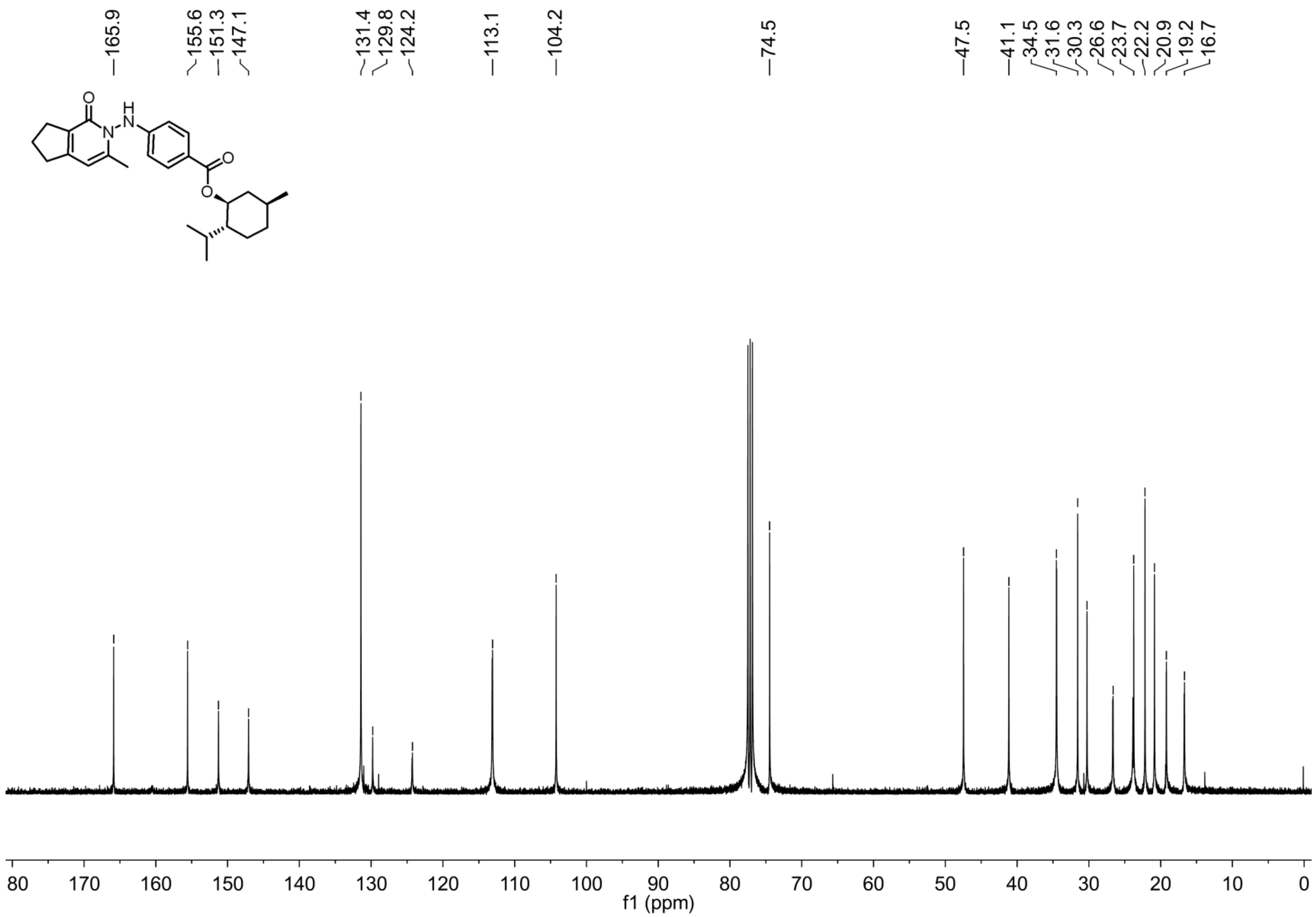

Supplementary Figure 74 ^{13}C NMR Spectrum of Compound **17**

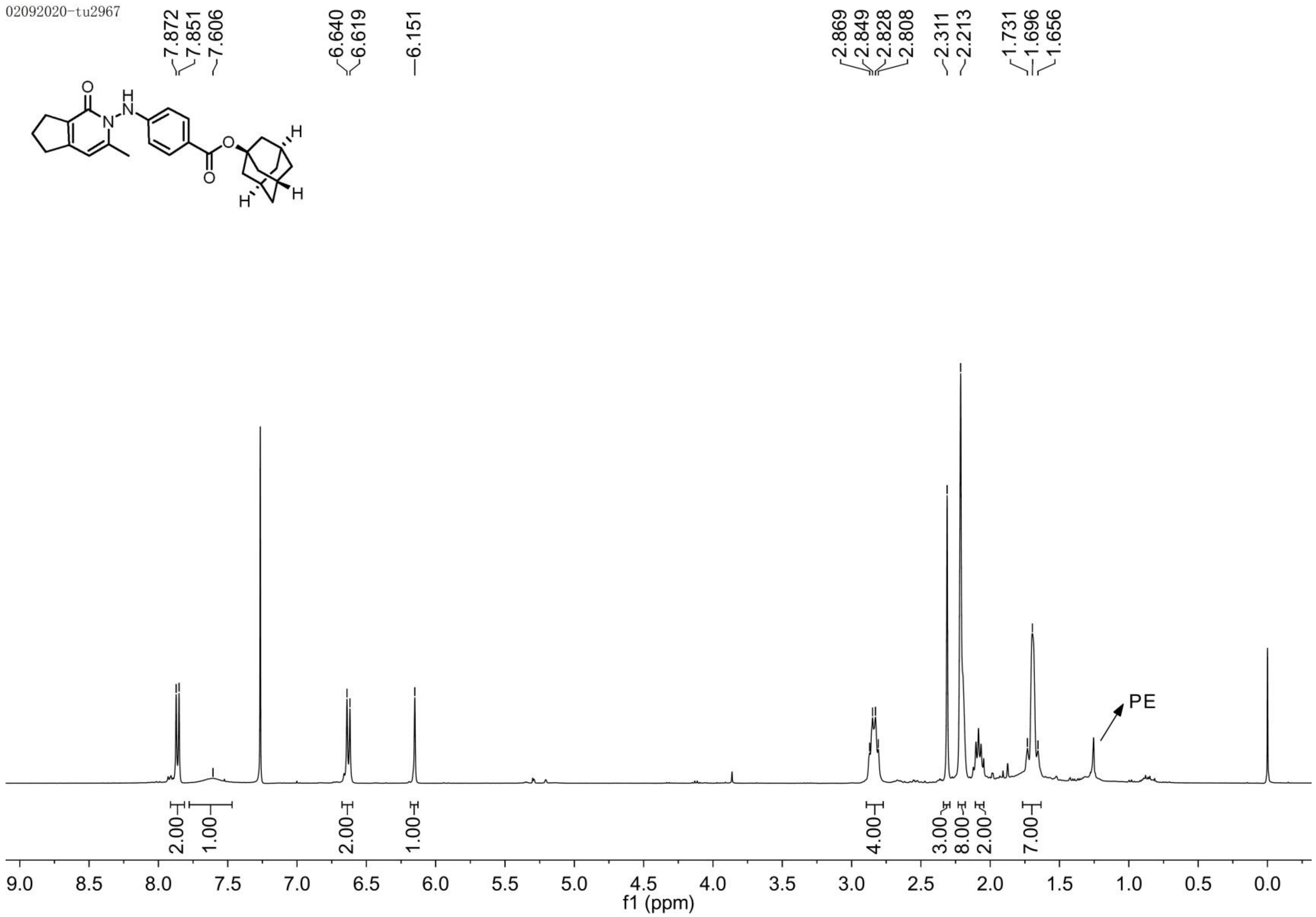

Supplementary Figure 75 ^{1}H NMR Spectrum of Compound **18**

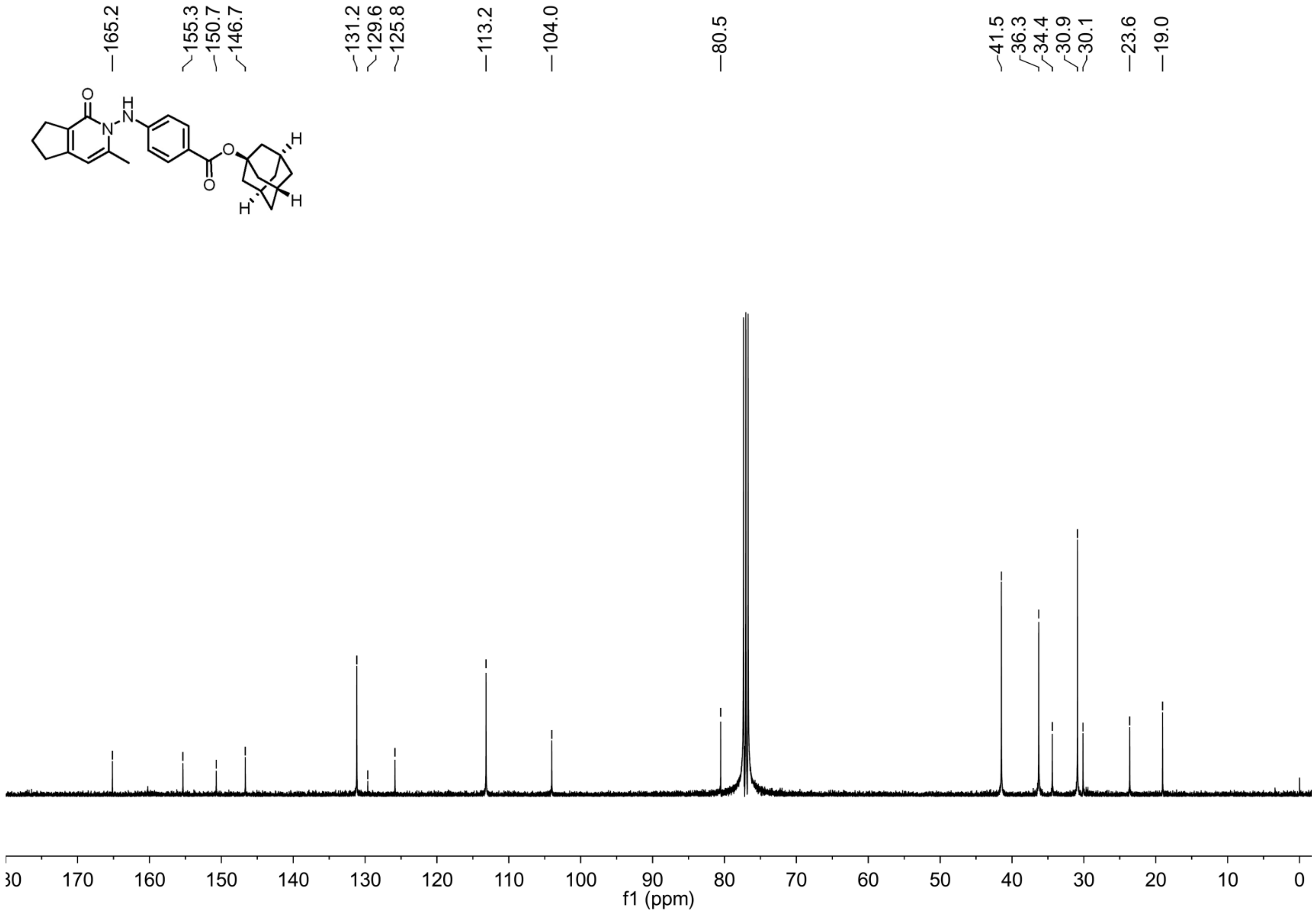

Supplementary Figure 76 ^{13}C NMR Spectrum of Compound **18**

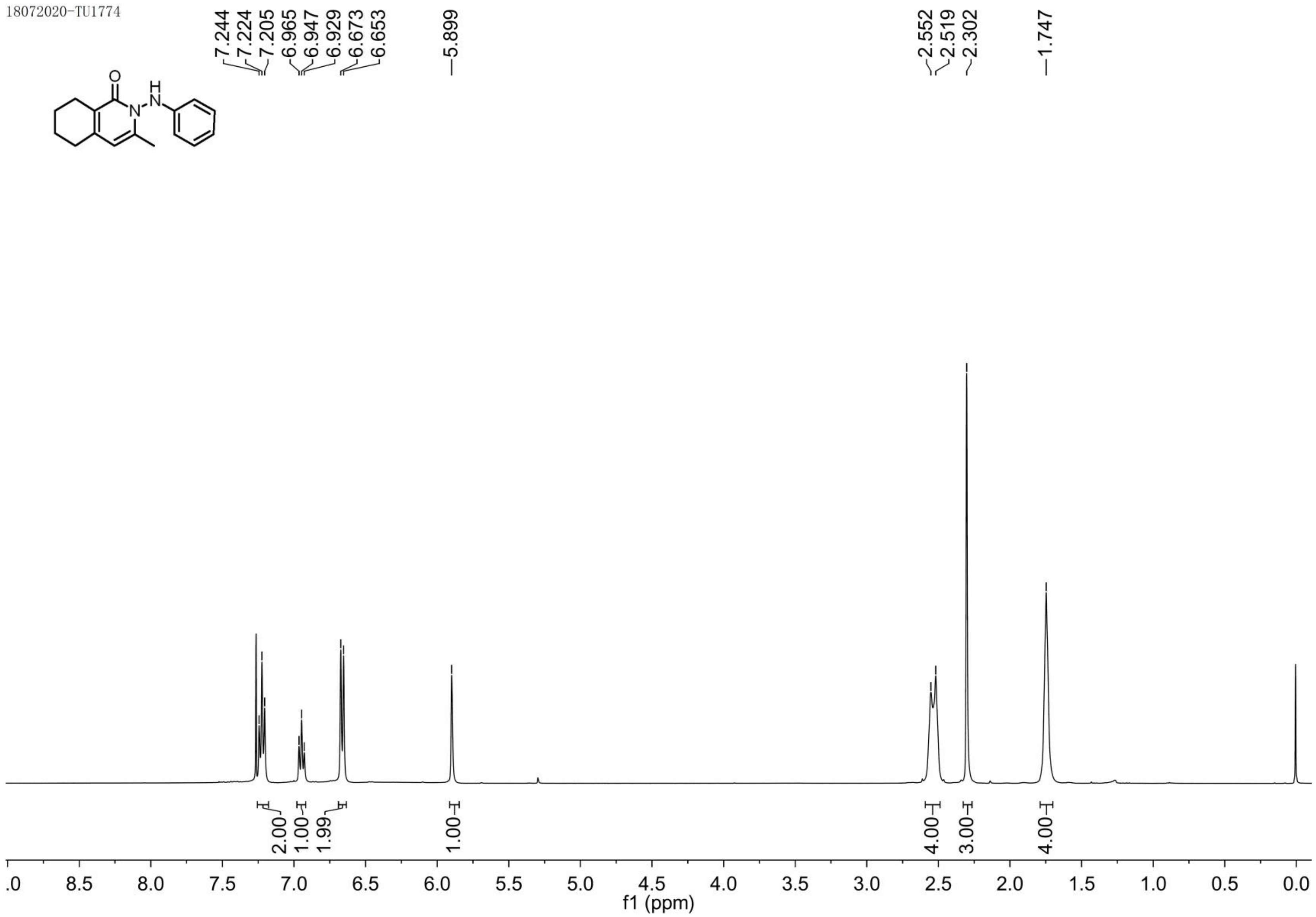

Supplementary Figure 77 ^{1}H NMR Spectrum of Compound **19**

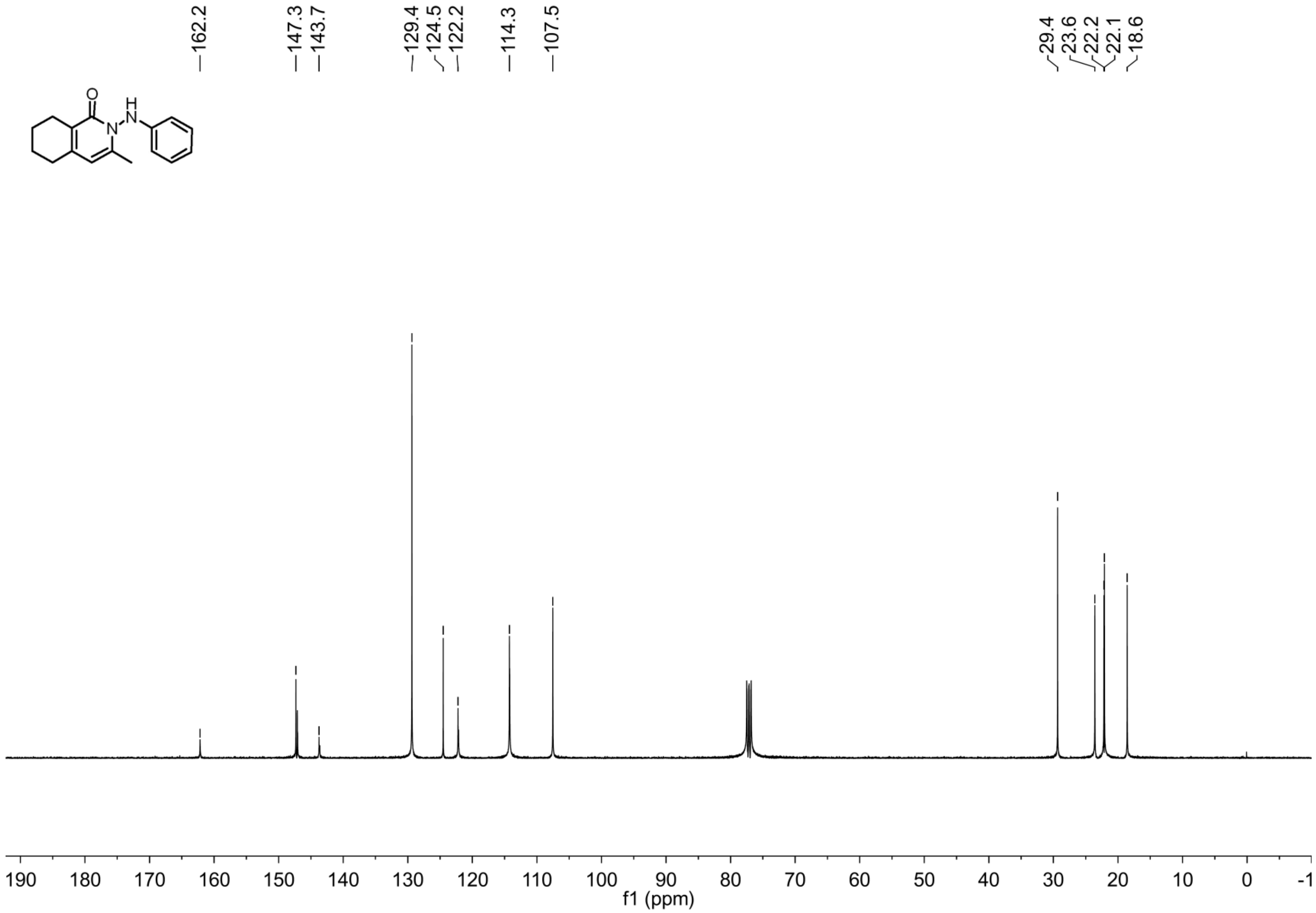

Supplementary Figure 78 ^{13}C NMR Spectrum of Compound **19**

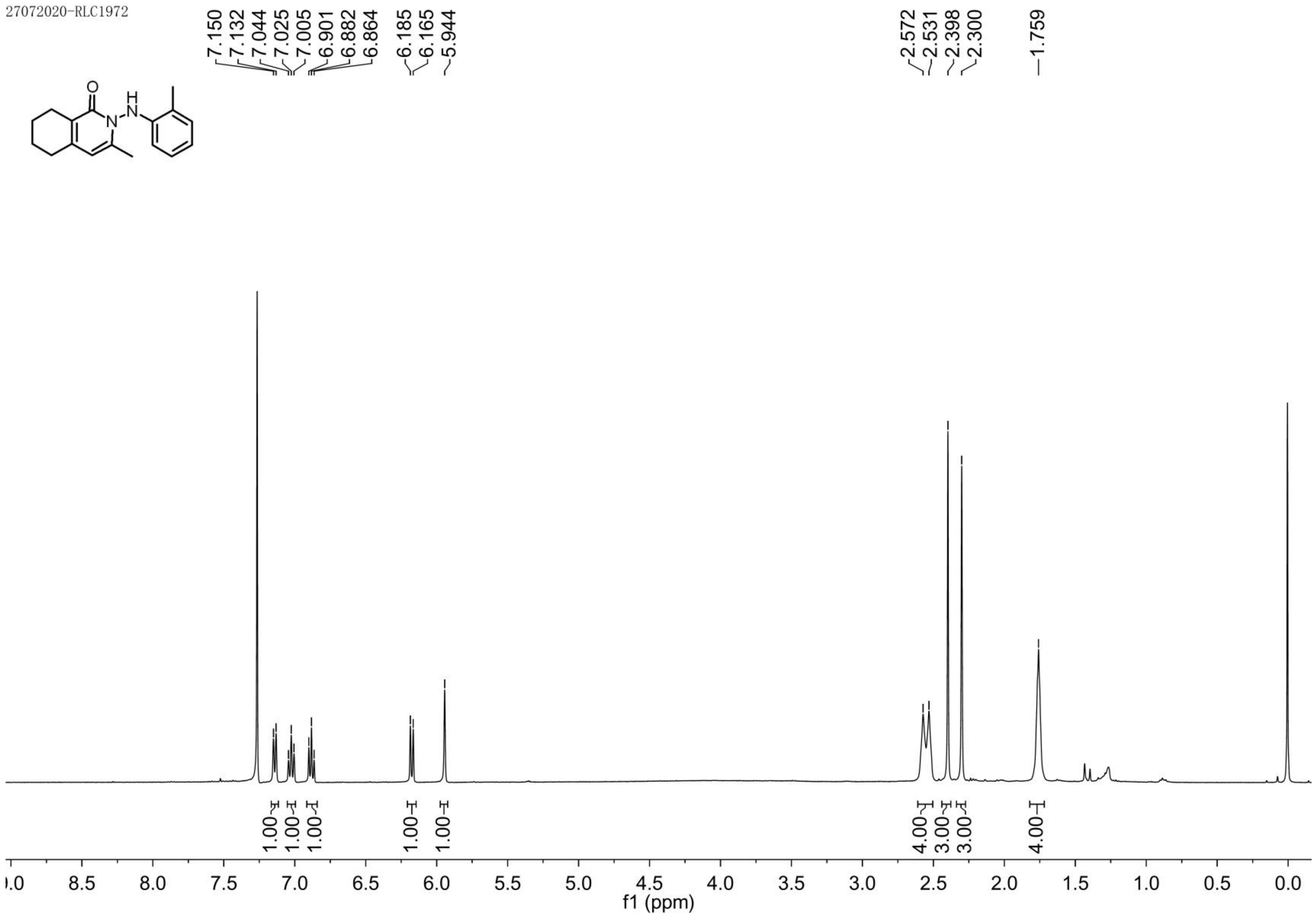

Supplementary Figure 79 ^{1}H NMR Spectrum of Compound **20**

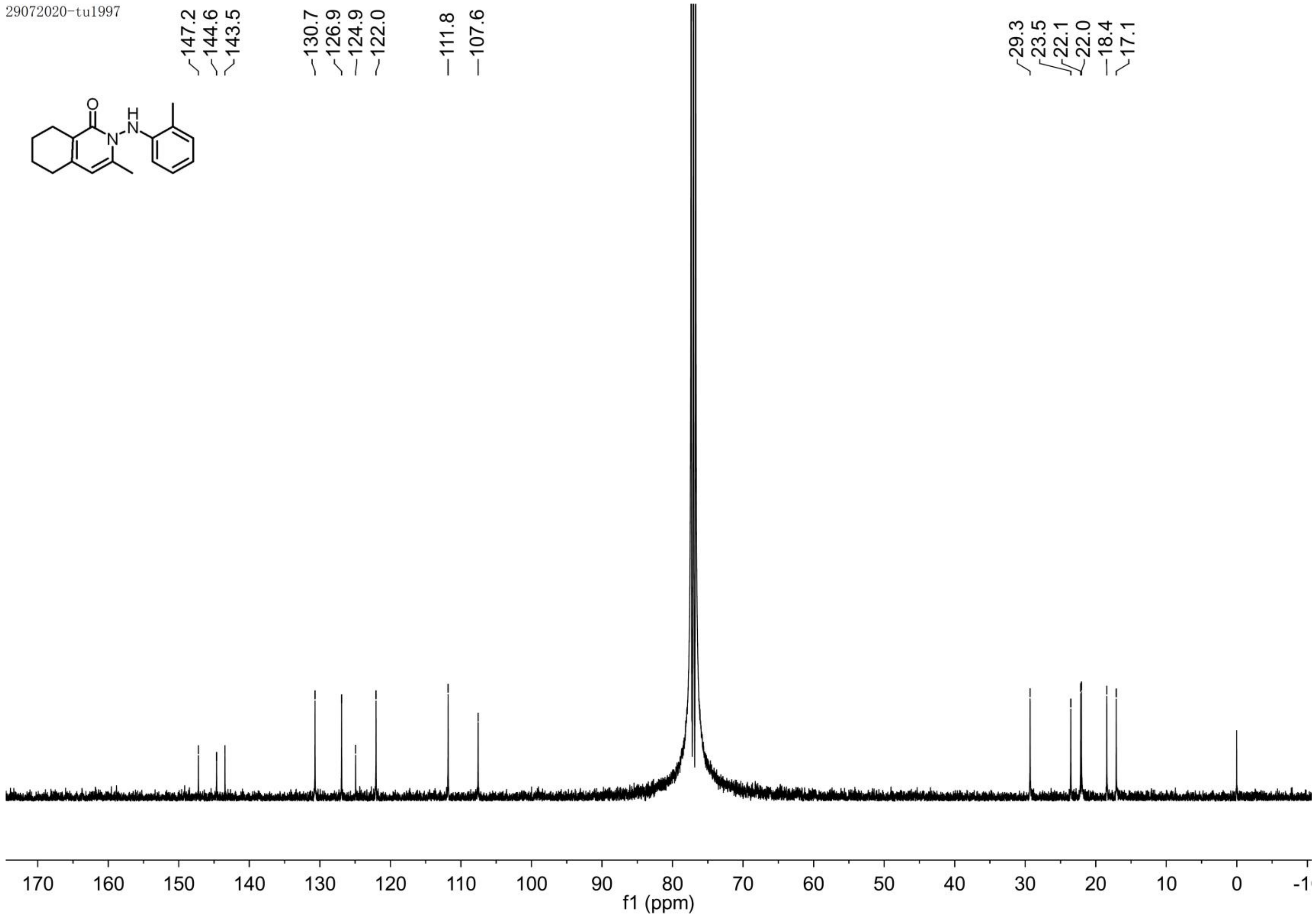

Supplementary Figure 80 ^{13}C NMR Spectrum of Compound **20**

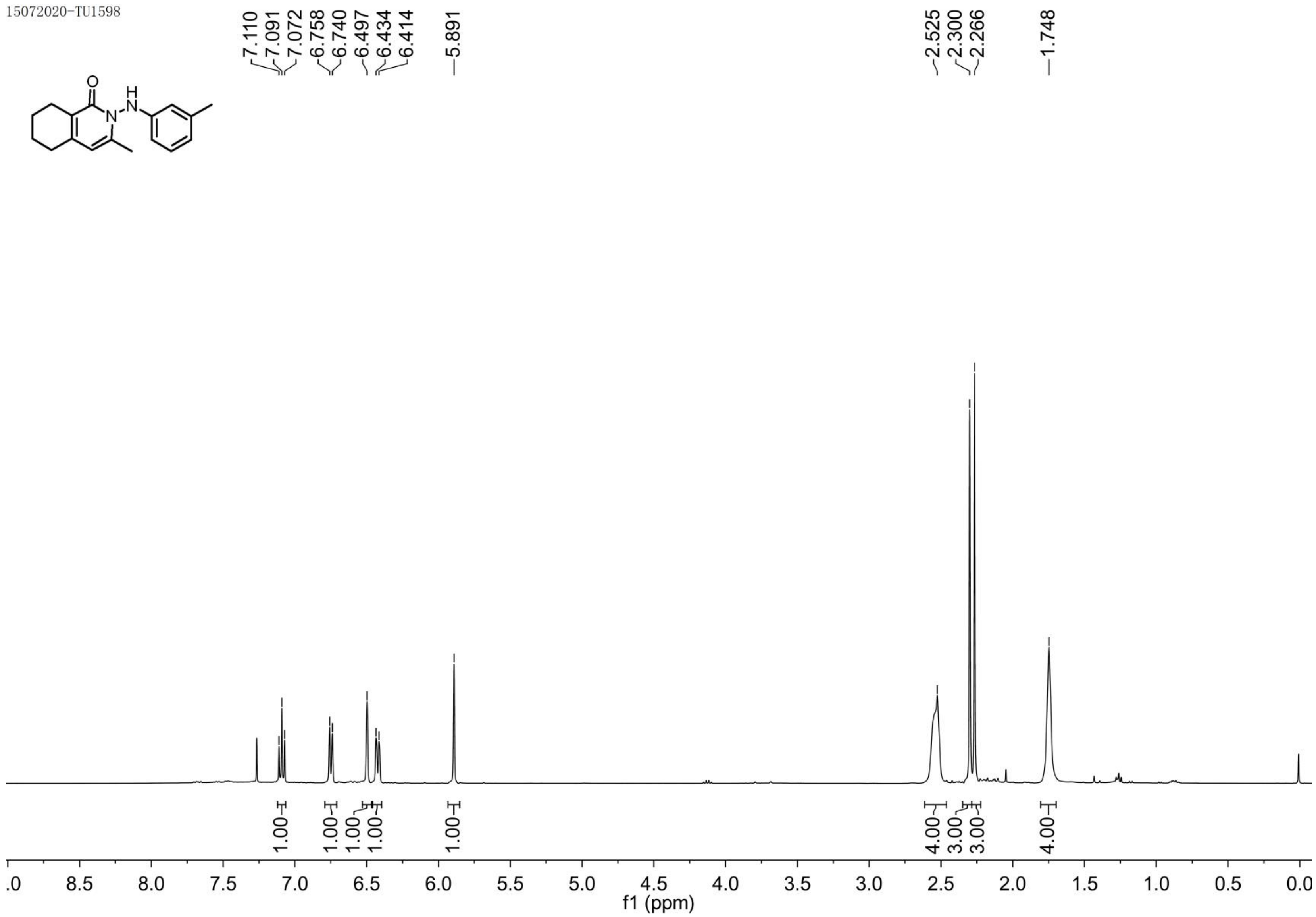

Supplementary Figure 81 ^{1}H NMR Spectrum of Compound **21**

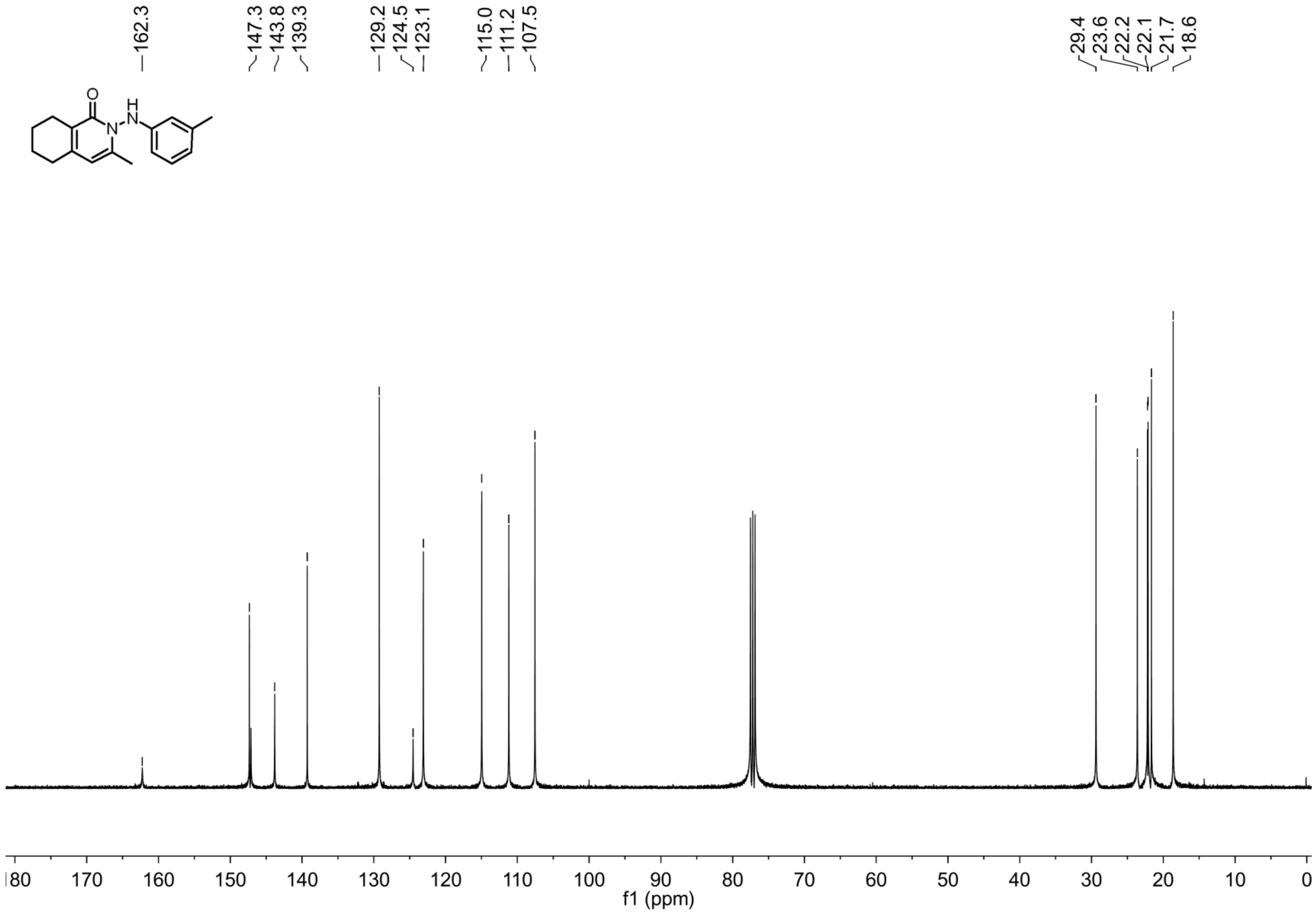

Supplementary Figure 82 ^{13}C NMR Spectrum of Compound **21**

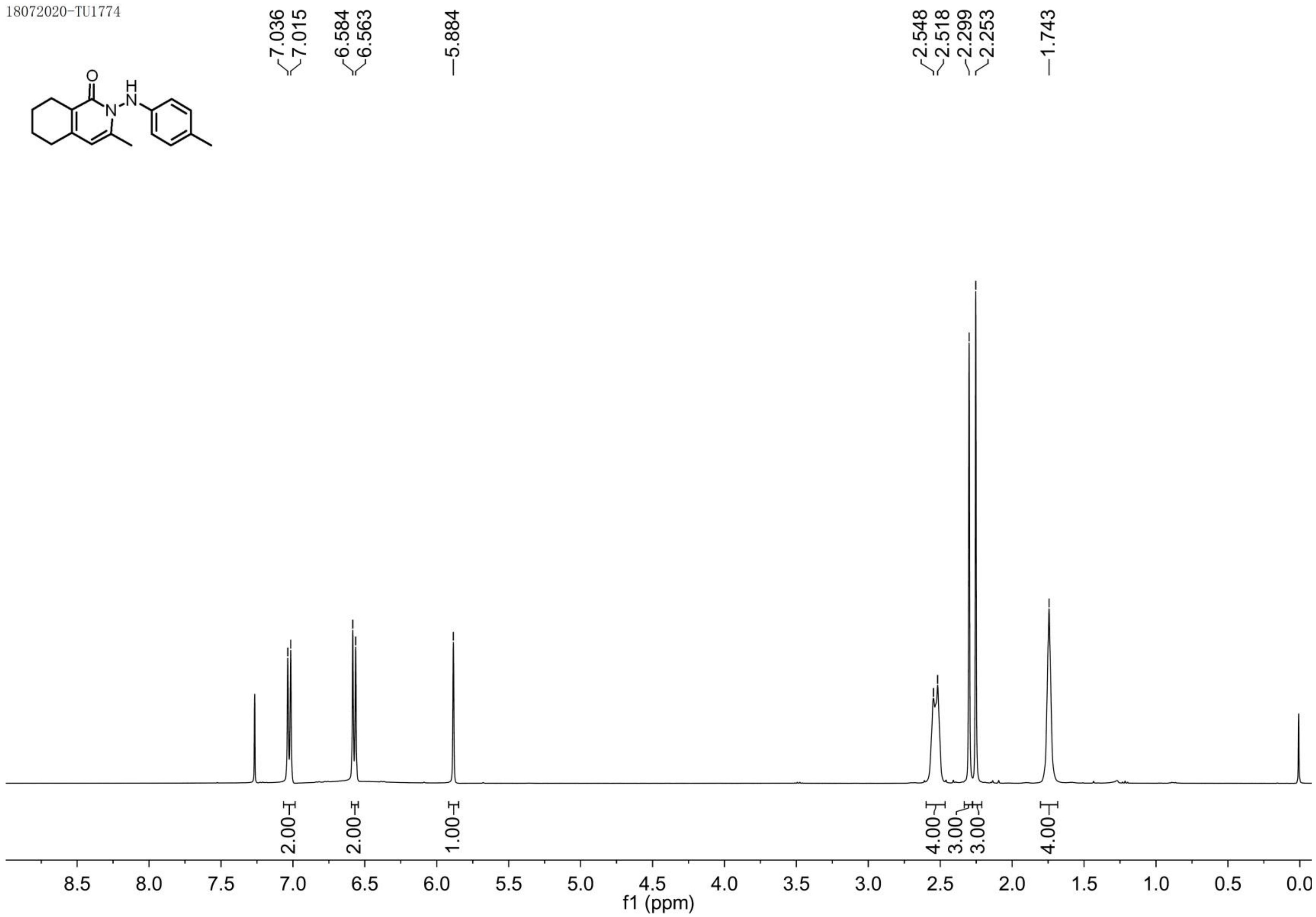

Supplementary Figure 83 ^{1}H NMR Spectrum of Compound **22**

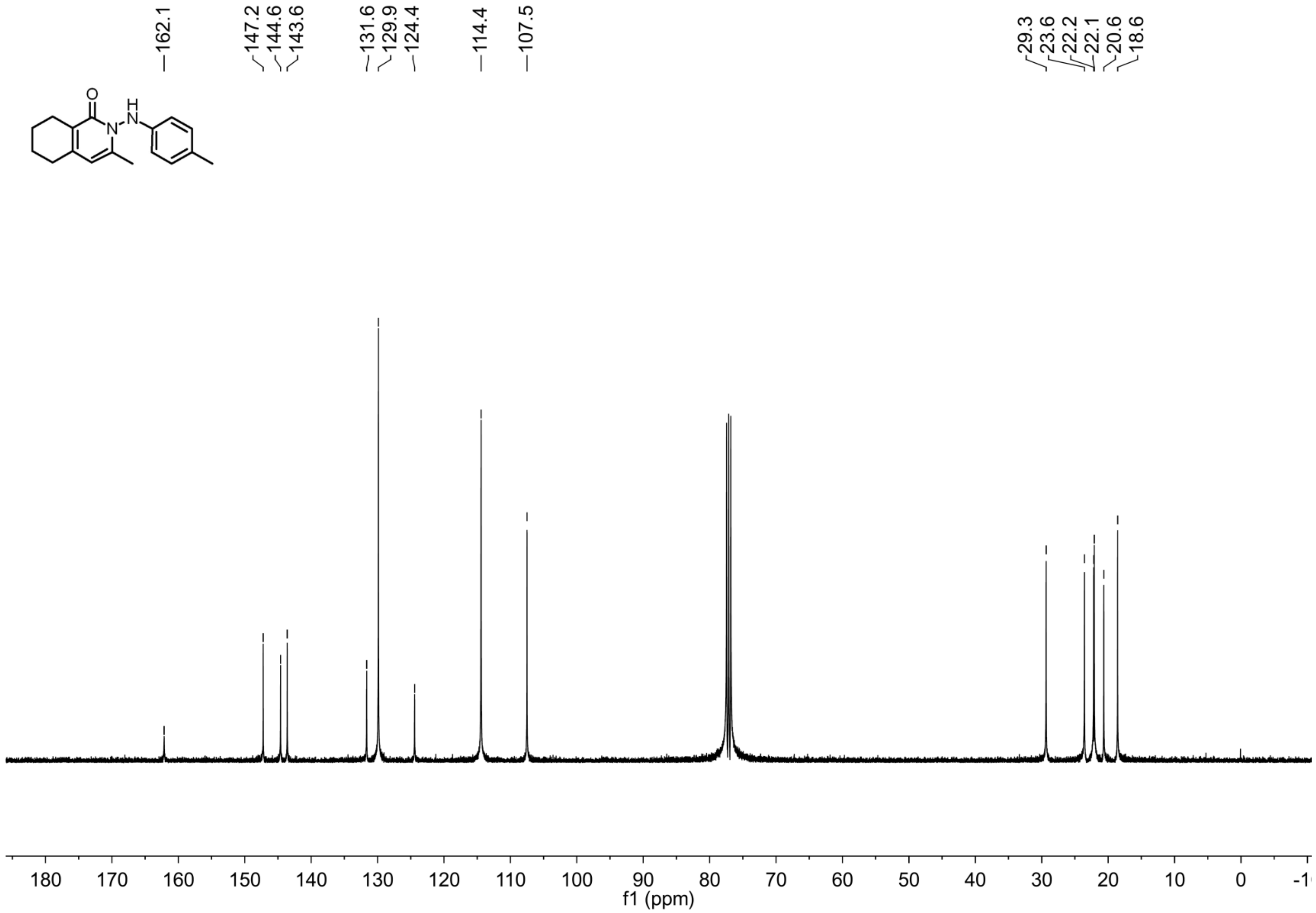

Supplementary Figure 84 ^{13}C NMR Spectrum of Compound **22**

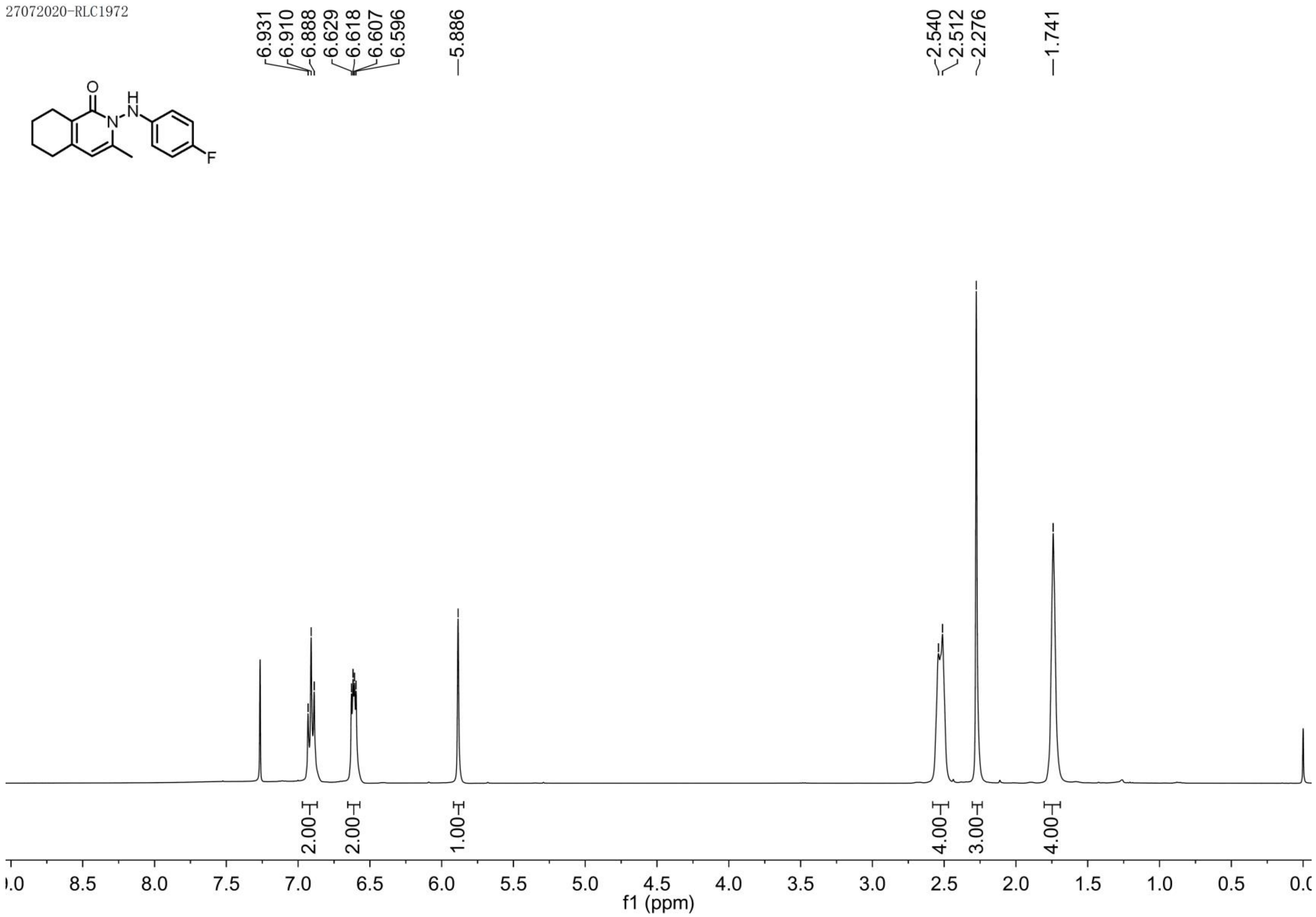

Supplementary Figure 85 ^{1}H NMR Spectrum of Compound **23**

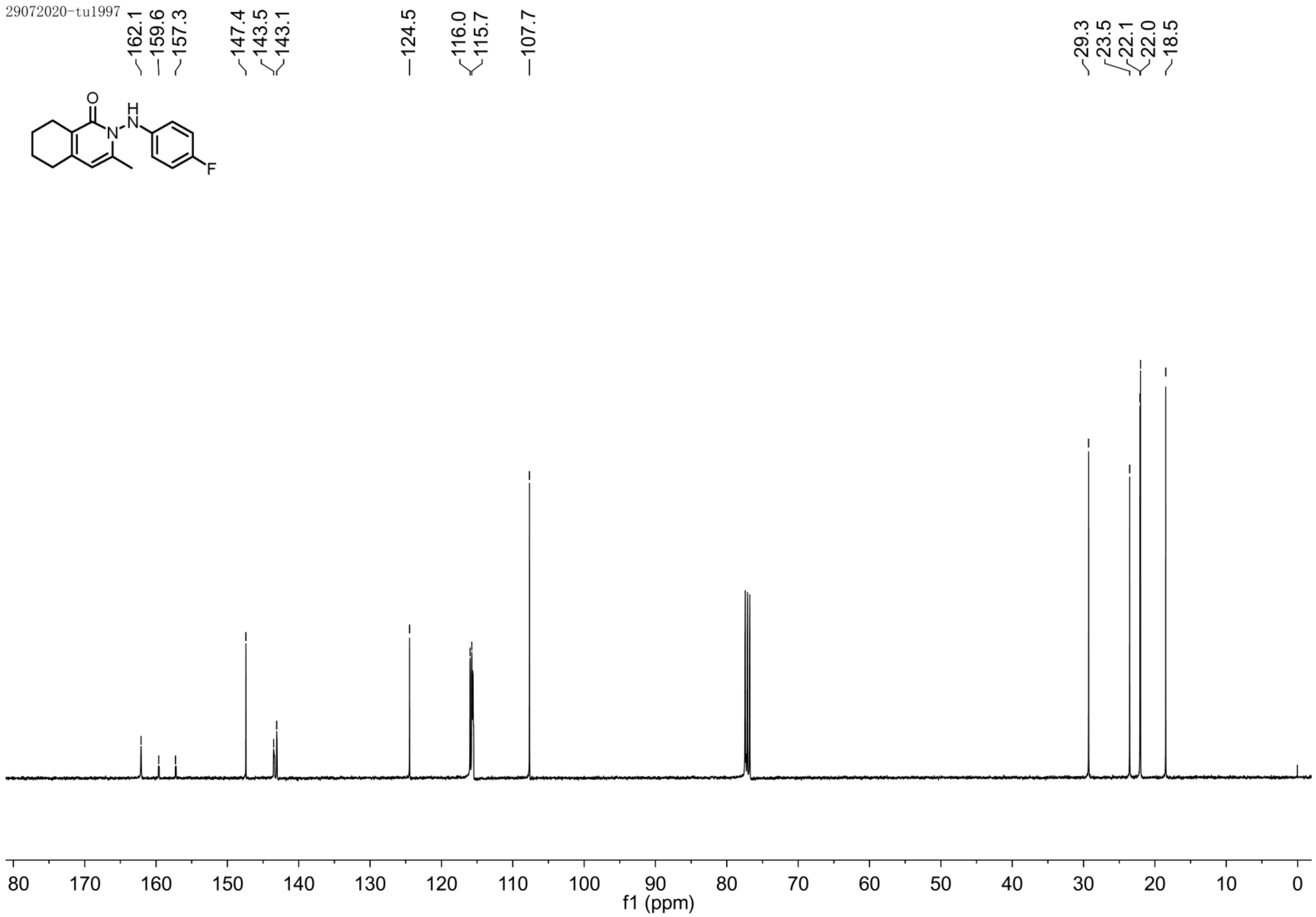

Supplementary Figure 86 ^{13}C NMR Spectrum of Compound **23**

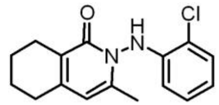

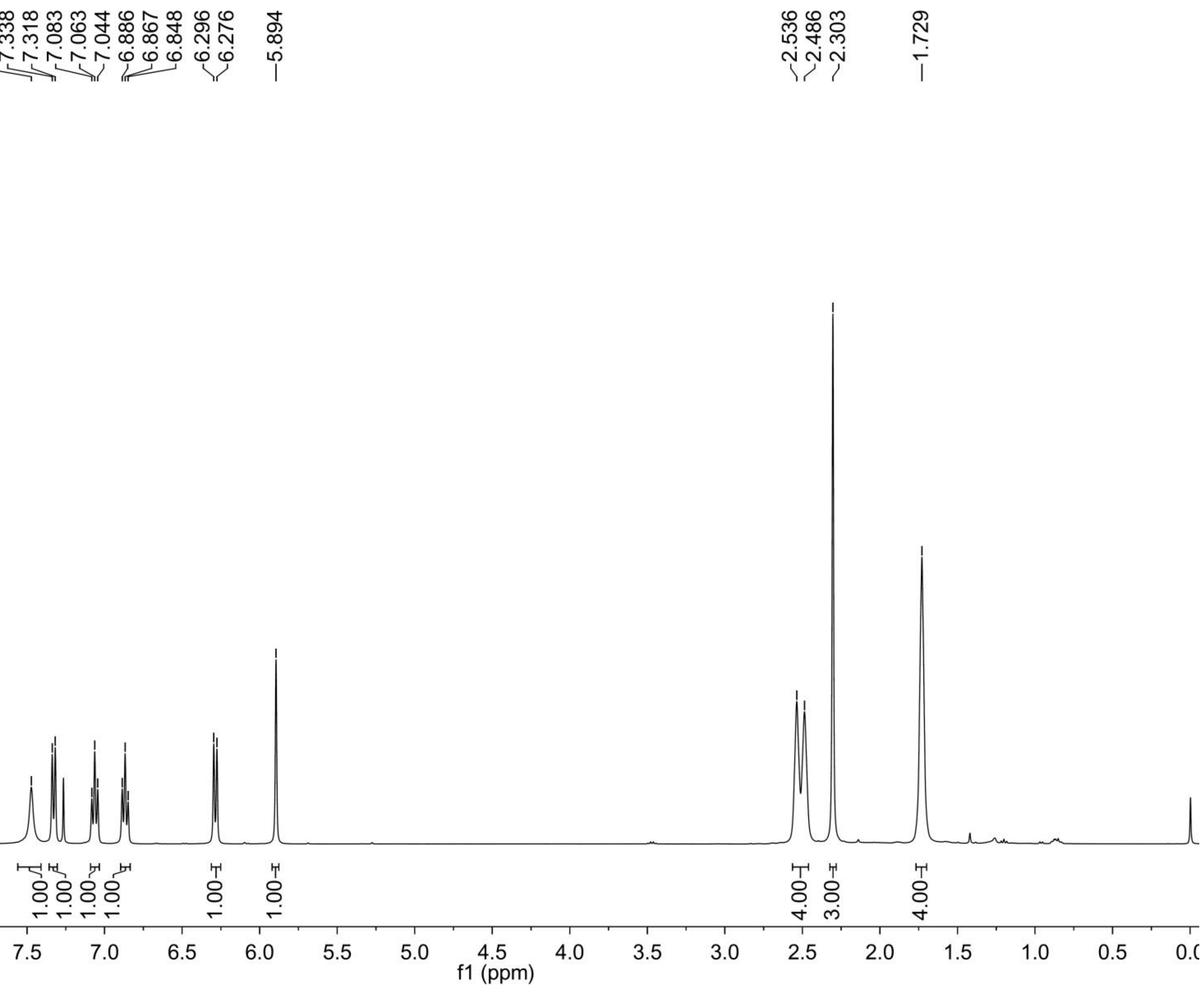

Supplementary Figure 87 ^{1}H NMR Spectrum of Compound **24**

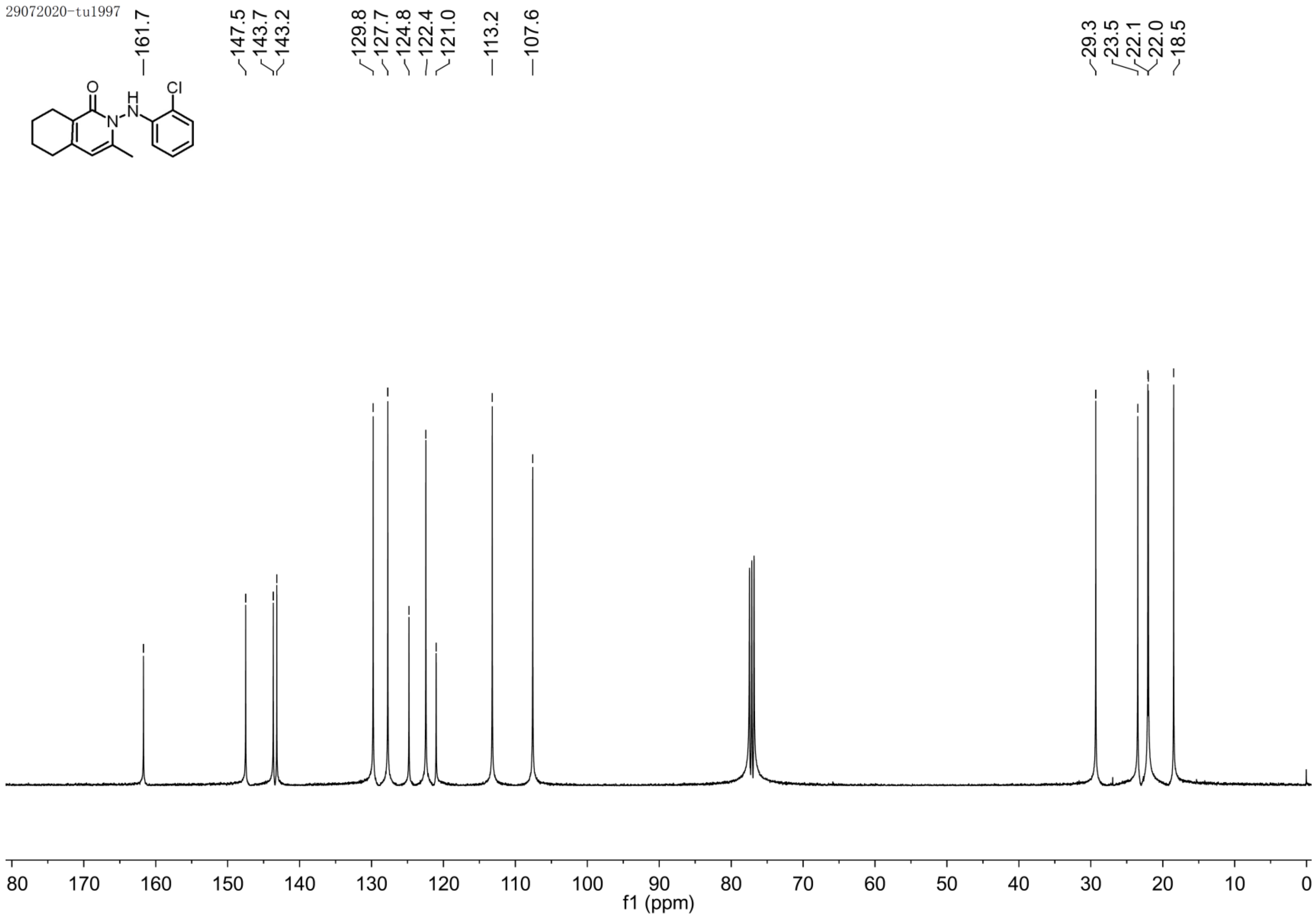

Supplementary Figure 88 ^{13}C NMR Spectrum of Compound **24**

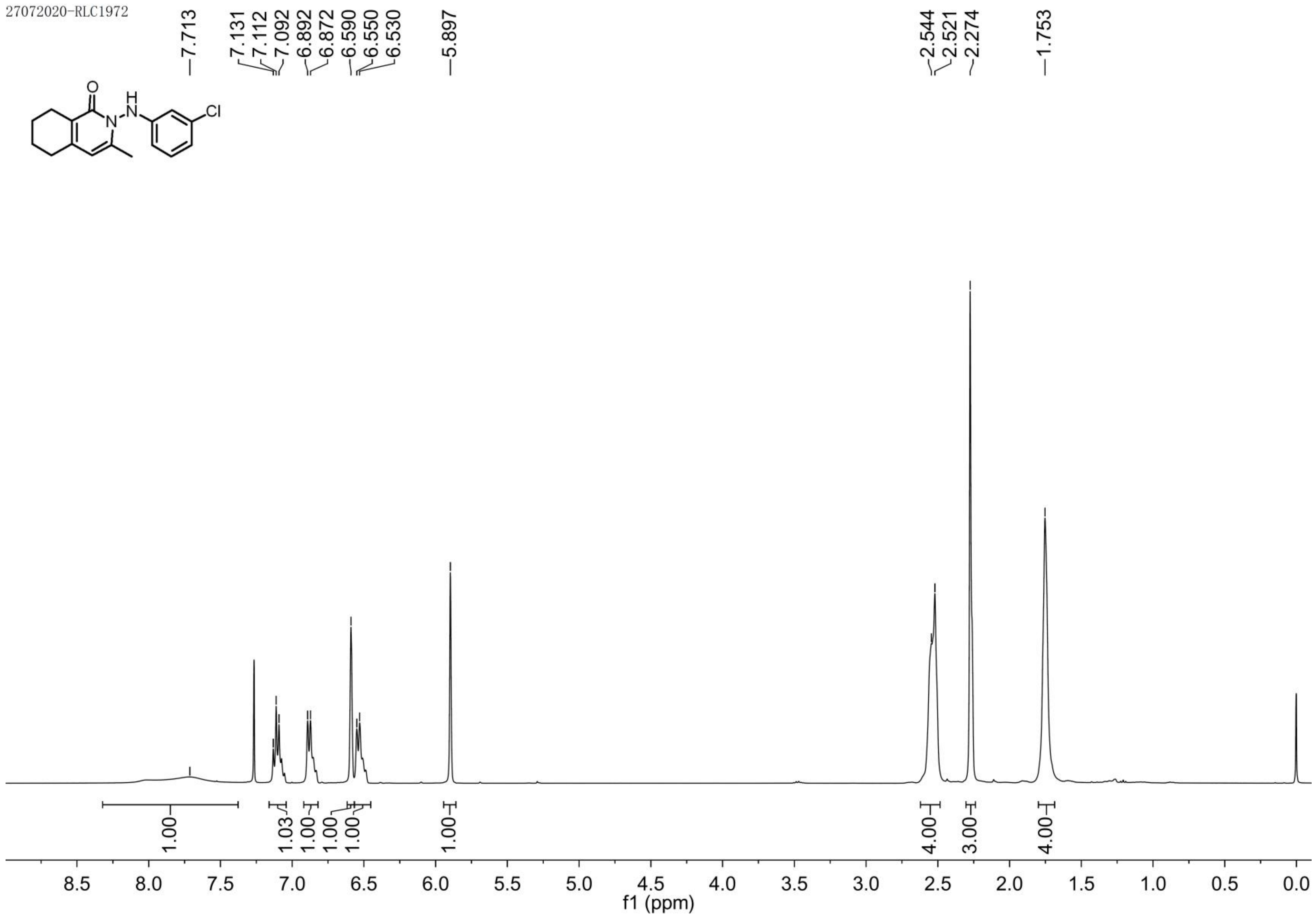

Supplementary Figure 89 ^{1}H NMR Spectrum of Compound **25**

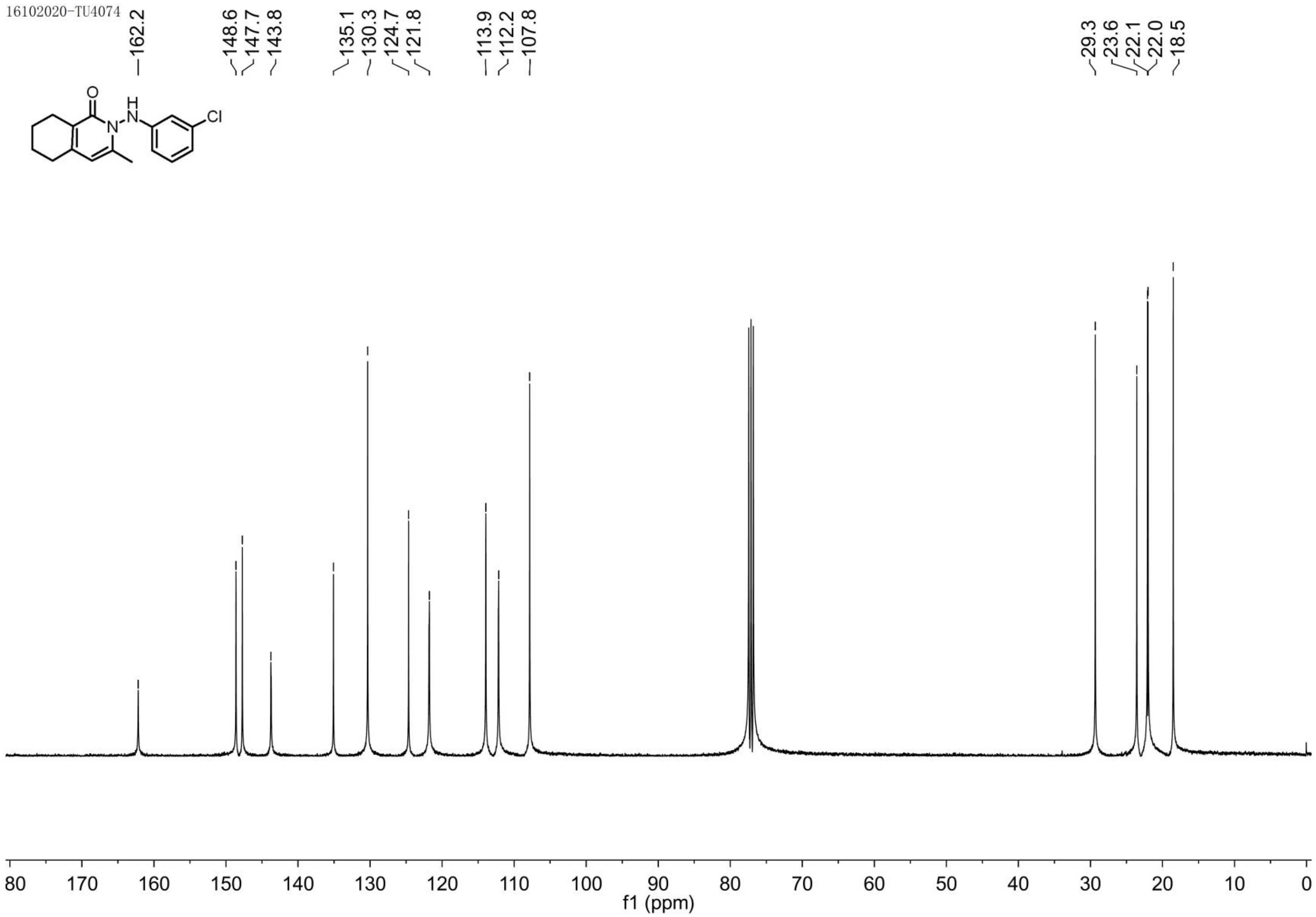

Supplementary Figure 90 ^{13}C NMR Spectrum of Compound **25**

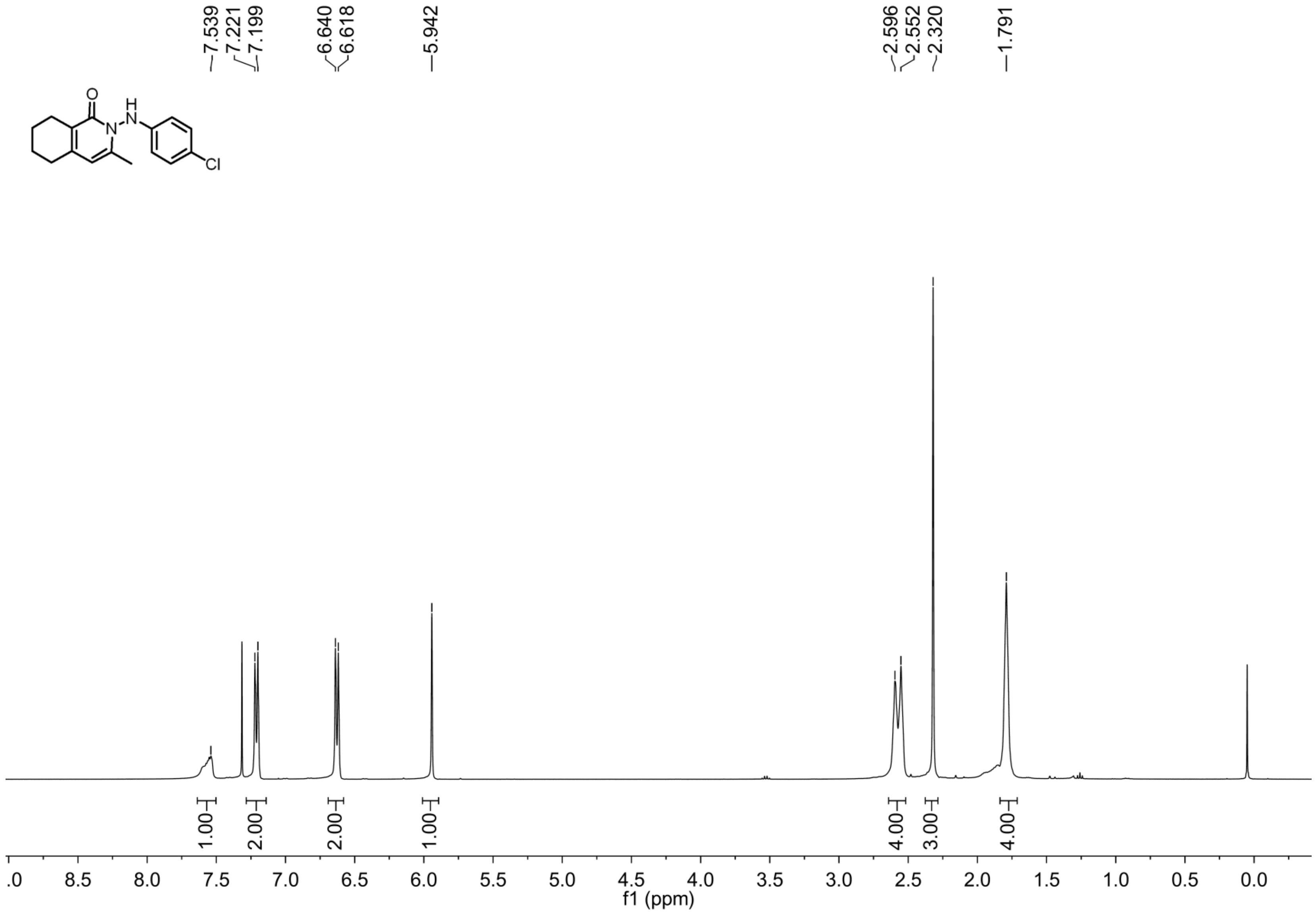

Supplementary Figure 91 ^{1}H NMR Spectrum of Compound **26**

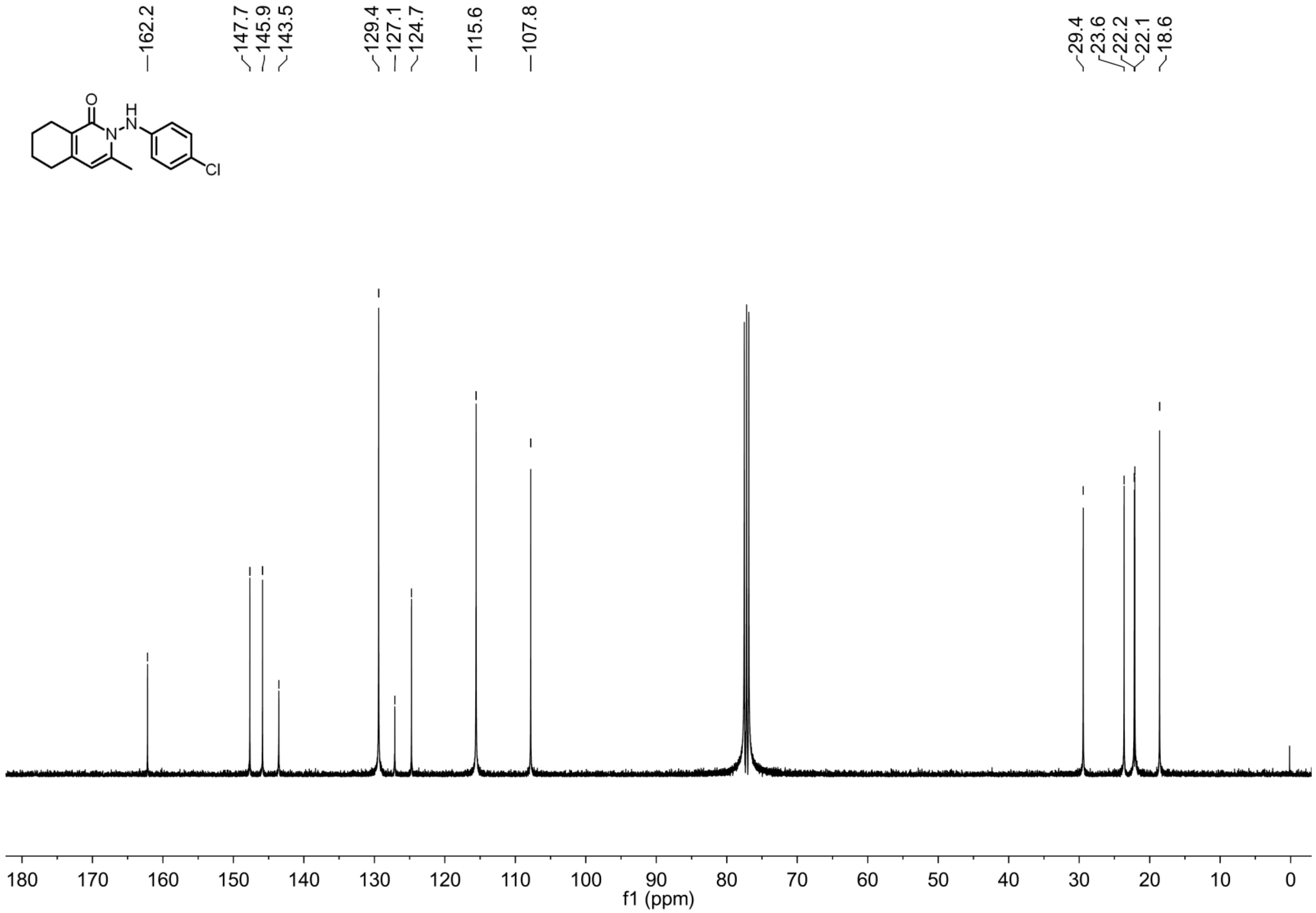

Supplementary Figure 92 ^{13}C NMR Spectrum of Compound **26**

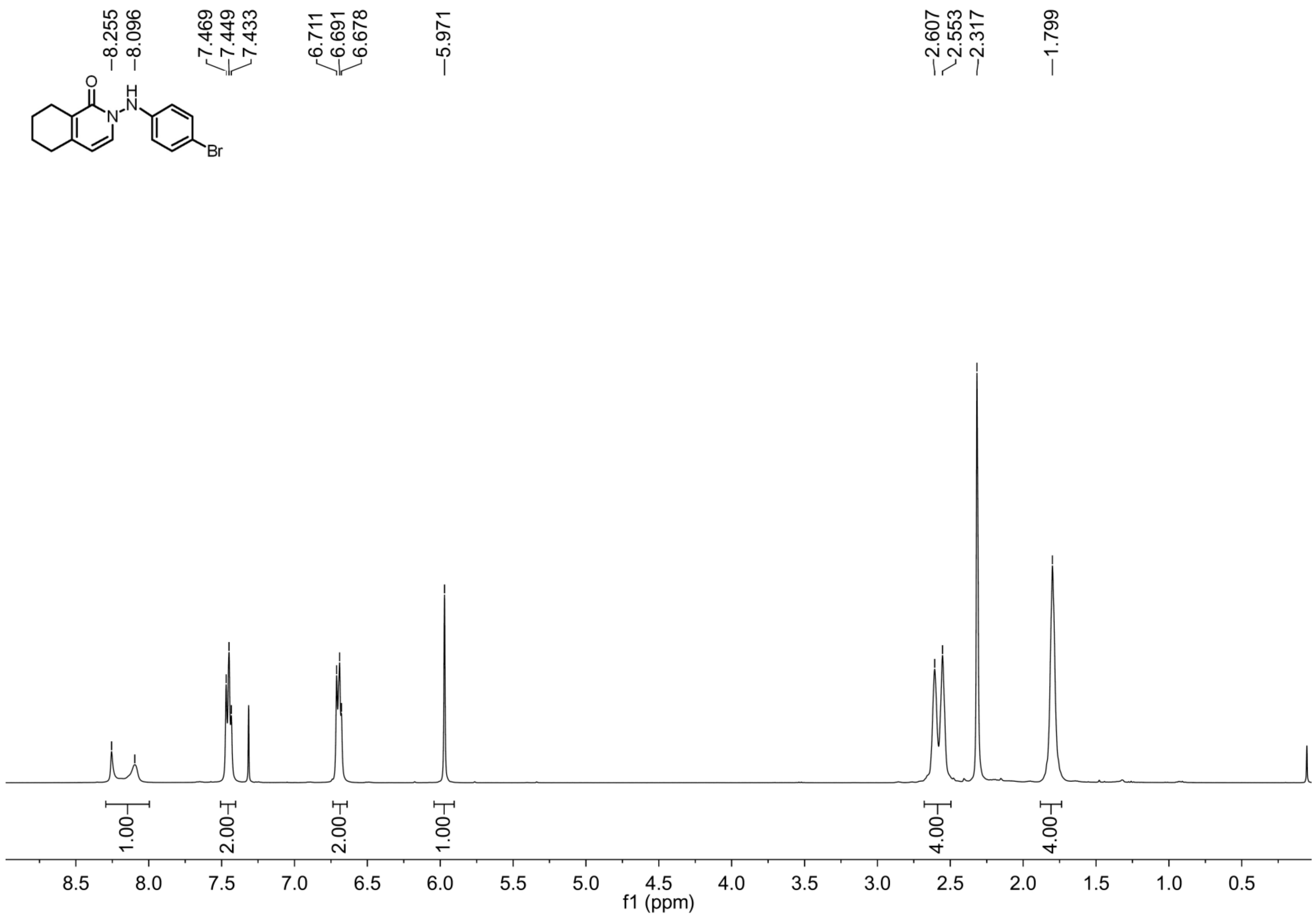

Supplementary Figure 93 ^{1}H NMR Spectrum of Compound **27**

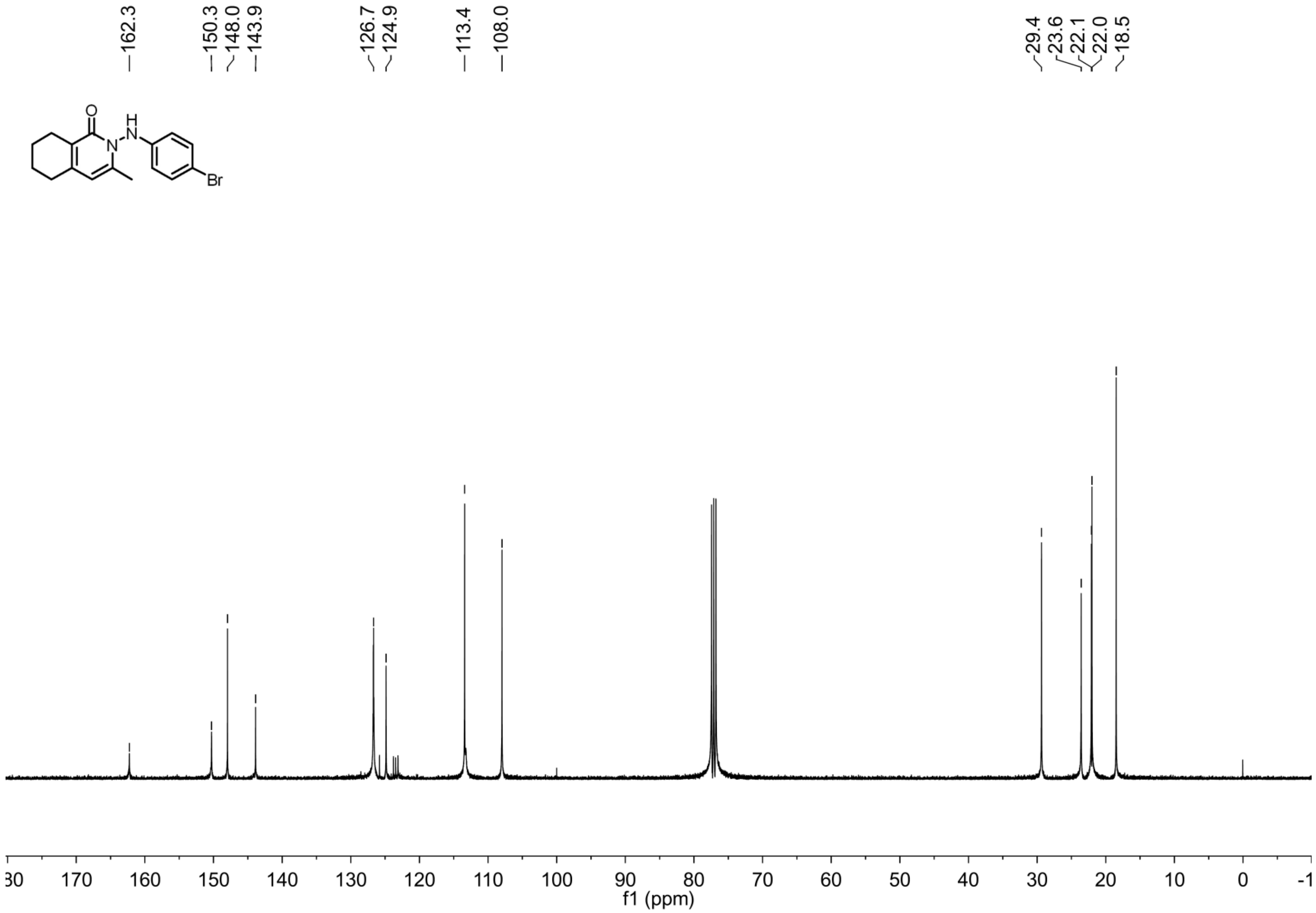

Supplementary Figure 94 ^{13}C NMR Spectrum of Compound **27**

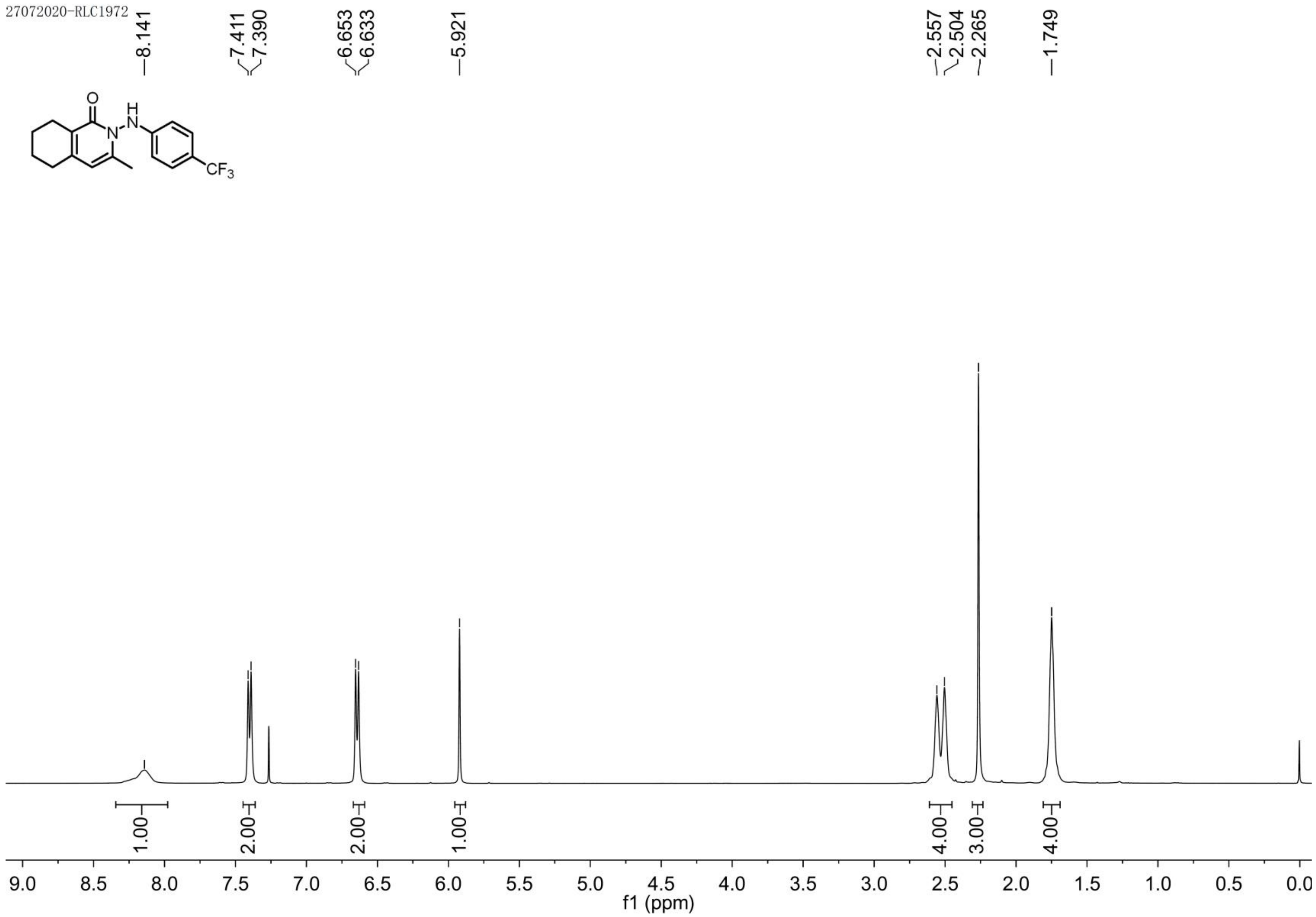

Supplementary Figure 95 ^{1}H NMR Spectrum of Compound **28**

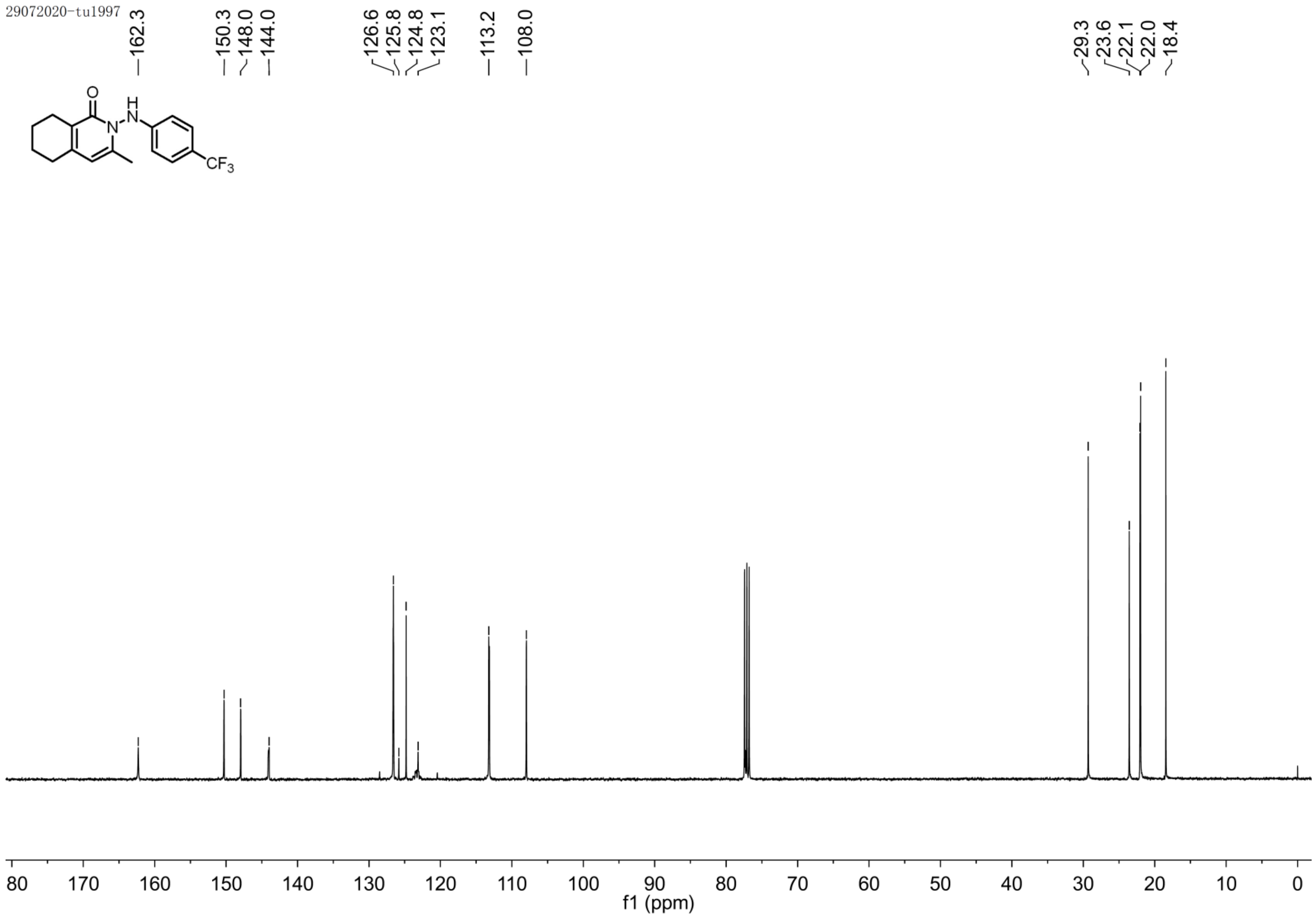

Supplementary Figure 96 ^{13}C NMR Spectrum of Compound **28**

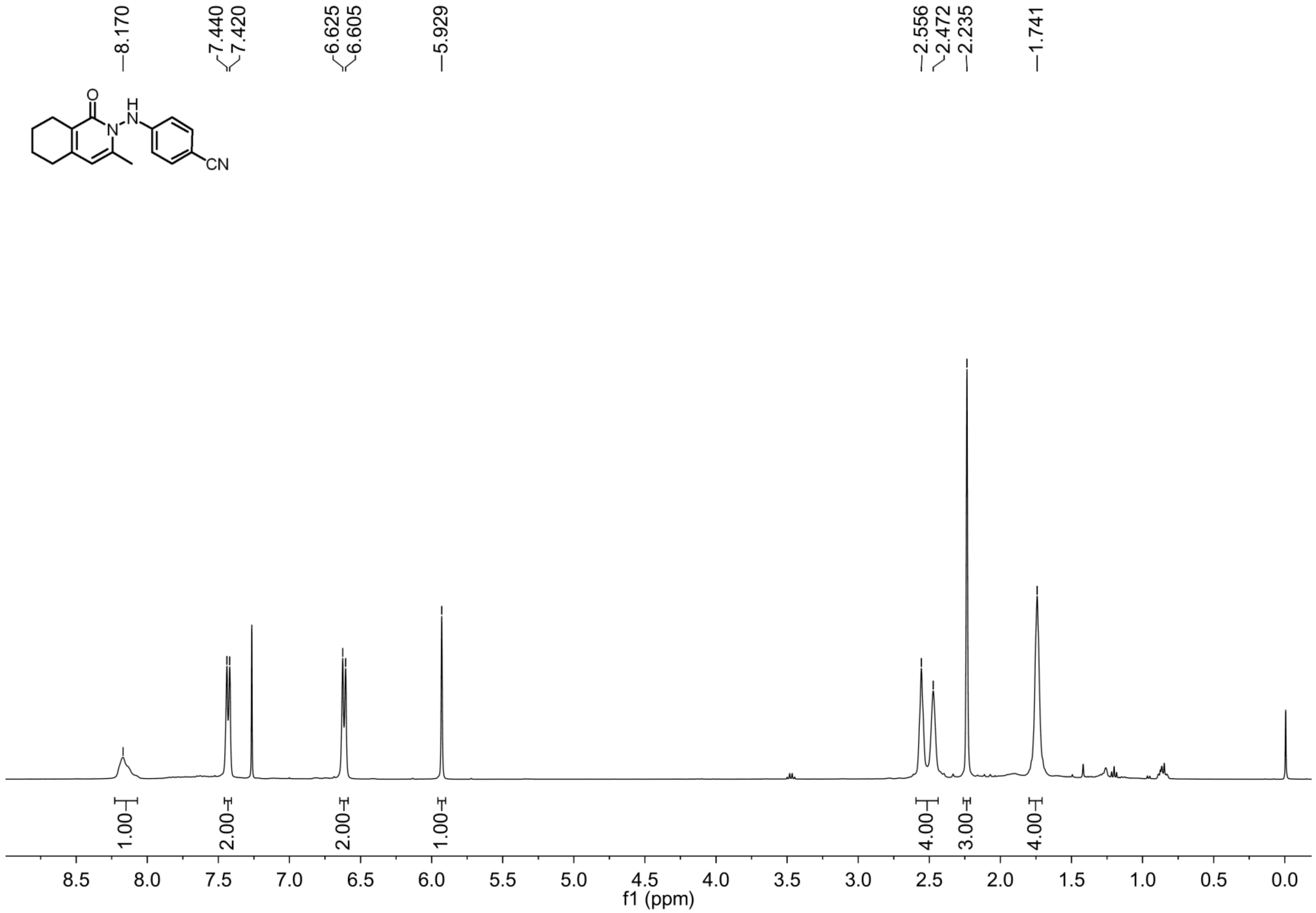

Supplementary Figure 97 ^{1}H NMR Spectrum of Compound **29**

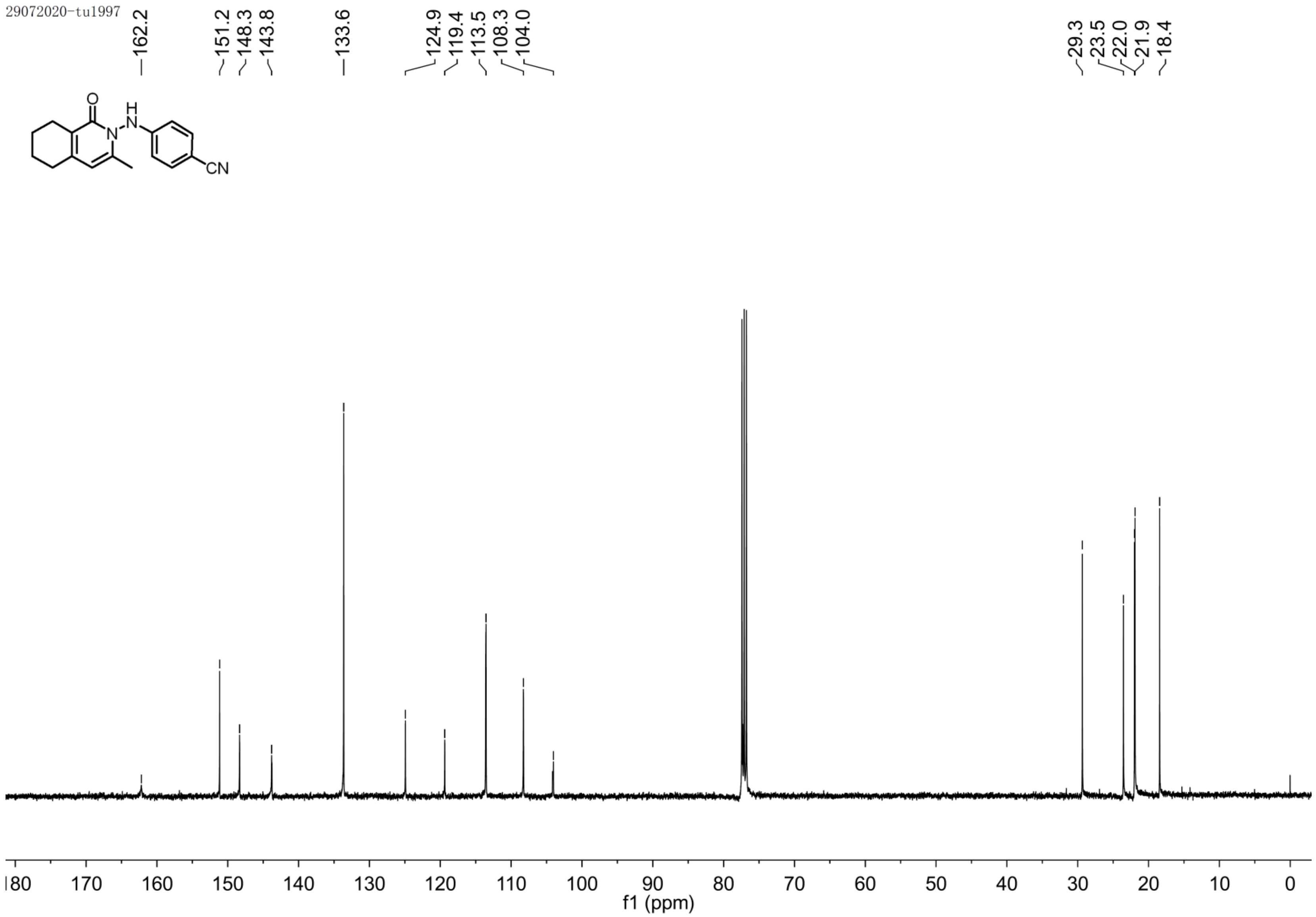

Supplementary Figure 98 ^{13}C NMR Spectrum of Compound **29**

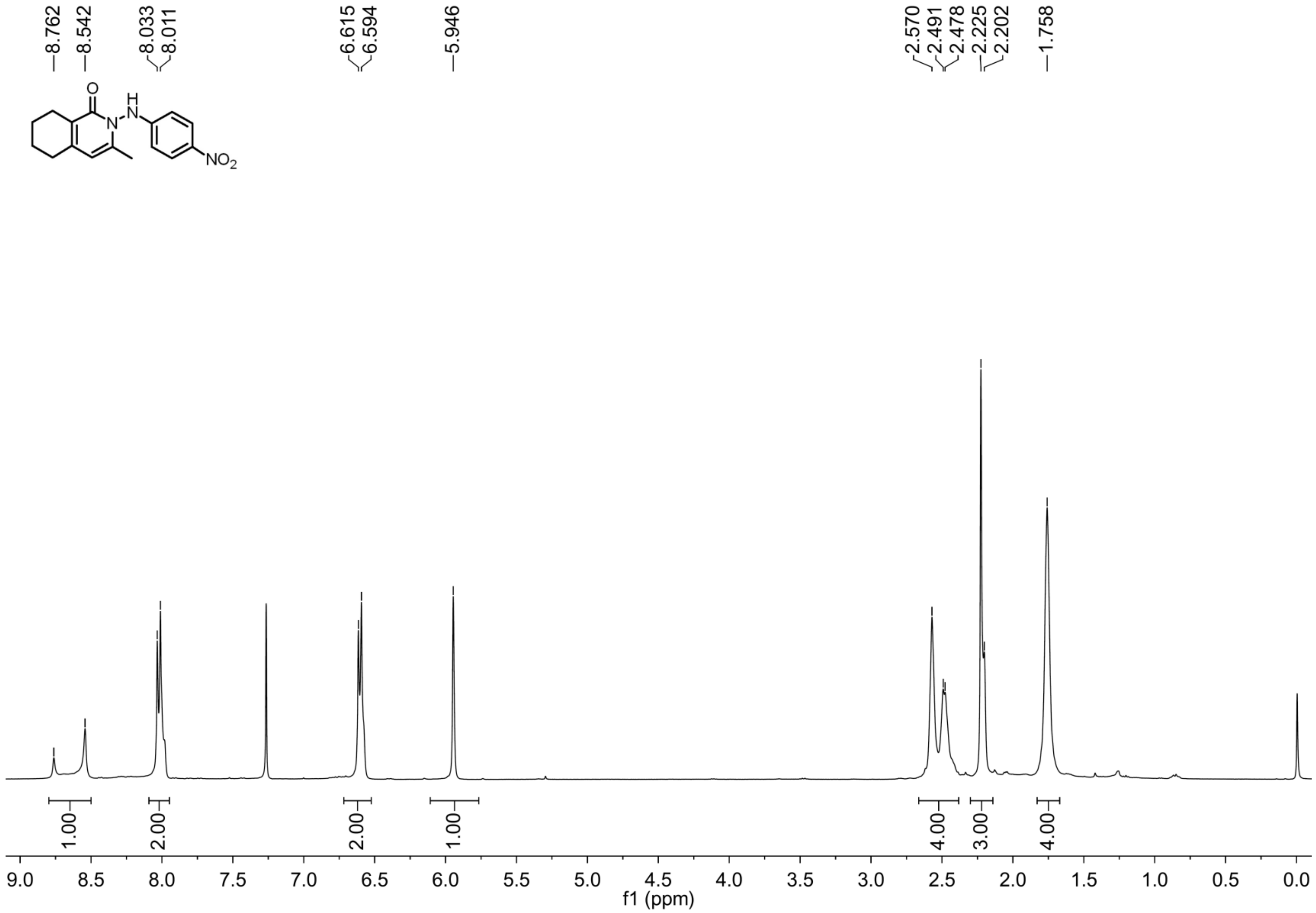

Supplementary Figure 99 ^{1}H NMR Spectrum of Compound **30**

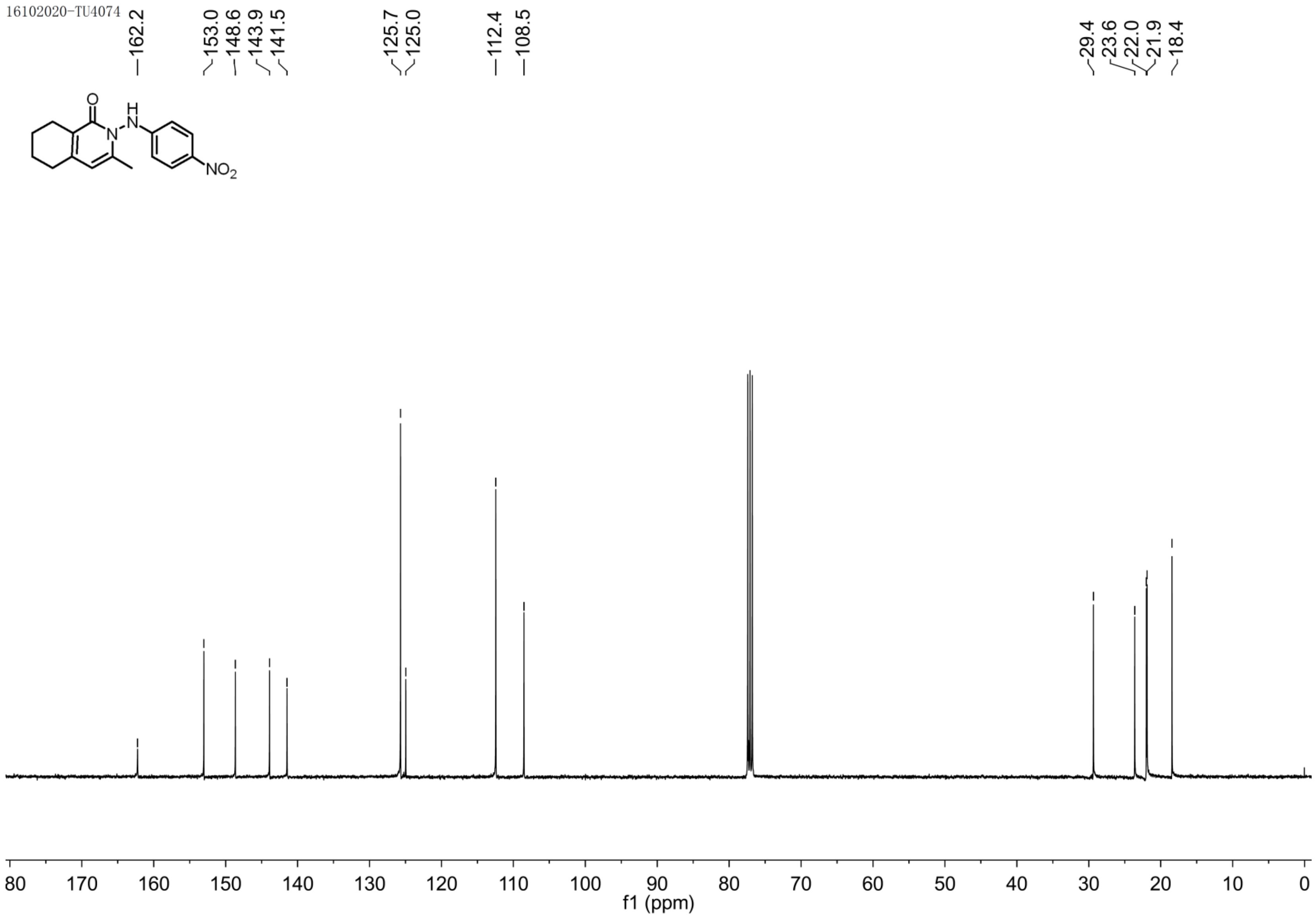

Supplementary Figure 100 ^{13}C NMR Spectrum of Compound **30**

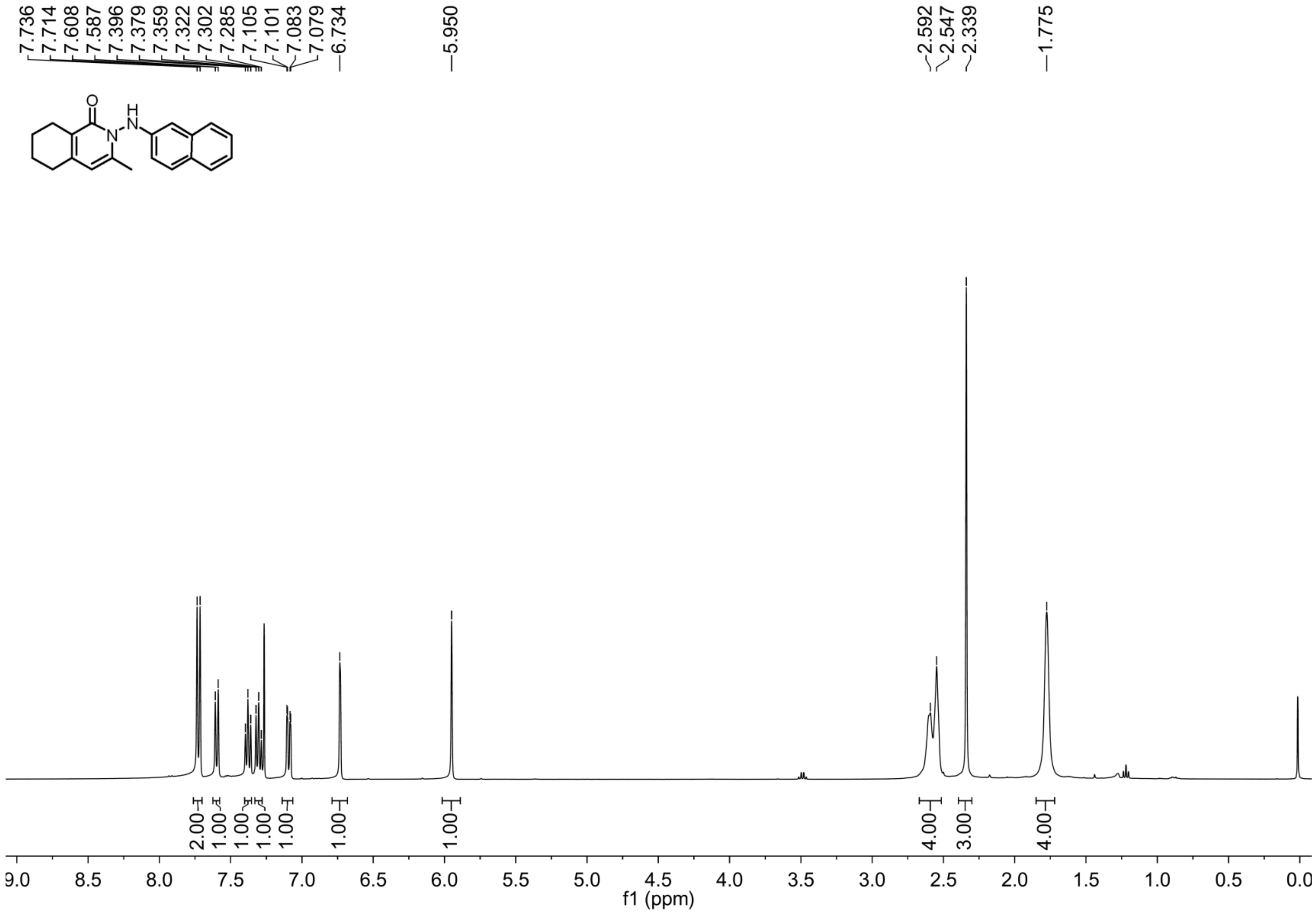

Supplementary Figure 101 ^{1}H NMR Spectrum of Compound **31**

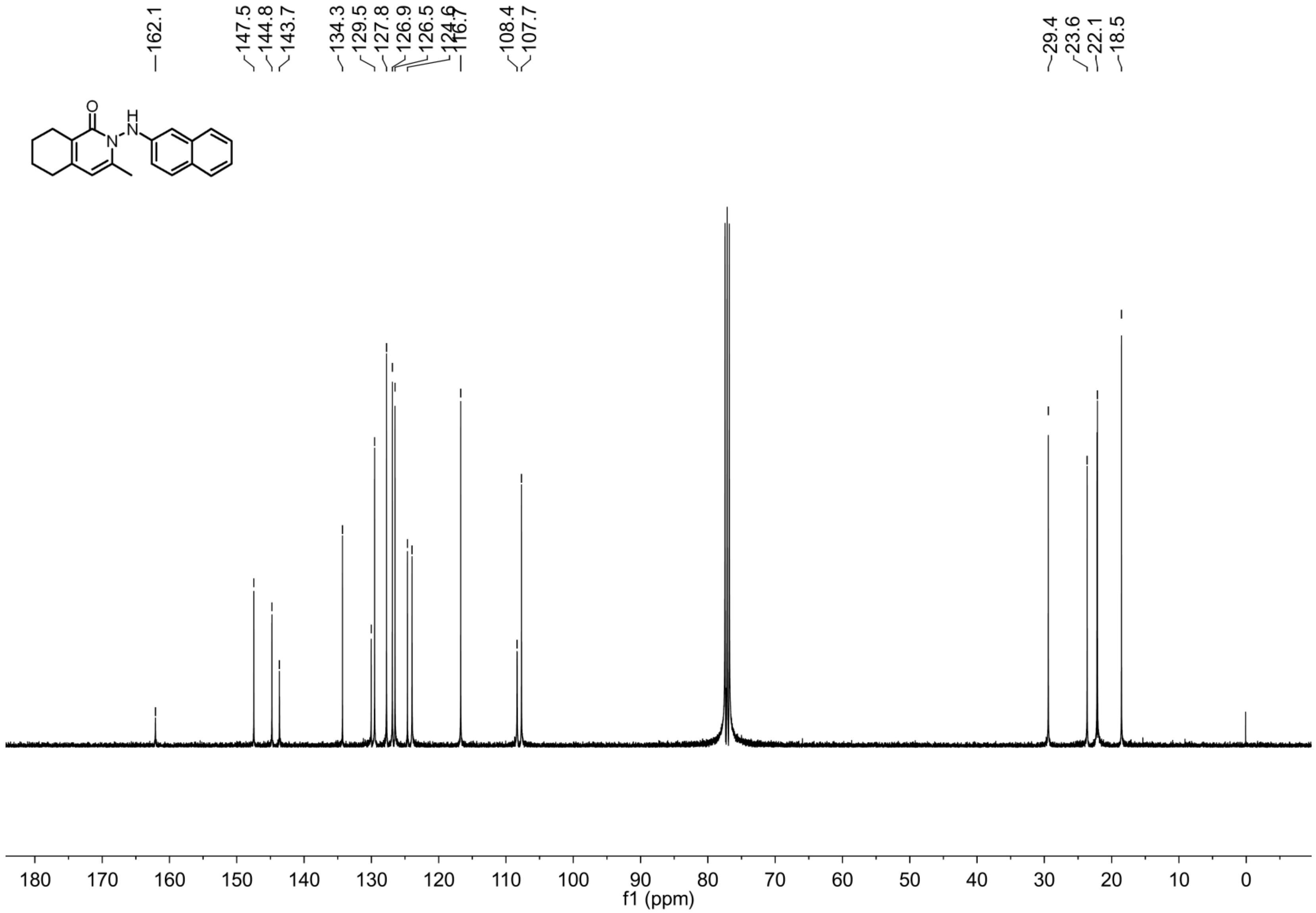

Supplementary Figure 102 ^{13}C NMR Spectrum of Compound **31**

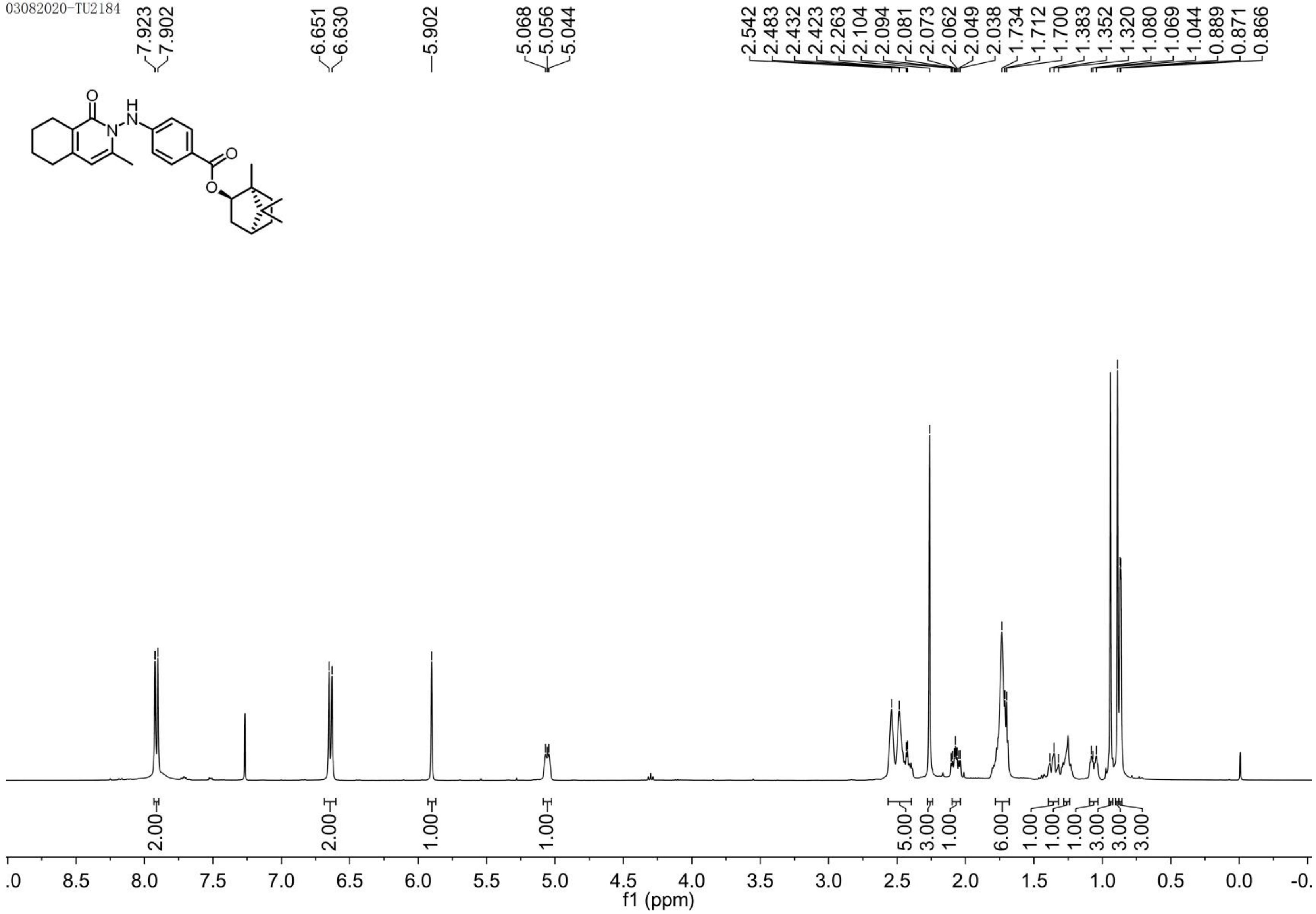

Supplementary Figure 103 ^{1}H NMR Spectrum of Compound **32**

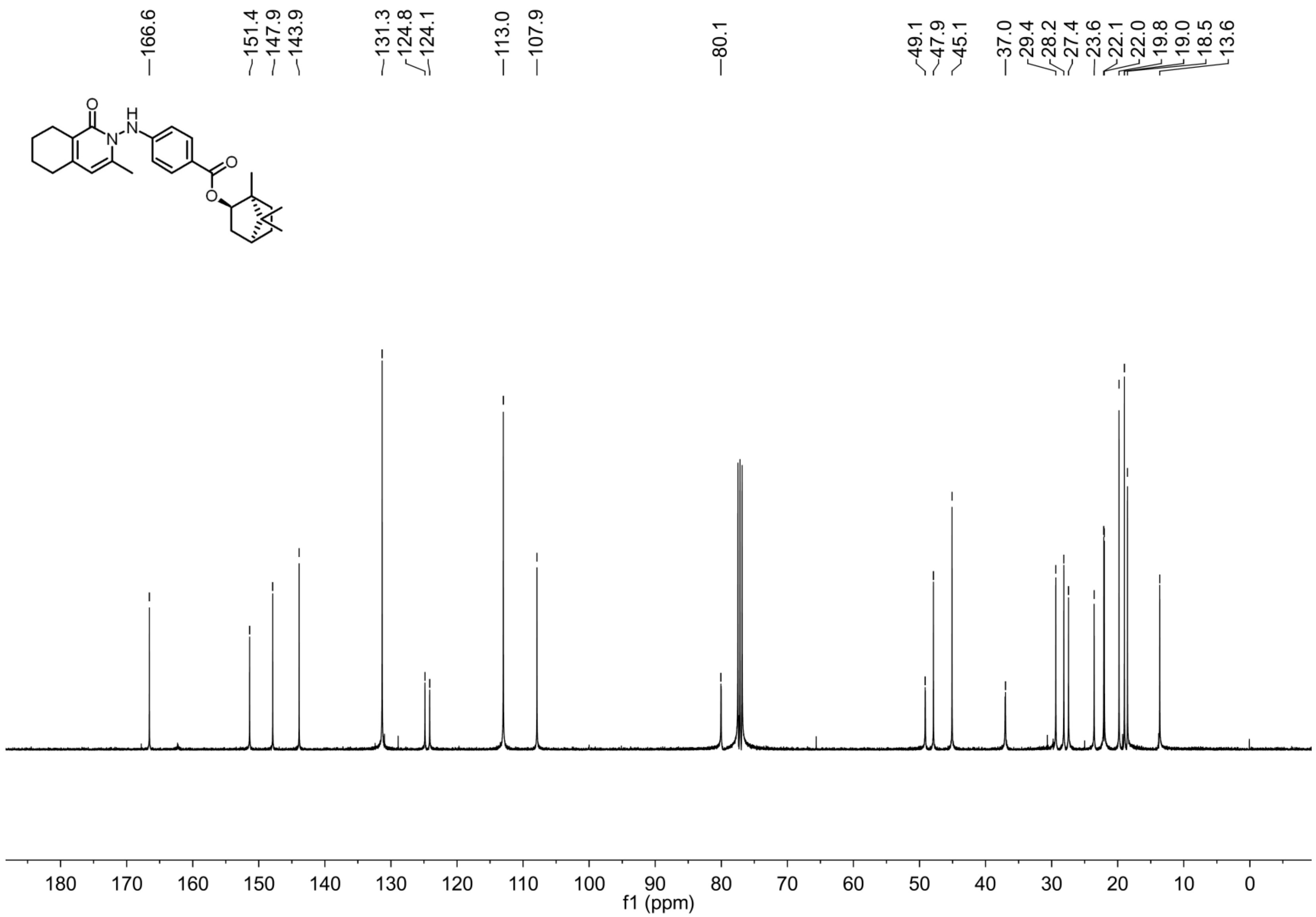

Supplementary Figure 104 ^{13}C NMR Spectrum of Compound **32**

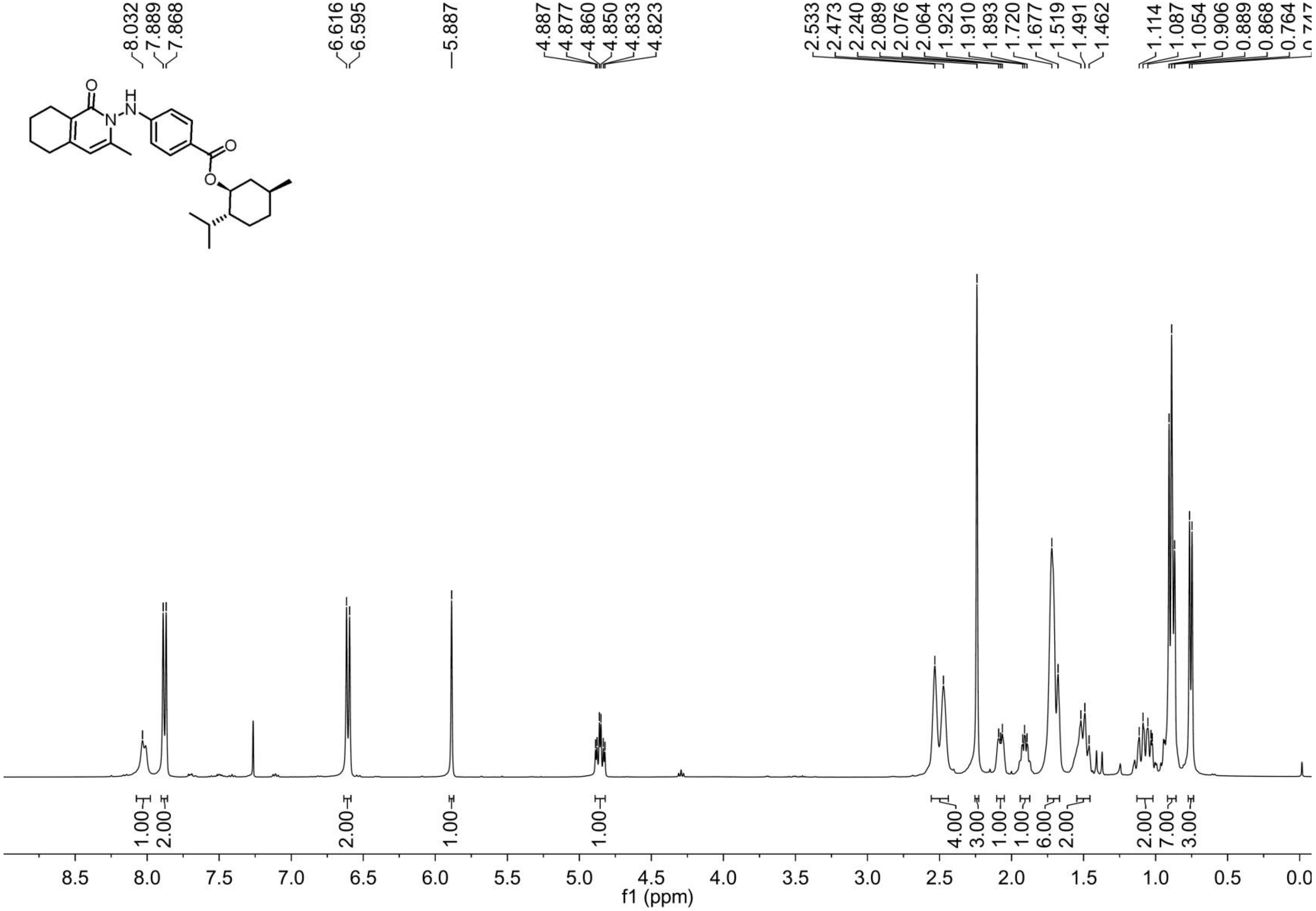

Supplementary Figure 105 ^{1}H NMR Spectrum of Compound **33**

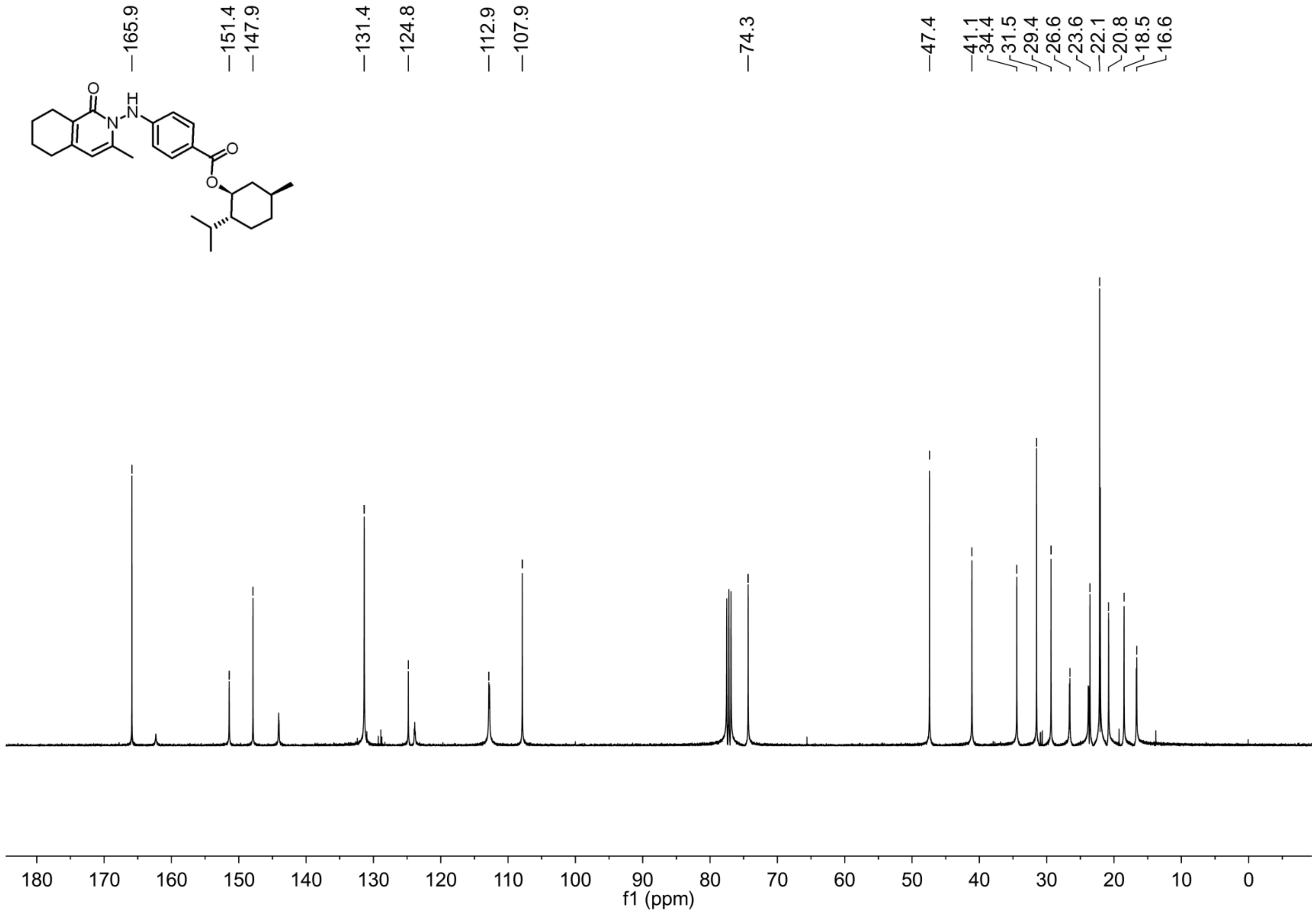

Supplementary Figure 106 ^{13}C NMR Spectrum of Compound **33**

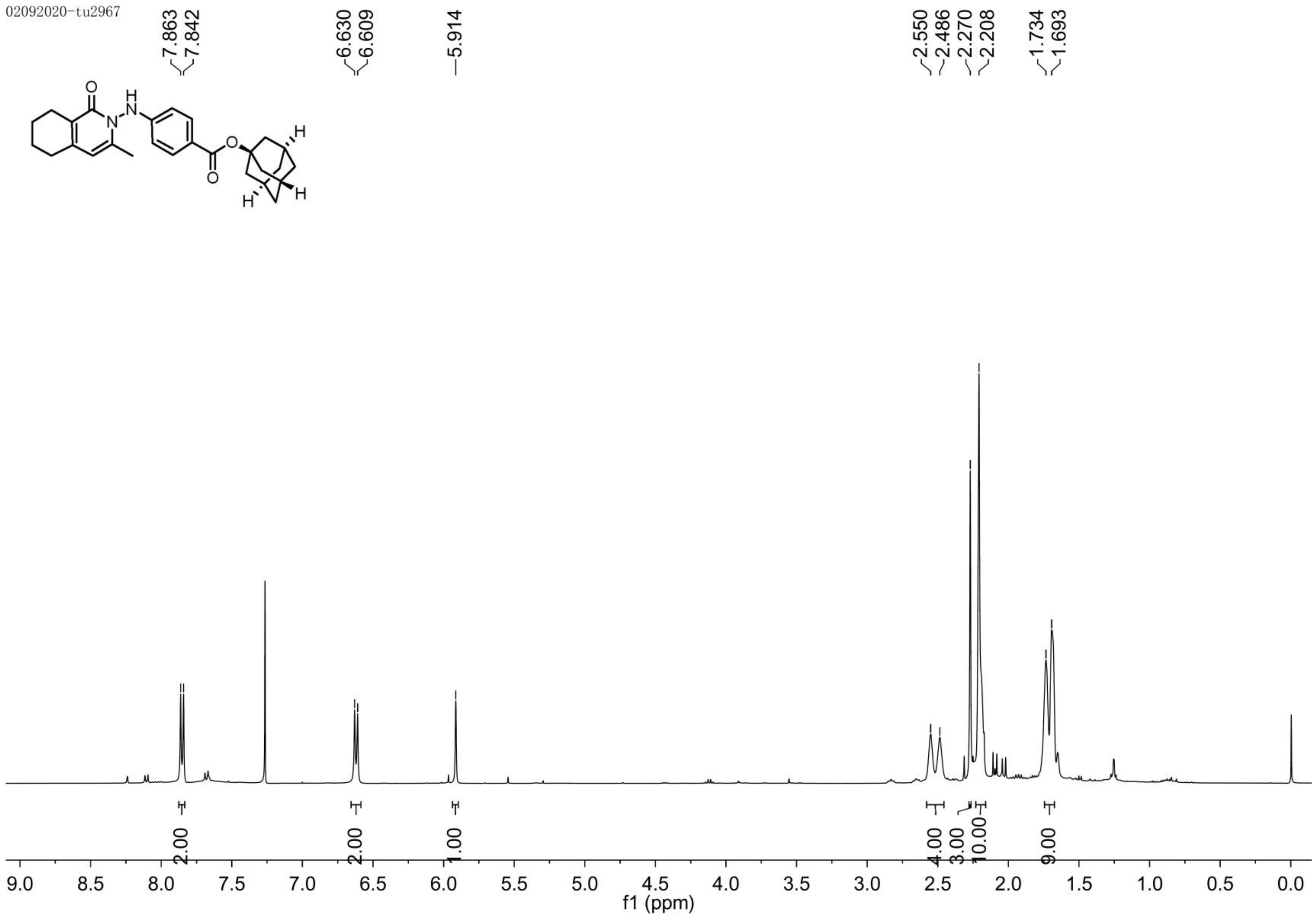

Supplementary Figure 107 ^{1}H NMR Spectrum of Compound **34**

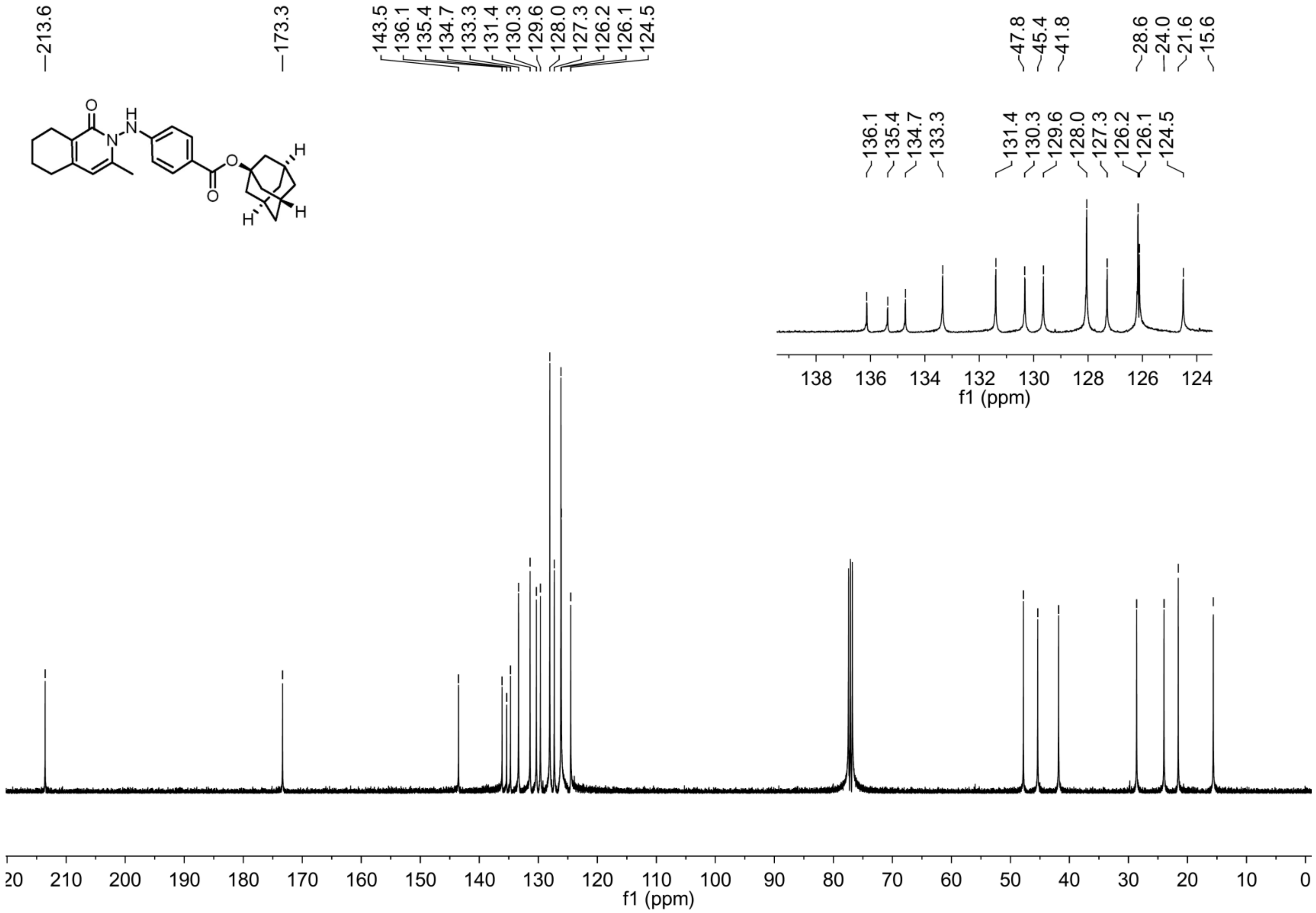

Supplementary Figure 108 ^{13}C NMR Spectrum of Compound **34**

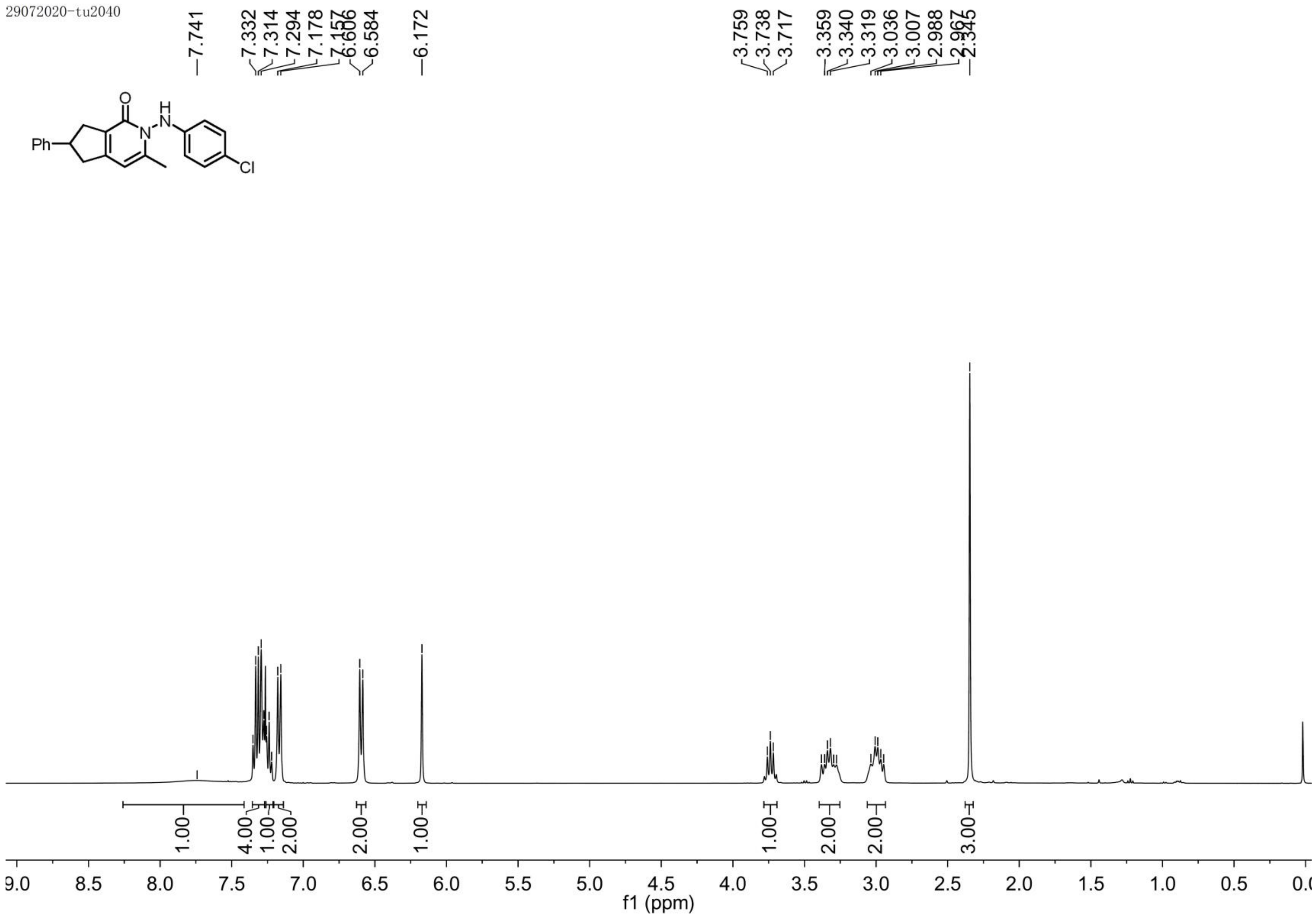

Supplementary Figure 109 ^{1}H NMR Spectrum of Compound **35**

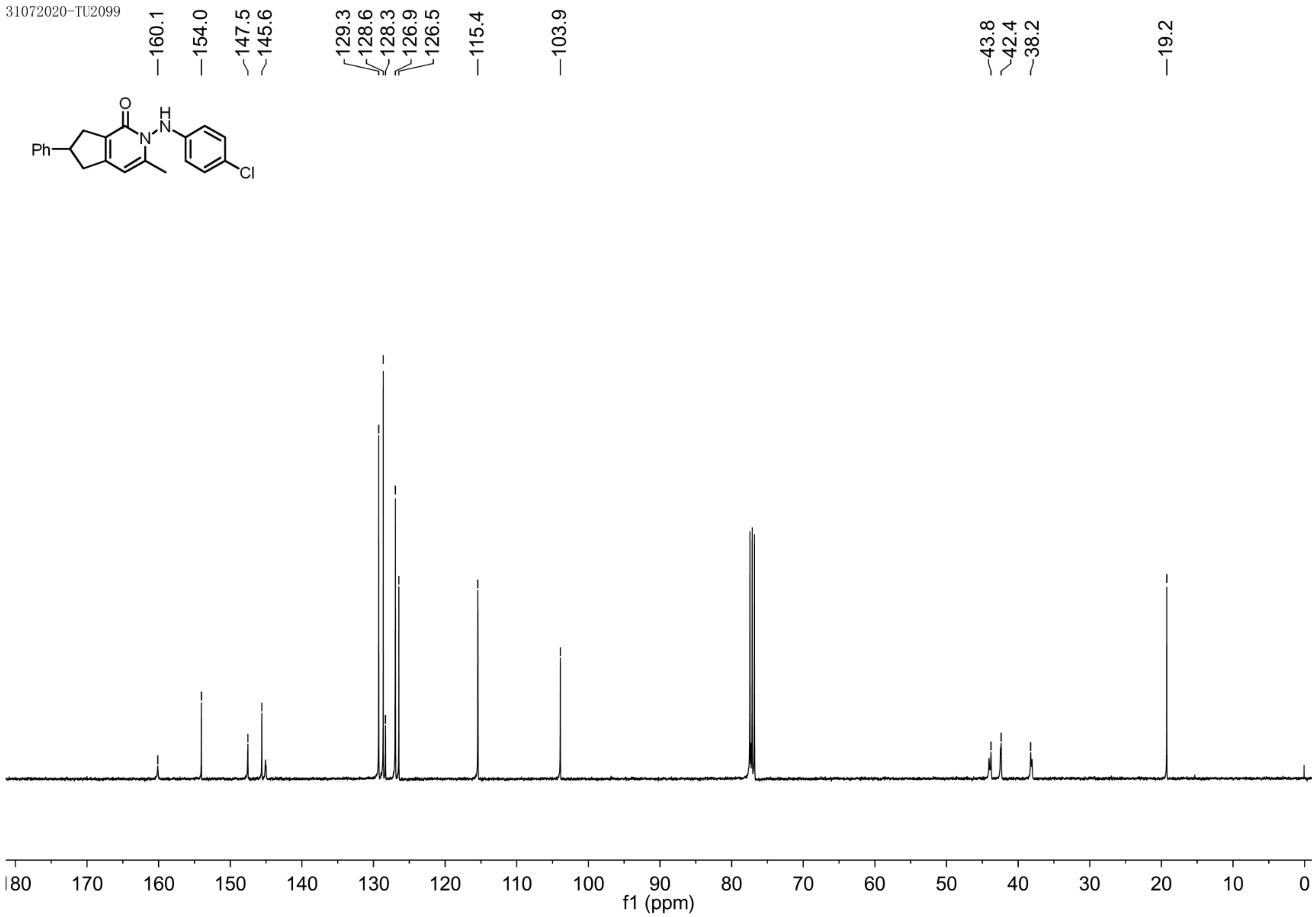

Supplementary Figure 110 ^{13}C NMR Spectrum of Compound **35**

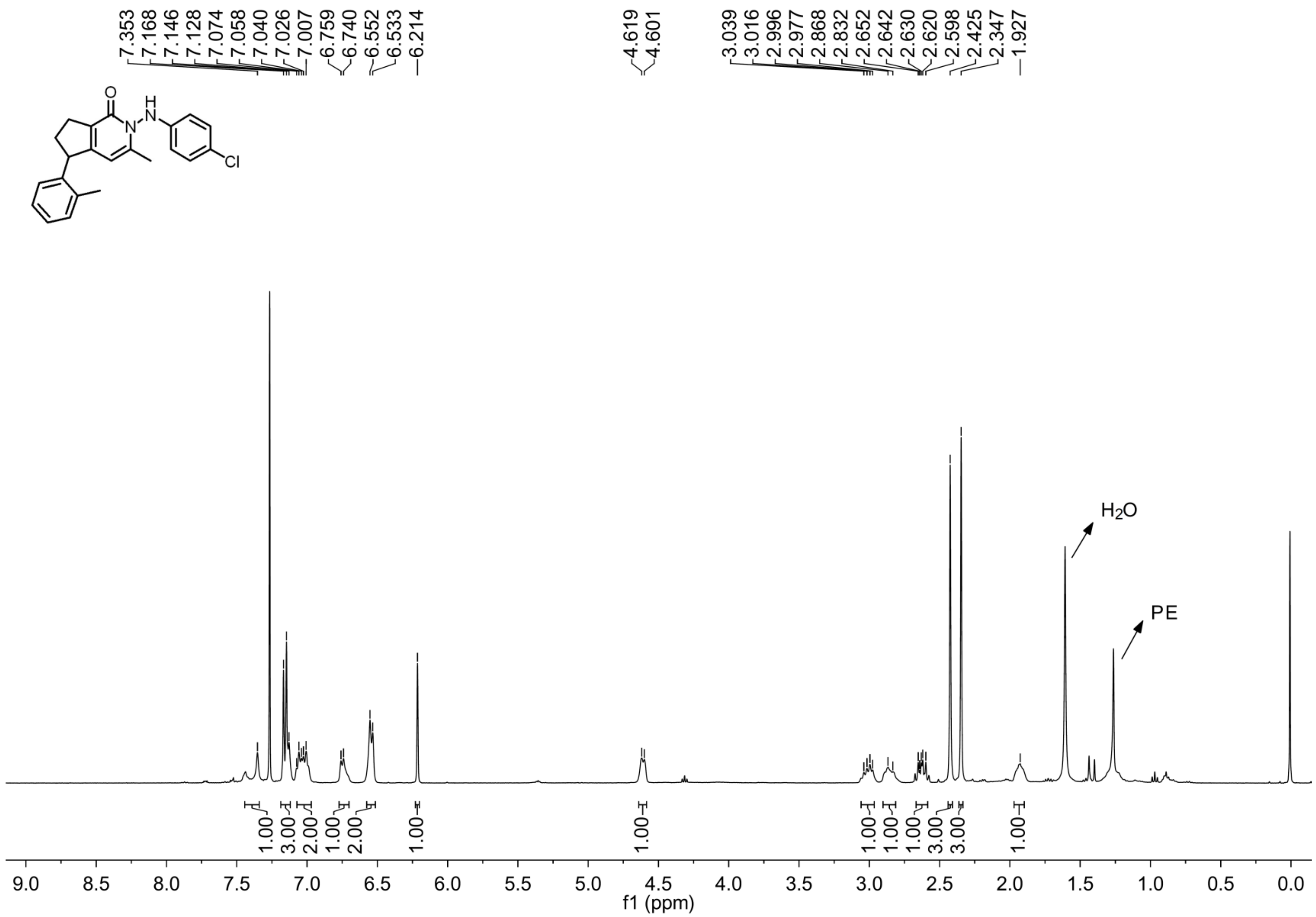

Supplementary Figure 111 ^{1}H NMR Spectrum of Compound **36**

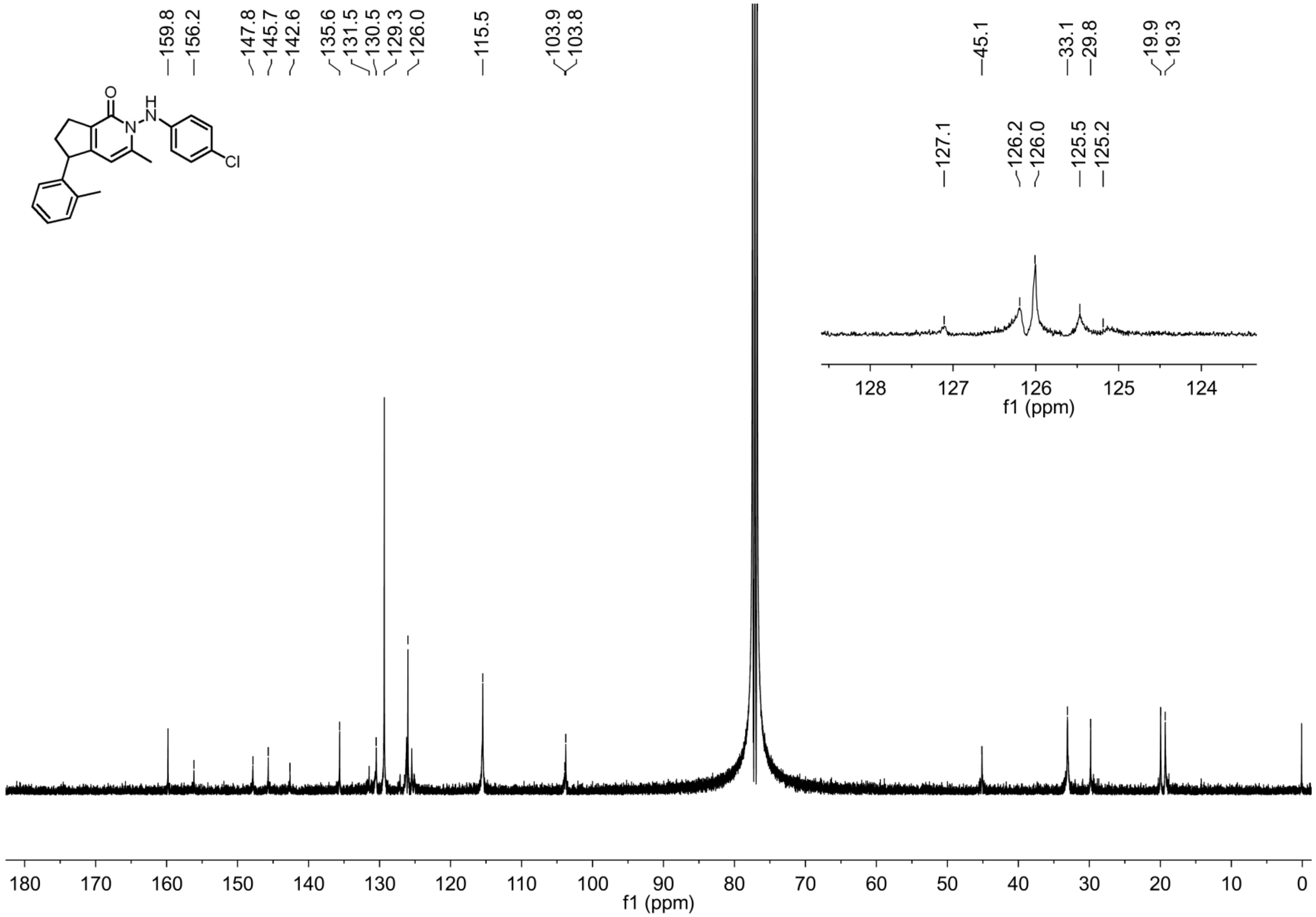

Supplementary Figure 112 ^{13}C NMR Spectrum of Compound **36**

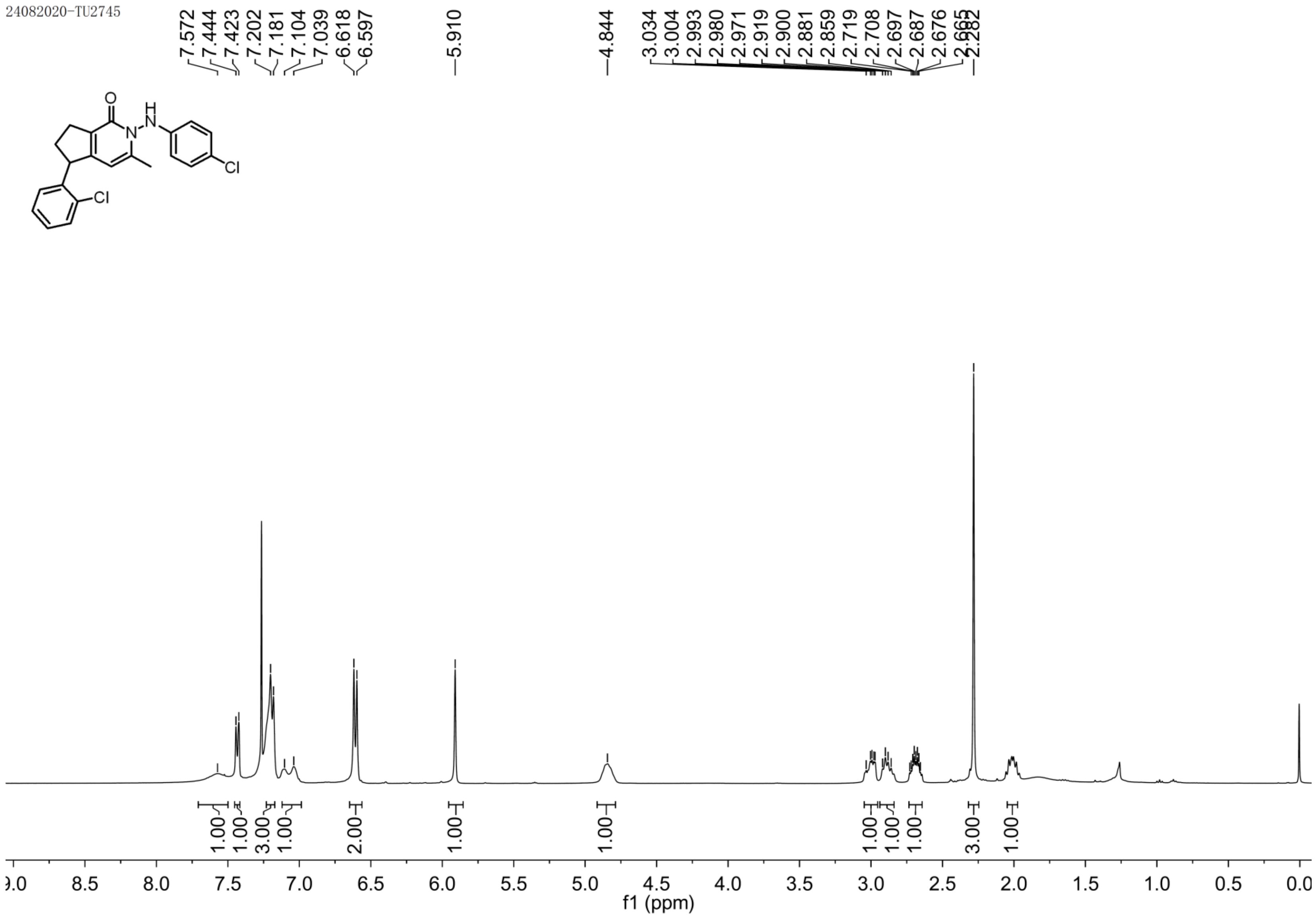

Supplementary Figure 113 ^{1}H NMR Spectrum of Compound **37**

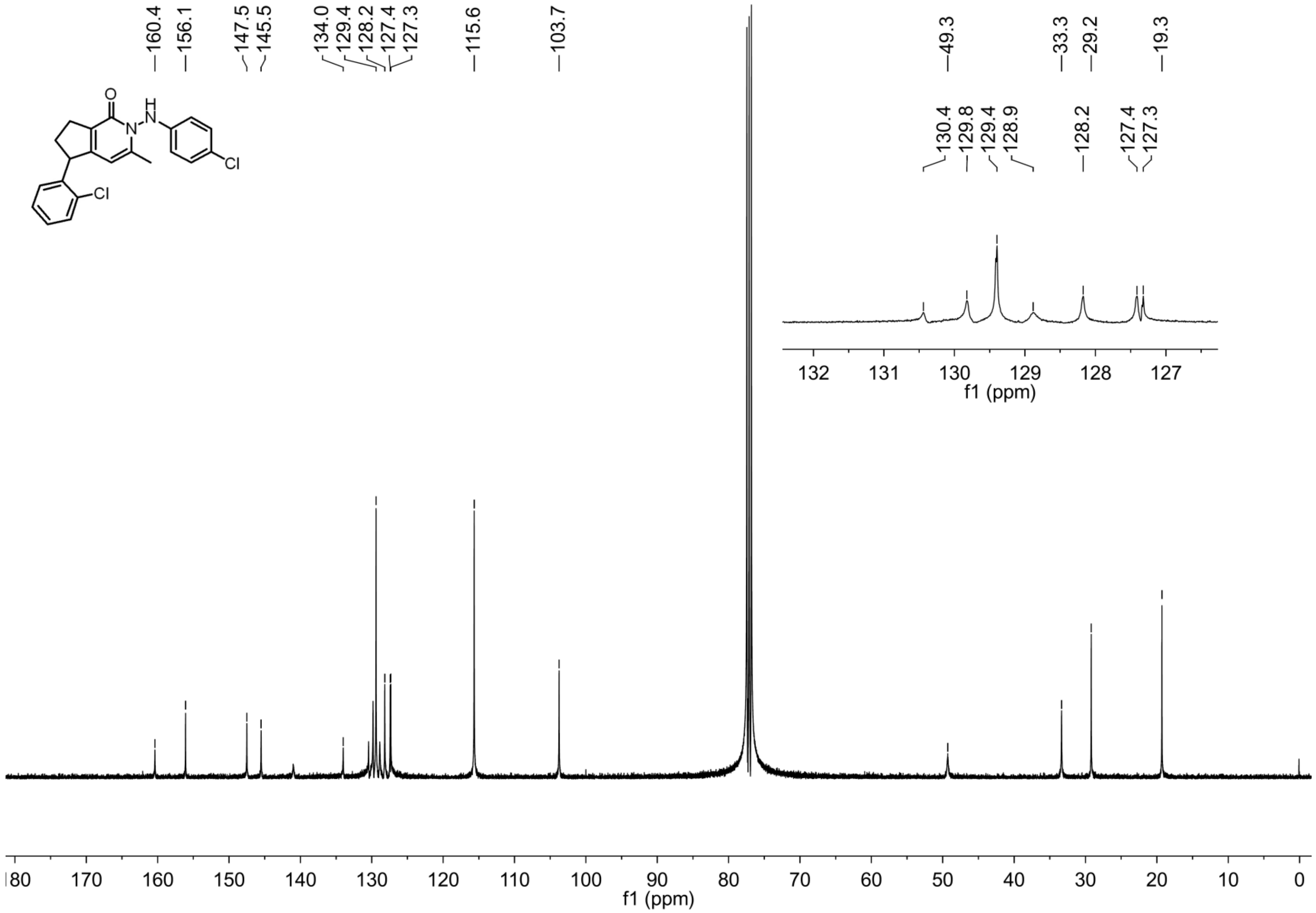

Supplementary Figure 114 ^{13}C NMR Spectrum of Compound **37**

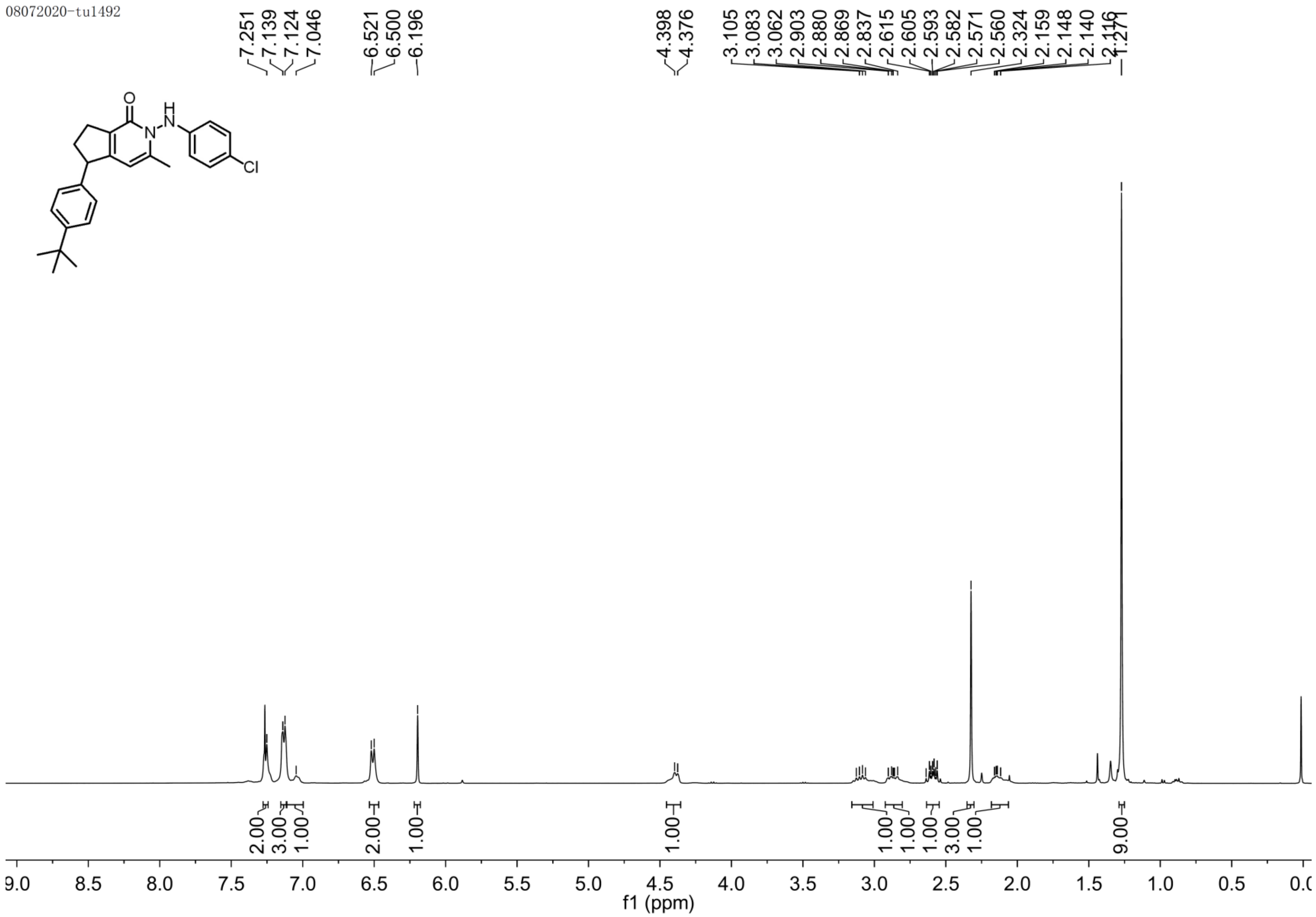

Supplementary Figure 115 ^{1}H NMR Spectrum of Compound **38**

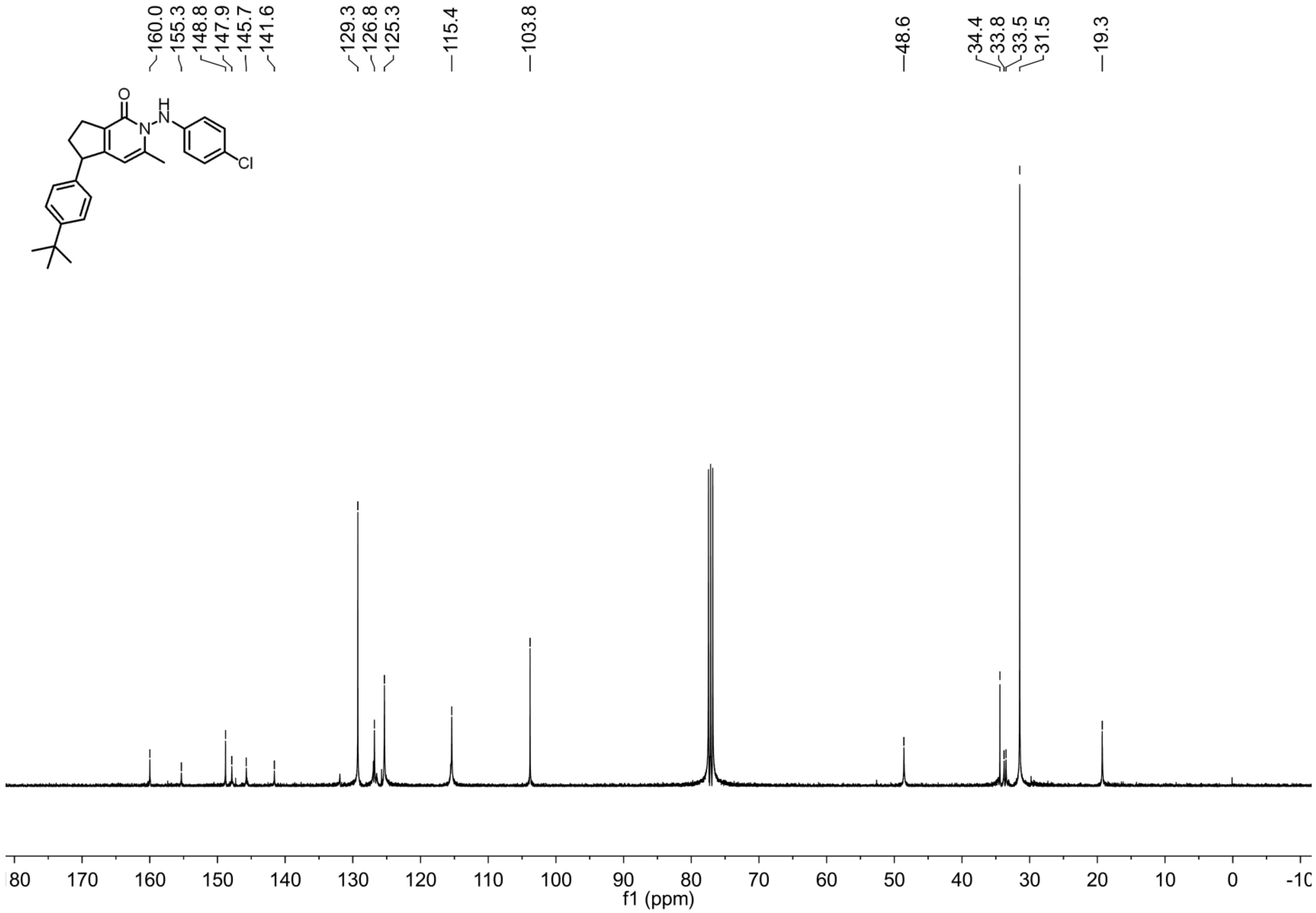

Supplementary Figure 116 ^{13}C NMR Spectrum of Compound **38**

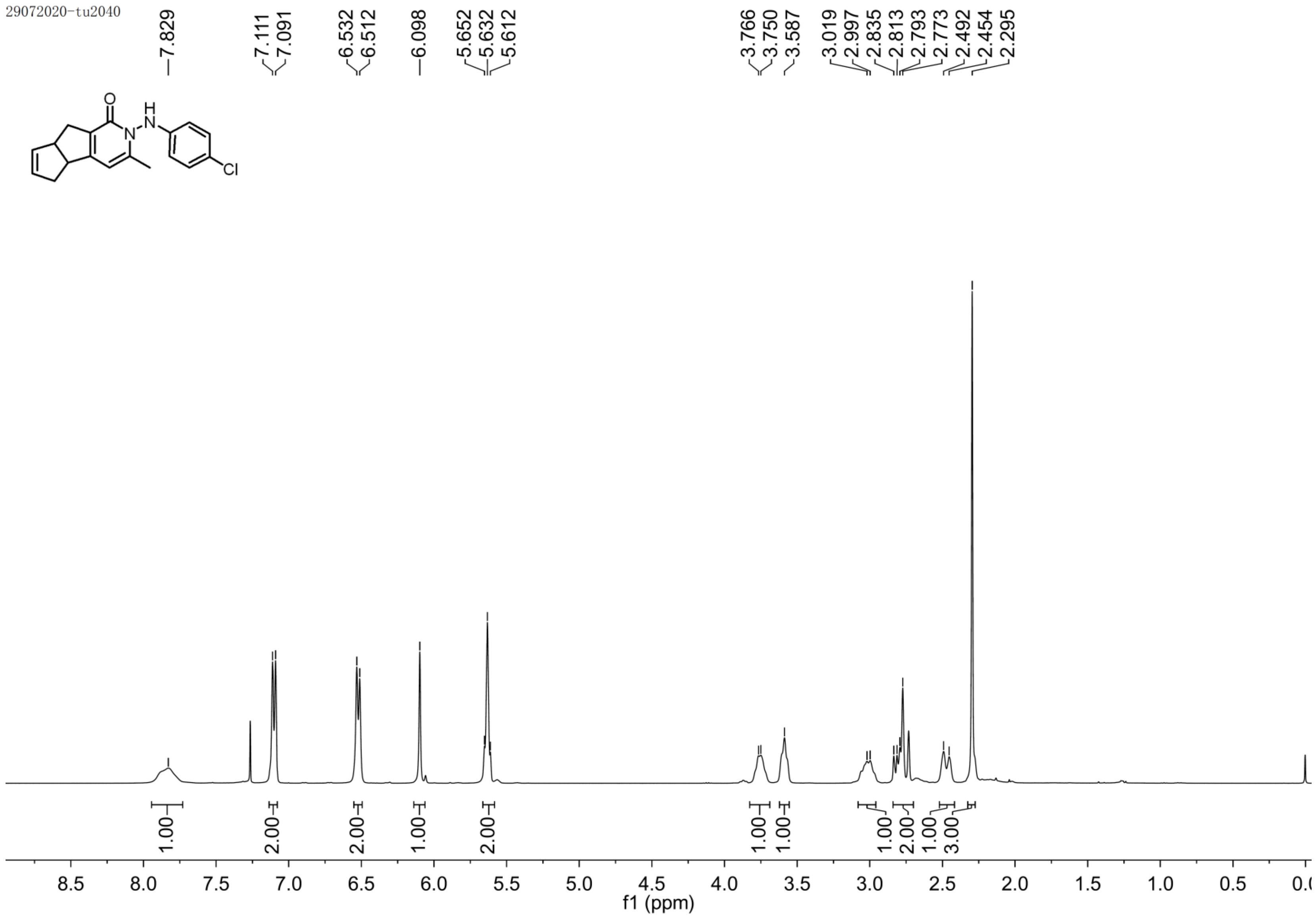

Supplementary Figure 117 ^{1}H NMR Spectrum of Compound **39**

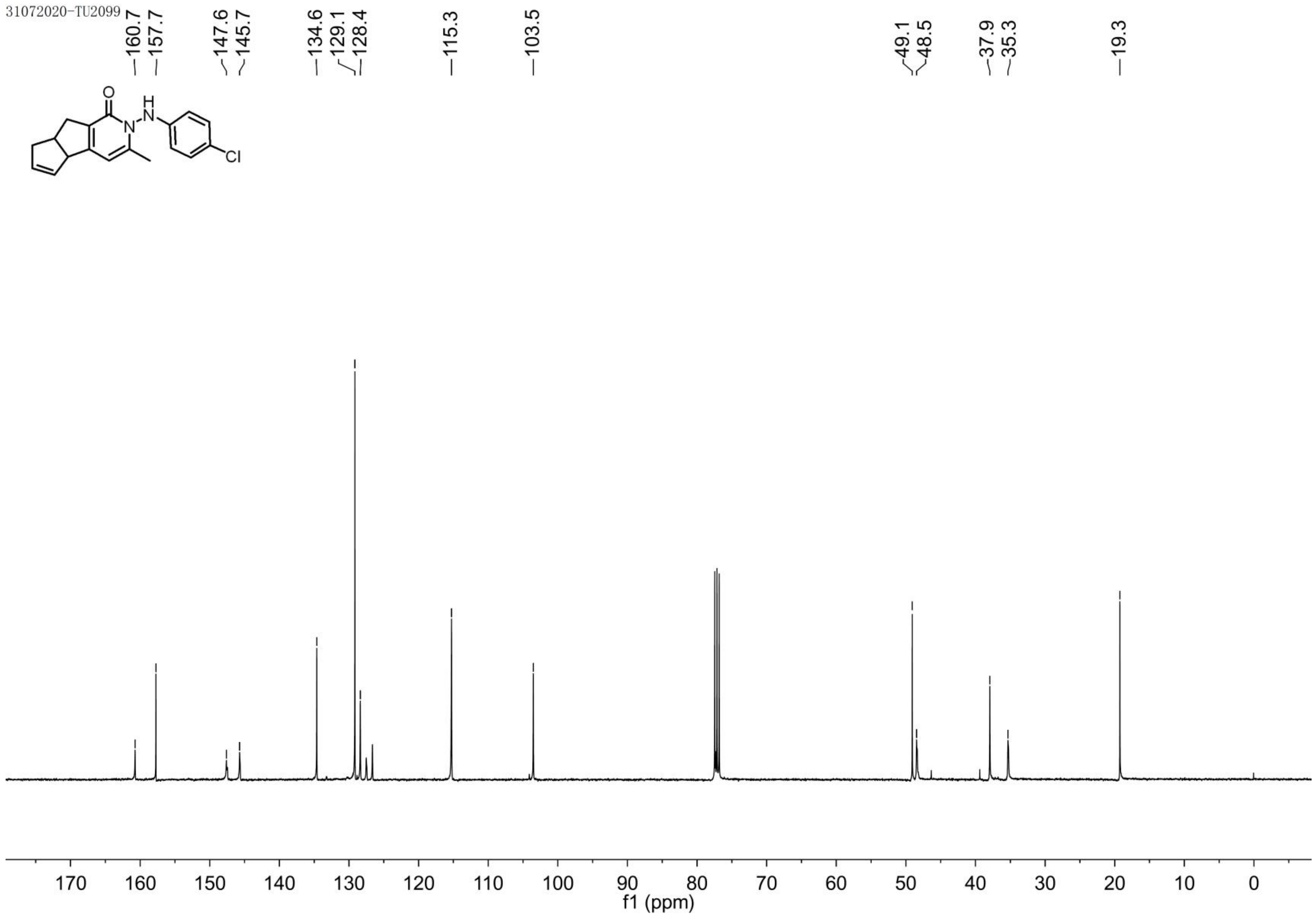

Supplementary Figure 118 ^{13}C NMR Spectrum of Compound **39**

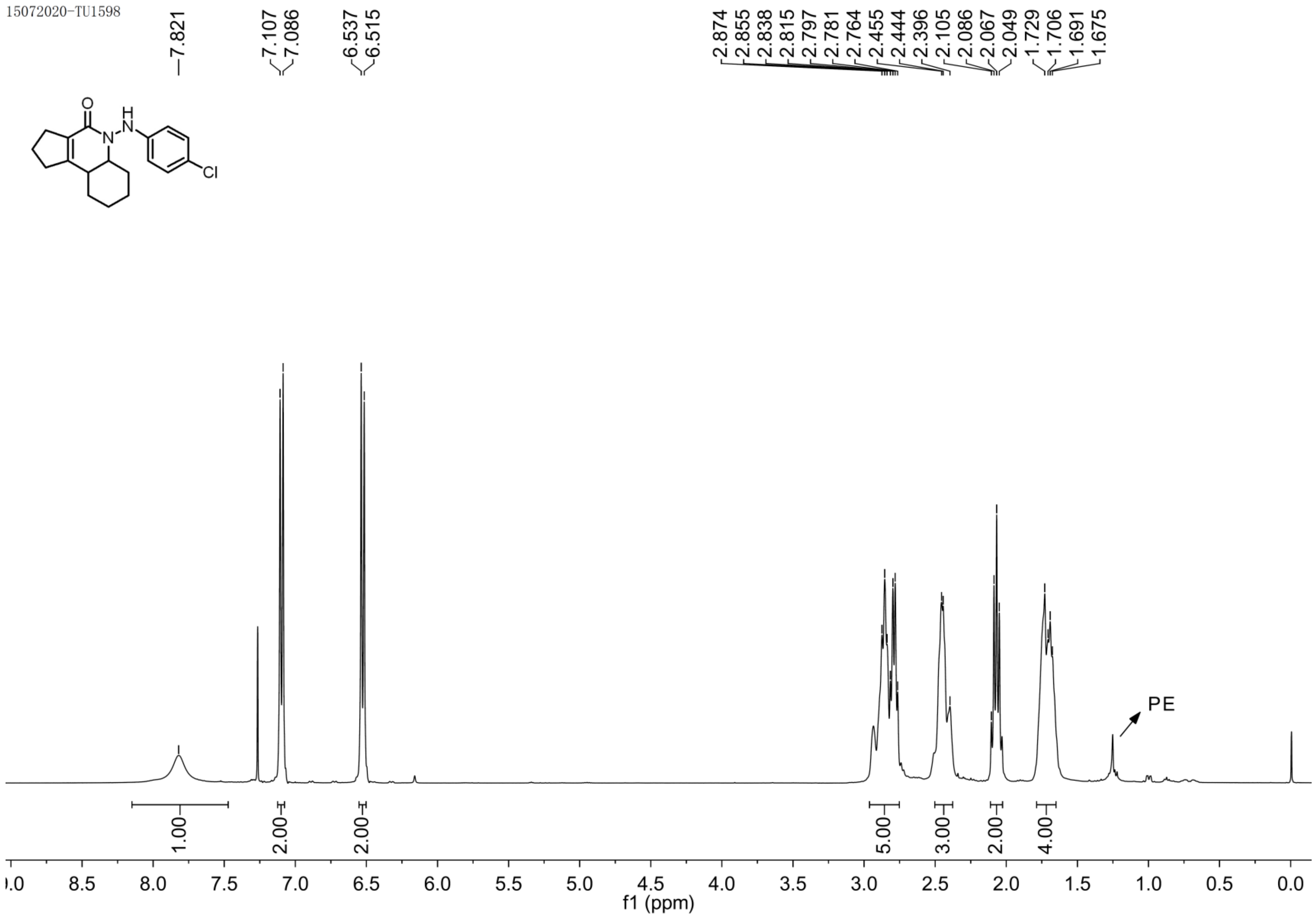

Supplementary Figure 119 ^{1}H NMR Spectrum of Compound **40**

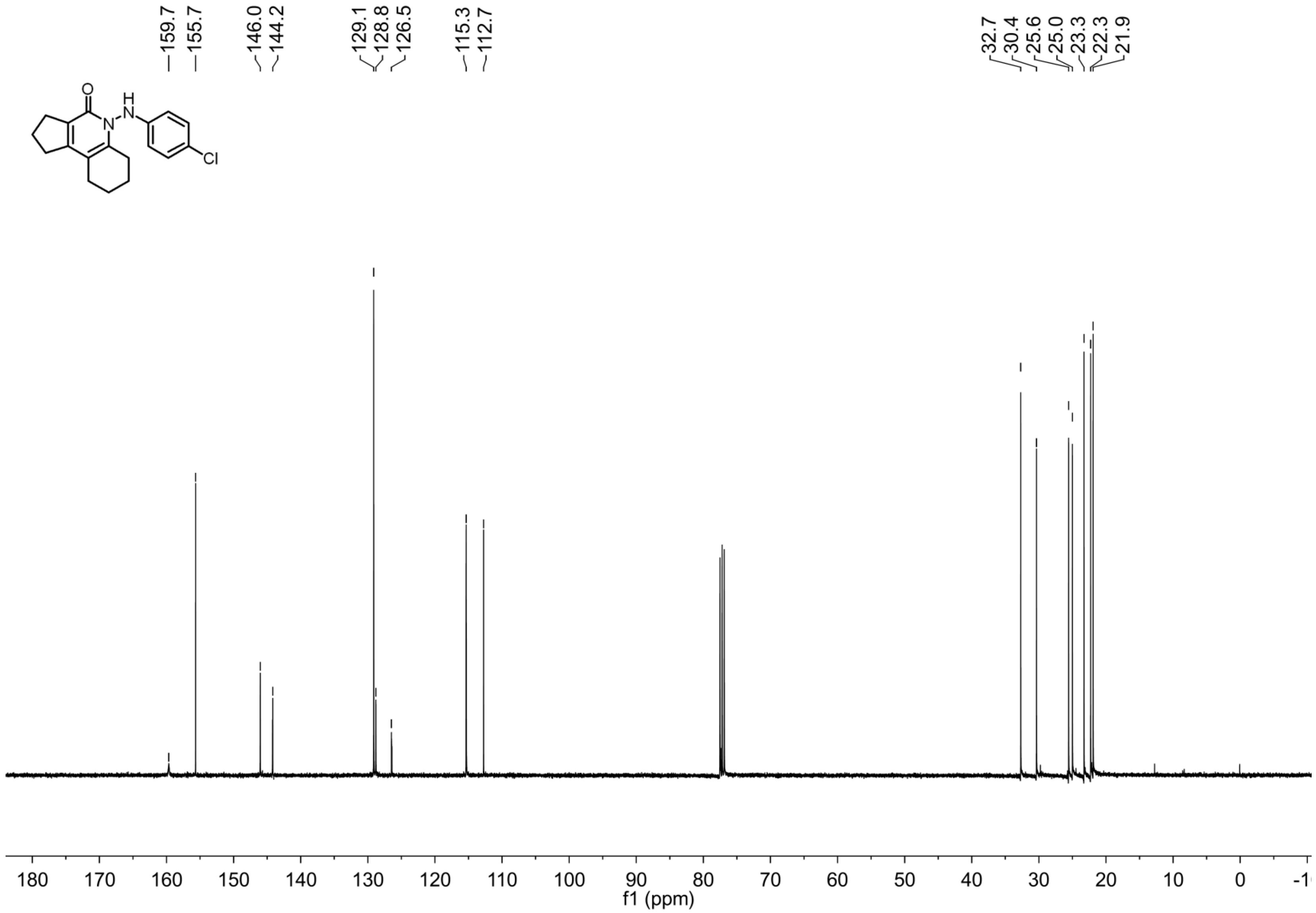

Supplementary Figure 120 ^{13}C NMR Spectrum of Compound **40**

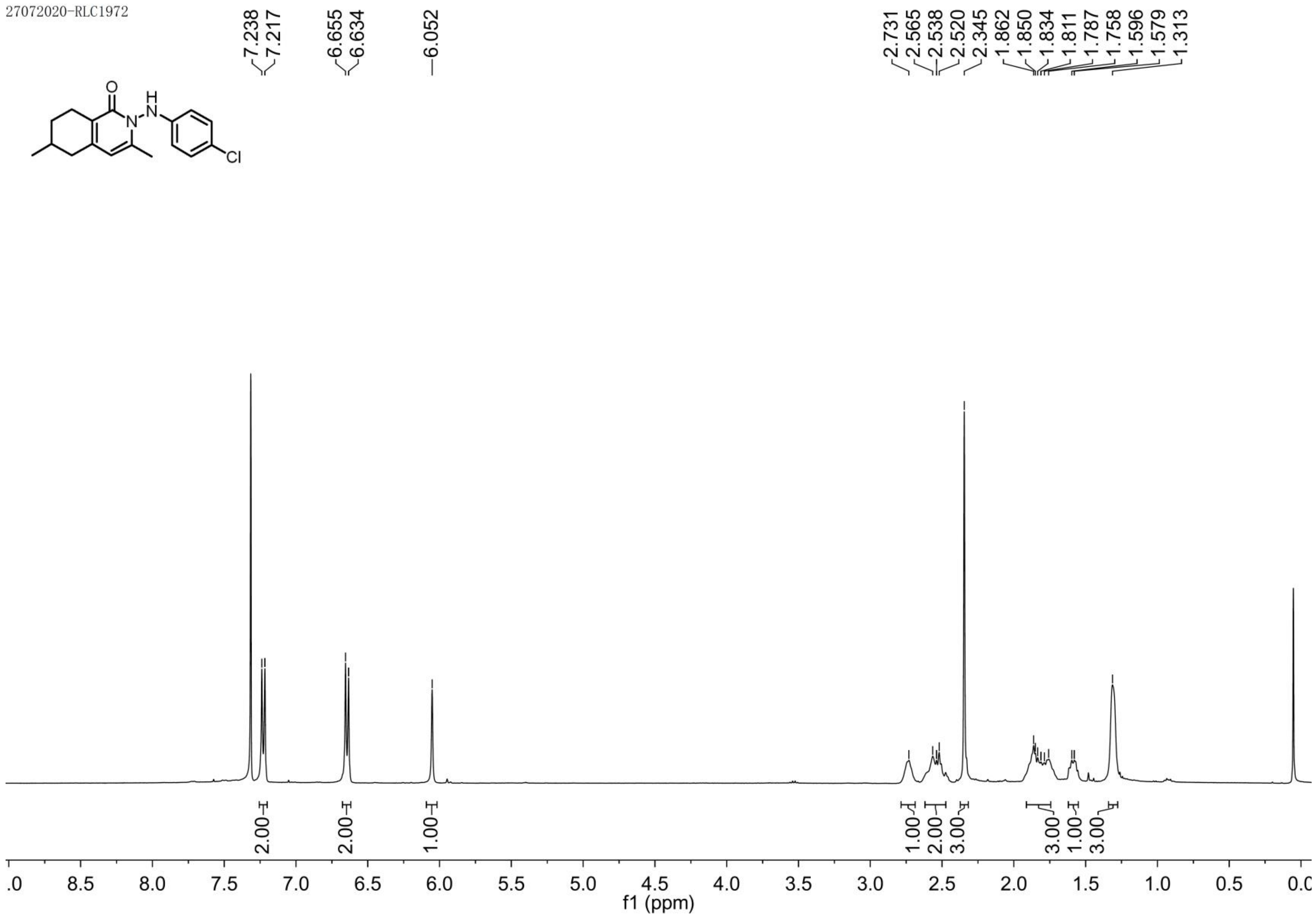

Supplementary Figure 121 ^{1}H NMR Spectrum of Compound **41**

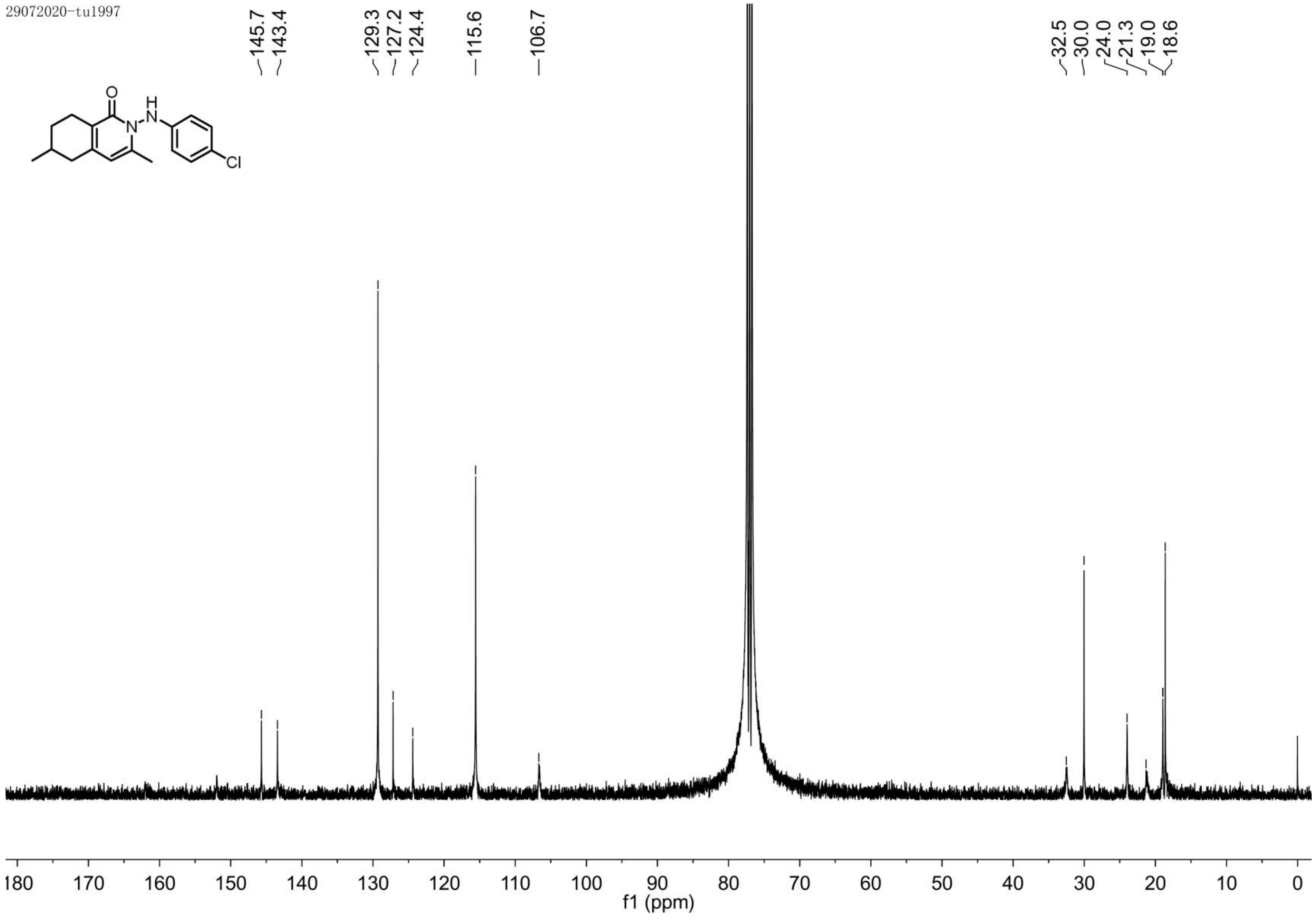

Supplementary Figure 122 ^{13}C NMR Spectrum of Compound **41**

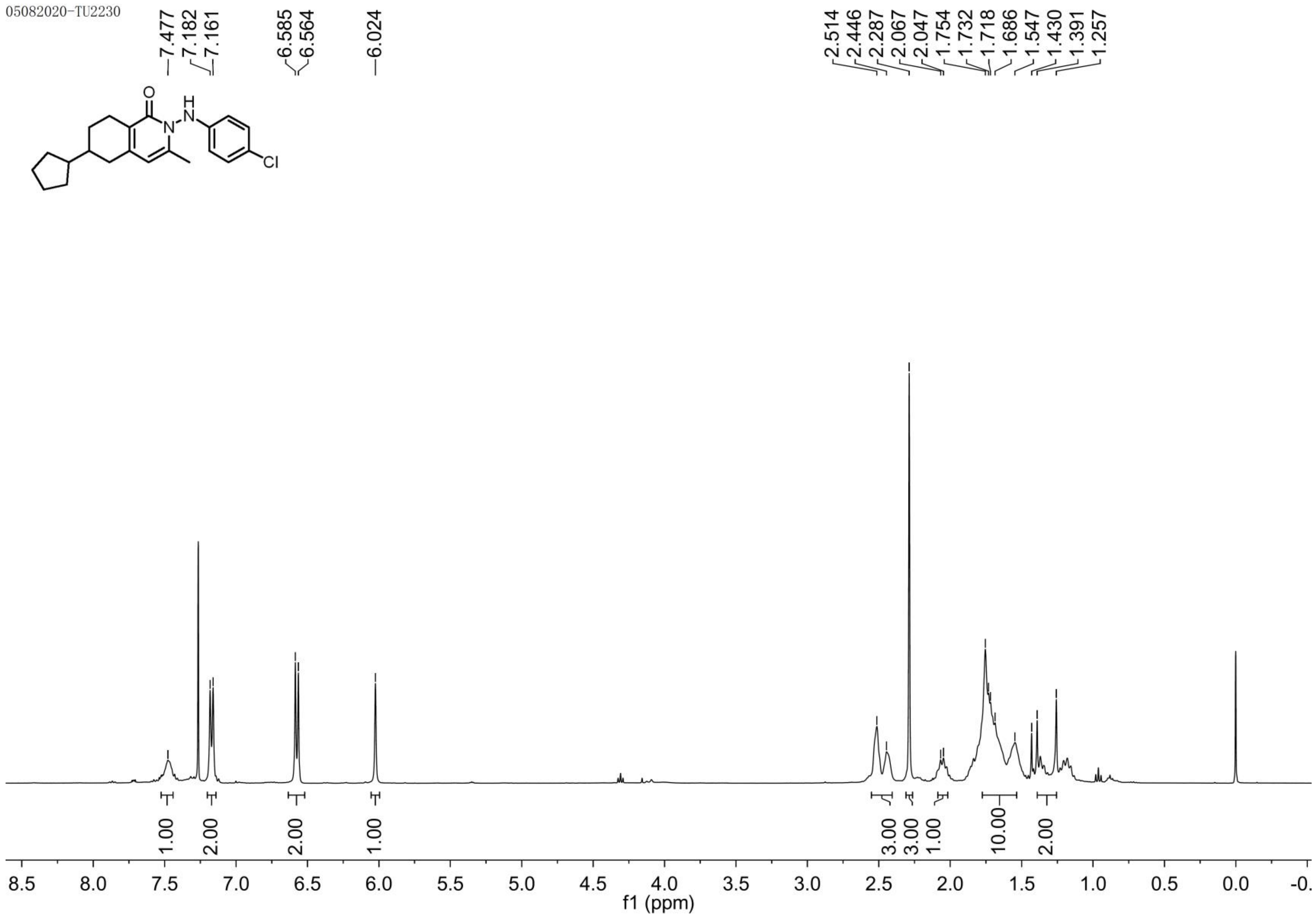

Supplementary Figure 123 ^{1}H NMR Spectrum of Compound **42**

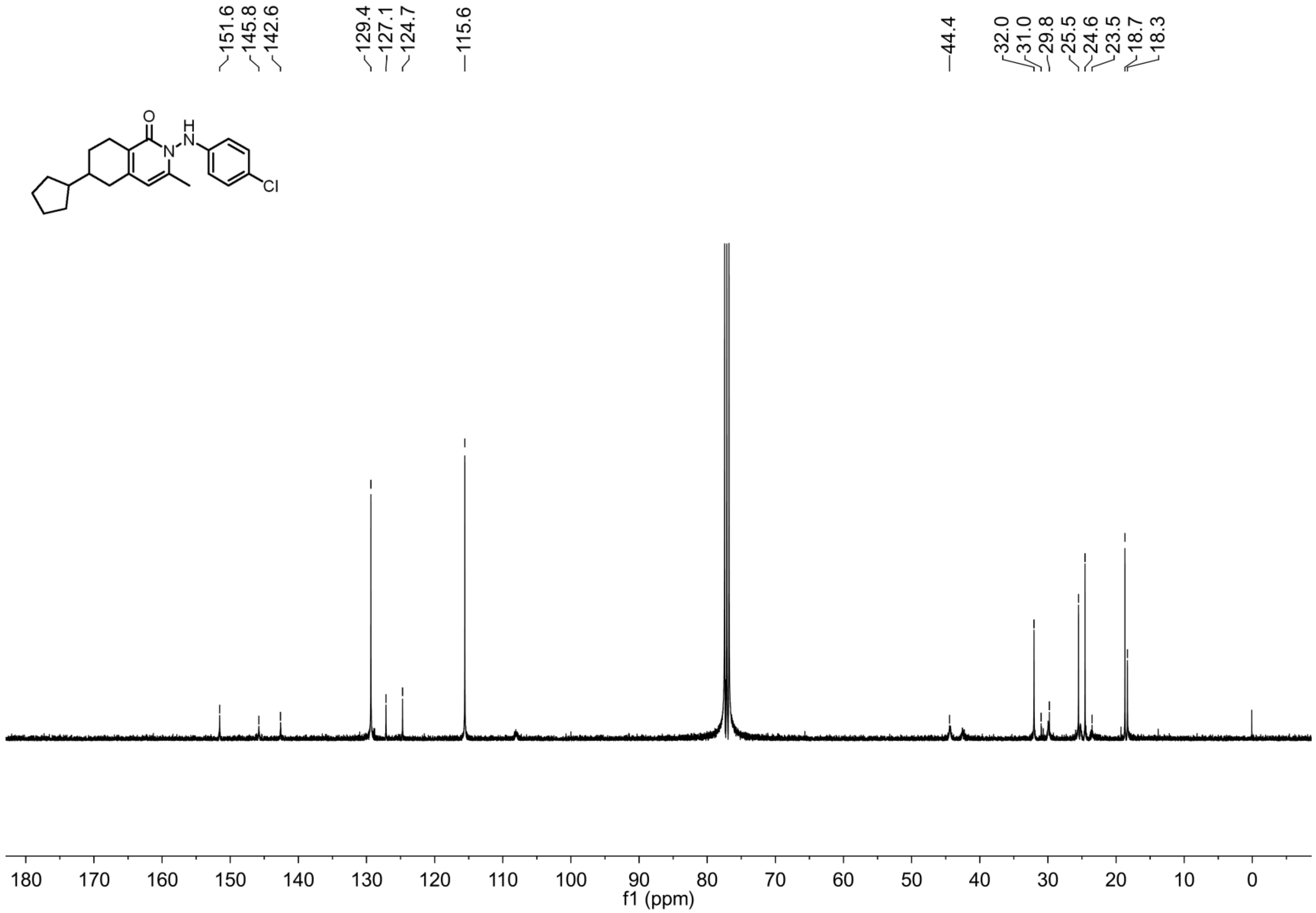

Supplementary Figure 124 ^{13}C NMR Spectrum of Compound **42**

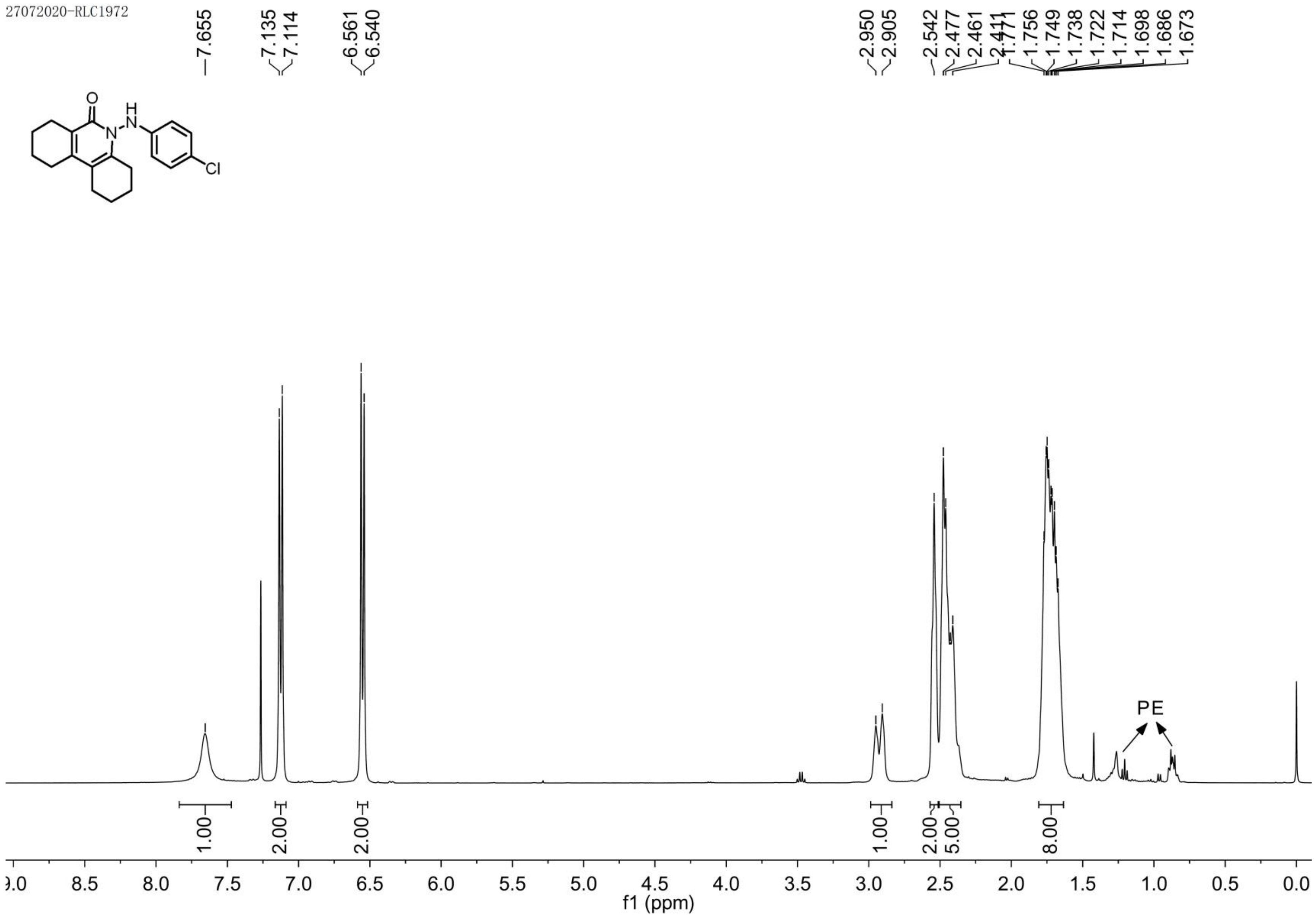

Supplementary Figure 125 ^{1}H NMR Spectrum of Compound **43**

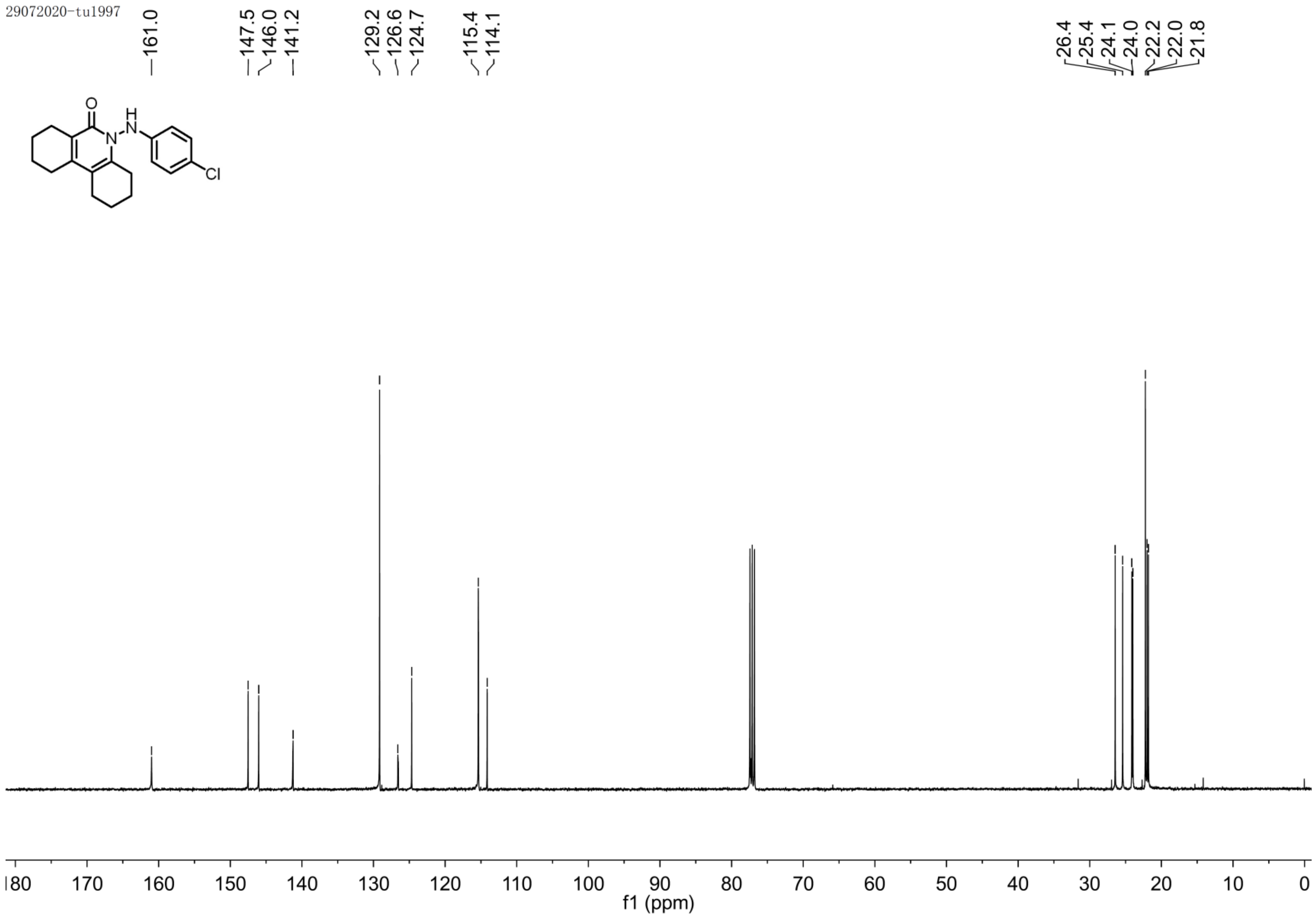

Supplementary Figure 126 ^{13}C NMR Spectrum of Compound **43**

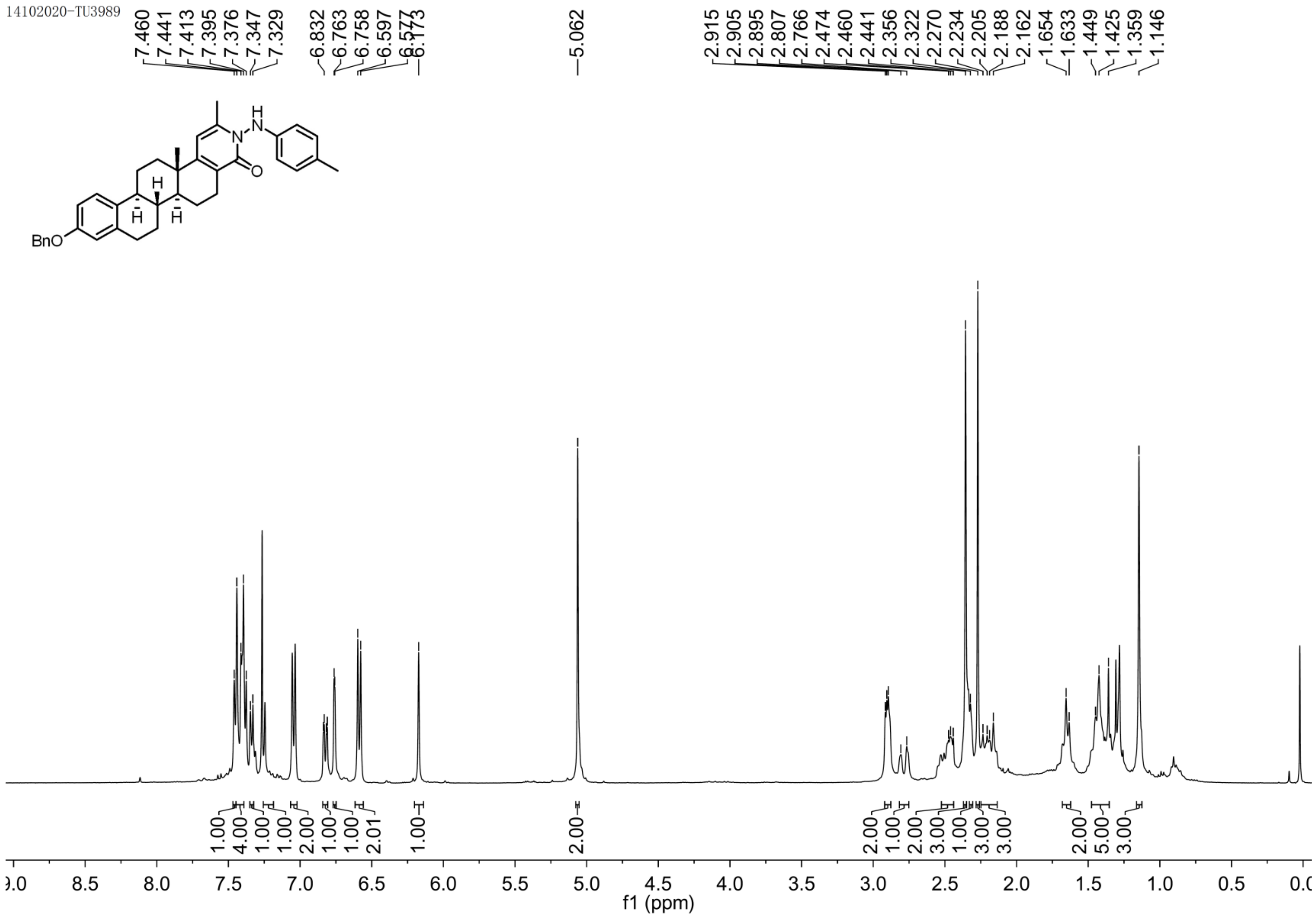

Supplementary Figure 127 ^{1}H NMR Spectrum of Compound **44**

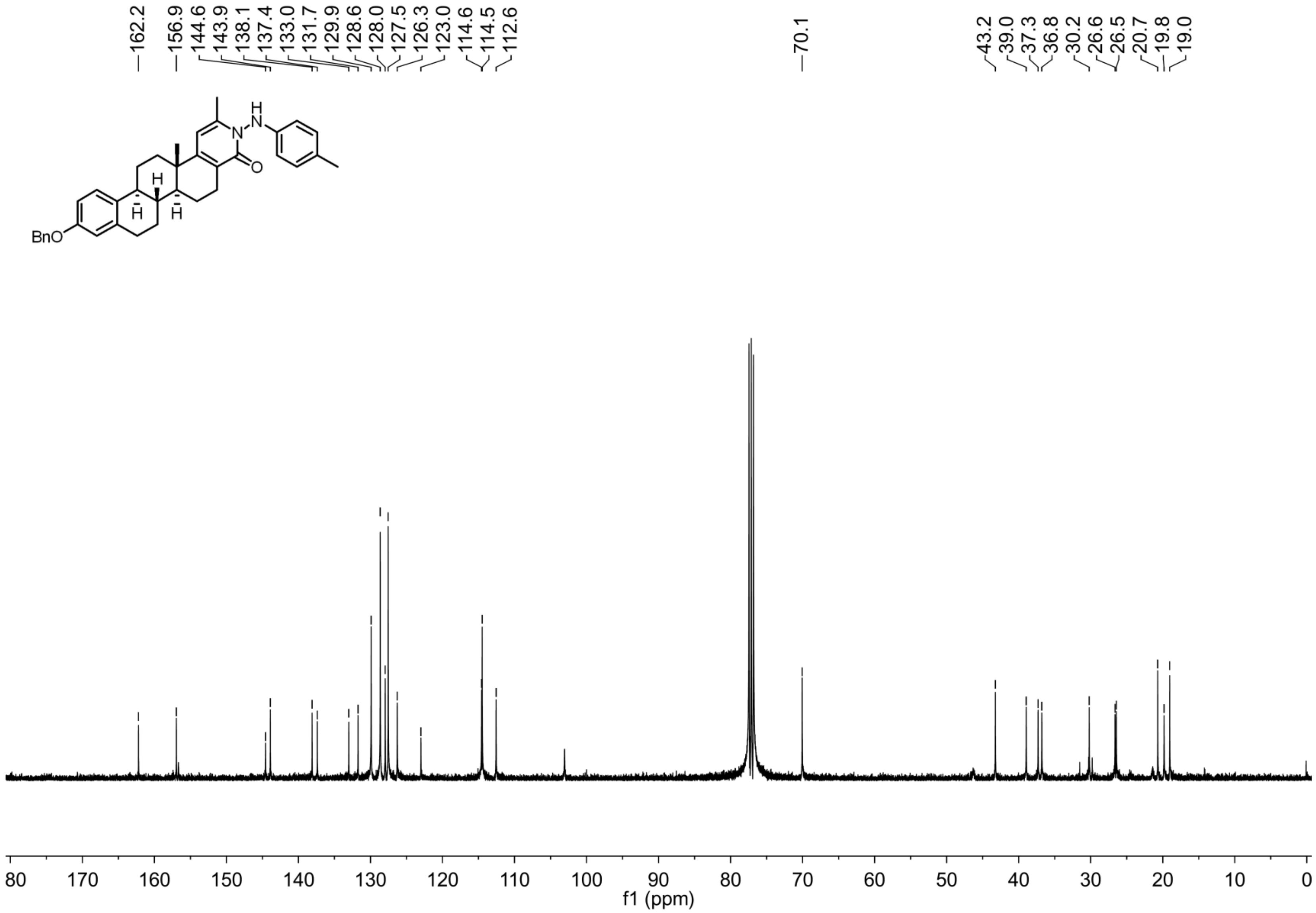

Supplementary Figure 128 ^{13}C NMR Spectrum of Compound **44**

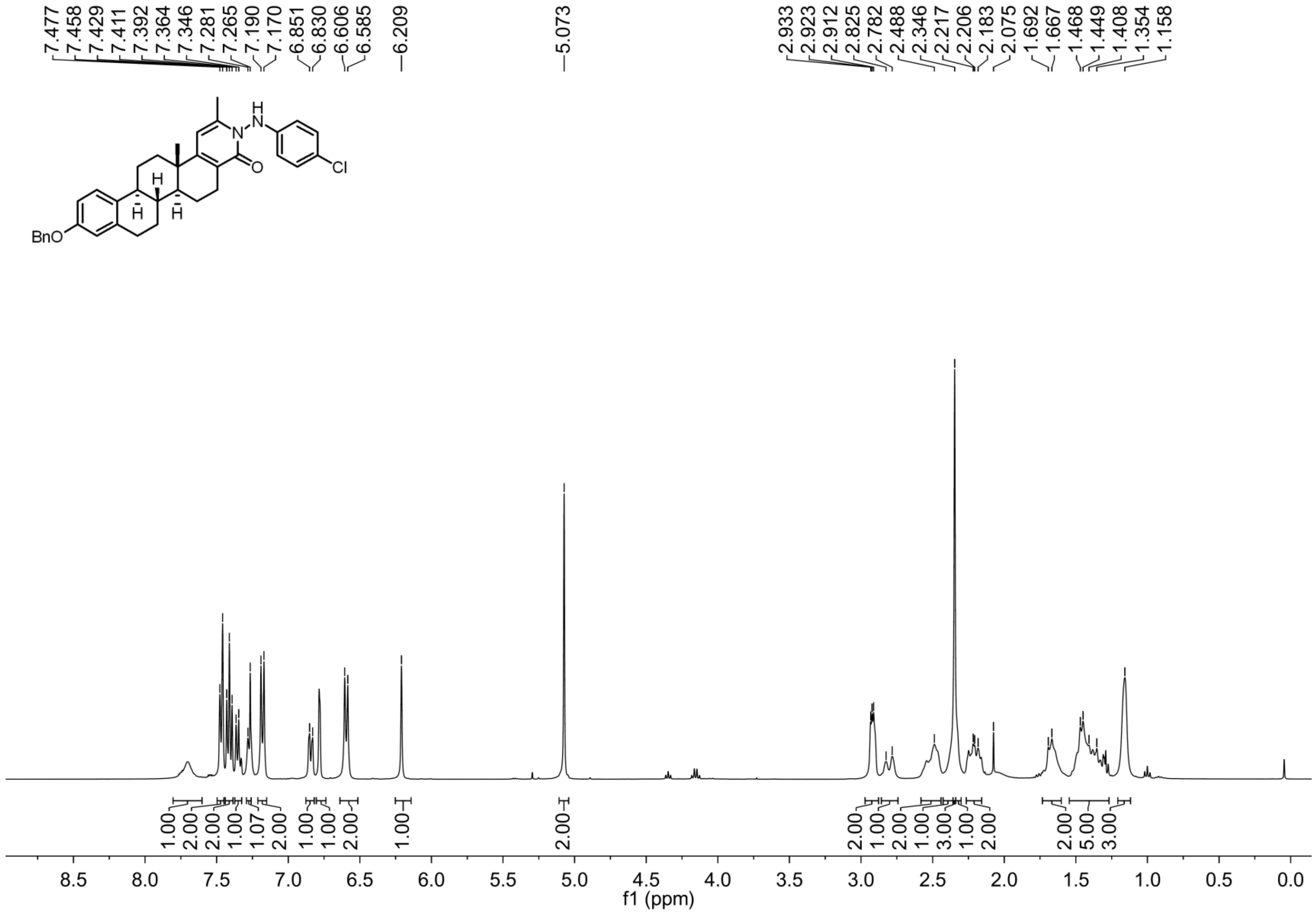

Supplementary Figure 129 ^{1}H NMR Spectrum of Compound **45**

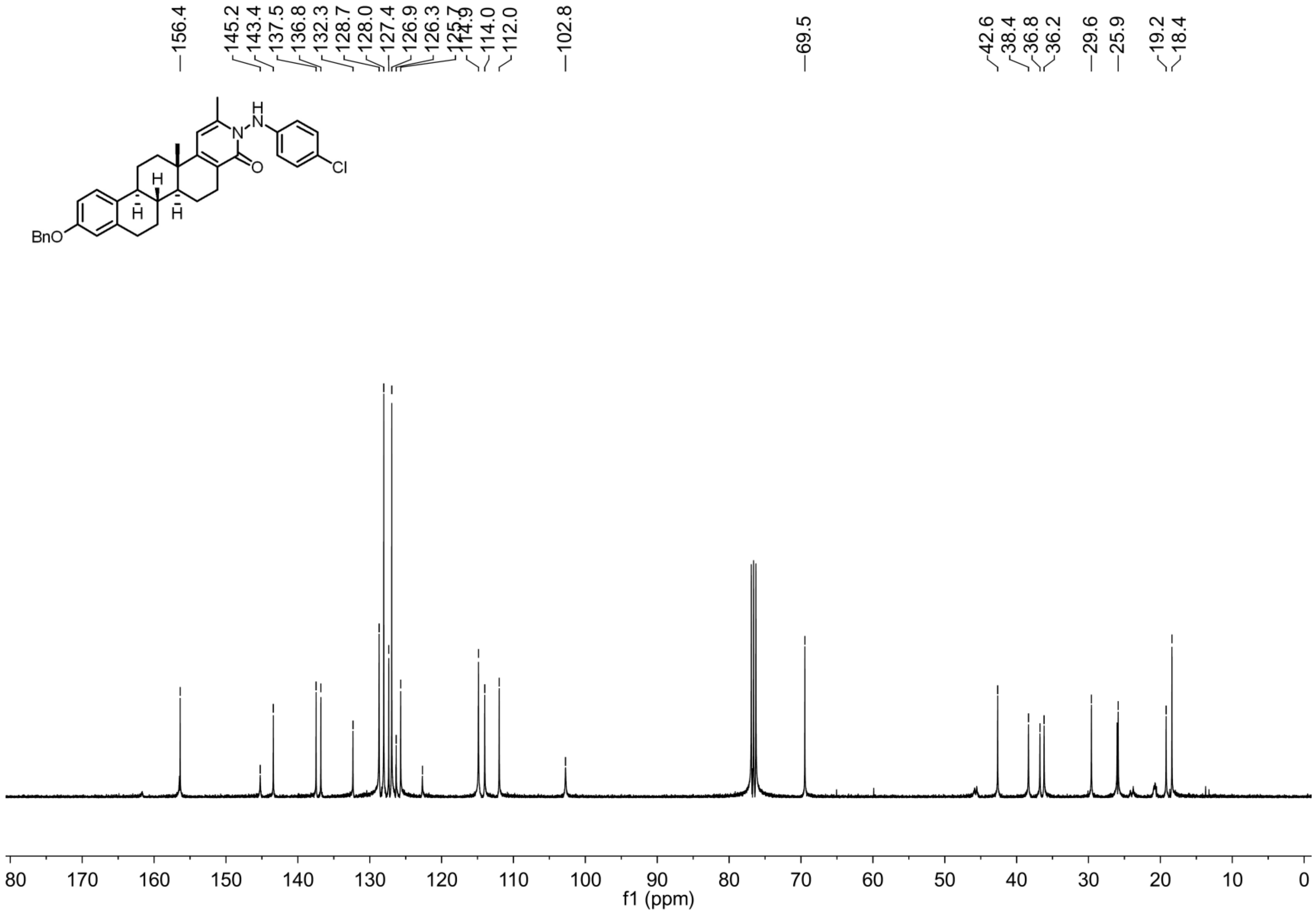

Supplementary Figure 130 ^{13}C NMR Spectrum of Compound **45**

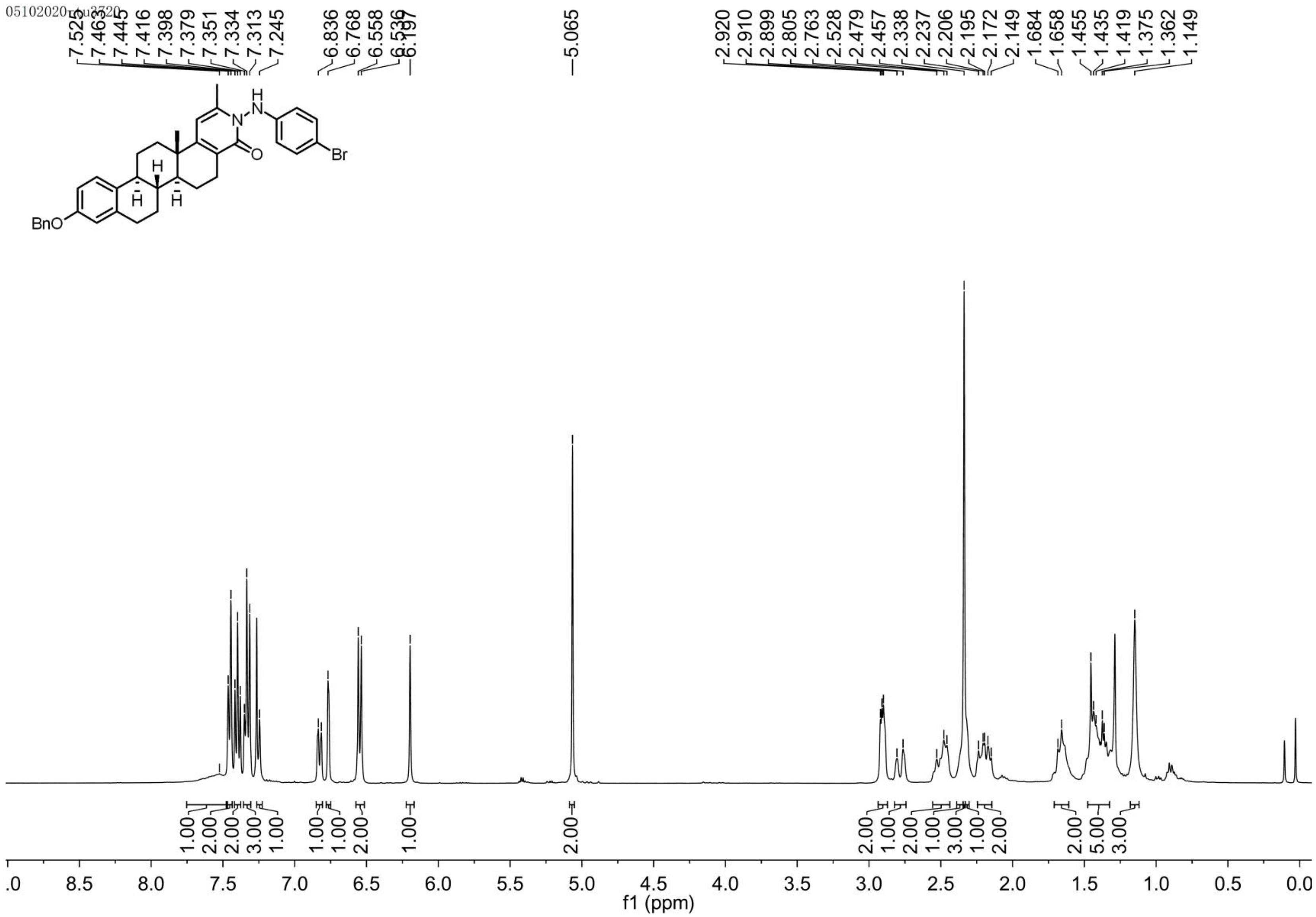

Supplementary Figure 131 ^{1}H NMR Spectrum of Compound **46**

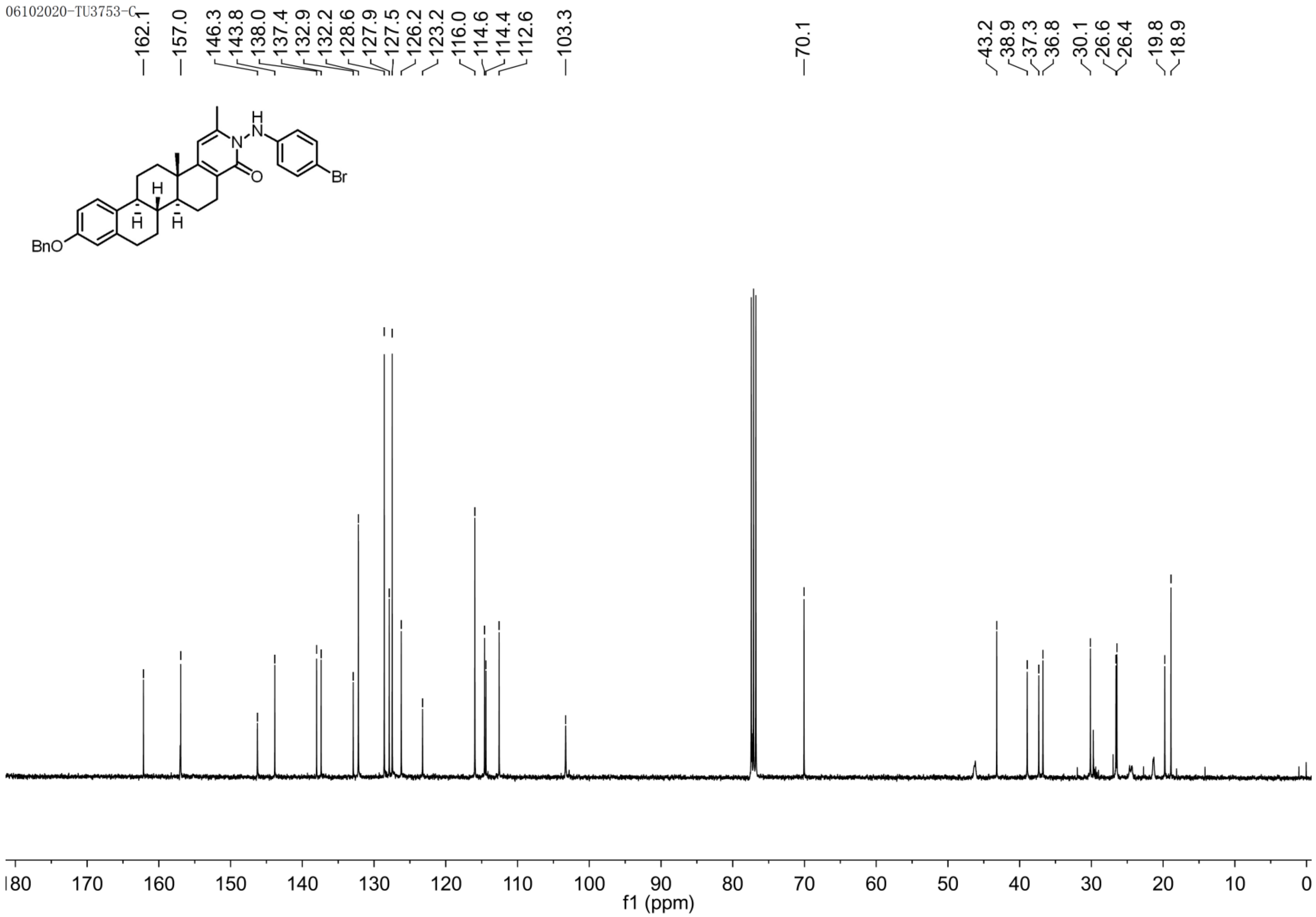

Supplementary Figure 132 ^{13}C NMR Spectrum of Compound **46**

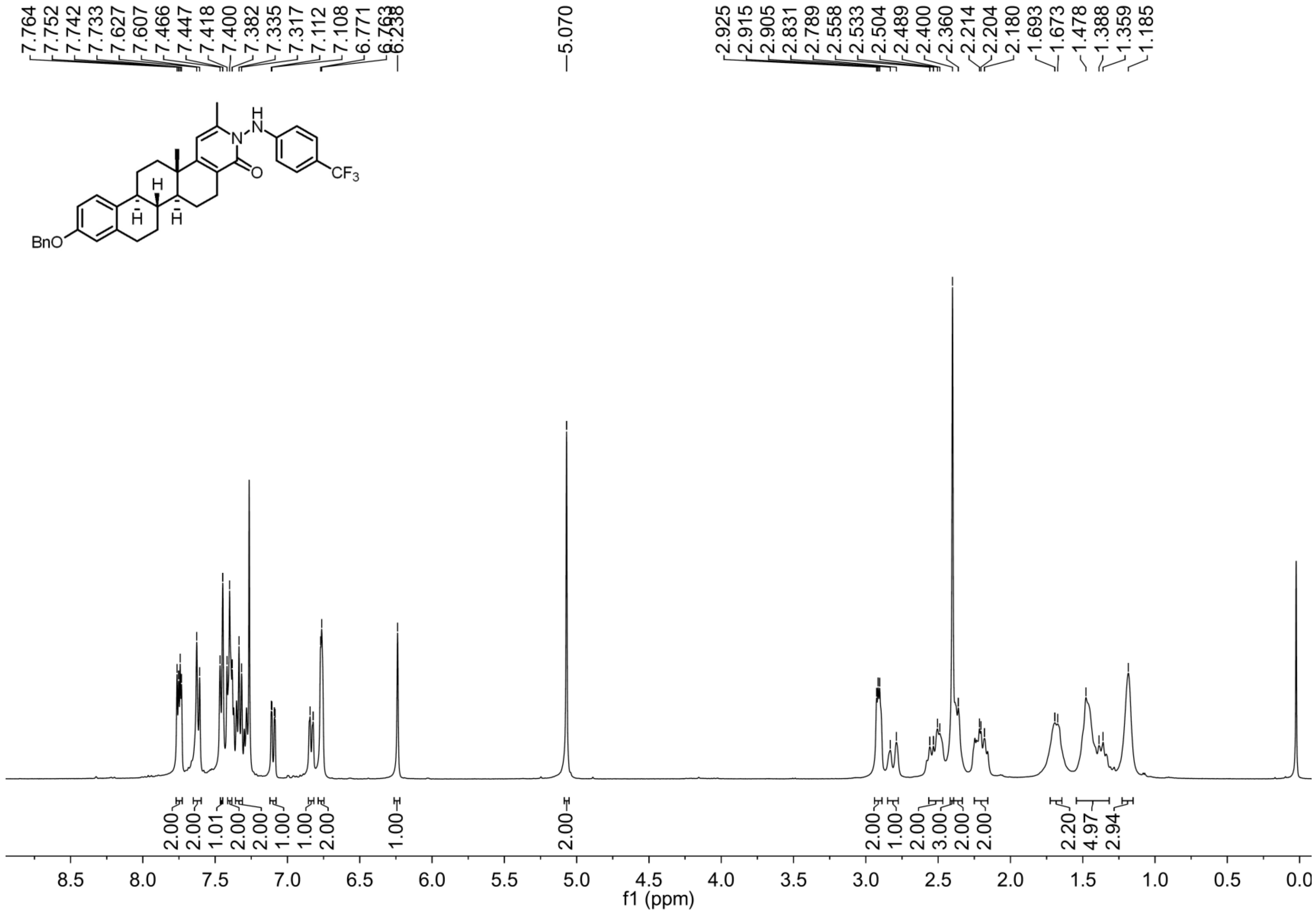

Supplementary Figure 133 ^{1}H NMR Spectrum of Compound **47**

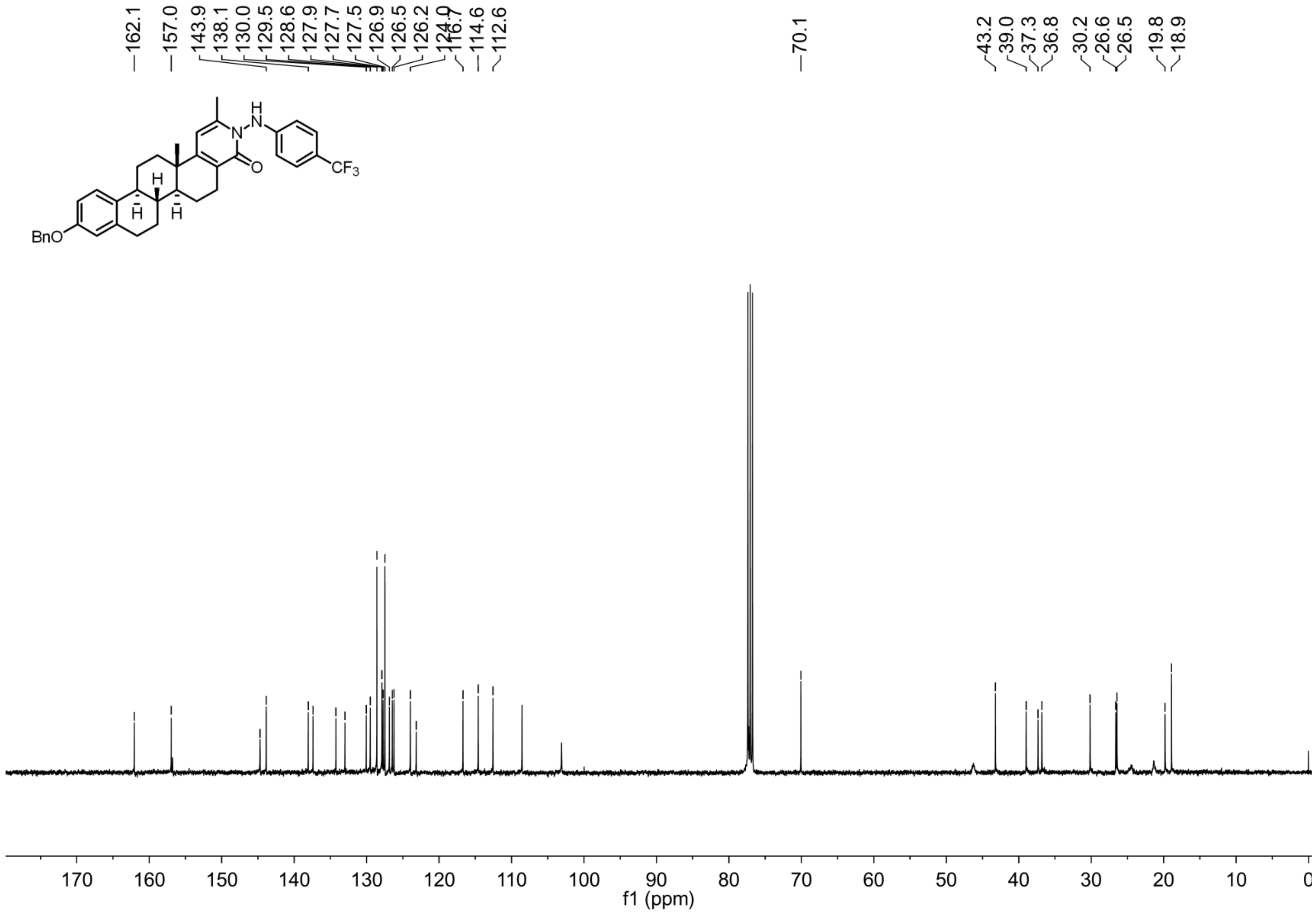

Supplementary Figure 134 ^{13}C NMR Spectrum of Compound **47**

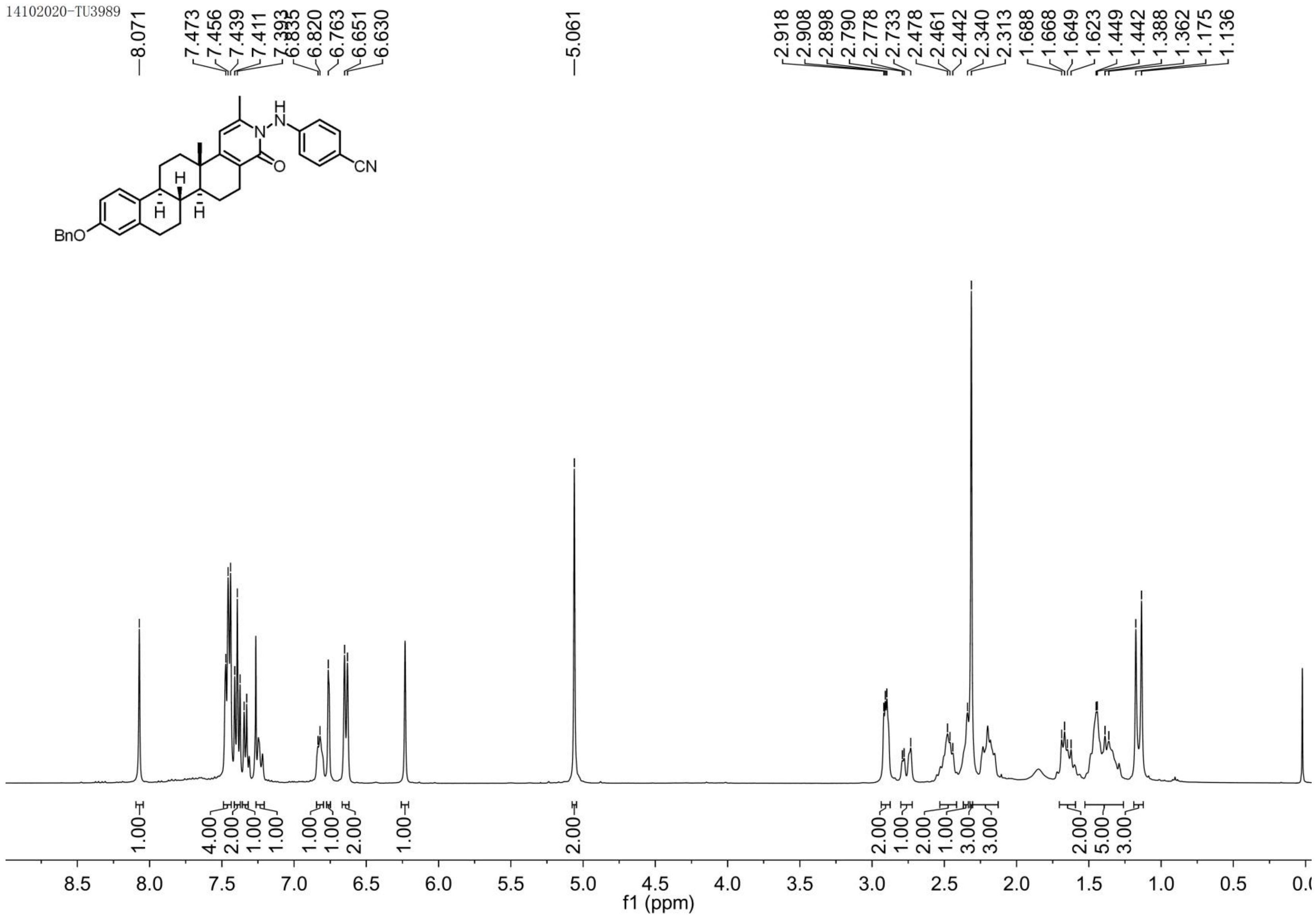

Supplementary Figure 135 ^{1}H NMR Spectrum of Compound **48**

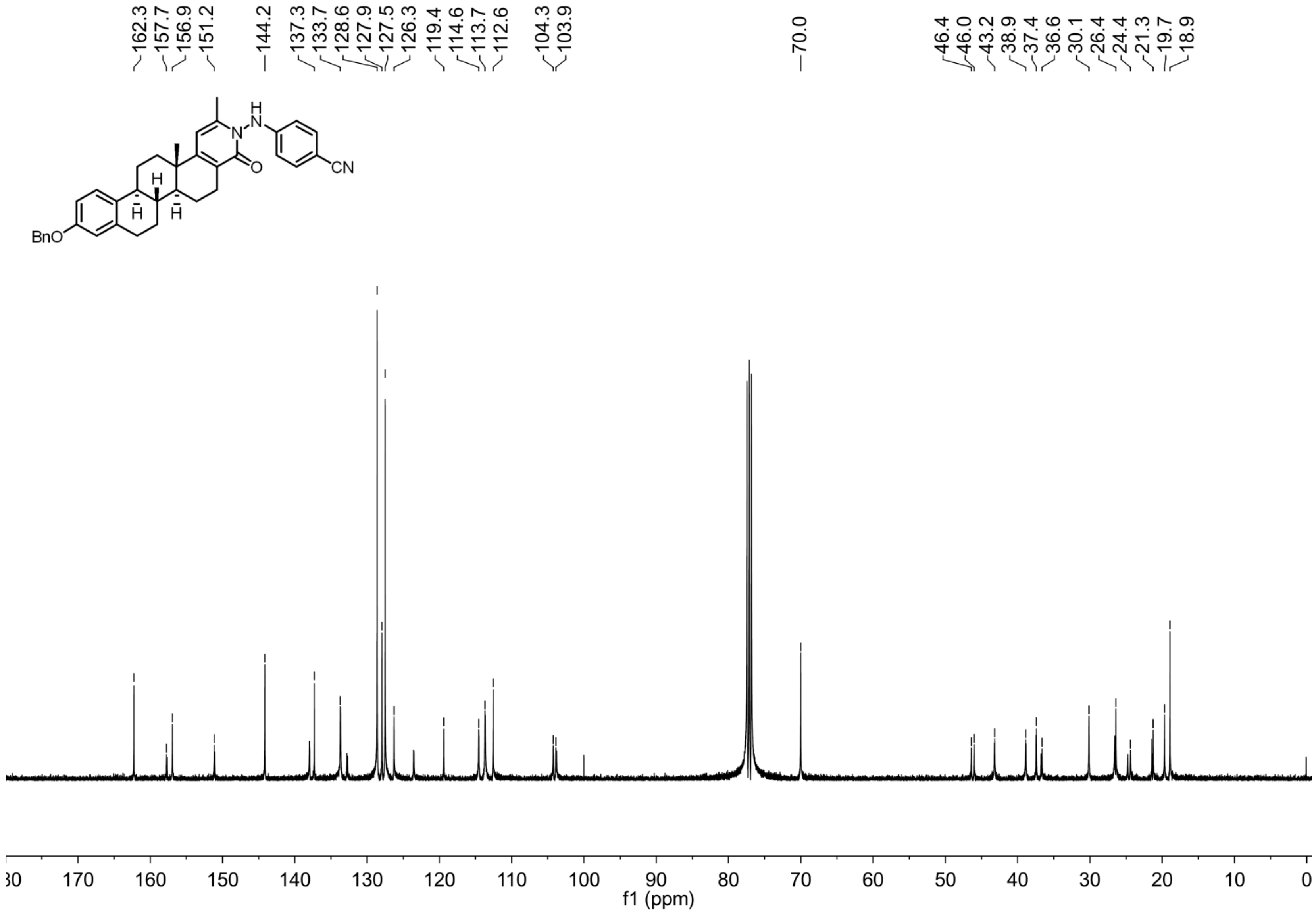

Supplementary Figure 136 ^{13}C NMR Spectrum of Compound **48**

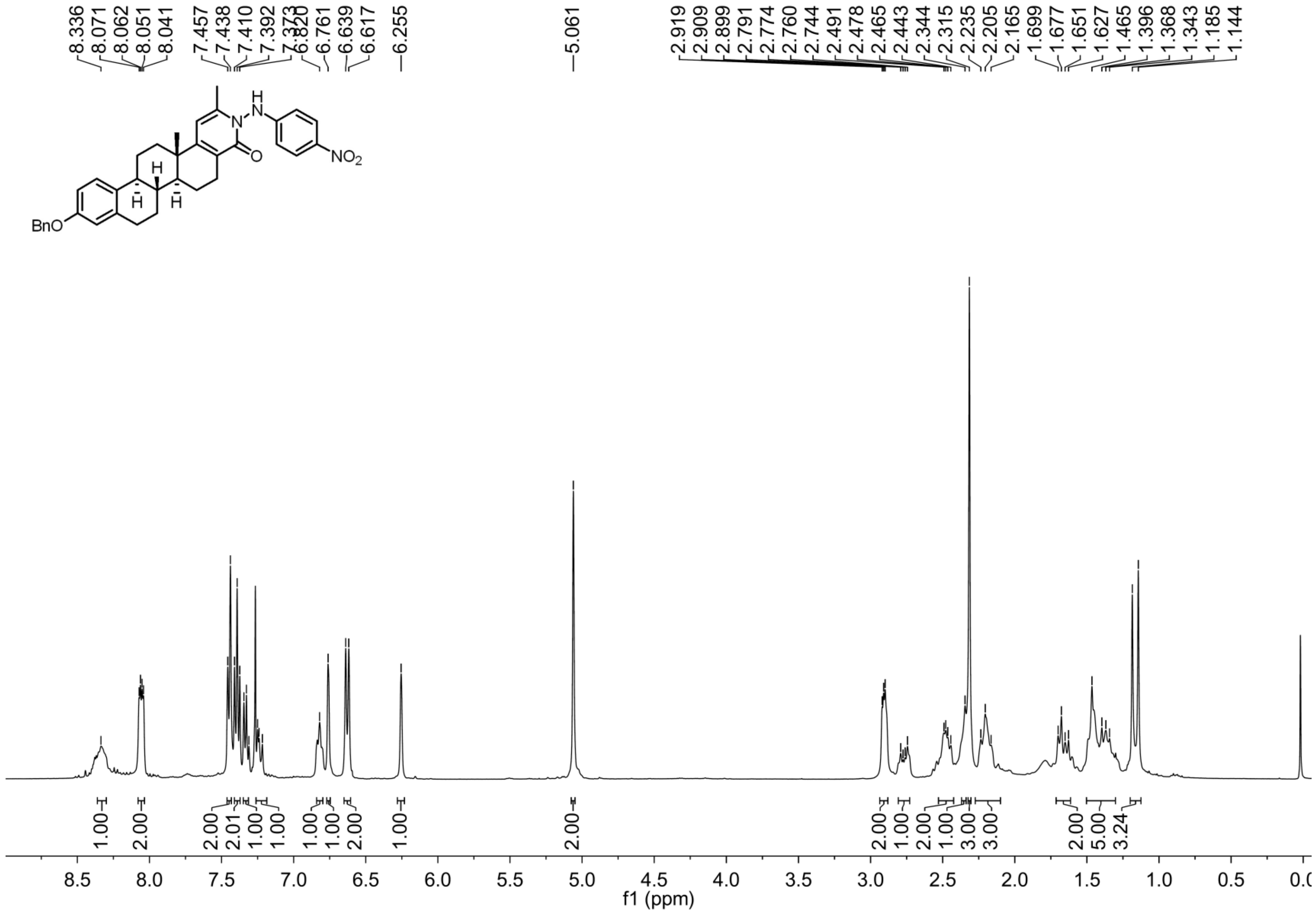

Supplementary Figure 137 ^{1}H NMR Spectrum of Compound **49**

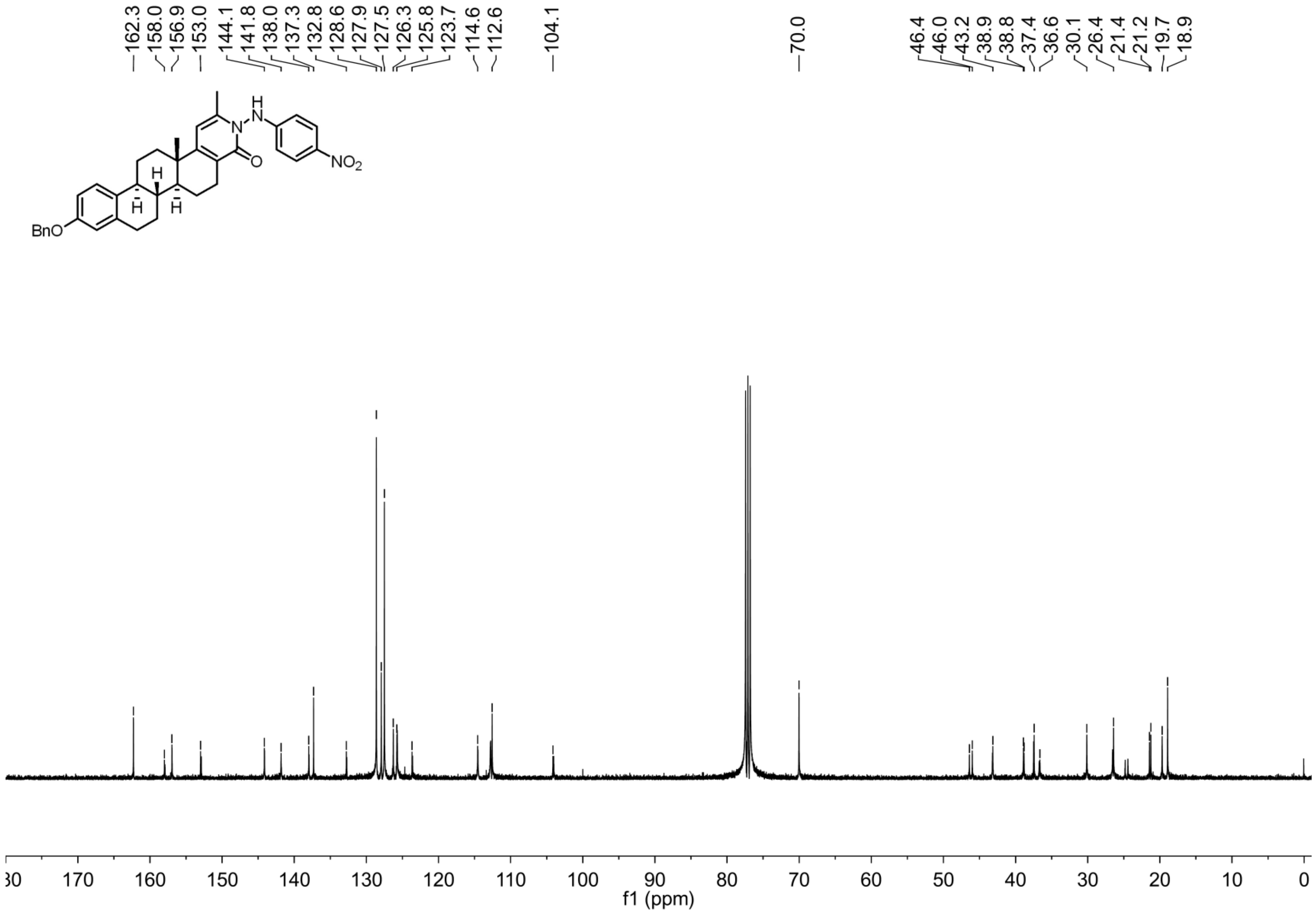

Supplementary Figure 138 ^{13}C NMR Spectrum of Compound **49**

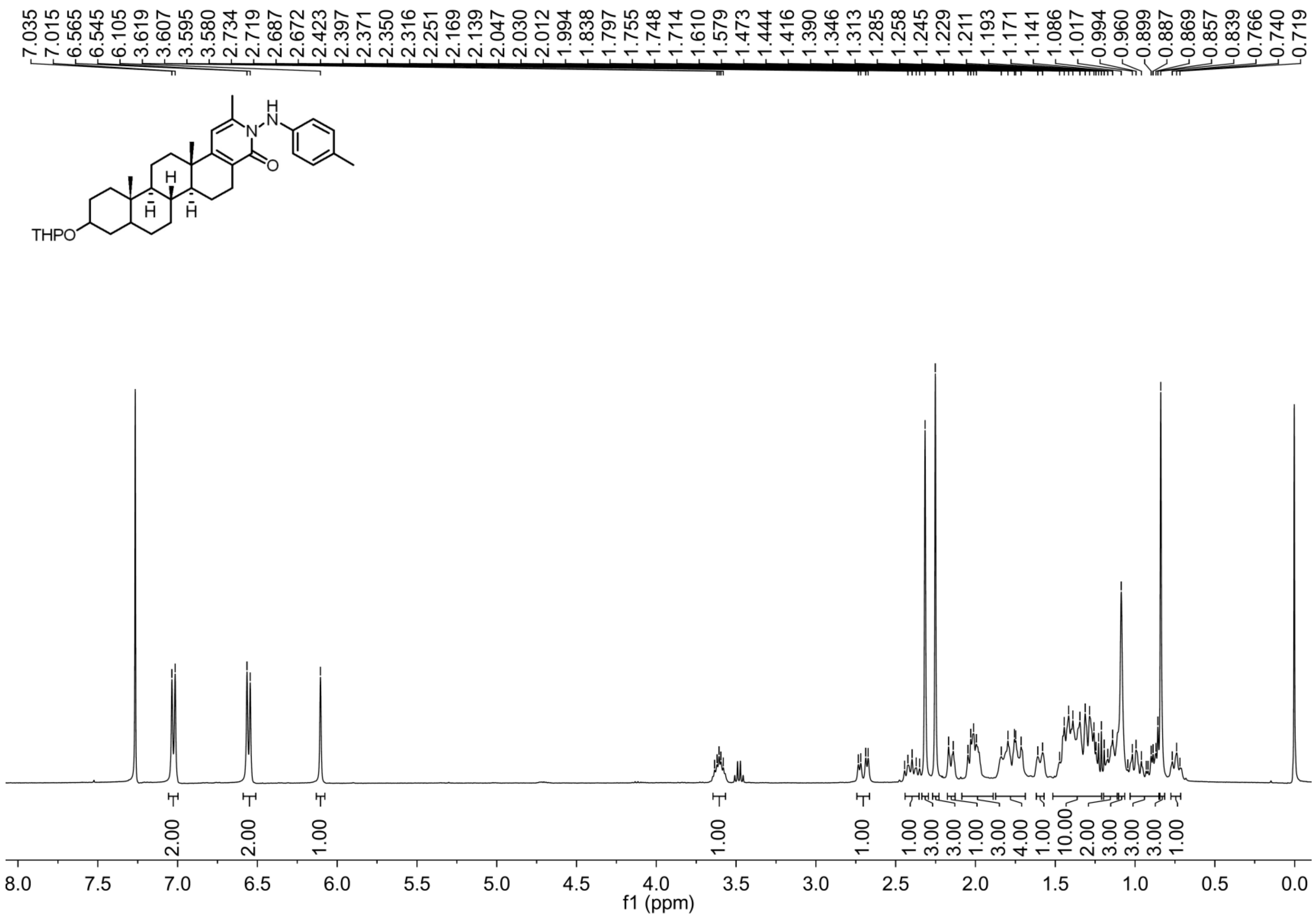

Supplementary Figure 139 ^{1}H NMR Spectrum of Compound **50**

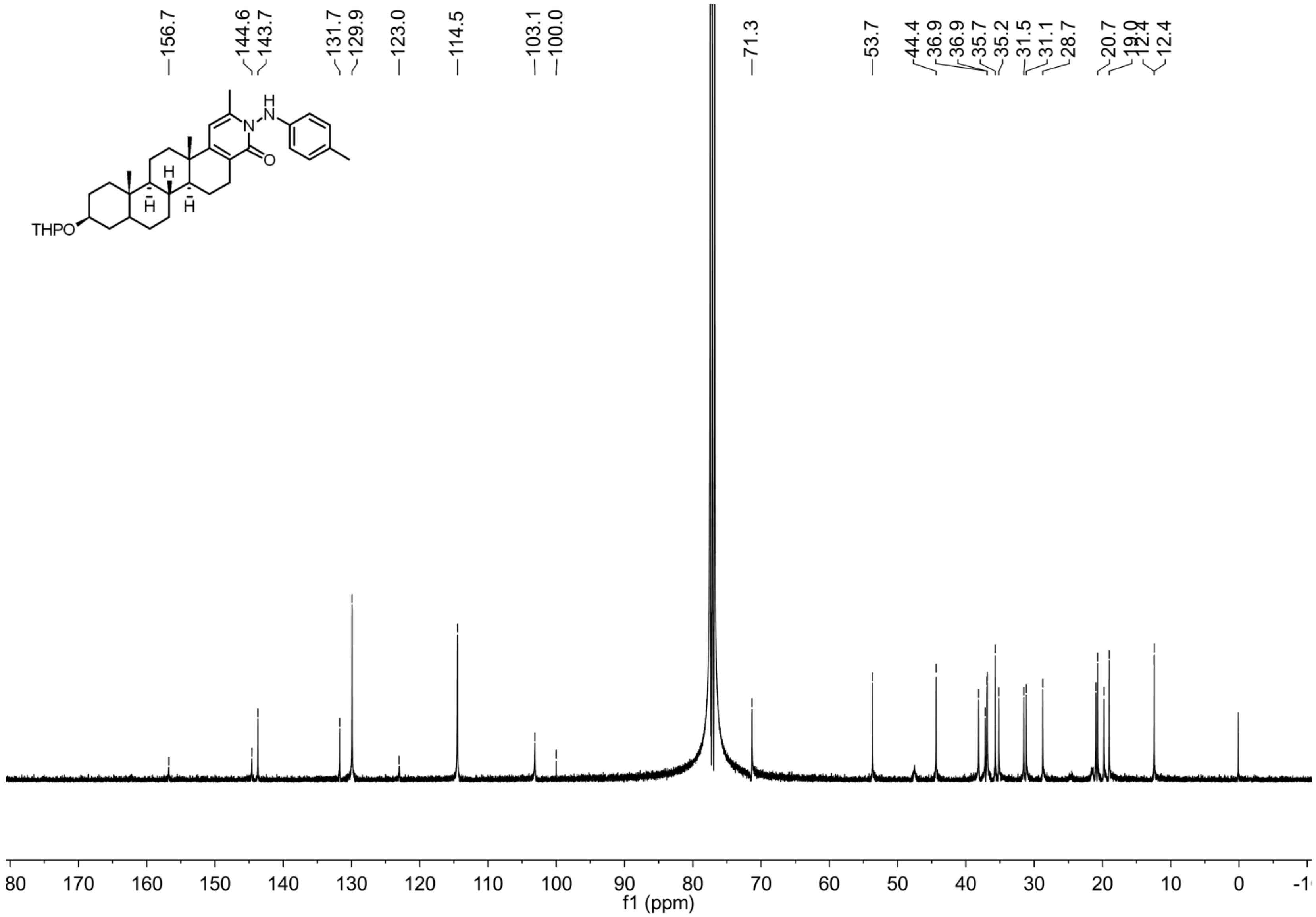

Supplementary Figure 140 ^{13}C NMR Spectrum of Compound **50**

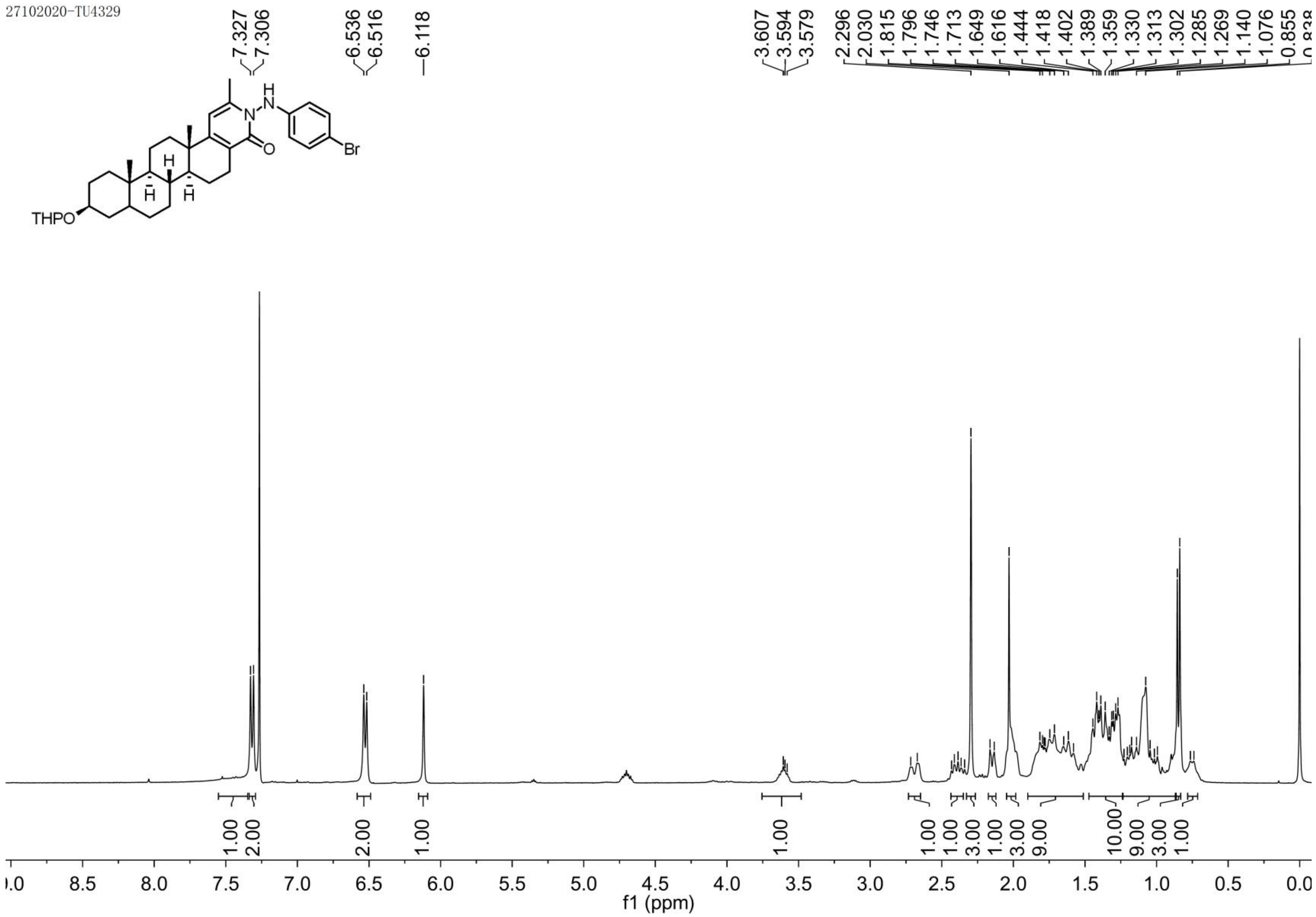

Supplementary Figure 141 ^{1}H NMR Spectrum of Compound **51**

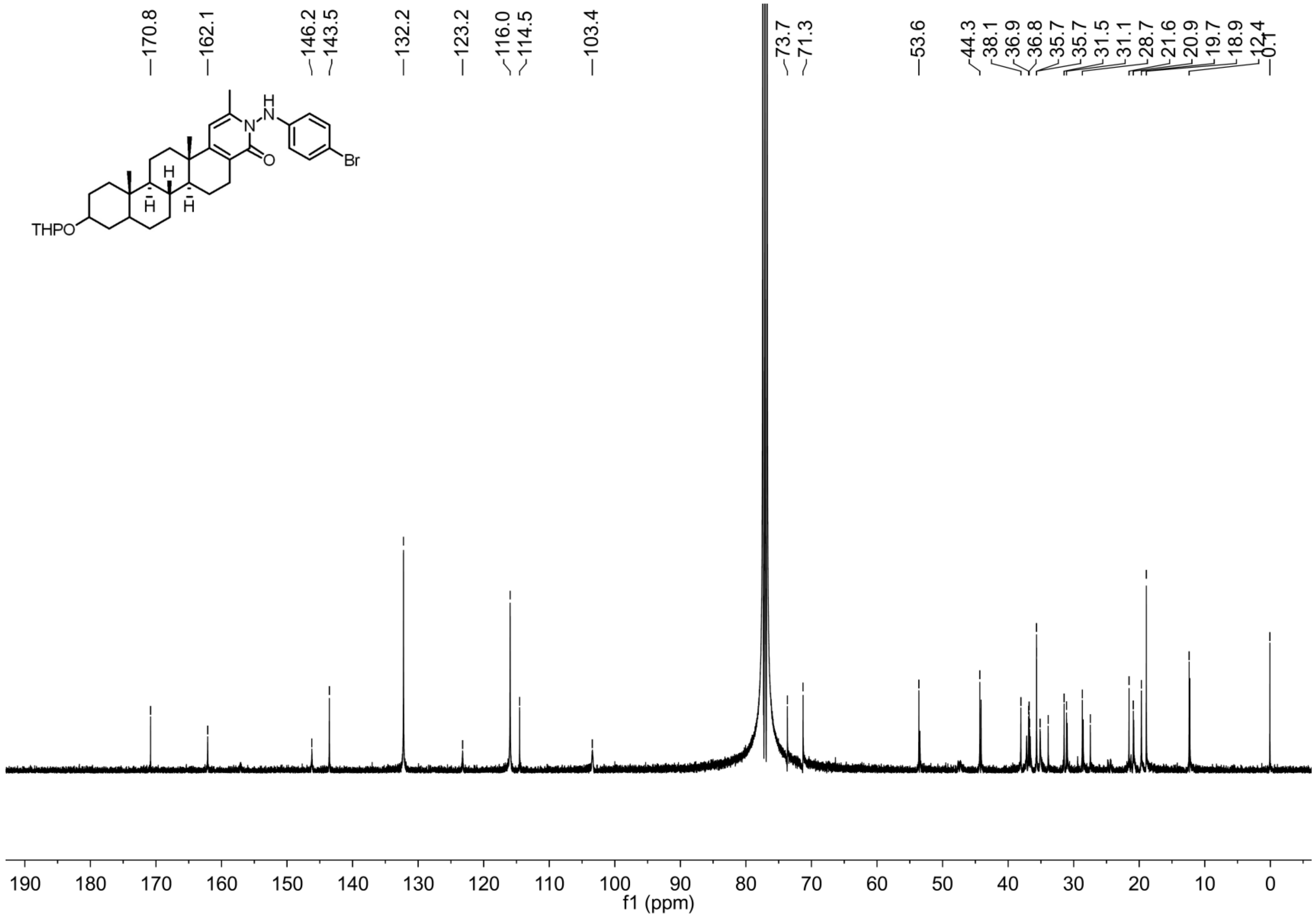

Supplementary Figure 142 ^{13}C NMR Spectrum of Compound **51**

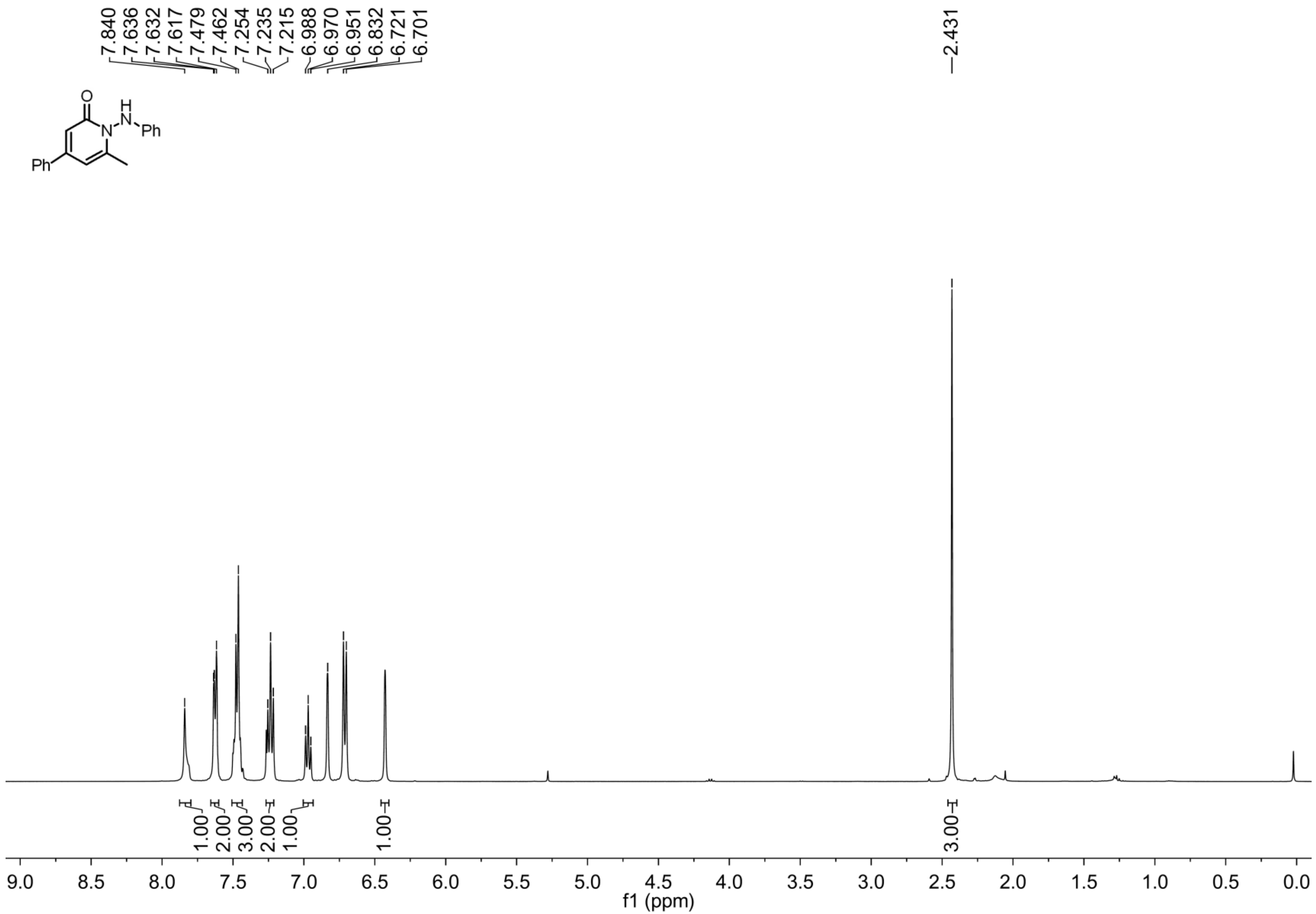

Supplementary Figure 143 ^{1}H NMR Spectrum of Compound **52**

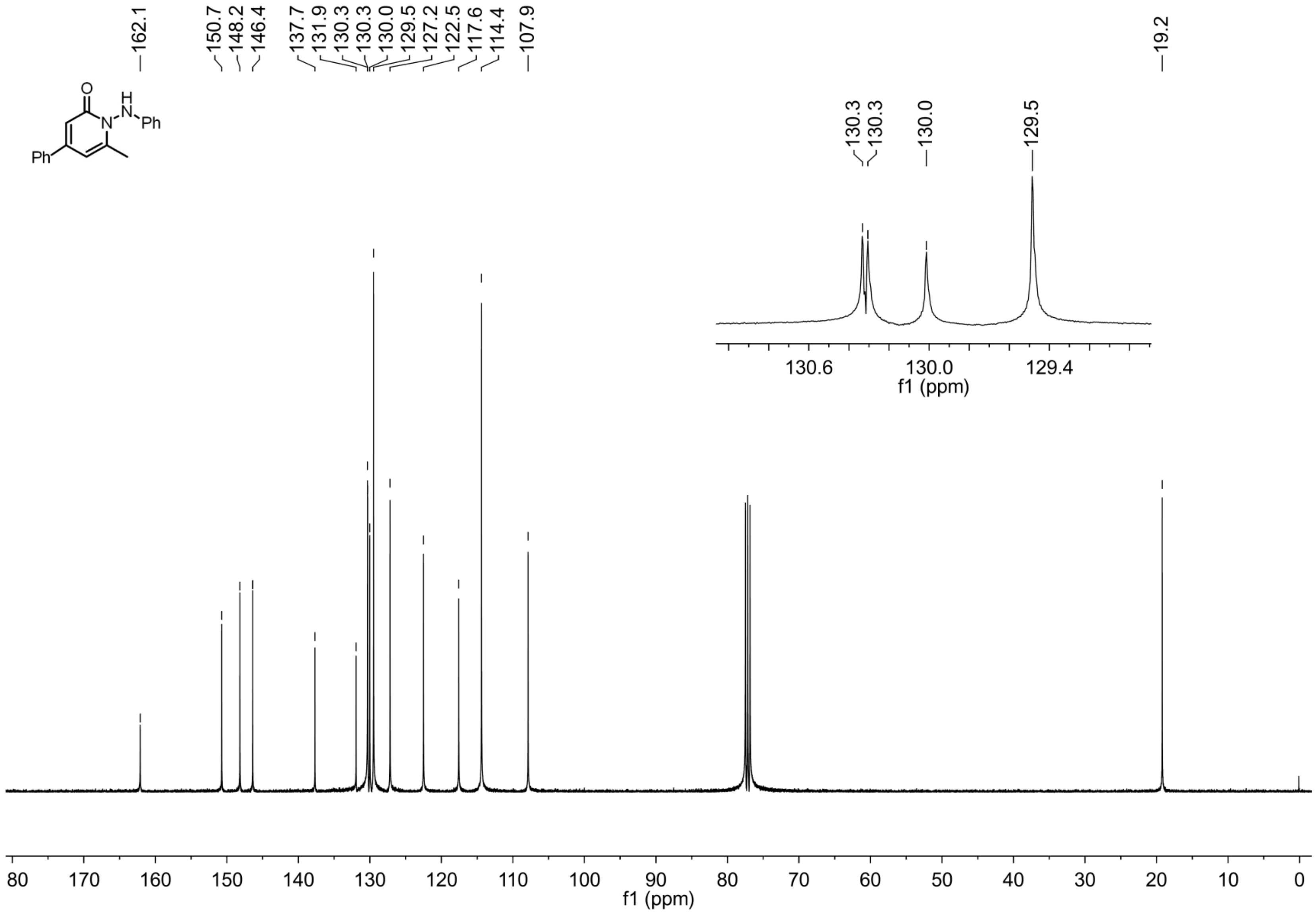

Supplementary Figure 144 ^{13}C NMR Spectrum of Compound **52**

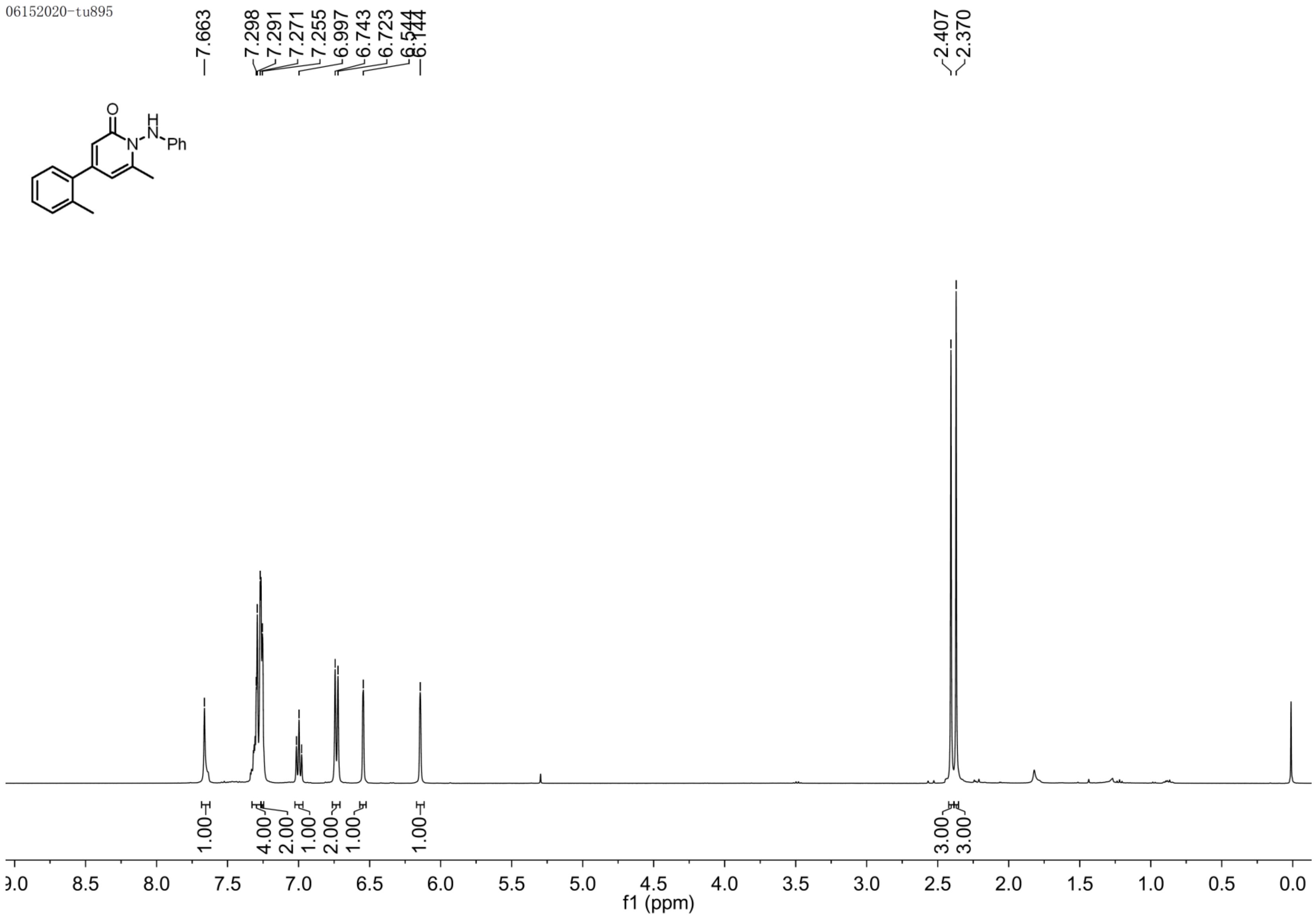

Supplementary Figure 145 ^{1}H NMR Spectrum of Compound **53**

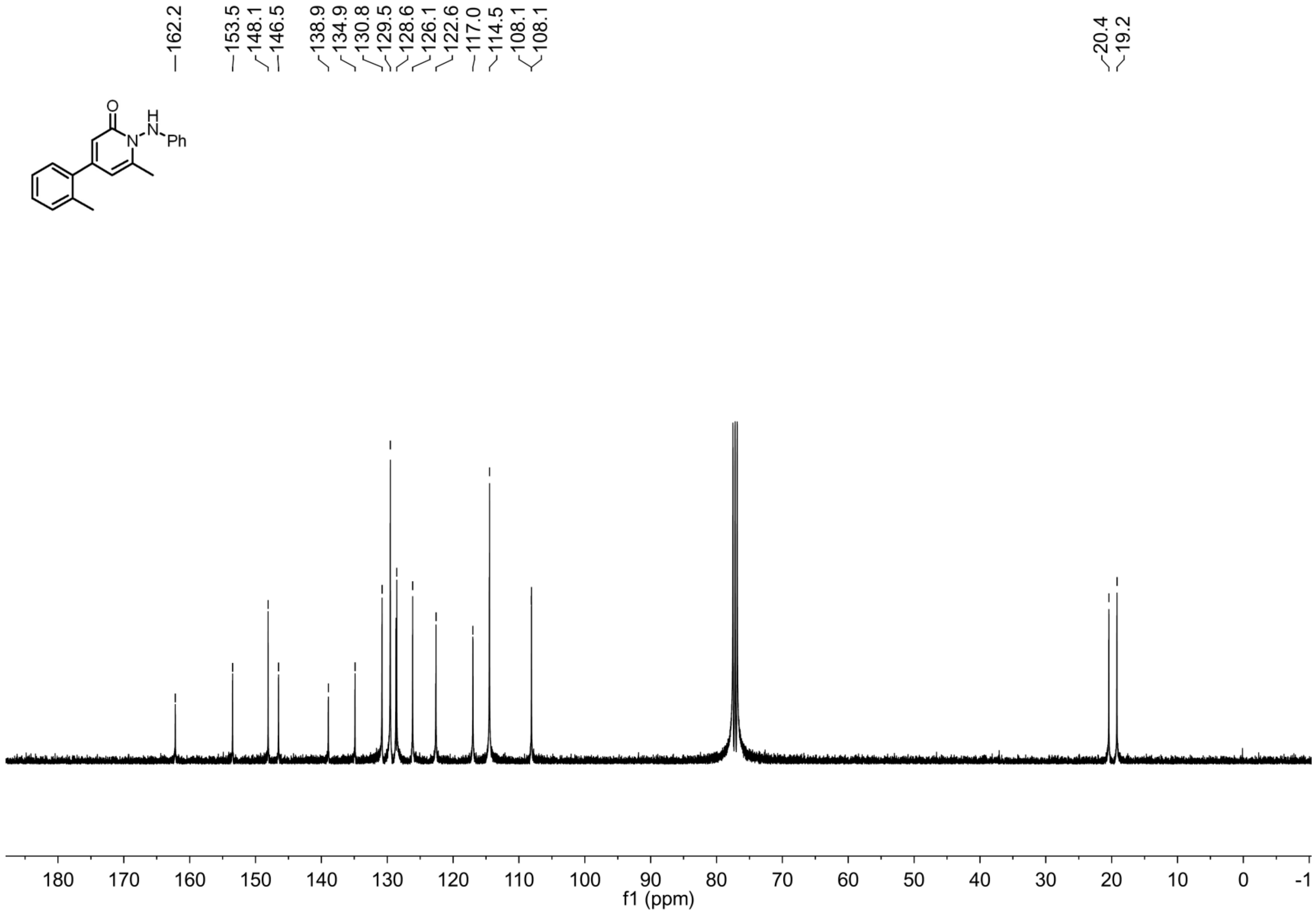

Supplementary Figure 146 ^{13}C NMR Spectrum of Compound **53**

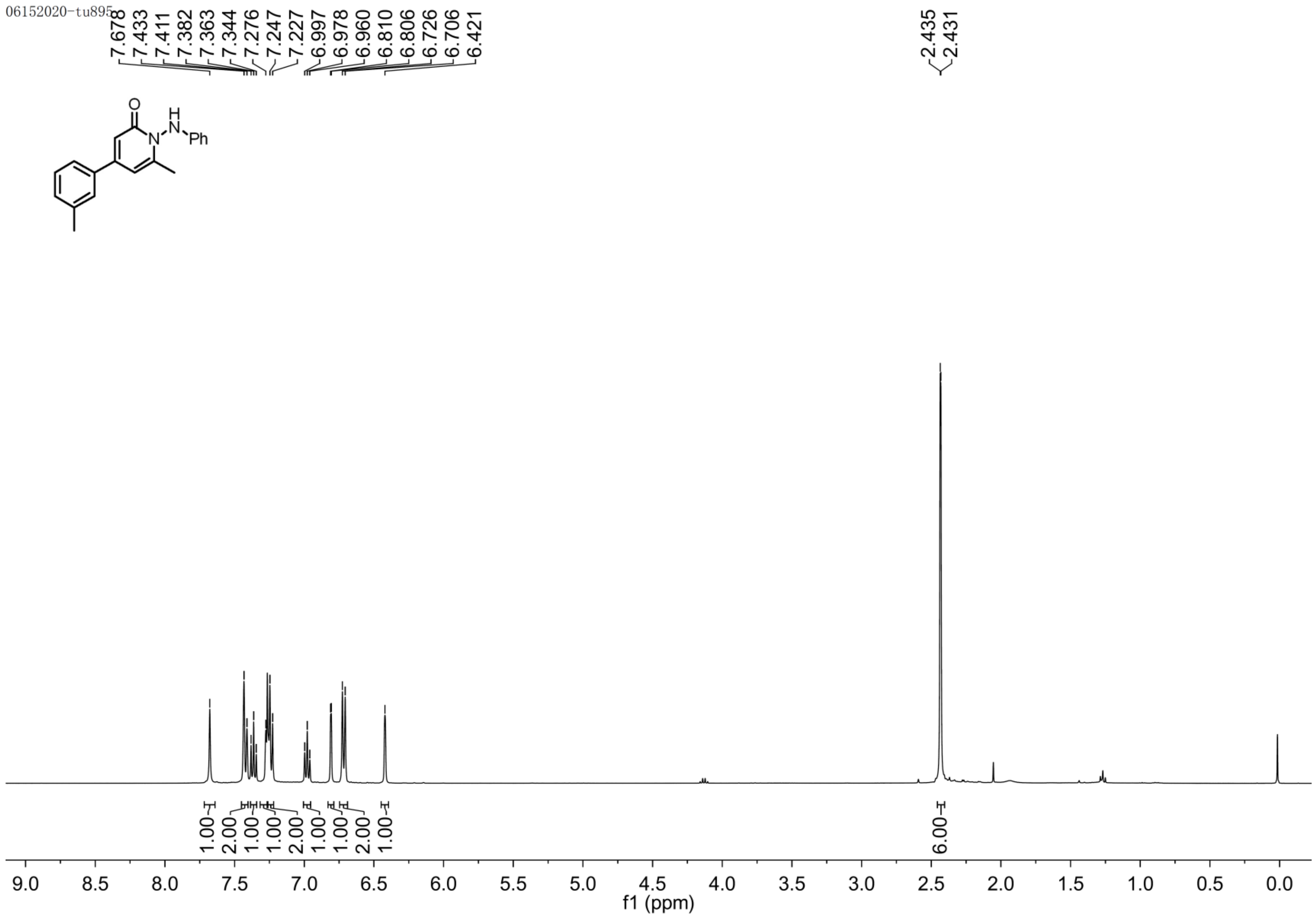

Supplementary Figure 147 ^{1}H NMR Spectrum of Compound **54**

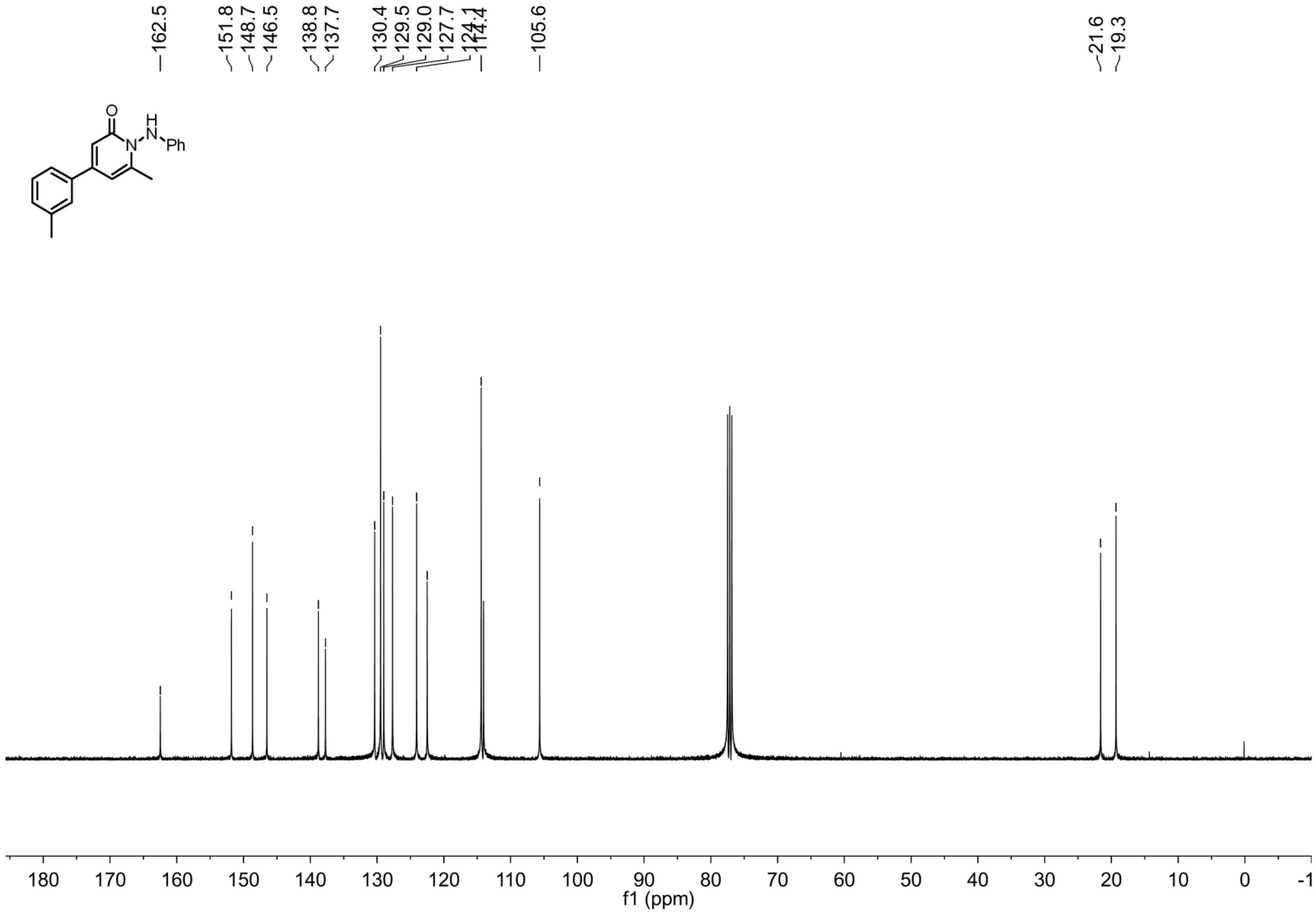

Supplementary Figure 148 ^{13}C NMR Spectrum of Compound **54**

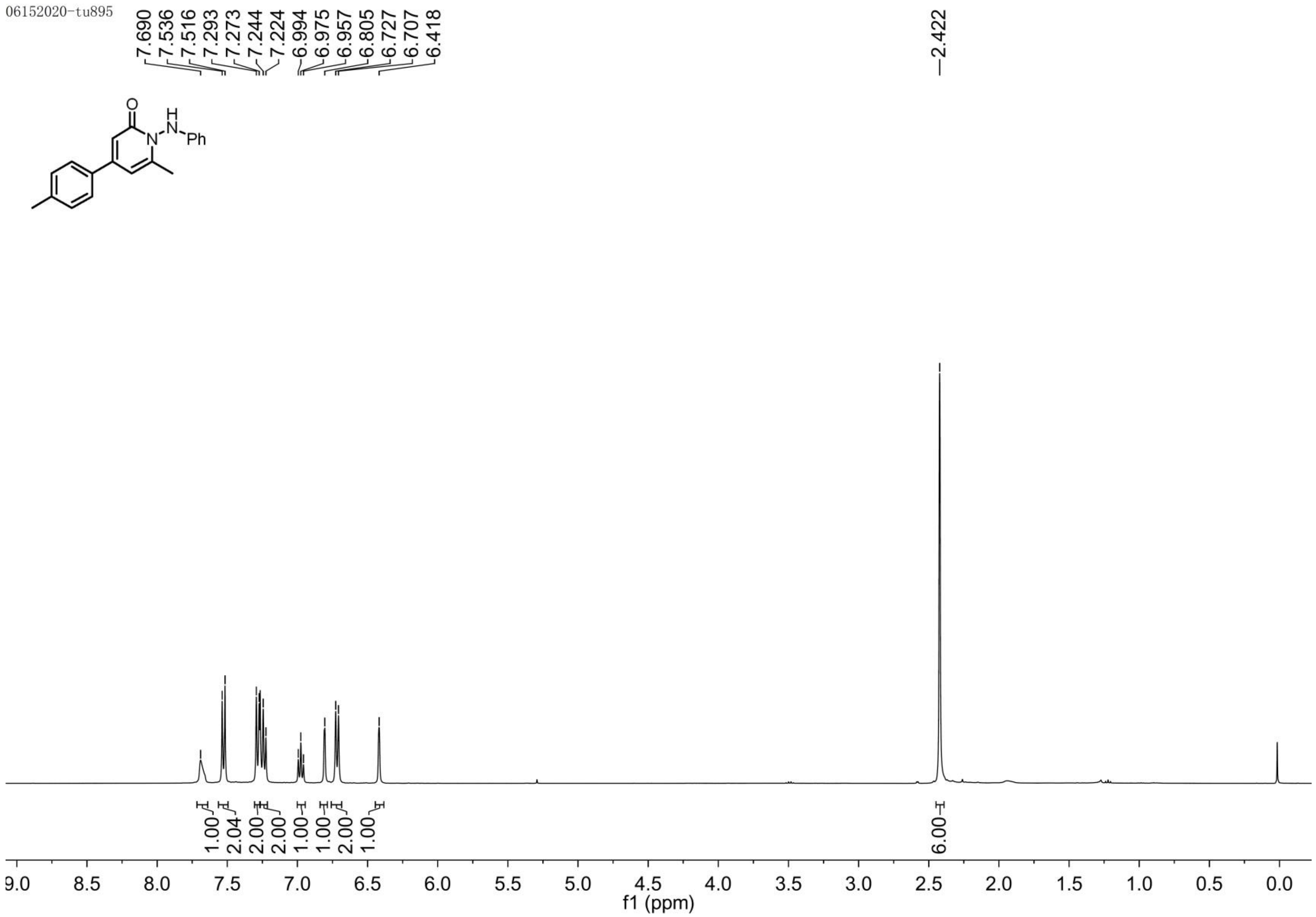

Supplementary Figure 149 ^{1}H NMR Spectrum of Compound **55**

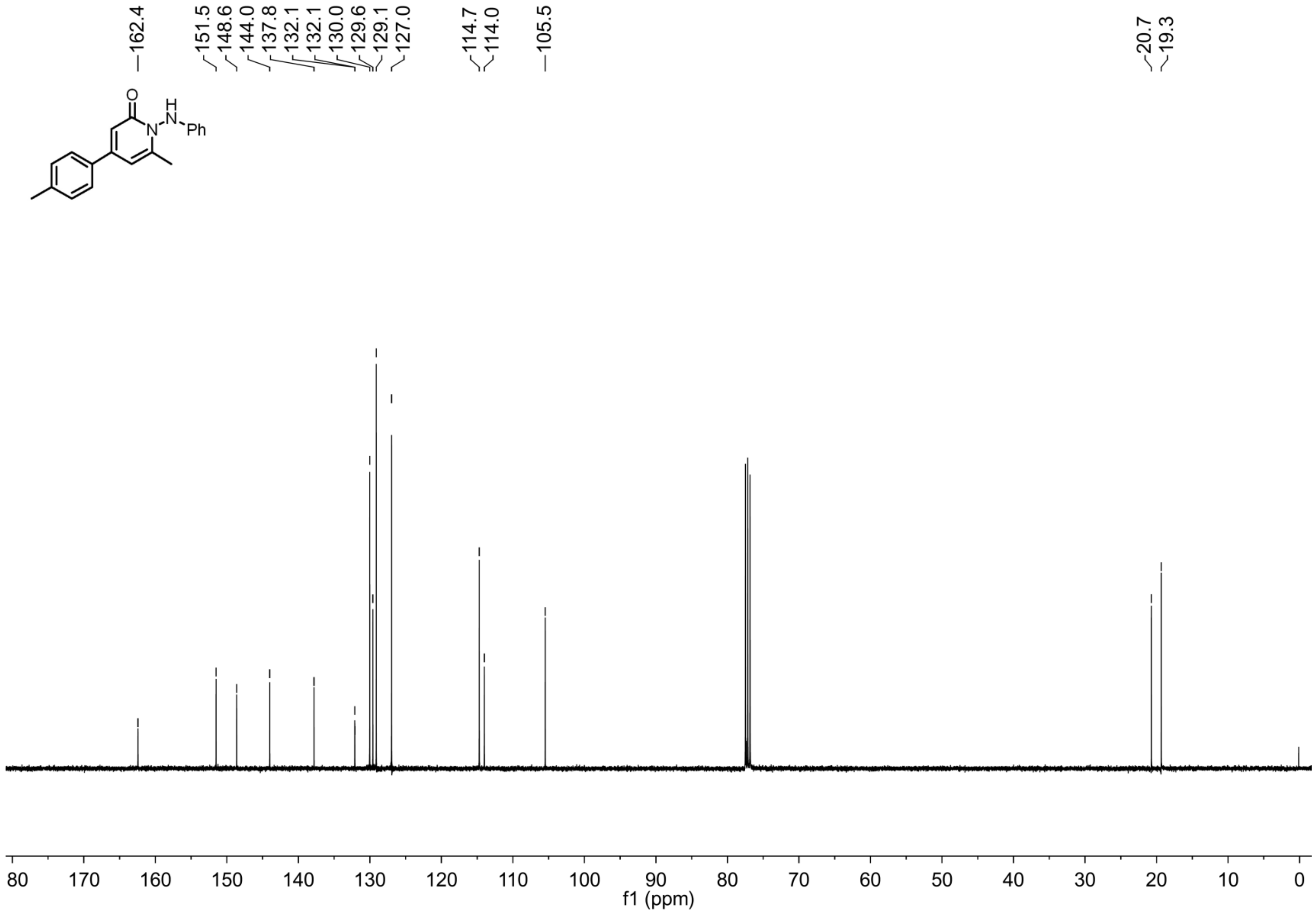

Supplementary Figure 150 ^{13}C NMR Spectrum of Compound **55**

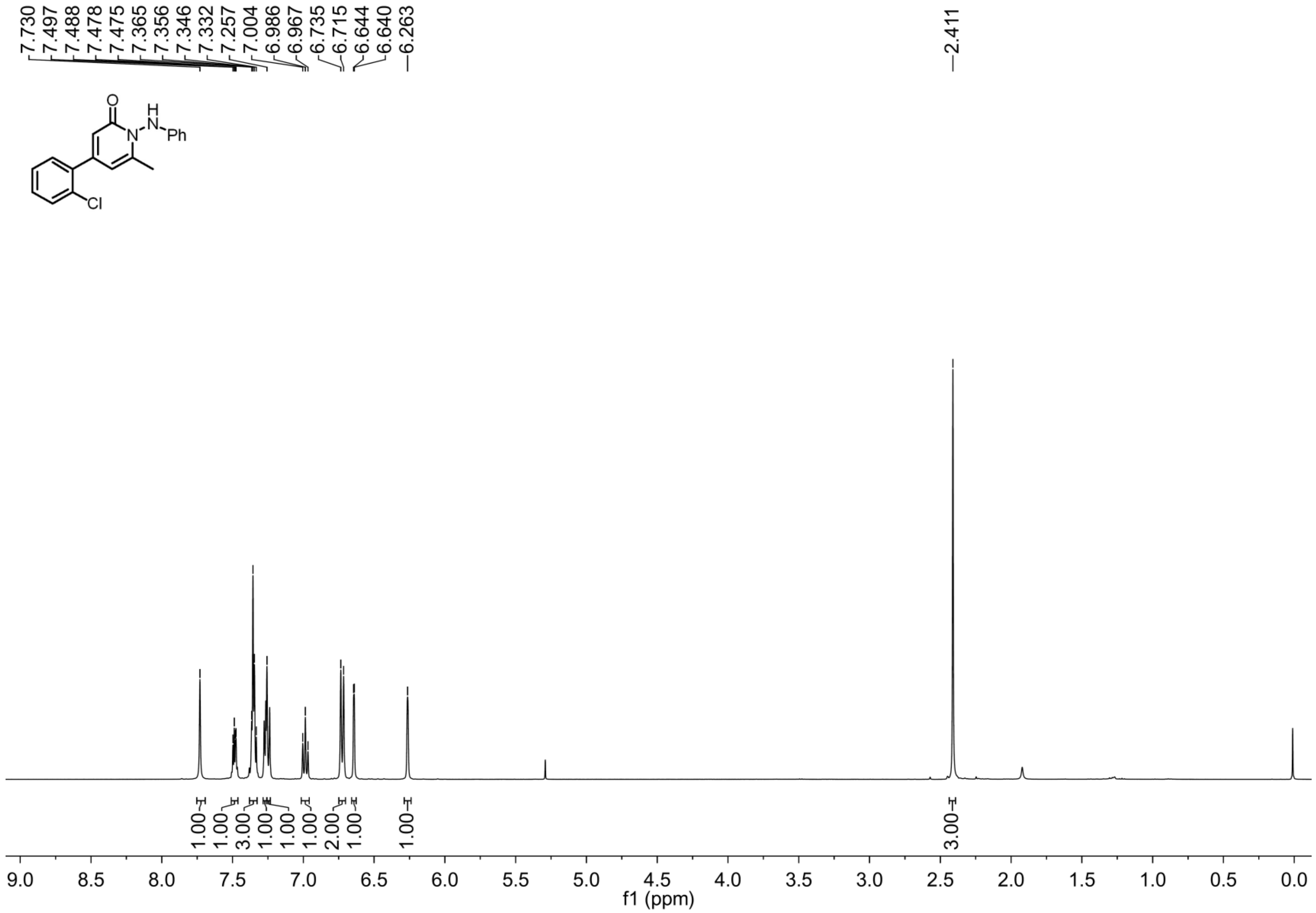

Supplementary Figure 151 ^{1}H NMR Spectrum of Compound **56**

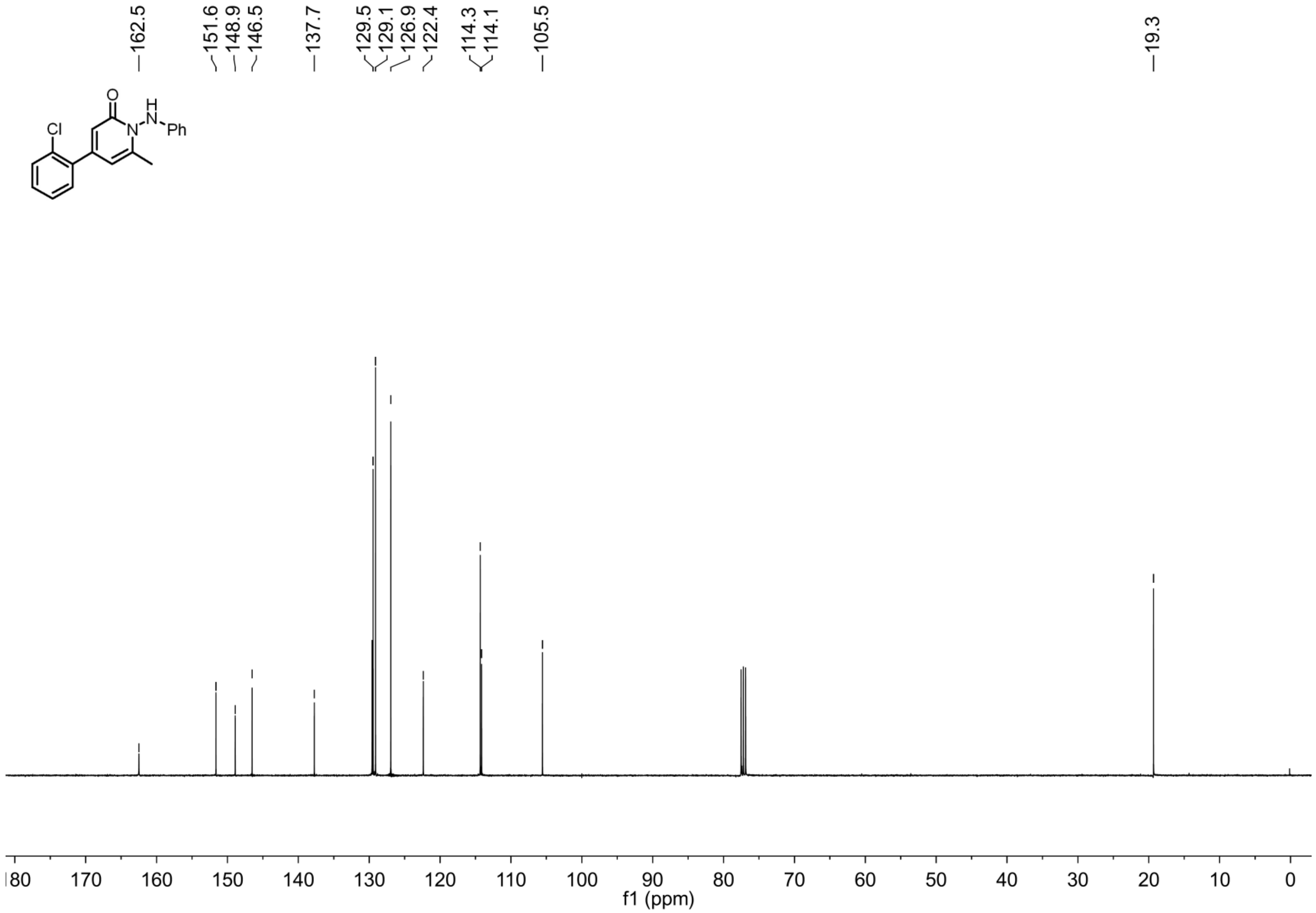

Supplementary Figure 152 ^{13}C NMR Spectrum of Compound **56**

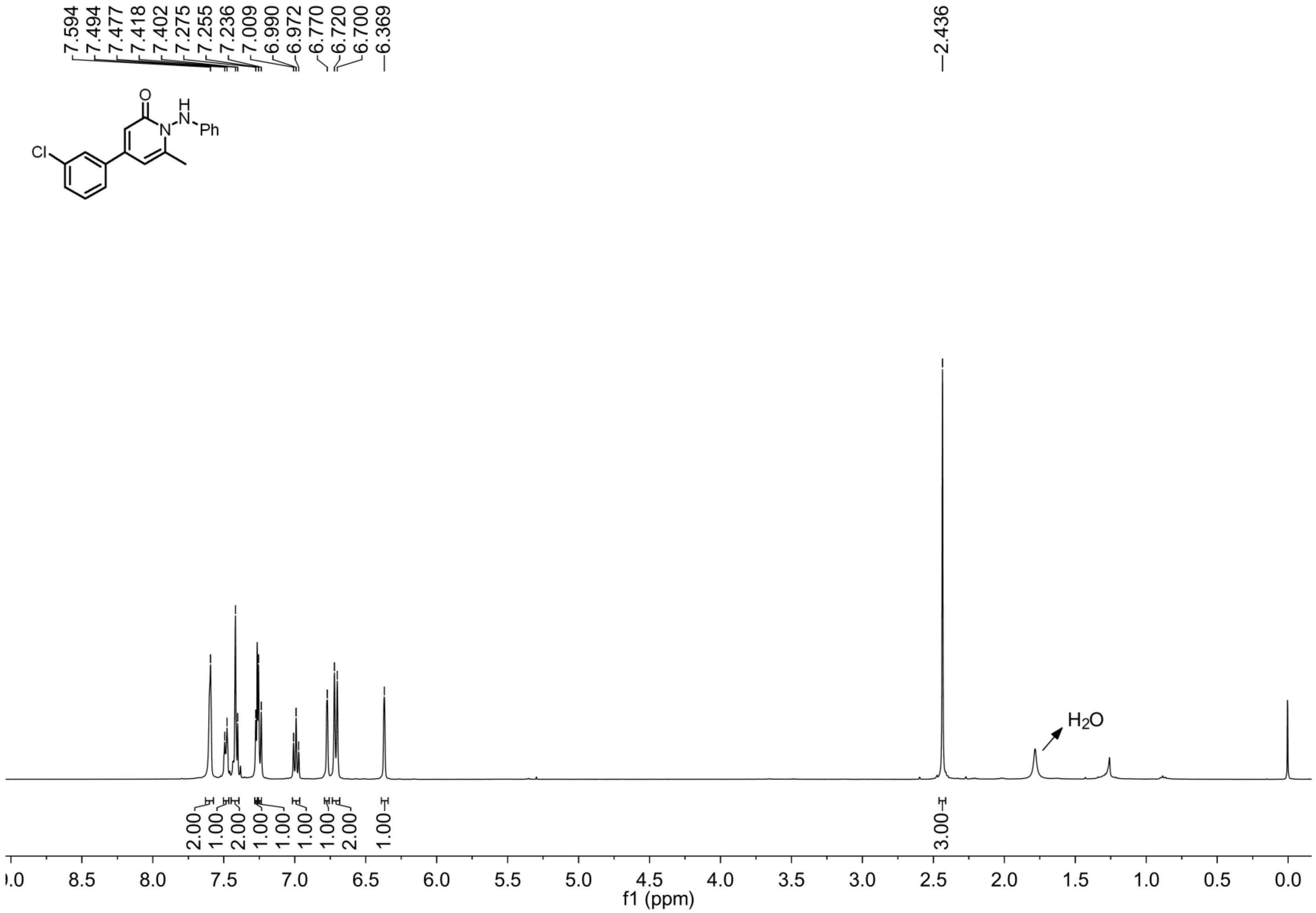

Supplementary Figure 153 ^{1}H NMR Spectrum of Compound **57**

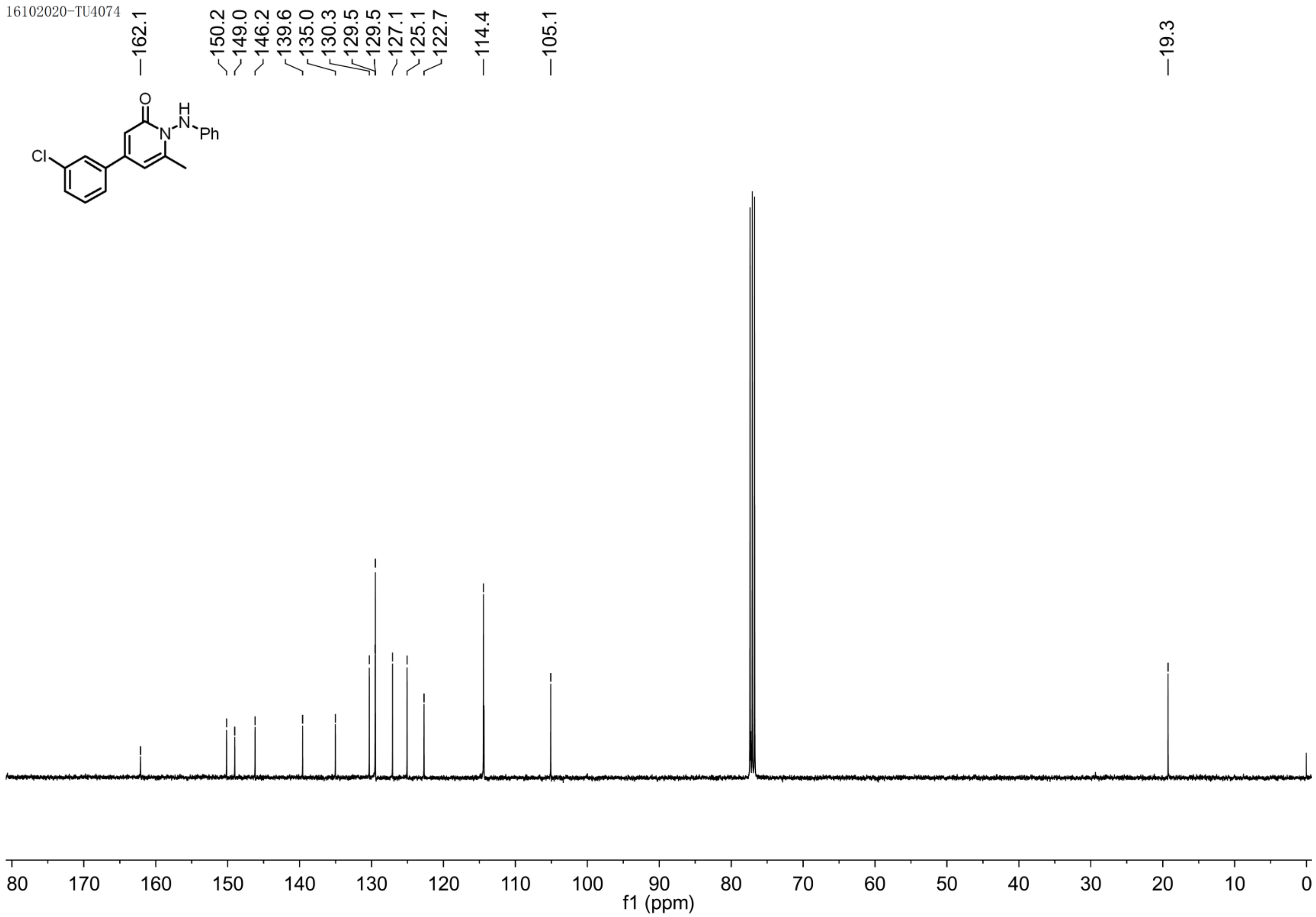

Supplementary Figure 154 ^{13}C NMR Spectrum of Compound **57**

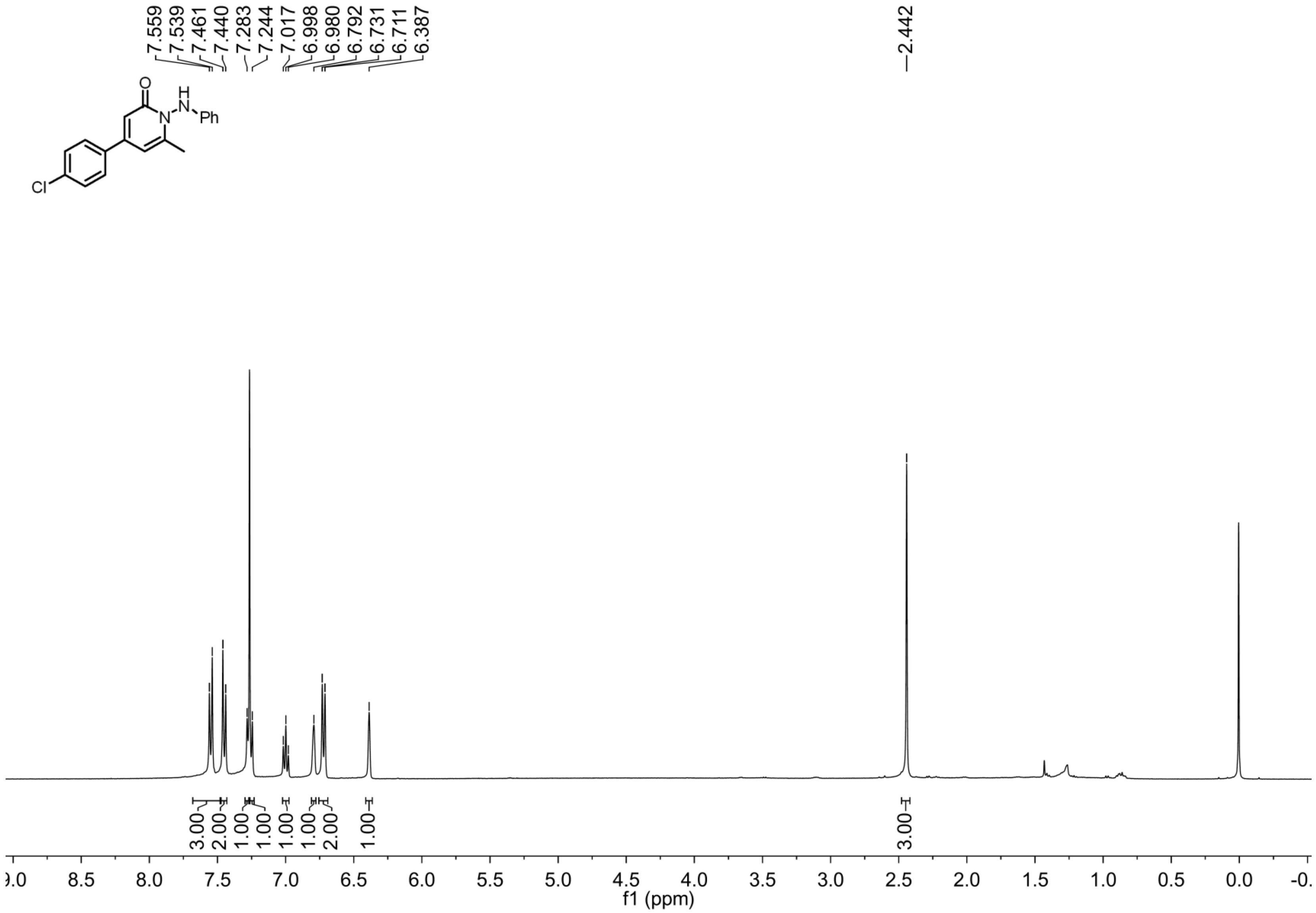

Supplementary Figure 155 ^{1}H NMR Spectrum of Compound **58**

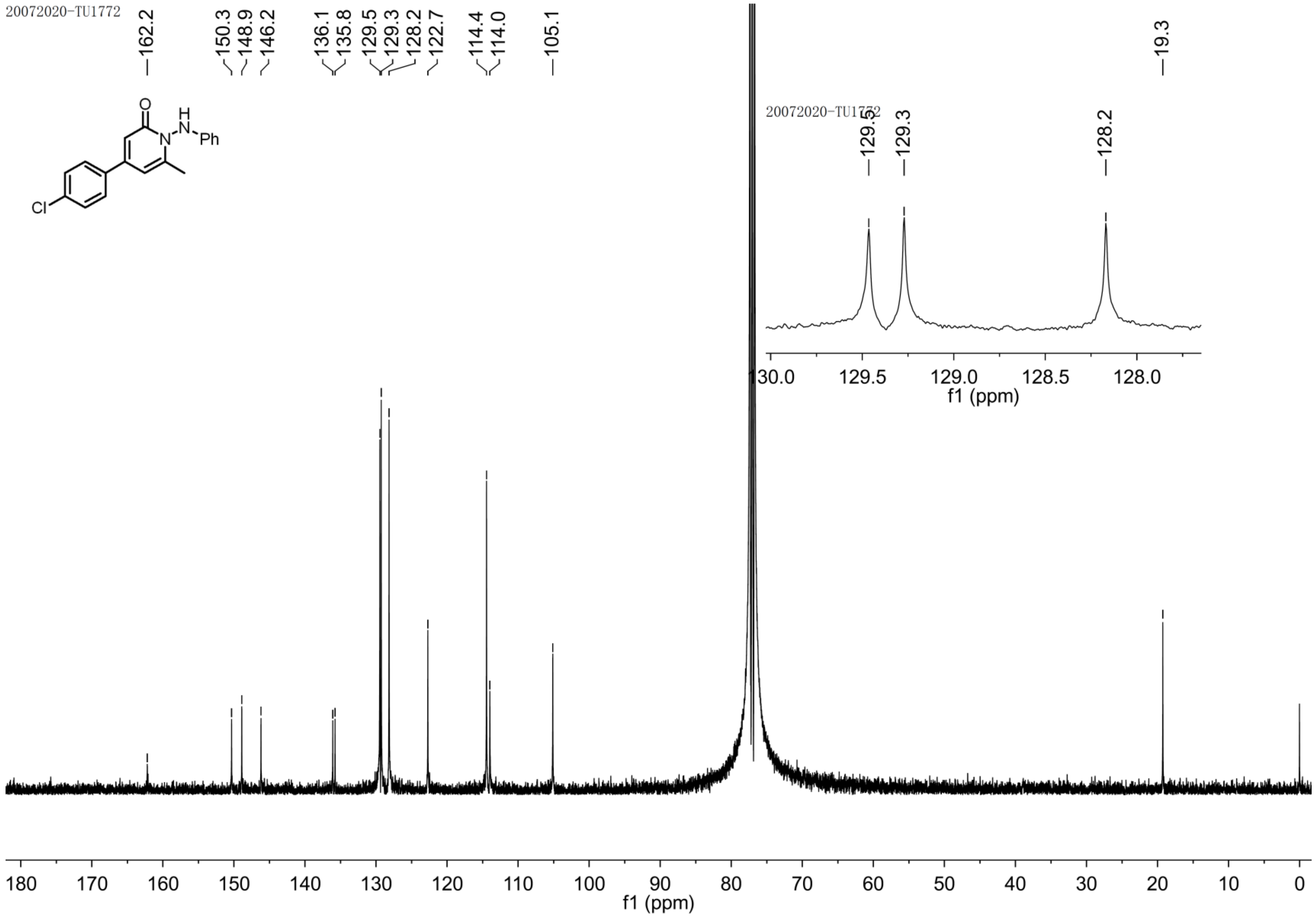

Supplementary Figure 156 ^{13}C NMR Spectrum of Compound **58**

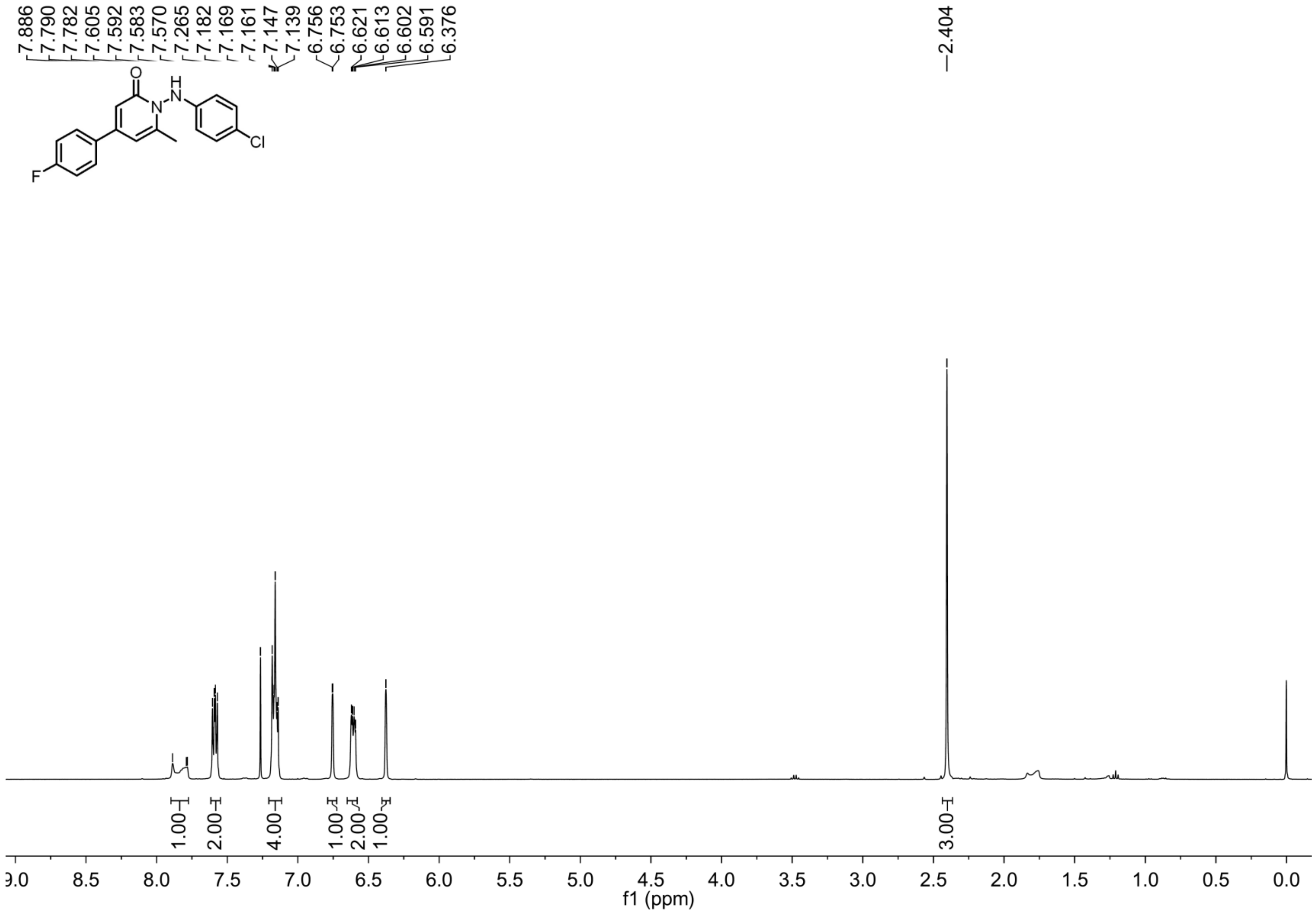

Supplementary Figure 157 ^{1}H NMR Spectrum of Compound **59**

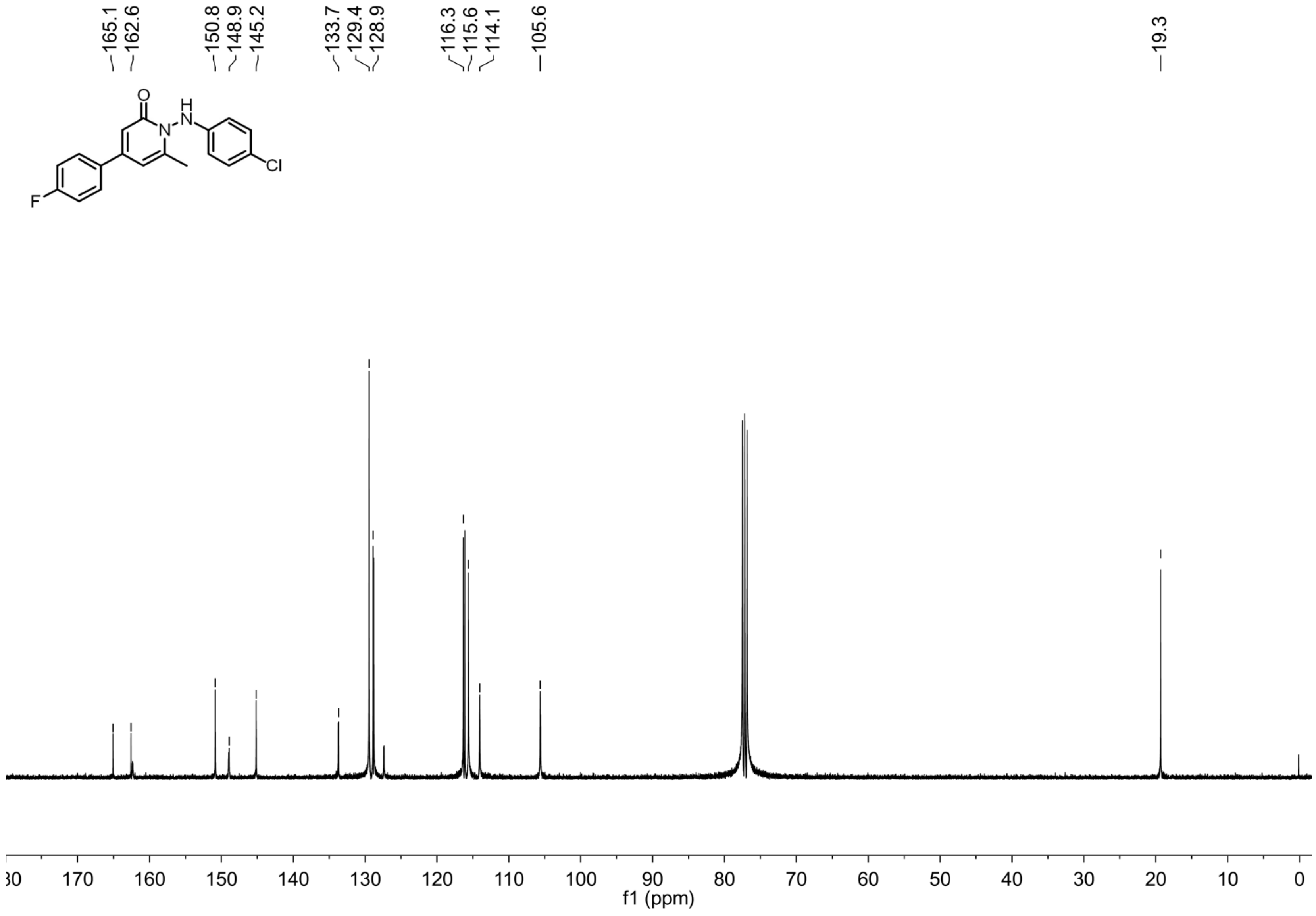

Supplementary Figure 158 ^{13}C NMR Spectrum of Compound **59**

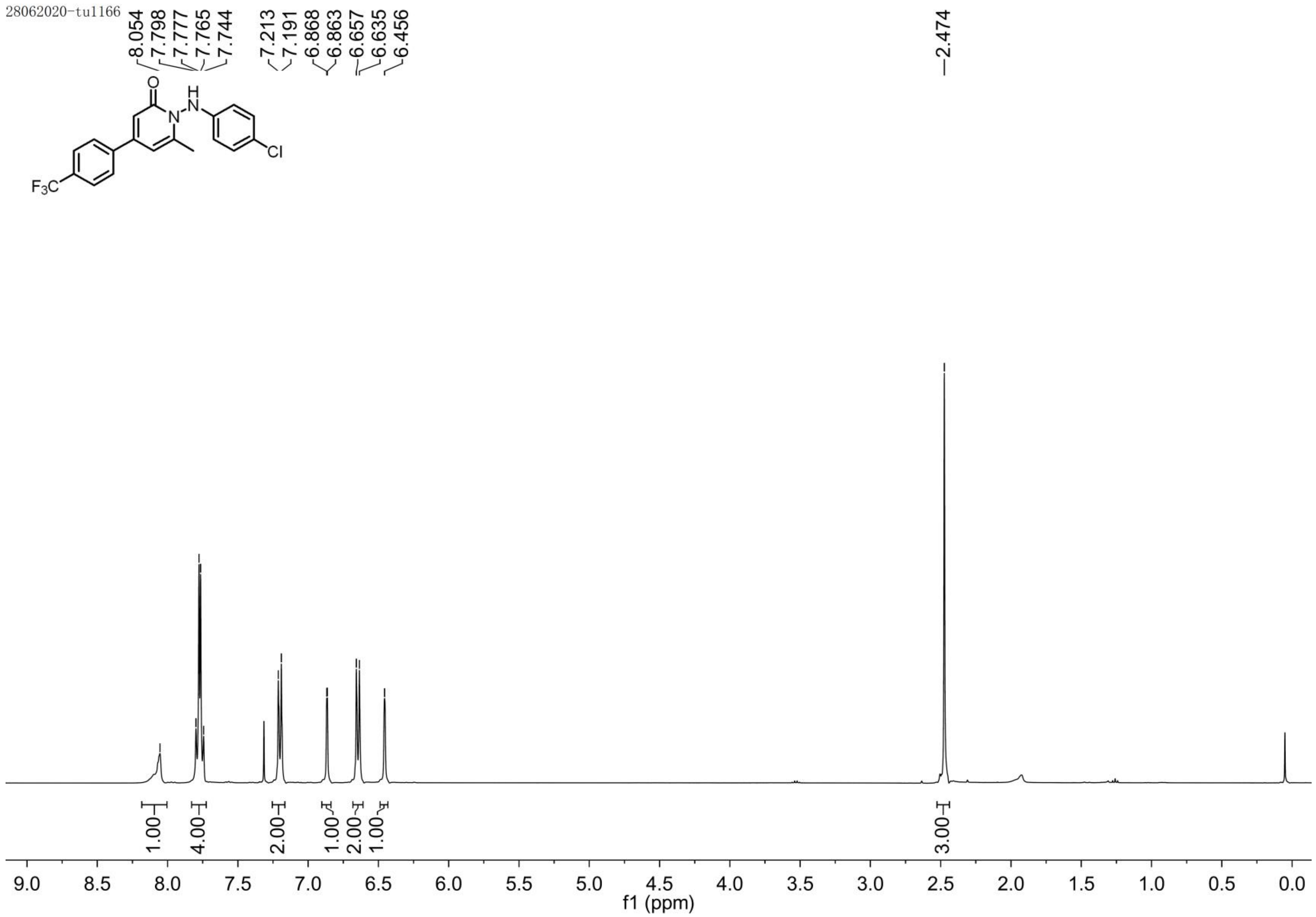

Supplementary Figure 159 ^{1}H NMR Spectrum of Compound **60**

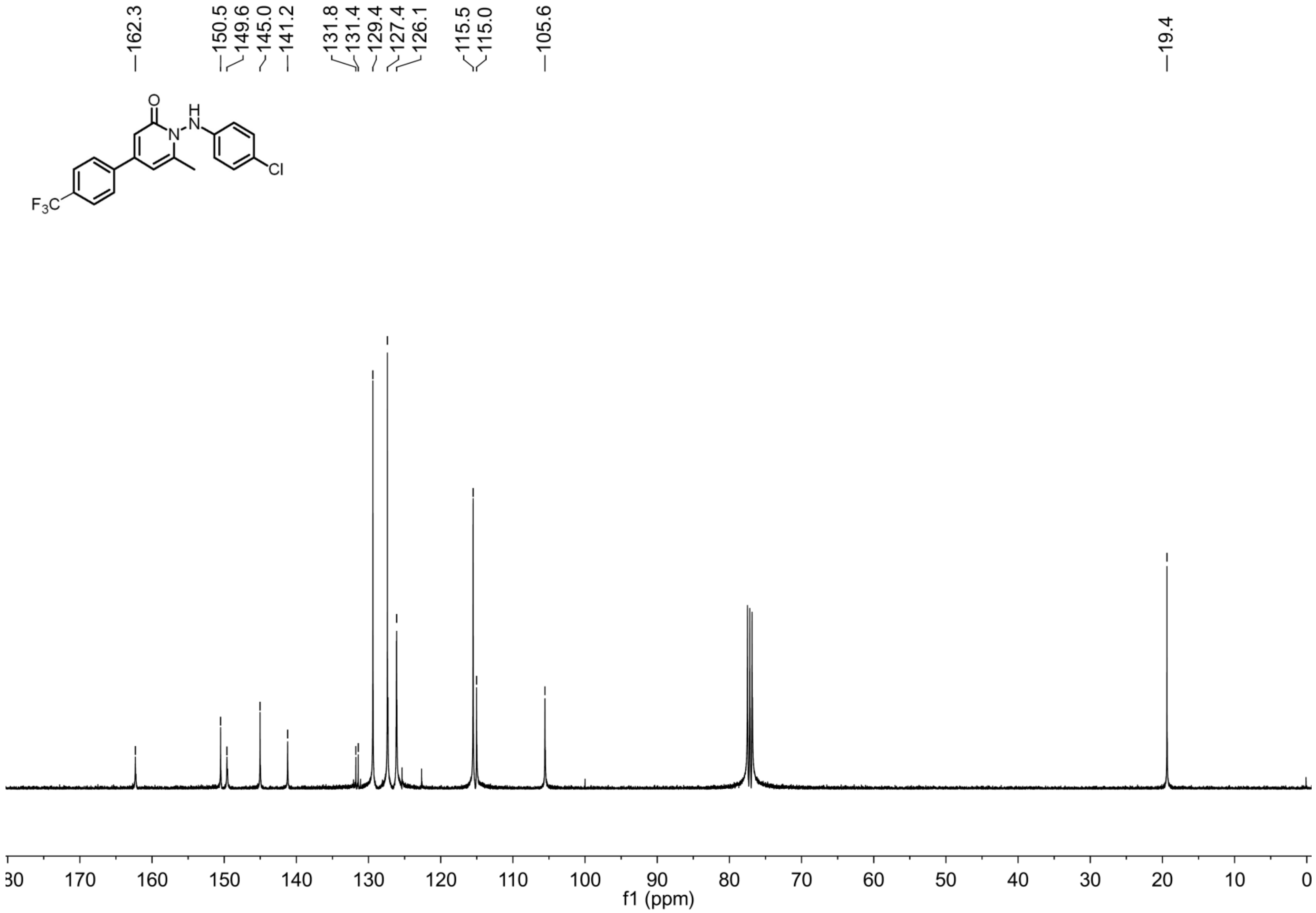

Supplementary Figure 160 ^{13}C NMR Spectrum of Compound **60**

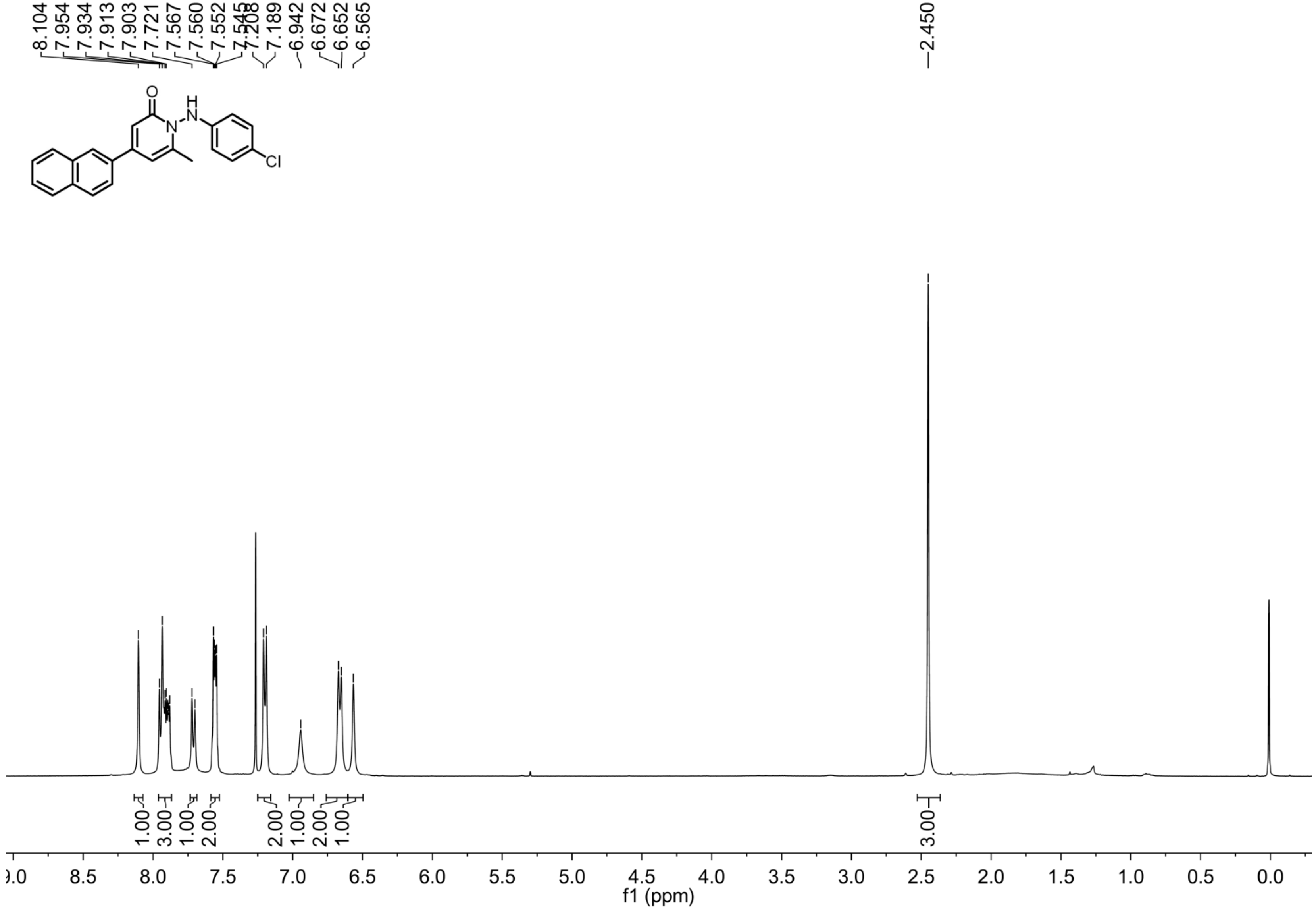

Supplementary Figure 161 ^{1}H NMR Spectrum of Compound **61**

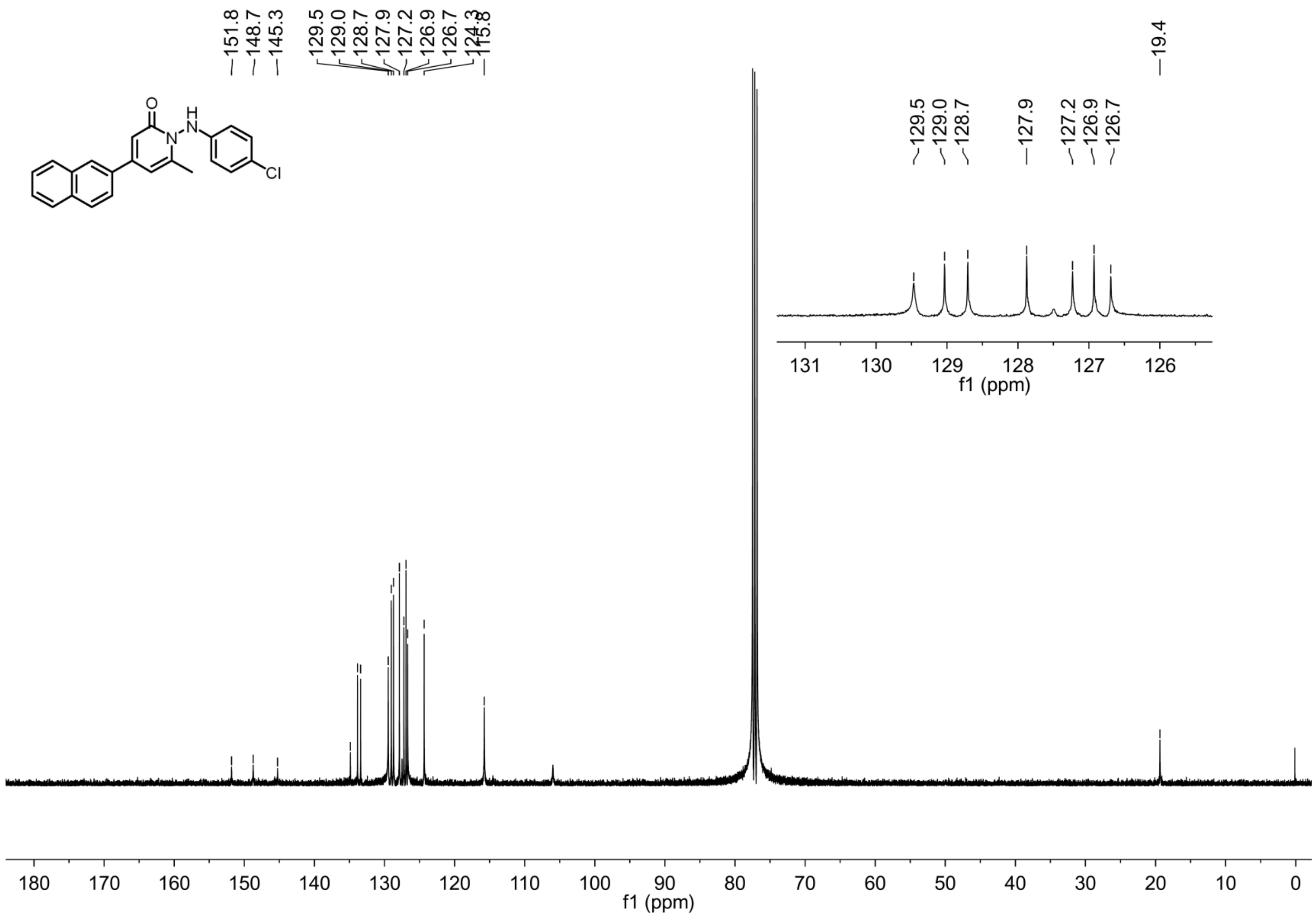

Supplementary Figure 162 ^{13}C NMR Spectrum of Compound **61**

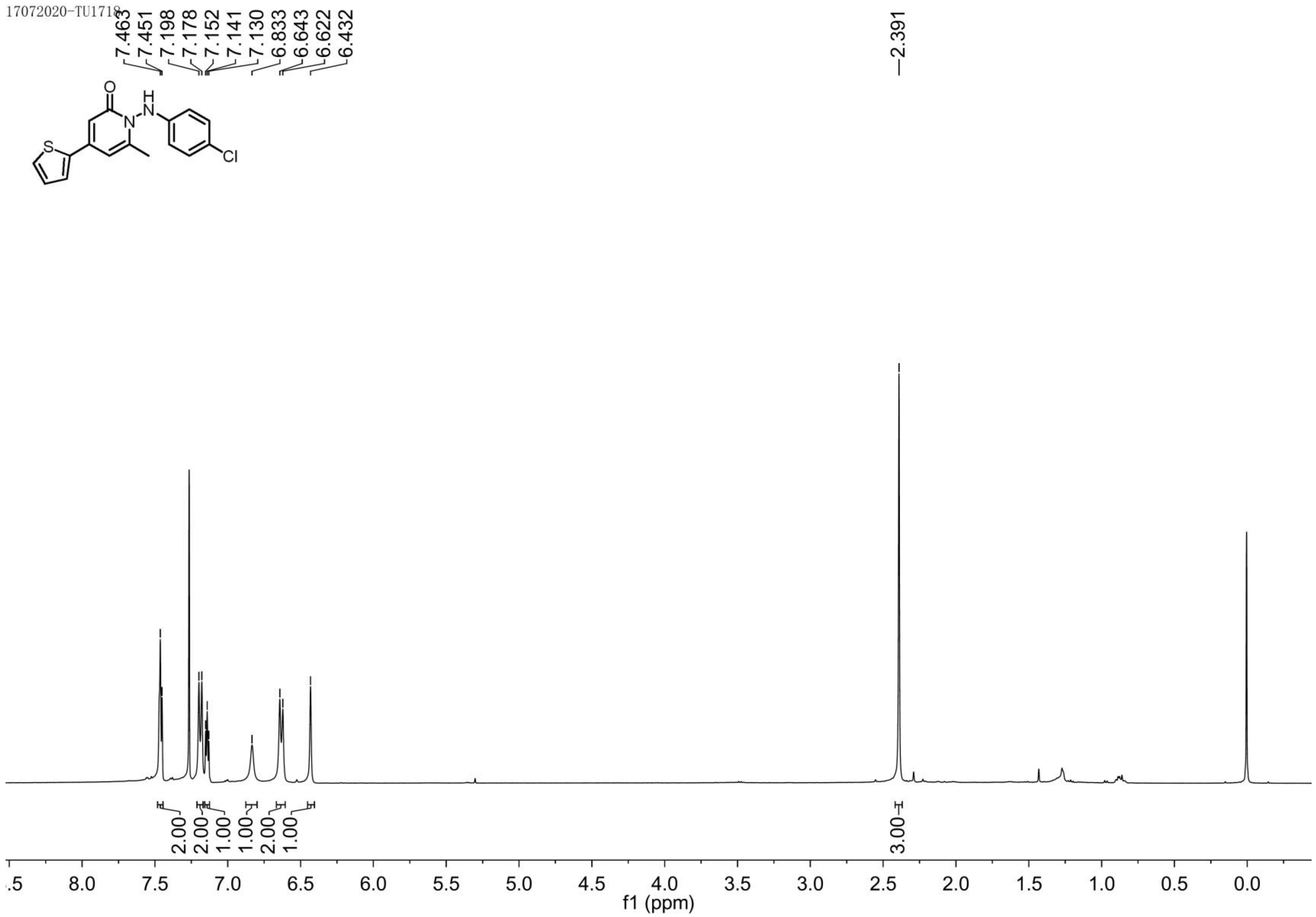

Supplementary Figure 163 ^{1}H NMR Spectrum of Compound **62**

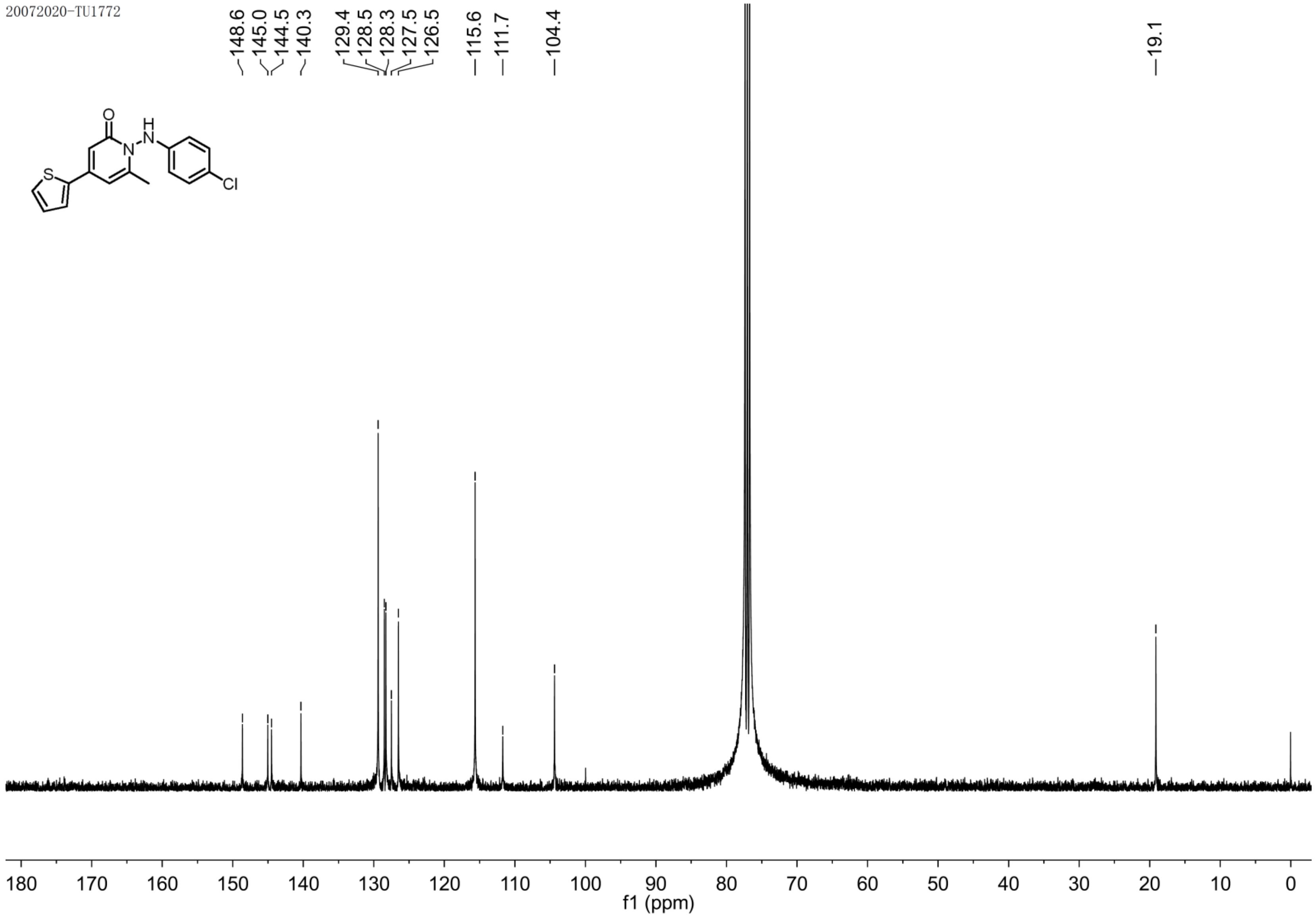

Supplementary Figure 164 ^{13}C NMR Spectrum of Compound **62**

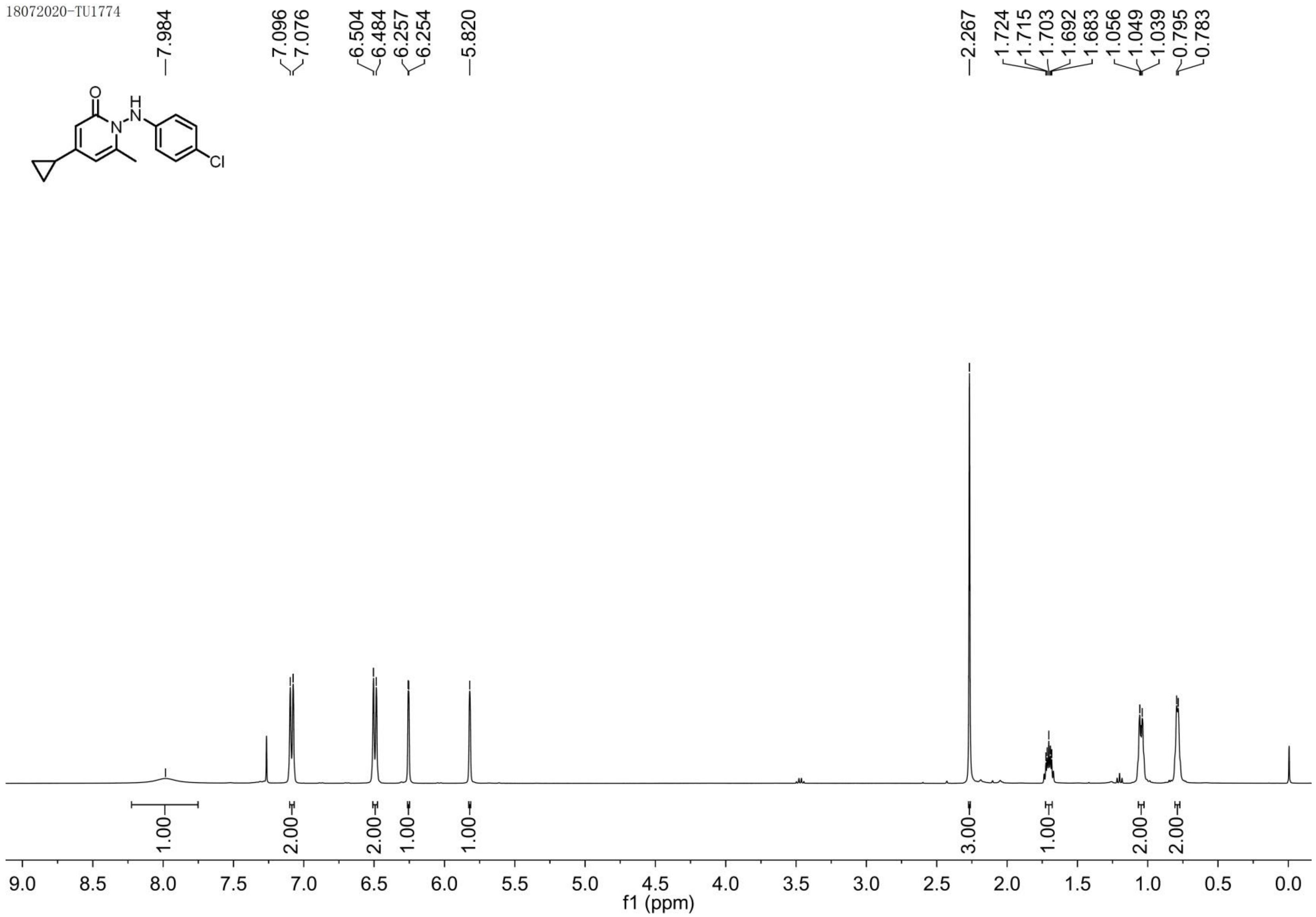

Supplementary Figure 165 ^{1}H NMR Spectrum of Compound **63**

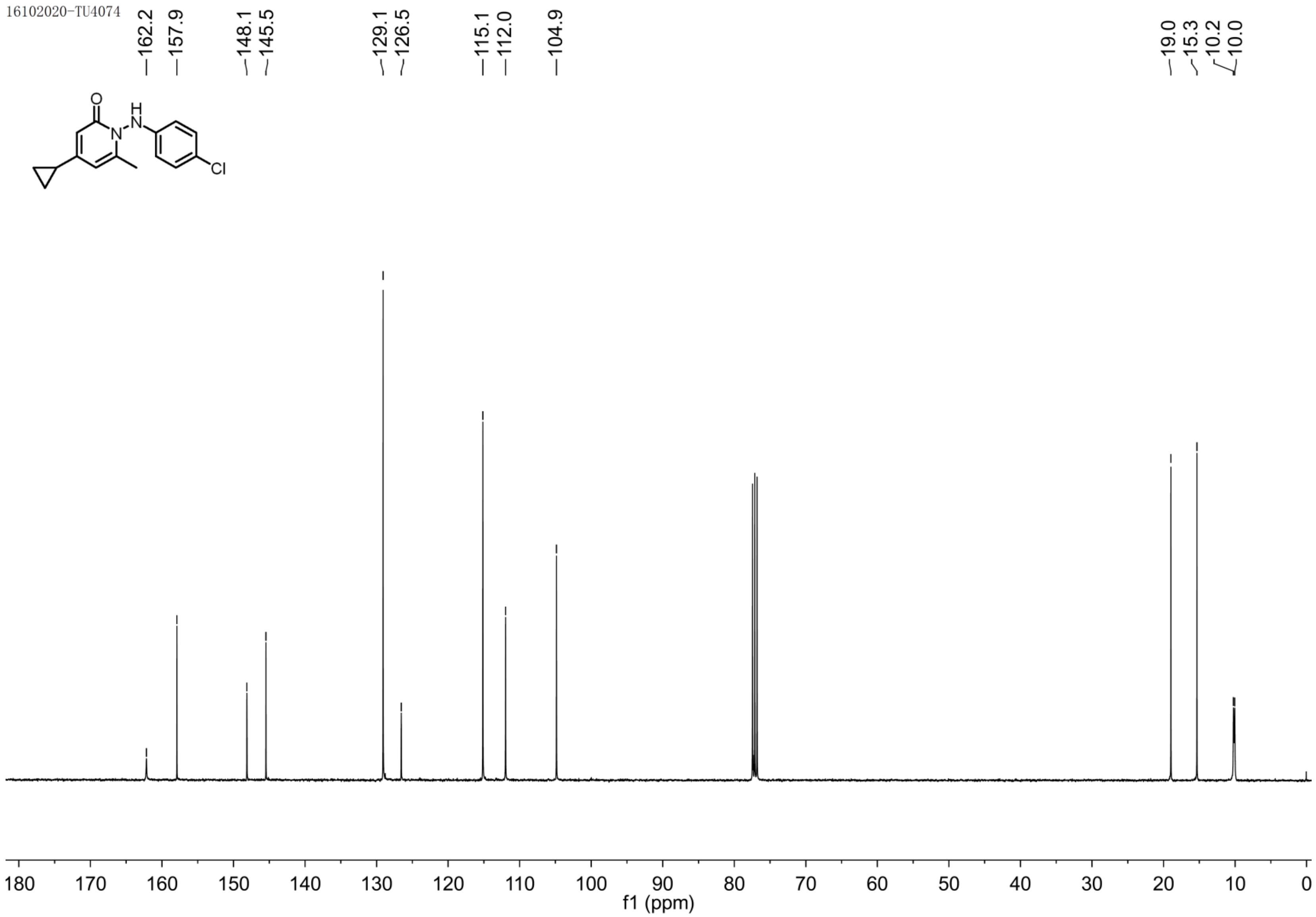

Supplementary Figure 166 ^{13}C NMR Spectrum of Compound **63**

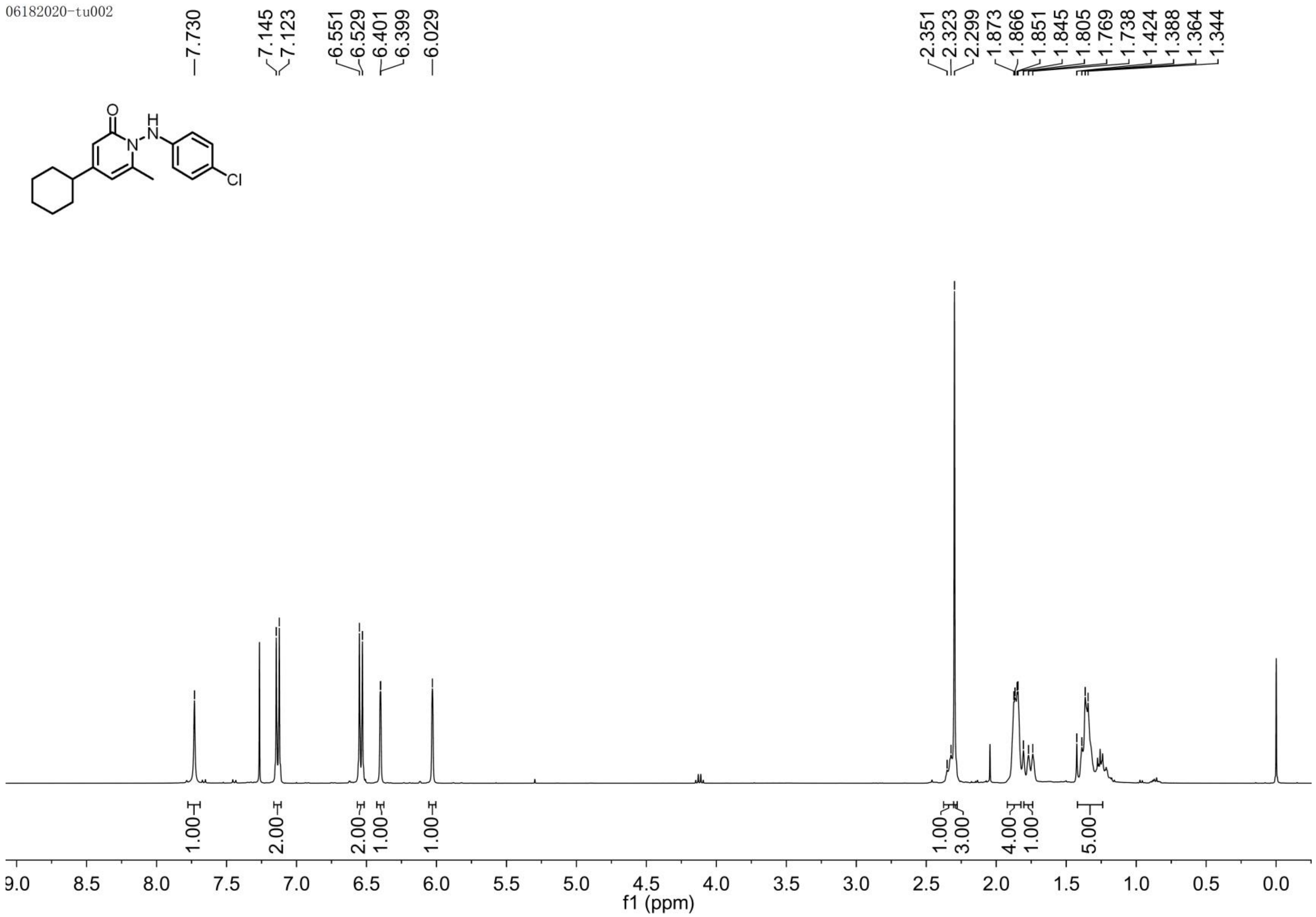

Supplementary Figure 167 ^{1}H NMR Spectrum of Compound **64**

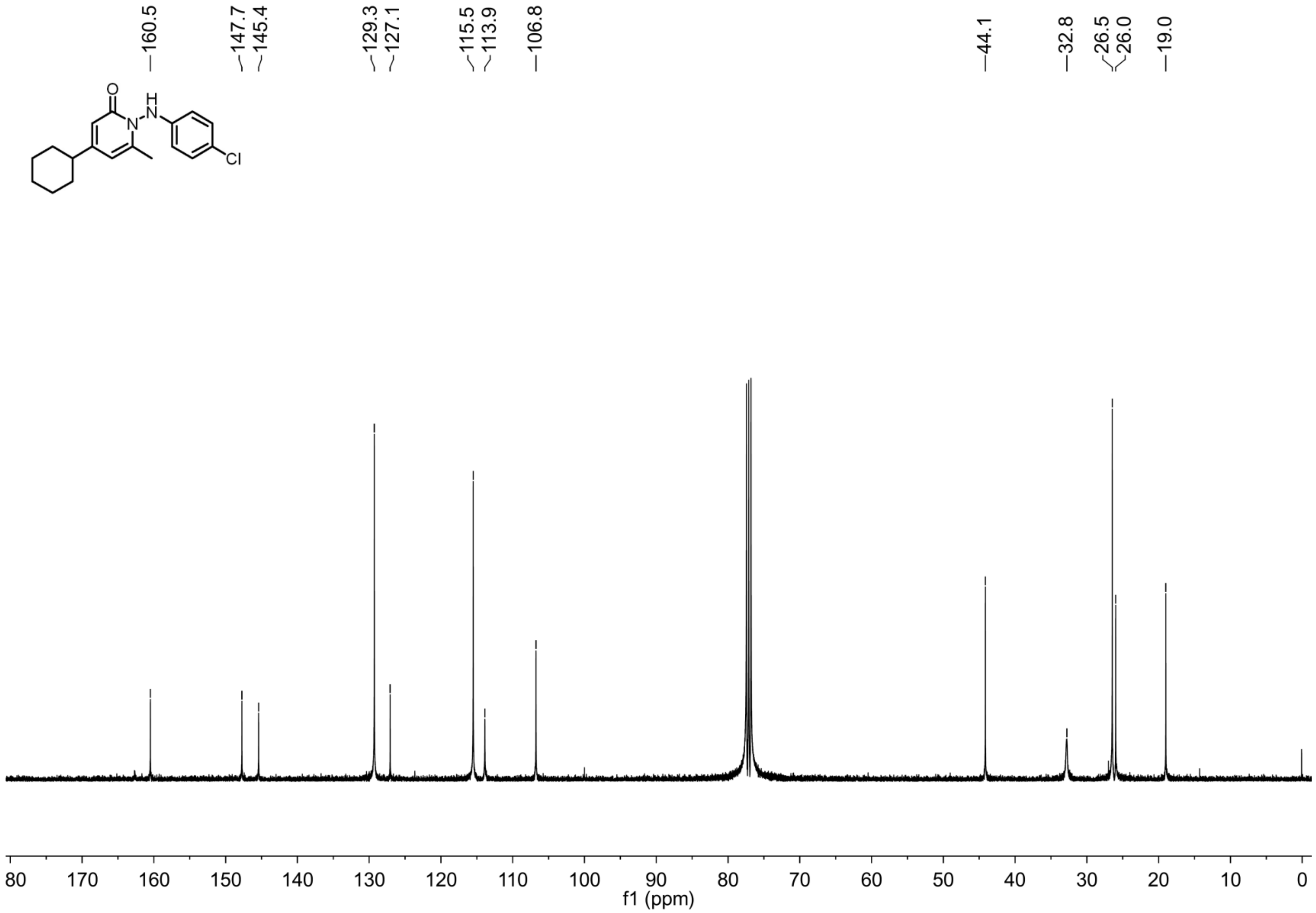

Supplementary Figure 168 ^{13}C NMR Spectrum of Compound **64**

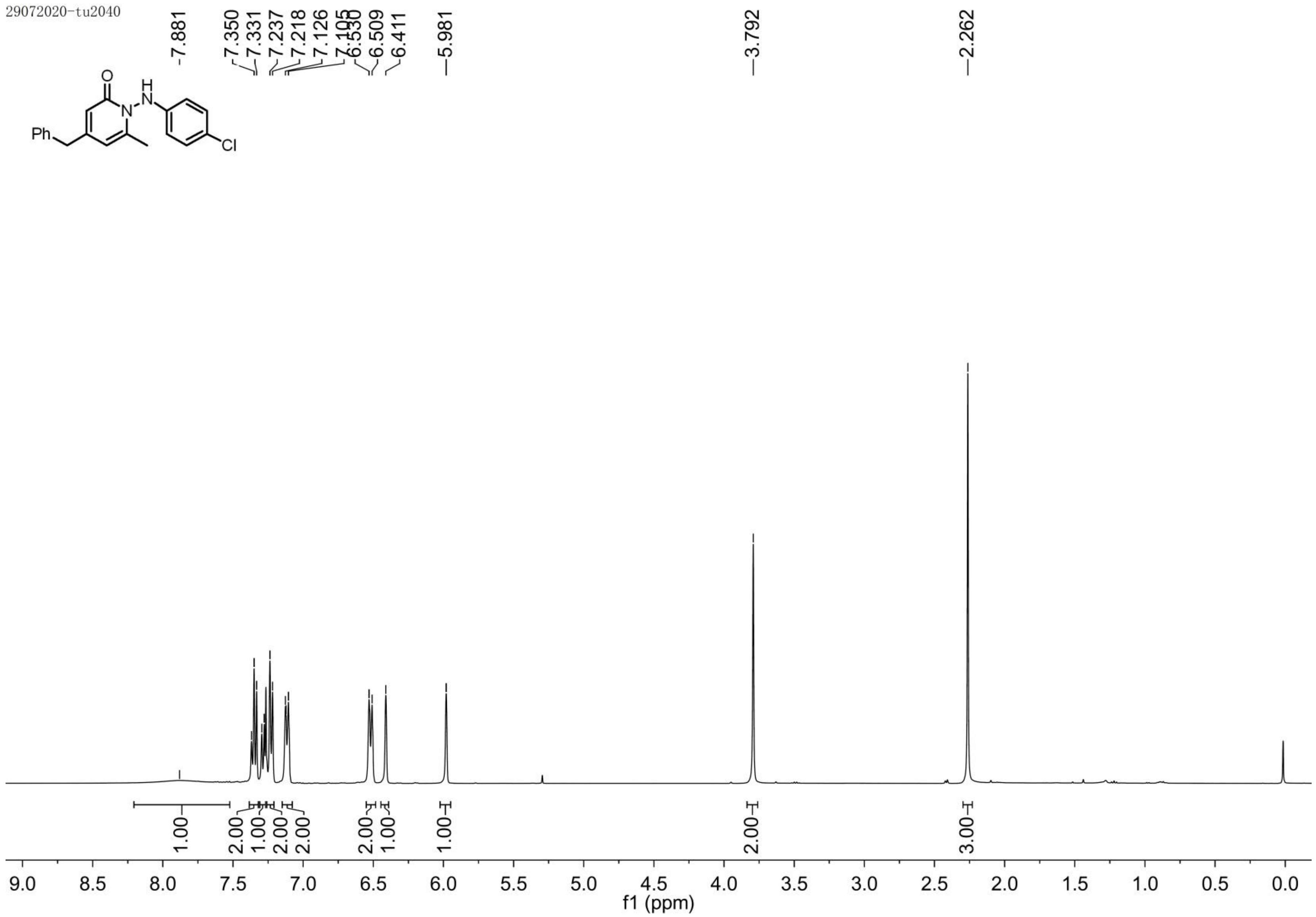

Supplementary Figure 169 ^{1}H NMR Spectrum of Compound **65**

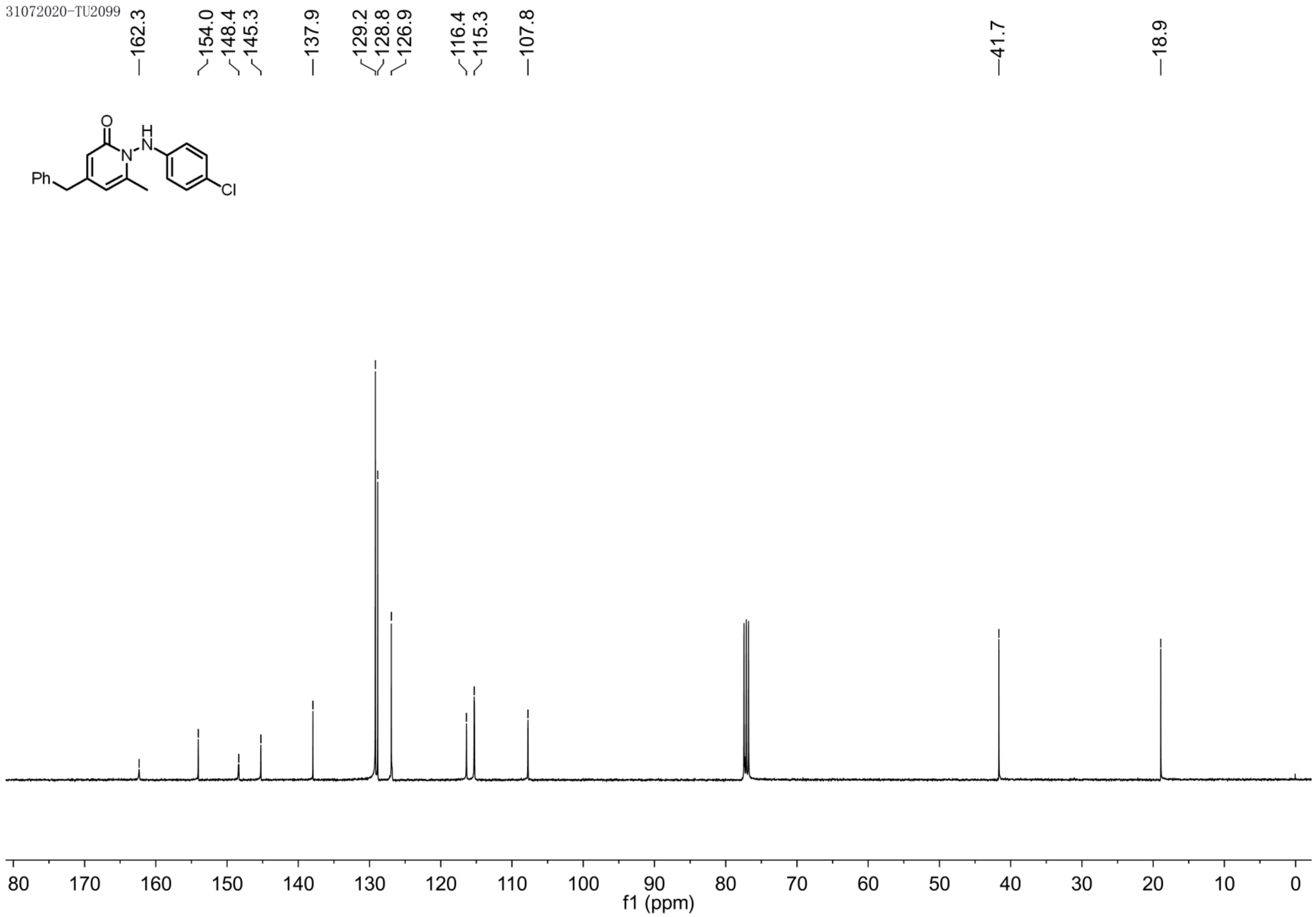

Supplementary Figure 170 ^{13}C NMR Spectrum of Compound **65**

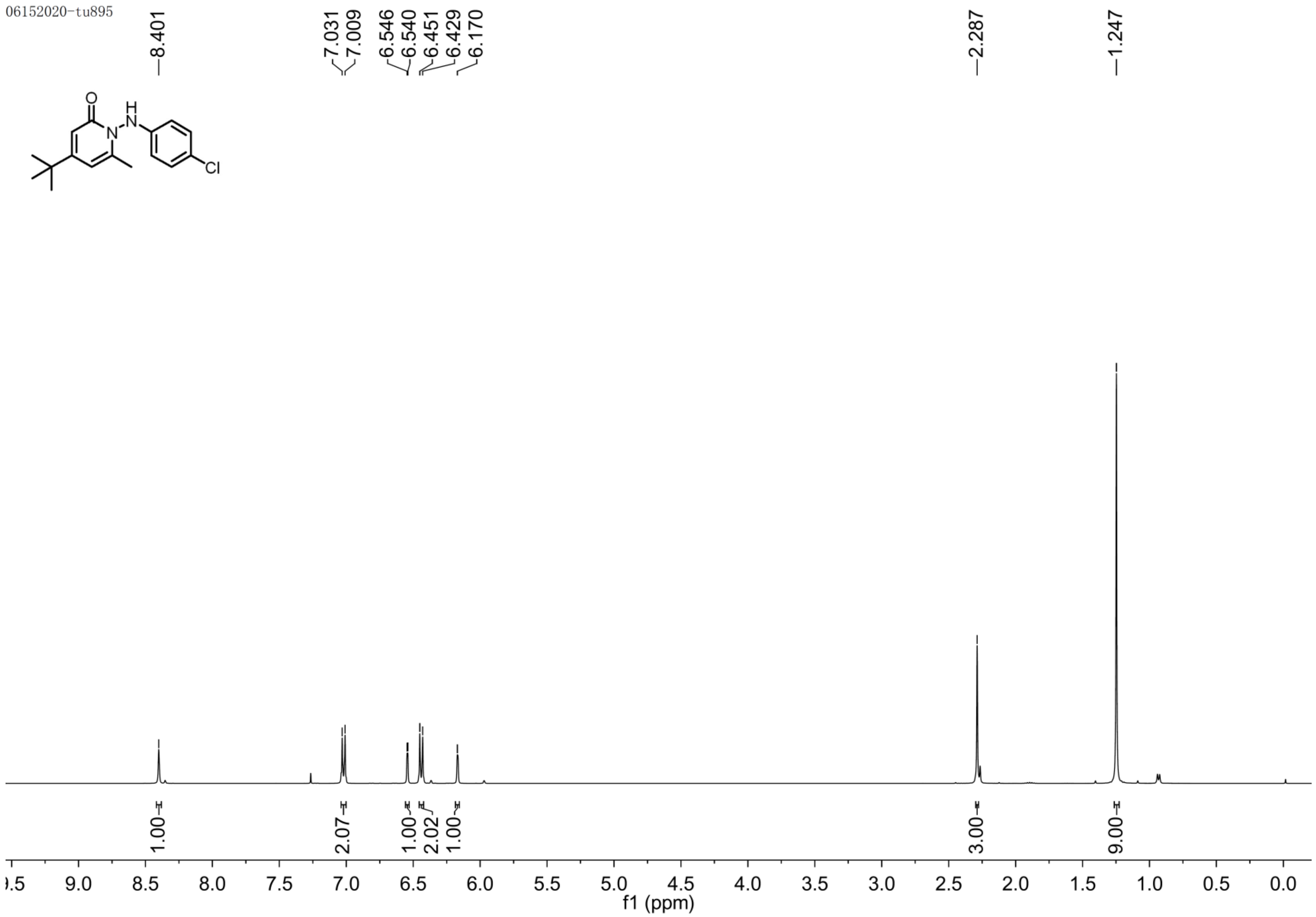

Supplementary Figure 171 ^{1}H NMR Spectrum of Compound **66**

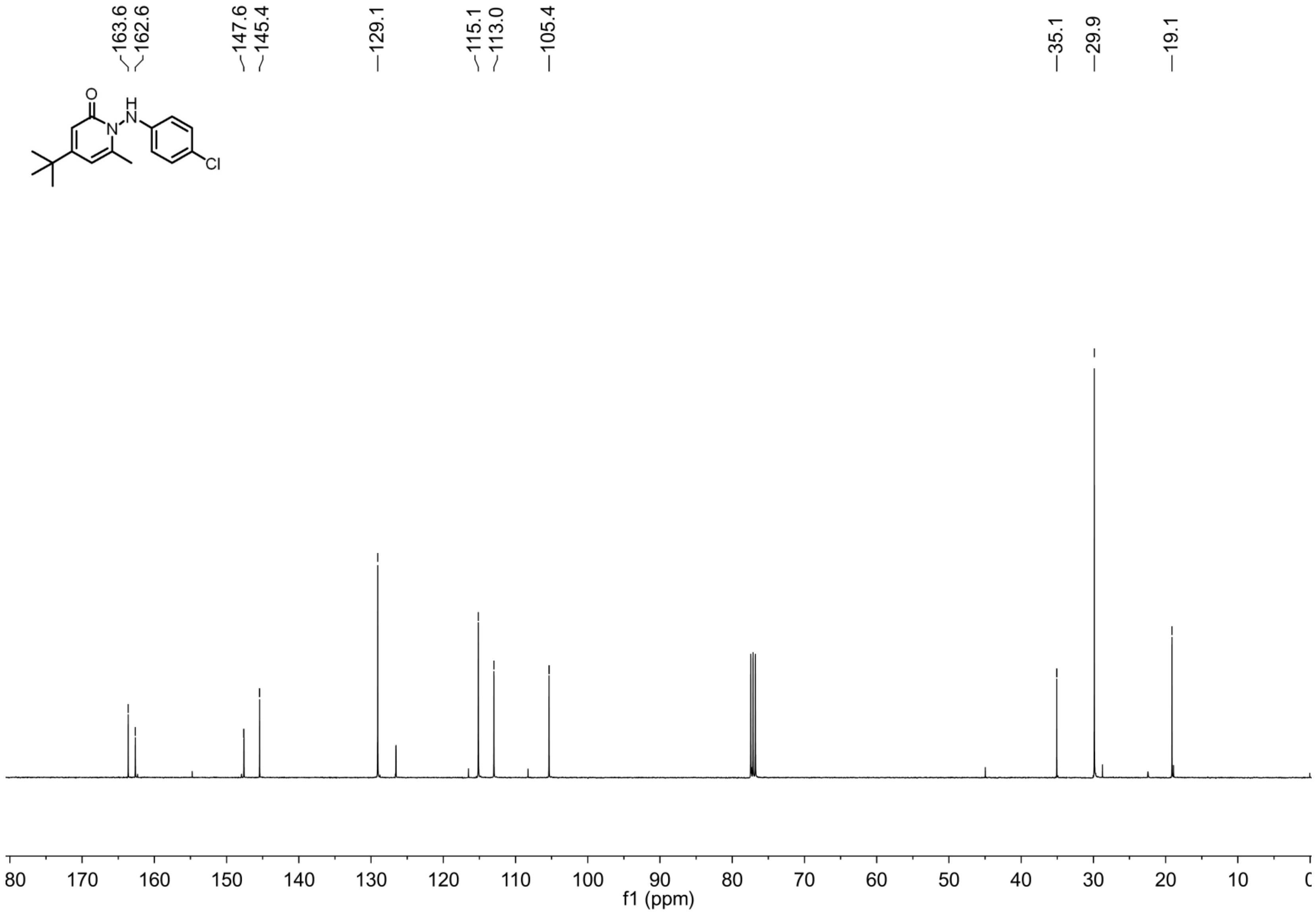

Supplementary Figure 172 ^{13}C NMR Spectrum of Compound **66**

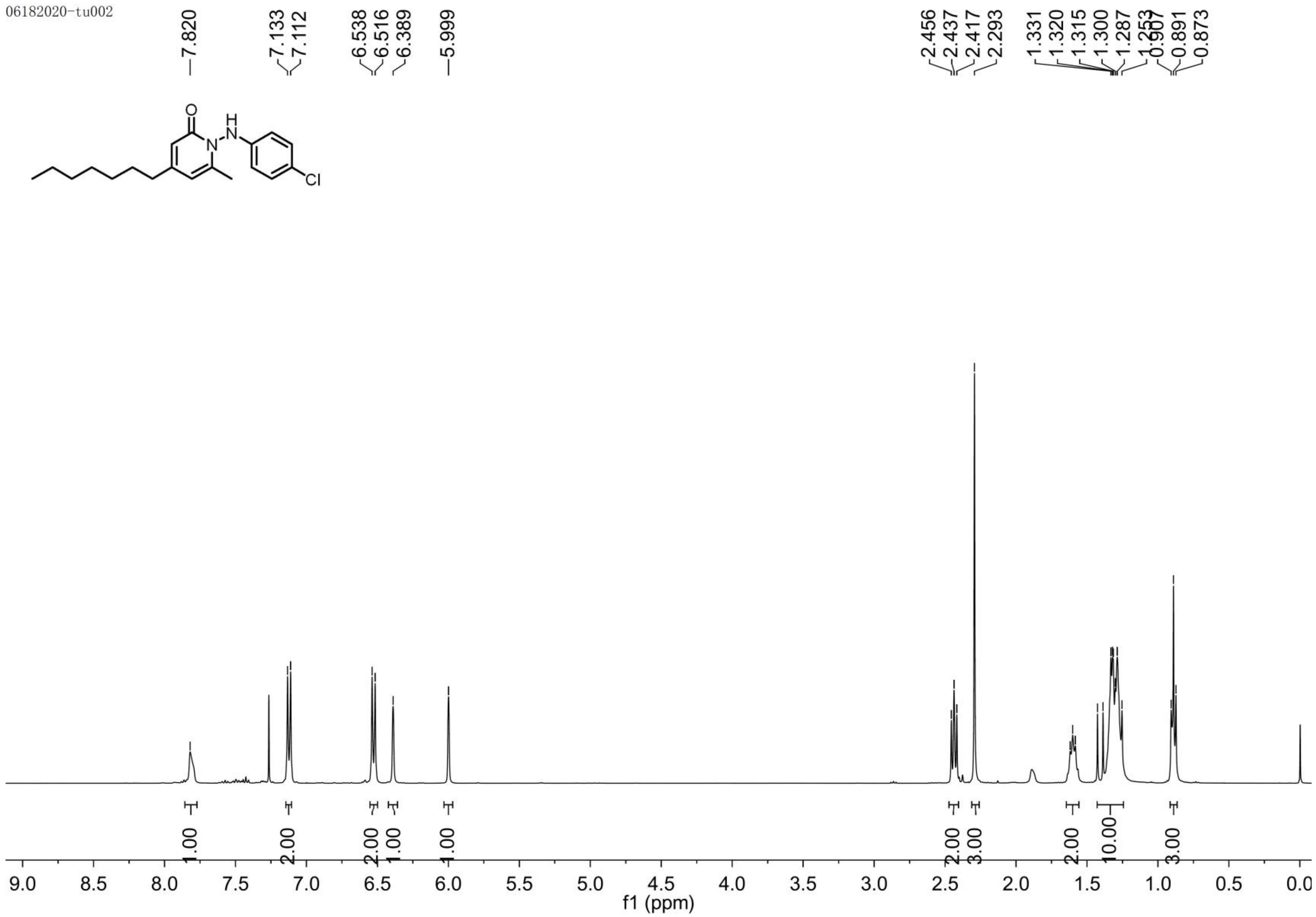

Supplementary Figure 173 ^{1}H NMR Spectrum of Compound **67**

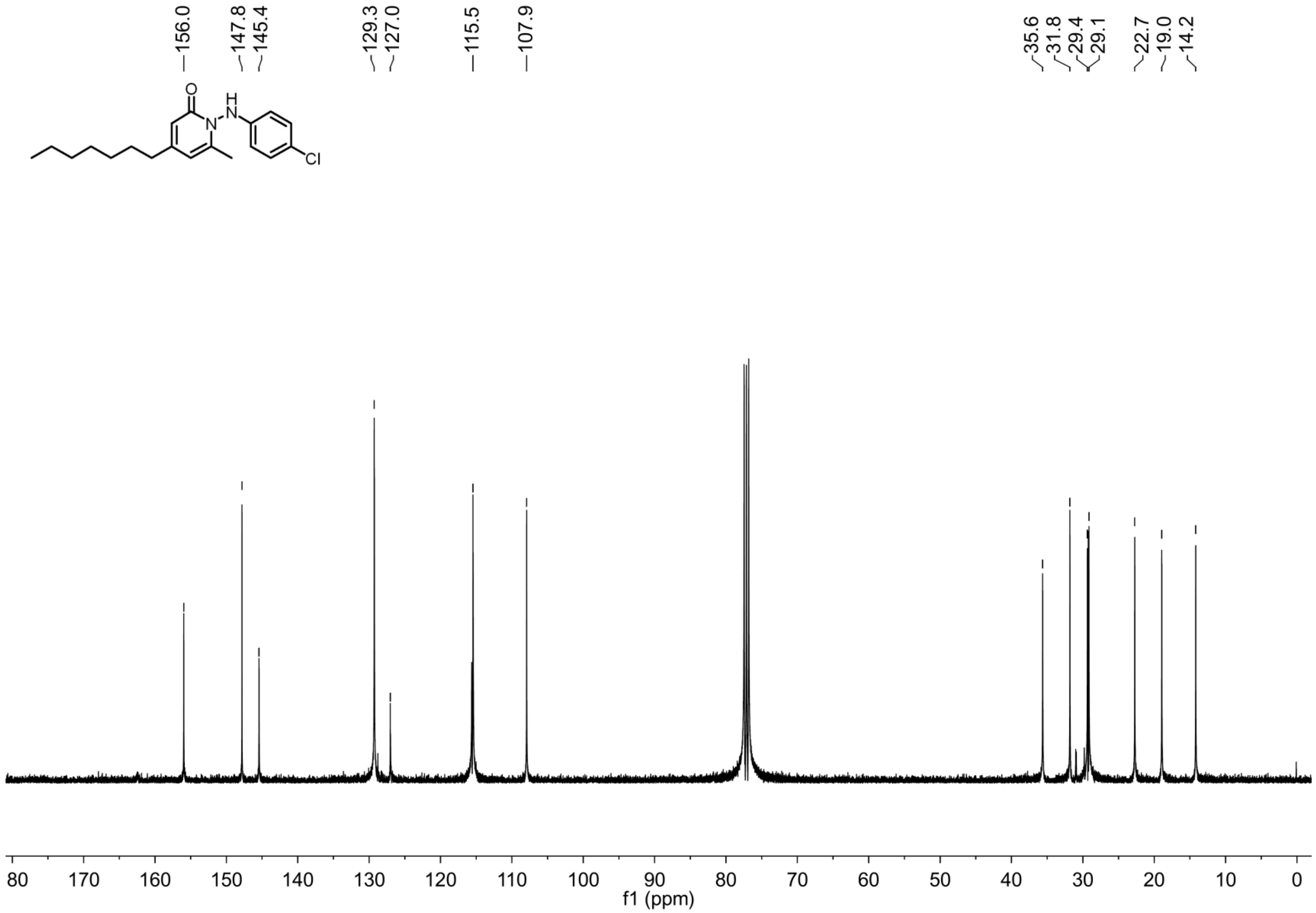

Supplementary Figure 174 ^{13}C NMR Spectrum of Compound **67**

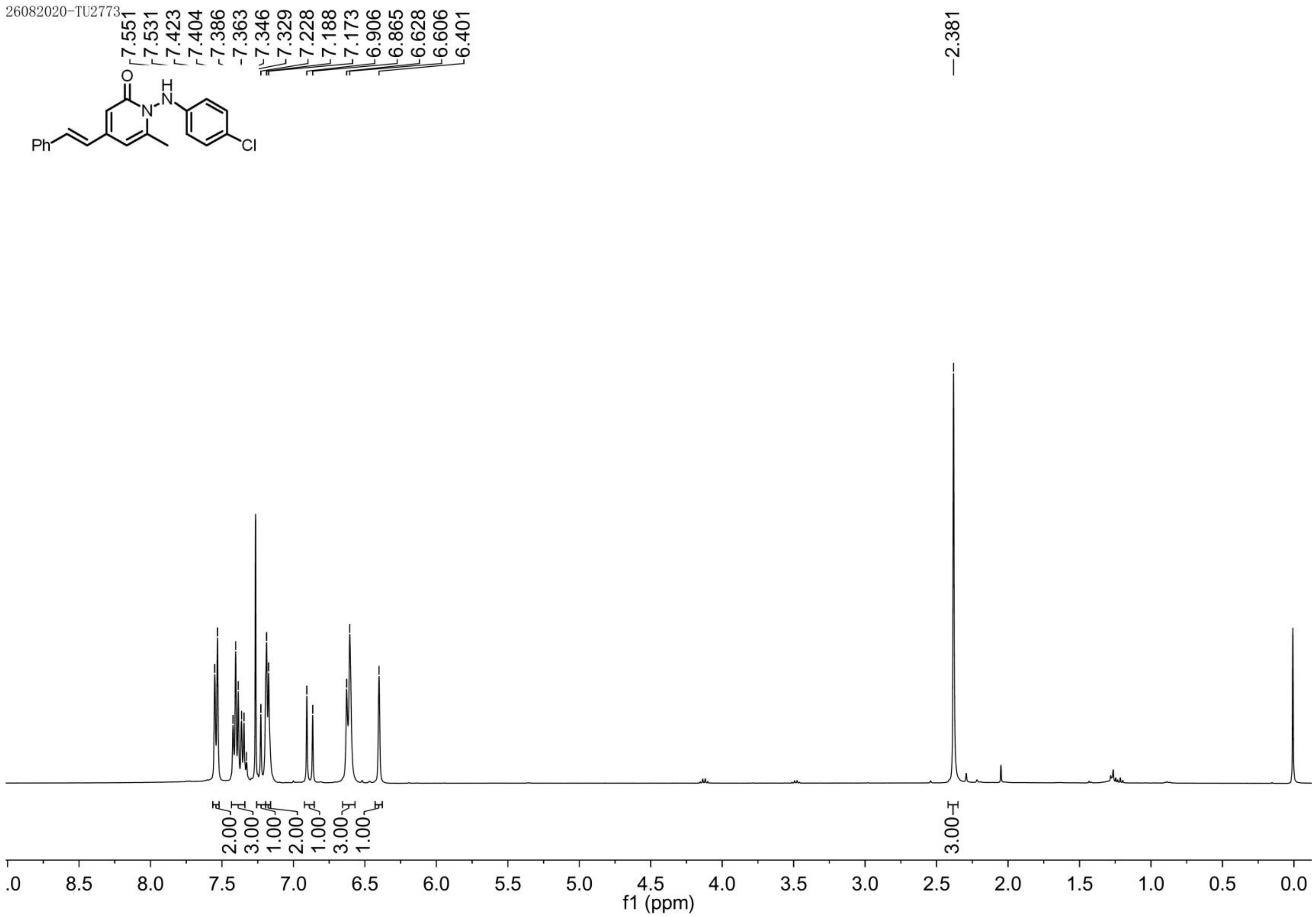

Supplementary Figure 175 ^{1}H NMR Spectrum of Compound **68**

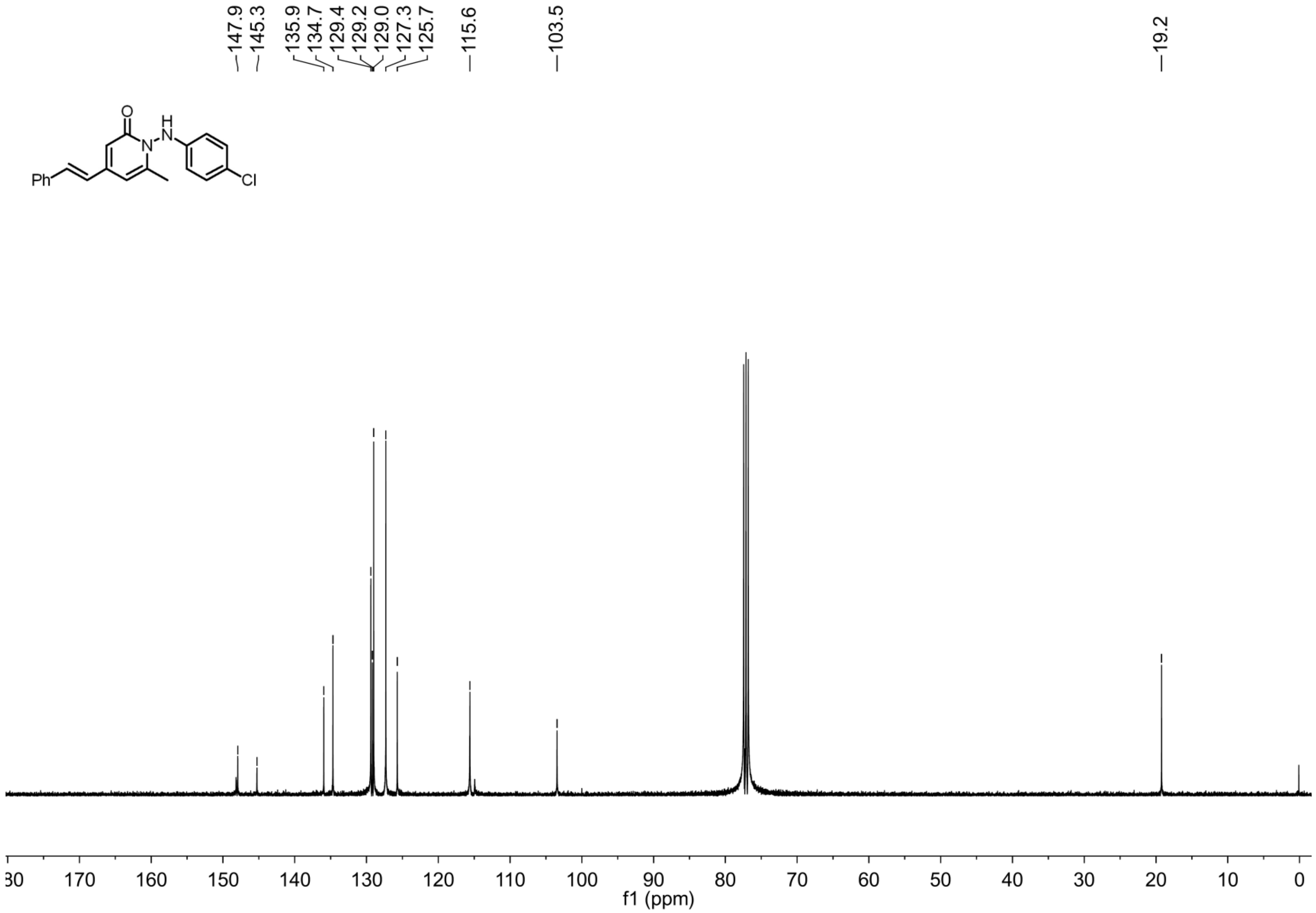

Supplementary Figure 176 ^{13}C NMR Spectrum of Compound **68**

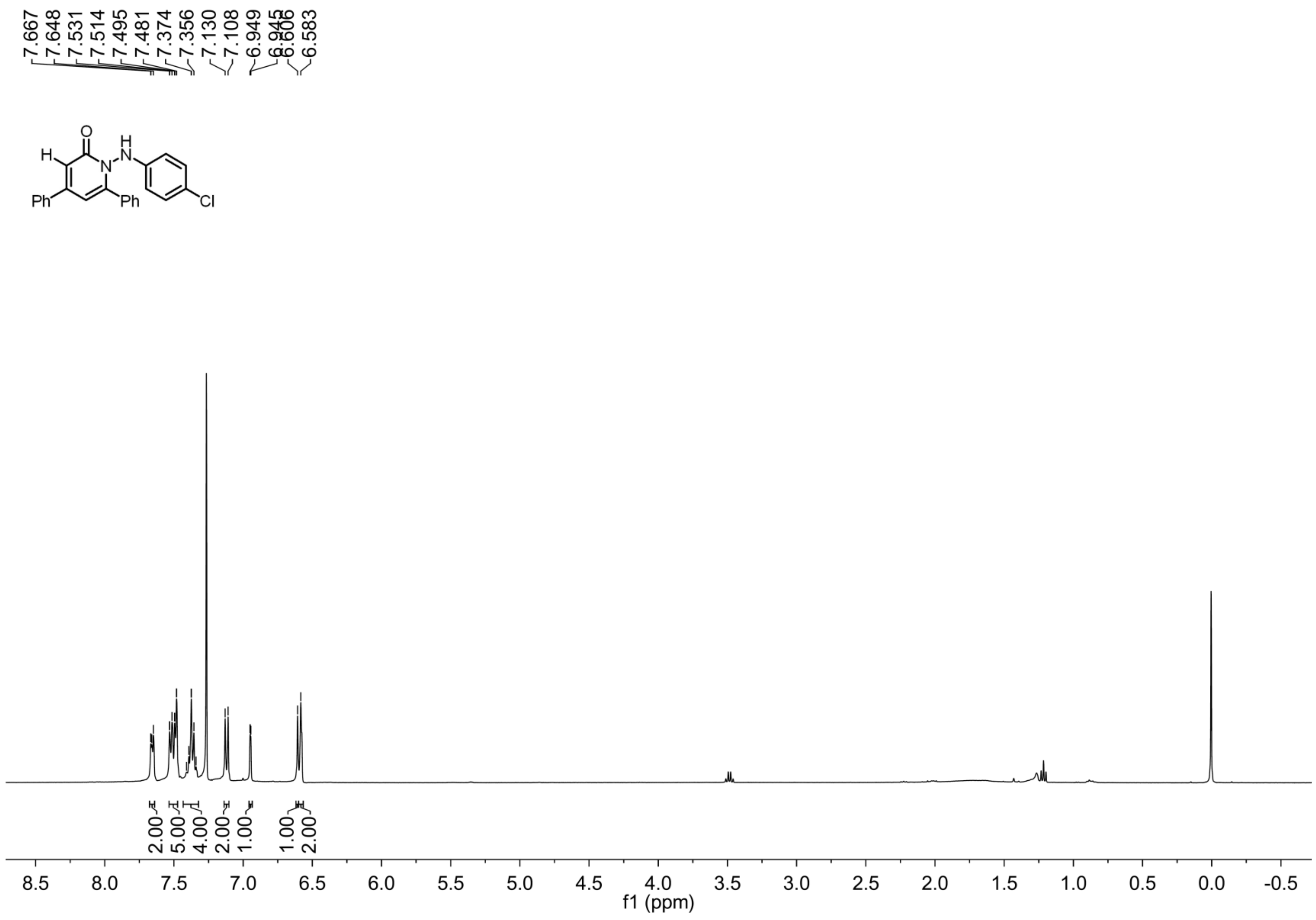

Supplementary Figure 177 ^{1}H NMR Spectrum of Compound **69**

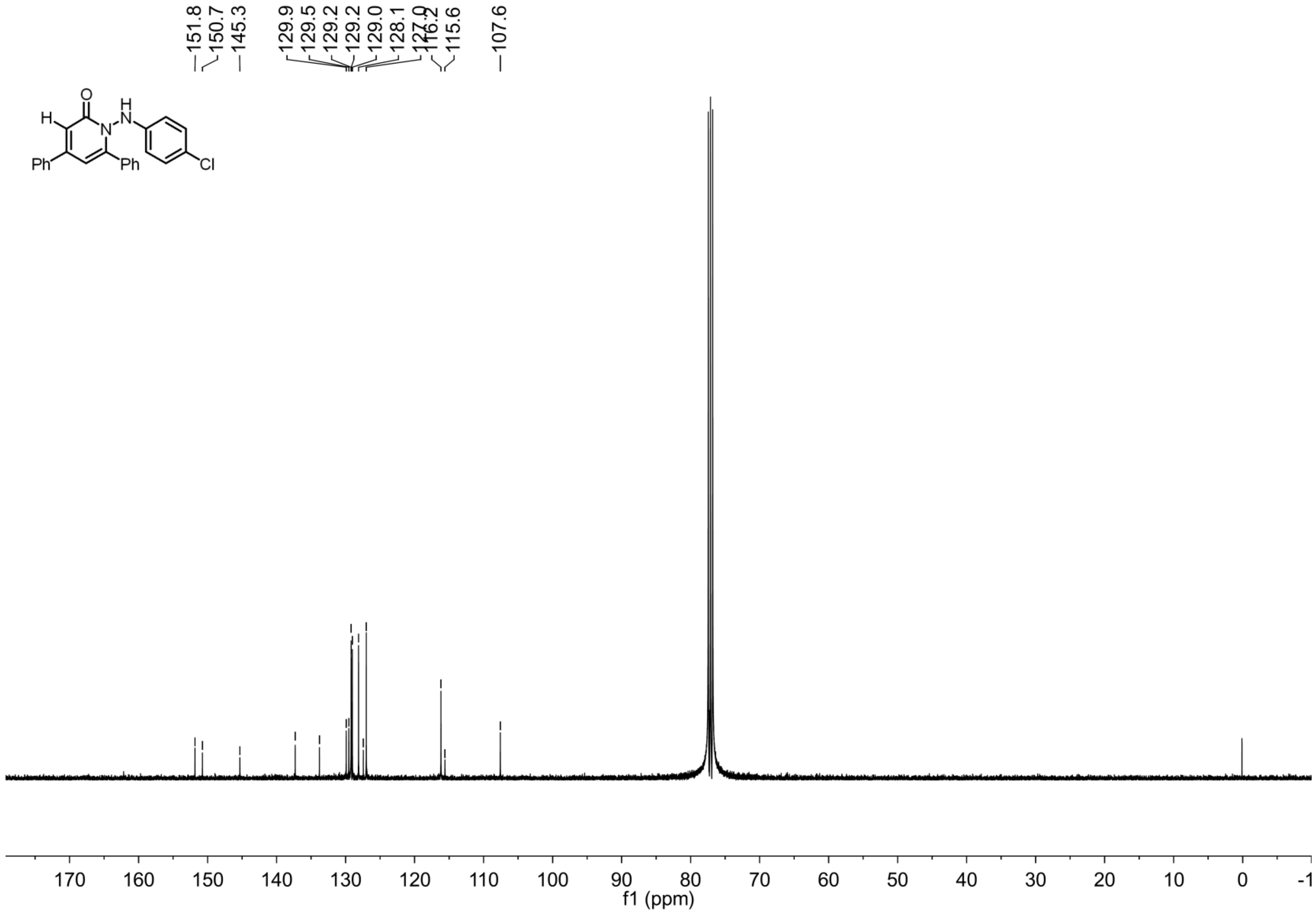

Supplementary Figure 178 ^{13}C NMR Spectrum of Compound **69**

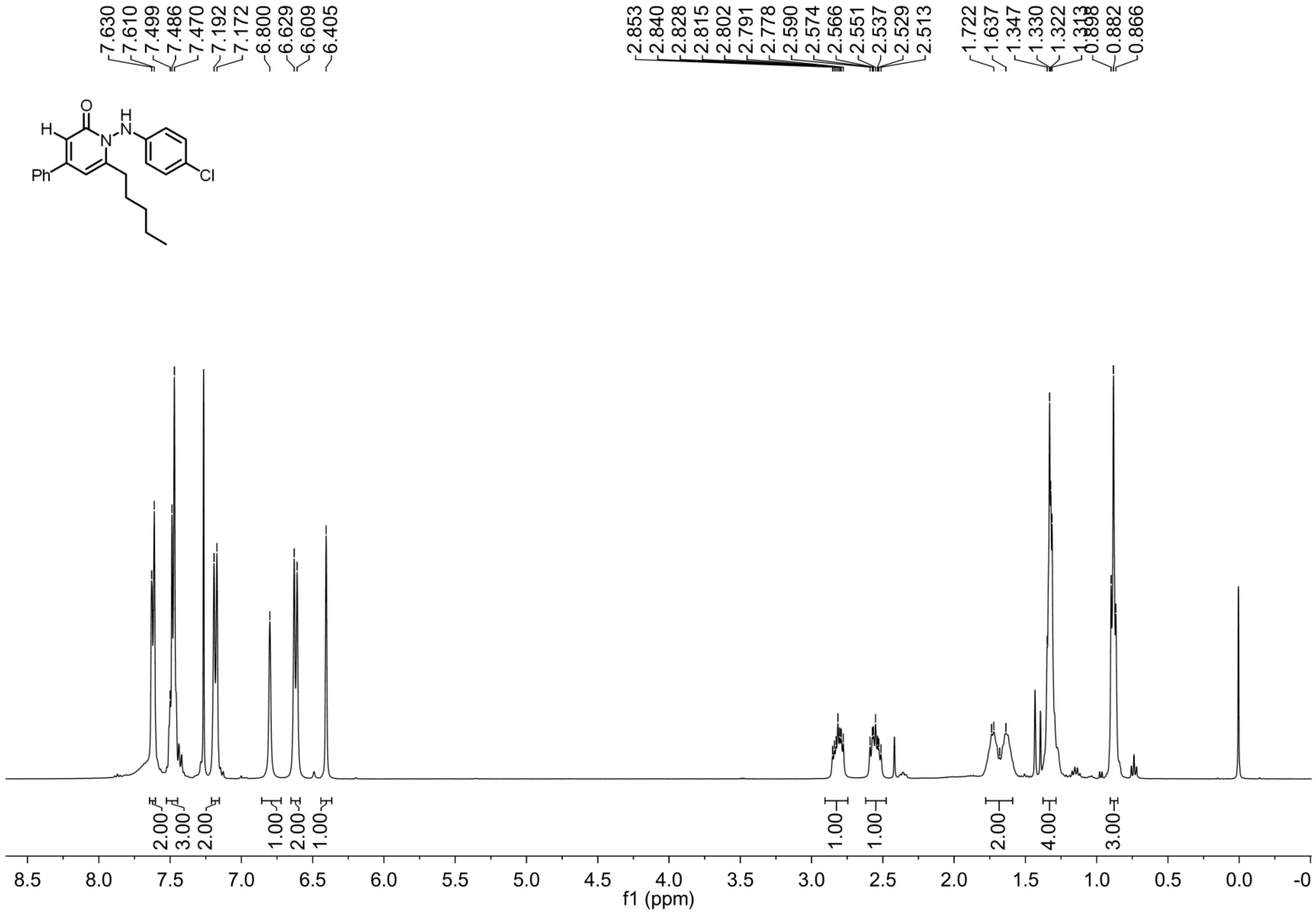

Supplementary Figure 179 ^{1}H NMR Spectrum of Compound **70**

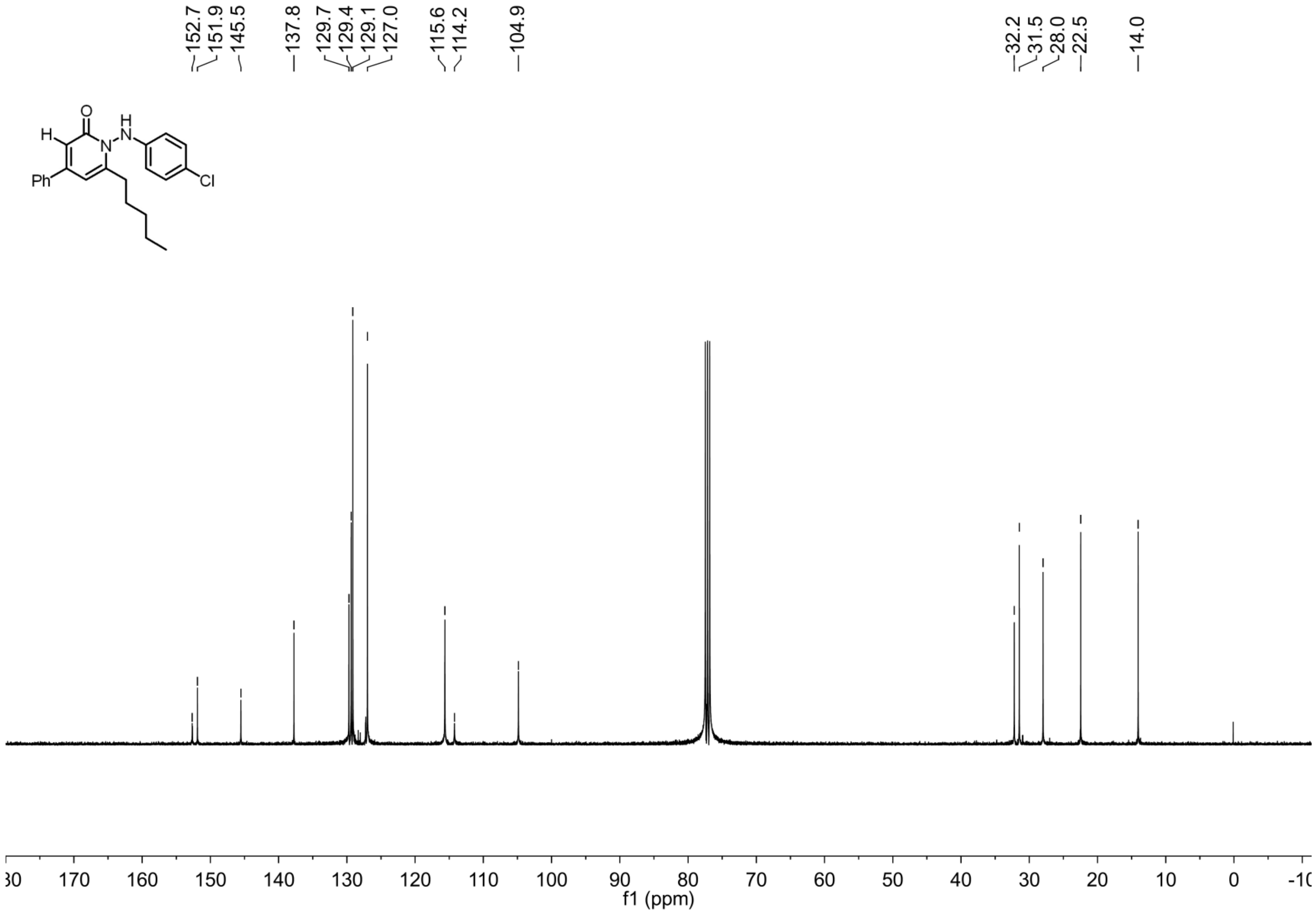

Supplementary Figure 180 ^{13}C NMR Spectrum of Compound **70**

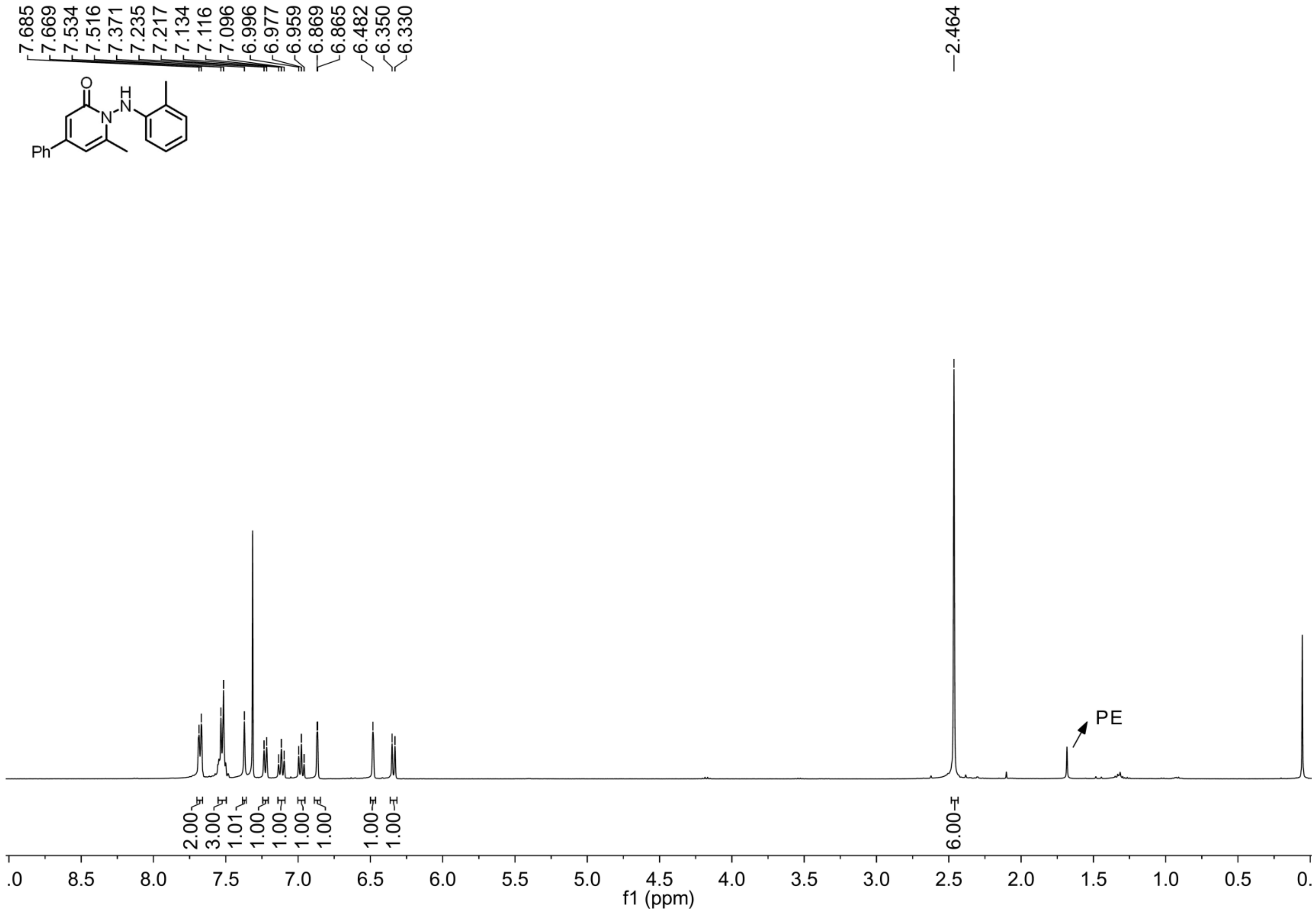

Supplementary Figure 181 ^{1}H NMR Spectrum of Compound **71**

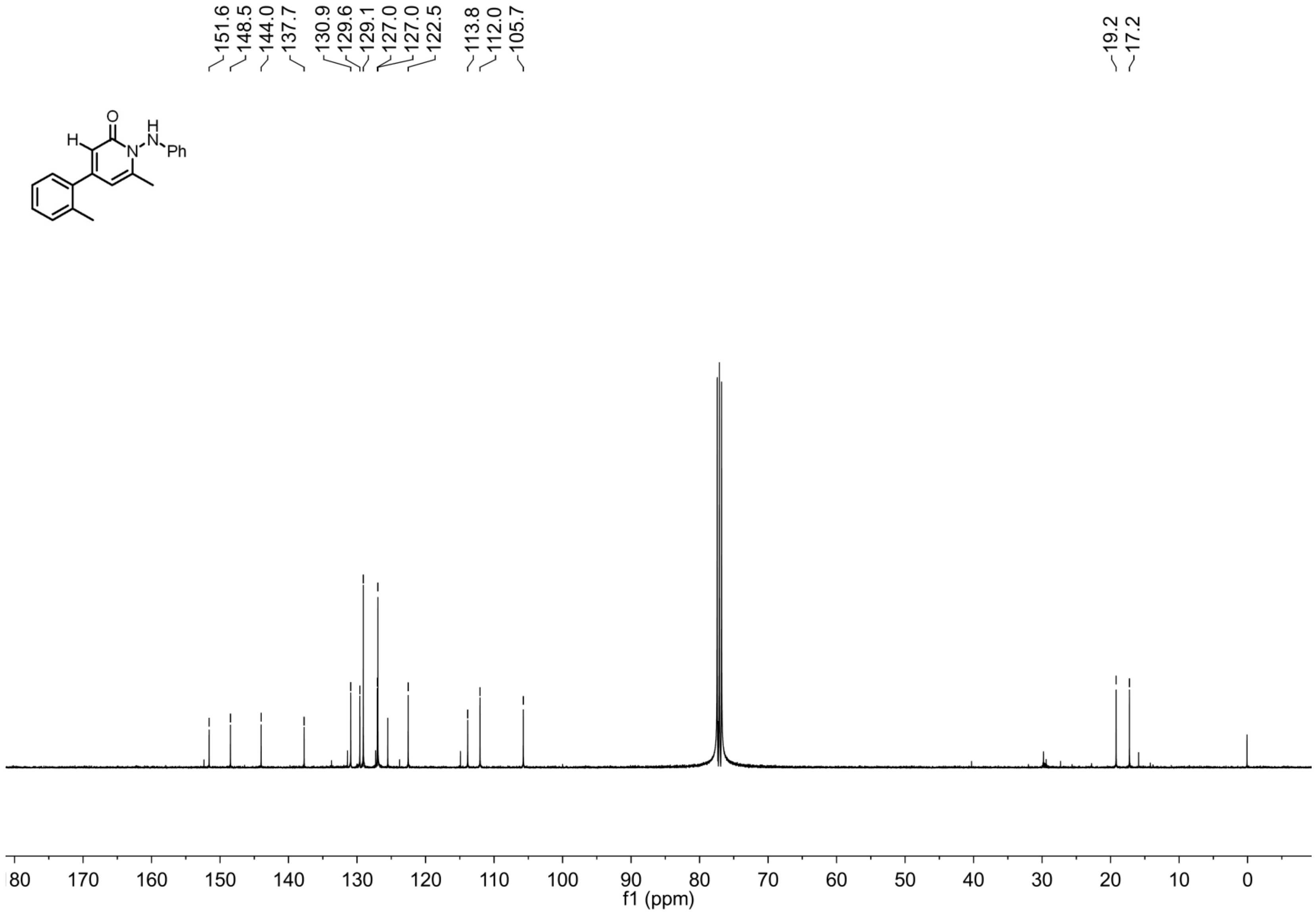

Supplementary Figure 182 ^{13}C NMR Spectrum of Compound **71**

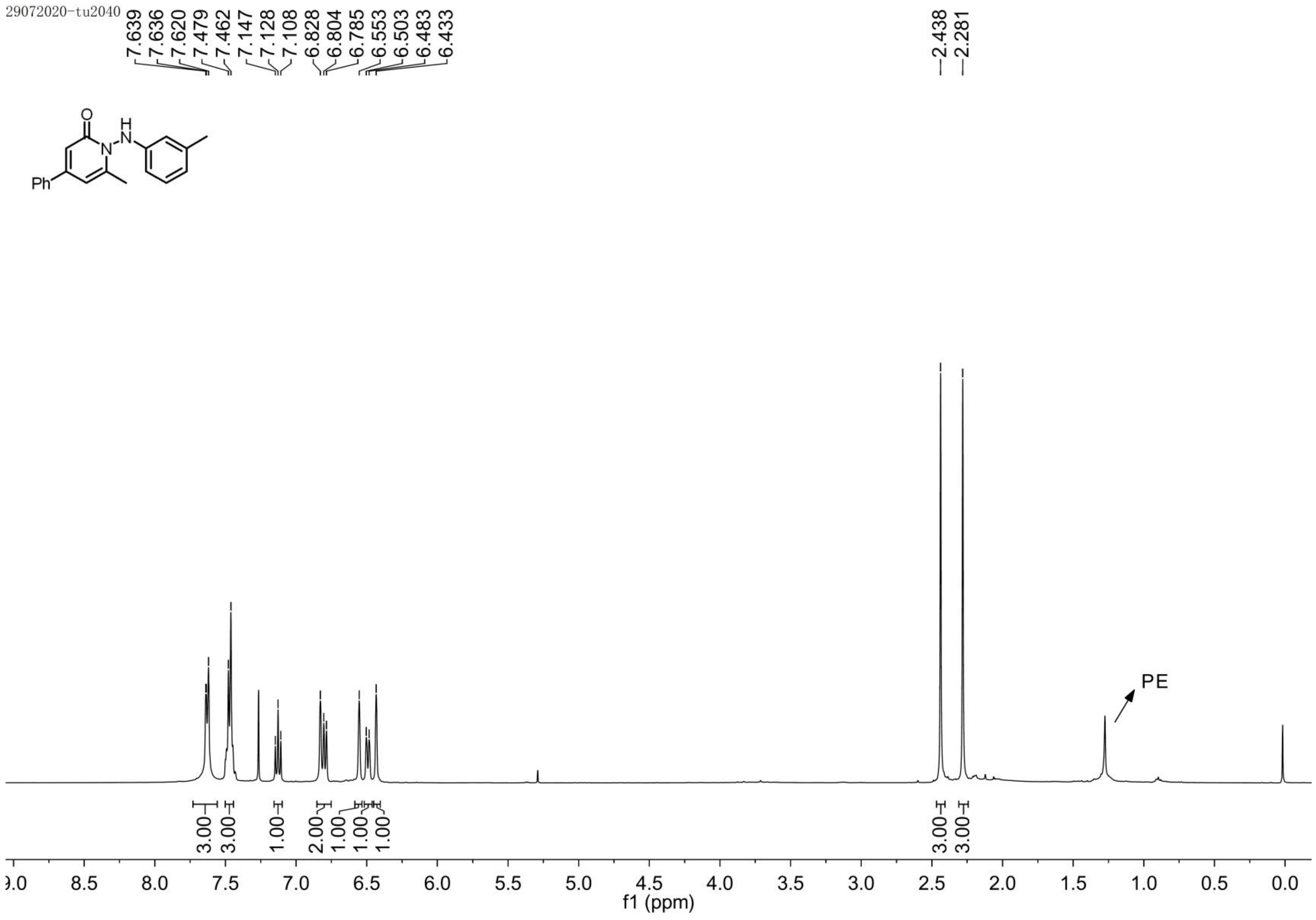

Supplementary Figure 183 ^{1}H NMR Spectrum of Compound **72**

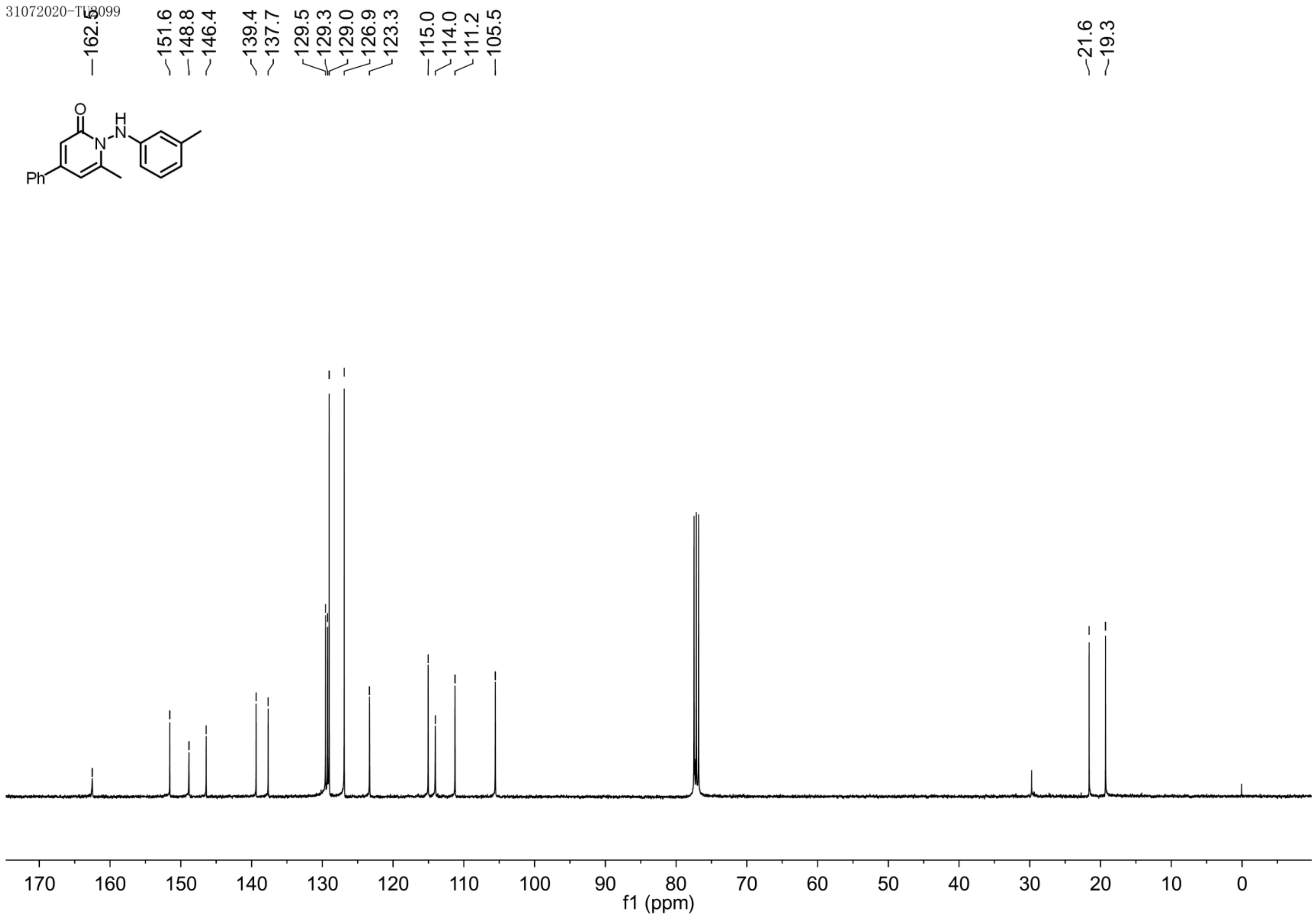

Supplementary Figure 184 ^{13}C NMR Spectrum of Compound **72**

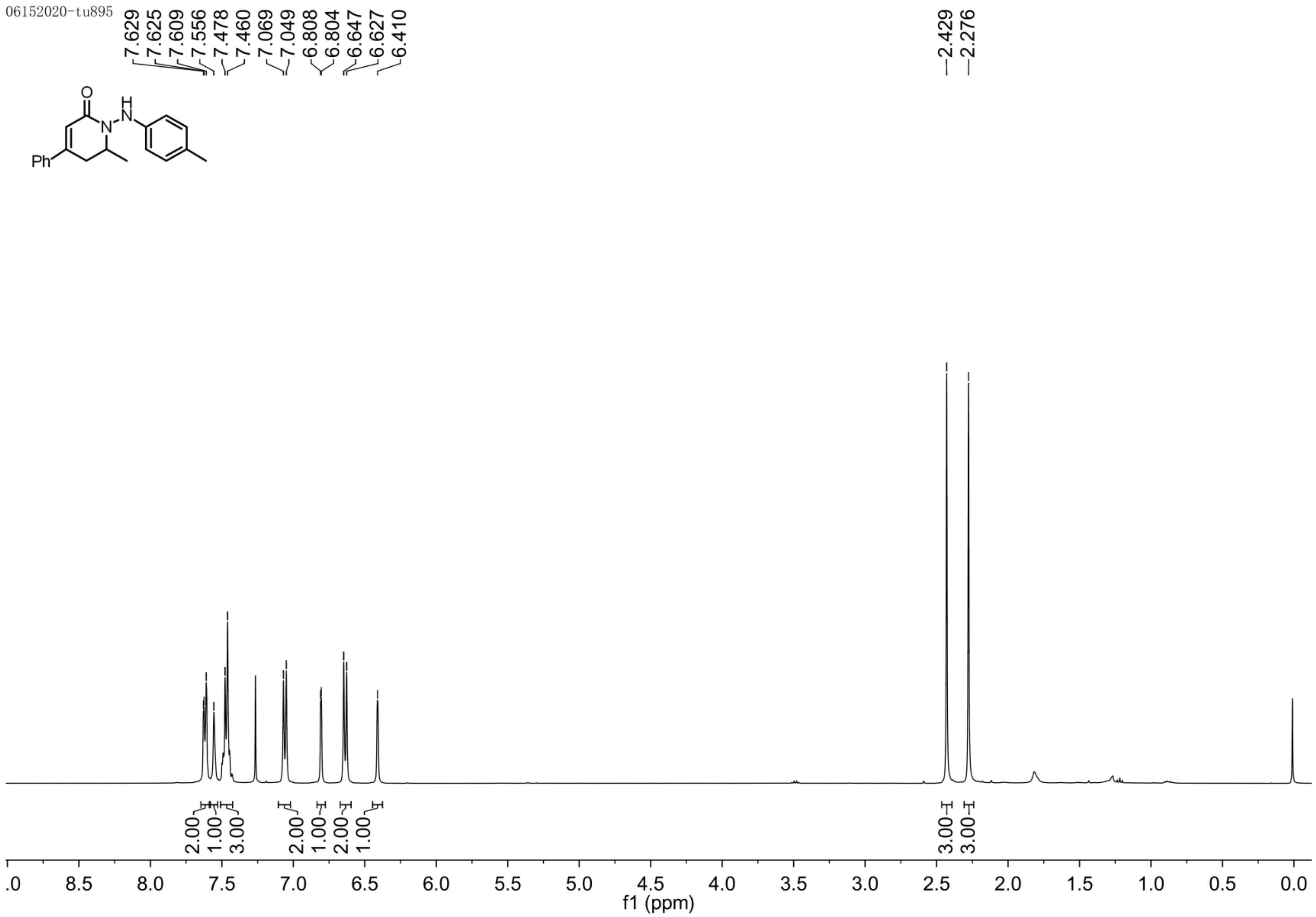

Supplementary Figure 185 ^{1}H NMR Spectrum of Compound **73**

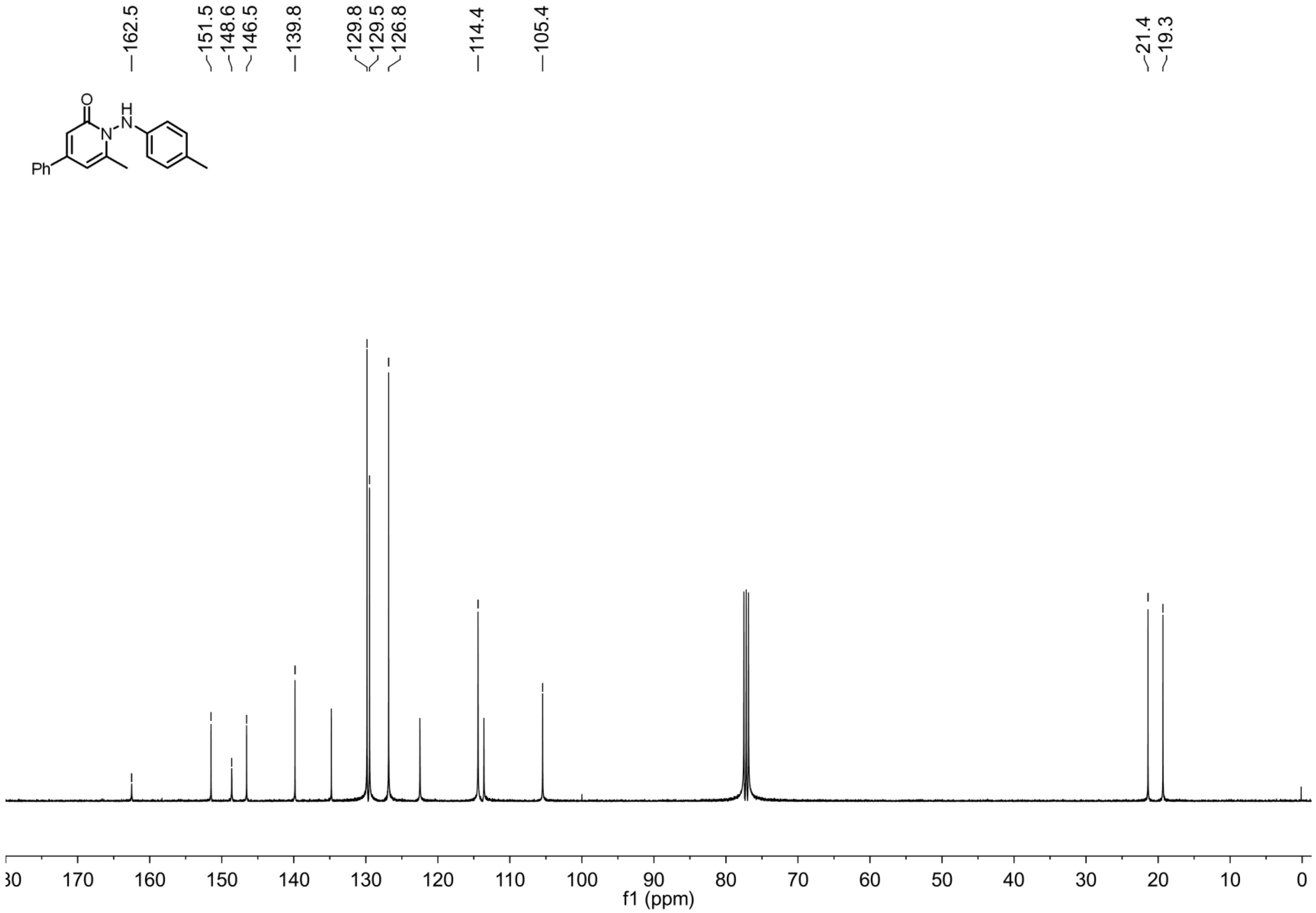

Supplementary Figure 186 ^{13}C NMR Spectrum of Compound **73**

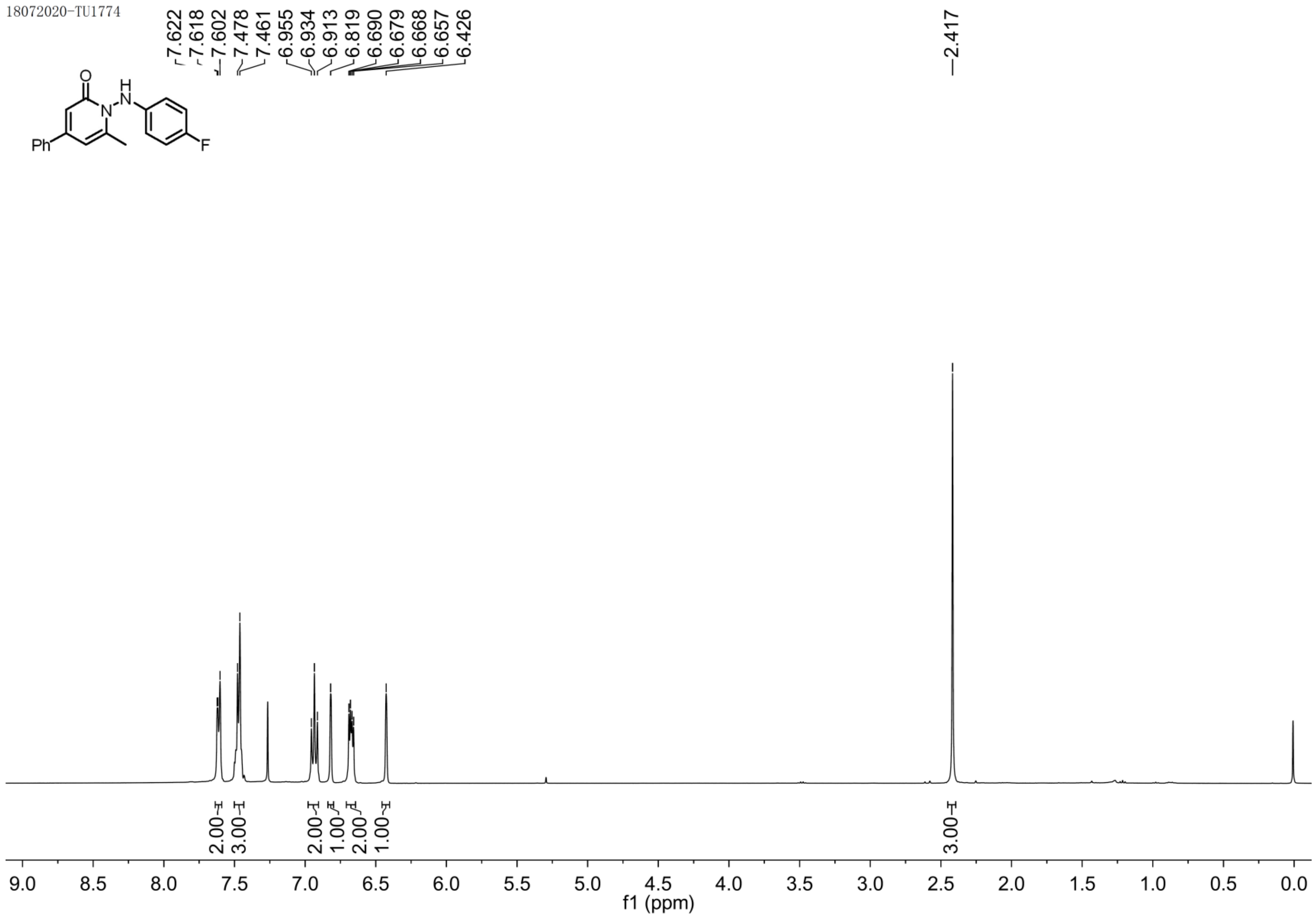

Supplementary Figure 187 ^{1}H NMR Spectrum of Compound **74**

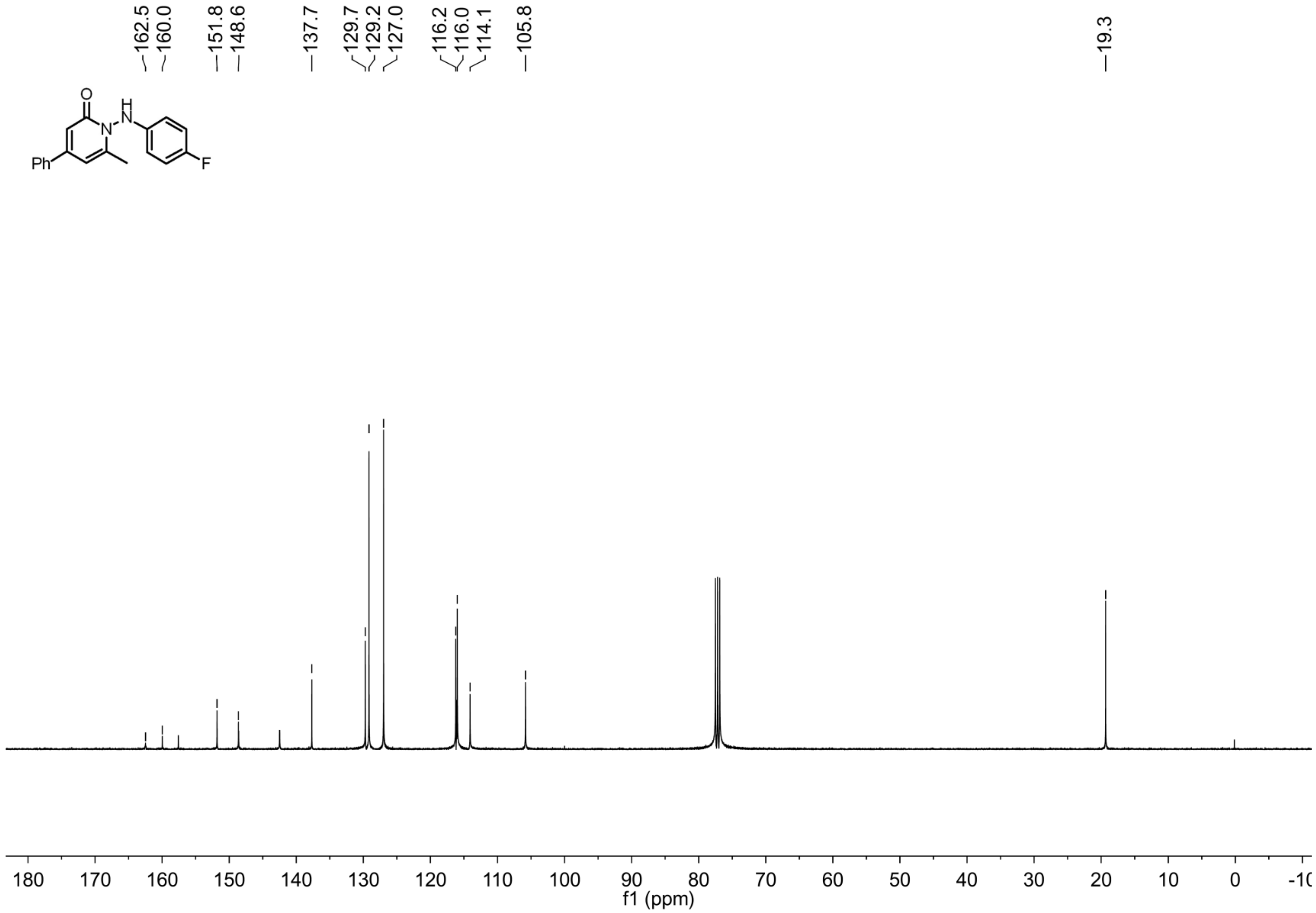

Supplementary Figure 188 ^{13}C NMR Spectrum of Compound **74**

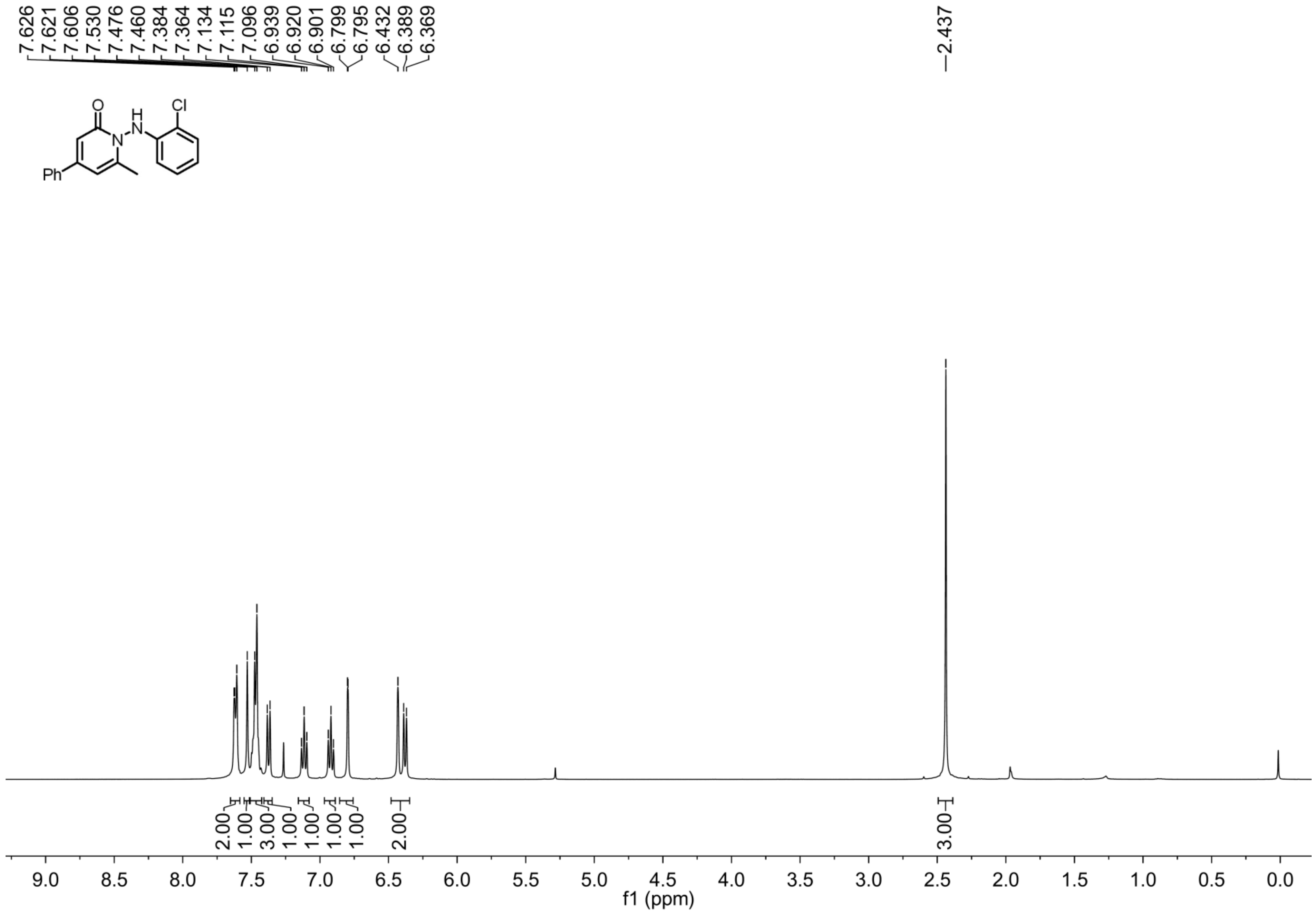

Supplementary Figure 189 ^{1}H NMR Spectrum of Compound **75**

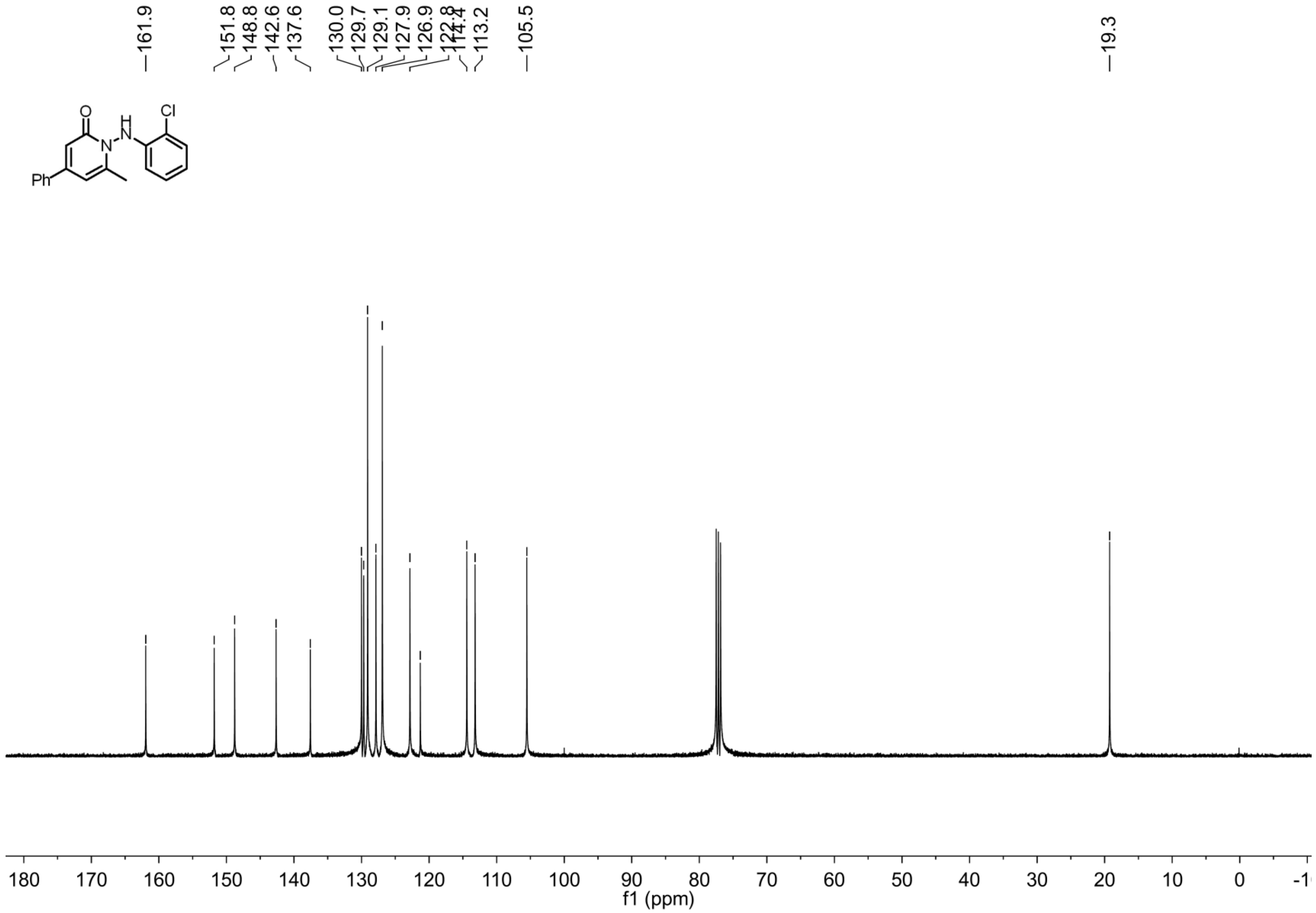

Supplementary Figure 190 ^{13}C NMR Spectrum of Compound **75**

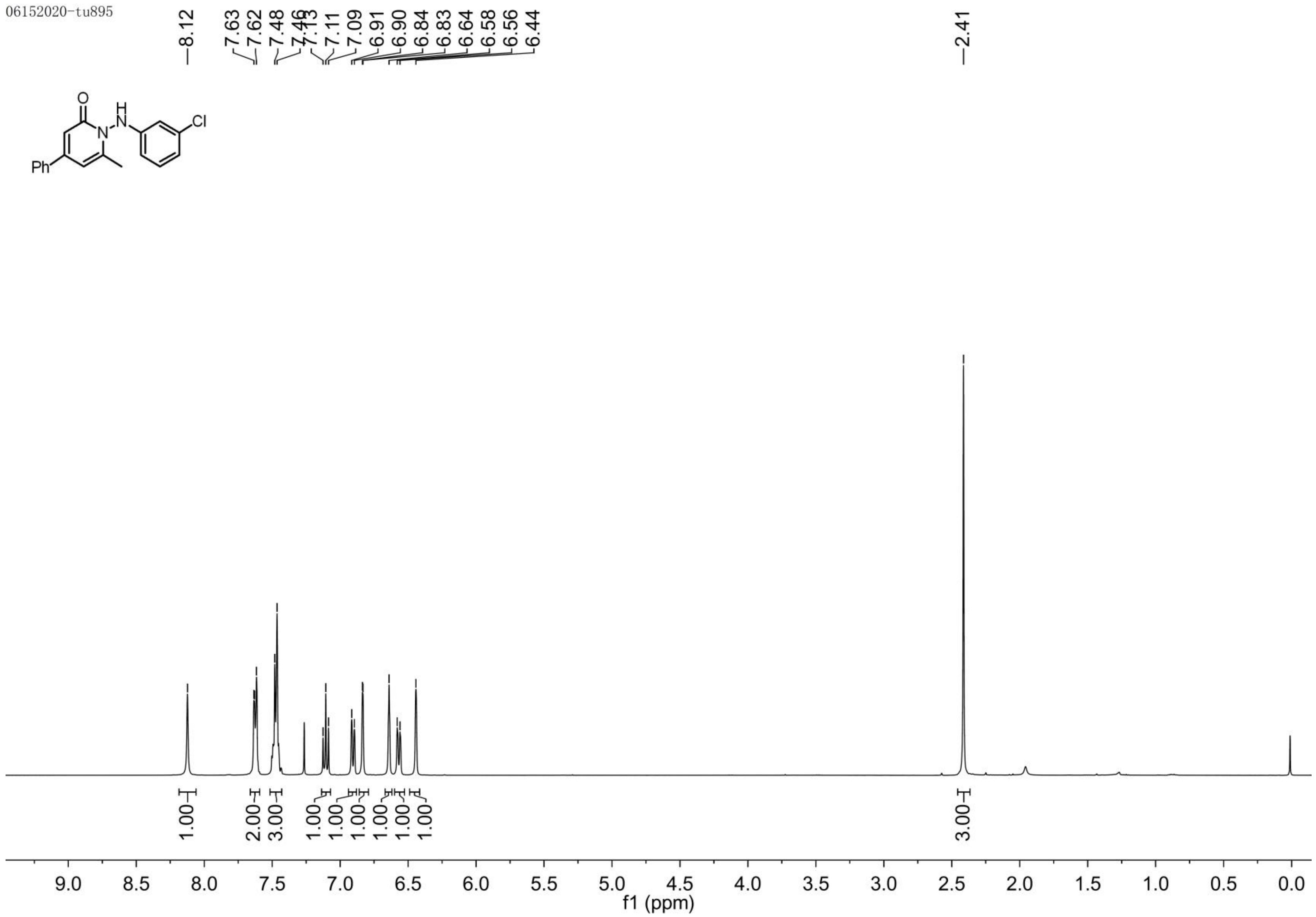

Supplementary Figure 191 ^{1}H NMR Spectrum of Compound **76**

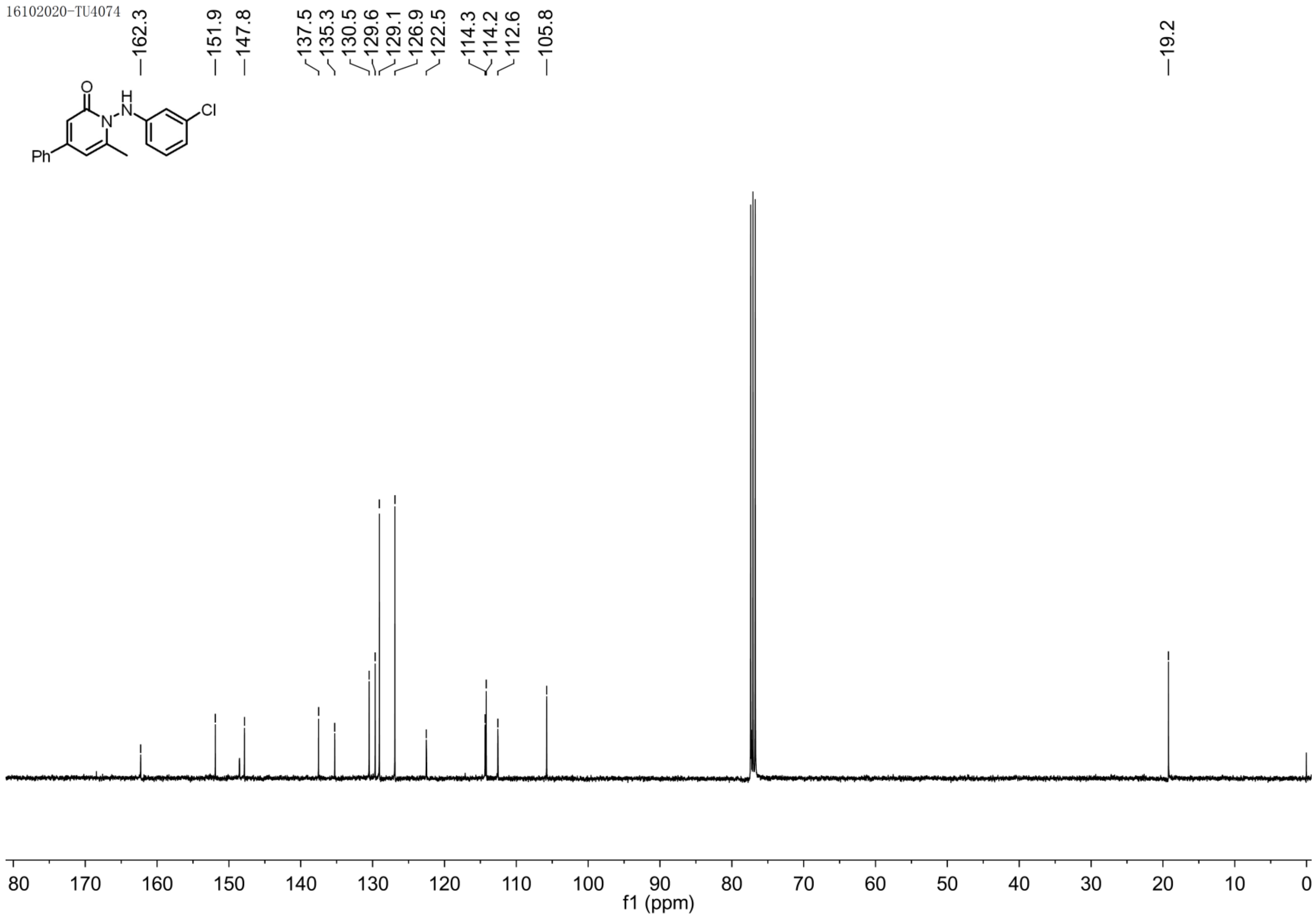

Supplementary Figure 192 ^{13}C NMR Spectrum of Compound **76**

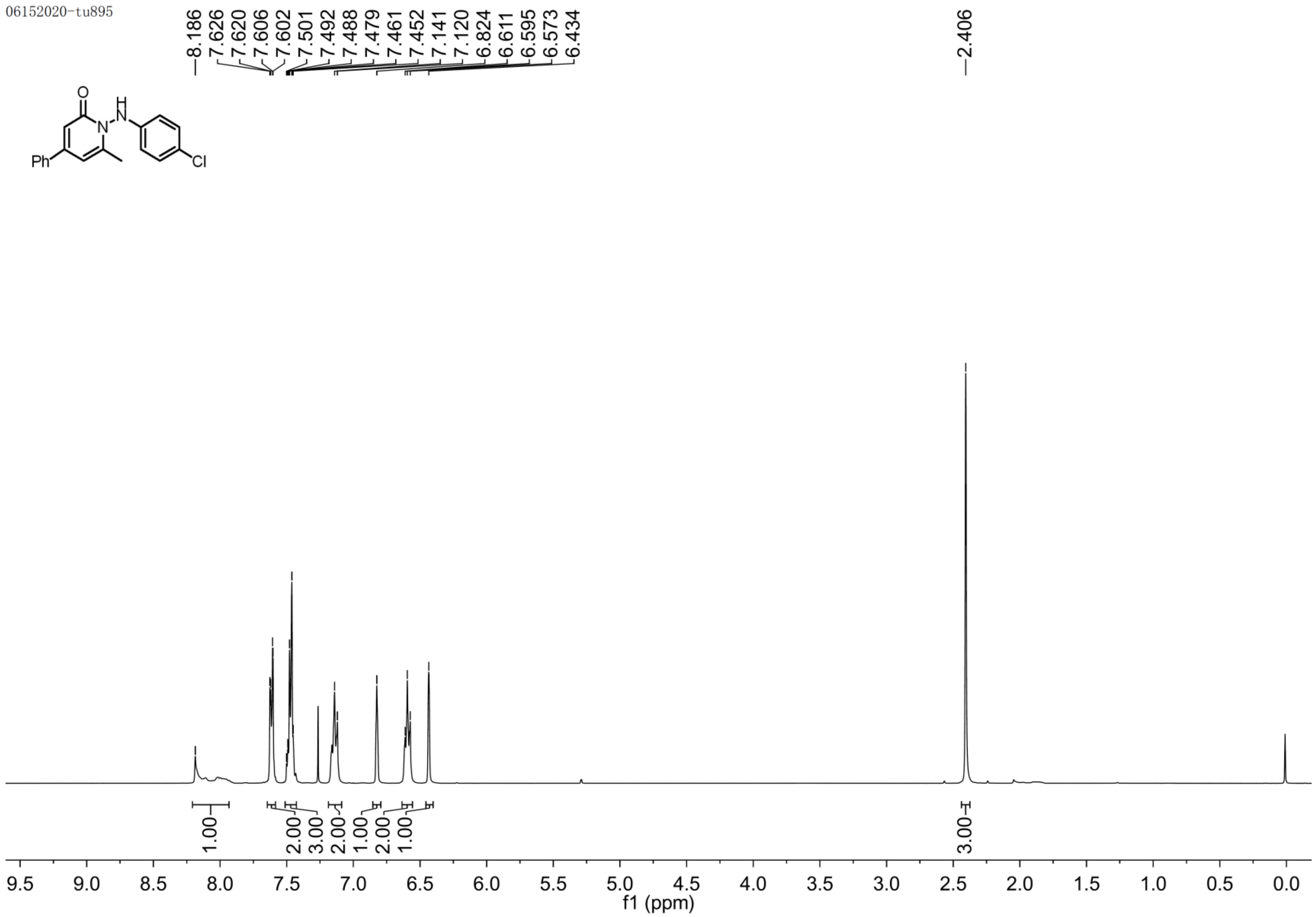

Supplementary Figure 193 ^{1}H NMR Spectrum of Compound **77**

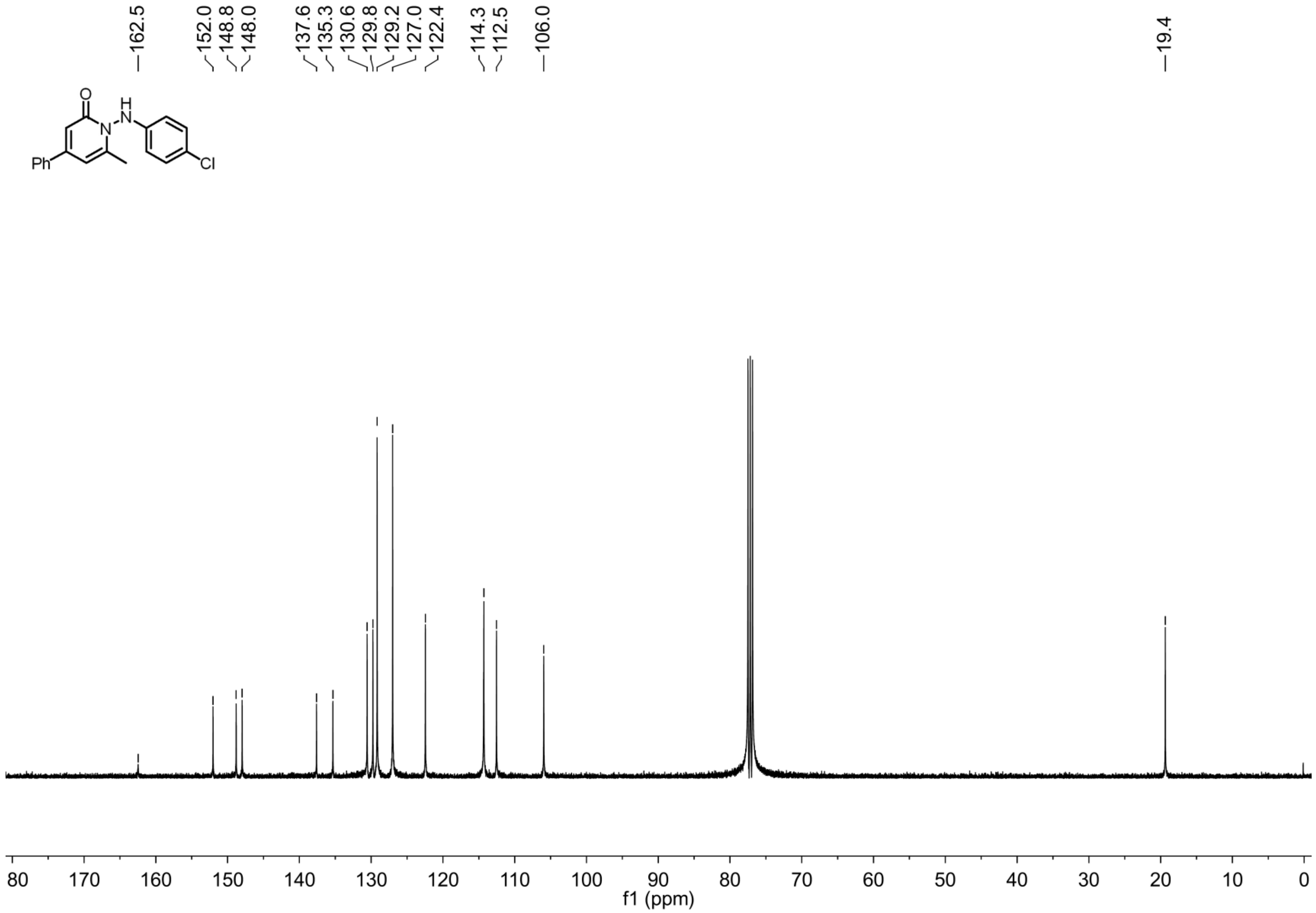

Supplementary Figure 194 ^{13}C NMR Spectrum of Compound **77**

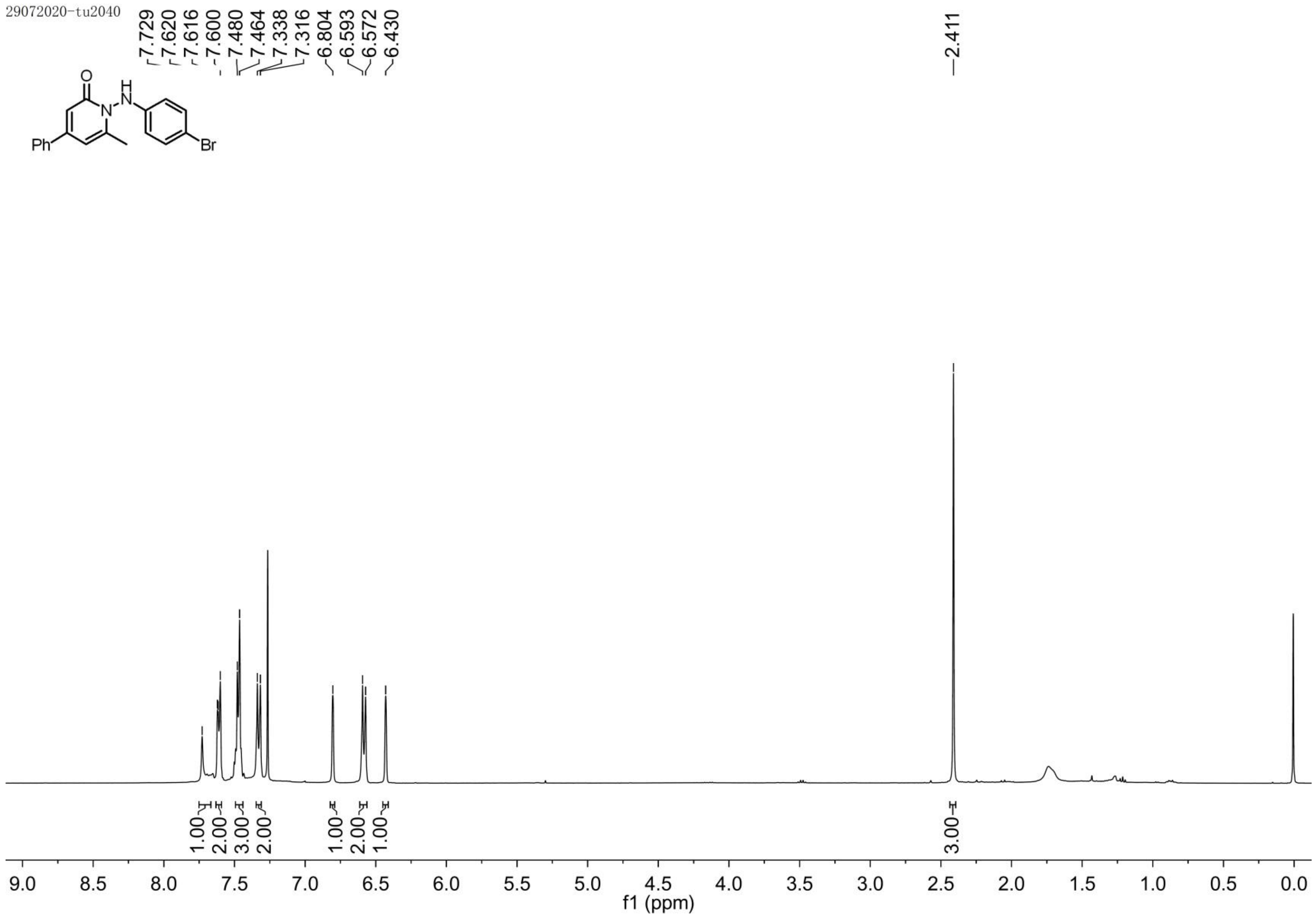

Supplementary Figure 195 ^{1}H NMR Spectrum of Compound **78**

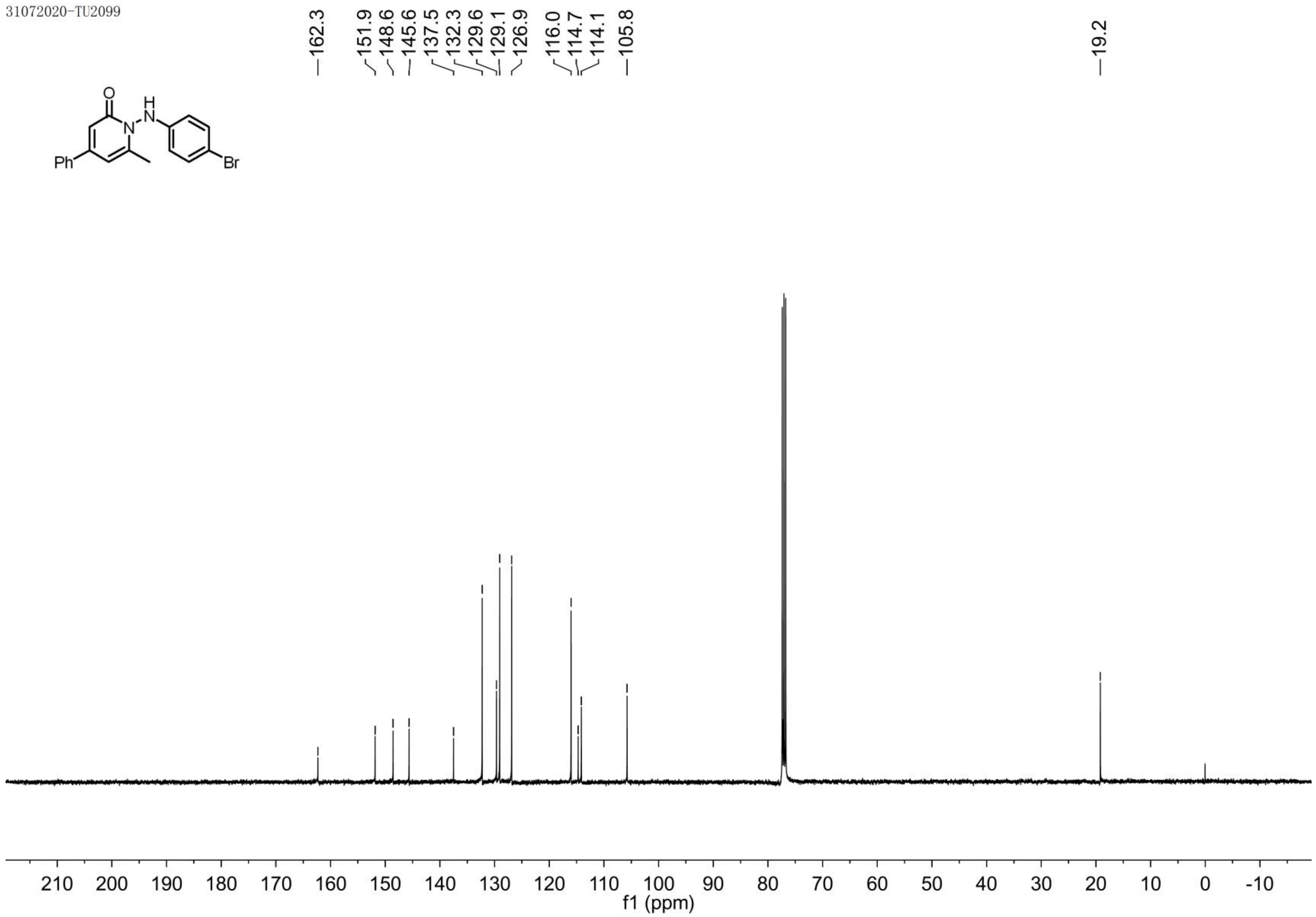

Supplementary Figure 196 ^{13}C NMR Spectrum of Compound **78**

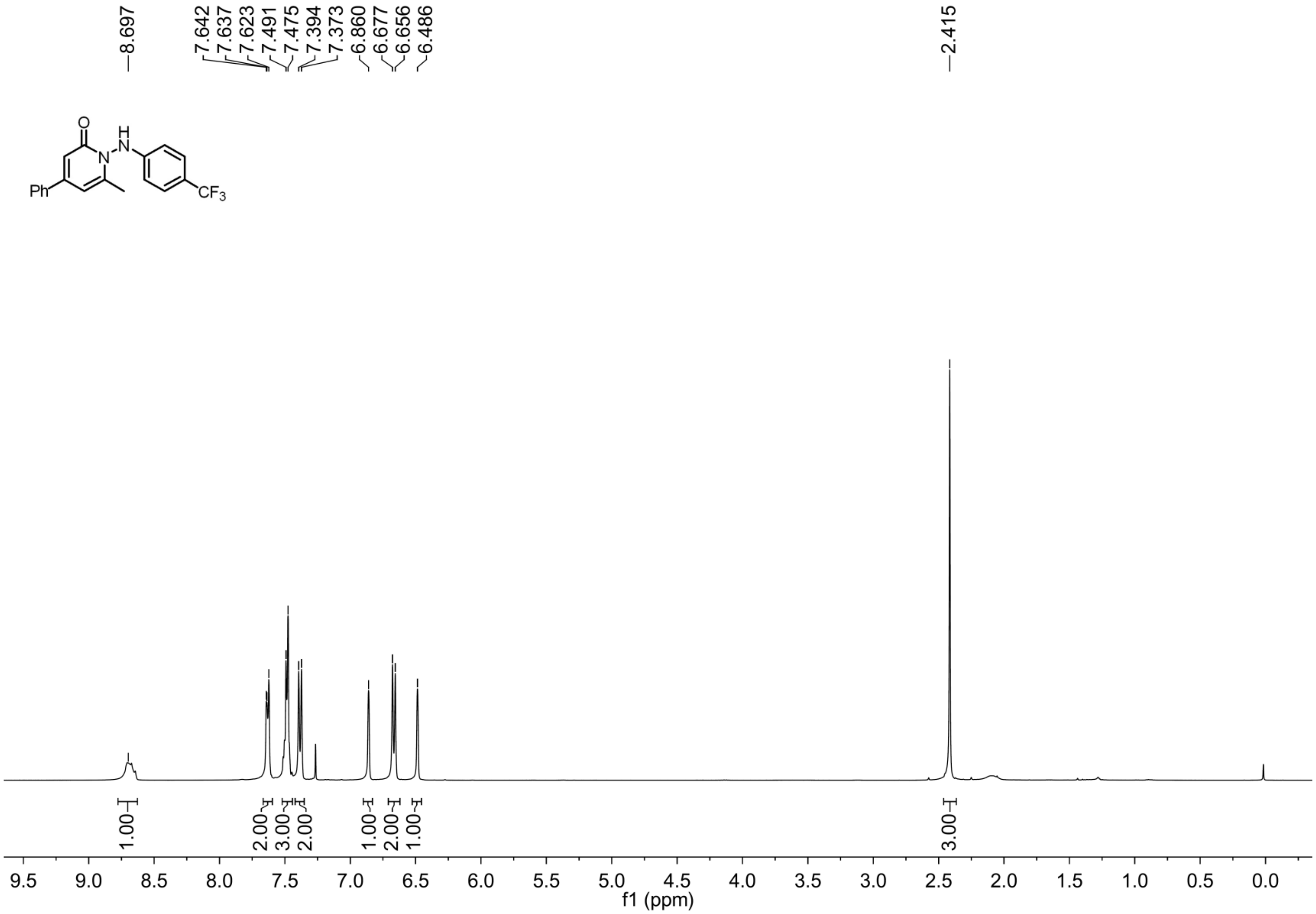

Supplementary Figure 197 ^{1}H NMR Spectrum of Compound **79**

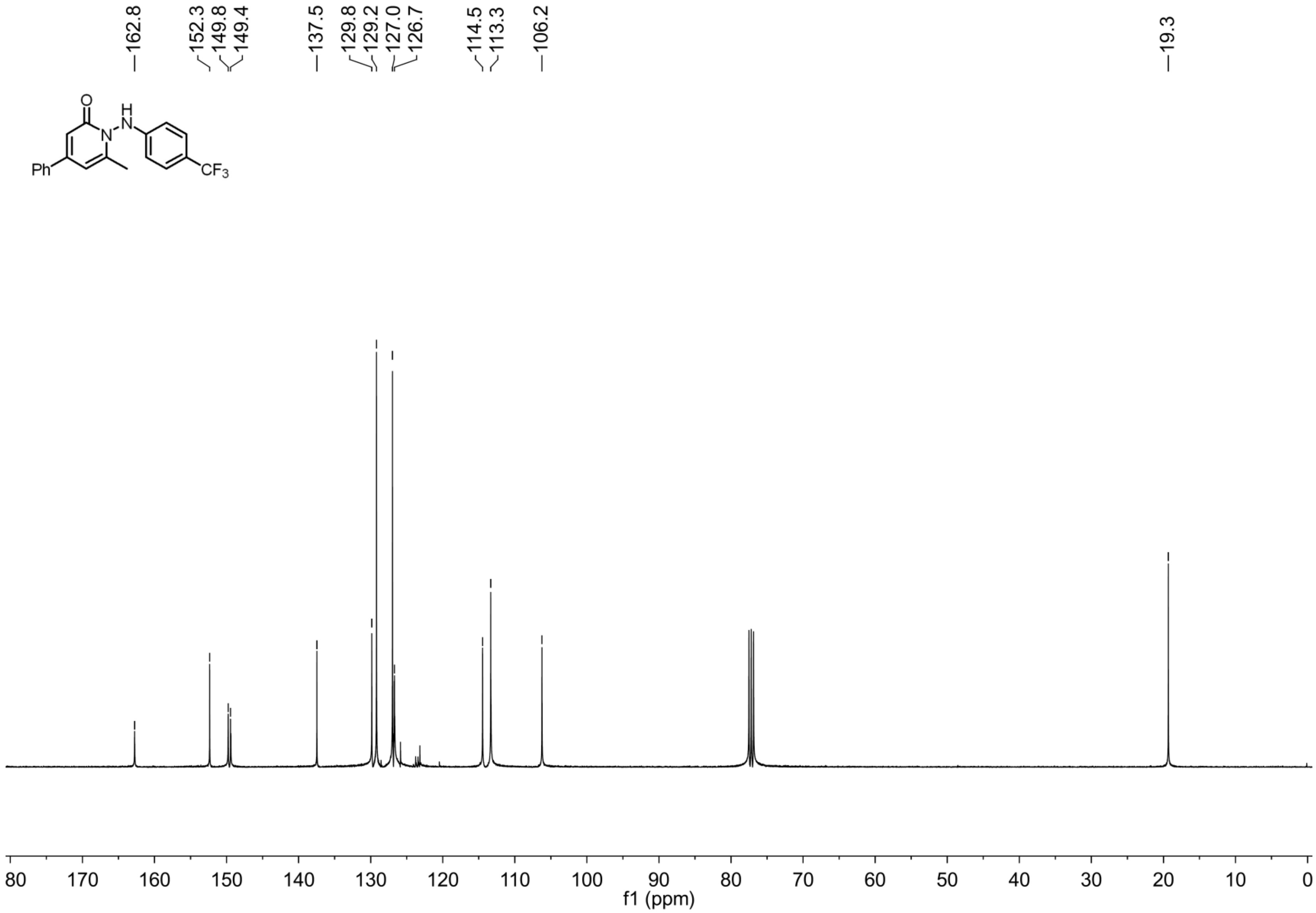

Supplementary Figure 198 ^{13}C NMR Spectrum of Compound **79**

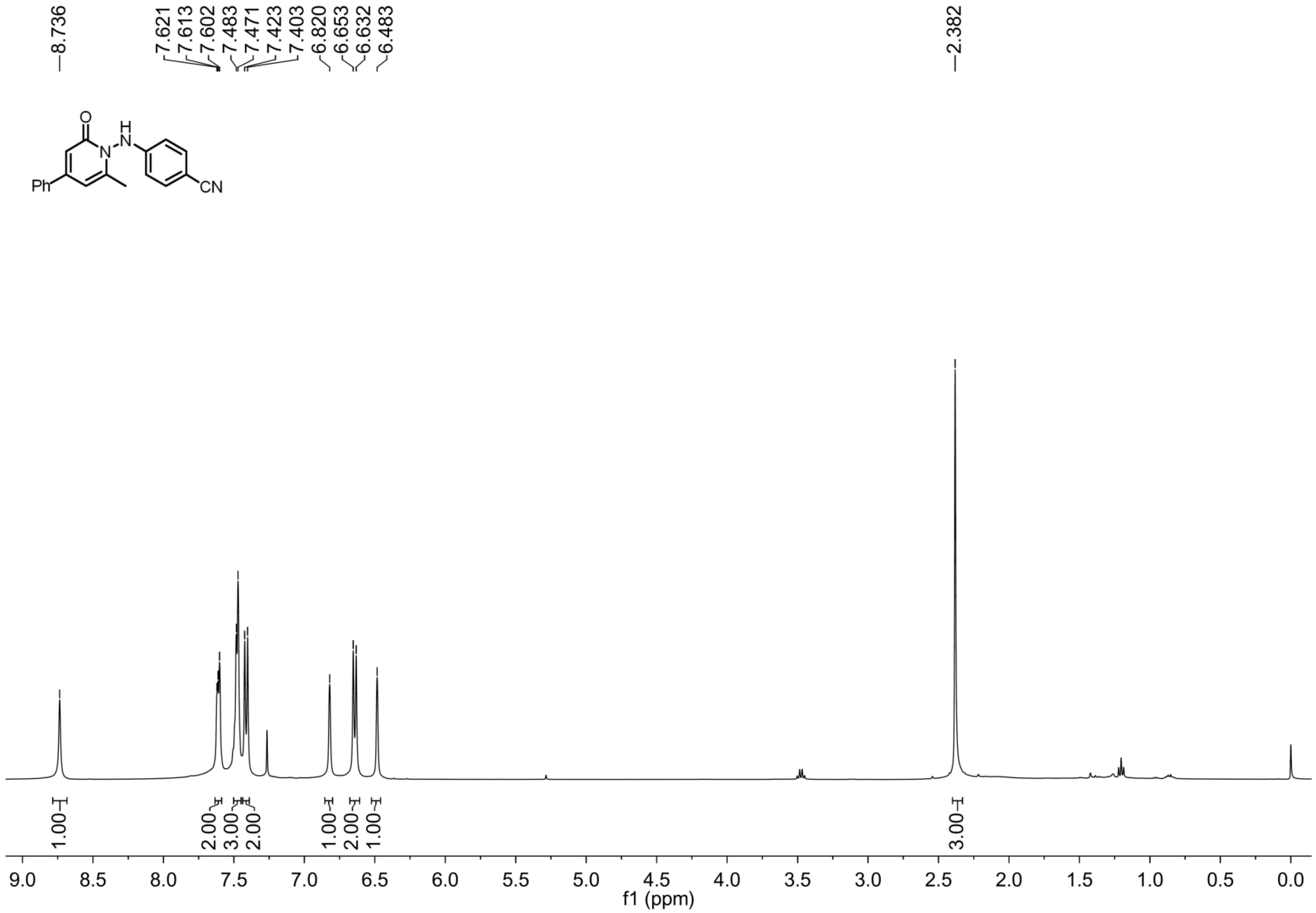

Supplementary Figure 199 ^{1}H NMR Spectrum of Compound **80**

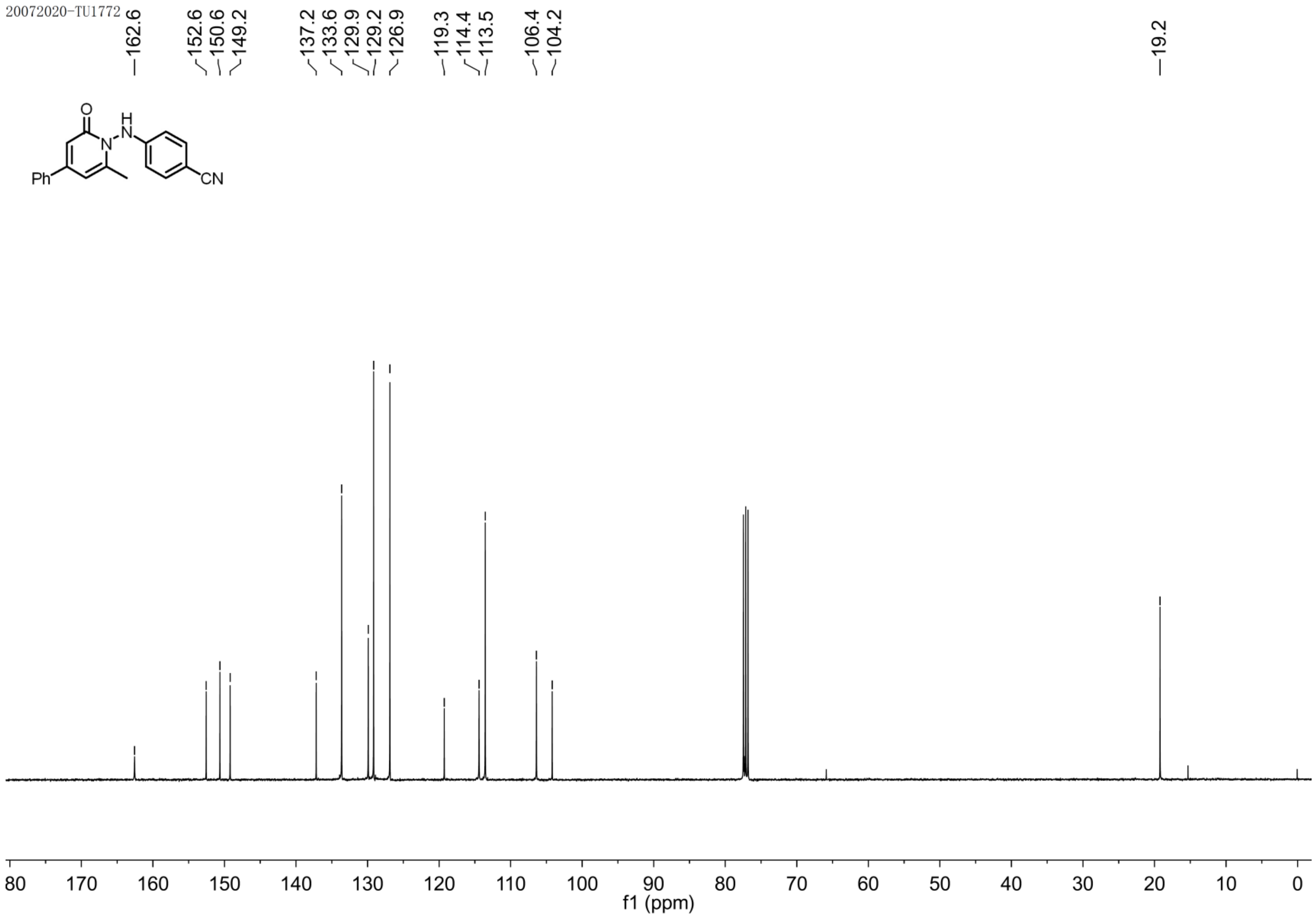

Supplementary Figure 200 ^{13}C NMR Spectrum of Compound **80**

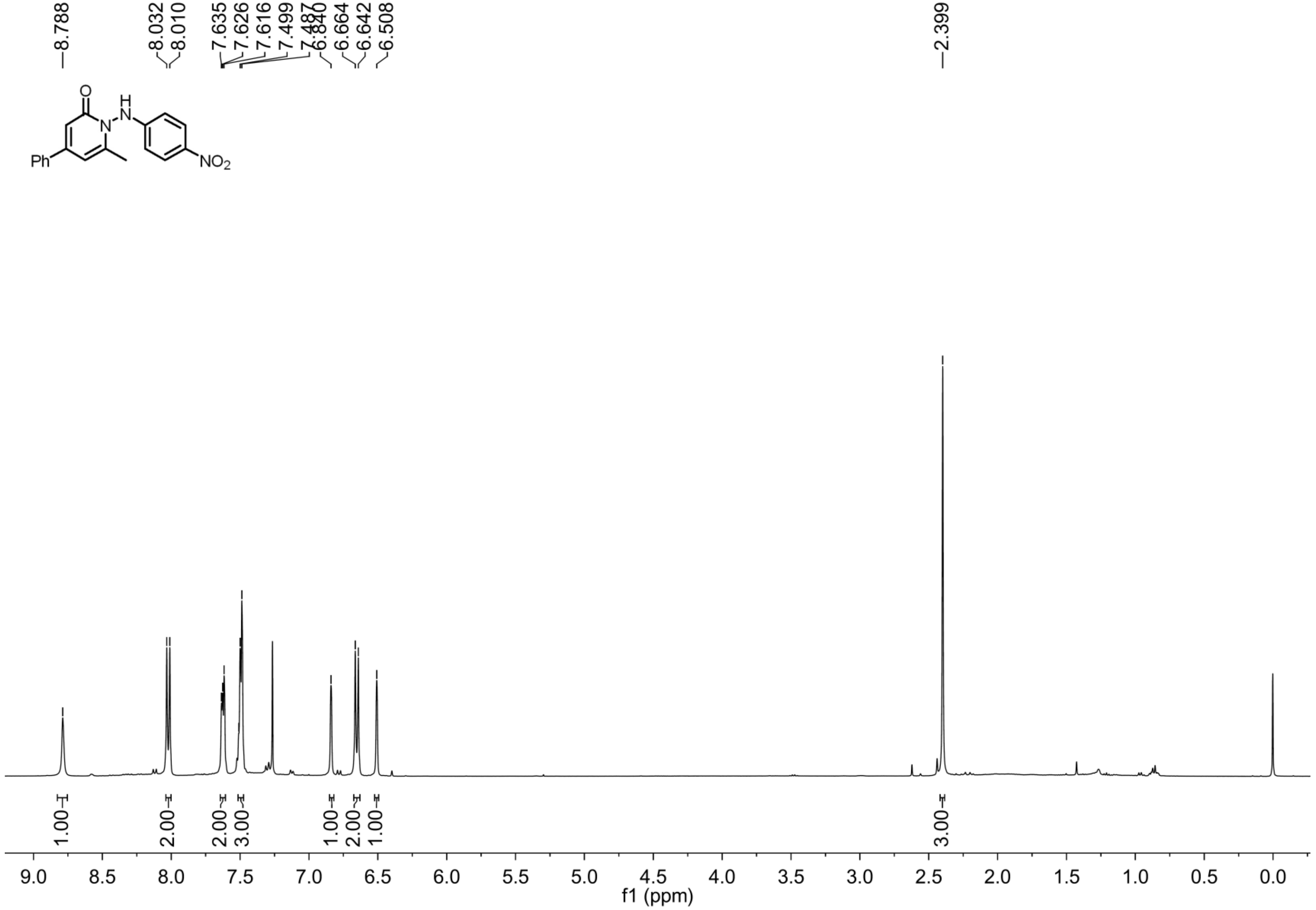

Supplementary Figure 201 ^{1}H NMR Spectrum of Compound **81**

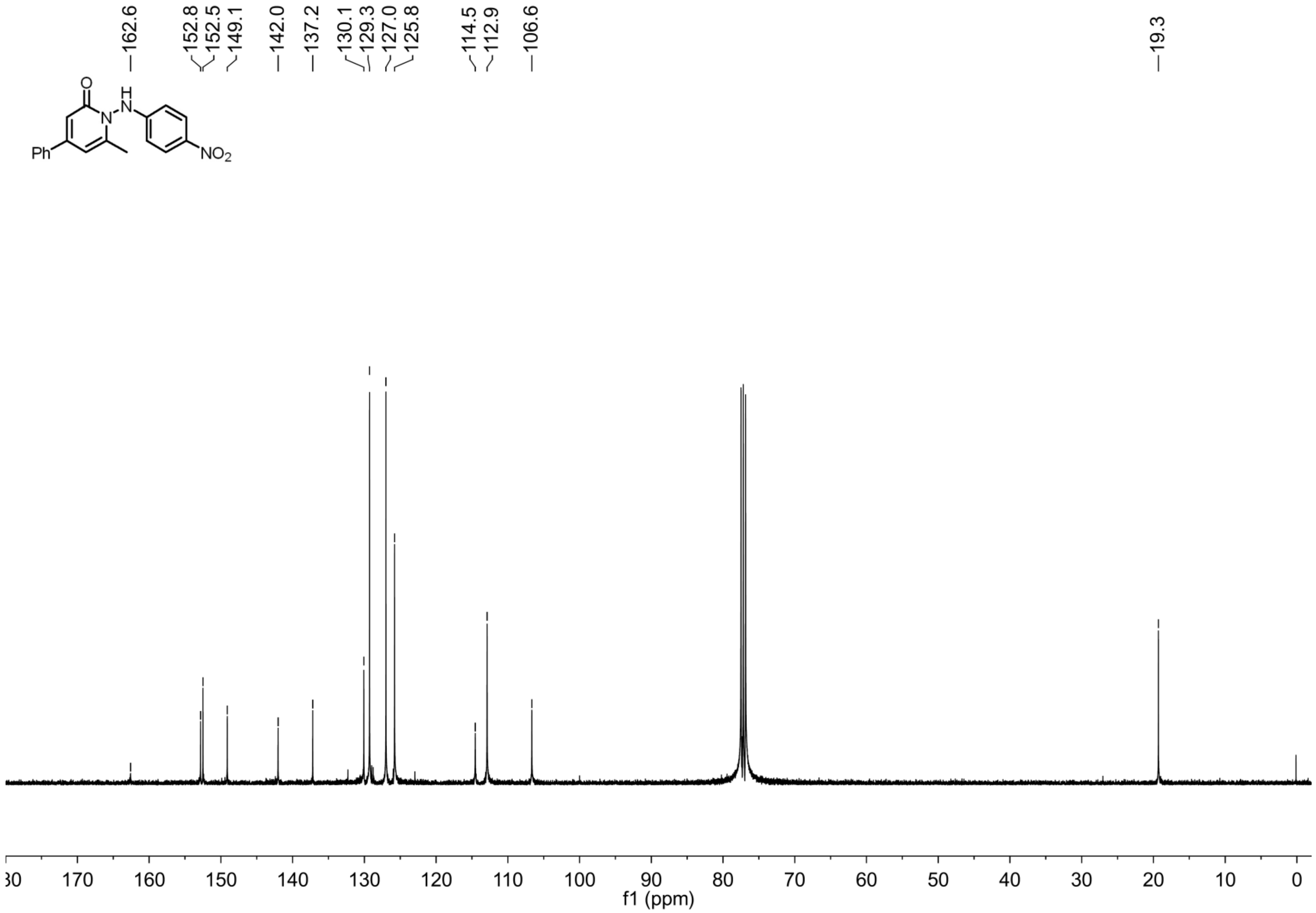

Supplementary Figure 202 ^{13}C NMR Spectrum of Compound **81**

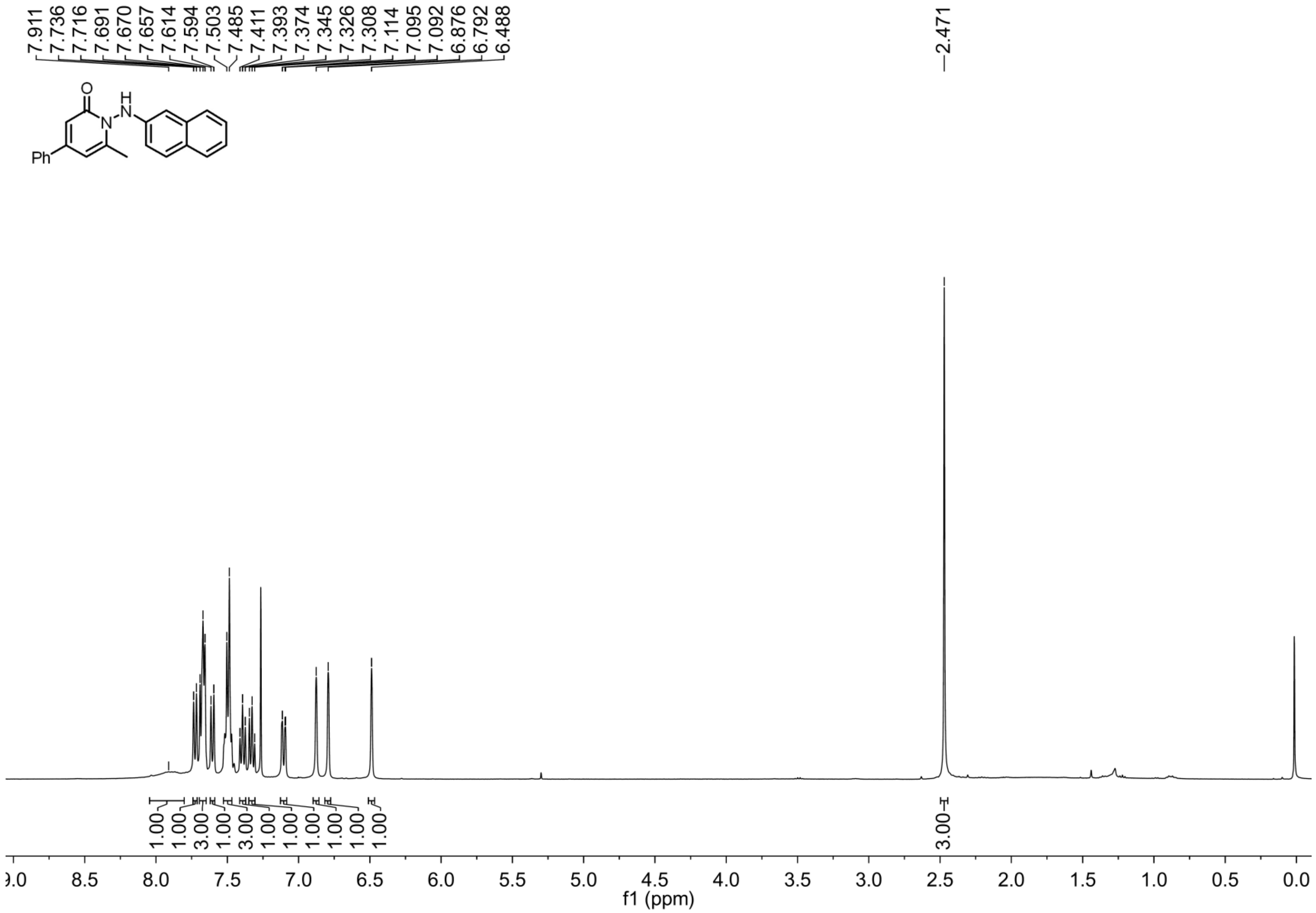

Supplementary Figure 203 ^{1}H NMR Spectrum of Compound **82**

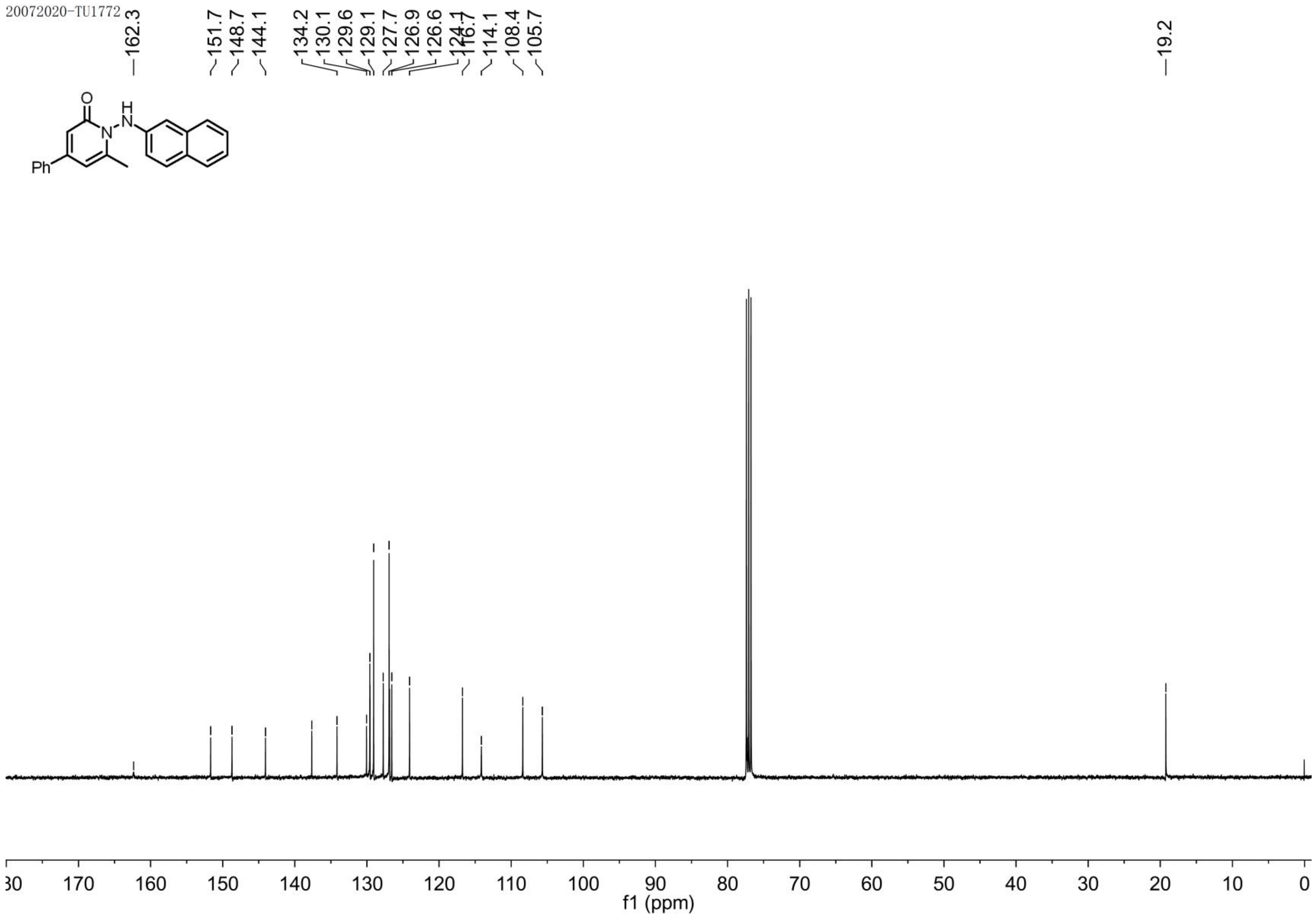

Supplementary Figure 204 ^{13}C NMR Spectrum of Compound **82**

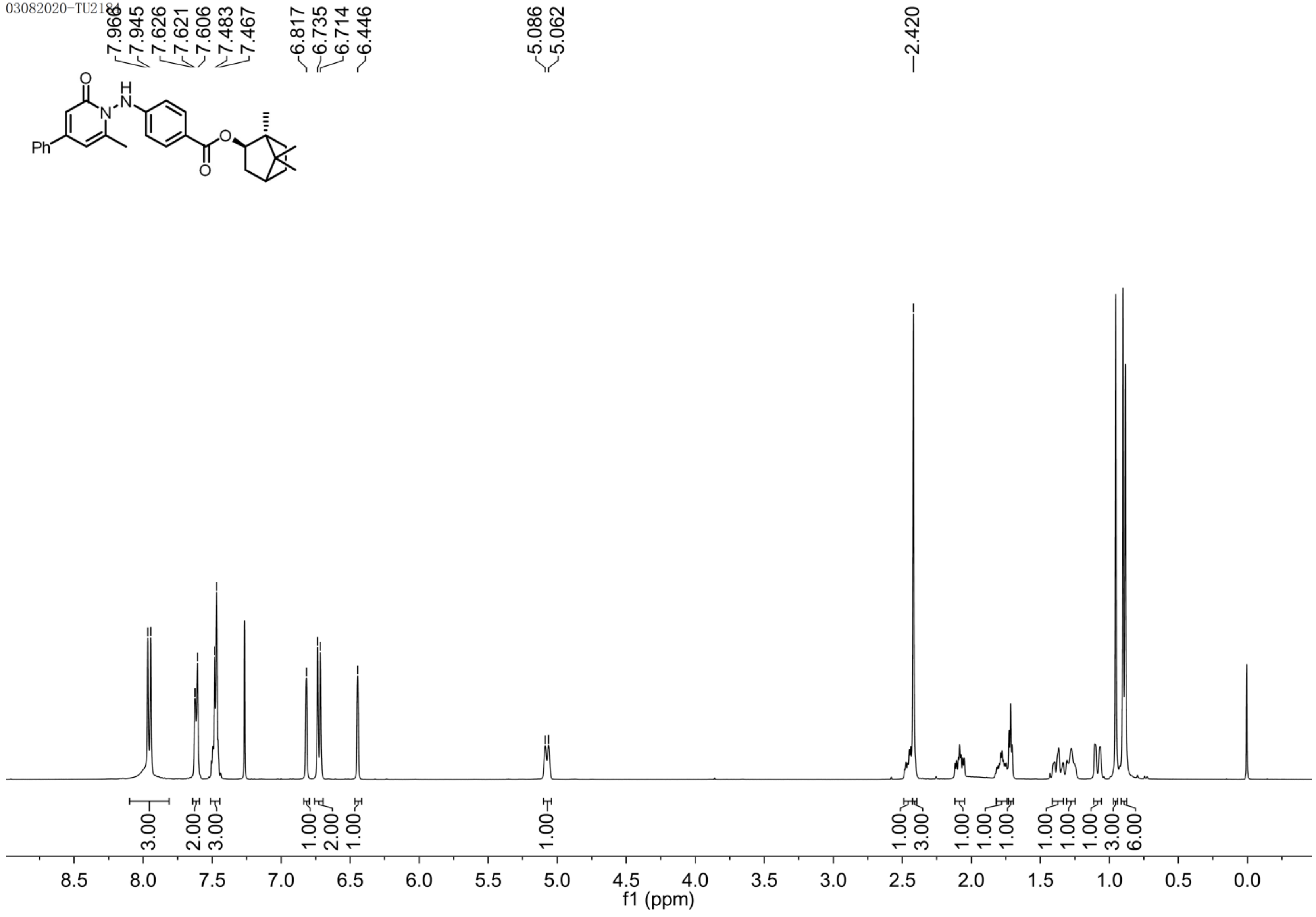

Supplementary Figure 205 ^{1}H NMR Spectrum of Compound **83**

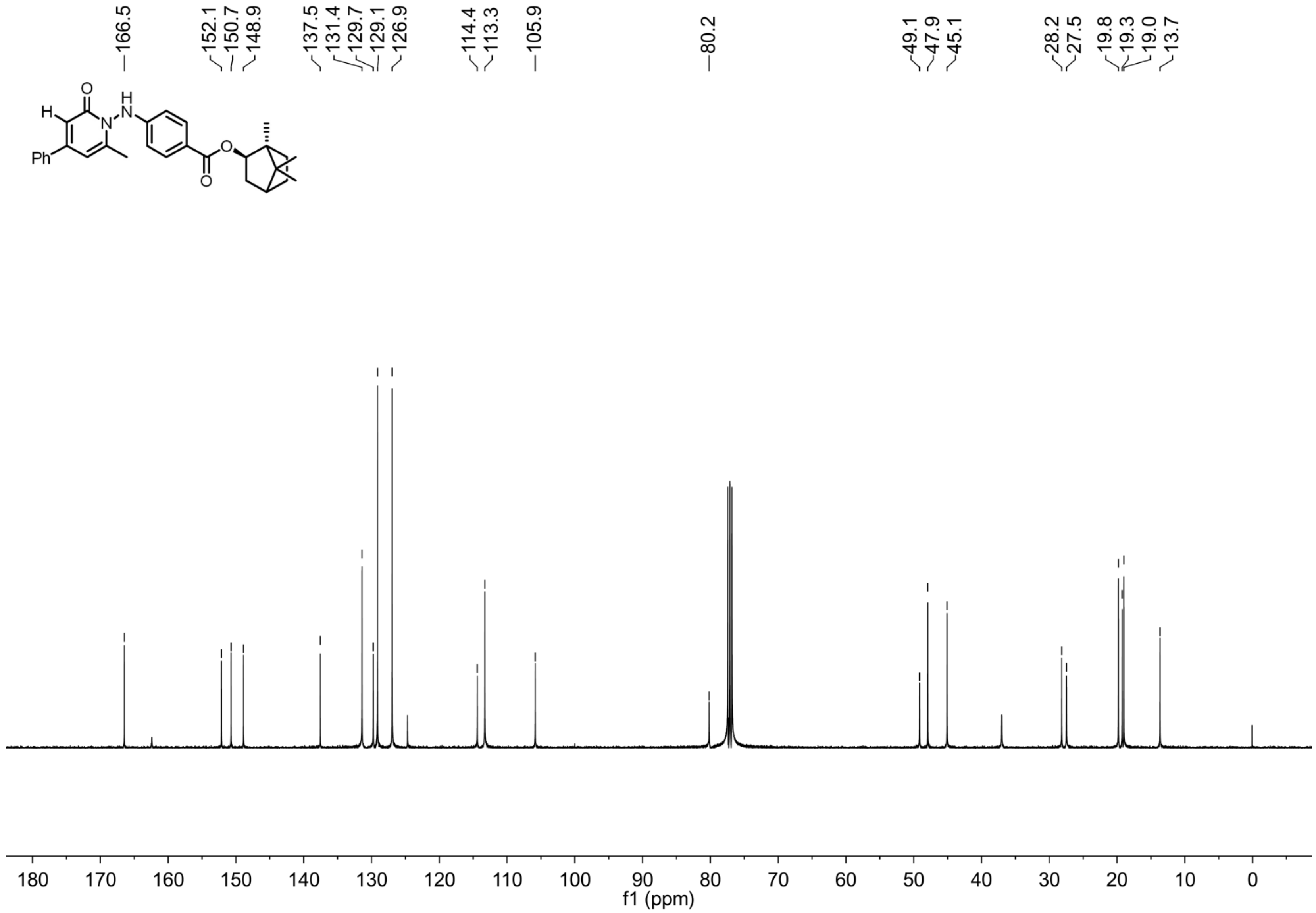

Supplementary Figure 206 ^{13}C NMR Spectrum of Compound **83**

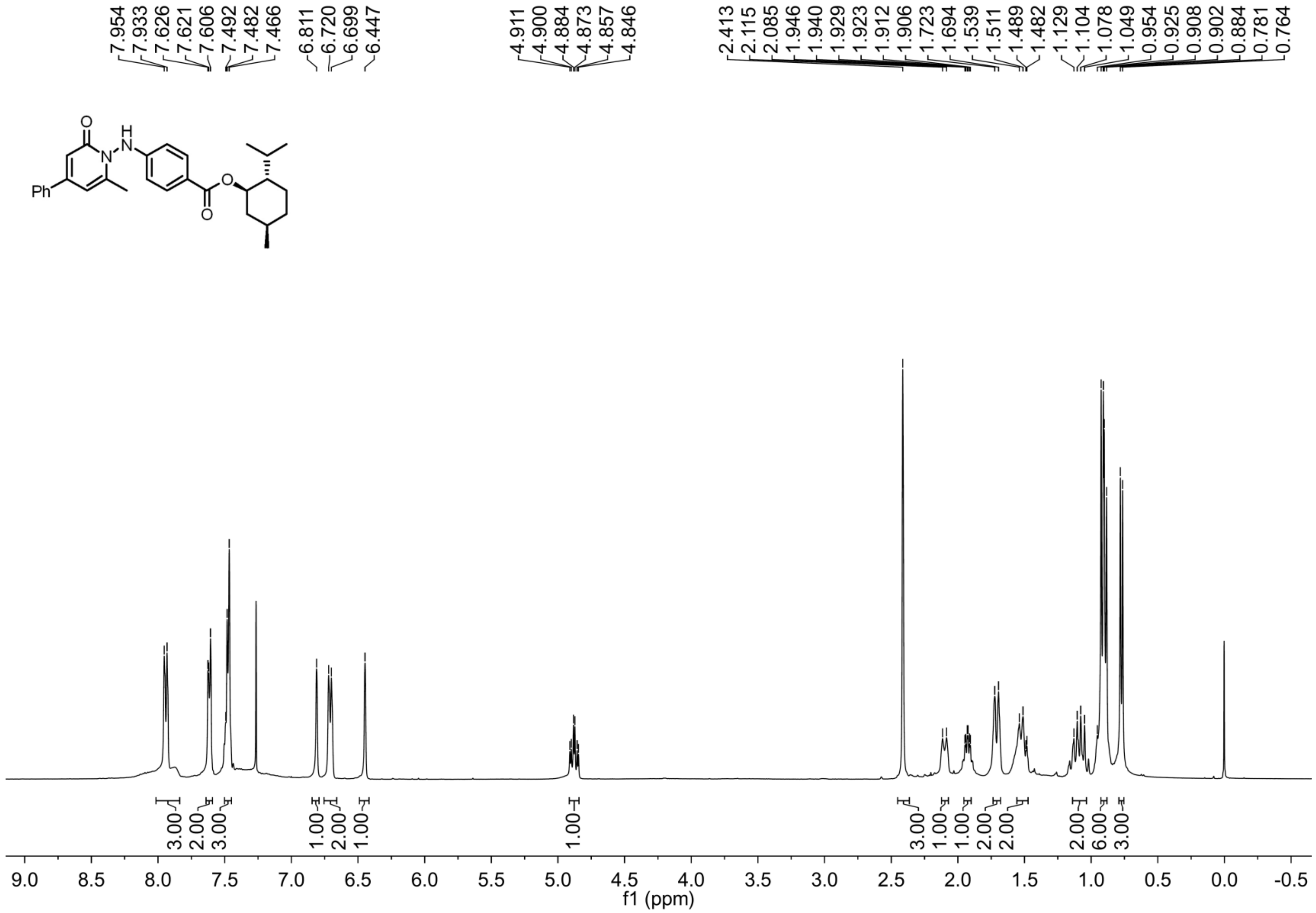

Supplementary Figure 207 ^{1}H NMR Spectrum of Compound **84**

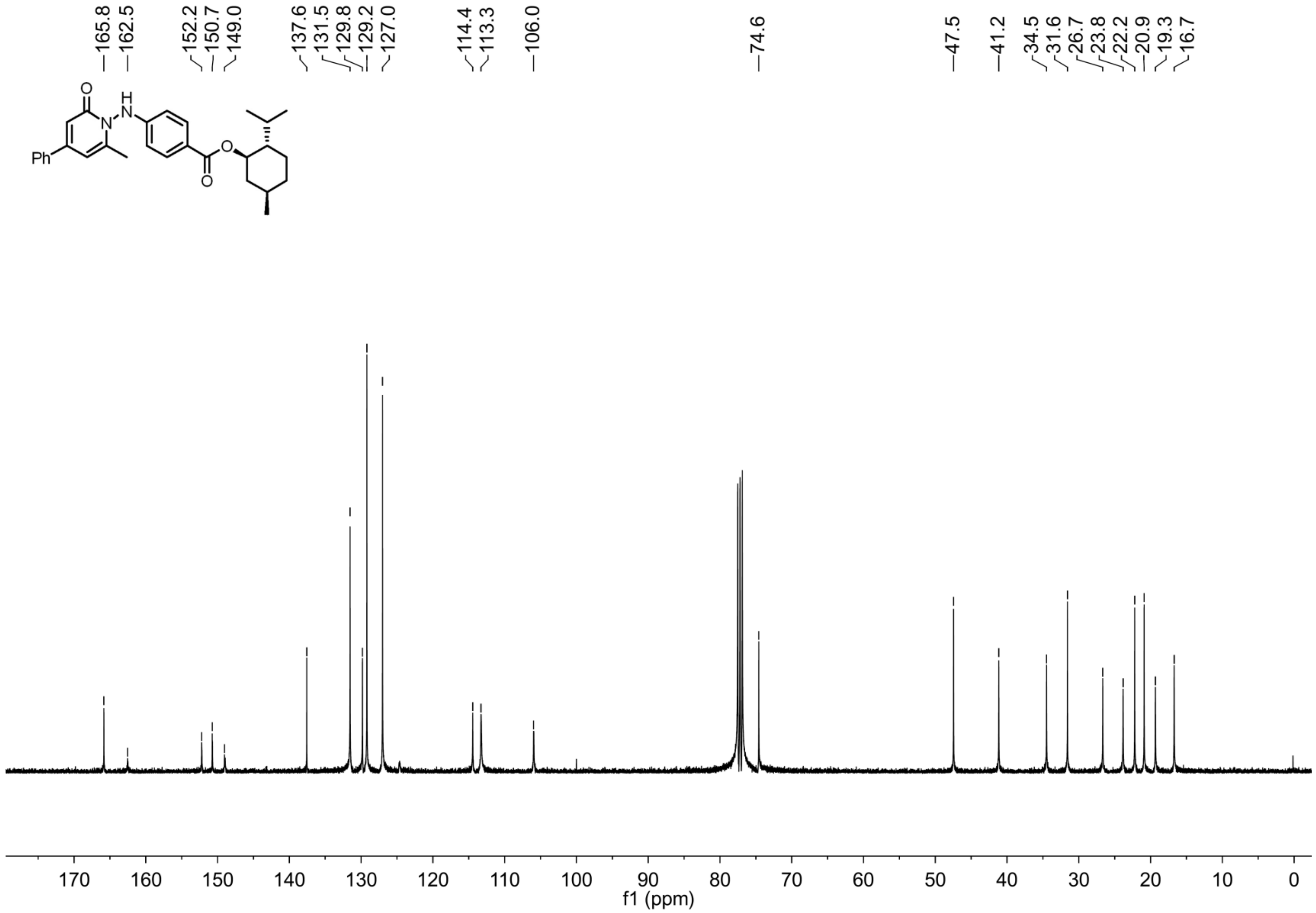

Supplementary Figure 208 ^{13}C NMR Spectrum of Compound **84**

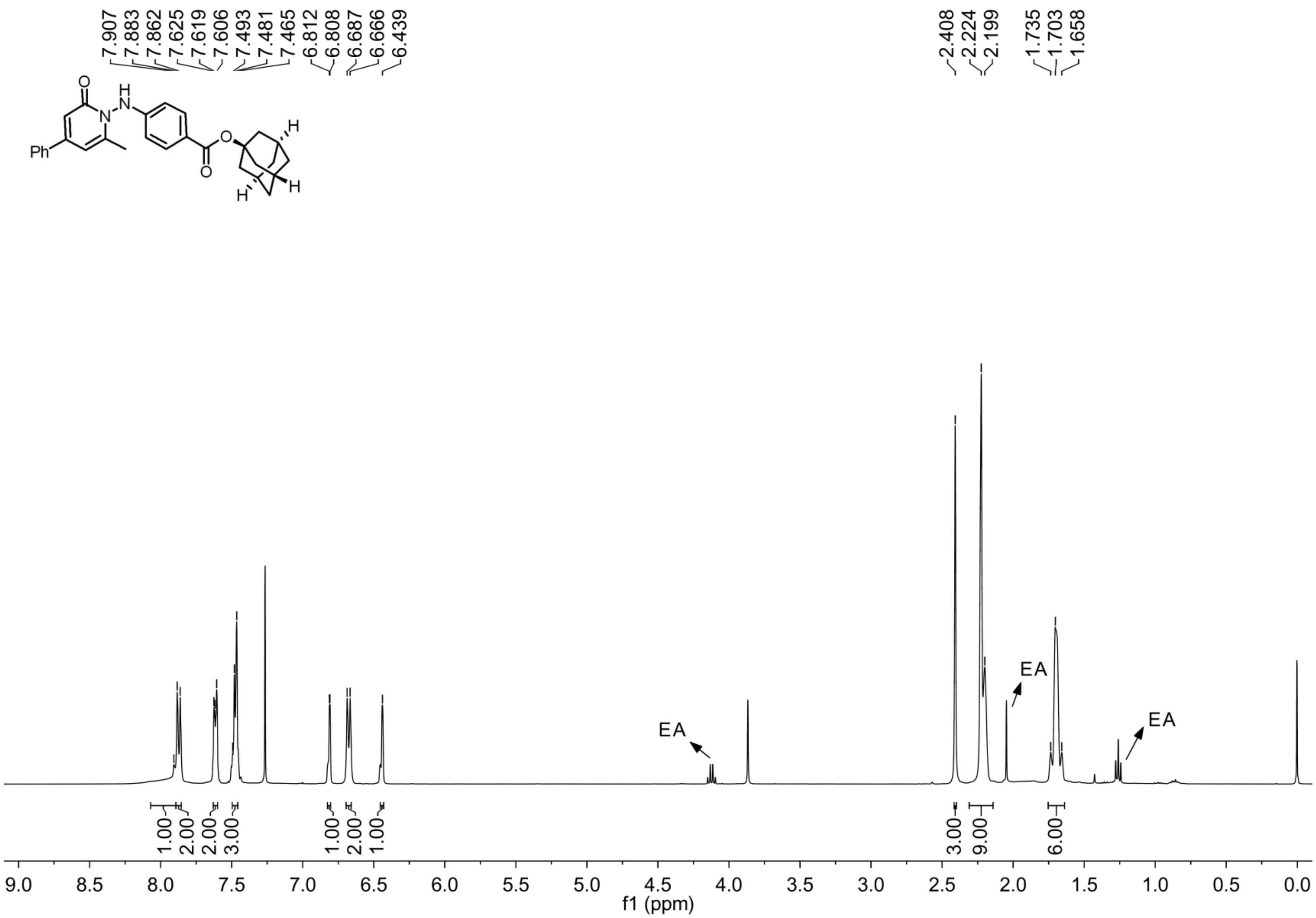

Supplementary Figure 209 ^{1}H NMR Spectrum of Compound **85**

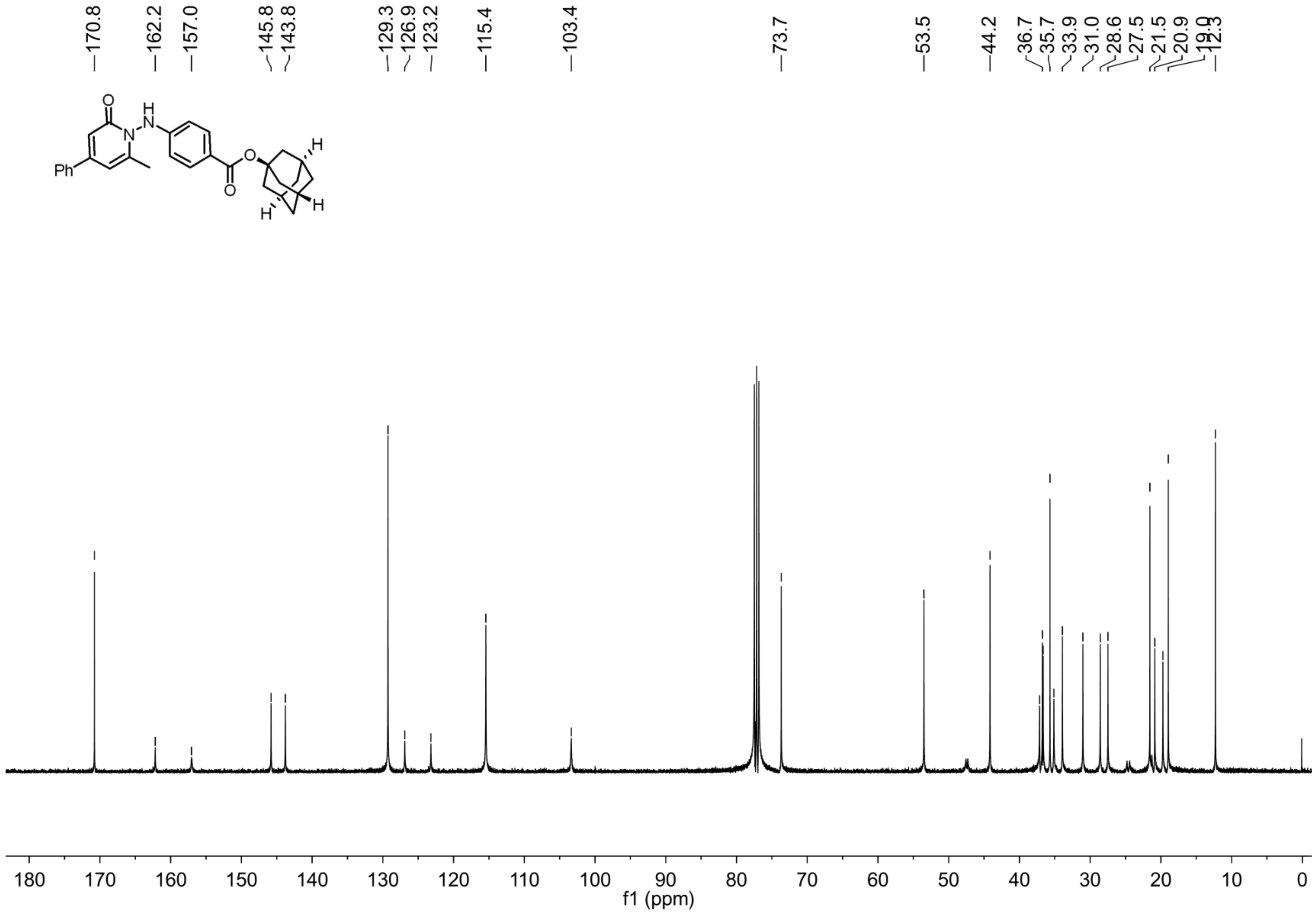

Supplementary Figure 210 ^{13}C NMR Spectrum of Compound **85**

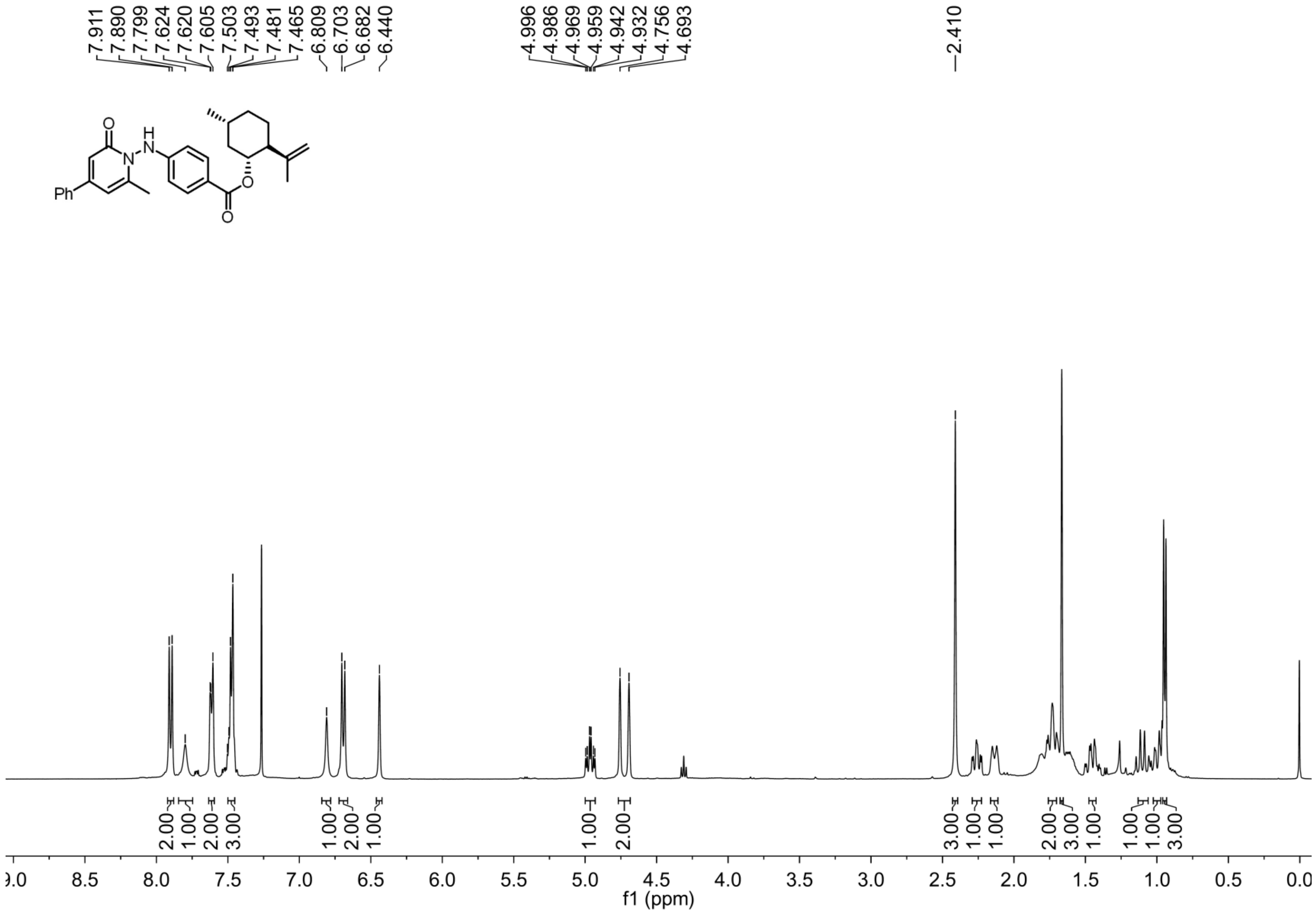

Supplementary Figure 211 ^{1}H NMR Spectrum of Compound **86**

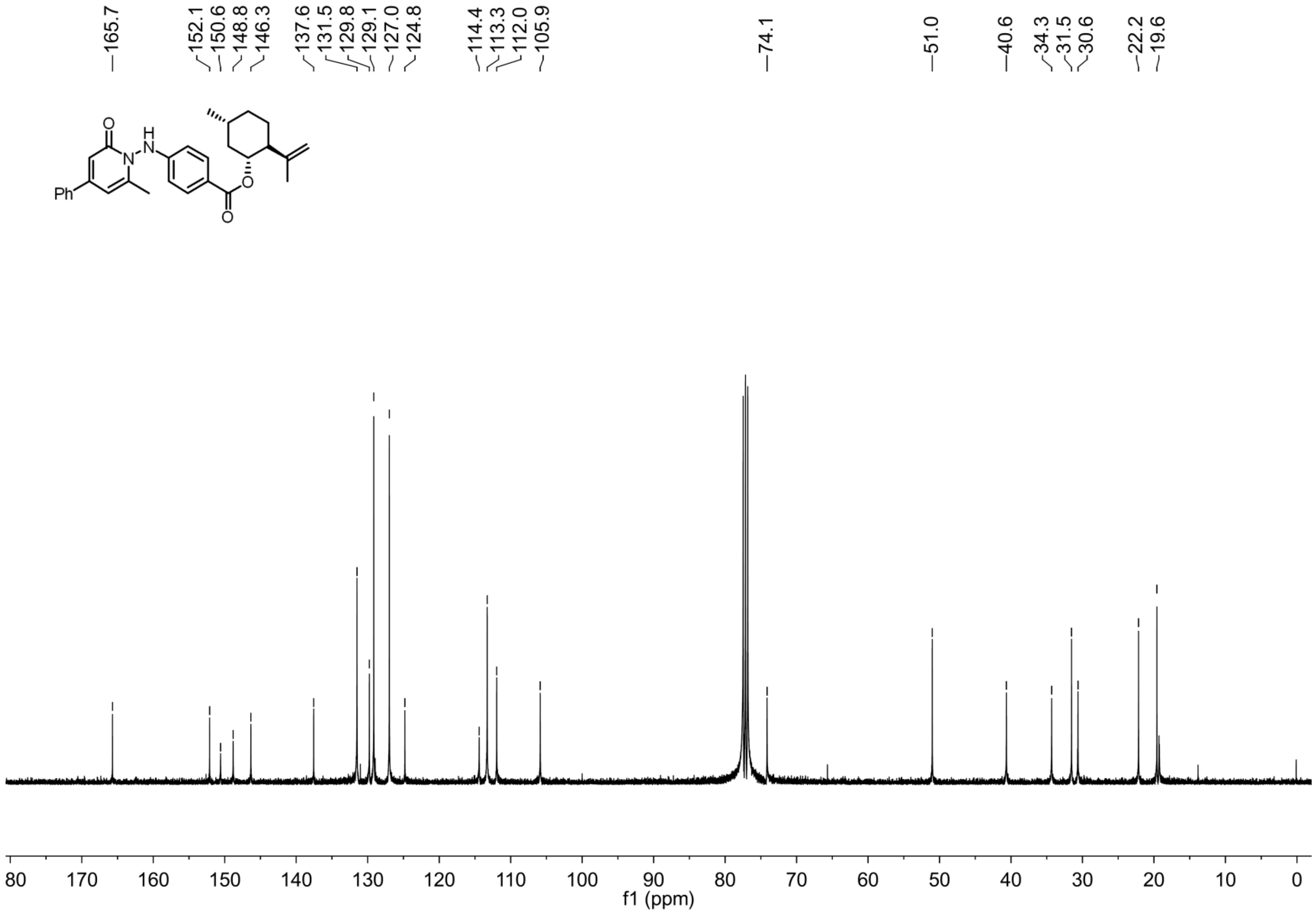

Supplementary Figure 212 ^{13}C NMR Spectrum of Compound **86**

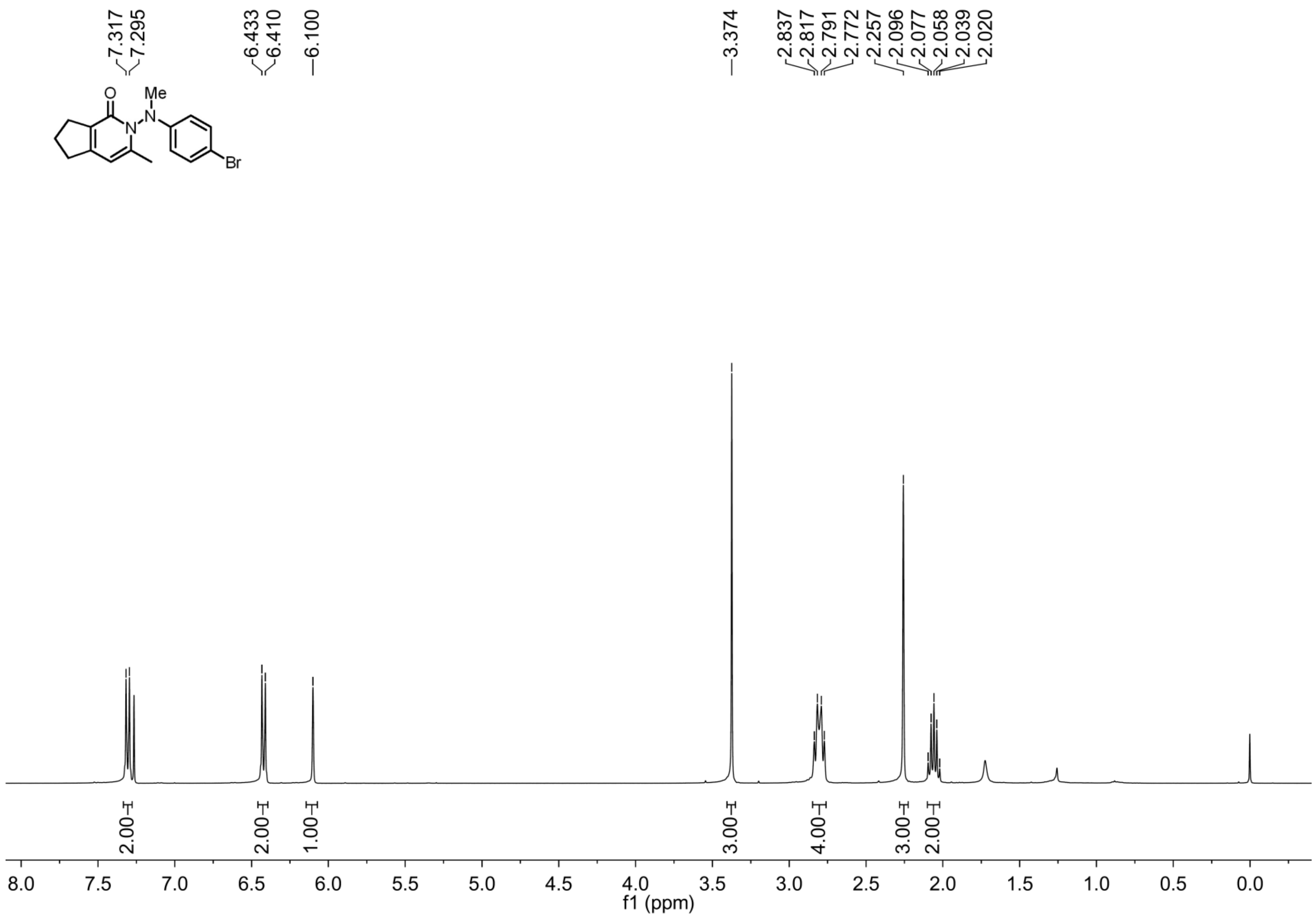

Supplementary Figure 213 ^{1}H NMR Spectrum of Compound **87**

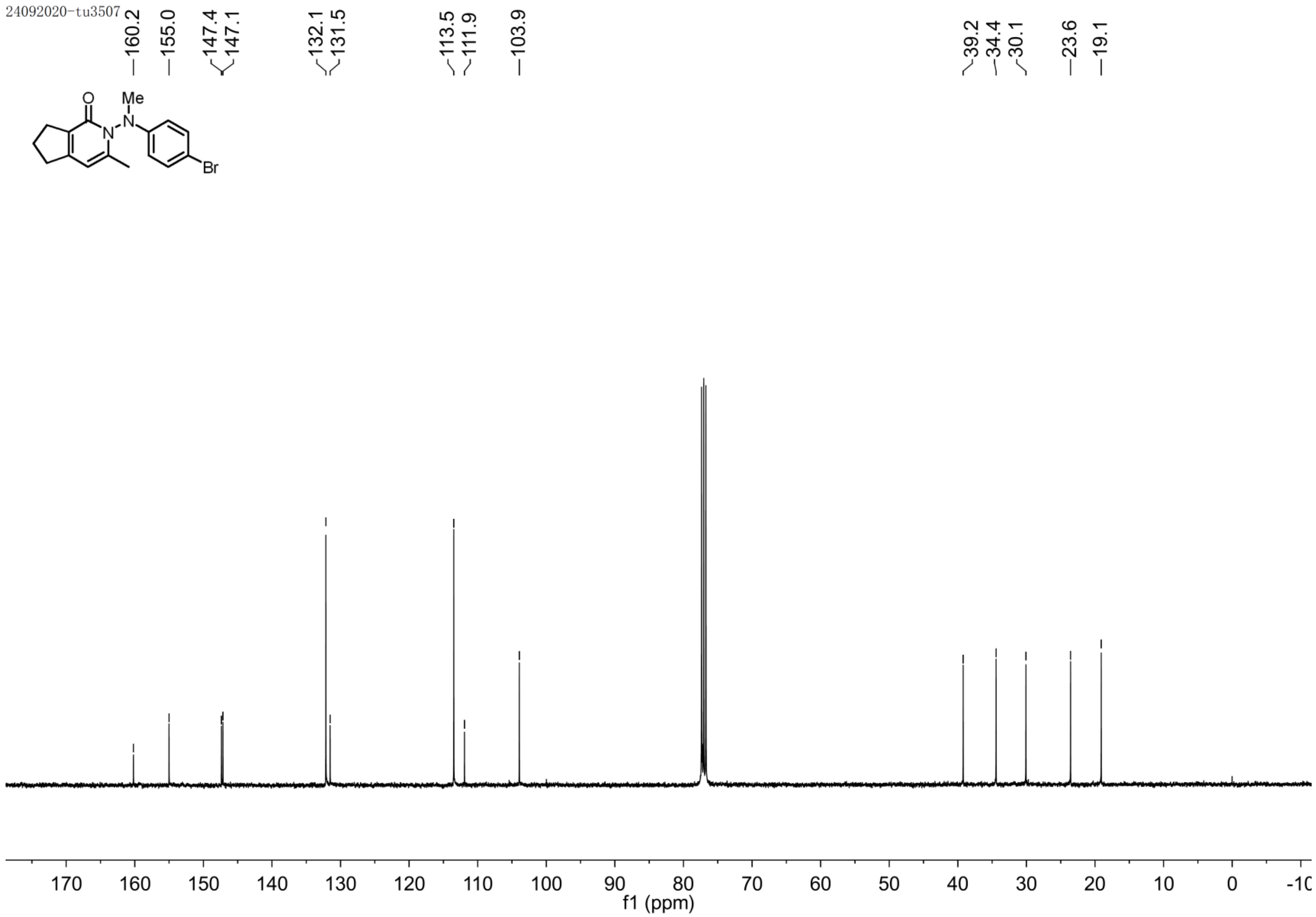

Supplementary Figure 214 ^{13}C NMR Spectrum of Compound **87**

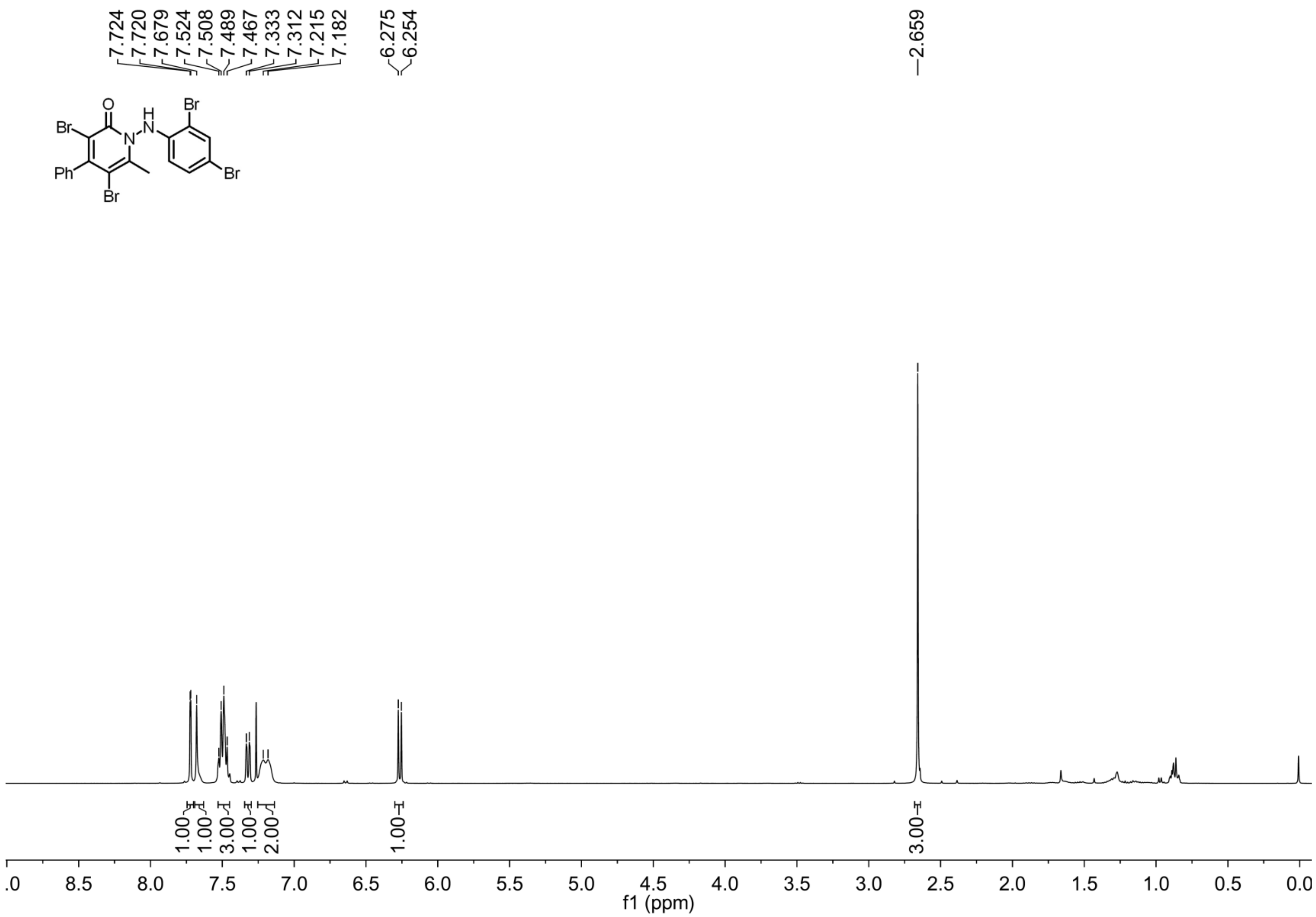

Supplementary Figure 215 ^{1}H NMR Spectrum of Compound **88**

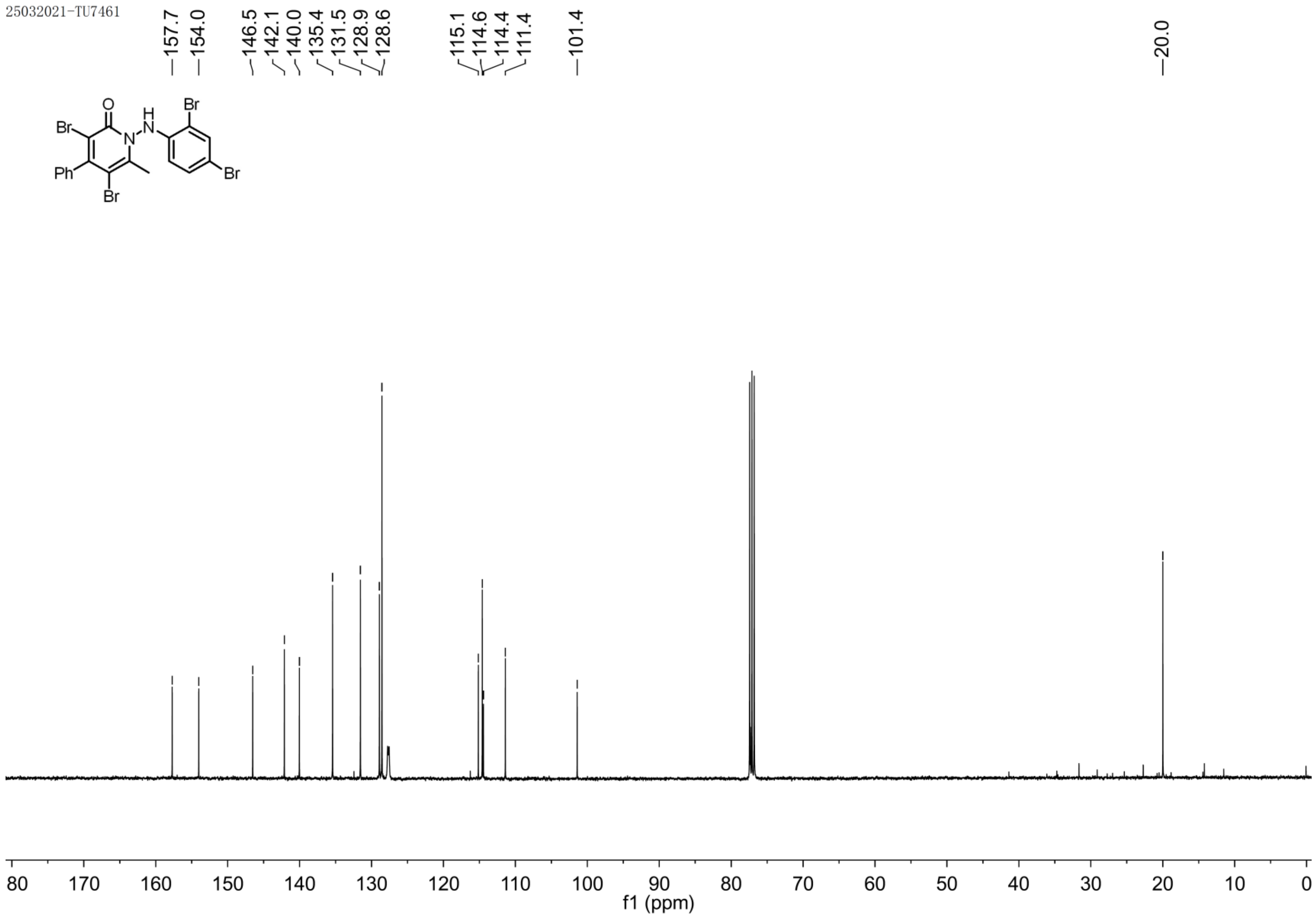

Supplementary Figure 216 ^{13}C NMR Spectrum of Compound **88**

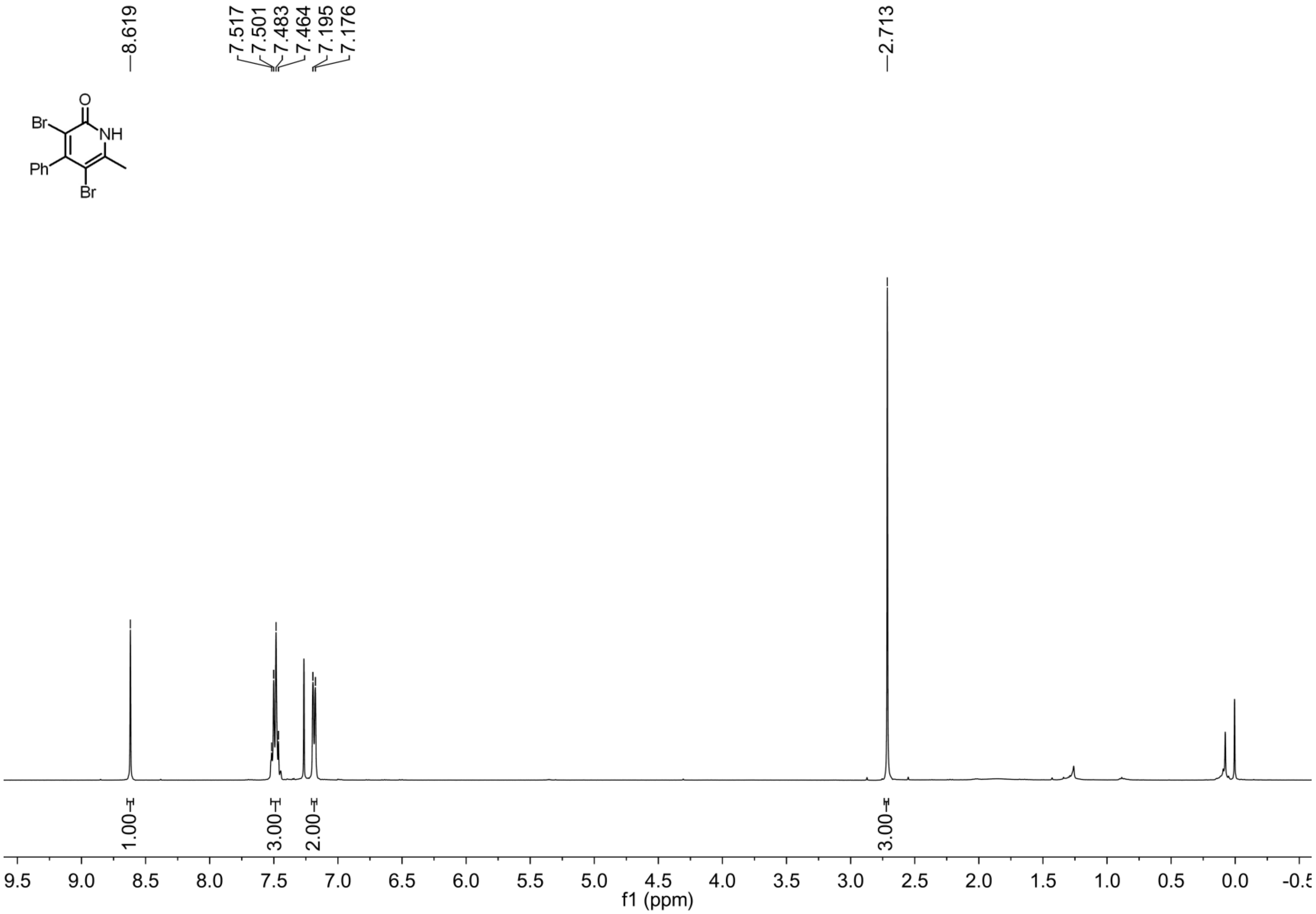

Supplementary Figure 217 ^{1}H NMR Spectrum of Compound **89**

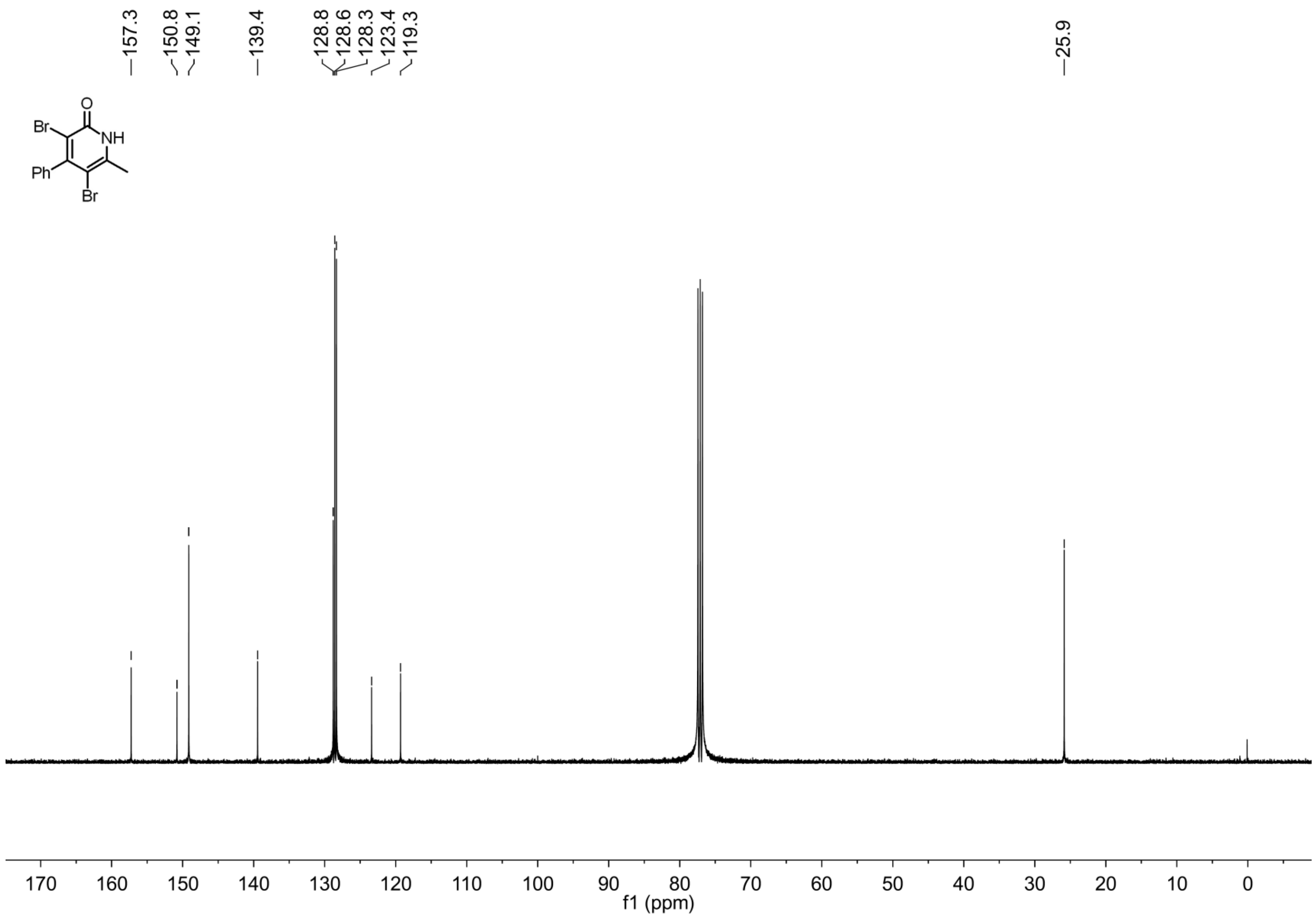

Supplementary Figure 218 ^{13}C NMR Spectrum of Compound **89**

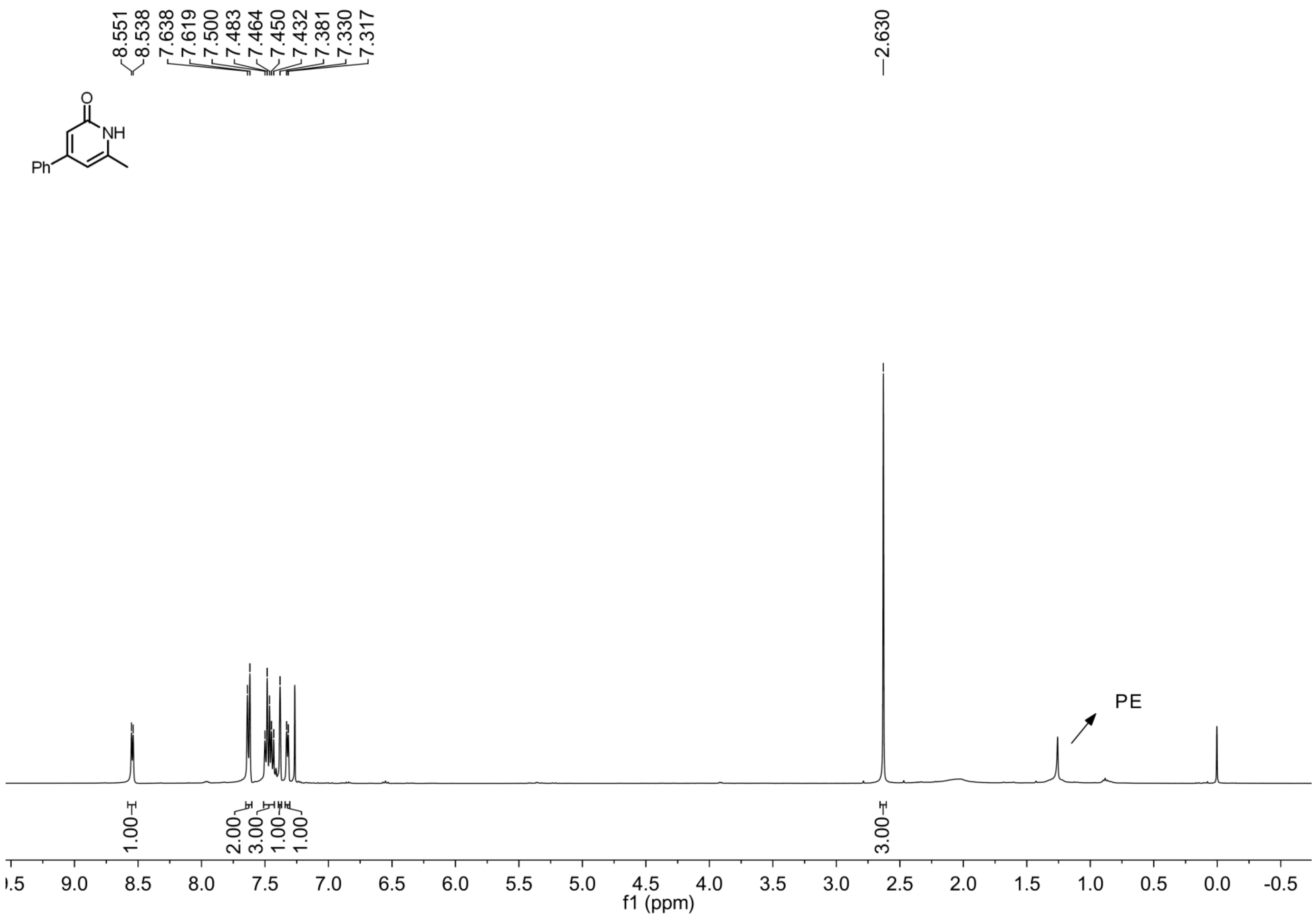

Supplementary Figure 219 ^{1}H NMR Spectrum of Compound **90**

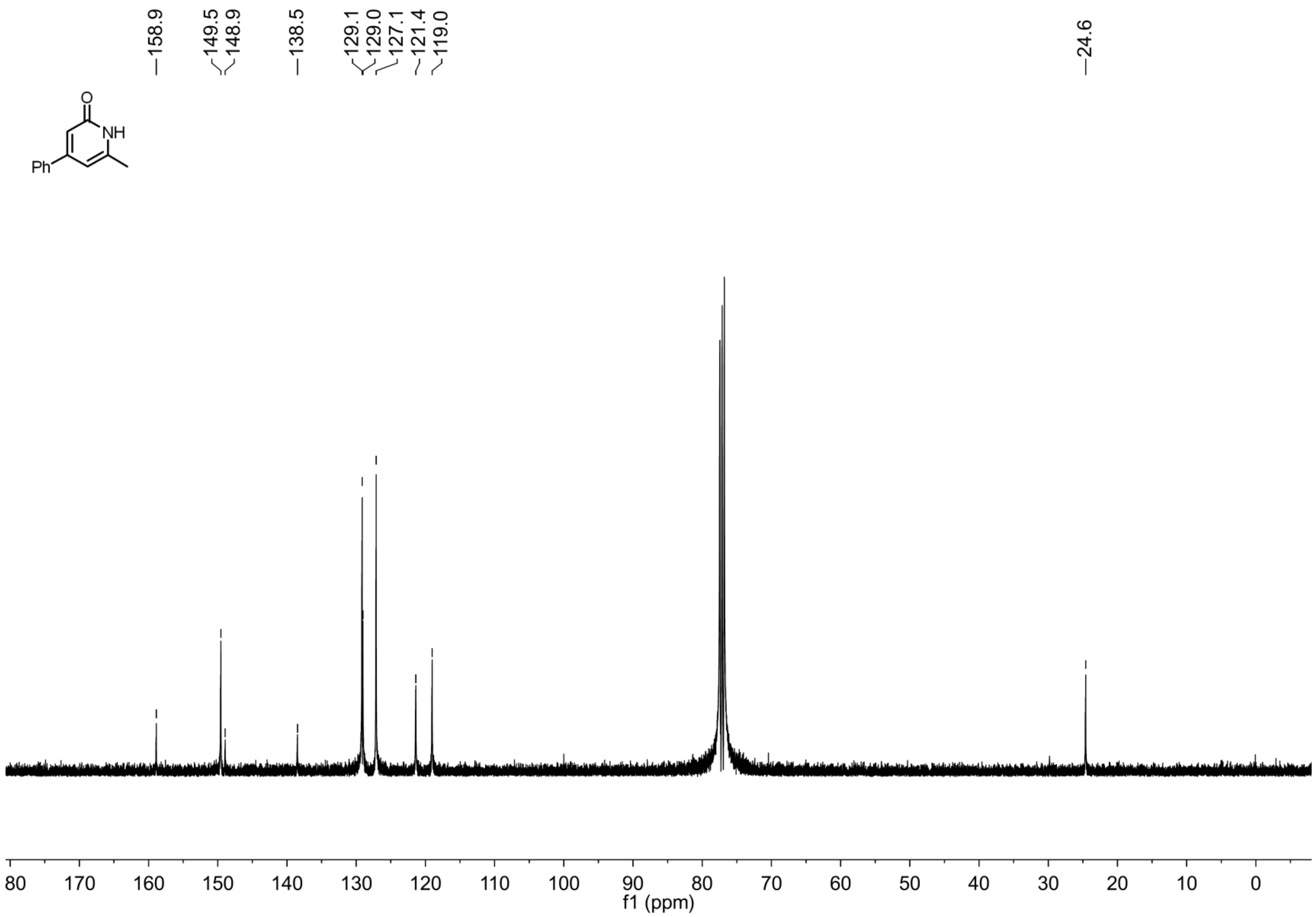

Supplementary Figure 220 ^{13}C NMR Spectrum of Compound **90**

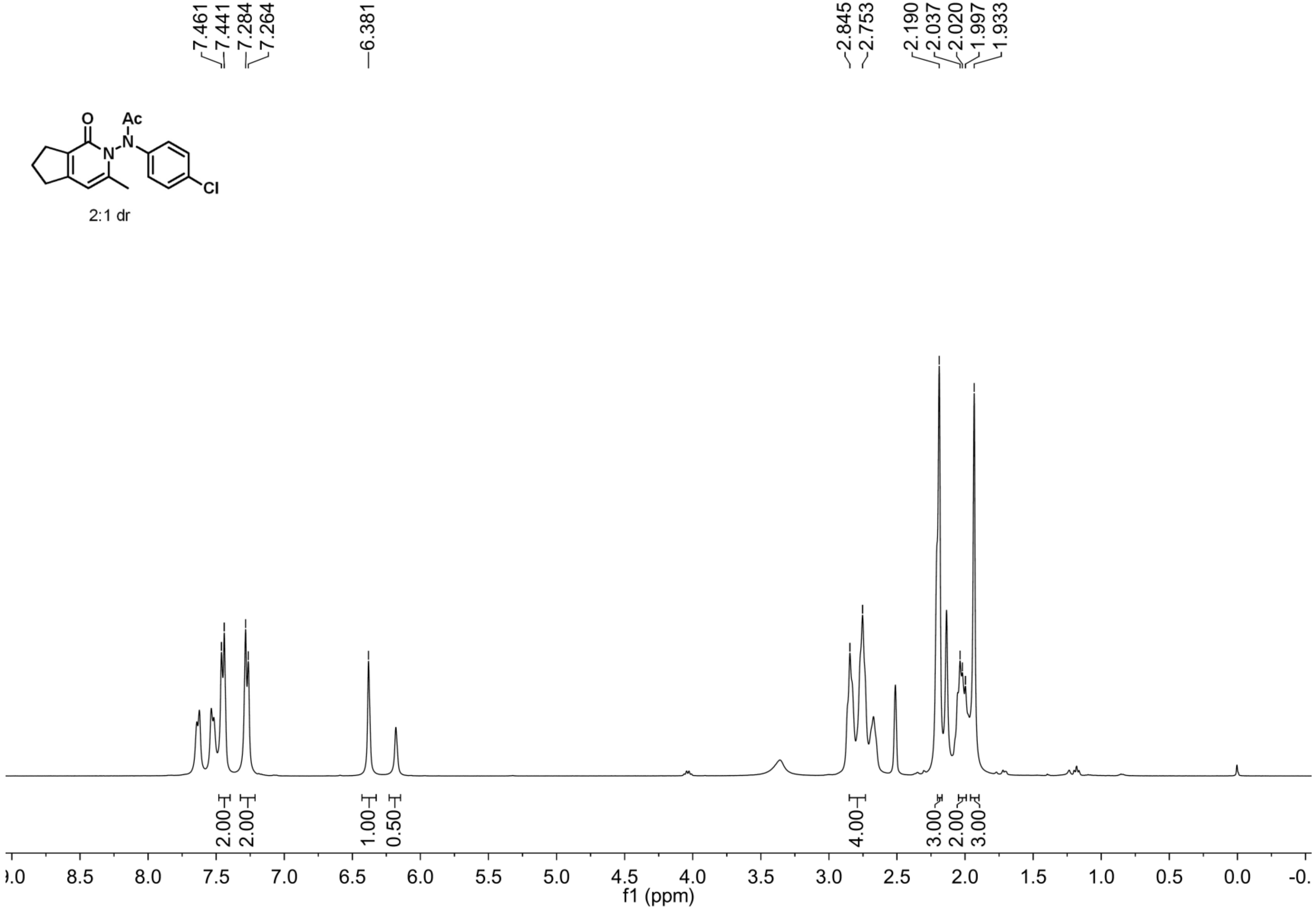

Supplementary Figure 221 ^{1}H NMR Spectrum of Compound ***N*-Ac 10**

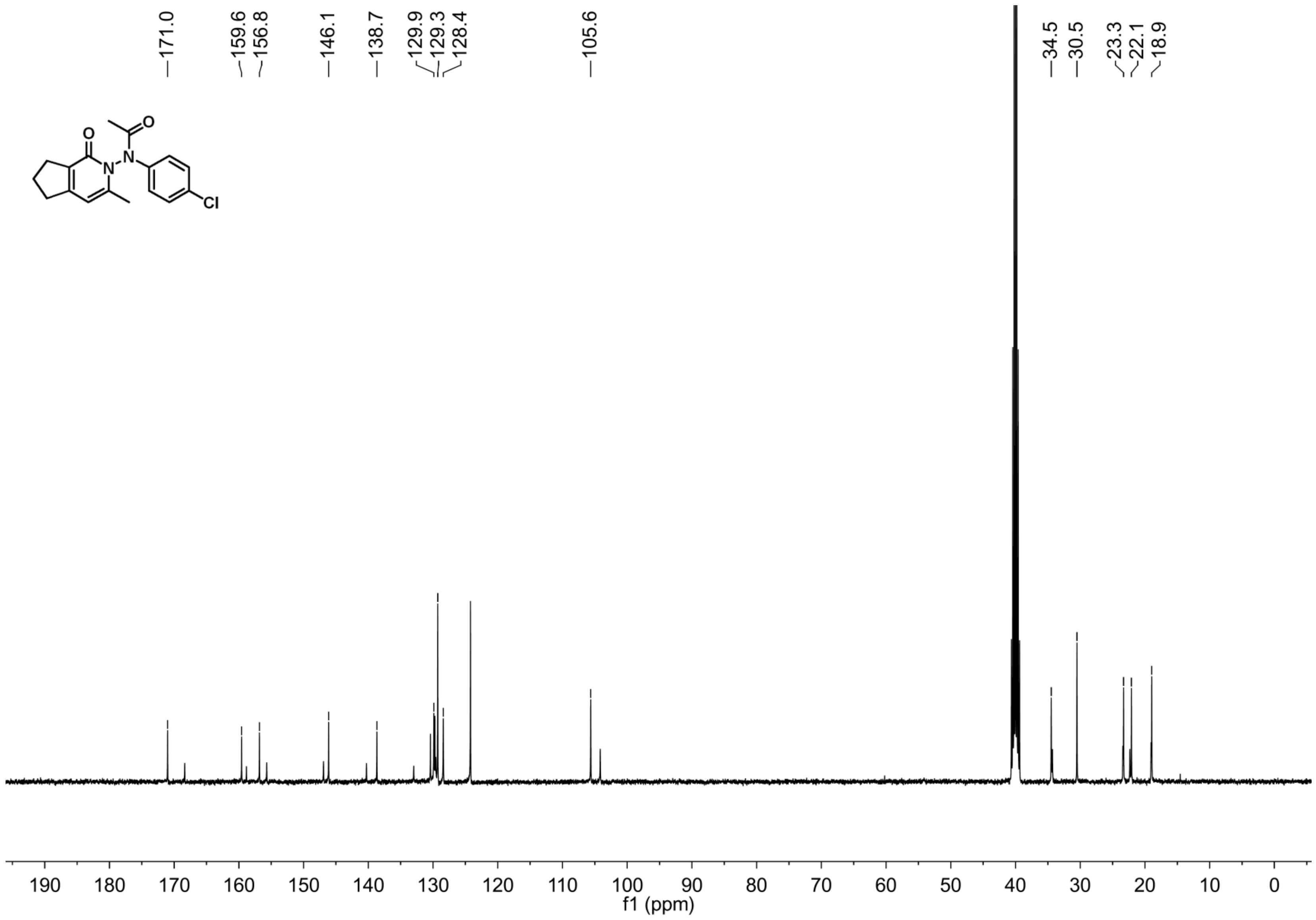

Supplementary Figure 222 ^{13}C NMR Spectrum of Compound ***N*-Ac 10**

3. Supplementary References

1. Lemière, G. et al. Generation and Trapping of Cyclopentenylidene Gold Species: Four Pathways to Polycyclic Compounds. *J. Am. Chem. Soc.* **131**, 2993-3006 (2009).
2. Zhang, L.-M. & Wang, S.-Z. Efficient Synthesis of Cyclopentenones from Enynyl Acetates via Tandem Au(I)-Catalyzed 3,3-Rearrangement and the Nazarov Reaction. *J. Am. Chem. Soc.* **128**, 1442-1443 (2006).
3. Shi, X.-D. Gorin, D. J. & Toste, F. D. Synthesis of 2-Cyclopentenones by Gold(I)-Catalyzed Rautenstrauch Rearrangement. *J. Am. Chem. Soc.* **127**, 5802-5803 (2005).
4. Wang, T. et al. Enantioselective Cyanation via Radical-Mediated C–C Single Bond Cleavage for Synthesis of Chiral Dinitriles. *Nat. Commun.* **10**, 5373 (2019).
5. Ai, W.-Y. Liu, Y.-Q. Wang, Q. Lu, Z.-L. & Liu, Q. Cu-Catalyzed Redox-Neutral Ring Cleavage of Cycloketone O-Acyl Oximes: Chemodivergent Access to Distal Oxygenated Nitriles. *Org. Lett.* **20**, 409-412 (2018).
6. Zuo, H.-D. et al. Copper-Catalyzed Asymmetric Deconstructive Alkynylation of Cyclic Oximes. *ACS Catal.* **11**, 6010-6019 (2021).
7. Chen, X. et al. Gold(I)-Catalyzed Tandem Cycloisomerization and Fluorination of 1,3(4)-Enyne Esters with NFSI: One-Pot Assembly of 5-Fluoro-Cyclopentenones. *Adv. Synth. Catal.* **360**, 3700-3708 (2018).
8. Tang, H.-J. Zhang, X. Zhang, Y.-F. & Feng, C. Visible-Light-Assisted Gold-Catalyzed Fluoroarylation of Allenoates. *Angew. Chem. Int. Ed.* **59**, 5242-5247, (2020).
9. Gaussian 09, Revision C.01, M. J. Frisch, G. W. Trucks, H. B. Schlegel, G. E. Scuseria, M. A. Robb, J. R. Cheeseman, G. Scalmani, V. Barone, G. A. Petersson, H. Nakatsuji, X. Li, M. Caricato, A. V. Marenich, J. Bloino, B. G. Janesko, R. Gomperts, B. Mennucci, H. P. Hratchian, J. V. Ortiz, A. F. Izmaylov, J. L. Sonnenberg, D. Williams-Young, F. Ding, F. Lipparini, F. Egidi, J. Goings, B. Peng, A. Petrone, T. Henderson, D. Ranasinghe, V. G. Zakrzewski, J. Gao, N. Rega, G. Zheng, W. Liang, M. Hada, M. Ehara, K. Toyota, R. Fukuda, J. Hasegawa, M. Ishida, T. Nakajima, Y. Honda, O. Kitao, H. Nakai, T. Vreven, K. Throssell, J. A. Montgomery, Jr., J. E. Peralta, F. Ogliaro, M. J. Bearpark, J. J. Heyd, E. N. Brothers, K. N. Kudin, V. N. Staroverov, T. A. Keith, R. Kobayashi, J. Normand, K. Raghavachari, A. P. Rendell, J. C. Burant, S. S. Iyengar, J. Tomasi, M. Cossi, J. M. Millam, M. Klene, C. Adamo, R. Cammi, J. W. Ochterski, R. L. Martin, K. Morokuma, O. Farkas, J. B. Foresman, and D. J. Fox Gaussian, Inc., Wallingford CT, **2013**
10. Marenich, A. V. Cramer, C. J. & Truhlar, D. G. Universal Solvation Model Based on Solute Electron Density and on a Continuum Model of the Solvent Defined by the Bulk Dielectric Constant and Atomic Surface Tensions. *J. Phys. Chem. B.* **113**, 6378-6396 (2009).
11. CYLview, 1.0b; Legault, C. Y. Universitéde Sherbrooke, **2009** (http://www.cylview.org).
