# Supplementary material for: Azoarene activation for Schmidt-type reaction and mechanistic insights
Source: Nat Commun. 2022 Dec 1;13:7393. doi: 10.1038/s41467-022-35141-4 (PMC9712421; doi:10.1038/s41467-022-35141-4)
Supplement: Supplementary file 2 — Description of Additional Supplementary Files [file 41467_2022_35141_MOESM2_ESM.docx]

**Description of Additional Supplementary Files**

File Name: Supplementary Data 1

Description: Cartessian coordinates of DFT optimized structures
