# Supplementary material for: Azoarene activation for Schmidt-type reaction and mechanistic insights
Source: Nat Commun. 2022 Dec 1;13:7393. doi: 10.1038/s41467-022-35141-4 (PMC9712421; doi:10.1038/s41467-022-35141-4)
Supplement: Supplementary file 3 — Supplementary Data 1 [file 41467_2022_35141_MOESM3_ESM.docx]

**Supplementary Data 1**

**Cartesian coordinates and energies of the optimized structures:**

The Cartesian coordinates of optimized structures are given below in the standard format, and units are in Å.

**1g**

Cartesian coordinates

C 0.922426 -1.119428 1.457066

C 0.375952 -0.074954 0.468450

C 1.180595 -0.759853 -0.671601

C 1.408526 -1.974882 0.262427

C 2.584800 -0.221560 -0.991647

C 3.510111 -1.297316 -0.505882

C 2.874120 -2.255389 0.181639

H 1.758767 -0.693115 2.028370

H 0.198527 -1.556680 2.157256

H 2.707635 -0.000384 -2.063892

H 2.805762 0.731491 -0.478201

H 4.590007 -1.259239 -0.674710

H 3.354148 -3.113222 0.660268

C -1.064139 -0.153166 0.275336

C -2.270600 -0.256574 0.128065

C -3.675671 -0.414130 -0.074689

C -4.132828 -1.775741 -0.504039

H -3.642533 -2.082315 -1.440048

H -3.870596 -2.537980 0.244940

H -5.218628 -1.802328 -0.657868

O 0.770642 1.259594 0.843658

C 0.661885 2.215622 -0.105549

O 0.255352 1.992751 -1.221282

C 1.117700 3.533537 0.419624

H 0.519839 3.821157 1.294300

H 2.160135 3.467752 0.758936

H 1.032429 4.303599 -0.352621

C -4.524101 0.617293 0.108797

H -5.597554 0.491266 -0.053385

H -4.172665 1.603686 0.420021

H 0.590751 -0.927297 -1.582481

H 0.763366 -2.845898 0.072132

**Cat**

Cartesian coordinates

C -0.529515 2.233261 -0.191709

C 0.759450 1.644483 -0.178708

C 1.883288 2.482088 -0.302640

C 1.760096 3.859940 -0.437334

C 0.493398 4.438247 -0.439100

C -0.629952 3.628222 -0.315438

C -1.809144 1.480140 -0.086866

C -2.364442 0.853907 -1.214851

C -3.573929 0.163913 -1.120017

C -4.244648 0.089135 0.102518

C -3.710025 0.724773 1.222737

C -2.506047 1.423925 1.126899

H 2.885221 2.051460 -0.290369

H 2.655757 4.476917 -0.534279

H 0.377700 5.519793 -0.538510

H -1.626510 4.077389 -0.321216

H -1.841093 0.913264 -2.173808

H -3.995803 -0.313500 -2.008090

H -5.189933 -0.453611 0.177200

H -4.236736 0.683278 2.179324

H -2.093607 1.924940 2.006960

P 1.086133 -0.143804 0.076389

C 2.339603 -0.730378 -1.230718

C 1.952668 -0.046797 -2.541009

C 3.800779 -0.450648 -0.885274

C 2.171468 -2.242805 -1.396397

H 0.886630 -0.188358 -2.780387

H 2.160135 1.031774 -2.536607

H 2.533646 -0.492654 -3.362504

H 4.131156 -0.999511 0.006728

H 4.423713 -0.800734 -1.722650

H 4.035173 0.611806 -0.745266

H 2.916829 -2.602751 -2.122113

H 2.331367 -2.799003 -0.462289

H 1.176950 -2.507121 -1.785488

C 1.675688 -0.305345 1.861786

C 2.806056 0.661865 2.200830

C 0.466282 0.022172 2.736269

C 2.110001 -1.747446 2.102884

H 3.696220 0.532166 1.572721

H 2.487882 1.711630 2.142554

H 3.116696 0.480749 3.241413

H -0.364522 -0.682718 2.576131

H 0.759955 -0.040420 3.795296

H 0.092146 1.042735 2.563139

H 2.315167 -1.887845 3.175203

H 1.325134 -2.467101 1.821664

H 3.029699 -2.009744 1.560960

Au -0.777056 -1.439895 -0.160313

**M1**

Cartesian coordinates

C 2.279262 1.445986 -1.459142

C 2.996410 0.497860 -0.492350

C 3.850925 1.692025 0.027805

C 2.890066 2.656339 -0.711499

C 5.232795 1.914204 -0.609800

C 5.055840 3.148029 -1.442896

C 3.781855 3.556808 -1.503188

H 2.686854 1.332629 -2.473070

H 1.183435 1.354732 -1.504362

H 6.023297 2.017416 0.150108

H 5.555078 1.068224 -1.242588

H 5.891288 3.623950 -1.963514

H 3.419492 4.410148 -2.082136

H 2.160928 3.178409 -0.072973

C 2.165249 -0.089756 0.574610

C 1.826211 -0.678505 1.615501

C 1.677457 -1.436707 2.824148

C 0.845276 -0.855353 3.921153

H 1.140453 0.180387 4.144517

H -0.218309 -0.824409 3.636263

H 0.931755 -1.448557 4.839141

O 3.691132 -0.544181 -1.191648

C 4.620782 -1.240490 -0.492849

O 4.828137 -1.054900 0.682463

C 5.354310 -2.194965 -1.369601

H 4.782002 -2.480118 -2.258841

H 6.279664 -1.706959 -1.709293

H 5.646103 -3.083080 -0.798454

C -2.621155 -1.449600 -1.714274

C -3.210296 -0.348706 -1.048558

C -4.513432 0.039469 -1.413833

C -5.223567 -0.623017 -2.408962

C -4.636389 -1.698265 -3.071199

C -3.352143 -2.099237 -2.722357

C -1.259619 -1.985145 -1.440563

C -0.189559 -1.646951 -2.282173

C 1.079724 -2.183173 -2.066923

C 1.296641 -3.074133 -1.014924

C 0.232892 -3.438688 -0.189351

C -1.036021 -2.897029 -0.398796

H -4.986783 0.885654 -0.914545

H -6.232370 -0.293537 -2.666911

H -5.177621 -2.226358 -3.859598

H -2.888407 -2.944592 -3.237330

H -0.355959 -0.952289 -3.111325

H 1.904449 -1.901416 -2.725368

H 2.293088 -3.491379 -0.843800

H 0.388248 -4.150995 0.625372

H -1.868024 -3.191522 0.246683

P -2.348039 0.651737 0.226213

C -2.406312 2.464996 -0.351842

C -2.142986 2.454406 -1.856977

C -3.707307 3.201948 -0.047721

C -1.260684 3.208637 0.339400

H -1.250450 1.864312 -2.120858

H -2.989971 2.060620 -2.435526

H -1.962993 3.485372 -2.198872

H -3.890973 3.298296 1.030939

H -3.628981 4.224148 -0.449705

H -4.593061 2.751517 -0.513536

H -1.296109 4.270221 0.049954

H -1.319833 3.168318 1.435762

H -0.275231 2.820789 0.041023

C -3.259410 0.333421 1.849677

C -4.770280 0.524332 1.774761

C -2.970857 -1.127663 2.197640

C -2.661408 1.248056 2.915844

H -5.068210 1.529328 1.451363

H -5.251366 -0.211112 1.116015

H -5.191680 0.371156 2.780616

H -1.891667 -1.332600 2.288476

H -3.440068 -1.371894 3.163337

H -3.385520 -1.819292 1.448537

H -3.033520 0.944695 3.906757

H -1.561018 1.191679 2.945225

H -2.945054 2.300410 2.772441

Au -0.091953 0.106182 0.629477

C 2.316064 -2.620169 2.907959

H 2.265081 -3.212156 3.825168

H 2.911456 -3.013894 2.081341

H 3.889218 1.758180 1.122858

**TS1**

Cartesian coordinates

C 2.127847 1.554764 -1.500543

C 2.816103 0.721589 -0.414035

C 3.340902 2.038464 0.249199

C 2.858585 2.814061 -1.001184

C 4.849093 2.243809 0.371042

C 5.214798 2.855289 -0.948518

C 4.141608 3.164385 -1.689107

H 2.337722 1.232635 -2.529970

H 1.039217 1.588533 -1.353682

H 5.096290 2.916955 1.209982

H 5.406674 1.317585 0.590448

H 6.251579 3.039924 -1.243118

H 4.174606 3.632691 -2.676805

C 2.022636 -0.167221 0.490359

C 2.501134 -1.092437 1.247311

C 2.362413 -2.107835 2.246974

C 0.961705 -2.235502 2.780635

H 0.592173 -1.277715 3.178548

H 0.264811 -2.543425 1.985229

H 0.925957 -2.984182 3.581370

O 3.888882 -0.009652 -1.073074

C 4.636588 -0.879426 -0.423974

O 4.430157 -1.228374 0.740953

C 5.752056 -1.409713 -1.240974

H 6.360934 -0.581524 -1.626193

H 6.372808 -2.092469 -0.654877

H 5.344779 -1.939414 -2.113080

C -2.741649 -1.201976 -1.788481

C -3.317301 -0.253457 -0.907618

C -4.657519 0.124370 -1.114026

C -5.420259 -0.399910 -2.150909

C -4.847772 -1.321466 -3.024048

C -3.525838 -1.709368 -2.838305

C -1.347679 -1.723335 -1.707389

C -0.365654 -1.245211 -2.588724

C 0.923145 -1.780353 -2.579019

C 1.247842 -2.811658 -1.698015

C 0.275025 -3.305725 -0.828194

C -1.011211 -2.764975 -0.829657

H -5.119593 0.858364 -0.454057

H -6.457658 -0.082248 -2.276518

H -5.428827 -1.740799 -3.848595

H -3.074302 -2.435879 -3.519046

H -0.617871 -0.442315 -3.288488

H 1.675665 -1.393151 -3.271150

H 2.258312 -3.230644 -1.687688

H 0.517391 -4.122113 -0.142444

H -1.769775 -3.163642 -0.149456

P -2.394539 0.581418 0.445820

C -2.530294 2.455345 0.117210

C -2.332235 2.640270 -1.387496

C -3.834378 3.108709 0.563363

C -1.373279 3.146672 0.845253

H -1.432957 2.118500 -1.753935

H -3.190667 2.288756 -1.976499

H -2.198489 3.710970 -1.606477

H -3.973956 3.064694 1.652300

H -3.801939 4.175476 0.290968

H -4.727276 2.692023 0.080006

H -1.444503 4.232738 0.677451

H -1.386735 2.985165 1.931961

H -0.391478 2.820128 0.468647

C -3.212233 0.021284 2.056143

C -4.728019 0.181795 2.107603

C -2.881003 -1.465057 2.181365

C -2.563507 0.794716 3.200649

H -5.070003 1.213089 1.954685

H -5.240182 -0.466505 1.384001

H -5.076731 -0.125907 3.106171

H -1.796406 -1.649459 2.190935

H -3.294750 -1.854556 3.124687

H -3.323902 -2.053875 1.363611

H -2.902497 0.379818 4.162538

H -1.464080 0.722825 3.179496

H -2.837812 1.859152 3.192982

Au -0.100949 0.081291 0.553190

C 3.361407 -2.889756 2.700964

H 3.149502 -3.627012 3.477785

H 4.383124 -2.817294 2.334590

H 2.783867 2.237676 1.174584

H 2.209381 3.690493 -0.840329

**M2**

Cartesian coordinates

C 2.263273 1.135545 -1.830535

C 2.824971 0.640001 -0.490938

C 2.882871 2.109328 0.021629

C 2.533159 2.592813 -1.407514

C 4.238322 2.705011 0.400087

C 4.736062 3.264666 -0.899897

C 3.817452 3.205765 -1.874216

H 2.797002 0.763995 -2.716782

H 1.197107 0.889219 -1.933243

H 4.134595 3.493102 1.165585

H 4.927898 1.966893 0.844820

H 5.737660 3.689245 -1.011418

H 3.962109 3.570421 -2.895060

C 2.109079 -0.356404 0.346415

C 2.807218 -1.330020 0.957747

C 2.416386 -2.398470 1.875017

C 1.005723 -2.406511 2.363994

H 0.733134 -1.450744 2.839079

H 0.294096 -2.559394 1.537643

H 0.851786 -3.209385 3.095625

O 4.248180 0.202902 -0.807884

C 4.834386 -0.715050 -0.154444

O 4.249130 -1.430725 0.726163

C 6.242986 -0.997372 -0.450163

H 6.746538 -0.101624 -0.826393

H 6.748506 -1.398790 0.433699

H 6.278345 -1.767445 -1.235513

C -3.030973 -1.196794 -1.549867

C -3.403113 -0.135867 -0.690790

C -4.724104 0.345882 -0.758778

C -5.660533 -0.190609 -1.635459

C -5.292803 -1.240766 -2.472991

C -3.992817 -1.730462 -2.424154

C -1.671546 -1.801998 -1.630487

C -0.753750 -1.337298 -2.584535

C 0.488324 -1.953198 -2.736147

C 0.831204 -3.045500 -1.938799

C -0.079200 -3.522867 -0.995928

C -1.323264 -2.909051 -0.844293

H -5.032798 1.166773 -0.111191

H -6.675223 0.212968 -1.660171

H -6.015085 -1.676894 -3.166990

H -3.698267 -2.552078 -3.082614

H -1.021172 -0.483636 -3.215141

H 1.190437 -1.577983 -3.485265

H 1.804161 -3.529850 -2.055820

H 0.179488 -4.382638 -0.372018

H -2.035495 -3.294482 -0.108810

P -2.246975 0.660288 0.498528

C -2.298309 2.531147 0.131103

C -2.267082 2.669759 -1.390792

C -3.486391 3.293711 0.708152

C -1.014805 3.147524 0.696140

H -1.451905 2.079710 -1.841744

H -3.208819 2.360958 -1.865952

H -2.092900 3.723885 -1.658163

H -3.501451 3.275160 1.806618

H -3.402026 4.350851 0.409931

H -4.460780 2.946528 0.341119

H -1.038207 4.237683 0.540052

H -0.889960 2.974216 1.774334

H -0.118760 2.760526 0.188749

C -2.925846 0.214166 2.210228

C -4.412040 0.480573 2.420929

C -2.679886 -1.287628 2.355066

C -2.105931 0.970148 3.253364

H -4.700079 1.526804 2.259609

H -5.045112 -0.155836 1.787891

H -4.666581 0.235785 3.464605

H -1.614116 -1.541983 2.256606

H -3.014048 -1.621885 3.350001

H -3.243404 -1.871408 1.610917

H -2.361173 0.599562 4.258499

H -1.021919 0.826251 3.114023

H -2.312008 2.050269 3.246795

Au 0.031954 0.012119 0.435080

C 3.287467 -3.346742 2.278546

H 2.961848 -4.125971 2.971198

H 4.324556 -3.384726 1.945321

H 2.103627 2.255524 0.782645

H 1.671245 3.270676 -1.524662

**TS2**

Cartesian coordinates

C -2.020327 1.500296 1.571737

C -2.543448 0.914517 0.278808

C -2.982864 2.288676 -0.218855

C -2.596555 2.874968 1.163495

C -4.459796 2.606965 -0.470788

C -4.919059 3.126345 0.858390

C -3.919837 3.272107 1.739026

H -2.413924 1.036888 2.487046

H -0.921452 1.460101 1.617707

H -4.571257 3.368590 -1.261026

H -5.036936 1.737870 -0.825726

H -5.965700 3.373456 1.056016

H -4.033524 3.648628 2.759116

H -1.857557 3.692494 1.177488

C -2.050331 -0.144930 -0.532283

C -2.799406 -1.151316 -1.031095

C -2.416310 -2.209817 -1.969469

C -1.067615 -2.131787 -2.605285

H -0.864734 -1.135566 -3.028214

H -0.268858 -2.333263 -1.873388

H -0.970776 -2.872514 -3.408727

O -4.195809 0.179959 1.019866

C -4.697883 -0.769026 0.413628

O -4.147456 -1.350258 -0.631151

C -5.991799 -1.365104 0.807531

H -6.508766 -0.726730 1.529075

H -6.616326 -1.528015 -0.079263

H -5.811032 -2.350929 1.259080

C 2.638002 -1.176569 1.851286

C 3.257991 -0.293003 0.932344

C 4.596222 0.078146 1.163607

C 5.310905 -0.379670 2.264382

C 4.689616 -1.223283 3.181614

C 3.371627 -1.610330 2.969217

C 1.254319 -1.715402 1.735623

C 0.251182 -1.290290 2.620282

C -1.027828 -1.845848 2.568281

C -1.320492 -2.849331 1.644693

C -0.324516 -3.297206 0.776700

C 0.949896 -2.732194 0.817159

H 5.096470 0.756336 0.472565

H 6.348186 -0.068356 2.406109

H 5.230579 -1.584661 4.059227

H 2.883884 -2.284162 3.678743

H 0.476843 -0.509609 3.353266

H -1.799249 -1.493412 3.258309

H -2.324830 -3.280642 1.597089

H -0.541654 -4.090436 0.056294

H 1.726520 -3.092433 0.136574

P 2.382272 0.507146 -0.475267

C 2.556227 2.390081 -0.191705

C 2.322501 2.623700 1.300945

C 3.883814 3.003917 -0.623363

C 1.432073 3.087129 -0.964222

H 1.401599 2.131015 1.656094

H 3.156059 2.274299 1.926129

H 2.203690 3.702957 1.484491

H 4.046429 2.928990 -1.707445

H 3.869778 4.078013 -0.379394

H 4.756701 2.580627 -0.110518

H 1.532925 4.176463 -0.837657

H 1.453177 2.882632 -2.043568

H 0.437762 2.802802 -0.587242

C 3.233024 -0.104097 -2.048884

C 4.752694 0.022640 -2.063851

C 2.873374 -1.585061 -2.157644

C 2.631408 0.657563 -3.227280

H 5.112874 1.049135 -1.920387

H 5.231460 -0.622464 -1.314888

H 5.121646 -0.311649 -3.046478

H 1.785408 -1.747635 -2.182503

H 3.293469 -1.996368 -3.088876

H 3.291556 -2.171704 -1.325338

H 2.987162 0.214306 -4.170300

H 1.530567 0.608130 -3.234971

H 2.927145 1.716228 -3.235756

Au 0.069286 0.081108 -0.609821

C -3.254069 -3.226173 -2.263203

H -2.953818 -3.993820 -2.980192

H -4.242541 -3.326827 -1.814384

H -2.336957 2.569218 -1.063958

**M3**

Cartesian coordinates

C -2.691207 1.243181 -2.164649

C -3.053135 0.309591 -1.047628

C -4.544720 0.477292 -1.295341

C -4.173637 1.678411 -2.211080

C -5.387972 1.006205 -0.136674

C -5.158093 2.486363 -0.219849

C -4.520633 2.849007 -1.343293

H -1.926879 2.009691 -1.962538

H -2.381777 0.686153 -3.065477

H -6.458880 0.761839 -0.252515

H -5.089371 0.568396 0.830400

H -5.509490 3.182584 0.547309

H -4.277409 3.879998 -1.616726

H -4.622542 1.735625 -3.215680

H -4.971719 -0.400474 -1.804970

C -2.370860 -0.362928 -0.135193

C -2.336281 -1.275524 0.889343

C -2.347462 -0.966491 2.334682

C -1.895306 -2.043296 3.267346

H -2.598731 -2.888582 3.281049

H -0.921972 -2.460250 2.967139

H -1.807426 -1.661401 4.291458

O -1.662430 -2.674224 -1.479627

C -2.007670 -3.264179 -0.495797

O -2.316691 -2.630779 0.698680

C -2.139838 -4.729387 -0.314467

H -1.933085 -5.247321 -1.254905

H -1.442814 -5.076845 0.460289

H -3.149899 -4.978905 0.035609

C 2.717895 2.256405 0.347896

C 3.241138 0.973032 0.062247

C 4.629680 0.840493 -0.124791

C 5.485745 1.933801 -0.051460

C 4.963177 3.201163 0.196130

C 3.595018 3.351868 0.392583

C 1.286942 2.532004 0.638065

C 0.487628 3.224223 -0.281129

C -0.840517 3.528150 0.023195

C -1.381522 3.159376 1.254374

C -0.583366 2.495252 2.187090

C 0.737843 2.176862 1.879224

H 5.056466 -0.136693 -0.351503

H 6.558633 1.793747 -0.200015

H 5.621019 4.072015 0.242324

H 3.181141 4.341442 0.603866

H 0.910687 3.524419 -1.244436

H -1.453023 4.066535 -0.704633

H -2.419394 3.405694 1.494290

H -0.986929 2.233100 3.168100

H 1.361896 1.663759 2.615715

P 2.180629 -0.494652 -0.236673

C 2.578552 -1.072155 -2.009452

C 2.706700 0.192363 -2.858548

C 3.830850 -1.932370 -2.142781

C 1.379842 -1.873679 -2.520527

H 1.845074 0.867211 -2.726422

H 3.620063 0.764026 -2.642983

H 2.736865 -0.089051 -3.922394

H 3.734817 -2.893095 -1.617717

H 3.983886 -2.166068 -3.208264

H 4.748148 -1.438238 -1.796716

H 1.609314 -2.256568 -3.527320

H 1.135013 -2.738077 -1.888379

H 0.472709 -1.256056 -2.601532

C 2.607146 -1.770124 1.099903

C 4.094450 -2.073216 1.249529

C 2.106557 -1.170780 2.415119

C 1.836167 -3.051037 0.789898

H 4.562856 -2.445797 0.330134

H 4.662475 -1.201785 1.602314

H 4.216710 -2.857437 2.013109

H 1.030377 -0.934780 2.391108

H 2.272087 -1.891719 3.231171

H 2.650498 -0.251031 2.678237

H 1.937506 -3.752429 1.633006

H 0.759710 -2.860935 0.645350

H 2.215869 -3.563792 -0.105333

Au -0.179539 -0.291951 -0.076460

C -2.795655 0.228734 2.758674

H -2.834292 0.460824 3.825466

H -3.135769 1.002514 2.065218

**TS3**

Cartesian coordinates

C -2.562816 2.937404 -0.535755

C -3.607746 2.066694 1.177293

C -2.829183 3.387777 0.916013

C -3.051276 1.445232 2.461757

C -1.790661 2.227715 2.687387

C -1.676102 3.278731 1.862974

H -1.555263 3.042648 -0.966004

H -3.285206 3.390375 -1.230392

H -3.753759 1.566297 3.304381

H -2.880072 0.359902 2.370071

H -1.070103 1.976063 3.470335

H -0.853069 3.998809 1.877234

H -3.379293 4.337321 1.013336

H -4.699230 2.159230 1.156330

C 2.822090 -1.542900 1.297216

C 3.291815 -0.530686 0.427203

C 4.679619 -0.411092 0.227258

C 5.586685 -1.278237 0.826520

C 5.116678 -2.312874 1.631718

C 3.751290 -2.434334 1.861579

C 1.412176 -1.707449 1.736169

C 0.833293 -0.763837 2.599478

C -0.426090 -0.987906 3.153377

C -1.140030 -2.142577 2.831798

C -0.581047 -3.077512 1.961848

C 0.690230 -2.868690 1.427628

H 5.065993 0.371997 -0.425861

H 6.657188 -1.151015 0.650794

H 5.813211 -3.015642 2.094841

H 3.380013 -3.221867 2.523071

H 1.397451 0.136268 2.863163

H -0.847413 -0.262412 3.852881

H -2.129296 -2.314464 3.264312

H -1.135822 -3.982345 1.702118

H 1.136011 -3.617480 0.765700

P 2.168056 0.528764 -0.567660

C 2.677362 2.339915 -0.280963

C 2.908436 2.489300 1.222252

C 3.904165 2.808992 -1.055349

C 1.488652 3.220253 -0.675778

H 2.042483 2.142446 1.809068

H 3.797046 1.945061 1.572743

H 3.058113 3.553506 1.462904

H 3.749228 2.773957 -2.142545

H 4.104647 3.860762 -0.796444

H 4.818001 2.249862 -0.816515

H 1.754639 4.278542 -0.524230

H 1.199919 3.103836 -1.729894

H 0.603355 3.009143 -0.056994

C 2.444016 -0.060821 -2.354747

C 3.897885 -0.152976 -2.804671

C 1.828108 -1.460549 -2.415342

C 1.673927 0.876679 -3.282181

H 4.452460 0.787839 -2.695565

H 4.450043 -0.944419 -2.280450

H 3.916396 -0.413957 -3.874752

H 0.748952 -1.452607 -2.190377

H 1.954766 -1.872901 -3.428802

H 2.315390 -2.159624 -1.717303

H 1.668965 0.459880 -4.301503

H 0.623577 0.996832 -2.970412

H 2.131309 1.874689 -3.345164

Au -0.196652 0.367464 -0.296514

C -2.988368 1.554834 -0.137186

C -4.771936 0.746143 -1.213372

C -3.062472 -0.760388 -0.553706

C -4.461029 -0.528836 -0.781250

H -5.784670 1.145144 -1.095748

H -4.166043 1.231778 -1.978024

C -5.487936 -1.542512 -0.429715

H -5.117522 -2.289941 0.283873

H -6.378366 -1.054669 -0.010650

H -5.824561 -2.079359 -1.329655

O -2.547785 -2.030196 -0.506199

C -2.832501 -2.886466 -1.546394

O -3.479200 -2.541364 -2.499399

C -2.223223 -4.216618 -1.306772

H -1.133314 -4.120169 -1.213460

H -2.590318 -4.638486 -0.362249

H -2.460157 -4.894738 -2.131110

C -2.274864 0.345133 -0.342747

**M4**

Cartesian coordinates

C -3.667294 1.979874 -0.802998

C -3.066119 2.319020 1.282519

C -3.311430 3.210830 0.041978

C -1.659149 2.629657 1.799407

C -1.089146 3.525863 0.743036

C -1.978252 3.836463 -0.211797

H -3.036260 1.813877 -1.687433

H -4.718740 1.890924 -1.107371

H -1.680587 3.127937 2.784614

H -1.047161 1.721801 1.944468

H -0.056759 3.886495 0.768780

H -1.772893 4.473035 -1.077508

H -4.124350 3.953011 0.107123

H -3.830228 2.409881 2.067025

C 2.583734 -0.842755 1.979843

C 3.116694 -0.309609 0.780697

C 4.506944 -0.104633 0.701401

C 5.357266 -0.388175 1.764109

C 4.826570 -0.884248 2.952316

C 3.457707 -1.106038 3.048767

C 1.150804 -1.176822 2.215742

C 0.354392 -0.355599 3.026238

C -0.964334 -0.707206 3.319877

C -1.503637 -1.891428 2.817555

C -0.714304 -2.725263 2.024211

C 0.601908 -2.372780 1.728652

H 4.939712 0.297588 -0.214762

H 6.430757 -0.214186 1.662614

H 5.476399 -1.104513 3.802403

H 3.038412 -1.507895 3.975065

H 0.773402 0.573025 3.426390

H -1.572050 -0.049571 3.948114

H -2.533989 -2.170996 3.054069

H -1.123810 -3.660895 1.634554

H 1.220314 -3.043322 1.127589

P 2.087595 0.217118 -0.652225

C 2.609339 1.999338 -1.076230

C 2.719646 2.726830 0.264184

C 3.891439 2.168356 -1.885143

C 1.462683 2.619660 -1.880001

H 1.856532 2.518768 0.918470

H 3.629043 2.456528 0.818959

H 2.747525 3.814272 0.093014

H 3.813589 1.723141 -2.886629

H 4.070061 3.245504 -2.032272

H 4.789209 1.769131 -1.396701

H 1.708133 3.669338 -2.107322

H 1.288317 2.112549 -2.839109

H 0.515066 2.614473 -1.321594

C 2.476276 -1.016168 -2.033408

C 3.962215 -1.231643 -2.296931

C 1.869424 -2.340615 -1.575056

C 1.773247 -0.550348 -3.305630

H 4.490478 -0.320410 -2.605028

H 4.481972 -1.656191 -1.426848

H 4.074031 -1.959047 -3.116746

H 0.798625 -2.242712 -1.334616

H 1.969977 -3.090416 -2.375638

H 2.385932 -2.739008 -0.689314

H 1.840483 -1.342134 -4.068497

H 0.702494 -0.347269 -3.141129

H 2.232734 0.350241 -3.736981

Au -0.292329 0.229987 -0.293561

C -3.344547 1.045730 0.439080

C -4.530027 0.181492 0.882917

C -3.004009 -1.127007 -0.260147

C -4.282265 -1.132535 0.258355

H -4.507721 0.066434 1.982146

H -5.528065 0.586188 0.653085

C -5.231723 -2.248835 0.252455

H -4.842370 -3.136533 -0.257720

H -5.510703 -2.518576 1.283319

H -6.173892 -1.940132 -0.227036

O -2.454926 -2.249890 -0.831707

C -2.018852 -2.157869 -2.128834

O -2.115823 -1.145408 -2.772294

C -1.493268 -3.466248 -2.594670

H -0.983135 -4.011510 -1.792248

H -2.342706 -4.085897 -2.918022

H -0.829285 -3.326390 -3.453661

C -2.314003 0.081063 -0.010985

**TS4**

Cartesian coordinates

C -3.335364 -1.439471 -1.009977

C -3.033298 0.138611 -2.664667

C -3.123212 -1.398536 -2.530409

C -1.665650 0.458730 -3.292098

C -1.039729 -0.886375 -3.482293

C -1.812405 -1.890580 -3.050872

H -2.788283 -2.191482 -0.427855

H -4.382672 -1.483311 -0.677543

H -1.762229 1.011891 -4.240673

H -1.045385 1.095783 -2.635963

H -0.044707 -1.011715 -3.918575

H -1.546381 -2.950715 -3.068568

H -3.985724 -1.863672 -3.033732

H -3.869176 0.589087 -3.214905

C 2.471368 2.234435 0.001921

C 3.057616 0.945581 0.056197

C 4.460378 0.849796 -0.009627

C 5.273092 1.968584 -0.142905

C 4.691425 3.232673 -0.225835

C 3.308870 3.352462 -0.153204

C 1.015490 2.527026 0.114750

C 0.283544 2.934171 -1.008778

C -1.062905 3.284415 -0.891685

C -1.692732 3.245583 0.351451

C -0.969624 2.857890 1.479313

C 0.372900 2.502837 1.362106

H 4.935465 -0.129692 0.034340

H 6.357773 1.850038 -0.189321

H 5.311797 4.123214 -0.341415

H 2.847809 4.342489 -0.205668

H 0.774404 2.970984 -1.987624

H -1.621495 3.593029 -1.781365

H -2.743642 3.531164 0.445428

H -1.455199 2.830353 2.458670

H 0.938058 2.219390 2.252411

P 2.085462 -0.619826 0.113793

C 2.733273 -1.703652 -1.315071

C 2.869906 -0.770219 -2.518057

C 4.039802 -2.453014 -1.070524

C 1.651080 -2.741167 -1.624484

H 1.975825 -0.139283 -2.655740

H 3.739637 -0.102504 -2.441608

H 2.993474 -1.368318 -3.435471

H 3.950322 -3.193542 -0.263505

H 4.295439 -3.011663 -1.984944

H 4.898627 -1.805646 -0.852938

H 1.990290 -3.375842 -2.458478

H 1.435981 -3.407146 -0.777689

H 0.705666 -2.269255 -1.929451

C 2.429210 -1.369298 1.818253

C 3.903969 -1.472002 2.191138

C 1.742854 -0.442647 2.820503

C 1.771798 -2.747036 1.869520

H 4.492685 -2.079998 1.493921

H 4.380366 -0.486633 2.278934

H 3.984579 -1.950298 3.180861

H 0.672052 -0.311555 2.596833

H 1.829342 -0.865405 3.834076

H 2.212837 0.552078 2.842297

H 1.806856 -3.131055 2.901688

H 0.712694 -2.712618 1.563531

H 2.289664 -3.482317 1.237359

Au -0.262223 -0.414280 -0.141950

C -3.217639 0.350655 -1.182597

C -4.356269 1.094042 -0.567255

C -3.054202 0.169417 1.077286

C -4.185245 0.890197 0.900024

H -4.216902 2.155036 -0.856572

H -5.348316 0.822697 -0.962091

C -5.089379 1.440743 1.929921

H -4.790081 1.135350 2.940318

H -5.103299 2.542224 1.899007

H -6.130092 1.121683 1.769066

O -2.536108 -0.112030 2.323658

C -2.154731 -1.389212 2.605039

O -2.303395 -2.304471 1.832959

C -1.609869 -1.492262 3.985274

H -1.165795 -0.552271 4.332233

H -2.442291 -1.738806 4.665526

H -0.882037 -2.312650 4.044600

C -2.350695 -0.149305 -0.184889

**M5**

Cartesian coordinates

C -0.566890 -1.576041 0.277488

C -2.356963 0.110660 0.843846

C -2.098284 -1.388194 0.450641

C -3.552265 0.580563 -0.013982

C -3.647762 -0.459813 -1.083253

C -2.877763 -1.523450 -0.824235

H -0.319170 -2.195500 -0.601706

H -0.112707 -2.089187 1.141121

H -4.487301 0.626394 0.572558

H -3.410808 1.599470 -0.413055

H -4.305535 -0.361362 -1.951606

H -2.814107 -2.417821 -1.451795

H -2.484769 -2.095227 1.206219

H -2.578961 0.233127 1.919202

C -1.047306 0.733077 0.486422

C -0.496777 2.113469 0.369150

C 1.124094 0.554601 -0.160879

C 0.929031 1.895565 -0.062367

H -1.051873 2.733010 -0.358587

H -0.550684 2.680517 1.315936

C 1.898576 2.981247 -0.334006

H 2.874780 2.583848 -0.641891

H 1.550920 3.658355 -1.131567

H 2.064124 3.618750 0.549810

O 2.294096 -0.017744 -0.623689

C 2.868982 -1.007641 0.112552

O 2.459634 -1.339181 1.196599

C 4.026403 -1.592599 -0.616269

H 3.660988 -2.164494 -1.480525

H 4.681473 -0.806569 -1.011510

H 4.593289 -2.260125 0.039021

C -0.092994 -0.169408 0.174075

**TS5**

Cartesian coordinates

C -2.934084 -1.480386 -2.543258

C -3.128776 0.736338 -1.847997

C -2.797642 -0.069084 -3.125048

C -2.146228 1.913331 -1.800833

C -1.097382 1.518411 -2.795419

C -1.439695 0.436199 -3.505093

H -1.996535 -2.054160 -2.523330

H -3.711802 -2.089359 -3.017544

H -2.684706 2.822420 -2.129959

H -1.752752 2.147387 -0.802206

H -0.177904 2.094043 -2.931038

H -0.843754 -0.019146 -4.299987

H -3.535070 0.120713 -3.924419

H -4.171453 1.043426 -1.693169

C 3.073474 1.680389 0.191875

C 3.244703 0.274152 0.250125

C 4.556788 -0.234931 0.298863

C 5.677250 0.586890 0.285275

C 5.508788 1.966202 0.203345

C 4.224584 2.492220 0.156101

C 1.785530 2.419699 0.186167

C 1.570432 3.404633 -0.792188

C 0.466650 4.252711 -0.730271

C -0.439001 4.142679 0.324014

C -0.250043 3.158076 1.294052

C 0.845919 2.298120 1.223215

H 4.716375 -1.311332 0.326223

H 6.675631 0.146053 0.327173

H 6.372939 2.634162 0.183846

H 4.089898 3.576098 0.114964

H 2.290936 3.510389 -1.608611

H 0.321589 5.010690 -1.504296

H -1.296715 4.816984 0.386357

H -0.959872 3.064575 2.118974

H 1.003041 1.550609 2.006366

P 1.890141 -0.960682 0.080312

C 2.341244 -1.956868 -1.495243

C 2.811239 -0.933497 -2.529945

C 3.390017 -3.049929 -1.317269

C 1.059445 -2.608284 -2.023148

H 2.104087 -0.093770 -2.632301

H 3.801171 -0.516363 -2.298207

H 2.882198 -1.419913 -3.515532

H 3.055843 -3.843439 -0.634548

H 3.565842 -3.526662 -2.294705

H 4.365016 -2.682886 -0.973174

H 1.307514 -3.228956 -2.898839

H 0.568522 -3.263201 -1.288838

H 0.327560 -1.855398 -2.351618

C 1.929390 -2.018503 1.658604

C 3.300343 -2.529984 2.086628

C 1.381373 -1.119524 2.767185

C 0.978461 -3.196259 1.452070

H 3.811073 -3.119939 1.314649

H 3.969311 -1.719142 2.404960

H 3.167793 -3.189343 2.958924

H 0.357521 -0.781616 2.547133

H 1.347418 -1.683713 3.712813

H 2.016089 -0.235810 2.936621

H 0.851043 -3.731707 2.405943

H -0.021489 -2.868961 1.123859

H 1.360435 -3.923992 0.721869

Au -0.396876 -0.351212 -0.186182

C -3.338152 -0.985246 -1.189008

C -4.679285 -1.180892 -0.561018

C -3.342711 0.071274 0.818225

C -4.595050 -0.433509 0.728045

H -5.530041 -0.910981 -1.207605

H -4.790466 -2.267623 -0.386664

C -5.689804 -0.341277 1.714631

H -5.438390 0.330236 2.545334

H -6.622220 0.016396 1.252959

H -5.920763 -1.330971 2.139893

O -2.856231 0.798204 1.885940

C -2.321417 0.078368 2.921741

O -2.319479 -1.125831 2.940393

C -1.749103 0.977324 3.957206

H -0.744836 1.293313 3.637795

H -2.350975 1.883737 4.089066

H -1.649335 0.442293 4.907030

C -2.484427 -0.278531 -0.320482

**M6**

Cartesian coordinates

C -2.637288 1.446909 -0.215091

C -1.537627 -0.726155 -0.889208

C -2.900353 -0.061948 -0.480185

C -1.424686 -2.031482 -0.073193

C -2.476828 -1.882791 0.978986

C -3.276446 -0.832953 0.751978

H -3.168713 1.804737 0.683706

H -3.007821 2.077856 -1.043472

H -1.613049 -2.924900 -0.694555

H -0.416697 -2.180325 0.351061

H -2.582559 -2.580688 1.814441

H -4.129205 -0.548972 1.376360

H -3.677760 -0.183039 -1.255392

H -1.474012 -0.933897 -1.971131

C -1.154322 1.493670 -0.103797

C -0.103098 2.482653 0.267766

C 0.868593 0.497375 -0.400452

C 1.188825 1.741452 0.041290

H -0.150762 3.407743 -0.333985

H -0.193097 2.827123 1.313756

C 2.535424 2.298419 0.302681

H 3.326671 1.679955 -0.143819

H 2.644752 3.318443 -0.097490

H 2.752668 2.365497 1.382249

O 1.743640 -0.502642 -0.780121

C 2.541575 -1.087468 0.159212

O 2.517833 -0.790189 1.325251

C 3.404460 -2.118641 -0.479334

H 2.786425 -2.866017 -0.993809

H 4.041370 -1.655752 -1.244741

H 4.031480 -2.611549 0.268854

C -0.571808 0.338699 -0.489498

**2h**

Cartesian coordinates

C 0.595873 1.228685 0.000014

C -0.783109 1.241689 -0.000003

C -1.451838 -0.000001 0.000003

C -0.783108 -1.241690 -0.000001

C 0.595874 -1.228686 0.000016

C 1.273690 0.000000 0.000031

H 1.153201 2.165486 0.000013

H -1.347255 2.173849 -0.000011

H -1.347255 -2.173850 -0.000006

H 1.153201 -2.165487 0.000016

Cl 2.990201 0.000001 0.000007

N -3.924971 0.000004 -0.000057

N -2.807830 -0.000004 -0.000012

**TS6**

Cartesian coordinates

C 0.017112 0.360330 -2.026400

C -0.084752 2.760430 -1.268874

C 0.733564 1.735162 -2.124319

C 0.926211 3.499328 -0.356038

C 2.189864 2.724259 -0.541401

C 2.089112 1.783259 -1.486915

H 0.707503 -0.466103 -1.789157

H -0.472560 0.079321 -2.969091

H 1.047850 4.553381 -0.655233

H 0.597701 3.532598 0.697363

H 3.093641 2.937474 0.036224

H 2.895646 1.108319 -1.789529

H 0.797732 2.039869 -3.182009

H -0.645709 3.485466 -1.883942

C -1.045280 1.895283 -0.552161

C -2.038766 2.114216 0.516583

C -1.911990 -0.136298 -0.145166

C -2.489327 0.706370 0.869896

H -1.660035 2.712992 1.359862

H -2.903431 2.683800 0.132820

C -3.889388 0.452528 1.324697

H -4.020051 -0.582856 1.664982

H -4.146057 1.121500 2.155993

H -4.603333 0.632805 0.508281

O -2.308224 -1.408903 -0.207247

C -2.204219 -2.184912 -1.378631

O -2.011692 -1.676512 -2.440764

C -2.418952 -3.607044 -1.044343

H -1.617426 -3.961811 -0.381432

H -3.360095 -3.727982 -0.492705

H -2.436719 -4.210939 -1.955071

C -0.990137 0.582012 -0.951578

N -0.539163 0.190581 2.716246

N -1.663839 0.504175 2.532256

C 0.538678 -0.321667 1.981595

C 1.774142 0.331672 2.051864

C 0.392168 -1.538208 1.299748

C 2.849271 -0.183455 1.343453

H 1.873205 1.253364 2.627348

C 1.480128 -2.061895 0.614068

H -0.551377 -2.081387 1.346816

C 2.691986 -1.368940 0.621717

H 3.809192 0.333948 1.347740

H 1.389138 -3.008060 0.078635

Cl 4.031116 -1.992368 -0.288240

**M7**

Cartesian coordinates

C 3.495778 -0.163270 -1.231197

C 3.511760 -1.974131 0.501171

C 3.817657 -1.660599 -0.997588

C 2.781644 -3.334829 0.538080

C 2.339353 -3.507275 -0.879916

C 2.898673 -2.614614 -1.705503

H 2.996577 0.019244 -2.193719

H 4.401110 0.461559 -1.232951

H 3.463627 -4.144527 0.845640

H 1.954071 -3.352618 1.265255

H 1.671370 -4.314804 -1.189973

H 2.750415 -2.579970 -2.788045

H 4.866836 -1.867828 -1.265680

H 4.407180 -1.985071 1.149085

C 2.670948 -0.831024 0.903882

C 1.863889 -0.522516 2.092213

C 1.727377 1.126314 0.387031

C 1.022082 0.674253 1.633876

H 1.263177 -1.371443 2.448174

H 2.526528 -0.240614 2.928659

C 0.774876 1.764896 2.645311

H 0.190159 2.583368 2.211301

H 0.217478 1.359734 3.500242

H 1.726222 2.169657 3.013993

O 1.304840 2.232402 -0.164204

C 2.059178 2.927241 -1.183706

O 3.226463 2.736419 -1.283213

C 1.157045 3.837871 -1.901591

H 0.366346 3.259740 -2.398370

H 0.657165 4.505010 -1.187574

H 1.715801 4.421473 -2.637281

C 2.639511 0.165154 -0.057798

N -1.218449 0.826267 1.077105

N -0.256812 0.045824 1.077538

C -2.439313 0.323860 0.586205

C -2.600483 -0.962212 0.042425

C -3.531551 1.197519 0.660289

C -3.842253 -1.366331 -0.418731

H -1.741819 -1.633427 -0.014865

C -4.781817 0.799557 0.204113

H -3.383278 2.192197 1.085914

C -4.924349 -0.480649 -0.332267

H -3.983445 -2.360915 -0.845442

H -5.638784 1.471689 0.259639

Cl -6.481786 -0.990763 -0.904410

**TS7**

Cartesian coordinates

C 4.125183 0.923356 -1.040038

C 4.559470 -0.922164 0.613067

C 5.070561 -0.262527 -0.713346

C 4.601013 -2.448168 0.398138

C 4.721271 -2.576240 -1.087145

C 4.982395 -1.406030 -1.682567

H 3.857047 0.969964 -2.106651

H 4.580426 1.896721 -0.796607

H 5.465099 -2.906521 0.908120

H 3.714042 -2.957847 0.809469

H 4.643302 -3.536824 -1.603086

H 5.144069 -1.269781 -2.755528

H 6.112479 0.090934 -0.633199

H 5.144012 -0.628569 1.502898

C 3.192242 -0.356847 0.734017

C 2.031794 -0.662378 1.595642

C 1.629324 1.136564 0.006268

C 1.026582 0.454359 1.250915

C 0.931883 1.466972 2.393974

H 0.316260 2.331707 2.122542

H 0.488115 0.988366 3.276789

H 1.934582 1.824735 2.661981

O 1.060907 1.979371 -0.706053

C -0.899191 2.334260 -0.922552

O -1.196020 1.843822 -1.923125

C -1.040437 3.533333 -0.123874

H -0.361824 4.286986 -0.548378

H -2.076125 3.880329 -0.242827

H -0.809994 3.383729 0.931959

C 2.961124 0.643581 -0.157193

N -1.156473 0.595225 0.501426

N -0.293963 -0.134298 1.019270

C -2.419215 -0.002857 0.272829

C -2.582848 -1.392774 0.176580

C -3.521784 0.848011 0.130634

C -3.842623 -1.926823 -0.041796

H -1.712202 -2.044791 0.260044

C -4.790298 0.319316 -0.070277

H -3.393156 1.929694 0.209297

C -4.939813 -1.065823 -0.152679

H -3.980949 -3.005033 -0.131851

H -5.656851 0.974182 -0.162956

Cl -6.521052 -1.734984 -0.401093

H 2.290041 -0.694925 2.665426

H 1.613665 -1.653085 1.356102

**M8**

Cartesian coordinates

C -3.516509 -0.605535 1.834857

C -4.368480 -0.574764 -0.531056

C -4.491907 -1.302505 0.850735

C -4.307346 -1.677402 -1.609035

C -4.003617 -2.909824 -0.818743

C -4.110614 -2.711487 0.500621

H -2.955842 -1.319389 2.457211

H -4.039741 0.066706 2.532765

H -5.268361 -1.774257 -2.141774

H -3.559861 -1.466776 -2.391409

H -3.765804 -3.866452 -1.291435

H -3.968218 -3.480115 1.265203

H -5.519007 -1.272842 1.251266

H -5.193430 0.131363 -0.729102

C -3.108605 0.195972 -0.370061

C -2.280670 0.971150 -1.319655

C -1.410971 0.875397 1.046347

C -1.151825 1.561164 -0.466081

H -1.885200 0.328834 -2.122013

H -2.862749 1.759310 -1.822461

C -1.267403 3.056929 -0.248060

H -0.621569 3.439658 0.551352

H -1.083356 3.614423 -1.175510

H -2.298162 3.274626 0.061973

O -0.629735 1.063023 1.935430

C 1.595912 2.698074 0.126882

O 1.861294 2.875590 1.268997

C 1.745324 3.601104 -1.031361

H 1.101817 3.348076 -1.879423

H 1.609493 4.642127 -0.717280

H 2.786883 3.492641 -1.378053

C -2.643508 0.161168 0.903134

N 1.173383 1.250229 -0.301261

N 0.045944 0.890838 -0.751832

C 2.198702 0.264639 -0.225797

C 1.839905 -1.090832 -0.207102

C 3.544335 0.644449 -0.141398

C 2.823075 -2.061887 -0.119940

H 0.787671 -1.374071 -0.241463

C 4.530421 -0.328220 -0.055510

H 3.847374 1.692015 -0.153273

C 4.165063 -1.675462 -0.047189

H 2.552081 -3.117446 -0.091828

H 5.580034 -0.040281 0.003422

Cl 5.392971 -2.889840 0.077562

**TS8**

Cartesian coordinates

C -3.315220 -0.869375 1.866674

C -4.329941 -0.477813 -0.386279

C -4.367485 -1.403339 0.868388

C -4.428013 -1.397527 -1.620317

C -4.075246 -2.738905 -1.062479

C -4.045256 -2.744698 0.275851

H -2.741499 -1.668431 2.357480

H -3.768800 -0.266807 2.667938

H -5.448170 -1.395952 -2.039074

H -3.771655 -1.076395 -2.444804

H -3.910683 -3.613854 -1.696201

H -3.848657 -3.622077 0.897450

H -5.359873 -1.424874 1.348834

H -5.123245 0.290849 -0.395979

C -3.026006 0.220406 -0.244139

C -2.320551 1.087849 -1.210828

C -1.232066 0.599943 1.244328

C -1.118665 1.690205 -0.521221

H -2.022963 0.491733 -2.088802

H -3.002279 1.863426 -1.595311

C -1.294515 3.073801 0.029666

H -0.572012 3.347383 0.808214

H -1.243377 3.826546 -0.770985

H -2.295995 3.155925 0.473648

O -0.337315 0.769438 1.976594

C 1.606490 2.731660 0.091070

O 2.103067 2.902525 1.165580

C 1.509090 3.713098 -1.015184

H 0.804248 3.432095 -1.803396

H 1.302105 4.717620 -0.629590

H 2.509057 3.742581 -1.478721

C -2.467450 -0.008373 0.978262

N 1.178275 1.349377 -0.296252

N 0.014229 1.021073 -0.779460

C 2.157858 0.315554 -0.229370

C 1.751001 -1.023432 -0.195769

C 3.521070 0.633469 -0.203475

C 2.698091 -2.037322 -0.140312

H 0.688827 -1.270822 -0.211052

C 4.470075 -0.378513 -0.137208

H 3.861644 1.668791 -0.241965

C 4.054166 -1.709041 -0.106445

H 2.383583 -3.080984 -0.110407

H 5.531968 -0.132029 -0.116984

Cl 5.239392 -2.977615 -0.013233

**M9**

Cartesian coordinates

C -3.065346 -1.915610 1.411488

C -4.300529 -0.155841 0.179229

C -4.355729 -1.634905 0.627487

C -4.890170 -0.105538 -1.245587

C -4.775548 -1.527902 -1.691464

C -4.484539 -2.357606 -0.682223

H -2.635084 -2.904500 1.203001

H -3.209757 -1.843746 2.499013

H -5.943091 0.220493 -1.221631

H -4.377230 0.610337 -1.905630

H -4.968529 -1.835458 -2.721914

H -4.398355 -3.444067 -0.757938

H -5.223685 -1.850001 1.272636

H -4.829693 0.542244 0.855048

C -2.856094 0.206513 0.265721

C -2.316326 1.448791 -0.318367

C -0.839352 -0.782208 1.246435

C -0.941533 1.848353 0.142406

H -2.308164 1.287491 -1.411880

H -3.048893 2.256508 -0.160047

C -0.846982 2.680493 1.367370

H 0.165676 2.699923 1.788604

H -1.164141 3.714946 1.165151

H -1.549411 2.299460 2.123591

O 0.242368 -0.916194 1.588331

C 1.884955 2.952244 -0.312455

O 3.072614 3.115424 -0.104166

C 0.986851 4.072245 -0.735015

H 0.130184 3.765837 -1.346076

H 0.616060 4.624723 0.138989

H 1.600006 4.775389 -1.310250

C -2.169661 -0.782840 0.943136

N 1.329306 1.664775 -0.211917

N 0.016562 1.358313 -0.570203

C 2.194192 0.527016 -0.239427

C 2.013631 -0.469298 -1.204064

C 3.196989 0.387892 0.724017

C 2.828839 -1.596862 -1.209665

H 1.225814 -0.364521 -1.951058

C 4.019542 -0.734475 0.720015

H 3.328280 1.152306 1.488850

C 3.828477 -1.721031 -0.245834

H 2.691507 -2.372996 -1.964173

H 4.799859 -0.848150 1.473756

Cl 4.852218 -3.134016 -0.242103

**TS9**

Cartesian coordinates

C -2.799158 -2.116557 1.251264

C -4.156304 -0.358230 0.142508

C -4.041500 -1.884786 0.377446

C -4.591194 -0.173326 -1.327804

C -4.250386 -1.492919 -1.939503

C -3.949778 -2.419272 -1.021738

H -2.212295 -2.994902 0.950471

H -3.049540 -2.238342 2.314856

H -5.672421 0.031049 -1.393040

H -4.102417 0.680247 -1.821780

H -4.299882 -1.668566 -3.016658

H -3.712600 -3.465183 -1.230395

H -4.924181 -2.300715 0.890726

H -4.857280 0.151864 0.828894

C -2.797345 0.172115 0.459557

C -2.421880 1.575697 0.208012

C -0.773762 -0.659468 1.535908

C -0.979670 1.936638 0.451248

H -2.697578 1.818032 -0.832064

H -3.101483 2.199235 0.815105

C -0.660262 2.778690 1.628737

H 0.415631 2.847398 1.828092

H -1.069482 3.794001 1.511498

H -1.169249 2.362157 2.512199

O 0.264888 -0.639793 2.010846

C 1.715758 2.958314 -0.625614

O 2.919616 3.132455 -0.657603

C 0.730388 4.020290 -1.002173

H -0.112971 3.633745 -1.588396

H 0.324502 4.527071 -0.116250

H 1.263505 4.776223 -1.588172

C -2.040731 -0.818825 1.056095

N 1.193903 1.705037 -0.268063

N -0.167851 1.407229 -0.400392

C 2.032378 0.552054 -0.278768

C 1.670231 -0.581158 -1.016381

C 3.201047 0.535839 0.489030

C 2.470778 -1.719362 -0.992469

H 0.751906 -0.575671 -1.605216

C 4.008492 -0.597033 0.510852

H 3.475851 1.406653 1.082716

C 3.637646 -1.718419 -0.229386

H 2.191399 -2.600960 -1.571818

H 4.918117 -0.612092 1.112720

Cl 4.644329 -3.143535 -0.193599

**M10**

Cartesian coordinates

C -1.858680 -2.752444 -1.562835

C -2.814256 -2.448173 0.725359

C -3.086786 -3.070655 -0.681327

C -4.089646 -1.665565 1.121331

C -4.869584 -1.605698 -0.150258

C -4.339610 -2.366204 -1.114013

H -2.136282 -2.390566 -2.565178

H -1.215236 -3.632508 -1.726746

H -4.653125 -2.180702 1.917546

H -3.866957 -0.663462 1.520958

H -5.780912 -1.010074 -0.248802

H -4.751980 -2.485694 -2.119774

H -3.250560 -4.160005 -0.632335

H -2.558893 -3.197010 1.494739

C -1.635180 -1.566283 0.481820

C -1.070166 -0.621054 1.453124

C -0.077751 -0.892083 -1.267889

C 0.008001 0.242485 0.968589

H -1.831688 0.027946 1.950303

H -0.670099 -1.148400 2.343246

C 0.672220 1.178339 1.882657

H 1.623568 0.729632 2.216321

H 0.928283 2.120841 1.384378

H 0.054988 1.364148 2.765765

O 0.441718 -0.896621 -2.355136

C -1.140160 -1.712426 -0.770103

N 1.546528 0.833896 -0.697312

N 0.444088 0.103387 -0.272957

C 2.822572 0.331132 -0.310874

C 3.017593 -1.036822 -0.097941

C 3.876864 1.225742 -0.094173

C 4.256278 -1.513018 0.323243

H 2.207026 -1.752185 -0.250558

C 5.120108 0.753206 0.308253

H 3.727114 2.295951 -0.231024

C 5.302033 -0.613821 0.517525

H 4.405719 -2.580898 0.489389

H 5.942377 1.450250 0.474855

Cl 6.856469 -1.199383 1.044207

C 1.367382 1.799192 -1.720905

O 2.337368 2.238114 -2.294820

C -0.034749 2.259196 -1.935271

H -0.700416 1.458600 -2.281024

H -0.460874 2.649539 -1.001587

H -0.027901 3.052122 -2.689064

O -3.030689 1.085748 3.270123

H -3.498181 1.433192 2.489649

H -2.392176 1.789264 3.446064

B -2.884046 2.097568 0.051367

F -1.793686 2.357801 0.915707

F -4.047977 1.936914 0.840475

F -3.048631 3.154944 -0.828045

F -2.633475 0.906174 -0.637336

**BF4^-^**

Cartesian coordinates

O 2.767214 -0.043234 0.009797

H 1.941660 -0.546632 -0.058040

H 2.421354 0.855607 -0.038210

B -0.657093 0.007415 0.003401

F 0.141921 1.152089 -0.143719

F -1.229104 -0.009132 1.276281

F -1.649899 -0.001219 -0.977871

F 0.157609 -1.141758 -0.154595

**TS10**

Cartesian coordinates

C 1.478792 2.668938 -1.858755

C 2.179929 3.225954 0.459880

C 2.577316 3.369492 -1.038391

C 3.483390 2.954513 1.229334

C 4.396230 2.440832 0.159132

C 3.911070 2.675356 -1.066684

H 1.874868 2.112016 -2.722151

H 0.744270 3.381860 -2.269971

H 3.883042 3.880955 1.676936

H 3.353180 2.259857 2.074359

H 5.357723 1.966411 0.373051

H 4.417587 2.424569 -2.002869

H 2.686822 4.422325 -1.349679

H 1.648732 4.106532 0.861076

C 1.225185 2.070049 0.424402

C 0.787406 1.277182 1.553258

C -0.105602 0.737728 -1.138106

C -0.237958 0.324787 1.331340

H 1.794042 0.448841 1.746022

H 0.808159 1.718021 2.557293

C -0.848415 -0.437775 2.434663

H -1.919209 -0.196148 2.519790

H -0.792453 -1.519602 2.247478

H -0.363443 -0.197377 3.385832

O -0.531536 0.382194 -2.215110

C 0.842135 1.773396 -0.849410

N -1.544444 -0.942034 -0.165773

N -0.600298 0.049590 0.062989

C -2.910977 -0.551442 -0.089898

C -3.278716 0.787638 -0.262787

C -3.894633 -1.502876 0.210711

C -4.611161 1.174121 -0.151860

H -2.531351 1.551883 -0.483227

C -5.228249 -1.123092 0.305674

H -3.623481 -2.543877 0.375444

C -5.581210 0.212956 0.121838

H -4.888918 2.220827 -0.285965

H -5.992199 -1.867017 0.535990

Cl -7.254008 0.685029 0.251346

C -1.095717 -2.135155 -0.778098

O -1.896755 -2.917645 -1.237210

C 0.383180 -2.353692 -0.769924

H 0.867266 -1.786109 -1.577424

H 0.864077 -2.062051 0.171816

H 0.575855 -3.414447 -0.956137

O 2.913878 -0.253368 1.924661

H 3.313485 -0.387044 1.028033

H 2.702830 -1.175471 2.168350

B 3.888292 -2.564679 -0.079152

F 3.114718 -2.787329 1.087294

F 5.207848 -2.897797 0.162782

F 3.364099 -3.280900 -1.138977

F 3.801372 -1.160958 -0.374246

**M11**

C -1.887737 -1.691195 -1.978598

C -2.277258 -3.152907 -0.005708

C -2.910956 -2.636469 -1.329619

C -3.422195 -3.225475 1.018161

C -4.453912 -2.307601 0.434629

C -4.166421 -1.975613 -0.835885

H -2.350621 -0.818308 -2.463003

H -1.300085 -2.199923 -2.761455

H -3.823879 -4.248932 1.109160

H -3.111164 -2.944201 2.038979

H -5.356194 -2.003025 0.973515

H -4.788198 -1.342705 -1.475392

H -3.188816 -3.454381 -2.016366

H -1.773856 -4.127676 -0.117544

C -1.238087 -2.097652 0.278267

C -0.537148 -1.875606 1.492411

C -0.061121 -0.246542 -0.834295

C 0.403973 -0.873256 1.553865

H -2.242620 -0.137393 1.647312

H -0.702844 -2.495621 2.374990

C 1.207743 -0.571900 2.763064

H 2.278609 -0.760953 2.593344

H 1.120903 0.483405 3.059089

H 0.881306 -1.200550 3.598181

O 0.211738 0.503931 -1.759668

C -1.024407 -1.307578 -0.825295

N 1.594577 0.883409 0.475898

N 0.620061 -0.103872 0.423253

C 2.923555 0.482682 0.169239

C 3.161143 -0.678322 -0.576408

C 4.013416 1.220214 0.652958

C 4.461818 -1.085424 -0.857762

H 2.333359 -1.281742 -0.951849

C 5.314242 0.820957 0.366676

H 3.849906 2.108738 1.259106

C 5.533025 -0.328119 -0.390895

H 4.637701 -1.986218 -1.447478

H 6.158057 1.400218 0.744501

Cl 7.165219 -0.828277 -0.751420

C 1.162929 2.223484 0.465637

O 1.950154 3.124901 0.271570

C -0.293193 2.432044 0.741822

H -0.908928 2.074234 -0.093260

H -0.612989 1.914074 1.657268

H -0.476297 3.504765 0.858833

O -3.200755 0.076986 1.617805

H -3.594154 -0.495526 0.896096

H -3.320977 1.079102 1.332994

B -3.624594 2.387921 -0.585696

F -3.533714 2.390540 0.898358

F -4.950700 2.512596 -0.918073

F -2.854333 3.422958 -1.047372

F -3.110363 1.142360 -0.961770

**TS11**

Cartesian coordinates

C 3.511733 -1.943939 -1.083784

C 4.706553 0.181865 -1.602078

C 4.877029 -1.364583 -1.505959

C 5.957054 0.802758 -0.950894

C 6.529557 -0.331839 -0.161927

C 5.953613 -1.501066 -0.466662

H 3.600266 -2.749679 -0.337645

H 2.962728 -2.385156 -1.933224

H 6.676363 1.160366 -1.708027

H 5.721153 1.686272 -0.334175

H 7.351522 -0.204455 0.547644

H 6.231469 -2.470036 -0.041809

H 5.209782 -1.812508 -2.458090

H 4.576033 0.537795 -2.637847

C 3.434620 0.425375 -0.840310

C 2.896636 1.670361 -0.437148

C 1.554611 -0.791411 0.157074

C 1.709968 1.719453 0.245241

H 3.405557 2.610177 -0.655570

C 1.125716 3.000324 0.725001

H 0.048226 3.099627 0.541063

H 1.292690 3.151666 1.803219

H 1.626116 3.831439 0.217600

O 0.919123 -1.792953 0.469900

C 2.790966 -0.751555 -0.554264

N -0.187923 0.525871 1.172350

N 1.070046 0.507716 0.520195

C -1.318346 0.946739 0.330152

C -1.139902 1.235296 -1.019987

C -2.594915 0.914608 0.891653

C -2.246779 1.528626 -1.810592

H -0.151932 1.224193 -1.482222

C -3.699687 1.211690 0.104403

H -2.754401 0.645389 1.934562

C -3.517801 1.517246 -1.241629

H -2.116182 1.751631 -2.870183

H -4.701573 1.170921 0.533364

Cl -4.902092 1.869005 -2.231986

C -0.071018 1.148661 2.600351

O -0.925623 1.882842 2.969747

C 1.108908 0.637052 3.327640

H 1.247411 -0.439994 3.168594

H 2.024551 1.138449 2.982361

H 0.981898 0.847548 4.393878

H -0.679410 -2.336767 1.647575

H -0.528100 -0.568347 1.495509

H -2.096506 -1.741527 1.814662

B -3.489455 -2.358494 -0.158269

F -4.431434 -1.691615 -0.927428

F -3.651550 -1.964799 1.218815

F -3.657293 -3.735642 -0.243126

F -2.192291 -2.003024 -0.537234

O -1.160709 -1.632512 2.113996

**M12**

Cartesian coordinates

C 3.835640 -0.436013 -2.204899

C 5.213915 0.801969 -0.538140

C 5.287431 -0.125422 -1.789585

C 6.276435 0.286676 0.452400

C 6.570909 -1.090852 -0.050955

C 6.044151 -1.310381 -1.261171

H 3.693606 -1.483609 -2.514811

H 3.507643 0.175576 -3.062776

H 7.184087 0.914715 0.441221

H 5.924293 0.301381 1.497610

H 7.181769 -1.806255 0.506127

H 6.156738 -2.232463 -1.838687

H 5.839374 0.335415 -2.626565

H 5.370939 1.867334 -0.777023

C 3.801429 0.611387 -0.064727

C 3.234596 1.083838 1.143504

C 1.677939 -0.383047 -0.768259

C 1.920352 0.828128 1.433589

H 3.818229 1.664463 1.858919

C 1.264870 1.298163 2.682588

H 0.255905 1.702783 2.525250

H 1.196045 0.503350 3.441913

H 1.872561 2.092531 3.128911

O 0.899674 -0.960743 -1.518029

C 3.056860 -0.087444 -0.982032

N -0.169725 -0.199802 0.717504

N 1.199721 0.078682 0.506132

C -1.099255 0.879295 0.347570

C -0.637729 2.146709 0.005485

C -2.456537 0.563674 0.297017

C -1.552658 3.123401 -0.375596

H 0.424979 2.392899 0.010573

C -3.370981 1.539447 -0.082901

H -2.820529 -0.432834 0.549556

C -2.911144 2.813677 -0.411279

H -1.205110 4.119271 -0.651329

H -4.432456 1.296340 -0.125312

Cl -4.048658 4.040584 -0.885119

C -0.459301 -0.933080 2.133235

O -1.408083 -0.566917 2.730127

C 0.484488 -2.036955 2.358031

H 0.446513 -2.718473 1.495438

H 1.520936 -1.687186 2.446328

H 0.196621 -2.569256 3.269386

H -1.138308 -2.412745 -1.318739

H -0.418122 -1.032961 0.065760

H -2.088873 -2.562655 -0.118497

B -4.530965 -2.241331 -0.570390

F -5.281562 -1.096578 -0.318283

F -3.699946 -2.501910 0.574580

F -5.348933 -3.339324 -0.784505

F -3.677181 -2.034394 -1.660063

O -1.145923 -2.441368 -0.351917

**TS12**

C 3.825285 -0.667832 -2.067830

C 4.963629 1.117864 -0.753573

C 5.206828 -0.073690 -1.727478

C 6.045297 1.018019 0.339217

C 6.534129 -0.389603 0.207309

C 6.088066 -0.976009 -0.910600

H 3.828093 -1.769409 -2.094086

H 3.469633 -0.348611 -3.062580

H 6.865309 1.737771 0.170500

H 5.657743 1.251208 1.345253

H 7.211100 -0.852056 0.930702

H 6.342756 -1.989612 -1.234188

H 5.732264 0.233119 -2.648032

H 4.993555 2.098594 -1.257863

C 3.566139 0.866852 -0.260527

C 2.902439 1.514838 0.805112

C 1.609870 -0.521388 -0.725239

C 1.610031 1.169220 1.123688

H 3.396367 2.282542 1.402341

C 0.893444 1.768185 2.283269

H -0.141833 2.062686 2.072911

H 0.862769 1.082178 3.143421

H 1.432898 2.661072 2.617024

O 0.948189 -1.394724 -1.290319

C 2.949854 -0.121295 -0.990974

N -0.333445 -0.178969 0.565223

N 0.995937 0.206019 0.343077

C -1.337179 0.803495 0.238366

C -1.025603 2.026219 -0.361814

C -2.666861 0.481107 0.515879

C -2.037777 2.931296 -0.662481

H 0.004828 2.286893 -0.609795

C -3.683597 1.378690 0.209416

H -2.928542 -0.486509 0.939215

C -3.360518 2.604091 -0.368762

H -1.797920 3.884944 -1.133895

H -4.722808 1.114638 0.409801

Cl -4.625510 3.744508 -0.737834

C -0.581003 -1.231711 2.448166

O -1.427149 -0.703588 3.030913

C 0.713702 -1.874471 2.595904

H 1.110191 -2.284859 1.664240

H 1.419663 -1.149067 3.021664

H 0.574590 -2.690145 3.318817

H -0.999719 -3.081713 0.622340

H -0.450289 -0.985026 -0.080818

H -2.377701 -2.686374 1.154386

B -3.508177 -2.383648 -1.050466

F -4.293008 -1.390501 -1.605554

F -3.858623 -2.532543 0.338638

F -3.683763 -3.597878 -1.689044

F -2.145402 -2.015446 -1.085685

O -1.448006 -2.828684 1.446725

**AcOH_2_^+^+BF_4_^-^**

C -2.609805 0.148391 0.004268

O -3.449351 0.985303 0.179153

C -2.754299 -1.326970 0.037709

H -2.051785 -1.763815 0.760147

H -3.778145 -1.603268 0.302479

H -2.502549 -1.748374 -0.945270

H -0.089863 -0.400352 -0.238674

H -1.219087 1.479246 -0.287919

B 2.168348 0.138188 0.058159

F 2.362960 -0.018303 1.367869

F 1.594504 1.280712 -0.347659

F 3.009209 -0.470618 -0.776952

F 0.708260 -0.960672 -0.166030

O -1.311909 0.506825 -0.282712

**TS13**

C 2.472477 -0.312438 -1.763764

C 4.029545 -0.976938 0.094250

C 3.542638 -1.352494 -1.346568

C 4.095237 -2.294314 0.895476

C 3.289959 -3.239445 0.063179

C 3.002110 -2.738427 -1.144205

H 1.615110 -0.769724 -2.283403

H 2.879222 0.444563 -2.451072

H 5.134456 -2.648332 1.003685

H 3.715461 -2.186896 1.924640

H 3.017162 -4.240982 0.406186

H 2.456956 -3.267857 -1.930684

H 4.365495 -1.364730 -2.080890

H 5.002484 -0.455260 0.107256

C 2.963615 -0.045822 0.545121

C 2.587621 0.508529 1.868281

C 1.041395 1.076258 0.099811

C 1.273033 1.263708 1.612543

H 2.460436 -0.278273 2.628784

H 3.351018 1.196186 2.266888

C 1.011021 2.546719 2.322002

H 0.082076 3.015731 1.973434

H 0.908185 2.367232 3.400612

H 1.845703 3.242360 2.172692

O 0.109168 1.786533 -0.548861

C 0.385481 2.405426 -1.789283

O 1.505499 2.524054 -2.180486

C -0.881469 2.844982 -2.404689

H -1.466179 1.963190 -2.702558

H -1.490881 3.399727 -1.680933

H -0.678842 3.461098 -3.284568

C 2.112113 0.299111 -0.455122

N -0.984283 0.619745 1.811886

N 0.182453 0.359642 1.389084

C -2.049734 0.061222 1.140320

C -1.954095 -0.673415 -0.064671

C -3.312695 0.280933 1.730125

C -3.096321 -1.174935 -0.655960

H -0.979868 -0.860719 -0.520034

C -4.459199 -0.225287 1.145017

H -3.361220 0.854920 2.657310

C -4.341176 -0.951807 -0.044466

H -3.040820 -1.746356 -1.583359

H -5.438229 -0.065180 1.597393

Cl -5.761858 -1.600618 -0.781046

**M13**

C -2.148951 -0.723170 1.728914

C -4.032860 -0.880316 0.054954

C -3.431522 -1.517776 1.356697

C -4.395076 -2.053167 -0.879034

C -3.671555 -3.214933 -0.278772

C -3.164532 -2.930926 0.926832

H -1.318259 -1.385524 2.022653

H -2.313019 -0.041511 2.580670

H -5.483346 -2.235445 -0.899062

H -4.114975 -1.863176 -1.928807

H -3.609272 -4.190114 -0.769313

H -2.626867 -3.638863 1.564349

H -4.142401 -1.501846 2.200324

H -4.914848 -0.247775 0.254072

C -2.901963 -0.027625 -0.414088

C -2.655730 0.776264 -1.645455

C -0.797909 0.866009 -0.037654

C -1.270080 1.387430 -1.438177

H -2.676012 0.178209 -2.570835

H -3.392895 1.582744 -1.783482

C -0.930373 2.723056 -1.995492

H 0.055321 3.066676 -1.653595

H -0.912850 2.693061 -3.093480

H -1.691723 3.452461 -1.692496

O 0.097354 1.523177 0.759393

C -0.412898 2.537489 1.571671

O -1.569991 2.840570 1.527003

C 0.672411 3.116196 2.392655

H 1.189943 2.328668 2.954440

H 1.420208 3.581726 1.736675

H 0.269475 3.866000 3.078473

C -1.899557 0.033335 0.473472

N 0.959104 0.463663 -1.821445

N -0.193677 0.444154 -1.301117

C 2.032349 -0.032427 -1.132197

C 1.977468 -0.707414 0.113894

C 3.275304 0.144408 -1.784394

C 3.139230 -1.183708 0.682413

H 1.024737 -0.893113 0.611791

C 4.442622 -0.312059 -1.204931

H 3.288665 0.656872 -2.747713

C 4.364889 -0.977591 0.024348

H 3.114443 -1.723710 1.629286

H 5.405950 -0.169480 -1.694610

Cl 5.806073 -1.583470 0.745500

**TS14**

C 2.264937 0.992451 1.547402

C 4.095464 0.664562 -0.135789

C 3.521772 1.653672 0.931439

C 4.480559 1.520901 -1.359646

C 3.740143 2.799125 -1.129207

C 3.231207 2.874640 0.106170

H 1.453002 1.713565 1.733726

H 2.488128 0.515755 2.514870

H 5.569052 1.696833 -1.401250

H 4.227182 1.034216 -2.316093

H 3.671532 3.587110 -1.883756

H 2.685196 3.731512 0.510916

H 4.249491 1.890307 1.725822

H 4.956285 0.076993 0.227033

C 2.933193 -0.240285 -0.363361

C 2.728106 -1.341307 -1.325992

C 0.730937 -0.798597 0.276799

C 1.346758 -1.899299 -1.230432

H 2.881783 -1.004784 -2.369939

H 3.457975 -2.152847 -1.183831

C 1.006573 -3.256957 -1.676142

H 0.091381 -3.611755 -1.180727

H 0.785584 -3.255938 -2.756887

H 1.836627 -3.948831 -1.498047

O -0.235856 -1.030569 1.156054

C 0.067183 -1.361350 2.511629

O 1.174014 -1.661087 2.828467

C -1.169617 -1.265535 3.305894

H -1.486875 -0.214240 3.352548

H -1.982353 -1.818615 2.818737

H -1.001048 -1.643143 4.317525

C 1.927614 -0.028810 0.509674

N -0.877967 -1.184331 -1.550859

N 0.292919 -1.001833 -1.063101

C -1.952487 -0.464770 -1.067881

C -1.890370 0.717536 -0.295074

C -3.211207 -0.934002 -1.498841

C -3.052095 1.393306 0.039342

H -0.932732 1.146148 0.005324

C -4.376485 -0.273747 -1.149050

H -3.246079 -1.834914 -2.114028

C -4.289199 0.887544 -0.376422

H -3.006702 2.317031 0.618057

H -5.347373 -0.645600 -1.478725

Cl -5.738700 1.730772 0.062190

**M14**

C -1.992905 -1.906637 -1.728880

C -3.921757 -1.274005 -0.263838

C -3.511141 -1.622336 -1.729824

C -4.824623 -0.018789 -0.342797

C -4.607641 0.473794 -1.735978

C -3.907691 -0.386087 -2.482463

H -1.462495 -1.404651 -2.552493

H -1.764596 -2.980294 -1.823837

H -5.882929 -0.272621 -0.163972

H -4.571524 0.738136 0.417057

H -5.003551 1.430350 -2.085912

H -3.645757 -0.239458 -3.533686

H -4.057126 -2.499367 -2.115377

H -4.450582 -2.098638 0.245117

C -2.612117 -1.056917 0.410565

C -2.344282 -0.586445 1.749812

C -0.248580 -1.324583 0.077476

C -0.966092 -0.504720 2.186778

H -2.696197 0.506958 1.866130

H -2.996436 -1.011159 2.533832

C -0.628585 0.009215 3.512264

H 0.008521 -0.707536 4.053154

H -0.004905 0.911574 3.406594

H -1.528140 0.231301 4.094755

O 0.741545 -1.725681 -0.697301

C 1.706119 -2.681637 -0.241210

O 1.484916 -3.326441 0.733601

C 2.831187 -2.720558 -1.193046

H 2.462990 -3.056680 -2.172436

H 3.251251 -1.716291 -1.341390

H 3.601114 -3.409158 -0.834585

C -1.552384 -1.395168 -0.399760

N 1.305887 -0.630931 1.827690

N 0.068517 -0.775928 1.339635

C 2.304673 -0.161536 1.002591

C 2.131691 0.628735 -0.158366

C 3.629050 -0.436711 1.417757

C 3.226893 1.115971 -0.861233

H 1.129159 0.879421 -0.507904

C 4.723894 0.042704 0.714203

H 3.776043 -1.046707 2.312486

C 4.519631 0.817057 -0.429791

H 3.072656 1.732394 -1.748877

H 5.738104 -0.186783 1.045406

Cl 5.897025 1.418910 -1.323118

O -2.909263 2.199552 2.360599

H -2.058012 2.393026 1.925467

H -3.537862 2.556909 1.715747

B -1.160015 2.346406 -0.525182

F -2.538138 2.574611 -0.406921

F -0.922851 1.291354 -1.415001

F -0.500926 3.489506 -0.946063

F -0.673589 1.977449 0.769276

**TS15**

C -2.078613 -1.632063 -1.842130

C -3.972682 -1.258547 -0.255872

C -3.597811 -1.380501 -1.764969

C -4.962097 -0.081182 -0.146580

C -4.774404 0.644862 -1.440502

C -4.040649 -0.054088 -2.312924

H -1.585438 -1.038894 -2.627100

H -1.838660 -2.686395 -2.055437

H -6.000149 -0.439010 -0.036603

H -4.782901 0.550809 0.738581

H -5.214780 1.626225 -1.633585

H -3.792067 0.266480 -3.328260

H -4.142876 -2.200187 -2.262768

H -4.417517 -2.182326 0.153270

C -2.646542 -1.042736 0.400137

C -2.334614 -0.714544 1.754099

C -0.289644 -1.220046 -0.040314

C -0.971327 -0.694359 2.173075

H -2.577619 0.527193 1.925010

H -3.053895 -0.984396 2.541275

C -0.591185 -0.368662 3.550919

H 0.035335 -1.167432 3.976128

H 0.049067 0.527976 3.563040

H -1.475199 -0.217363 4.178812

O 0.693194 -1.523364 -0.887418

C 1.546993 -2.617132 -0.611430

O 1.291355 -3.378875 0.270485

C 2.660496 -2.626611 -1.581107

H 2.261324 -2.709817 -2.600925

H 3.213154 -1.678010 -1.535265

H 3.331549 -3.464874 -1.375618

C -1.602298 -1.255998 -0.480807

N 1.294934 -0.789640 1.759671

N 0.052976 -0.847415 1.266329

C 2.322482 -0.282043 0.994784

C 2.208695 0.640626 -0.071995

C 3.626318 -0.661758 1.393568

C 3.337940 1.150933 -0.700898

H 1.226062 0.973760 -0.406339

C 4.755779 -0.158762 0.764333

H 3.729133 -1.372561 2.217233

C 4.607981 0.745086 -0.289777

H 3.228521 1.869616 -1.515620

H 5.751691 -0.469032 1.086621

Cl 6.026340 1.375354 -1.096760

O -2.741552 1.881480 2.297324

H -1.880101 2.196932 1.938029

H -3.365922 2.248268 1.646023

B -1.065831 2.433263 -0.455457

F -2.457263 2.557456 -0.312073

F -0.767809 1.431611 -1.380093

F -0.500786 3.638225 -0.829475

F -0.543652 2.051188 0.828405

**M15**

C 2.714072 -0.644967 2.119388

C 3.182036 -2.622949 0.682941

C 3.838347 -1.564728 1.615074

C 4.186849 -2.865735 -0.454404

C 5.019324 -1.618711 -0.434545

C 4.823786 -0.907004 0.688587

H 3.027484 0.403806 2.223538

H 2.342471 -0.960554 3.107798

H 4.816865 -3.750131 -0.259209

H 3.702854 -3.064118 -1.425768

H 5.740531 -1.367060 -1.217769

H 5.350543 0.015949 0.948298

H 4.384225 -2.020021 2.458766

H 2.923804 -3.557732 1.207649

C 1.919308 -1.922249 0.256802

C 1.060253 -2.192740 -0.806481

C 0.540662 -0.050169 0.850023

C -0.039840 -1.375570 -1.052217

H 2.708284 -0.781638 -2.379094

H 1.245120 -3.019349 -1.496878

C -0.879584 -1.505281 -2.261984

H -1.898695 -1.856752 -2.041532

H -0.995015 -0.521324 -2.742459

H -0.424688 -2.209138 -2.967365

O 0.296473 1.045842 1.569696

C -0.977130 1.217959 2.139100

O -1.607213 0.277069 2.526567

C -1.289605 2.658062 2.249658

H -0.447885 3.201314 2.697982

H -1.425673 3.068492 1.240231

H -2.197713 2.809256 2.840041

C 1.661677 -0.822809 1.076623

N -1.301176 0.586702 -0.436613

N -0.319796 -0.328025 -0.191630

C -2.590239 0.123997 -0.399236

C -3.015808 -1.128986 0.108123

C -3.601208 1.016589 -0.837306

C -4.364470 -1.463498 0.177147

H -2.276267 -1.840957 0.484507

C -4.946078 0.685379 -0.770373

H -3.296543 1.991717 -1.227132

C -5.329471 -0.558407 -0.261539

H -4.667593 -2.432075 0.581454

H -5.703260 1.392497 -1.115652

Cl -7.031054 -0.974386 -0.165771

O 3.173866 0.038263 -2.115794

H 2.453059 0.808063 -1.971014

H 3.644639 -0.156246 -1.259722

B 2.099536 2.766570 -0.789721

F 2.872003 1.972359 0.055348

F 1.030610 3.346462 -0.165972

F 2.875436 3.669819 -1.476824

F 1.553973 1.803414 -1.799029

**M16**

C 3.348363 -1.421280 -1.320672

C 4.989142 0.331820 -0.647871

C 4.854217 -1.130969 -1.167069

C 6.108078 0.318061 0.408485

C 6.204544 -1.129097 0.774747

C 5.531353 -1.912409 -0.076719

H 3.070589 -2.443587 -1.023646

H 3.010442 -1.303313 -2.363599

H 7.061879 0.685917 -0.006837

H 5.890510 0.973758 1.267319

H 6.801384 -1.486176 1.617903

H 5.490197 -3.004086 -0.032391

H 5.373498 -1.288057 -2.127516

H 5.194071 1.058727 -1.451720

C 3.627608 0.613101 -0.085503

C 3.213103 1.656008 0.735972

C 1.407912 -0.289781 -0.042347

C 1.897335 1.725577 1.174886

H -0.772798 -0.064834 1.255620

H 3.907395 2.421932 1.084736

C 1.412973 2.758756 2.113527

H 0.775909 3.506712 1.617743

H 0.805191 2.310099 2.910935

H 2.264854 3.285747 2.554327

O 0.519629 -1.267025 -0.284067

C -0.431868 -1.089559 -1.310339

O -0.523280 -0.036547 -1.867994

C 2.716649 -0.378861 -0.462550

N -0.304462 0.853890 1.151543

N 1.006615 0.772018 0.731729

C -1.130609 1.854195 0.599305

C -0.715819 2.736163 -0.403938

C -2.429302 1.959265 1.117164

C -1.584670 3.713648 -0.881005

H 0.281574 2.654906 -0.840966

C -3.303132 2.925635 0.633863

H -2.752309 1.273085 1.902740

C -2.873461 3.804112 -0.360368

H -1.259468 4.395010 -1.668616

H -4.313926 3.002125 1.036918

Cl -3.964342 5.028552 -0.958955

O -2.138517 -1.206568 1.211579

H -2.134887 -2.177878 1.296493

H -2.922433 -1.057007 0.665361

B -3.491738 -4.150810 0.293745

F -4.003921 -2.888359 -0.047759

F -4.442831 -4.885008 0.988907

F -3.078503 -4.821144 -0.853371

F -2.354009 -3.948400 1.137329

C -1.148894 -2.353019 -1.562971

H -1.199907 -2.986732 -0.674357

H -2.149820 -2.145926 -1.953501

H -0.594926 -2.903990 -2.338020

**TS16**

C 3.114552 -1.673088 -1.089469

C 4.911503 0.041278 -0.863995

C 4.634145 -1.449115 -1.228862

C 6.179070 0.048287 0.011181

C 6.293858 -1.374852 0.457051

C 5.465420 -2.183209 -0.215236

H 2.864681 -2.640325 -0.623708

H 2.607244 -1.672275 -2.070649

H 7.070607 0.354166 -0.563652

H 6.111731 0.763332 0.847852

H 7.006540 -1.701952 1.218924

H 5.398622 -3.266907 -0.082898

H 4.968939 -1.703746 -2.249029

H 5.034246 0.685154 -1.751207

C 3.660361 0.449587 -0.137138

C 3.434022 1.617764 0.610724

C 1.405461 -0.350260 0.337133

C 2.201991 1.822121 1.198336

H -0.462643 0.209838 1.770521

H 4.212121 2.367998 0.758709

C 1.878009 3.000034 2.036980

H 1.138541 3.661037 1.559801

H 1.443275 2.689675 2.997519

H 2.783027 3.587320 2.223634

O 0.474402 -1.189360 0.354693

C -0.935876 -1.237138 -1.277472

O -0.781152 -0.264036 -1.875604

C 2.673154 -0.509635 -0.266893

N -0.039657 1.110907 1.539424

N 1.216938 0.880966 0.994865

C -0.914026 1.948043 0.815326

C -0.521417 2.685350 -0.306516

C -2.228859 2.069290 1.288893

C -1.425670 3.535199 -0.941773

H 0.489135 2.591332 -0.710656

C -3.135156 2.908249 0.656304

H -2.539254 1.486310 2.158822

C -2.724879 3.646270 -0.456492

H -1.116394 4.105522 -1.819095

H -4.157746 2.993683 1.028641

Cl -3.853418 4.722715 -1.243878

O -2.225837 -0.862281 0.167323

H -2.370399 -1.703268 0.666131

H -3.089470 -0.721701 -0.260807

B -3.800544 -3.745995 0.422851

F -4.132082 -2.724673 -0.485694

F -4.881632 -4.055008 1.226673

F -3.330686 -4.856912 -0.259567

F -2.737422 -3.234192 1.251427

C -0.986009 -2.680157 -1.355084

H -0.993740 -3.155063 -0.372830

H -1.899446 -2.946092 -1.906313

H -0.110586 -3.004950 -1.932905

**P-39**

Cartesian coordinates

C -2.767283 -1.192093 -2.186578

C -3.840415 -2.493836 -0.350464

C -4.106435 -1.797647 -1.720469

C -5.038357 -2.150589 0.555188

C -5.651249 -0.970444 -0.129990

C -5.152554 -0.782063 -1.357785

H -2.882099 -0.205939 -2.661685

H -2.260799 -1.829824 -2.929891

H -5.753946 -2.988168 0.618654

H -4.738406 -1.942623 1.596051

H -6.443686 -0.369675 0.324101

H -5.474028 -0.003746 -2.055524

H -4.498781 -2.494036 -2.480400

H -3.695556 -3.583535 -0.437850

C -2.553531 -1.869112 0.098649

C -1.959661 -1.928412 1.366891

C -0.785204 -0.447142 -0.690761

C -0.784166 -1.247674 1.602157

H -2.413197 -2.487256 2.186552

C -0.085602 -1.218449 2.905350

H 0.915066 -1.669930 2.840207

H 0.057842 -0.187304 3.258114

H -0.664949 -1.771237 3.651624

O -0.212941 0.239784 -1.636505

C -1.974265 -1.134271 -0.925478

N 0.986301 0.097172 0.768196

N -0.217730 -0.532012 0.560496

C 2.176777 -0.563553 0.464803

C 2.195165 -1.886749 0.004881

C 3.389473 0.120993 0.644463

C 3.407231 -2.517309 -0.268622

H 1.265478 -2.439800 -0.148393

C 4.596242 -0.509562 0.373236

H 3.377178 1.154939 0.992628

C 4.601951 -1.828638 -0.081814

H 3.415717 -3.547023 -0.628809

H 5.535149 0.028169 0.514738

Cl 6.121245 -2.623558 -0.420072

C -2.271367 2.749720 0.832020

O -2.684738 3.870557 0.651875

C -2.986528 1.661137 1.557816

H -2.432169 1.390279 2.467704

H -3.996013 1.979943 1.835451

H -3.046055 0.757911 0.934767

H 0.531794 0.823676 -1.340354

H 0.960260 1.105769 0.933634

H -0.624169 3.099151 -0.062896

B 1.797779 3.224850 -0.558552

F 1.756859 2.886262 0.805344

F 0.667640 4.014303 -0.853828

F 2.961667 3.872620 -0.880249

F 1.691390 2.015190 -1.310327

O -1.067826 2.344554 0.386540

**R1a**

C 0.709456 -0.256103 -0.104706

C 1.924851 -0.156967 -0.127224

C 3.350200 -0.067530 -0.115040

C 4.082113 -1.210486 0.521412

H 3.778421 -1.344536 1.570426

H 3.857451 -2.159592 0.011764

H 5.167582 -1.054120 0.494279

O -1.393704 0.804733 -0.543235

C -1.231057 1.888895 0.238515

O -0.568978 1.890095 1.250089

C -1.978526 3.054799 -0.316750

H -1.677718 3.246401 -1.354874

H -3.055085 2.838445 -0.335549

H -1.799549 3.947045 0.290603

C 3.984338 0.993335 -0.653664

H 5.075260 1.056489 -0.639405

H 3.436837 1.817008 -1.117341

C -0.736059 -0.443532 -0.145161

C -1.169917 -1.446930 -1.223482

C -1.373732 -0.987661 1.162348

C -2.537041 -1.908371 -0.753626

H -0.462187 -2.290404 -1.217207

H -1.137376 -0.991595 -2.222554

C -2.313112 -2.121919 0.737112

H -1.910013 -0.174570 1.667241

H -0.599098 -1.319074 1.866798

H -3.280528 -1.113391 -0.926078

H -2.893574 -2.805769 -1.278332

H -3.243623 -2.139583 1.321629

H -1.824438 -3.096550 0.895359

**M3a**

C -2.567422 0.255129 0.003243

C -2.688440 -0.759373 0.905606

C -2.561790 -0.651749 2.376019

C -2.168531 -1.886195 3.119456

H -2.967555 -2.641914 3.098064

H -1.283576 -2.364004 2.670626

H -1.946286 -1.660669 4.169355

O -2.412513 -1.972941 -1.624913

C -2.904214 -2.573917 -0.710139

O -3.044927 -2.044931 0.555754

C -3.404291 -3.970724 -0.717034

H -3.381116 -4.375718 -1.732361

H -2.778165 -4.591874 -0.061596

H -4.424793 -4.016669 -0.316462

C 2.792276 1.906771 0.311600

C 3.126756 0.563478 0.011155

C 4.467319 0.256615 -0.287831

C 5.454129 1.235397 -0.326474

C 5.112672 2.563448 -0.082427

C 3.797855 2.885357 0.233969

C 1.450044 2.367807 0.747377

C 0.720821 3.288199 -0.017779

C -0.509314 3.769792 0.433278

C -1.017726 3.357219 1.663878

C -0.285514 2.463470 2.447488

C 0.932316 1.965861 1.988976

H 4.750121 -0.770442 -0.517821

H 6.484244 0.958769 -0.561192

H 5.871155 3.348406 -0.127328

H 3.531189 3.922465 0.454197

H 1.117764 3.620512 -0.981975

H -1.071544 4.475822 -0.183439

H -1.976462 3.743831 2.020325

H -0.660858 2.154580 3.426109

H 1.506122 1.273069 2.609132

P 1.879722 -0.761855 -0.229517

C 2.082504 -1.317299 -2.042538

C 2.255289 -0.036614 -2.859939

C 3.236488 -2.278367 -2.304760

C 0.782011 -1.991112 -2.483239

H 1.471049 0.705365 -2.634186

H 3.231504 0.444106 -2.704687

H 2.173922 -0.278265 -3.930890

H 3.103936 -3.240543 -1.790420

H 3.268599 -2.496799 -3.383792

H 4.222338 -1.875551 -2.040946

H 0.892572 -2.324715 -3.526948

H 0.526517 -2.878343 -1.887565

H -0.076138 -1.305753 -2.445704

C 2.228729 -2.127315 1.033256

C 3.680305 -2.591035 1.089051

C 1.855526 -1.544724 2.396554

C 1.310917 -3.303609 0.708791

H 4.055915 -2.970424 0.130378

H 4.355146 -1.798547 1.440002

H 3.757088 -3.415730 1.814893

H 0.813986 -1.186220 2.429447

H 1.964725 -2.323431 3.167548

H 2.515014 -0.710967 2.680370

H 1.360770 -4.040977 1.525037

H 0.256256 -2.993966 0.608897

H 1.605683 -3.821139 -0.214838

Au -0.411841 -0.225200 0.050837

C -2.852631 0.516241 2.973357

H -2.790014 0.624382 4.058704

H -3.168887 1.390360 2.398011

C -3.031413 1.165300 -0.841350

C -2.258667 1.837682 -1.946424

C -4.454778 1.677291 -0.862166

C -3.174910 2.955579 -2.433099

H -2.104607 1.086223 -2.743995

H -1.253731 2.162618 -1.636956

C -4.571401 2.389617 -2.206607

H -4.575946 2.402314 -0.037355

H -5.197494 0.885099 -0.689061

H -3.031263 3.853969 -1.809372

H -2.981507 3.251274 -3.473303

H -5.363370 3.151314 -2.218734

H -4.812632 1.660526 -2.997699

**TS3a**

C 2.757796 -1.047211 1.738620

C 3.256418 -0.345466 0.615918

C 4.649638 -0.297637 0.422877

C 5.534541 -0.932706 1.286979

C 5.037944 -1.649841 2.372701

C 3.665790 -1.698988 2.589724

C 1.321896 -1.129581 2.123326

C 0.729693 -0.095562 2.864089

C -0.585980 -0.208562 3.312304

C -1.331906 -1.351952 3.020869

C -0.751725 -2.383815 2.283364

C 0.567770 -2.276658 1.843267

H 5.058466 0.245701 -0.429409

H 6.609710 -0.871256 1.104745

H 5.718008 -2.163417 3.056293

H 3.271294 -2.245670 3.450437

H 1.315679 0.796725 3.103421

H -1.028404 0.599422 3.901047

H -2.364020 -1.438550 3.371739

H -1.329955 -3.279897 2.045770

H 1.025084 -3.094369 1.278268

P 2.166180 0.452033 -0.632604

C 2.741526 2.255813 -0.805291

C 2.995455 2.760462 0.615120

C 3.976744 2.480223 -1.671407

C 1.580514 3.054990 -1.402631

H 2.142622 2.561788 1.283842

H 3.892503 2.316314 1.069440

H 3.144258 3.851189 0.592394

H 3.811216 2.198705 -2.720469

H 4.216928 3.555362 -1.666272

H 4.872919 1.959539 -1.310216

H 1.888821 4.105904 -1.519598

H 1.273726 2.694121 -2.394371

H 0.692642 3.040888 -0.752264

C 2.404828 -0.569508 -2.212341

C 3.853353 -0.840475 -2.604724

C 1.714675 -1.908522 -1.937815

C 1.682405 0.150083 -3.349409

H 4.452246 0.069475 -2.740069

H 4.367839 -1.488095 -1.881732

H 3.862486 -1.376279 -3.567284

H 0.634541 -1.787399 -1.756684

H 1.835174 -2.569467 -2.810638

H 2.149080 -2.431370 -1.071100

H 1.646508 -0.505450 -4.233552

H 0.643070 0.404034 -3.084635

H 2.195267 1.073940 -3.652801

Au -0.187761 0.417274 -0.257632

C -4.755739 0.726995 -0.860799

C -2.999060 -0.787867 -0.430437

C -4.401053 -0.594448 -0.609377

H -5.780706 1.060610 -0.663249

H -4.226749 1.300729 -1.620077

C -5.394986 -1.667016 -0.364082

H -4.973712 -2.519950 0.183770

H -6.247820 -1.264034 0.200754

H -5.805150 -2.045319 -1.312555

O -2.418680 -2.027498 -0.477793

C -2.717705 -2.867751 -1.525647

O -3.408238 -2.521035 -2.446473

C -2.077642 -4.189750 -1.320710

H -1.009065 -4.077975 -1.095729

H -2.535180 -4.685651 -0.453134

H -2.211145 -4.818893 -2.205000

C -2.256604 0.335813 -0.126595

C -3.033155 1.454430 0.297529

C -3.733217 1.507593 1.641462

C -2.700025 2.878746 -0.072060

C -4.387814 2.877130 1.666879

H -2.911025 1.491127 2.383474

H -4.374203 0.646073 1.868383

C -3.339843 3.765028 1.001661

H -3.081880 3.097335 -1.081281

H -1.607270 2.995929 -0.154986

H -5.319742 2.860261 1.077273

H -4.651948 3.204598 2.681642

H -3.752341 4.697969 0.594808

H -2.581221 4.054481 1.745500

**M4a**

C 2.804513 1.432639 -1.337061

C 3.215497 0.341249 -0.535647

C 4.593533 0.086406 -0.406680

C 5.548985 0.887658 -1.021845

C 5.141860 1.983002 -1.780064

C 3.784811 2.243342 -1.933182

C 1.388431 1.775228 -1.645983

C 0.712961 1.100414 -2.673915

C -0.580987 1.472066 -3.038831

C -1.223450 2.519238 -2.377241

C -0.561644 3.194730 -1.351322

C 0.738010 2.832607 -0.994405

H 4.932885 -0.760896 0.190157

H 6.609937 0.656811 -0.902609

H 5.879464 2.629051 -2.261939

H 3.460156 3.088968 -2.545353

H 1.216817 0.285806 -3.202485

H -1.086258 0.942892 -3.850886

H -2.238297 2.810163 -2.661782

H -1.059331 4.014500 -0.827225

H 1.259097 3.378248 -0.201807

P 2.033343 -0.716238 0.393375

C 2.398425 -2.523034 -0.076767

C 2.653747 -2.537963 -1.583368

C 3.565422 -3.170876 0.661088

C 1.129655 -3.332767 0.206738

H 1.856710 -2.023292 -2.143477

H 3.612904 -2.075669 -1.856117

H 2.678352 -3.580076 -1.937982

H 3.378399 -3.264655 1.739679

H 3.704821 -4.192179 0.272274

H 4.520896 -2.648854 0.520693

H 1.319200 -4.394241 -0.017619

H 0.806964 -3.274097 1.256050

H 0.287710 -3.007056 -0.422902

C 2.368429 -0.337666 2.222280

C 3.825687 -0.411878 2.663003

C 1.871388 1.094395 2.430936

C 1.517396 -1.292855 3.056983

H 4.290607 -1.391369 2.493989

H 4.447629 0.356970 2.184860

H 3.871181 -0.220738 3.747016

H 0.802114 1.192162 2.191567

H 2.002527 1.379392 3.487013

H 2.432408 1.822492 1.824436

H 1.549892 -0.982016 4.112954

H 0.459764 -1.286087 2.746273

H 1.881752 -2.329248 3.014021

Au -0.324675 -0.308054 0.107937

C -4.676740 -0.351170 -0.495436

C -3.088702 1.024921 0.506643

C -4.440461 0.910820 0.229968

H -5.077961 -0.128845 -1.500523

H -5.471060 -0.940614 -0.006443

C -5.483836 1.877803 0.578119

H -5.091855 2.758177 1.098422

H -6.026851 2.200029 -0.323826

H -6.245025 1.393230 1.209862

O -2.558195 2.140255 1.107135

C -1.902007 1.974537 2.301877

O -1.898268 0.927222 2.893572

C -1.242199 3.241327 2.704954

H -0.438498 3.475592 1.992078

H -1.952329 4.077181 2.668613

H -0.820376 3.150398 3.710059

C -2.344542 -0.065999 0.030373

C -3.302515 -1.043134 -0.550911

C -2.932366 -1.549148 -1.957785

C -3.288399 -2.381823 0.230783

C -3.787288 -2.801936 -2.130464

H -1.857130 -1.798343 -1.959364

H -3.089950 -0.787526 -2.736254

C -3.962736 -3.378696 -0.715035

H -3.772387 -2.292677 1.214520

H -2.236525 -2.656008 0.413942

H -4.764242 -2.537387 -2.564201

H -3.327722 -3.512243 -2.832295

H -5.028983 -3.488390 -0.465970

H -3.521849 -4.380113 -0.607558

**TS4a**

C -2.937953 1.566897 -0.784892

C -3.176515 0.250581 -0.320811

C -4.510032 -0.190971 -0.226113

C -5.584563 0.626033 -0.558953

C -5.347754 1.929879 -0.986389

C -4.039319 2.385142 -1.093569

C -1.599802 2.179529 -0.995293

C -1.218649 3.293149 -0.232979

C -0.022632 3.960646 -0.494624

C 0.806075 3.534215 -1.532658

C 0.434802 2.430538 -2.302329

C -0.754617 1.754005 -2.031821

H -4.723949 -1.198167 0.129504

H -6.604071 0.243707 -0.472815

H -6.179123 2.590880 -1.242080

H -3.847048 3.403146 -1.443106

H -1.874680 3.641650 0.570425

H 0.257487 4.826341 0.110887

H 1.734348 4.068992 -1.750158

H 1.073176 2.100328 -3.125698

H -1.050816 0.899390 -2.646801

P -1.847640 -0.856288 0.305444

C -2.315699 -1.230415 2.120196

C -2.741803 0.103637 2.732242

C -3.407108 -2.276572 2.320725

C -1.055963 -1.712783 2.845359

H -2.004330 0.901058 2.542109

H -3.716789 0.451049 2.361382

H -2.825580 -0.006199 3.824702

H -3.111438 -3.266473 1.945771

H -3.590257 -2.388635 3.401162

H -4.370376 -2.006714 1.870284

H -1.315131 -1.961392 3.886591

H -0.614434 -2.613467 2.396726

H -0.276839 -0.937154 2.878360

C -1.878135 -2.398275 -0.797060

C -3.251184 -3.015742 -1.034139

C -1.315091 -1.939445 -2.142074

C -0.936794 -3.431760 -0.182474

H -3.777021 -3.290794 -0.110815

H -3.905703 -2.360995 -1.625305

H -3.120353 -3.940642 -1.617886

H -0.303264 -1.523963 -2.033538

H -1.252144 -2.798402 -2.829093

H -1.955948 -1.182351 -2.620320

H -0.774126 -4.249240 -0.902341

H 0.050336 -3.003150 0.052070

H -1.346721 -3.882536 0.732820

Au 0.404473 -0.096018 0.331927

C 4.660944 -0.470700 1.243735

C 3.369604 -0.295132 -0.643325

C 4.597948 -0.674477 -0.234284

H 5.523791 0.120379 1.589501

H 4.738795 -1.430990 1.786114

C 5.703688 -1.216932 -1.050502

H 5.458602 -1.221957 -2.120191

H 6.628922 -0.637165 -0.913703

H 5.945555 -2.249926 -0.754912

O 2.948274 -0.251738 -1.956009

C 2.332106 -1.357575 -2.472704

O 2.209751 -2.382940 -1.853317

C 1.855429 -1.085692 -3.854785

H 1.017729 -0.374110 -3.819358

H 2.643942 -0.615497 -4.455997

H 1.516717 -2.009968 -4.332562

C 2.489352 0.158323 0.456169

C 3.328033 0.119112 1.596488

C 3.009543 1.796827 0.899273

C 2.914573 0.359767 3.011061

C 2.184468 2.458737 1.981617

H 2.765003 2.126957 -0.120621

H 4.095982 1.983905 0.959115

C 1.834171 1.424210 3.049754

H 3.803482 0.658316 3.588839

H 2.576353 -0.594745 3.446564

H 2.773705 3.283591 2.409525

H 1.276689 2.906752 1.552519

H 1.735727 1.865732 4.050300

H 0.858090 0.971103 2.813704

**M5a**

C -0.930260 2.151364 0.136388

C 0.666059 0.561352 -0.250747

C 0.530204 1.897873 -0.082573

H -1.344522 2.848771 -0.614694

H -1.114611 2.647459 1.106849

C 1.579102 2.942055 -0.108948

H 2.576639 2.509095 -0.261909

H 1.411872 3.679519 -0.911228

H 1.609028 3.520571 0.828762

O 1.857206 -0.067839 -0.570021

C 2.344659 -0.996904 0.296157

O 1.829327 -1.233712 1.359660

C 3.558314 -1.648499 -0.266381

H 3.274636 -2.276694 -1.122341

H 4.266043 -0.900503 -0.644477

H 4.041385 -2.274699 0.489312

C -0.616169 -0.133539 -0.166210

C -1.572338 0.802092 0.065309

C -0.889990 -1.588569 -0.343230

C -3.021559 0.490485 0.205118

C -2.376845 -1.819241 -0.593671

H -0.561300 -2.143388 0.553984

H -0.280951 -1.998730 -1.167534

C -3.239005 -1.011639 0.368648

H -3.575651 0.861153 -0.679150

H -3.460867 1.041340 1.054768

H -2.622912 -1.528307 -1.630446

H -2.613218 -2.891429 -0.516529

H -4.303183 -1.259073 0.234616

H -2.987683 -1.303651 1.403734

**TS6a**

C -0.858074 -1.426534 1.396230

C -1.764628 -2.217350 0.529801

C -1.799075 0.038819 -0.025097

C -2.284136 -1.225927 -0.487372

H -1.278783 -3.113799 0.110396

H -2.609841 -2.610369 1.121551

C -3.672598 -1.335339 -1.025516

H -3.854659 -0.594825 -1.814777

H -3.839830 -2.334245 -1.448855

H -4.412400 -1.173773 -0.228629

O -2.168665 1.158611 -0.655340

C -2.427242 2.351709 0.045820

O -2.651728 2.334520 1.218259

C -2.407413 3.490513 -0.893102

H -1.388979 3.622185 -1.285883

H -3.054574 3.285895 -1.755117

H -2.727297 4.405414 -0.387714

C -0.913146 -0.082208 1.079253

N -0.252480 -1.600627 -2.281837

N -1.402705 -1.746097 -2.064721

C 0.852037 -0.932016 -1.733004

C 1.976299 -1.681244 -1.366399

C 0.852372 0.467884 -1.680234

C 3.084948 -1.023256 -0.855612

H 1.961809 -2.768210 -1.458663

C 1.979975 1.120985 -1.197715

H -0.009693 1.033854 -2.036011

C 3.077787 0.372134 -0.771442

H 3.957953 -1.590099 -0.530756

H 2.001672 2.210536 -1.149381

Cl 4.460583 1.187349 -0.112568

C 0.060230 -1.978087 2.408500

C -0.082080 0.952574 1.757468

C 0.674183 -0.895959 3.287315

H 0.847223 -2.532448 1.857309

H -0.448113 -2.761271 2.994498

C 1.104557 0.310306 2.466799

H -0.707883 1.495786 2.485225

H 0.243826 1.716649 1.032095

H -0.065472 -0.578311 4.041235

H 1.523299 -1.309384 3.848507

H 1.600801 1.055072 3.104669

H 1.856496 -0.003581 1.722329

**M7a**

C -0.637095 -2.003333 0.151638

C -1.575803 -2.011181 -0.989410

C -1.523330 0.051579 0.133294

C -2.181693 -0.606375 -1.021143

H -1.073635 -2.316204 -1.922942

H -2.357765 -2.773723 -0.838044

C -3.691651 -0.558870 -0.886010

H -4.047863 0.479068 -0.904351

H -4.149909 -1.094871 -1.726392

H -4.019949 -1.026026 0.052143

O -1.871891 1.289454 0.407996

C -1.900351 1.806930 1.746854

O -2.120139 1.068654 2.652389

C -1.678210 3.260350 1.715078

H -0.633417 3.453043 1.430773

H -2.308190 3.731135 0.950461

H -1.873979 3.697068 2.697823

C -0.615471 -0.781747 0.813415

N -0.887567 0.528248 -2.800418

N -1.947182 0.045778 -2.435206

C 0.314279 0.608332 -2.052428

C 1.247124 -0.429874 -2.140448

C 0.598978 1.768495 -1.325334

C 2.434326 -0.344272 -1.424476

H 1.029178 -1.311016 -2.747478

C 1.788991 1.853768 -0.609689

H -0.119806 2.589996 -1.308549

C 2.690432 0.792147 -0.653505

H 3.158471 -1.159881 -1.460985

H 2.015215 2.742842 -0.019620

Cl 4.156606 0.882076 0.277311

C 0.225020 -3.130683 0.530829

C 0.293017 -0.492255 1.963839

C 0.883714 -2.938562 1.888963

H 0.985331 -3.219416 -0.271986

H -0.339044 -4.074461 0.452087

C 1.413135 -1.522805 2.046709

H -0.296577 -0.496501 2.895773

H 0.696086 0.531488 1.881999

H 0.147327 -3.149681 2.682072

H 1.689549 -3.673656 2.016214

H 1.941436 -1.409865 3.003306

H 2.162229 -1.327546 1.260640

**R1l**

C -4.295965 -0.337699 -0.358891

C -5.181504 0.817228 -0.349096

C -5.928378 1.774868 -0.461828

C -6.743893 2.936140 -0.615300

C -6.229583 3.972083 -1.570005

H -5.241913 4.344308 -1.258206

H -6.095528 3.552173 -2.578374

H -6.913595 4.826849 -1.641461

O -4.563561 -1.230025 0.774462

C -4.747955 -0.769120 2.026499

O -4.693299 0.393475 2.348782

C -5.040647 -1.907894 2.948270

H -5.968271 -2.410464 2.643465

H -4.243817 -2.661432 2.898169

H -5.142744 -1.549146 3.976873

C -7.900731 3.078128 0.062505

H -8.514034 3.973600 -0.065001

H -8.259340 2.314097 0.755853

C -4.531993 -1.240291 -1.610480

C -2.767842 -0.031513 -0.465867

C -3.162344 -1.805960 -2.035688

H -5.008592 -0.658379 -2.410275

H -5.247204 -2.024752 -1.330090

C -2.274051 -1.440852 -0.860300

H -3.201607 -2.889118 -2.218866

H -2.814207 -1.340813 -2.971456

C 3.872859 -0.351813 0.199946

C 3.298503 -1.411279 -0.519428

C 1.960614 -1.748931 -0.345997

C 1.159784 -1.014234 0.563432

C 1.739277 0.042197 1.259591

C 3.088245 0.379914 1.095592

C 1.331682 -2.919466 -1.049270

C -0.288092 -1.424051 0.613313

C -0.760625 -1.484846 -0.868010

C -0.100704 -2.667510 -1.564762

C -1.986362 0.481895 0.762524

C -1.246325 -0.619615 1.508704

H 1.293738 -3.747934 -0.320203

H 3.929171 -1.971255 -1.215650

H 1.137148 0.633812 1.954867

H 3.504006 1.211673 1.665698

H -0.703464 -3.576908 -1.404558

H -1.239056 1.206274 0.395983

H -1.977340 -1.314710 1.952556

H -0.316592 -2.471536 0.975351

H -0.094489 -2.499555 -2.653057

H 1.987759 -3.279578 -1.854417

H -2.639041 1.053015 1.436739

H -0.709240 -0.196369 2.370808

H -2.587410 -2.115749 -0.043499

H -0.389934 -0.565142 -1.358653

C -2.596849 0.992834 -1.592611

H -3.228976 0.803965 -2.471403

H -2.851992 1.998352 -1.226115

H -1.553263 1.030961 -1.936709

O 5.188061 -0.114291 -0.040141

C 5.819013 0.924314 0.674255

H 5.305484 1.887066 0.483191

H 5.738917 0.747980 1.763214

C 7.260420 1.025408 0.276376

C 8.120856 1.820297 1.045775

C 7.771965 0.369633 -0.847720

C 9.461839 1.959453 0.697889

H 7.730606 2.332918 1.930317

C 9.117351 0.506826 -1.193812

H 7.111394 -0.255055 -1.451651

C 9.966618 1.301125 -0.425196

H 10.118906 2.582110 1.310351

H 9.503948 -0.013863 -2.073889

H 11.019876 1.405715 -0.697192

**M4l**

C -6.254111 0.907821 -0.374313

C -5.497136 2.103854 -0.341806

C -6.182485 3.331135 -0.408201

C -7.570240 3.395019 -0.469941

C -8.313751 2.217249 -0.451965

C -7.655807 0.993167 -0.409030

C -5.669991 -0.459977 -0.424683

C -5.151436 -0.959824 -1.629014

C -4.673811 -2.267996 -1.708357

C -4.698834 -3.093064 -0.582871

C -5.210594 -2.604401 0.619467

C -5.702208 -1.300916 0.695971

H -5.621652 4.266595 -0.409889

H -8.067219 4.366161 -0.524376

H -9.405233 2.249438 -0.484811

H -8.234505 0.065724 -0.421888

H -5.136725 -0.315969 -2.513453

H -4.283649 -2.644668 -2.657720

H -4.326436 -4.119376 -0.644753

H -5.231115 -3.243249 1.505758

H -6.118124 -0.925587 1.636015

P -3.669422 2.128879 -0.140700

C -2.963723 3.121516 -1.599524

C -3.735237 2.672099 -2.839975

C -3.037802 4.636898 -1.453651

C -1.497487 2.713452 -1.772465

H -3.739530 1.575334 -2.946519

H -4.778552 3.018119 -2.841255

H -3.250363 3.084074 -3.738738

H -2.437680 5.006513 -0.611143

H -2.627654 5.100828 -2.364836

H -4.061547 5.018310 -1.344280

H -1.057067 3.293555 -2.598753

H -0.885098 2.902810 -0.879137

H -1.399922 1.647095 -2.028499

C -3.369948 2.948171 1.546150

C -4.103759 4.261088 1.793259

C -3.843046 1.920080 2.576579

C -1.864023 3.153315 1.699039

H -3.894829 5.032590 1.041090

H -5.191713 4.126184 1.859922

H -3.777755 4.664495 2.765042

H -3.258503 0.989811 2.514400

H -3.714849 2.328318 3.591735

H -4.908779 1.666092 2.459023

H -1.639846 3.451138 2.735242

H -1.297224 2.229567 1.495666

H -1.476852 3.946267 1.043418

Au -2.508408 0.031431 0.002668

C -0.407945 -3.746652 -0.488052

C -1.638177 -2.680699 1.159855

C -1.069381 -3.892742 0.822086

H 0.564595 -4.261672 -0.516153

H -1.020563 -4.260856 -1.250139

C -1.139951 -5.142509 1.584509

H -1.682950 -5.909929 1.009717

H -0.128815 -5.555351 1.726163

H -1.625152 -5.020392 2.558905

O -2.412183 -2.529971 2.283454

C -2.098421 -1.503540 3.139542

O -1.117658 -0.820431 2.997033

C -3.109428 -1.412530 4.222375

H -4.113854 -1.289124 3.795980

H -3.126195 -2.347494 4.798470

H -2.881060 -0.577188 4.890260

C -1.436219 -1.701334 0.171827

C -0.409274 -2.225558 -0.768187

C -0.588451 -1.810277 -2.256922

C 1.039651 -1.616452 -0.438646

C 0.802395 -1.477616 -2.832354

H -1.271032 -0.946861 -2.316452

H -1.081544 -2.618988 -2.815774

C 1.746660 -1.992254 -1.763379

H 0.975101 -1.948153 -3.810710

H 0.923408 -0.392594 -2.982761

C 7.456366 -0.282163 0.276415

C 7.084684 -0.556167 -1.046868

C 5.941017 -1.316383 -1.313617

C 5.158238 -1.816757 -0.252225

C 5.537392 -1.526096 1.060310

C 6.675362 -0.770692 1.330413

C 5.530026 -1.672060 -2.717233

C 3.914360 -2.550172 -0.675193

C 3.211370 -1.608412 -1.691441

C 4.009803 -1.595374 -2.987608

C 1.803007 -2.186629 0.772604

C 2.970032 -3.097617 0.404072

H 5.855987 -2.712221 -2.893592

H 7.678211 -0.184179 -1.884656

H 4.935832 -1.888412 1.898915

H 6.971872 -0.541938 2.356431

H 3.714884 -2.449188 -3.620279

H 2.179346 -1.341557 1.372146

H 2.571600 -4.059182 0.042751

H 4.245390 -3.427864 -1.265024

H 3.765613 -0.691229 -3.567019

H 6.093335 -1.070025 -3.444442

H 1.123486 -2.719982 1.461428

H 3.531721 -3.362696 1.312698

H 1.719431 -3.096017 -1.840554

H 3.273934 -0.587712 -1.273644

C 0.905958 -0.102074 -0.264418

H 0.272122 0.369763 -1.027871

H 0.460963 0.124567 0.715290

H 1.887885 0.392087 -0.290757

O 8.545468 0.447641 0.627073

C 9.340079 1.009843 -0.392203

H 8.721969 1.651979 -1.049183

H 9.754217 0.217169 -1.043405

C 10.450218 1.818180 0.208411

C 10.546911 2.045187 1.584435

C 11.413220 2.380874 -0.640526

C 11.585120 2.822946 2.100396

H 9.802691 1.609112 2.252929

C 12.447069 3.157837 -0.125219

H 11.347080 2.205849 -1.718781

C 12.536804 3.382826 1.250178

H 11.647740 2.991922 3.178556

H 13.189385 3.589682 -0.801267

H 13.347897 3.992643 1.655785

**TS4l**

C 4.694808 2.333083 1.918163

C 4.593717 2.843817 0.604538

C 5.192281 4.087053 0.325445

C 5.871203 4.813146 1.297164

C 5.966777 4.306936 2.591364

C 5.381310 3.082297 2.889083

C 4.122738 1.041461 2.389248

C 2.904687 1.027083 3.084670

C 2.432603 -0.149278 3.664376

C 3.171300 -1.329182 3.557341

C 4.376448 -1.328717 2.854603

C 4.852506 -0.150238 2.279323

H 5.122769 4.506334 -0.677957

H 6.321865 5.774364 1.040181

H 6.494744 4.863996 3.368766

H 5.451674 2.679960 3.903186

H 2.334559 1.954972 3.189131

H 1.489425 -0.140707 4.216920

H 2.809147 -2.247635 4.026455

H 4.959583 -2.249116 2.769138

H 5.812671 -0.147175 1.755083

P 3.695576 1.976906 -0.745990

C 2.488397 3.255695 -1.488276

C 1.865607 4.000740 -0.308397

C 3.088705 4.244482 -2.483083

C 1.384418 2.474214 -2.202028

H 1.468648 3.309494 0.453272

H 2.572979 4.681048 0.185777

H 1.019885 4.607861 -0.667221

H 3.449885 3.750156 -3.395163

H 2.297965 4.945232 -2.794511

H 3.903759 4.855923 -2.076550

H 0.691676 3.182318 -2.683489

H 1.766427 1.804550 -2.986152

H 0.800857 1.868608 -1.496529

C 5.051757 1.456395 -1.965051

C 6.023847 2.552947 -2.384640

C 5.841817 0.371344 -1.232804

C 4.375285 0.853942 -3.194699

H 5.542150 3.403572 -2.882044

H 6.617116 2.932929 -1.541807

H 6.740460 2.126174 -3.104489

H 5.191069 -0.452781 -0.903242

H 6.607084 -0.047334 -1.905409

H 6.361986 0.768907 -0.347316

H 5.134269 0.371439 -3.830561

H 3.637579 0.082372 -2.923779

H 3.871607 1.612927 -3.810253

Au 2.497168 -0.039179 -0.278482

C 0.273402 -3.782328 -1.054587

C 2.397597 -3.106573 -0.530345

C 1.710258 -4.171555 -0.994538

H -0.079824 -3.732284 -2.100663

H -0.415076 -4.487992 -0.559943

C 2.238386 -5.487449 -1.411298

H 1.678590 -6.312552 -0.946630

H 2.144900 -5.627295 -2.500015

H 3.298714 -5.605028 -1.152151

O 3.747979 -3.100149 -0.253210

C 4.630994 -2.992951 -1.293145

O 4.280475 -2.811215 -2.429991

C 6.025084 -3.162984 -0.803116

H 6.208818 -2.559245 0.094601

H 6.179608 -4.212969 -0.515845

H 6.741305 -2.903885 -1.588236

C 1.548018 -1.928659 -0.247474

C 0.228535 -2.384695 -0.511011

C 0.761441 -2.379009 1.330828

C -1.071674 -1.607355 -0.422768

C -0.405669 -1.622825 1.917117

H 1.720621 -2.151918 1.814100

H 0.642574 -3.470870 1.404649

C -1.569573 -1.967564 1.009915

H -0.554730 -1.985115 2.946574

H -0.201702 -0.543887 1.989954

C -7.592036 -0.178228 0.161019

C -6.920514 -0.399303 1.373421

C -5.746640 -1.144246 1.413138

C -5.215404 -1.685818 0.216902

C -5.880783 -1.442418 -0.980699

C -7.066069 -0.698724 -1.024747

C -5.045726 -1.463346 2.704961

C -3.913701 -2.424893 0.376326

C -2.992848 -1.488662 1.206887

C -3.505147 -1.414300 2.639124

C -2.166082 -2.027278 -1.447024

C -3.218834 -2.968444 -0.881758

H -5.344486 -2.488757 2.985367

H -7.345297 0.021153 2.289213

H -5.480987 -1.836451 -1.919196

H -7.559349 -0.534145 -1.983557

H -3.095569 -2.254190 3.224485

H -2.668916 -1.105058 -1.779506

H -2.748146 -3.933790 -0.637576

H -4.114854 -3.297728 1.029525

H -3.125983 -0.499394 3.120606

H -5.428360 -0.825633 3.514214

H -1.718368 -2.447254 -2.360221

H -3.956300 -3.209937 -1.661352

H -1.619979 -3.073226 1.051196

H -3.085600 -0.477190 0.771350

C -0.831308 -0.108919 -0.594841

H -0.095064 0.313958 0.102233

H -0.466914 0.087574 -1.614010

H -1.765649 0.456009 -0.472094

O -8.735302 0.548153 0.239057

C -9.452503 0.797370 -0.950494

H -8.826622 1.355271 -1.671208

H -9.709879 -0.159773 -1.445244

C -10.702808 1.564220 -0.644514

C -11.240328 1.616870 0.645521

C -11.377261 2.214575 -1.686347

C -12.430128 2.304856 0.887323

H -10.718478 1.117025 1.463695

C -12.567485 2.896731 -1.445263

H -10.961585 2.183483 -2.698055

C -13.099022 2.944613 -0.155367

H -12.837085 2.339944 1.901274

H -13.080888 3.399472 -2.268775

H -14.030680 3.482956 0.035927

**M5l**

C -4.438225 -2.053697 -0.247775

C -5.962725 -0.356106 -0.100970

C -5.890953 -1.690807 -0.320333

H -4.240858 -2.810544 0.533949

H -4.096247 -2.527361 -1.186680

C -6.994870 -2.643966 -0.574769

H -6.755525 -3.341083 -1.392610

H -7.217676 -3.267390 0.307788

H -7.927402 -2.126786 -0.841744

O -7.099745 0.430921 -0.123671

C -8.069957 0.233394 0.814675

O -7.980601 -0.579761 1.697656

C -9.208314 1.161761 0.572646

H -8.861127 2.203139 0.591637

H -9.630623 0.990904 -0.426478

H -9.986371 1.020580 1.328415

C -4.648380 0.231735 0.126953

C -3.730867 -0.764833 0.030480

C -4.346545 1.667824 0.402929

C -2.271316 -0.533772 0.313247

C -2.860893 1.908165 0.702320

H -4.980792 2.043303 1.224352

H -4.655067 2.267693 -0.473851

C -1.996172 0.944119 -0.099443

H -2.605073 2.949223 0.453172

H -2.658454 1.807962 1.781536

C 4.283866 0.214615 -0.477677

C 3.566382 1.419422 -0.421247

C 2.215895 1.460526 -0.753035

C 1.549809 0.275640 -1.151665

C 2.272314 -0.913000 -1.194336

C 3.633231 -0.958826 -0.868354

C 1.425806 2.739187 -0.756468

C 0.071544 0.421175 -1.399281

C -0.489868 1.173051 -0.159598

C -0.001667 2.615537 -0.183952

C -1.299306 -1.473268 -0.448408

C -0.728309 -0.848835 -1.708589

H 1.347220 3.064019 -1.809046

H 4.092277 2.326227 -0.109379

H 1.777645 -1.844240 -1.484387

H 4.164168 -1.910449 -0.915425

H -0.683384 3.229561 -0.796335

H -0.460166 -1.729180 0.221833

H -1.553529 -0.599109 -2.395088

H -0.055896 1.111475 -2.257427

H -0.046411 3.041133 0.830917

H 1.988664 3.538332 -0.253557

H -1.792825 -2.433270 -0.669515

H -0.113601 -1.575447 -2.261419

H -2.354467 1.042938 -1.143241

H -0.038465 0.692197 0.729147

C -2.050295 -0.773484 1.816626

H -2.766338 -0.228163 2.447201

H -2.174311 -1.843024 2.046754

H -1.033203 -0.493416 2.135471

O 5.594986 0.283308 -0.129736

C 6.346167 -0.909686 -0.140896

H 5.875525 -1.669493 0.510102

H 6.354260 -1.344530 -1.159822

C 7.749158 -0.645826 0.314480

C 8.287077 0.643917 0.368997

C 8.558528 -1.732537 0.672145

C 9.608952 0.840360 0.772126

H 7.663513 1.497918 0.097906

C 9.878544 -1.536311 1.069207

H 8.143832 -2.744633 0.638879

C 10.409469 -0.246157 1.121355

H 10.015422 1.854323 0.812930

H 10.495534 -2.395071 1.345388

H 11.444149 -0.089391 1.436314

**M11a**

Cartesian coordinates

C -3.167564 1.692950 0.513923

C -4.160451 0.640179 -1.517929

C -4.454364 1.608080 -0.332774

C -5.404475 -0.251292 -1.678021

C -6.112754 -0.081150 -0.370881

C -5.604519 0.924445 0.352674

H -3.364877 1.698997 1.596702

H -2.584073 2.606079 0.308313

H -6.038415 0.082068 -2.517643

H -5.151118 -1.300398 -1.903606

H -6.971871 -0.691695 -0.080686

H -5.982355 1.256531 1.323772

H -4.756962 2.612566 -0.673167

H -3.916084 1.166137 -2.456120

C -2.939297 -0.095445 -1.055095

C -2.349435 -1.239349 -1.617458

C -1.242342 -0.027011 0.656269

C -1.210649 -1.768174 -1.056272

H -2.775955 -1.733081 -2.491734

C -0.540769 -2.994908 -1.549019

H 0.499878 -2.812325 -1.852540

H -0.513829 -3.779481 -0.778190

H -1.084550 -3.387327 -2.413792

O -0.780879 0.471301 1.751064

C -2.402075 0.492703 0.074329

N 0.505146 -1.633313 0.610345

N -0.667816 -1.129008 0.054561

C 1.730931 -1.191523 0.014767

C 1.705774 -0.513714 -1.210659

C 2.962500 -1.397199 0.653519

C 2.882867 -0.069795 -1.801623

H 0.767078 -0.289736 -1.717781

C 4.139919 -0.953185 0.062427

H 3.009664 -1.888642 1.619471

C 4.098147 -0.297445 -1.164907

H 2.845549 0.464651 -2.751783

H 5.092205 -1.112913 0.570340

Cl 5.577630 0.261913 -1.899006

C 0.361869 -2.644656 1.591207

O 1.325421 -3.254499 1.994172

C -1.032697 -2.870271 2.089598

H -1.442209 -1.964219 2.558166

H -1.728152 -3.169495 1.293526

H -0.998739 -3.663573 2.841915

H 1.458763 0.985188 3.428203

H 0.190043 0.290836 2.047374

H 2.074787 0.881447 2.034721

B 1.381376 2.803875 0.486529

F 0.494183 2.124472 -0.359923

F 2.502883 1.956043 0.748816

F 1.829097 3.969019 -0.121622

F 0.762144 3.087238 1.704777

O 1.571064 0.358491 2.696986

**H_2_O**

Cartesian coordinates

O 0.000000 0.000000 0.121193

H 0.000000 0.751216 -0.484770

H 0.000000 -0.751216 -0.484770

**M7b**

Cartesian coordinates

C 4.239322 0.191648 -1.116008

C 4.246326 -1.407984 0.821410

C 4.867782 -1.123351 -0.586646

C 3.913472 -2.915086 0.873477

C 3.958791 -3.310878 -0.568011

C 4.472757 -2.354390 -1.350757

H 3.982185 0.145603 -2.184075

H 4.916313 1.051568 -0.995864

H 4.660656 -3.468615 1.465421

H 2.943865 -3.117692 1.355748

H 3.642927 -4.299008 -0.911920

H 4.633040 -2.442667 -2.428459

H 5.965569 -1.030908 -0.549380

H 4.899045 -1.116832 1.664372

C 3.065206 -0.522396 0.837082

C 1.923081 -0.339258 1.739424

C 1.887251 1.101254 -0.169845

C 1.045959 0.732899 1.047082

C 0.788957 1.956196 1.904416

H 0.226703 2.732751 1.368878

H 0.202833 1.676995 2.789208

H 1.740086 2.389286 2.241369

O 1.641652 2.072197 -0.998749

C 0.320647 2.638614 -1.264382

O -0.588164 1.895242 -1.422197

C 0.435767 4.094906 -1.398175

H 1.151895 4.325832 -2.198868

H -0.538961 4.531989 -1.627647

H 0.842649 4.529240 -0.475760

C 3.050486 0.339967 -0.238824

N -1.238585 0.470264 0.979675

N -0.162929 -0.001875 0.573386

C -2.402484 -0.195425 0.542133

C -2.418042 -1.114139 -0.519516

C -3.592763 0.138260 1.201256

C -3.611622 -1.699597 -0.908708

H -1.488186 -1.344662 -1.042152

C -4.791388 -0.457317 0.828380

H -3.565153 0.865327 2.016391

C -4.788687 -1.371168 -0.225662

H -3.642973 -2.404462 -1.740534

H -5.721256 -0.213999 1.343270

Cl -6.282837 -2.109403 -0.716387

H 2.259837 -0.002279 2.734023

H 1.380029 -1.280854 1.918174

O -1.969524 3.405879 0.781267

H -2.392173 3.279828 -0.078386

H -1.816719 2.491604 1.072025

**TS7b**

Cartesian coordinates

C 4.265341 0.738147 -0.936558

C 4.547495 -1.151424 0.695968

C 5.132767 -0.504167 -0.605874

C 4.532038 -2.675604 0.467297

C 4.677602 -2.792779 -1.016907

C 5.005821 -1.629379 -1.592371

H 4.044204 0.827228 -2.011061

H 4.760610 1.678380 -0.645832

H 5.368098 -3.170960 0.989094

H 3.618307 -3.153697 0.856497

H 4.566317 -3.743090 -1.545489

H 5.198155 -1.488929 -2.659687

H 6.190820 -0.213345 -0.493107

H 5.110718 -0.888967 1.609364

C 3.204586 -0.525757 0.777530

C 2.003354 -0.781082 1.594740

C 1.743535 1.039020 0.007554

C 1.032724 0.355588 1.202488

C 0.869829 1.376011 2.318078

H 0.360971 2.287101 1.983291

H 0.295212 0.941146 3.146710

H 1.856833 1.668867 2.699921

O 1.284365 1.966438 -0.693079

C -0.440302 2.456943 -1.336938

O -1.054307 1.528020 -1.615103

C -0.175814 3.855532 -1.553153

H 0.537067 3.923884 -2.387261

H -1.122930 4.332079 -1.834220

H 0.256857 4.325093 -0.667529

C 3.054411 0.497050 -0.108091

N -1.258456 0.242075 1.124955

N -0.200360 -0.265200 0.701087

C -2.455545 -0.328818 0.639676

C -2.509425 -1.302210 -0.371391

C -3.641219 0.160293 1.203357

C -3.733932 -1.780589 -0.808171

H -1.580502 -1.664911 -0.814274

C -4.874347 -0.315933 0.774857

H -3.583740 0.916453 1.990883

C -4.908379 -1.282354 -0.230373

H -3.790950 -2.531449 -1.597854

H -5.801498 0.057643 1.210528

Cl -6.444039 -1.874653 -0.785784

H 2.215468 -0.795782 2.675081

H 1.574007 -1.769189 1.360099

O -1.517982 3.077712 0.821713

H -2.447710 3.270453 0.632118

H -1.545780 2.194675 1.240190

**M8b**

Cartesian coordinates

C 3.992284 0.793167 -1.266006

C 4.740420 -0.885564 0.450009

C 5.055232 -0.296364 -0.965790

C 4.863035 -2.419791 0.330833

C 4.842669 -2.648809 -1.145737

C 4.954563 -1.511244 -1.842133

H 3.608282 0.739455 -2.296071

H 4.394554 1.811241 -1.141787

H 5.802744 -2.786262 0.777561

H 4.059198 -2.952148 0.865377

H 4.782760 -3.647935 -1.585261

H 4.994158 -1.444286 -2.932836

H 6.068743 0.134770 -1.026488

H 5.401151 -0.495224 1.243594

C 3.357089 -0.397662 0.692953

C 2.350886 -0.693426 1.738945

C 1.614748 0.945134 0.015089

C 1.185535 0.248108 1.411461

C 0.996566 1.382400 2.409539

H 0.327352 2.165244 2.029271

H 0.599062 0.996941 3.358246

H 1.963911 1.860138 2.608280

O 0.859886 1.692834 -0.558878

C -1.621876 3.172165 -0.718614

O -2.128094 2.258544 -1.314061

C -1.013137 4.391393 -1.317378

H -1.065440 4.346195 -2.408917

H -1.535951 5.292806 -0.967736

H 0.036924 4.483804 -1.008304

C 2.953457 0.497861 -0.241922

N -1.051709 0.135700 0.935953

N 0.024841 -0.498631 1.081801

C -2.241264 -0.482281 0.522467

C -2.244955 -1.803825 0.058149

C -3.423407 0.263651 0.609395

C -3.444212 -2.381905 -0.318114

H -1.310909 -2.361924 -0.010932

C -4.625886 -0.318437 0.239770

H -3.398269 1.292451 0.970792

C -4.626974 -1.636522 -0.221646

H -3.471572 -3.406339 -0.689348

H -5.556496 0.245278 0.305886

Cl -6.123134 -2.374201 -0.684839

H 2.745080 -0.525132 2.753745

H 2.034554 -1.747958 1.709122

O -1.593547 3.114608 0.644824

H -1.172436 3.905619 1.022753

H -1.120640 1.159003 1.118366
